# Clinical decision support in hematological malignancies using a case-grounded AI agent

**DOI:** 10.1038/s41591-026-04494-4

**Published:** 2026-06-30

**Authors:** Julian Zoller, Michael Kalz, Xuewei Wu, Sven Cuntz, Linus Kruk, Niklas Kehl, Julius J. Michel, Cornelius Funk, Sarah Richter, Tobias Tix, David Sedloev, Jonathan Naboschni, Jan H. Frenking, Silvia Barbosa, Oliver L. Saldanha, Alanna Kirschner, Anna D. Metzler, Antonia Schach, René Onken, Tim R. Wagner, Martin Dugas, Andreas Trumpp, Martin Dreyling, Michael von Bergwelt-Baildon, Kai Rejeski, Tim Sauer, Peter Dreger, Marc S. Raab, Carsten Müller-Tidow, Jakob N. Kather, Mirco J. Friedrich

**Affiliations:** 1 JRG Hematology and Immune Engineering, German Cancer Research Center (DKFZ), Heidelberg, Germany; 2 Medical Faculty, University of Heidelberg and Department of Hematology, Oncology and Rheumatology, Heidelberg University Hospital, Heidelberg, Germany; 3 Heidelberg Institute for Stem Cell Technology and Experimental Medicine (HI-STEM), Heidelberg, Germany; 4 German Cancer Consortium (DKTK), Core Center Heidelberg, Heidelberg, Germany; 5 Department of Medical Oncology, National Center for Tumor Diseases, Heidelberg University Hospital, Heidelberg, Germany; 6 Department of Radiology, The First Affiliated Hospital of Jinan University, Guangzhou, China; 7 Department of Medicine III–Hematology/Oncology, LMU University Hospital, LMU Munich, Munich, Germany; 8 Else Kroener Fresenius Center for Digital Health, Faculty of Medicine, TUD Dresden University of Technology, Dresden, Germany; 9 Medical Faculty, Heidelberg University, Institute of Medical Informatics, Heidelberg, Germany; 10 Department of Medicine I, Faculty of Medicine, TUD Dresden University of Technology, Dresden, Germany; 11 Pathology and Data Analytics, Leeds Institute of Medical Research at St James’s, University of Leeds, Leeds, UK

**Keywords:** Translational research, Data integration, Machine learning

## Abstract

Multidisciplinary tumor boards integrate longitudinal treatment histories, molecular profiling and rapidly evolving evidence to guide decisions in hematological malignancies, yet access to this level of subspecialty deliberation is increasingly uneven. Here we develop HemaGuide, a locally deployable, modular large language model agent that converts unstructured clinical documents into structured case representations, autonomously routes cases to specialized decision modes (‘guideline’, ‘advanced’ and ‘molecular’) and grounds recommendations in disease-specific guideline flowcharts and a clinical decision memory of >2,000 real-world tumor board cases. In expert-blinded benchmarking on 45 high-complexity cases across six foundation models, HemaGuide substantially improved concordance with tumor board decisions. A systematic ablation study across 11 layers confirmed that performance gains were routing-type-dependent, with no single component sufficient across case types. Automated classification of 70 clinically relevant missense variants showed high concordance with expert standards; no oncogenic variant was downgraded to benign and the whole workflow was completed under real-time conditions on commodity hardware with a median latency of 39 s rather than the hours typically required for manual molecular board workflows. In a simulated practice study, agent-assisted resident physicians achieved near-senior concordance and partially outperformed senior physicians in their subspecialty. External validation on 555 independent cases from a second academic center yielded 81.8% concordance across 47 entities, and a prospective 1-month silent trial on 64 consecutive, unselected cases achieved 82.8% concordance. Hallucinations occurred in 2 of 664 evaluated cases (0.3%). Together these data provide evidence that locally deployable, case-grounded large language model agents can deliver auditable clinical decision support across hematological malignancies, with concordance maintained across institutions and under real-time conditions on commodity hardware.

## Abstract (web summary)

A locally deployable, case-grounded large language model agent achieved high concordance with hematology tumor board decisions across retrospective, external and prospective evaluations.

## Main

Hematological malignancies increasingly demand tumor-board-level reasoning: beyond entity and stage, clinicians must integrate cytogenetics and molecular lesions of the disease, comorbidities and competing risks of the patient, prior toxicities and, especially after multiple lines of therapy or at the end of standard-of-care, the rapidly shifting evidence base for off-label, clinical trial and precision oncology options^1,2^. In response, many tertiary centers rely on multidisciplinary (and increasingly molecularly guided) tumor boards in which multiple specialists synthesize heterogeneous data to determine a best-available treatment plan—a workflow that, in principle, is well aligned with large language model (LLM)-based clinical agents designed to retrieve, orchestrate and justify information across sources^3^.

Yet, this level of deliberation is time-consuming and resource-intensive. With rising population age, incidence rates and a rapidly expanding therapeutic landscape, it is increasingly difficult to convene subspecialty tumor boards at every clinical decision point, a constraint that disproportionately affects nontertiary centers where hematology expertise may be limited to one or two physicians covering the full disease spectrum. This gap is further compounded by growing economic pressures on health systems worldwide^4^.

While early LLM applications focused on well-defined clinical tasks, recent evidence, particularly in specialized fields such as hematology and oncology, demonstrates their growing capability to support complex decision-making and longitudinal patient care^5–7^. However, ensuring the consistent clinical reliability and seamless integration of these models into real-world hematological workflows remains a critical area of active development. A key safety concern is hallucination: fabricated or erroneous outputs; and even when language is fluent, failures in numerical or structured reasoning could be clinically detrimental. Recent evidence suggests these limitations can be mitigated when LLMs are not used as stand-alone chatbots but instead embedded as reasoning engines within systems that ground outputs in authoritative evidence and execute task-specific steps through tools by combining retrieval with structured tool use to improve completeness, accuracy and traceability^3,8^.

Consistent with this ‘agentic’ paradigm, where the bottleneck is not a single prediction but the integration of diverse clinical data into a coherent action plan, agents can actively gather missing information, query knowledge repositories and synthesize evidence into an actionable treatment recommendation^9^.

## Results

### A case memory-grounded agent architecture for hematology tumor board decision support

**Fig. 1:**
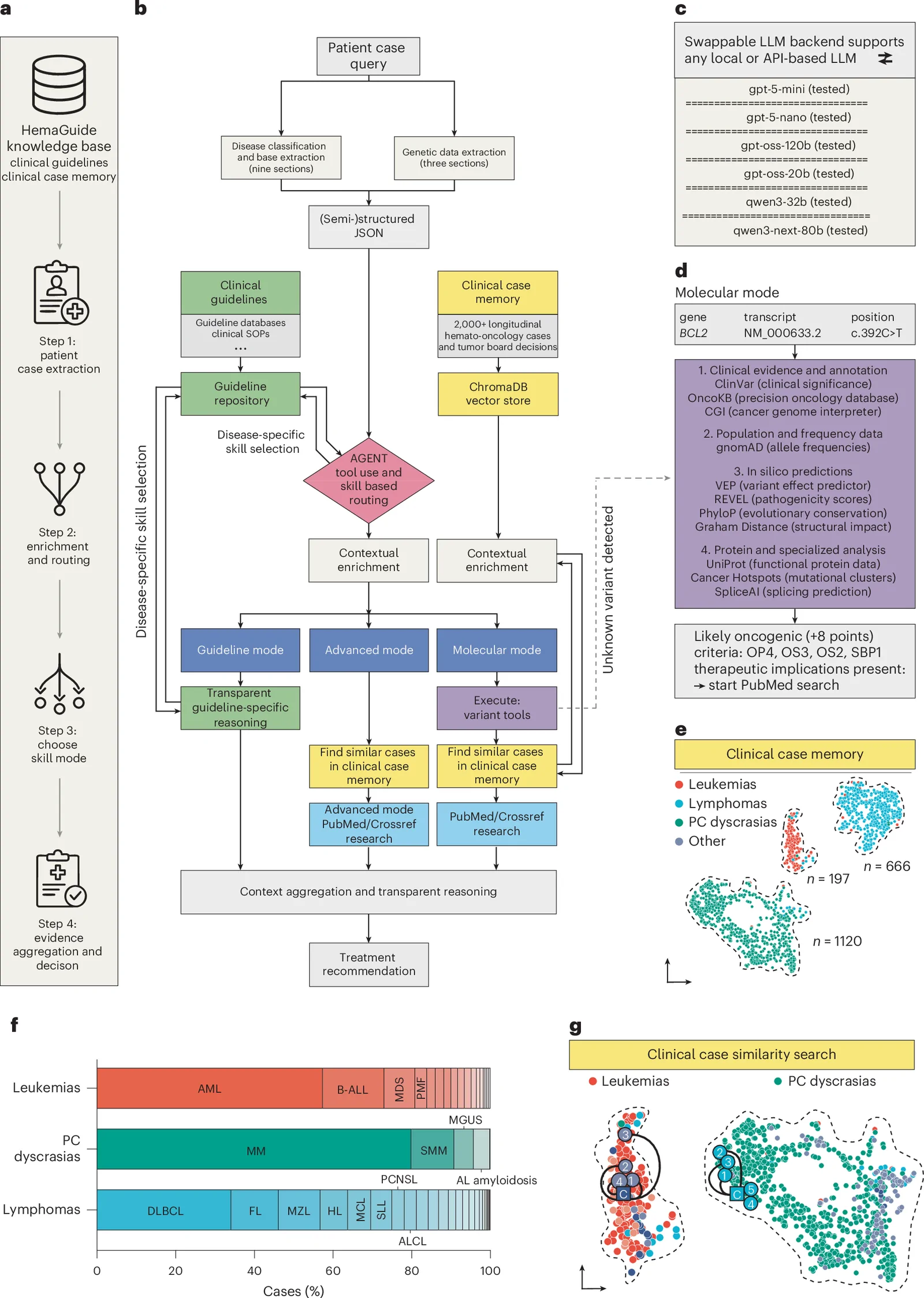
HemaGuide architecture and construction of a clinical case memory for malignant hematology. **a**, A four-step overview of the HemaGuide workflow: (step 1) case extraction from routine clinical documents, (step 2) enrichment and routing, (step 3) mode selection and (step 4) evidence aggregation into an auditable treatment recommendation. **b**, A system schematic illustrating the conversion of unstructured patient material into a semi-structured JSON case representation (nine clinical sections; optional molecular layers), contextual enrichment and agentic routing into one of three decision modes: guideline mode (clinical guideline and SOP-guided reasoning), advanced mode (retrieval of clinically similar longitudinal cases from the clinical case memory plus targeted literature) and molecular mode (variant interpretation and targeted PubMed and Crossref retrieval), followed by context aggregation into a transparent treatment recommendation. **c**, Swappable LLMs enabling deployment with multiple local or API-based LLMs (tested backbones listed). **d**, The molecular mode evidence stack: variant evidence and annotation, population frequency, in silico predictors and protein/specialized analyses; actionable or likely oncogenic findings trigger targeted PubMed and Crossref retrieval. **e**, An embedding-based visualization of the clinical case memory showing clustering by major hematological diagnostic groups with case counts. **f**, The relative disease composition of the clinical case memory across leukemias, PC dyscrasias and lymphomas. **g**, An embedding-based visualization of two representative query cases (‘C’) and their five highest-ranked similar cases (circles) identified via the advanced mode similarity search within the clinical case memory dataset. ALCL, anaplastic large cell lymphoma; AML, acute myeloid leukemia; B-ALL, B-cell acute lymphoblastic leukemia; DLBCL, diffuse large B-cell lymphoma; FL, follicular lymphoma; HL, Hodgkin lymphoma; MCL, mantle cell lymphoma; MDS, myelodysplastic syndrome; MGUS, monoclonal gammopathy of undetermined significance; MM, multiple myeloma; MZL, marginal zone lymphoma; PCNSL, primary central nervous system lymphoma; PMF, primary myelofibrosis; SLL, small lymphocytic lymphoma; SMM, smoldering multiple myeloma. AL amyloidosis, amyloid light-chain amyloidosis.

**Extended Data Fig. 1:**
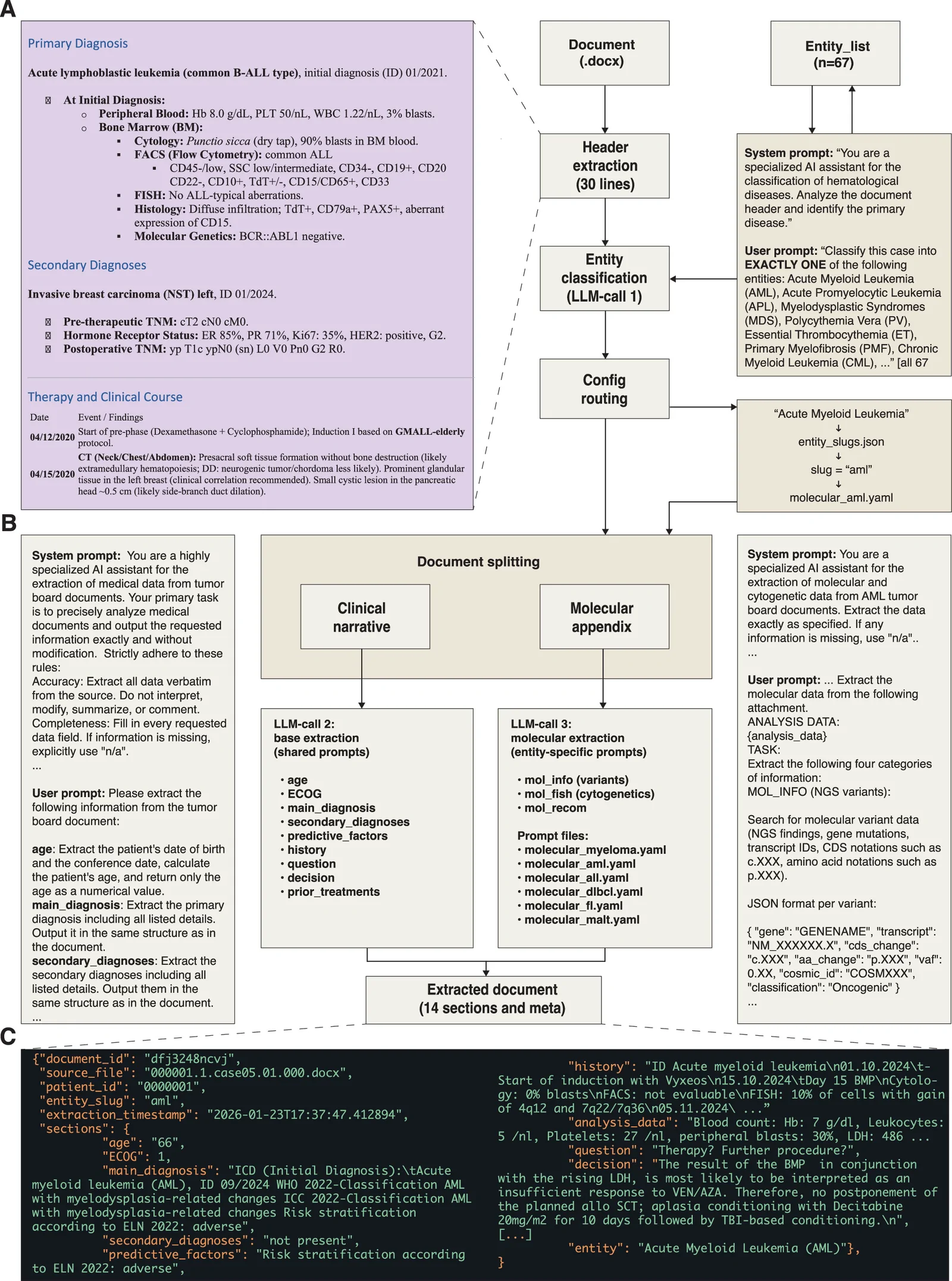
Prompt- and schema-driven architectural framework for automated entity routing and structured document extraction. (**a**) Schematic of the *HemaGuide* extraction pipeline. Unstructured clinical documents are processed via a 30-line header extraction to provide context for supervised entity classification (LLM call 1), which assigns the case to one of n = 67 entities. This classification enables config routing, mapping the entity to its matching YAML configuration file. (**b**) The source document is bifurcated into a clinical narrative and a molecular appendix for parallel processing. Base extraction (LLM call 2) utilizes shared prompts across all entities to capture core clinical parameters such as primary/secondary diagnoses, disease history and prior therapies, while molecular extraction (LLM call 3) employs entity-specific schemas for high-resolution data including mutational variants and cytogenetics. System prompts enforce strict verbatim extraction and mandatory “n/a” reporting for missing fields to ensure data integrity. (**c**) The process culminates in a structured JSON document comprising 12 content sections and comprehensive metadata, facilitating transparent clinical auditing and downstream decision support.

We hypothesized that clinically reliable decision support in hematological malignancies requires grounding not only in guidelines but also in a structured repository of real-world patient trajectories that captures the sequence-dependent treatment histories, toxicity constraints and feasibility considerations shaping late-line cancer care^3,8^. We therefore developed HemaGuide, an openly available clinical decision support architecture that converts routine unstructured tumor board briefing or case summaries into a semi-structured case representation spanning nine clinical sections with optional molecular layers, performs contextual enrichment and autonomous routing, and invokes one of three specialized decision tools (‘guideline’, ‘advanced’ and ‘molecular’) before aggregating context into a conference-style recommendation with an auditable rationale (Fig. 1a,b and Extended Data Fig. 1). Documents are ingested manually via a graphical interface. The architecture is model-agnostic through a swappable backbone (Fig. 1c), enabling deployment on local infrastructure so that model choice can be optimized for cost, latency and regulatory constraints without altering the clinical logic.

Central to HemaGuide are two curated knowledge bases: a guideline and standard operating procedure (SOP) repository, encoded as disease-specific decision flowcharts, and a clinical case memory comprising >2,000 real-world hematology tumor board case discussions accrued across 2024–2025, spanning leukemias (*n* = 197), lymphomas (*n* = 666) and plasma cell (PC) dyscrasias (*n* = 1,120) (Fig. 1e,f). Notably, the relative disease frequencies broadly mirror real-world incidence patterns (for example, AML (acute myeloid leukemia) and B-ALL (B-cell acute lymphoblastic leukemia) among leukemias and DLBCL (diffuse large B-cell lymphoma) among lymphomas), while we intentionally enriched the dataset for rare, high-complexity entities, most prominently AL amyloidosis, to reflect the disproportionate clinical reasoning burden these cases impose (Fig. 1f). The corpus is deliberately enriched for clinical settings in which expert consensus is most often required: newly diagnosed cases (*n* = 907), relapsed/refractory cases (*n* = 665), immunotherapeutic cases including CAR-T cell therapy (*n* = 344) and cases with personalized molecular diagnostics (*n* = 107). In parallel, a molecular interpretation toolkit operationalizes the ClinGen (Clinical Genome Resource)/CGC (Cancer Genomics Consortium)/VICC (Variant Interpretation for Cancer Consortium) point-based standards for somatic variant classification and couples automated evidence retrieval to targeted PubMed searches triggered only for variants classified as (probably) oncogenic (Fig. 1d).

**Extended Data Fig. 2:**
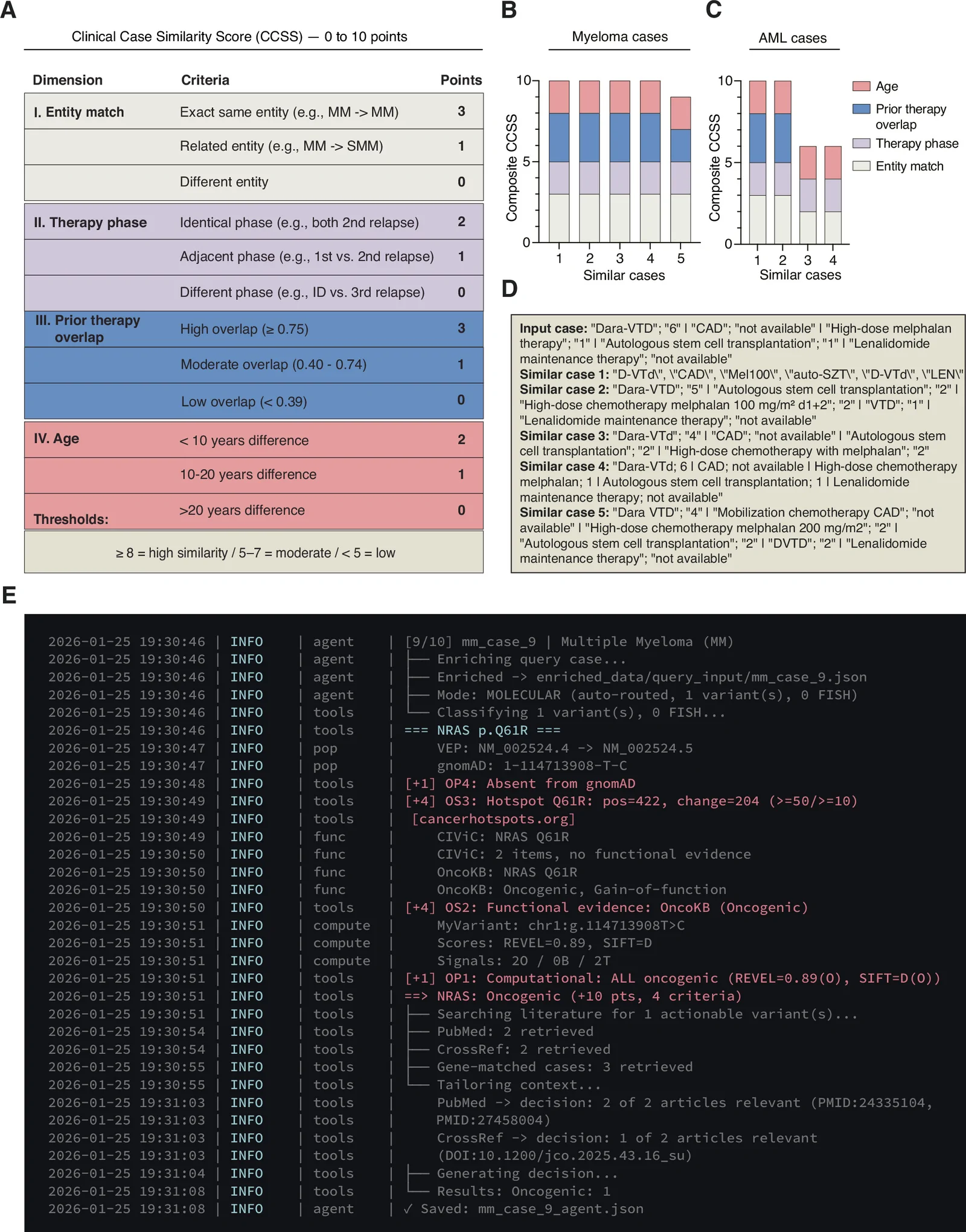
Vector-based case similarity retrieval and automated variant interpretation workflow. (**a**) Scoring criteria of the Clinical Case Similarity Score: a 10-point composite metric scoring entity match (0-3), therapy phase (0-2), prior therapy overlap (0-3) and age (0-2); high similarity defined as total score ≥8/10, moderate similarity as 5-7/10 and low similarity as <5/10. (**b**) Composite CCSS for the five highest-ranked similar cases retrieved for a representative multiple myeloma query case. Retrieved cases are ordered by descending similarity score (rank 1-5), with each bar representing one case. (**c**) Composite CCSS for the five highest-ranked similar cases retrieved for a representative AML query case. (**d**) Prior therapy histories of the multiple myeloma query case and its five top-ranked similar cases listing therapy name and number of cycles administered (or “not available” where cycle count was not documented). Cases are shown in order of decreasing similarity rank (1-5). (**e**) Representative execution log for a multiple myeloma query case (mm_case_9) illustrating the Molecular Mode automated analysis pipeline. The system classifies the NRAS p.Q61R variant as oncogenic (+10 points) based on four ClinGen/CGC/VICC criteria: population frequency (OP4, absent from gnomAD), hotspot recurrence (OS3, cancerhotspots.org), functional evidence (OS2, OncoKB gain-of-function annotation) and computational prediction consensus (OP1, REVEL = 0.89, SIFT = deleterious). This classification triggers targeted literature retrieval across PubMed and CrossRef, with relevance filtering retaining three articles for the final treatment recommendation.

When the input document does not contain a recognizable hematological entity, the system assigns a generic fallback classification and routes the case to advanced mode, which retrieves clinically similar cases across all disease groups rather than forcing the case into entity-specific guideline pathways (Methods). To enable precedent-based reasoning, HemaGuide performs an embedding-based similarity search over the clinical case memory. Using a ten-point metric across four dimensions (entity match, therapy phase, prior therapy overlap and patient age; similarity defined as ≥8/10) across all three entity groups, two expert hematologists confirmed that the top retrieved cases were clinically similar and useful for informing treatment reasoning (Extended Data Fig. 2b,c and Supplementary Table 3). When no cases exceed the configured similarity threshold, the system degrades to plain LLM generation and transparently reports the absence of grounding evidence to the user.

### Tool-augmented case memory grounding improves guideline compliance and patient-context integration

**Fig. 2:**
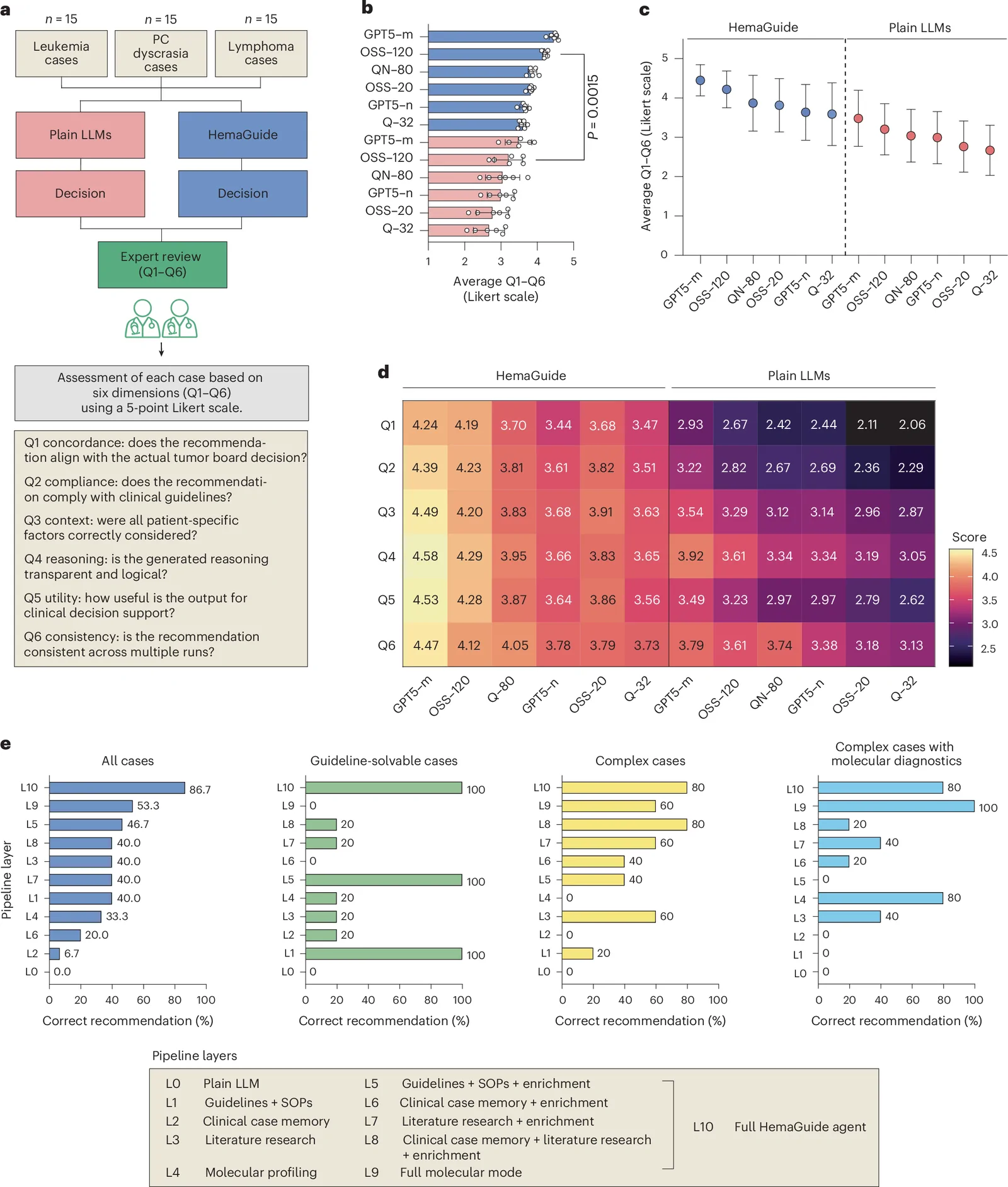
Tool-augmented case grounding improves expert-rated quality and reduces variability compared with plain LLMs. **a**, The benchmark design: plain LLMs versus the same backbones embedded in the HemaGuide pipeline were prompted with 45 complex malignant hematology patient cases (15 leukemia, 15 PC dyscrasia and 15 lymphoma). Outputs were evaluated by blinded expert hematologists using six 5-point Likert dimensions: Q1, tumor -board concordance; Q2, guideline compliance; Q3, patient-context integration; Q4, reasoning quality transparency; Q5, clinical utility; and Q6 run-to-run consistency. **b**, Aggregate performance (mean Q1–Q6) across models, comparing average expert scores for HemaGuide*-*embedded systems relative to their plain counterparts. Statistical significance was determined by an unpaired *t*-test. Data are mean ± s.d. Aggregate performance (mean Q1–Q6) across models, comparing average expert scores for HemaGuide-embedded systems relative to their plain counterparts. Each data point represents the mean score for one Likert dimension (Q1–Q6) averaged across all 45 independent patient cases (15 leukemia, 15 PC dyscrasia and 15 lymphoma; each case corresponding to one unique patient and one tumor board decision) and four blinded expert hematologist evaluators per case (*n* = 6 dimensions per model configuration). Statistical significance was determined by a two-tailed paired *t*-test. Data are mean ± s.d. **c**, The case-level dispersion of expert scores demonstrating reduced variability (more homogeneous output quality) for HemaGuide (gpt-oss-120b) compared with the corresponding plain LLM. Each data point represents one independent patient case (*n* = 45 independent patient cases per configuration: 15 leukemia, 15 PC dyscrasia and 15 lymphoma), with the score per case calculated as the mean across all six Likert dimensions and four blinded expert hematologist evaluators. Data are mean ± s.d. Case-level dispersion of expert scores demonstrate reduced variability (more homogeneous output quality) for HemaGuide (gpt-oss-120) compared with plain LLMs. Data are mean ± s.d. **d**, A dimension-wise performance heat map (Q1–Q6) across model configurations. Average scores are shown. **e**, A systematic ablation analysis quantifying the contribution of individual pipeline components to tumor board concordance. In total, 15 cases (five complexity: solvable by guidelines only, complex cases and cases with molecular diagnostics) were processed at 11 ablation levels—from a plain LLM baseline (L0; no tools or retrieval) through isolated components (guideline + SOP flowcharts, clinical case memory, literature and the molecular toolkit) and progressively enriched combinations to the full agent with autonomous routing (L10)—and independently scored by two independent hematologists (inter-rater reliability: Cohen’s *κ* = 0.849). A correct recommendation was defined as a mean TBCS ≥8/10 across both raters. Left: overall concordance across all 15 cases, sorted by ascending performance, showing a progression from 0% (plain LLM) to 87% (full agent). Right: concordance stratified by case type revealing that performance gains are routing-dependent: guideline cases (second from left) are fully resolved by the flowchart component alone (100%); advanced cases (third from left) require the combination of clinical case memory, literature and contextual enrichment (80%); and molecular cases (right) depend on the molecular interpretation toolkit (100% with the full molecular pipeline). The three highest-performing layers per case type are indicated.

**Extended Data Fig. 3:**
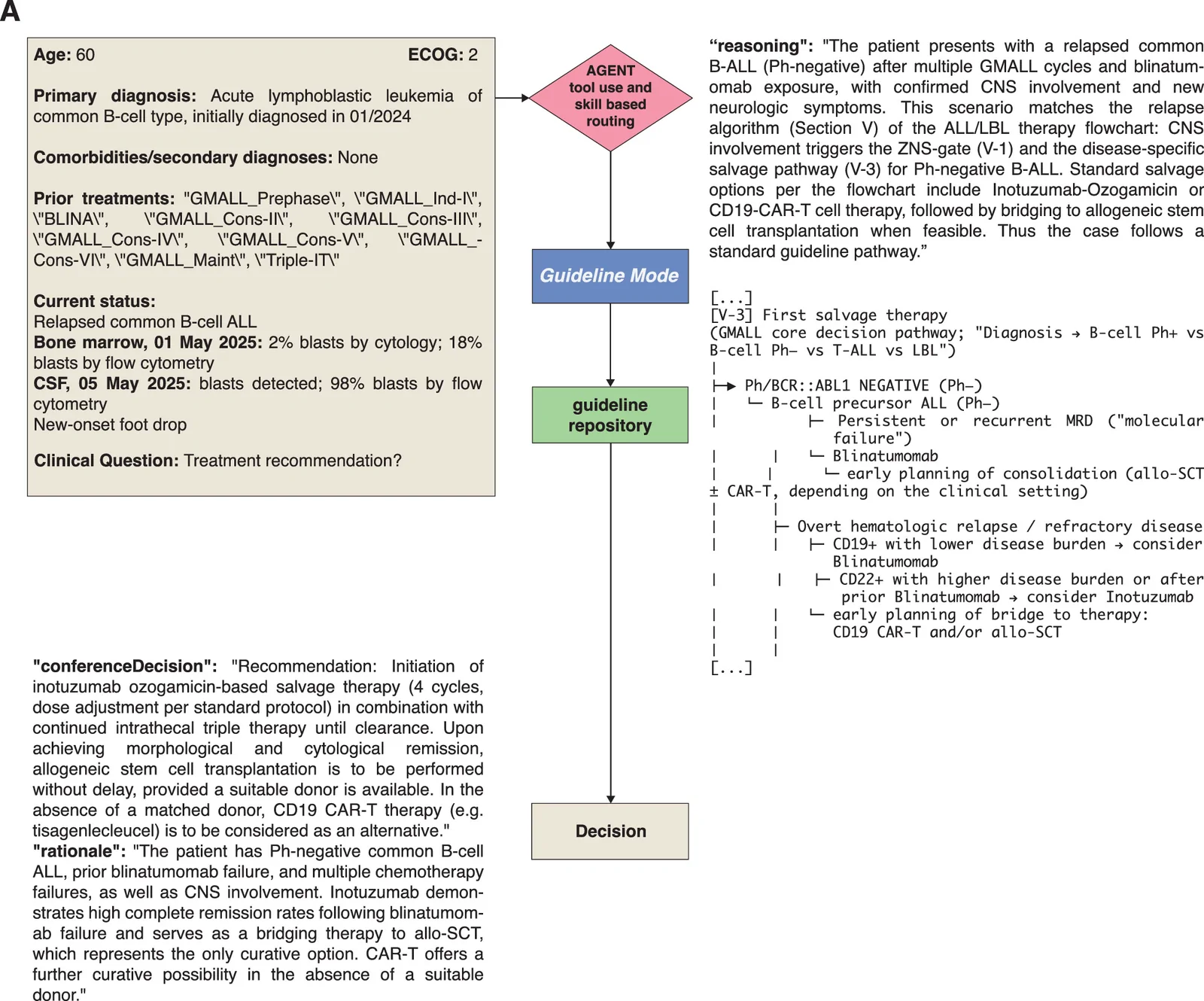
End-to-end *Guideline Mode* case example: autonomous flowchart traversal for relapsed B-ALL. (**a**) Representative case demonstrating *Guideline Mode* execution for a 60-year-old patient with relapsed common B-cell ALL (Ph-negative) after multiple GMALL chemotherapy cycles and blinatumomab, with confirmed CNS involvement and new neurological symptoms (foot drop). The structured case representation (left) shows extracted clinical parameters including age, ECOG status, prior treatment lines and the clinical question. The agent autonomously routes the case to *Guideline Mode*, identifies the applicable decision pathway within the ALL/LBL treatment flowchart (section V, relapse algorithm), and traverses the flowchart nodes for Ph-negative B-ALL with overt relapse after prior blinatumomab exposure. The reasoning trace (right) documents the flowchart traversal logic and the resulting recommendation: inotuzumab ozogamicin-based salvage therapy with bridging to allogeneic stem cell transplantation, or CD19 CAR-T cell therapy if no suitable donor is available - concordant with the actual tumor board decision.

**Extended Data Fig. 4:**
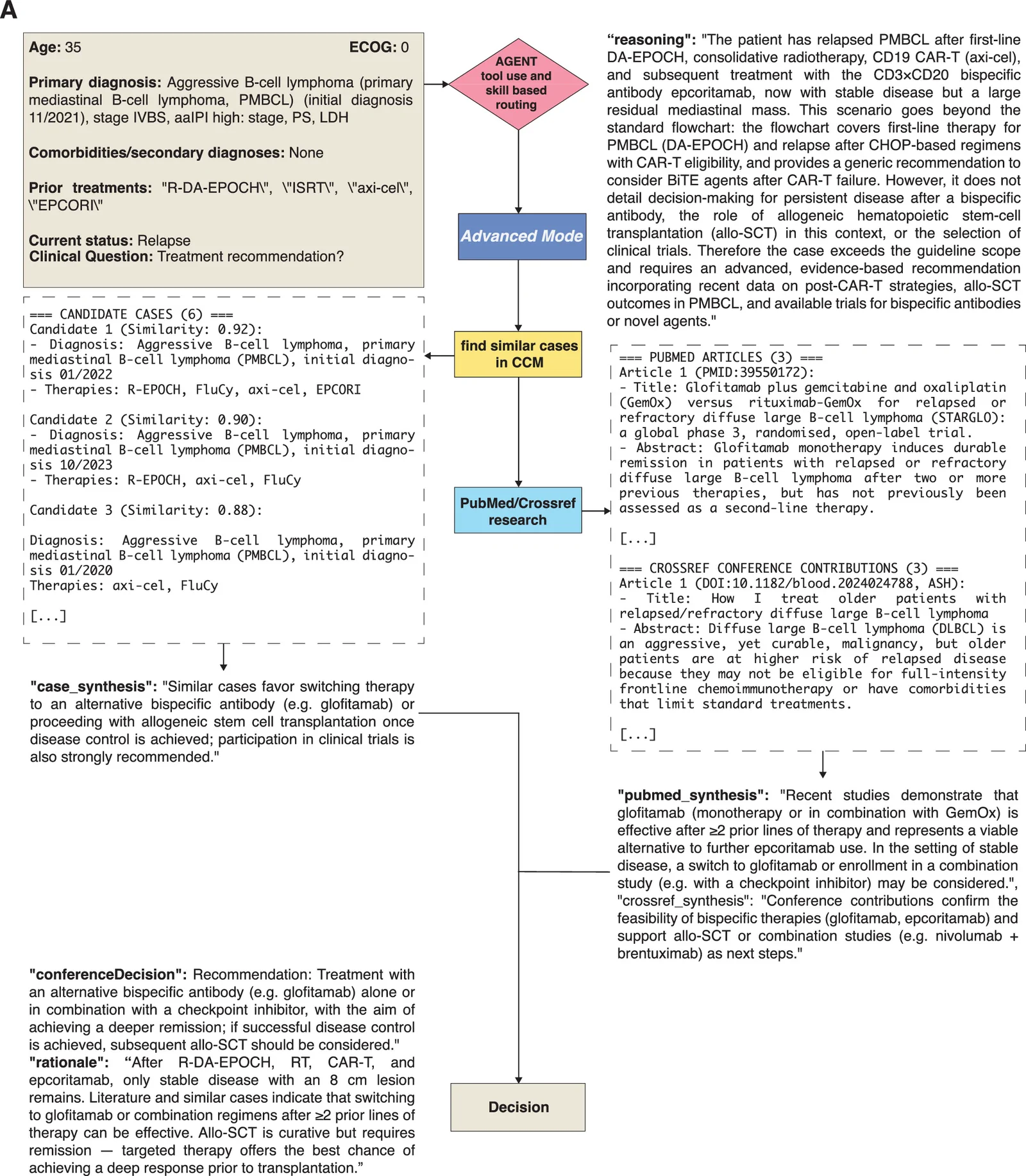
End-to-end *Advanced Mode* case example: multi-source evidence synthesis for relapsed primary mediastinal B-cell lymphoma beyond guideline coverage. (**a**) Representative case demonstrating *Advanced Mode* execution for a 35-year-old patient with relapsed primary mediastinal B-cell lymphoma (PMBCL) after R-DA-EPOCH, consolidative radiotherapy, CAR-T cell therapy (axi-cel) and the bispecific antibody epcoritamab, with persistent stable disease and a residual mediastinal mass. The agent determines that the case exceeds guideline scope and autonomously routes to *Advanced Mode*, which retrieves clinically similar cases from the Clinical Decision Memory (top candidates at cosine similarity 0.88-0.92, all with comparable PMBCL treatment trajectories) and conducts targeted PubMed and CrossRef literature searches. The case synthesis, literature synthesis and conference abstract synthesis are shown alongside the retrieved evidence. The resulting recommendation: switch to an alternative bispecific antibody (glofitamab) with subsequent allogeneic stem cell transplantation upon disease control, is concordant with the actual tumor board decision and supported by both precedent cases and published evidence.

To quantify how much architecture matters beyond raw model capability, we benchmarked six LLMs (gpt-5-mini, gpt-5-nano, gpt-oss-120b, gpt-oss-20b, qwen3-next-80b and qwen3-32b) either as plain models or embedded in the HemaGuide pipeline on 45 de-identified, high-complexity cases (15 per entity group) (Fig. 2a and Extended Data Figs. 3 and 4). Blinded expert hematologists scored each output on six 5-point Likert criteria: tumor board concordance (Q1), guideline compliance (Q2), patient-specific contextualization (Q3), reasoning transparency (Q4), clinical utility (Q5) and run-to-run consistency (Q6) (Supplementary Table 4 and Supplementary Note 1).

Embedding LLMs within HemaGuide shifted performance from occasionally plausible to clinically actionable. The highest-capacity open-weight model (gpt-oss-120b) achieved the strongest overall ratings through HemaGuide (mean 4.22 ± 0.063) versus substantially lower and more variable scores as a plain model (3.21 ± 0.39; *P* = 0.0015) (Fig. 2b). This pattern generalized across model families, with smaller open models showing the most marked gains. Variance across cases narrowed substantially: while plain models exhibited wide, case-dependent swings, which we believe forms a critical barrier to dependable clinical use, HemaGuide outputs clustered at the high end of the scale (Fig. 2c). A per-dimension analysis revealed a ‘stable but wrong’ failure mode in plain models, where high consistency coexisted with poor concordance and inadequate patient-context integration, whereas HemaGuide selectively improved the decision-critical dimensions while maintaining consistency (Fig. 2d).

To isolate the contribution of each architectural component, we conducted a systematic ablation study across 11 levels (L0–L10), from a plain LLM baseline through isolated components and progressively enriched combinations to the full agent with autonomous routing. In total, 15 cases (five per routing type: guideline, advanced and molecular) were processed at each level and independently scored by two hematologists (inter-rater reliability: Cohen’s *κ* = 0.849, Pearson’s *r* = 0.919). The plain LLM baseline achieved 0% concordance across all 15 cases (mean score 4.1); the full agent achieved 86.7% concordance (mean score 9.0). No single component was sufficient across all case types; instead, concordance gains were strongly routing-type-dependent (Fig. 2e and Supplementary Table 5). Guideline-amenable, mostly newly diagnosed cases were fully resolved by flowcharts alone (L1 100%), advanced cases required the combination of case memory, literature and contextual enrichment (L8 80%), and molecular cases depended on the molecular interpretation toolkit (L9 100%). These patterns suggest that the autonomous routing mechanism—selecting the appropriate tool combination for each clinical scenario—is the critical integrating element justifying the system’s multimodal architecture.

### Molecular mode reproduces expert variant classification and accelerates precision treatment recommendations

**Fig. 3:**
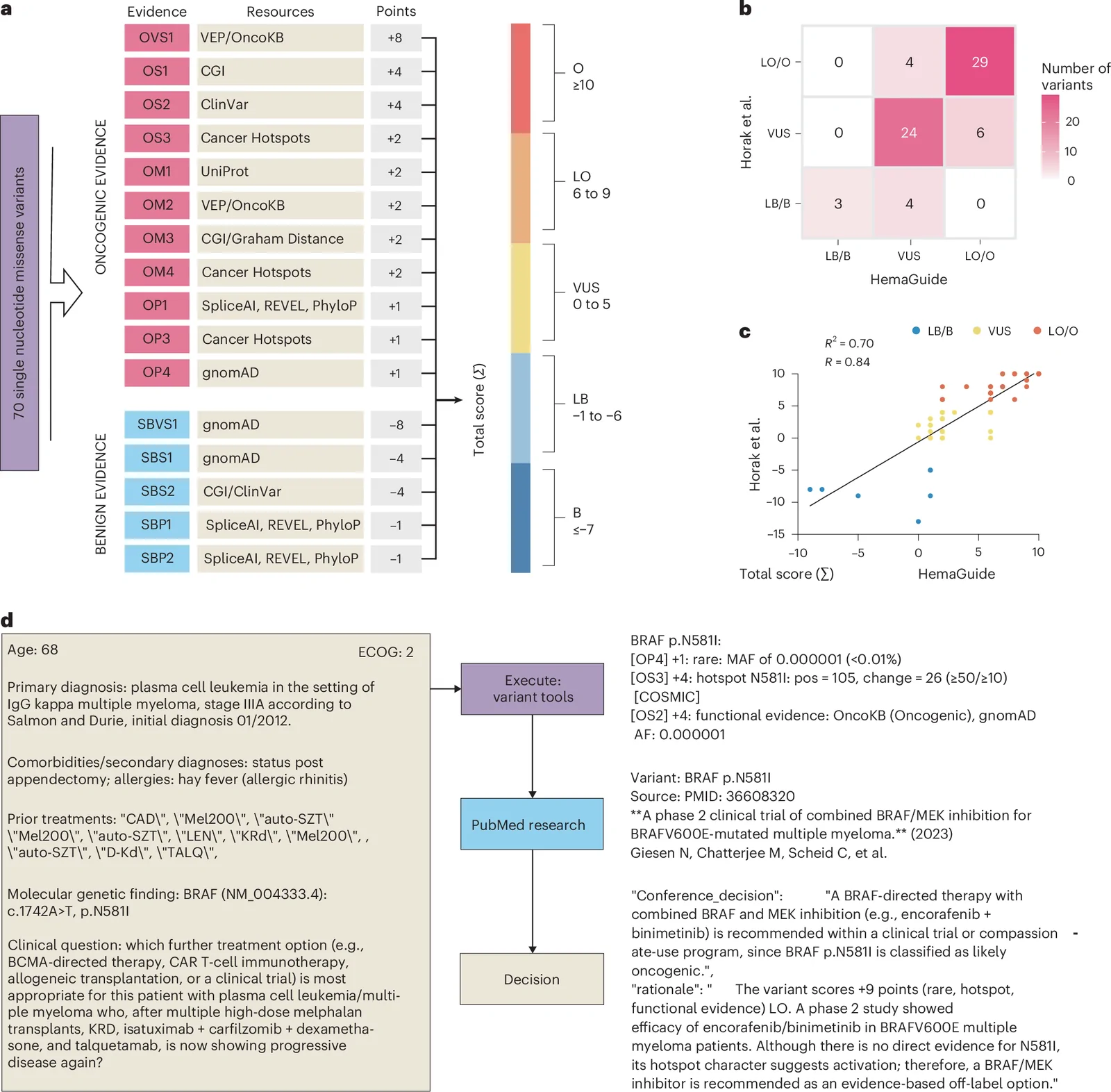
Molecular mode reproduces manual guideline-based classification of single-nucleotide missense variants and generates evidence-linked precision oncology recommendations. **a**, The implementation of a point-based somatic variant oncogenicity framework according to ClinGen/CGC/VICC recommendations: evidence categories, point weights and the knowledge resources queried for each criterion, yielding a total score (*Σ*) and categorical classification (LB/B, VUS and LO/O). **b**, A confusion matrix comparing HemaGuide molecular mode classifications with manual reference classifications across 70 single-nucleotide missense variants (three-bin grouping shown), including predictive value summaries. Performance on structural variants, gene fusions and copy-number alterations was not evaluated in this benchmark. **c**, The quantitative agreement between HemaGuide and manual scoring shown by correlation of total scores, with Pearson’s correlations reported. **d**, An end-to-end molecular mode example: extraction of the relevant clinical context and molecular finding, automated criterion-level scoring with point breakdown, triggered PubMed retrieval and synthesis into a conference-ready recommendation with explicit rationale and cited evidence.

We benchmarked HemaGuide’s automated oncogenicity assessment on 70 single-nucleotide missense variants against reference classifications derived from the ClinGen/CGC/VICC framework^10^. Using three clinically pragmatic bins (likely benign/benign (LB/B), variant of uncertain significance (VUS), likely oncogenic/oncogenic (LO/O)), HemaGuide concurred with the reference for 56/70 variants (Fig. 3b and Supplementary Table 6). Discordant calls concentrated at category boundaries. Overall discrimination was strong (sensitivity 0.88, specificity 0.84, PPV (positive predictive value) 0.83 and NPV (negative predictive value) 0.89), with quantitative scores correlating closely with reference totals (Pearson’s *r* = 0.84, ICC = 0.806) (Fig. 3c). No variant classified as LO/O by expert consensus was classified as LB/B; an asymmetry that matters because false downgrading of oncogenic drivers could deprive patients of mechanism-based options.

An end-to-end evaluation in a representative rare late-line case with sequencing information (PC leukemia, progressive disease after multiple prior lines including talquetamab) demonstrated the full pipeline: HemaGuide extracted clinical context, identified a BRAF p.N581I variant, classified it as LO (+9 points), triggered a focused PubMed search retrieving a phase 2 trial of combined BRAF/MEK inhibition^11^ and synthesized a conference-ready recommendation concordant with the original tumor board decision. The whole workflow completed under real-time conditions on commodity hardware with a median latency of 39 s rather than the hours typically required for manual molecular board workflows (Fig. 3d). Importantly, the molecular mode complements rather than replaces nonmolecular reasoning, only when sequencing information is available: the majority of approved therapies in hematological malignancies are selected on clinical rather than molecular criteria, and ‘guideline’ and ‘advanced’ modes address these dimensions independently.

### HemaGuide improves decision adequacy and augments clinician performance across subspecialties

**Fig. 4:**
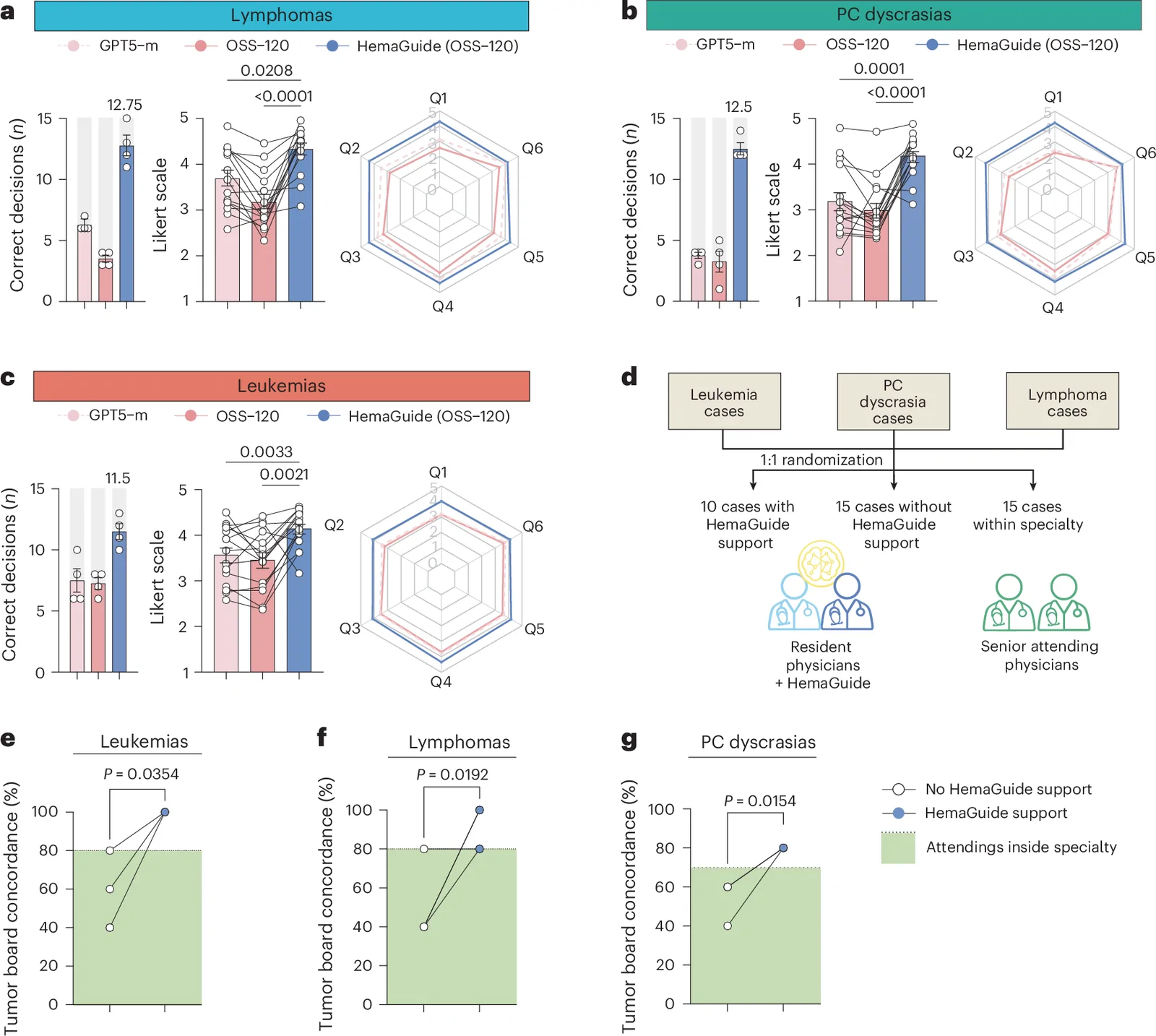
Controlled evaluation of end-to-end HemaGuide workflow and augmentation of clinician performance across subspecialties in a simulated practice setting. **a**–**c**, An entity-stratified controlled evaluation using previously unseen complex cases for lymphomas (**a**), PC dyscrasias (**b**) and leukemias (**c**), comparing a high-capacity open-weight local backbone (plain gpt-oss-120b), a strong closed model baseline (gpt-5-mini) and the final HemaGuide configuration executing full routing and mode selection. For each entity, *n* = 15 independent patient cases were evaluated, with each case independently scored by four blinded resident physicians. Left: per-entity case adequacy (number of correct decisions out of 15) shown as bar charts; each data point represents the number of cases rated as correct (Q1 ≥4 on a 5-point Likert-scale) by one individual rater. Data are mean ± s.e.m. Middle: expert Likert scoring; each data point represents the mean Q1–Q6 score for one patient case, averaged across the four blinded raters (*n* = 15 independent patient cases per model configuration). Statistical significance was determined by a two-tailed paired *t*-test; *P* values are shown. Exact *P* values, listed from top to bottom as indicated by the brackets in each panel (gpt-5-mini versus HemaGuide; gpt-oss-120b versus HemaGuide): *P* = 0.0208 and *P* = 0.0000306 (**a**), *P* = 0.0001 and *P* = 0.0000226 (**b**), and *P* = 0.0033 and *P* = 0.0021 (**c**). Data are mean ± s.e.m. Right: radar plots summarizing performance across Q1–Q6, highlighting that HemaGuide improves concordance, guideline compliance, patient-context integration, reasoning transparency and utility while maintaining high consistency. **d**, The simulated practice study design. Resident physicians (*n* = 4) evaluated 30 cases unaided or with HemaGuide support. Senior attending physicians (*n* = 3) evaluated cases within their subspecialty and a set outside their subspecialty. **e**–**g**, Tumor-board concordance rates across groups for leukemias (**e**), lymphomas (**f**) and PC dyscrasias (**g**), showing improved concordance for residents using HemaGuide and performance comparable to (or exceeding) senior physicians when cases fall outside the senior’s entity subspecialty; individual dots and group summaries are shown. Each data point represents the concordance rate (percentage of correctly recommended cases out of 10 cases per entity) of one individual evaluator. Significance was determined by a two-tailed unpaired *t*-test; *P* values are shown. Data are mean ± s.e.m.

We next tested whether a deployment-oriented HemaGuide configuration could deliver prospectively useful recommendations with a high-capacity open-weight backbone (gpt-oss-120b) executing the full end-to-end workflow. Across three diagnostic groups using complex, previously unseen cases, HemaGuide reached a median of 11.5–12.75/15 correct recommendations per entity group, compared with 3.5–7.5/15 for plain gpt-oss-120b and 3.75–7.5/15 for gpt-5-mini (Fig. 4a–c and Supplementary Table 4). Likert-based scoring confirmed that HemaGuide improved the medical decision-critical dimensions: concordance, guideline compliance and patient-context integration, while maintaining high transparency and utility.

In a simulated practice study, resident physicians evaluated 30 complex cases (10 per entity group) either unaided or with HemaGuide support; senior attending physicians evaluated cases within their subspecialties (Fig. 4d and Supplementary Table 7). Unaided residents matched the tumor board ground truth in approximately 60% of cases, whereas residents with HemaGuide support achieved near-senior concordance, and in some comparisons superior performance, relative to senior physicians assessing cases inside their primary disease expertise (Fig. 4e–g).

### External validation confirms concordance across an independent multi-entity cohort

**Fig. 5:**
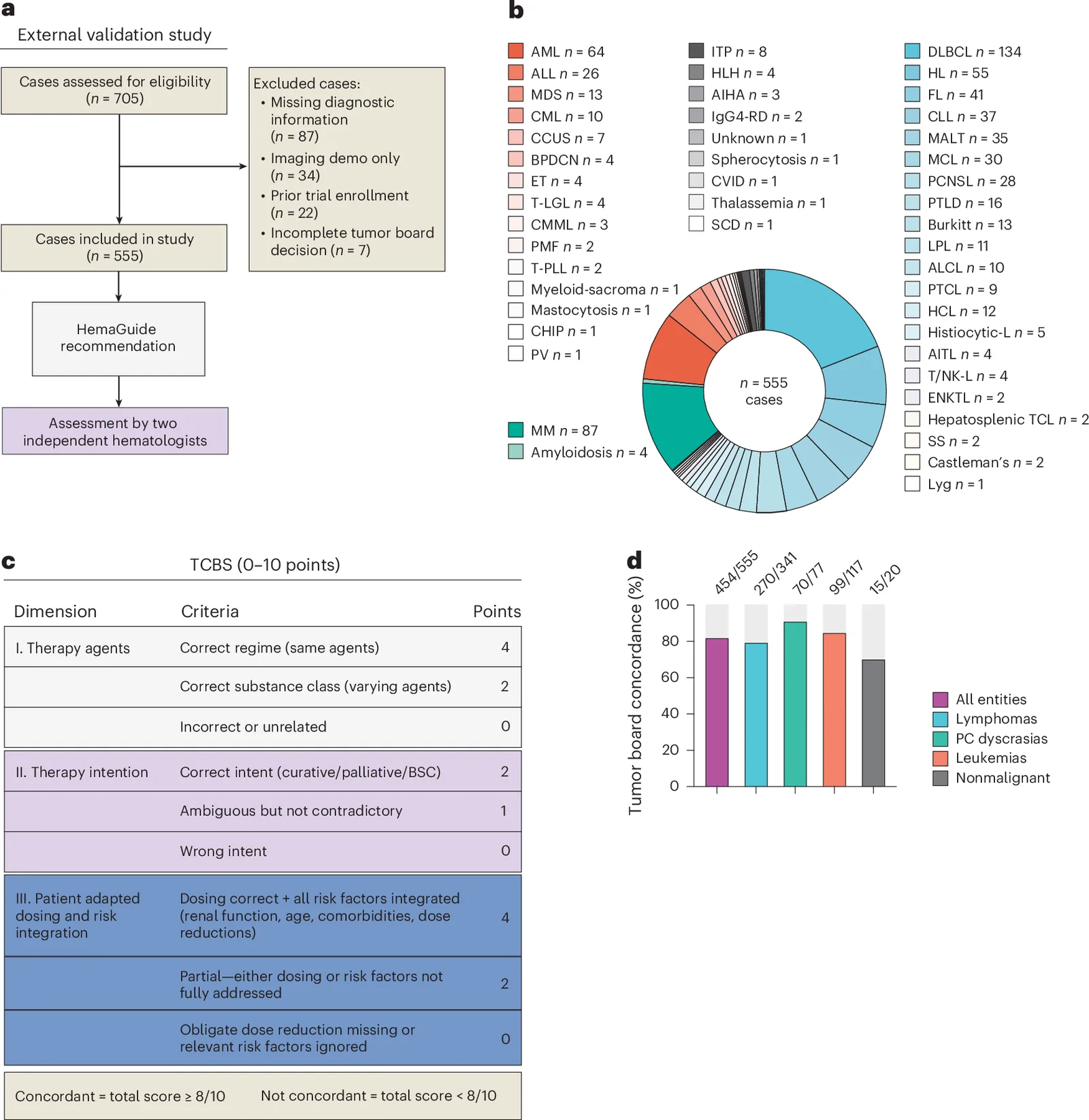
External validation of HemaGuide on an independent multi-entity cohort from a second academic center. **a**, The study design: de-identified tumor board cases from LMU Munich were processed by HemaGuide and scored against ground-truth tumor board decisions using the TBCS. **b**, The entity composition of the 555 evaluable external cases, spanning lymphomas (*n* = 341), leukemias (*n* = 117), PC dyscrasias (*n* = 77) and nonmalignant hematological conditions (*n* = 20), with exclusion criteria shown. **c**, The definition of the TBCS: a 10-point composite metric scoring therapy agents (0–4), therapy intention (0–2) and patient-adapted dosing and risk integration (0–4); concordance defined as a total score ≥8/10. **d**, Concordance rates by entity group, showing an overall concordance of 81.8% (454/555). Data are mean ± s.e.m. AIHA, autoimmune hemolytic anemia; AITL, angioimmunoblastic T-cell lymphoma; ALCL, anaplastic large cell lymphoma; ALL, acute lymphoblastic leukemia; AML, acute myeloid leukemia; BPDCN, blastic plasmacytoid dendritic cell neoplasm; BSC, best supportive care; CCUS, clonal cytopenia of undetermined significance; CHIP, clonal hematopoiesis of indeterminate potential; CLL, chronic lymphocytic leukemia; CML, chronic myeloid leukemia; CMML, chronic myelomonocytic leukemia; CVID, common variable immunodeficiency; DLBCL, diffuse large B-cell lymphoma; ENKTL, extranodal NK/T-cell lymphoma; ET, essential thrombocythemia; FL, follicular lymphoma; HCL, hairy cell leukemia; HL, Hodgkin lymphoma; HLH, hemophagocytic lymphohistiocytosis; IgG4-RD, IgG4-related disease; ITP, immune thrombocytopenia; LPL, lymphoplasmacytic lymphoma; Lyg, lymphomatoid granulomatosis; MALT, mucosa-associated lymphoid tissue lymphoma; MCL, mantle cell lymphoma; MDS, myelodysplastic syndrome; MM, multiple myeloma; PCNSL, primary central nervous system lymphoma; PMF, primary myelofibrosis; PTCL, peripheral T-cell lymphoma; PTLD, post-transplant lymphoproliferative disorder; PV, polycythemia vera; SCD, sickle cell disease; SS, Sézary syndrome; TCL, T-cell lymphoma; T-LGL, T-cell large granular lymphocytic leukemia; T-PLL, T-cell prolymphocytic leukemia; T/NK-L, T/NK-cell lymphoma.

To evaluate generalizability, we conducted an external validation using de-identified tumor board cases from Munich University Hospital on-site. After exclusions (missing diagnostic information, *n* = 87; imaging-only, *n* = 34; trial enrollment, *n* = 22; and incomplete ground truth, *n* = 7), 555 evaluable cases spanning 47 entities remained (Fig. 5a,b and Supplementary Table 8).

Using a 10-point composite metric capturing therapy agents (0–4), therapy intention (0–2) and patient-adapted dosing/risk integration (0–4), with concordance defined as ≥8/10 (Fig. 5c), HemaGuide achieved an overall concordance of 81.8% (454/555), with entity-group rates of 79.2% for lymphomas (270/341), 90.9% for PC dyscrasias (70/77), 84.6% for leukemias (99/117) and 70.0% for nonmalignant conditions (15/20) (Fig. 5d), as rated by two independent hematologists (*κ* = 0.934). This evaluation used the original HemaGuide configuration without local re-optimization, confirming that the decision logic is not overfit to a single institution’s document structure or therapeutic style.

### Prospective silent validation confirms real-time concordance

**Fig. 6:**
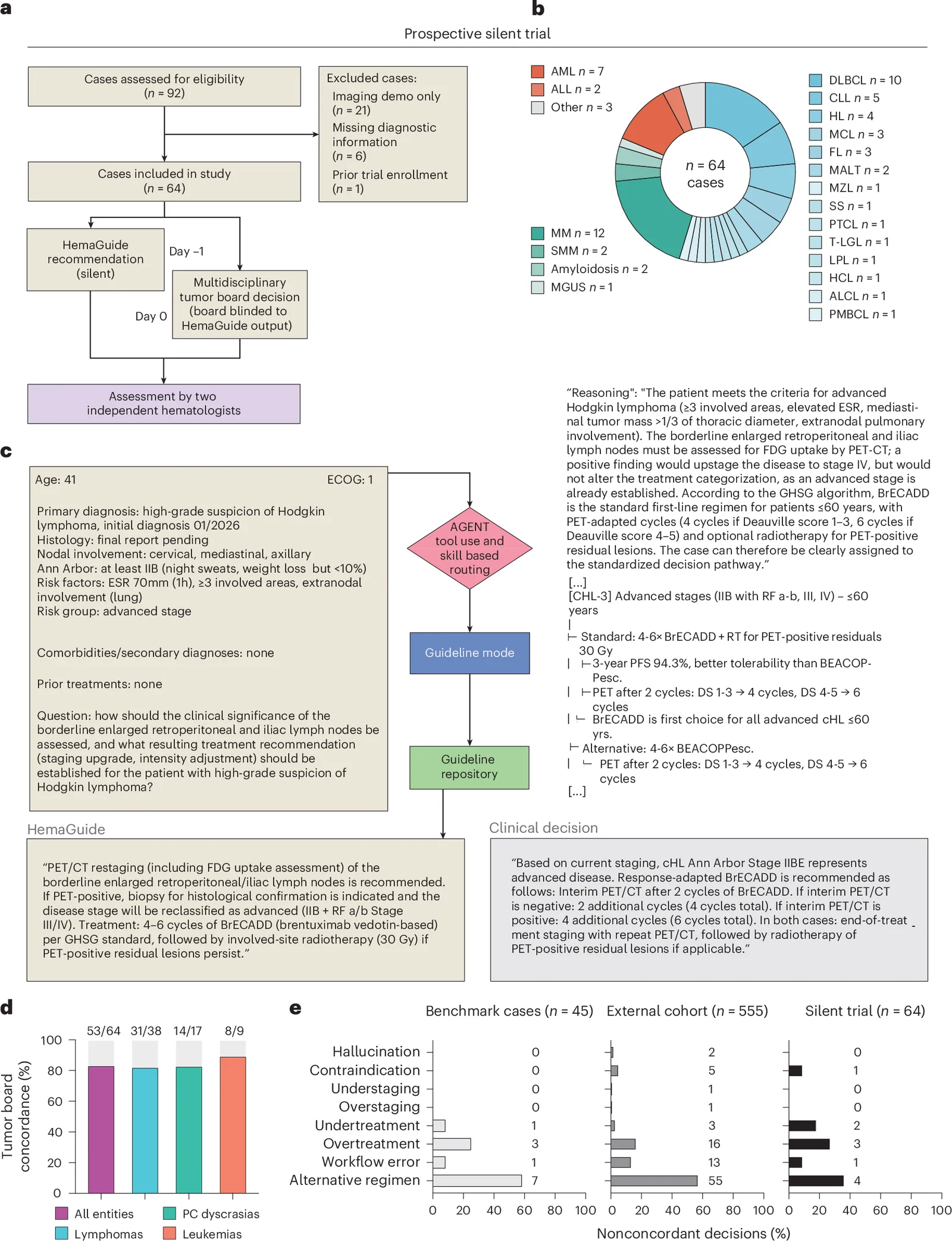
Prospective silent validation of HemaGuide on consecutive, unselected tumor board cases. **a**, The study design: HemaGuide generated recommendations 1 day before the scheduled tumor board conference (day −1); attending physicians made the clinical decision (day 0) blinded to HemaGuide output. **b**, The entity composition of the 64 evaluable cases, with exclusion criteria shown. **c**, A representative case illustrating end-to-end guideline mode execution: for a patient with high-grade suspicion of classical Hodgkin lymphoma (Ann Arbor stage ≥IIB), HemaGuide traversed the institutional cHL (classical Hodgkin lymphoma) decision flowchart and recommended response-adapted BrECADD (brentuximab vedotin, etoposide, cyclophosphamide, doxorubicin, dacarbazine, dexamethasone) with positron emission tomography (PET)-guided cycle determination, concordant with the subsequent tumor board decision. **d**, Concordance rates by entity group, showing an overall concordance of 82.8% (53/64). Data are mean ± s.e.m. **e**, The distribution of nonconcordant decisions by HemaGuide across all study cohorts illustrating the relative frequency of different discordance categories. Numerical values next to the bars indicate the respective absolute counts (*n*).

To approximate real-world deployment, we conducted a 1-month prospective silent trial in which HemaGuide generated recommendations for consecutive tumor board cases without influencing clinical decisions. For each case, the system processed the clinical document 1 day before the scheduled conference (day −1); the corresponding entity-specific tumor board decided the following day (day 0), blinded to HemaGuide output (Fig. 6a and Supplementary Table 9). From 92 consecutive cases, 64 met inclusion criteria across 25 entities (Fig. 6b). HemaGuide achieved 82.8% concordance (53/64), with entity-group rates of 81.6% for lymphomas (31/38), 82.4% for PC dyscrasias (14/17) and 88.9% for leukemias (8/9) (*κ* = 0.795) (Fig. 6c,d).

### Systematic discordance analysis reveals clinically interpretable failure modes

To contextualize nonconcordant cases, we categorized all discordances across evaluation cohorts into eight clinically meaningful types (Fig. 6e and Supplementary Table 11). The dominant category was alternative regimen selection (mean 50.7% ± 7.1% s.e.m. of discordant cases), in which HemaGuide identified a regimen that differed in specifics from the tumor board choice but was guideline-consistent. This pattern was consistent across cohorts (benchmark 58.3%, silent trial 36.4% and external validation 57.3%) and reflects expected inter-institutional variability rather than clinical errors.

Hallucinations—fabricated drugs, clinical facts or treatment histories with no basis in the input data—were observed in 2 of 664 evaluated cases (0.3%), both in the external validation dataset; none occurred in the benchmark or silent trial cohorts. Together with the ablation data showing 0% concordance for ungrounded plain LLMs, these findings suggest that the retrieval-first, tool-constrained architecture substantially mitigates but does not eliminate confabulation risk.

### A locally deployable interface for institutional decision support

**Extended Data Fig. 5:**
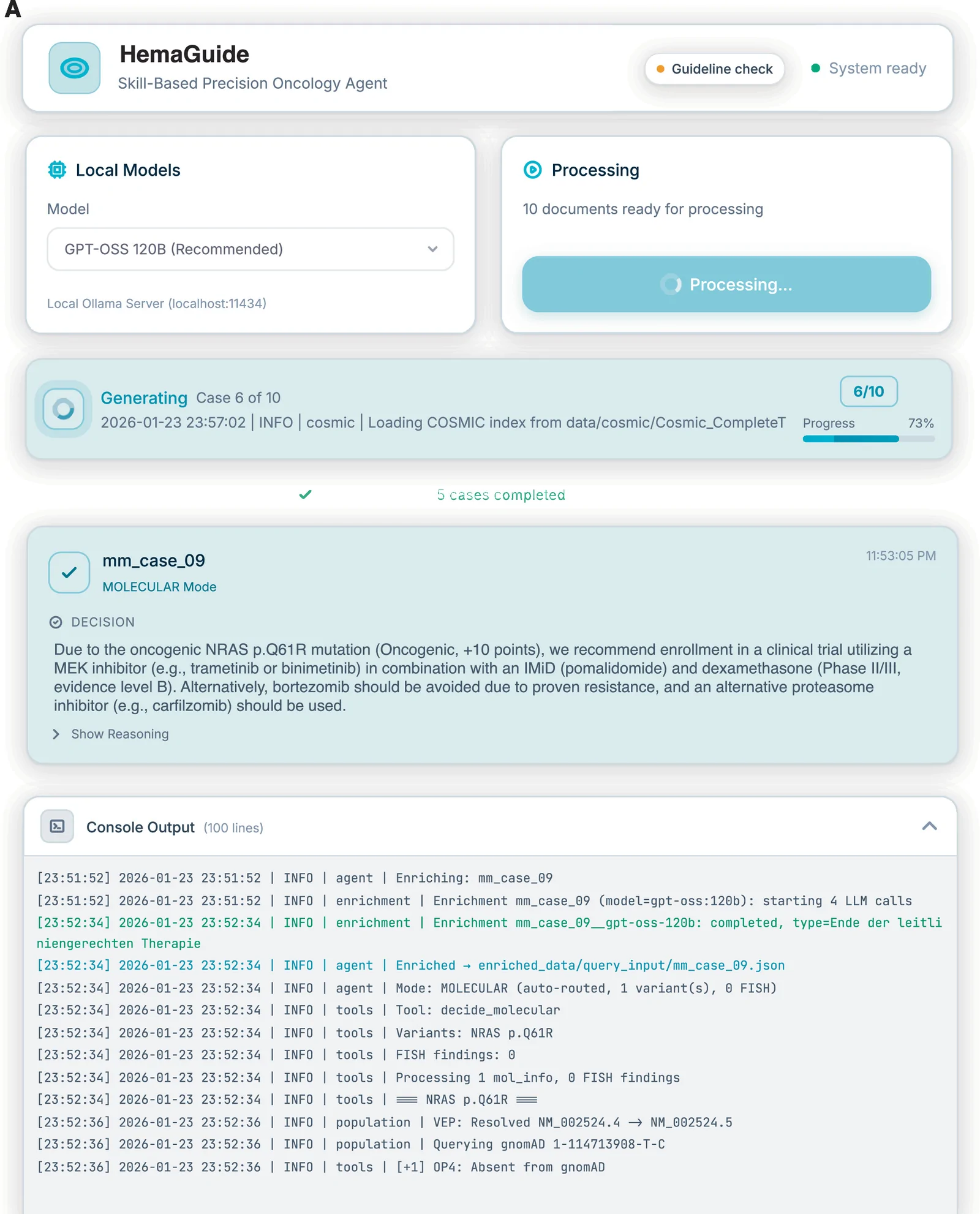
Graphical user interface for locally deployed *HemaGuide*, supporting manual document ingestion and mode-routed decision support. (**a**) Graphical user interface view of *HemaGuide* designed for institutional evaluation and tumor board preparation, including manual batch ingestion of clinical documents (Word-format tumor board summaries), real-time monitoring of extraction and enrichment progress, model selection and local inference configuration (for example via a local server), and presentation of mode-routed recommendations. The interface displays natural-language decisions and expandable reasoning traces for *Guideline*, *Advanced*, and *Molecular Modes*, alongside console-level audit logs that document tool invocation and intermediate steps (for example, variant processing and database queries). Example outputs illustrate an evidence-linked molecular recommendation driven by an oncogenic MAPK-pathway alteration and an advanced-mode recommendation leveraging late-line immunotherapy reasoning, formatted for tumor-board review and documentation. Documents are ingested manually via the interface; the system does not currently integrate with electronic health record or hospital information systems.

**Extended Data Fig. 6:**
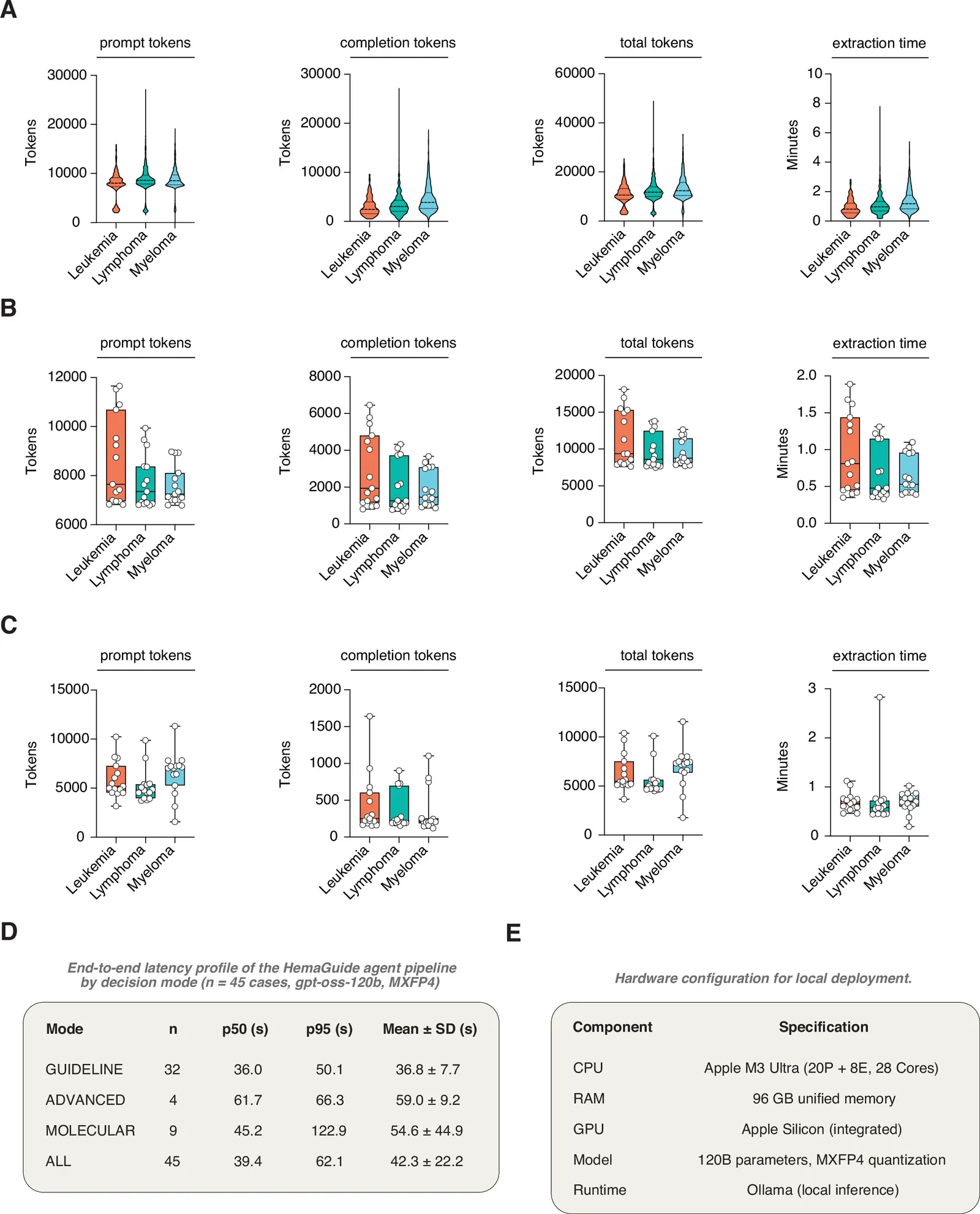
End-to-end latency profile and hardware configuration for local *HemaGuide* deployment. (**a**) Token usage (prompt, completion, total) and processing time for the full CCM extraction, stratified by tumor board group (n = 153 leukemia-, n = 774 lymphoma-, n = 1,114 plasma cell dyscrasia cases). (**b**) Token usage and processing time for the extraction step of the 45 benchmark cases, stratified by disease group (leukemia, lymphoma, myeloma; n = 45 cases). Box plots show the median (centre line) and the 25th to 75th percentiles (box bounds), with whiskers extending from the minimum to the maximum value. (**c**) Token usage and processing time for the decision generation step of the 45 benchmark cases, stratified by disease group (leukemia, lymphoma, myeloma; n = 45 cases). Box plots show the median (centre line) and the 25th to 75th percentiles (box bounds), with whiskers extending from the minimum to the maximum value. (**d**) End-to-end latency profile by decision mode: median (p50), 95th percentile (p95) and mean ± SD in seconds for *Guideline Mode* (n = 32), *Advanced Mode* (n = 4), *Molecular Mode* (n = 9) and all modes combined (n = 45). All measurements obtained using gpt-oss-120b with MXFP4 quantization via Ollama. (**e**) Hardware configuration used for local deployment: Apple M3 Ultra (28-core CPU), 96 GB unified memory, integrated Apple Silicon GPU, with a 120-billion-parameter model under MXFP4 quantization.

**Extended Data Fig. 7:**
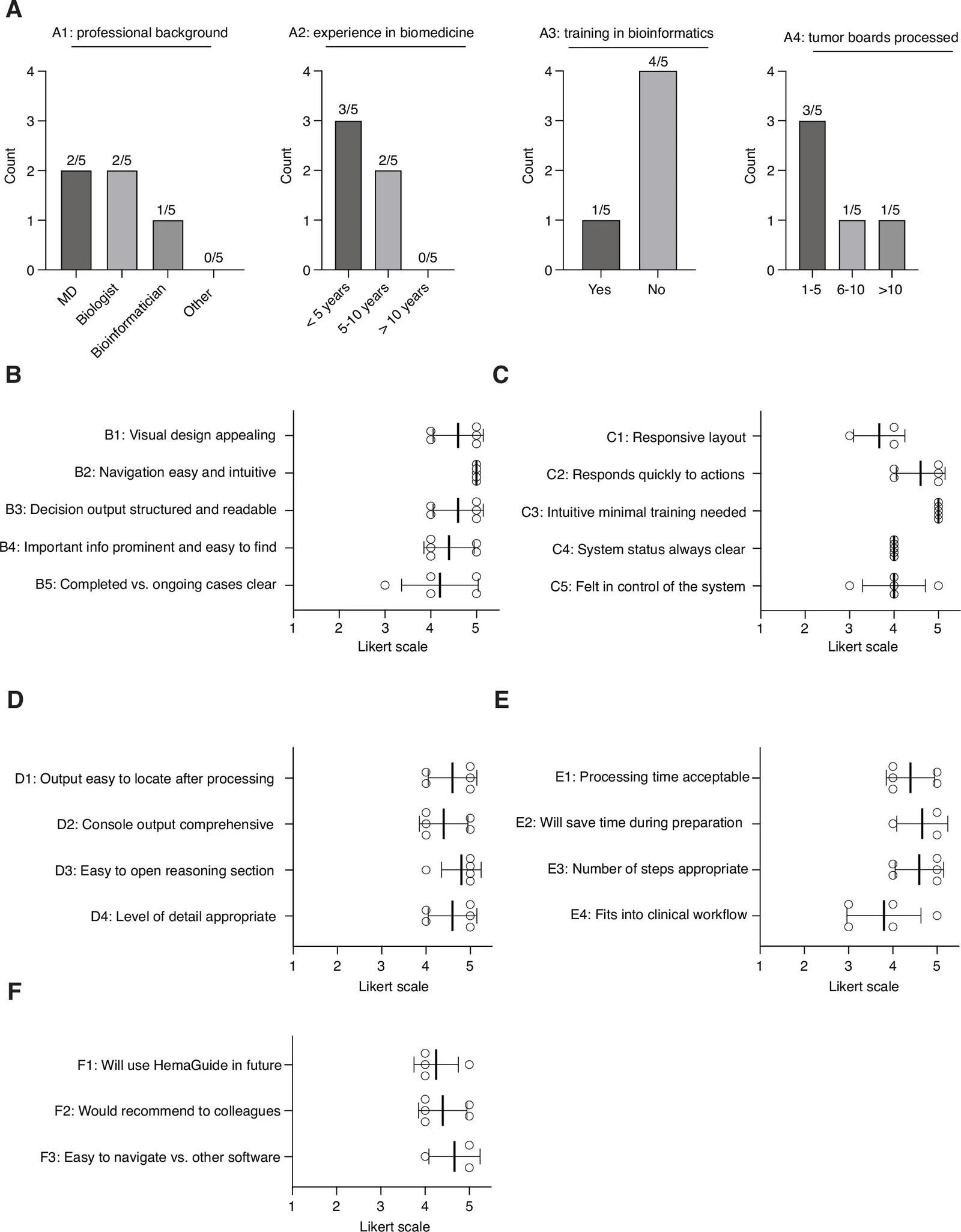
Structured usability evaluation of the HemaGuide interface across professional user groups (n = 5 independent participants). (**a**) Participant demographics: professional background (MD, biologist, bioinformatician, other), years of experience in biomedicine, prior training in bioinformatics and number of tumor boards processed. (**b**) Visual design and layout domain (B1-B5): Likert-scale ratings (1-5) for visual appeal, navigation intuitiveness, decision output readability, information prominence and case status clarity. (**c**) Responsiveness and intuitiveness domain (C1-C5): ratings for responsive layout, action responsiveness, minimal training requirement, system status transparency and perceived user control. (**d**) Data presentation domain (D1-D4): ratings for output locatability, console output comprehensiveness, reasoning section accessibility and level of detail. (**e**) Workflow efficiency domain (E1-E4): ratings for processing time acceptability, anticipated time savings during tumor board preparation, appropriateness of the number of processing steps and fit into clinical workflows. (**f**) Overall user satisfaction domain (F1-F3): ratings for intention to use *HemaGuide* in the future, willingness to recommend to colleagues and ease of navigation compared with other clinical software. Data are presented as mean ± SD; individual participant ratings are overlaid.

We developed a graphical interface operationalizing the full workflow for tumor board preparation, molecular conferences and outside case referrals (Extended Data Fig. 5). Running on commodity hardware (Apple M3 Ultra, 96 GB unified memory) with a 120-billion-parameter backbone under MXFP4 quantization, the full pipeline usually completes in under a minute for most cases (median 39 s, 95th percentile (p95) 62 s), with guideline mode fastest (median 36 s) and molecular mode exhibiting the highest variability (median 45 s, p95 123 s) due to multidatabase variant annotation (Extended Data Fig. 6 and Supplementary Table 10). An automated guideline versioning mechanism compares local flowcharts against source websites and alerts users when updates are available. A structured usability evaluation across diverse professional backgrounds confirmed favorable ratings for workflow integration and willingness to recommend the system (Extended Data Fig. 7).

Collectively, these data demonstrate that a locally deployable, case-grounded HemaGuide configuration can convert a high-capacity open-weight LLM from a variable stand-alone system into a consistently operating decision support tool, with concordance maintained across institutions and under real-time conditions. Whether these gains translate to improved workflow efficiency, decision quality or patient outcomes remains to be established through formal prospective deployment studies.

## Discussion

Late-line hematological malignancies concentrate many of the hardest problems in oncology into a single decision point: patients arrive with long, sequence-dependent treatment histories, narrow therapeutic windows shaped by cumulative toxicity and rapidly evolving standards of care that are often the least prescriptive when the stakes are highest. Here, we show that a retrieval-first, workflow-aligned approach by grounding LLM outputs in curated guideline flowcharts, a contemporary case memory and structured molecular interpretation can convert unstructured clinical documentation into auditable treatment recommendations with concordance that generalizes across institutions and holds under real-time conditions.

The ablation study makes explicit why guideline-only decision support is structurally mismatched to contemporary refractory disease. Guideline flowcharts alone restored full concordance for standard first-line scenarios (L1 100%) but achieved only 20% on advanced cases requiring precedent-based reasoning. For these cases, which represent the clinical scenarios where subspecialty expertise is scarcest, the combination of a curated case memory, literature retrieval and contextual enrichment was required (L8 80%). This parallels recent evidence that LLM performance on contrived knowledge-based exams does not correlate with real-world clinical performance, and that performance against human experts depends strongly on the level of training and realism of the task.

The need for workflow-aligned tool usage becomes particularly acute in molecular oncology, where variant interpretation remains bottlenecked by manual curation^12^. The developed molecular mode approximates the logic of a molecular tumor board workflow rather than treating precision oncology as a generic text-generation task. This approach addresses an emerging care gap: the number of patients with centralized sequencing results is rising faster than the capacity of specialist molecular boards, increasing the risk that actionable mutations are inconsistently recognized^13^.

Our simulated practice evaluation suggests a plausible clinical niche: decision support as a force multiplier when subspecialty expertise is unevenly available. Agent-assisted residents approached near-senior performance, reflecting a pragmatic feature of malignant hematology where expertise is entity-centered while patient journeys increasingly cross boundaries. Whether HemaGuide provides meaningful augmentation at the attending level remains untested. Furthermore, the observation that best-in-class proprietary models benefit less from agentic integration than open-weight models suggests the system’s long-term value may center on institutional customization, data sovereignty and auditability rather than raw reasoning augmentation.

The structured discordance analysis provides important context for interpreting concordance rates. The dominant failure mode was alternative regimen selection (50.7% of nonconcordant cases): clinically defensible recommendations that differed in regimen specifics from the tumor board choice, rather than clinically unsafe errors. Hallucinations were rare (2/664 cases, 0.3%) and confined to the external validation cohort, with none observed in the benchmark or prospective evaluations. Together with the ablation data showing 0% concordance for ungrounded plain LLMs, these findings suggest that the retrieval-first architecture substantially mitigates but does not eliminate confabulation risk.

Several limitations temper the interpretation of these findings. First, the clinical decision memory, guideline flowcharts and internal evaluation cohorts originate from a single academic tertiary-care center with access to the full therapeutic spectrum. External validation (555 cases, Munich University Hospital) and the prospective silent trial (64 consecutive cases) provide evidence of generalizability, but both sites are European tertiary-care centers with comparable therapeutic access; transferability to different countries, drug approval landscapes or resource-constrained settings remains untested. However, the architecture is designed to be adaptable: flowcharts and case memory content can be replaced with institution-specific equivalents.

Second, tumor board consensus decisions serve as the reference standard. While tumor boards represent the highest available standard of clinical deliberation, inter-board variability is well documented and outcome data linking specific recommendations to long-term patient benefit are sparse^3^. Evaluating physicians were blinded to model identity and uninvolved in system development. Future evaluations should prioritize outcome-adjacent endpoints and prospective adjudication by independent external experts.

Third, although our retrieval-first design reduces hallucinations, we have not performed formal calibration of the similarity threshold nor systematic evaluation of low-confidence behavior. The prospective silent trial, while providing initial real-time evidence, remains limited in scale (*n* = 64), duration (1 month) and scope (conducted at the development institution). Workflow integration remains limited to manual document ingestion without EHR (electronic health record) connectivity^14^.

In sum, coupling a curated case memory with workflow-constrained reasoning, structured molecular interpretation and autonomous mode selection improves the consistency, traceability and concordance of LLM-generated treatment recommendations in complex hematological malignancies. Future studies are required for multicenter validation, formal evaluation under uncertainty, EHR integration and demonstration of improved clinical outcomes; however, these results support a practical path toward locally deployable decision support that complements expert tumor board deliberation on commodity hardware, at a cost and latency compatible with routine pre-conference preparation.

## Methods

### Clinical datasets

All research procedures were conducted in accordance with the Declaration of Helsinki. Clinical tumor board documents and physician notes used to construct the HemaGuide clinical case memory were obtained via the Big Data Repository Used for Artificial Intelligence to Better Understand Hematologic Neoplasia (‘BRAIN’) database, a department-wide research repository established at the Department of Hematology, Oncology and Rheumatology of Heidelberg University Hospital. This study framework was approved by the Ethics Committee of the Medical Faculty of Heidelberg University (reference S-837/2019) and the project ‘HemaGuide – a case-grounded AI agent for clinical decision support in hematological malignancies’ was specifically approved by the Ethics Committee of the Medical Faculty of Heidelberg University (reference S-149/2026). Consent-independent data use was performed on the legal basis of Section 6(1) and (2) of the German Health Data Use Act (Gesundheitsdatennutzungsgesetz): the data originate from the healthcare institution’s own clinical records. In accordance with the BRAIN framework, data are handled under applicable confidentiality and data-protection regulations, with patient information processed locally and in pseudonymized form only; no transfer of patient data to third parties occurs and third parties did not receive access to original source documents. For benchmarking against external open- and closed-source models, cases were fully anonymized before use: on the basis of each pseudonymized original case, a new, clinically equivalent case was constructed that preserves the underlying decision situation in its complexity but precludes re-identification of the original patient. As part of this anonymization process, all temporal information relating to diagnosis, treatment course and disease dynamics was systematically altered; cytogenetic and molecular genetic findings were entirely replaced by fictitious yet clinically coherent constellations; rare co-diagnoses and unusual medication combinations that could be identifying in aggregate were substituted with clinically comparable alternatives or generalized; and further potentially identifying details (for example, occupational history, origin and family constellation) were removed. Age values were adjusted only within narrow ranges that preserved the original clinical decision context and never crossed clinically relevant thresholds (for example, transplant eligibility, intensive versus nonintensive therapy stratification or fitness categories per ELN (European LeukemiaNet)/IMWG (International Myeloma Working Group) criteria), such that no influence on routing or treatment recommendations was introduced by the anonymization step. No original genetic data were published or transmitted at any point. All study datasets, intermediate files and logs were stored exclusively on access-controlled infrastructure located on hospital premises and were processed for research purposes only. To ensure data protection, HemaGuide was deployed with a locally hostable inference stack so that case narratives were not transmitted to external model providers.

### System architecture

HemaGuide works through a pipeline architecture with the following steps: (1) pre-runtime memory-construction, (2) structured extraction from input cases, (3) contextual enrichment and routing, (4) agentic decision tool selection and execution and (5) context aggregation with transparent reasoning. The architecture implements three specialized decision tools, which are autonomously selected by the agent: guideline mode, advanced mode and molecular mode. Each mode invokes distinct computational tools and input case handling.

### Knowledge-base architecture for guidelines and SOP repositories

The system maintains a curated repository of entity-specific treatment algorithms, mainly derived from European guidelines and institutional SOPs. These guidelines are encoded as structured decision flowcharts representing canonical treatment pathways organized hierarchically by disease entity, risk stratification category and treatment line.

Flowcharts are stored as plain-text files (.txt) and designed with explicit decision nodes and branching logic for direct insertion into small LLM contexts without any further processing. This ensures that guideline-based reasoning can operate on authoritative, version-controlled source material, which could also be maintained and updated by an LLM at some point in the future.

### Knowledge-base architecture for clinical case memory

A corpus of more than 2,000 institutional tumor board decisions with longitudinal patient follow-up data is indexed in a ChromaDB vector store to enable semantic similarity search. Unlike conventional document-level embedding approaches, we implement section-aware indexing that generates independent embedding vectors for separate clinically meaningful document segments. For each extracted case, the system computes embeddings, for example, for the clinical history narrative, primary diagnosis with staging information and (after the enrichment phase described below) the ‘expanded’ clinical question and a therapy summary. Each embedding is stored with associated metadata including a unique document identifier, section name, entity classification, source file path and fully anonymous case number. This decomposition enables retrieval on the basis of specific clinical dimensions: a query case with an unusual treatment history can match historical cases with similar therapeutic trajectories even when exact diagnoses differ, while a case with a rare cytogenetic profile can match on diagnostic features independently of treatment history. Embeddings are generated using lightweight models suitable for local deployment with the default setting: embeddinggemma:300 m via Ollama with a maximum dimension of 768 or cloud-based alternative for faster and more precise processing via text-embedding-3-large with up to 3,072 dimensions. The vector store uses cosine similarity as a distance metric with a configurable similarity threshold (default 0.7) to remove lower confidence matches. We empirically determined this threshold and added an additional over-retrieval by a factor of 3 of the desired number of similar cases, which were re-ranked and filtered by relevance with another LLM call, where the model was prompted to be an expert in analyzing medical similarity.

### Deduplication control

We implemented a patient-level deduplication filter that checks the unique, fully anonymized case identifier before evaluation runs, excluding all tumor board documents from the same patient (across different time points and case identifiers). This prevents both retrieval of the query case itself and retrieval of temporally adjacent cases from the same patient’s trajectory.

### Preprocessing and information extraction from clinical documents

Clinical tumor board documents or physician’s notes in Microsoft Word format (.docx) undergo a two-phase extraction process to generate semi-structured JSON representations suitable for downstream processing, starting with the classification of the hematological entity. The primary extraction phase employs entity-aware prompts to populate an nine-field clinical schema. We also extract the individual prior therapy lines from the patient history that have been used so far and transform them into a standardized format. This module is applied to both preprocessing for memory-building and for input document extraction during runtime. A parallel extraction module processes molecular appendix content, demarcated by the institutional ‘molecular genetics results’ header, to capture three molecular fields: next-generation sequencing variant data (gene symbol, transcript identifier, coding sequence change in Human Genome Variation Society (HGVS) notation, amino acid change and variant allele frequency), fluorescence in situ hybridization findings with aberration percentages and molecular pathology recommendations. Entity-specific gene panels prime extraction for the most frequent drivers without restricting it. Following extraction, a rule-based post-processing module applies entity-specific risk classification schemas to cytogenetic findings to ensure consistency with established prognostic frameworks including the Revised International Staging System for myeloma^15^ and European LeukemiaNet recommendations for acute leukemias^16^, as documented classification may be missing or outdated. All extraction calls operate at temperature 0.1 to minimize output variability while preserving extraction accuracy.

### Agent orchestration for contextual enrichment of attention-focused retrieval

Before decision generation, extracted cases pass through a three-phase enrichment protocol, with the aim of more structured and focused representations optimized for downstream retrieval and language model attention allocation. This enrichment addresses one key limitation of retrieval-augmented generation (RAG)-based applications with smaller language models: the difficulty of identifying relevant information within lengthy or very complex clinical narratives. The enrichment phases proceed sequentially, each implemented as a separate LLM call at temperature 0.1. First, a question expansion call synthesizes the original clinical question with relevant history and diagnostic context to produce a comprehensive problem statement that articulates the specific clinical dilemma, relevant prognostic factors and constraints on treatment selection including comorbidities and patient preferences. Second, a classification call assigns a tumor board type category (initial diagnosis, relapse, refractory disease, second opinion or routine follow-up) on the basis of the expanded question content. Third, a decision expansion call integrates the documented tumor board recommendation with the full clinical context to produce an enriched decision rationale that explicates the reasoning underlying the treatment selection.

Rather than presenting the language model with extensive unstructured narratives from which relevant precedents must be identified, the enriched representations provide concise, structured question–decision pairs. This attention-focusing mechanism proves particularly valuable when operating with smaller, locally deployed models (≤120B parameters) that exhibit degraded performance when relevant information is distributed across the context window.

### Agent orchestration for agentic routing

Following the extraction and enrichment of query cases, an agent routing module evaluates case characteristics to select the appropriate decision tool from our three options: on the basis of the entity of the case, the corresponding entity-specific data from our library is loaded into the context window and the model can assess the complexity and then select the appropriate tool. Cases explicitly flagged as molecular tumor board consultations, identified through the presence of molecular genetics data, automatically route to molecular mode without requiring a language model routing call. For nonmolecular cases, a routing call presents the case summary alongside descriptions of available decision tools and instructs the model to select the appropriate pathway. The routing decision considers whether the clinical question falls within established guideline coverage (favoring guideline mode), whether unusual features suggest benefit from historical precedent review and literature consultation (favoring advanced mode), or whether molecular findings require systematic variant interpretation (requiring molecular mode).

### Handling of diagnostically uncertain cases

When entity classification during extraction does not yield a recognized hematological entity, for example, because the diagnosis is pending, ambiguous or outside the system’s current entity list, the system assigns a generic fallback classification rather than forcing an incorrect entity assignment. Cases with explicit molecular genetics data are routed to molecular mode regardless of entity classification certainty. Nonmolecular fallback cases are automatically routed to advanced mode, where the similarity search operates without entity filtering: the system queries the clinical decision memory across all disease groups using only the clinical history embedding vector, thereby broadening the evidence base to capture relevant precedents that may span multiple diagnostic categories. For literature retrieval, the term that is recognized and used as the main diagnosis (and not categorized as a hematological entity) is translated from German to English and then used for PubMed and Crossref queries. If no retrieval source meets the configured similarity threshold and literature searches return no relevant results, the system degrades to a plain LLM generation, transparently flagging the absence of grounding evidence and the unclear diagnosis in the output metadata alongside a failure reason (for example, ‘no sources’, ‘LLM synthesis error’ or ‘all sources irrelevant’). This cascading fallback architecture ensures that diagnostically uncertain cases are never forced into entity-specific guideline pathways, while making the degree of available evidentiary support explicit to the reviewing clinician.

### Agent orchestration of guideline mode

Guideline mode provides protocol-based decision support for cases conforming to established early-line treatment algorithms. Upon invocation, the tool retrieves the entity-specific treatment flowchart from the repository and constructs a generation prompt that embeds the complete flowchart directly in the language model context. The generation call receives the full enriched case data alongside the flowchart and explicit instructions to follow the algorithmic decision pathway while documenting which flowchart nodes were traversed to reach the recommendation. This transparency requirement enables verification that the generated recommendation logically follows from the guideline structure rather than representing model confabulation. The output comprises a tumor board recommendation stating the proposed treatment regimen and a rationale documenting the decision pathway with explicit flowchart references. Guideline mode is appropriate for initial diagnosis cases with standard risk profiles, where treatment selection follows well-defined algorithmic pathways and the primary value of decision support lies in ensuring guideline adherence and documentation completeness rather than synthesizing novel treatment approaches.

### Agent orchestration of advanced mode

For cases exceeding guideline-based decision pathways, HemaGuide employs a multisource evidence synthesis workflow that integrates our clinical case memory, peer-reviewed literature by PubMed search and conference proceedings by Crossref search through deterministic retrieval followed by a synthesis step with an LLM call, which selects the most relevant information and compacts it.

### Similar case retrieval

For case retrieval, the clinical case memory ChromaDB vector store is queried using a section matching strategy. For a given query case, the system generates embedding vectors for the clinical sections, then executes similarity searches against the corresponding indexed fields in the knowledge base, with clinical history similarity proving most effective and therefore used exclusively. Results can be aggregated across sections by computing mean similarity scores for each candidate document for identification of cases that exhibit clinical similarity across multiple dimensions rather than single-feature matches. The retrieval applies entity filtering to constrain results to the same hematological malignancy category and enforces a similarity threshold to exclude low-confidence matches in a first step. The default configuration over-retrieves the number of desired similar cases (*n* = 2) up to the factor of three and excludes low confidence matches below the cosine similarity threshold of 0.7, which turned out to be a reasonable threshold for our clinical case memory. Then, LLM-based re-ranking filters again for the three most similar cases regarding the prior treatments.

### PubMed retrieval

Literature retrieval is performed by constructing deterministic PubMed queries from structured case features without language model involvement for maximum reproducibility across executions. Query construction combines entity-specific Medical Subject Heading terms, disease state descriptors derived from the tumor board type classification (for example, ‘relapsed OR refractory’ for relapse cases, ‘newly diagnosed OR first-line’ for initial diagnosis cases) and gene symbols for any actionable variant identified during extraction. The query targets high-evidence publication types: randomized controlled trials, meta-analyses and systematic reviews published within the preceding 5 years, with automatic expansion to 15 years if insufficient results are retrieved.

### Crossref retrieval

Conference literature retrieval supplements PubMed coverage by querying the Crossref API (application programming interface) for proceedings from major hematology/oncology meetings not consistently indexed in MEDLINE. The module targets three primary venues: the American Society of Hematology Annual Meeting (*Blood*), the American Society of Clinical Oncology Annual Meeting (*Journal of Clinical Oncology*) and the European Hematology Association Congress (*HemaSphere*). Queries are filtered by ISSN to ensure precision, with a default 5-year publication window that automatically extends to 10 years if initial retrieval yields insufficient results.

### Context synthesis

Retrieved evidence (similar cases and literature abstracts) is ranked again and then an LLM selects the most promising results on the basis of this ranking. Afterward, the selected information undergoes context tailoring through a synthesis call that extracts clinically relevant insights. The tailoring prompt instructs the model to identify patterns across similar cases (for example, consistent treatment selections for comparable clinical scenarios and outcomes observed with specific regimens) and extract applicable evidence from literature (for example, efficacy data, safety signals and guideline recommendations) without synthesizing these into a premature treatment decision, yet.

### Treatment recommendations in advanced mode

The final generation call receives the complete enriched case, the entity-specific guideline flowchart, the synthesized case precedent summary and the synthesized literature evidence. The model integrates these context sources to generate an annotated treatment recommendation that balances guideline adherence with case-specific considerations informed by clinical case memory precedent and currently available evidence.

### Agent orchestration of molecular mode with federated knowledge curation

The molecular mode implements automated somatic variant classification according to the ClinGen/CGC/VICC SOP published by Horak et al.^10^. This module extends beyond retrieval-augmented generation by orchestrating real-time queries across eight biomedical knowledge bases to systematically evaluate 12 evidence criteria for each detected somatic variant. In addition, it performs a gene matching search in the clinical case memory.

In brief, HGVS notation is translated to genomic coordinates via the Ensembl Variant Effect Predictor REST API^17^ with fallback to NCBI Variation Services when transcript versions are not recognized. Population frequency is queried from the Genome Aggregation Database (gnomAD v4)^18^ to retrieve allele frequencies across population subgroups (SBVS1, SBS1 and OP4). Mutational hotspot recurrence is queried from the cancerhotspots.org^19^ database, with fallback to local Catalogue of Somatic Mutations in Cancer^20^ data, to identify variants at established mutational hotspots (OS3, OM3 and OP3). Null-variant status in tumor suppressors is identified by rule-based parsing (OVS1 and OM2). Functional domain membership is mapped against UniProt^21^. In silico pathogenicity predictions are retrieved from MyVariant.info^22,23^, which aggregates the database of Non-synonymous Functional Predictions (dbNSFP)^24^ annotations. It is then evaluated across REVEL, CADD, SIFT and PolyPhen-2 (OP1 and SBP1). Precedence rules specified by Horak et al.^10^ are applied.

### Classification and treatment recommendations in molecular mode

Points from all applicable criteria are summed to yield oncogenicity classifications: oncogenic (≥10 points), likely oncogenic (6 to 9 points), variant of uncertain significance (0 to 5 points), likely benign (−6 to −1 points) or benign (≤−7 points). For variants classified as oncogenic or likely oncogenic, HemaGuide executes targeted literature searches.

Retrieved literature is assessed through an LLM and rated by relevance (high, medium and low). Only articles rated as high or medium are retained for the final context.

### Agent orchestration for context aggregation and transparent reasoning

Regardless of which decision tool is invoked, all contextual elements are aggregated into a structured prompt for the final generation of a treatment recommendation. The generation call operates at temperature 0.3 to balance output coherence with appropriate response creativity, and produces three outputs: The ‘decision’ stating the treatment recommendation in the format expected by an institutional multidisciplinary tumor board, the ‘reason’ documenting the evidence basis and reasoning pathway and, for molecular mode cases, a human-readable molecular report detailing per-variant classification with criterion-level justification.

This architecture is designed to maintain maximum transparency by documenting all intermediate artifacts. Tool invocations are logged with timestamps and input parameters. Retrieved similar cases are recorded with similarity scores and section-level match details. Together, this audit trail enables post hoc verification of decision pathways and supports the human-in-the-loop review model whereby AI-generated recommendations serve as decision support subject to expert validation rather than autonomous clinical decisions.

### CCSS and TBCS for comparing tumor board cases and conference decisions

To standardize comparing medical text-based information within the speciality of hematology, we had expert hematologists develop two assessment scores: (1) the clinical case similarity score (CCSS) and (2) the tumor board concordance score (TBCS).

The CCSS is used to establish a metric to make clinical tumor board cases comparable and comprises the following four dimensions: (1) entity match, (2) therapy phase, (3) prior therapy overlap and (4) age (Extended Data Fig. 2a). On the basis of the points given in each dimension and ranging from 0 to 10, cases with a score <5 have low similarity, 5–7 signals moderate similarity and >7 is considered highly similar.

The TBCS focuses on comparing the tumor board decision only and spans the following three dimensions: (1) therapy agents, (2) therapy intention and (3) Patient-adapted dosing and risk integration (Fig. 5c). The concordance score covers the range from 0 to 10 and a total of 8 points or more shows concordance.

Every time these scores were applied, we had at least two physicians evaluating them and always calculated the inter-rater reliability.

### Silent trial of a prospective real-world testing scenario

We conducted a silent trial at the University Hospital Heidelberg for the total duration of 1 month, during which we obtained and de-identified all tumor board cases that were listed just before the tumor board conference started and processed them in parallel. Following this process, we arrived at a decision by HemaGuide just before the actual conference decision was available. Cases were excluded if they lacked sufficient diagnostic information, were primarily imaging demonstrations without clinical case discussion, or if the tumor board recommendation was just following enrollment into a clinical trial. The silent trial was run with a total case number of 64 (lymphoma 38, myeloma 17 and leukemia 9) and the docx files were fed into the system as they were originally prepared by the physicians for the real conferences.

We compared the results generated by HemaGuide against the ground truth with our TBCS to guarantee consistent outcomes with the external cohort. The concordance comparison was carried out by two independent hematologists and inter-rater reliability was calculated. In cases of disagreement, a third independent reviewer was consulted to reach a final determination.

### Large-scale validation with data from another academic center

We used an external cohort of 555 independent and de-identified tumor board cases from Munich University Hospital (LMU), covering 47 distinct hematological entities, to validate HemaGuide. All cases were processed by the agent; the system generated the tumor board decisions and this output was assessed with our TBCS against the ground truth from Munich tumor board documents. The concordance comparison was carried out by two independent hematologists with a third reviewer resolving any discordant assessments. Inter-rater reliability was calculated and indicated for all data points.

### Ablation study for the incremental evaluation of architectural components

To quantify the contribution of single system components to decision quality, we conducted a systematic ablation study isolating eleven configuration levels (L0–L10) that progressively add data layers (what the system has access to) and intelligence layers (how the system processes information), ranging from plain LLM baseline as the lower bound (L0; no tools and no additional information) through isolated data layers (L1 guideline flowcharts, L2 clinical case memory, L3 literature retrieval only and L4 molecular tools results only) and progressively enriched combinations (L5–L7: adding enrichments and LLM-intelligence functionality (=intel) to L1–L3, L8: clinical case memory, literature and intel, and L9: molecular and intel) to the full agent with autonomous routing (L10).

We selected a stratified subset of 15 cases of our 45 complex cases to ensure balanced coverage of the complex spectrum across routing paths: 5 cases per mode. Each ablation level was then evaluated on all 15 cases, leading to 165 combinations.

We had all 165 HemaGuide decisions evaluated against the ground truth by two independent hematologists. Evaluators were blinded to the ablation level and configuration. We used a concordance score that was utilized to assess correctness of the ablation results by comparing the following dimensions: (1) therapy agents (0, 2, 4), (2) therapy intent (0, 1, 2) and (3) dosing risk (0, 2, 4). On the basis of these individual ratings a concordance score was calculated, showing concordance between decisions when scoring 8 or above, and based on this concordance score we computed an inter-rater reliability.

### Human evaluation and benchmarking of HemaGuide versus base models

In the first evaluation step, we performed a blinded, physician-rated comparison of HemaGuide against baseline LLMs. Four resident physicians (postgraduate years 1, 2, 4 and 5, spanning the range of hematology training experience) independently evaluated model outputs across 45 complex malignant hematology cases (15 leukemia, 15 lymphoma and 15 PC dyscrasia case journeys). Case selection was stratified by disease timepoint to ensure coverage of the clinical complexity spectrum encountered in hematological tumor boards: newly diagnosed cases (*n* = 4–5 per entity), representing standard first-line decision scenarios; relapsed/refractory cases (*n* = 7–8 per entity), representing the highest-complexity decisions where decision support tools are most clinically relevant; and molecular tumor board cases (*n* = 3 per entity), designed to evaluate molecular mode performance on variant interpretation tasks. Evaluating physicians were not involved in the design or development of HemaGuide and had no prior exposure to its outputs.

For each model configuration, three independent outputs per case were generated (runs 1–3), yielding 135 model runs and corresponding recommendations per configuration for expert assessment. Reviewers were blinded to model identity and configuration throughout; case assignment order was randomized to minimize order effects. Dimensions Q1–Q5 (tumor board concordance, guideline compliance, patient-context integration, reasoning quality and clinical utility) were assessed on run 1 only to avoid rating fatigue and anchoring bias from repeated exposure to the same case; dimension Q6 (run-to-run consistency) was assessed across all three runs. The evaluation dimensions were: (1) tumor board concordance (alignment with the interdisciplinary tumor board recommendation), (2) guideline compliance (consistency with current national and international guidelines, including Onkopedia, DGHO and ESMO), (3) patient-context integration (correct identification and consideration of prior therapies, disease status and therapy-relevant comorbidities), (4) reasoning quality (transparency and comprehensibility of the rationale), (5) clinical utility (usefulness for decision support in practice) and (6) consistency (stability of the recommendation across three repeated runs given identical input).

We used interdisciplinary tumor board consensus decisions as the reference standard, as these represent the highest routinely documented level of clinical deliberation at our institution. We acknowledge that tumor board decisions are not uniquely correct and that inter-board variability has been documented in literature; the use of single-institution tumor board consensus as ground truth is a pragmatic choice that may introduce systematic bias toward the therapeutic preferences and trial portfolio of our center (‘Discussion’).

All participating resident physicians provided written informed consent before study participation. As the evaluation involved no direct patient contact and utilized only de-identified case material, resident participation was classified as a quality assessment activity and conducted in accordance with institutional guidelines.

### Model specifications

The system supports multiple language models to accommodate varying deployment requirements. Cloud-based deployments utilize OpenAI API endpoints (including gpt-5-nano and gpt-5-mini) with JSON mode enforcement for structured extraction outputs. Local and privacy-preserving deployments utilize Ollama-hosted models (including gpt-oss-120b and Qwen3-Next-80B-A3B). A hybrid configuration supports Ollama Cloud endpoints for organizations requiring European data residency without local GPU infrastructure. Embedding generation for the clinical case memory uses embeddinggemma:300 m (768 dimensions) for local deployments or text-embedding-3-large (3,072 dimensions) for cloud deployments. Vector storage uses ChromaDB with cosine similarity as a distance metric and persistent storage to enable incremental knowledge base updates.

### Quantification and statistical analysis

Data are represented as individual values or as mean ± s.d., unless indicated otherwise. Group sizes (*n*) and applied statistical tests are indicated in figure legends. Statistical significance and multiple hypothesis testing corrections were assessed as indicated in figure legends. All reported *P* values are two-tailed, unless indicated otherwise. All analyses were performed using either R v4.5.1 (www.R-project.org) or GraphPad Prism 11.0. For all expert assessment experiments, LLM outputs were blinded to the assessors. Owing to the nature of this study, sample size determination was not applicable, as all available clinical cases from 2024 to 2025 were included in this study.

### Data visualization

Data visualization was performed in GraphPad Prism 11.0 or in the R programming environment (v 4.5.1) using the ggplot2 package for heat maps, confusion matrices and dot plots; the fmsb package for radar charts; and the viridis package for color mapping. Figures were produced using Adobe Illustrator 2026.

### Reporting summary

Further information on research design is available in the Nature Portfolio Reporting Summary linked to this article.

## Online content

Any methods, additional references, Nature Portfolio reporting summaries, source data, extended data, supplementary information, acknowledgements, peer review information; details of author contributions and competing interests; and statements of data and code availability are available at https://doi.org/10.1038/s41591-026-04494-4.

## Supplementary information

Supplementary Information Simulated malignant hematology patient cases used for expert benchmarking. Clinical case summaries including diagnosis, treatment history, expert conference decisions and corresponding LLM-generated therapy recommendations (three runs per case).

Reporting Summary

Peer Review File

**Supplementary Table 1** Composition of the Clinical Case Memory: number of cases per disease entity, **Supplementary Table 2** De-identified case-level metadata across the clinical case memory: newly diagnosed (*n* = 907), relapsed/refractory (*n* = 665), post CAR-T (*n* = 344) and molecular tumor board cases (*n* = 107), **Supplementary Table 3** Similarity scores for the five highest-ranked cases retrieved by the clinical case memory for a representative myeloma and leukemia query case, decomposed into entity match (0–3), therapy phase (0–2), therapy overlap (0–3) and age proximity (0–2), with the composite similarity score per retrieved case, **Supplementary Table 4** Individual Likert-scale ratings (Q1–Q6) for 45 complex hematological malignancy cases evaluated by four physicians across six LLM backbones in plain and HemaGuide-embedded (agent) mode. Q1 assessed tumor board concordance/clinical accuracy, Q2 guideline compliance/concordance, Q3 patient-specific contextualization and context integration, Q4 reasoning transparency and treatment sequencing, Q5 clinical utility and risk–benefit assessment, and Q6 run-to-run consistency and actionability, **Supplementary Table 5** Case-level evaluation data for the systematic ablation study across eleven configuration levels (L0–L10), including subdimension scores and overall TBCS, **Supplementary Table 6** Molecular mode benchmark variant set: representative somatic variants with gold-standard ClinGen/CGC/VICC classifications (per Horak et al.) used for comparison to automated interpretation, **Supplementary Table 7** Case-level data for the simulated practice study, reporting treatment recommendations from four resident physicians assessed unaided and with HemaGuide support alongside three senior subspecialist evaluations, with ground truth, TBCS subdimension scores and binary concordance judgments for each of the 30 cases, **Supplementary Table 8** Case-level concordance evaluation of HemaGuide on an independent multi-entity external validation cohort from LMU Munich, reporting ground truth, HemaGuide recommendation, TBCS with subdimension scores and binary correctness adjudication, **Supplementary Table 9** Case-level evaluation of consecutive tumor board cases from a 1-month prospective silent validation, reporting ground truth, HemaGuide recommendation, TBCS with subdimension scores and binary correctness assessment, **Supplementary Table 10** Token usage and processing time for HemaGuide across three operational stages: knowledge base extraction (2,041 clinical case memory cases), structured case extraction (45 benchmark cases) and agent-based decision generation (45 benchmark cases), stratified by disease group (lymphomas, PC dyscrasias and leukemias) and reporting duration (seconds), prompt tokens, completion tokens and total tokens per case, **Supplementary Table 11** Classification of all discordant cases across the benchmark, silent trial and external validation cohorts by entity and primary discordance reason (alternative regimen selection, overtreatment, undertreatment, workflow error, hallucination, contraindication nonrecognition or staging error), **Supplementary Table 12** Lookup table mapping the abbreviated entity identifiers used throughout the study to their full clinical nomenclature.

## Data Availability

All constructed clinical cases used for benchmarking, along with the corresponding agent outputs, are available in the Supplementary Information.

## Code availability

An implementation of our source codes for researchers to extend on our work is available via GitHub at https://github.com/Friedrich-Lab/HemaGuide. Instructions for using the repository are detailed in the respective README.md file.
