# Supplementary material for: Clinical decision support in hematological malignancies using a case-grounded AI agent
Source: Nat Med. 2026 Jun 30;32(9):3224–34. doi: 10.1038/s41591-026-04494-4 (PMC13577921; doi:10.1038/s41591-026-04494-4)
Supplement: Supplementary file 1 [file 41591_2026_4494_MOESM1_ESM.pdf]

nature medicine

Supplementary information https://doi.org/10.1038/s41591-026-04494-4

# Clinical decision support in hematological malignancies using a case-grounded AI agent

In the format provided by the authors and unedited

**Supplementary Table 1 | Composition of the Clinical Case Memory (CCM): number of cases per disease entity.**

**Supplementary Table 2 | De-identified case-level metadata across the CCM: newly diagnosed (n=907), relapsed/refractory (n=665), post CAR-T (n=344), and molecular tumorboard cases (n=107).**

**Supplementary Table 3 | Similarity scores for the five highest-ranked cases retrieved by the Clinical Case Memory for a representative myeloma and leukemia query case, decomposed into entity match (0-3), therapy phase (0-2), therapy overlap (0-3), and age proximity (0-2), with the composite similarity score per retrieved case.**

**Supplementary Table 4 | Individual Likert-scale ratings (Q1–Q6) for 45 complex hematological malignancy cases evaluated by four physicians across six LLM backbones in plain and HemaGuide-embedded (agent) mode. Q1 assessed tumor board concordance/clinical accuracy, Q2 guideline compliance/concordance, Q3 patient-specific contextualization and context integration, Q4 reasoning transparency and treatment sequencing, Q5 clinical utility and risk–benefit assessment, and Q6 run-to-run consistency and actionability.**

**Supplementary Table 5 | Case-level evaluation data for the systematic ablation study across eleven configuration levels (L0-L10), including subdimension scores and overall TBCS.**

**Supplementary Table 6 | Molecular Mode benchmark variant set: representative somatic variants with gold-standard ClinGen/CGC/VICC classifications (per Horak *et al.*) used for comparison to automated interpretation.**

**Supplementary Table 7 | Case-level data for the simulated practice study, reporting treatment recommendations from four resident physicians assessed unaided and with HemaGuide support alongside three senior subspecialist evaluations, with ground truth, TBCS subdimension scores, and binary concordance judgments for each of the 30 cases.**

**Supplementary Table 8 | Case-level concordance evaluation of HemaGuide on an independent multi-entity external validation cohort from LMU Munich, reporting ground truth, HemaGuide recommendation, TBCS with subdimension scores, and binary correctness adjudication.**

**Supplementary Table 9 | Case-level evaluation of consecutive tumor board cases from a one-month prospective silent validation, reporting ground truth, HemaGuide recommendation, TBCS with subdimension scores and binary correctness assessment.**

**Supplementary Table 10 | Token usage and processing time for HemaGuide across three operational stages: knowledge base extraction (2,041 CCM cases), structured case extraction (45 benchmark cases), and agent-based decision generation (45 benchmark**

**cases), stratified by disease group (lymphomas, plasma cell dyscrasias, leukemias) and reporting duration (seconds), prompt tokens, completion tokens, and total tokens per case.**

**Supplementary Table 11 | Classification of all discordant cases across the benchmark, silent trial, and external validation cohorts by entity and primary discordance reason (alternative regimen selection, overtreatment, undertreatment, workflow error, hallucination, contraindication non-recognition, or staging error).**

**Supplementary Table 12 | Lookup table mapping the abbreviated entity identifiers used throughout the study to their full clinical nomenclature.**

# l_case_1

Age: 51

ECOG: 1

Primary diagnosis:
Acute myeloid leukemia, ID 01/2026
Classification according to WHO 2022: AML with CEBPA-Mutation
Classification according to ICC: AML with in-frame bZIP CEBPA-Mutation
At initial diagnosis:
Peripheral blood: Hb 9,4 g/dl, Plt 42/nl, WBC 62/nl, 88% blasts
Bone marrow aspiration and biopsy from 01.01.2026:
Cytology: Detection of 72% myeloid blasts
FACS: CD45 low' SSC low, CD34+' CD117+' HLA-DR+' CD38+, CD33+' CD13+, CD7-, CD56+/-, CD64+
LAIP in the blast gate: 91 %
LAIP of CD45+ cells: 82 %
Karyotype (BM/PB): 46, XX [20] normal female karyotype
FISH (BM): Exclusion MECOM-, KMT2A- and CBFB-Rearrangement; no Detection of t(8;21), t(9;22), t(15;17); no deletions 5q, 7q, 17p
Molecular genetics (BM): NPM1 neg., CEBPA pos. (biallelic: in-frame bZIP-Mutation + N-terminal Frameshift-Mutation), FLT3-ITD neg., FLT3-TKD neg., IDH1/2 neg., BCR::ABL1 neg., DDX41 neg., TP53 WT, MLL-PTD neg.
Risk score (after ELN): Favorable risk

Secondary diagnoses:
None

Treatment and course:
Cytoreductive therapy with Hydroxyurea from 03.01.-12.01.26

Question for the tumor board:
Treatment initiation?

Tumor board decision:
Treatment initiation with Daunorubicin/Cytarabin (7+3 regimen). With Detection of a In-Frame-Mutation in the bZIP-domain of CEBPA, additional administration of GO. In case of response no indication for consolidating allogeneic stem cell transplantation.

# l_case_2

Age: 48

ECOG: 2

Primary diagnosis:
Acute promyelocytic leukemia, AML M3 ID 01/26
Sanz risk classification: High risk
Initial blood count: L 5,00/nl, Hb 5,0 g/dl, Plt 5/nl
FISH: Detection of a translocation t(15;17)
Molecular genetics: PML::RARA pos.
FACS: CD45 low' SSC intermediate to high, CD34-' CD117+/- (low)' HLA-DR-' CD38+ (low), CD33+' CD13+, CD64+ (99% in the blast gate, 85% the CD45+ cells)
Bone marrow aspiration:
Cytology: 70% promyelocytes, compatible with AML M3
FACS: CD45 low, SSC intermediate-high, CD34-, CD117+, HLA-DR-, CD38+, CD33+, CD13+, CD64+ (95% in the blast gate, 95% the CD45+ cells)
Molecular genetics: PML::RARA pos.
Karyotype: 46, XX, t(15;17)(q24;q21) [7]
FISH: Detection of a translocation t(15;17)

Secondary diagnoses:
Allergies: none

Treatment and course:
ED AML M3 01.01.2026
Therapy ATO/ATRA and Idarubicin on 01.01.26 started.
Currently: Leukocytes 65.000/µl:

Question for the tumor board:
Treatment initiation

Tumor board decision:
Continuation of therapy with ATO/ATRA with high-risk APL under addition of HU 2g 2x daily. No leukapheresis.

# l_case_3

Age: 58

ECOG: 0

Primary diagnosis:
Acute myeloid leukemia, ID 01/2026
ICC 2022: AML with MDS-associated gene mutations (SF3B1)
WHO 2022: AML, therapy-related (t-AML)
L 1,6/nl, Hb 8,2 g/dl, Plt 68/nl, blasts 2%, LDH 195 U/l
BM histology: hypercellular, signs of dysplasia
BM cytology: 35% myeloblasts, trilineage dysplasia
FACS: 15% blasts, LAIP: CD45 low' SSC low, CD34+' CD117+' HLA-DR+' CD38+/-, CD33+' CD13-, CD56+/-
Molecular genetics: NPM1 WT, FLT3-ITD/TKD neg., IDH1 neg., IDH2 neg., TP53 WT
FISH: unremarkable
NGS: SF3B1 Mutation positive (38,7%)
Karyotype: 46,XY,del(7)(q31q36)[6]/46,XY,del(20)(q11.2)[2]/46,XY[12]
Risk profile: adverse

Secondary diagnoses:
Breast carcinoma, right, ID 05/2019
Stage: pT1c, pN0 (0/3 sn), L0, G2, R0, cM0, History of adjuvant chemotherapy and radiation

Treatment and course:
ED AML 05.01.2026

Question for the tumor board:
Treatment initiation?

Tumor board decision:
Induction with Vyxeos, prospectively allogeneic stem cell transplantation

# l_case_4

Age: 60

ECOG: 2

Primary diagnosis:
Acute lymphoblastic leukemia, common B-ALL type, ID 03/2024
At initial diagnosis:
Peripheral blood: Hb 8,2 g/dl, Plt 12/nl, Leukocytes 2,8/nl, 15% blasts
Bone marrow:
Cytology: 72% blasts
Histology: 90% blasts of a B-ALL. CD79a+, CD10+,
CD34+, TdT+, CD20(+) Ki-67 85%. Bcl6- CD23- CyclinD1- CD5- MPOFACS: CD45 low, SSC low, CD34+, CD19+, CD10++
FACS: ED-LAIP (55 %): CD45 low, SSC low, CD34+, CD19+, CD10+, CD22+
Molecular genetics: BCR-ABL1 not detectable
Histology (reference pathology): 65 % infiltration B-lymphoblastic leukemia; MF-1

Secondary diagnoses:
Allergies: none

Treatment and course:

Currently:
Relapse of the common B-ALL
Bone marrow aspiration from 03.07.2025:
Cytology: 5% blasts
FACS: 22% blasts, Immunophenotype: CD34+, CD19+, CD10+, CD20-, CD22+, CD38+, TdT+, CD33-, CD13-
Lumbar puncture from 08.07.2025:
Cytology: 1500 cells/µl, Detection of blasts
FACS: 90% blasts
Initiation of local therapy with intrathecal triple therapy 3x/week from the 10.07.2025
Initiation of systemic therapy with of a Dexamethason pre-phase therapy analogous the GMALL recommendations
Newly developed motor impairments

Question for the tumor board:
Treatment recommendation?

Tumor board decision:
Relapse therapy with Inotuzumab ozogamicin; parallel intrathecal triple therapy for treatment of the ZNS-Manifestation, after of a Cycle Inotuzumab systemic CNS-active therapy (HD-MTX i.v. depending on general condition and blood count with or without HD-Cytarabin); subsequently Continuation of therapy with Inotuzumab. CD19-CAR / Allo-SCT to be considered.

================================================================================
TREATMENT TIMELINE
================================================================================
22.01.24 Start Pre-phase with Dexamethason and Cyclophosphamid (each 200 mg/qm) after GMALL
28.01.24 Induction I based on the GMALL-protocol 07/2003 for BCR-ABL1 negative ALL without
Rituximab and PEG-Asparaginase, liposomal Doxorubicin
Received: MTX 15 mg i. t. (Day 6), days 6+7, days 13+14
07.03.24 Remission assessment (Bone marrow aspiration)
Cytology: 3 % blasts
FACS: blasts with unremarkable immunophenotype, no definite evidence of MRD positivity
FACS/Molecular genetics/Classical cytogenetics and FISH:
MRD neg.
11.03.24 1. Cycle Blinatomumab

25.04.24 2. Cycle Blinatomumab
02.06.24 Remission assessment (Bone marrow aspiration)
Cytology: 2% blasts
FACS: no definite evidence of MRD positivity
20.06.24 3. Cycle Blinatomumab
08.08.24 Remission assessment (Bone marrow aspiration)
Cytology: 3% blasts
FACS: no definite evidence of MRD positivity
09.09.24 &
22.09.24 Consolidation II (Part 1 and 2) analogous GMALL in dose reduction
MTX 1000mg/m2 (d1, d15), PEG-Asp 500 U/m2 (d2, d16), 6-MP 25mg/m2 d1-7 and d15-21
26.10.2024 Remission assessment (Bone marrow aspiration)
Cytology: 3% blasts
FACS: blasts with unremarkable phenotype, no definite evidence of MRD positivity
MRD: negative
03.11.2024 -
07.11.2024 Consolidation III analogous GMALL
Cytarabin 1000mg/m2 (d1, d3, d5), Cyclophosphamid 200mg/m2 (d1,d3,d5
24.11.2024 Consolidation IV analogous GMALL
MTX 1,5g/m2 iv. PEG-Asp.500U/m2, 6MP 25mg/m2.
MTX 15mg absolute, Cytarabin 40mg absolute, Dexa 4mg absolute
08.12.2024 Continuation Consolidation IV d15
30.12.24 Consolidation V analogous GMALL
Cytarabin 1000mg/m2 (d1, d3, d5), Cyclophosphamid 200mg/m2 (d1,d3,d5)
23.01.25 Consolidation VI analogous GMALL
MTX 1,5g/m2 iv. PEG-Asp.500U/m2, 6MP 25mg/m2.
MTX 15mg absolute, Cytarabin 40mg absolute, Dexa 4mg absolute (intrathecal)
08.02.25 GMALL Consolidation VI d15
MTX 1,5g/m2 iv. PEG-Asp.500U/m2, 6MP 25mg/m2 (08.02. - 14.02.2025)
02.03.25 Start of the Maintenance therapy
Remission assessment (Bone marrow aspiration)
Cytology: 2% blasts
FACS: MRD negative
MRD: negative
01.03.25 Remission assessment
Cytology (BM): 4% blasts
FACS: blasts with unremarkable immunophenotype, no definite evidence of MRD positivity
FACS/Molecular genetics/Classical cytogenetics and FISH:
MRD neg.
================================================================================

# l_case_5

Age: 72

ECOG: 1

Primary diagnosis:
Common B-ALL, ID 11/2021
At initial diagnosis:
Peripheral blood: Hb 7,2 g/dl, Plt 28/nl, WBC 3,5/nl, 12% blasts
Bone marrow:
Cytology: 88% blasts
Flow cytometry:
CD45 low, SSC low, CD34+, CD19+, CD20+/-, CD22+, CD10+, TdT+, CD33-, CD38+
FISH: unremarkable findings, no ALL-typical translocations
Histology: Interstitial and nodular infiltration, MF-0/1
Molecular genetics: BCR::ABL1 negative

Secondary diagnoses:
Breast carcinoma, right, ID 03/2023
- Stage: pT1c, pN0 (0/2 sn), L0, G2, R0, cM0
- Hormone receptor status: ER 80%, PR 60%, Ki67: 15%, HER2: negative

Treatment and course:

Question for the tumor board:
Treatment indication?

Tumor board decision:
With rising MRD positivity initially Treatment initiation with Blinatumomab. CAR-T / Allo-SCT to be considered.

================================================================================
TREATMENT TIMELINE
================================================================================
12.04.2020 Start Pre-phase (Dexamethason + Cyclophosphamid); Induction I based on GMALL-elderly.
15.04.2020 CT H/T/A: presacral soft tissue formation without bone destruction (a.e. extramedullary hematopoiesis; DD neurogenic tumor/Chordom less likely). Prominent glandular tissue left breast (if applicable, gynecological correlation). Small cystic lesion pancreatic head ~0,5 cm (a.e. side branch dilatation).
18.04.2020 Lumbar puncture: 1 cell/µl; Flow cytometry without blast population.
26.04.2020 Lumbar puncture: 1 cell/µl; Flow cytometry without blast population.
10.05.2020 BM: Cytology ~1% blasts; FACS: no evidence of pathologic blast population (ED-LAIP not detectable).
12.05.2020 CT H/T/A: stable findings ggü. 15.04.2020; presacral formation continued a.e. extramedullary hematopoiesis.
14.05.2020 Induction II (Day 21-32 after GMALL-elderly).
16.05.2020 Lumbar puncture: 1 cell/µl; Flow cytometry without blast population.
24.05.2020 Lumbar puncture: 1 cell/µl; Flow cytometry without blast population.
30.05.2020 Lumbar puncture: 1 cell/µl; Flow cytometry without blast population.
14.06.2020 Remission assessment (Day 62): BM <5% blasts; Flow cytometry/Kiel: MRD negative.
21.06.2020 Consolidation I Part 1: MTX 500 mg/m2 + PEG-Asparaginase 500 U/m2.
07.07.2020 Consolidation I Part 2: MTX 500 mg/m2; without PEG-Asparaginase (transaminase elevation + transfusion-requiring coagulopathy).
18.07.2020 Remission assessment: complete remission; MRD Kiel negative.
30.07.2020 Consolidation II: HD-AraC 500 mg/m2.
31.08.2020 Re-induction therapy (GMALL-elderly; Start).
06.10.2020 Remission assessment before Consolidation III: BM <5% blasts; FACS without pathologic blast population; MRD negative. CT H/T/A: presacral stable, slight size increase para-aortic.
08.10.2020 Consolidation III Part 1 (week 24).

03.11.2020 Consolidation III Part 2 (d16): MTX 500 mg/m2 + PEG-Asparaginase 500 U/m2.
01.12.2020 Consolidation IV: Cytarabin 500 mg/m2 on Day 2/4/6.
21.12.2020 Remission assessment before Consolidation V: no blast increase; FACS/Kiel: MRD negative.
28.12.2020 Consolidation V Part 1: intrathecal triple therapy + MTX 500 mg/m2 + PEG-Asparaginase 500 U/m2. LP: 1 cell/µl; FACS without blasts.
11.01.2021 Consolidation V Part 2: MTX 500 mg/m2 + PEG-Asparaginase 500 U/m2.
02.02.2021 Start Maintenance: MTX 30 mg weekly (Mi) + Folsan 5 mg (Do) + 6-MP (100/50 mg alternating).
24.02.2021 Stop Maintenance: thrombocytopenia Grade IV, leukopenia Grade II, anemia Grade II.
12.03.2021 Imaging/Orthopedics: older BWK2-, BWK11- and BWK12-fractures; right SI joint dysfunction.
18.05.2021 Zoledronsäure 4 mg i.v. with Osteoporosis.
10.06.2021 Hematologic Relapse.
17.06.2021 Relapse confirmed: BM ~51% blasts, CD22 positive.
25.07.2021 Start Inotuzumab-Ozogamicin (Cycle 2; administration on d1/d8/d15).
22.08.2021 Start Inotuzumab-Ozogamicin (Cycle 3; administration on d1/d8/d15).
12.2021 Again MRD-positive.
05.01.2022 Enrollment in CAR-T-study (HD-CAR-1). With high blast burden Bridging: GMALL-elderly Pre-phase, subsequently Blinatumomab.
10.01.2022 Leukapheresis for CAR-T cell production.
11.01.2022 BM: ~52% blasts; MRD positive.
17.01.2022 Start Pre-phase after GMALL-elderly.
20.01.2022 Lumbar puncture (Pre-phase): FACS without Detection of a blast population.
24.01.2022 Blinatumomab Cycle 1, Day 1.
28.04.2022 Clinical screening/pre-infusion: Clearance for CAR-T-study.
06.05.2022 CAR-T cell infusion.
03.06.2022 Day +28: CR, MRD negative (PB/BM).
06.08.2022 Day +92: CR, MRD negative (PB/BM).
13.10.2022 Day +160: CR, MRD negative (PB).
05.11.2022 Day +183: CR, MRD negative (PB).
10.12.2022 Day +218: CR, MRD negative (PB).
12.01.2023 Day +251: CR, MRD negative (PB).
09.02.2023 Day +279: CR, MRD negative (PB).
18.03.2023
04.2023 Invasive breast carcinoma NST, left (ID 04/2023). Pre-treatment: cT2 cN0 cM0; ER ~80%, PR ~70%, Ki-67 ~30%, HER2 positive, G2. Postoperative: ypT1c ypN0 (1sn) L0 V0 Pn0 G2 R0.
05.2023 CT Thorax/Abdomen + scintigraphy: no metastasis.
11.06.2023 Neoadjuvant Trastuzumab/Pertuzumab started.
15.06.2023
24.06.2023 Neoadjuvant Tamoxifen.
16.08.2023 Surgery: BET + SLNB left.
29.08.2023 Tumorboard: T-DM1 post-neoadjuvant generally recommended with not pCR, however waived (CAR-T history). Recommendation: Completion Trastuzumab q3W + aromatase inhibitor + Radiatio from 20.09.2023.
19.09.2023 Kiel MRD positive (PB): 5E-5 (0,005%).
12.10.2023 Kiel MRD positive: PB 1E-5 (0,001%); BM 4E-5 (0,004%).
05.12.2023 Kiel MRD positive (PB): 8E-5 (0,008%).
08.01.2024
20.02.2024 D+655: continued hematologic CR; MRD at the detection limit with continued PCR-detectable CAR-T cells. No treatment indication. Additionally: FACS MRD pos (PB/BM each ~0,01%); Kiel MRD pos (PB/BM each 1E-4).
19.03.2024 D+683: continued hematologic CR; MRD at the detection limit, CAR-T in PCR detectable. No treatment indication. Kiel MRD pos (PB): 4E-4 (0,001%).
================================================================================

# l_case_6

Age: 53

ECOG: 1

Hematologic primary diagnosis:
AML after lineage switch from pre-existing B-ALL (ID 03/2022), with KMT2A-Rearrangement
Cytology: 28% monocytic blasts
FACS: 26% blasts in the Gate: CD45 intermediate, SSC intermediate, CD34-, CD117-, HLA-DR+ (high), CD38+, CD33+, CD13+/-, CD64+, CD14-
Molecular genetics: NPM1 neg., FLT3-ITD/TKD neg., KRAS G12D pos., CEBPA neg.

Previous diagnosis: Pro-B-ALL, Initial diagnosis 12/2022
Cytology: 58% blasts.
Histology: Diffuse blast infiltration (85%), CD34+, PAX5+, TdT+.
FISH: KMT2A Rearrangement in 42% the analyzed interphase nuclei.
FACS: blast gate: CD34+, CD79a+, CD19+, CD22+, TdT+, MPO-, CD3-, CD7-, CD20-
BCR::ABL1 negative
Secondary diagnoses:
Arterial hypertension
Type 2 diabetes mellitus

Treatment and course:

Question for the tumor board:
Further Therapy?

Tumor board decision:
Enrollment in Menin-Inhibitor-study.

================================================================================
TREATMENT TIMELINE
================================================================================
13.09.2021 Pre-phase (GMALL elderly): Dexamethason + Cyclophosphamid
20.09.2021 Induction I (GMALL EWALL BOLD)
12.10.2021 Remission assessment: 4% blasts; FACS (BM) MRD positive
19.10.2021 Blinatumomab Cycle 1 (28 µg/d)
17.11.2021 Remission assessment: 3% blasts; FACS (BM) MRD negative
02.12.2021 Consolidation I (2 parts)
06.01.2022 Remission assessment: 3% blasts; FACS (BM) MRD positive
11.01.2022 Blinatumomab Cycle 2 (28 µg/d)
16.02.2022 Consolidation II: HD-AraC (Cytarabin 1000 mg/m2)
28.04.2022 Re-induction; assessment: BM <5% blasts, FACS negative, MRD (Kiel) negative; CSF negative
26.06.2022 Start Maintenance therapy
10.11.2022 assessment: BM <5% blasts; FACS positive; MRD (Kiel) negative
12.01.2023 assessment: BM <5% blasts; FACS positive; MRD (Kiel) negative
08.03.2023 Relapse with lineage switch to AML (mono.): 24-40% blasts; FACS ~21% (CD34-/CD117-; CD33+/CD64+); Mol.: CEBPA+, NRAS+ (NPM1-/FLT3-)
15.03.2023 Venetoclax/Azacitidin Cycle 1
29.03.2023 BMA d15: 24% blasts; FACS MRD positive (~50% the CD45+ cells, LAIP)
13.04.2023 BMA d28: 17% blasts; FACS MRD positive (~15% the CD45+ cells, LAIP)
14.04.2023 Venetoclax/Azacitidin Cycle 2
04.05.2023 BMA: cytologically CR; FACS MRD negative
10.05.2023 Venetoclax/Azacitidin Cycle 3
08.06.2023 AML-Relapse: FACS ~30% monocytic blasts; FISH: KMT2A-Rearrangement ~7%
================================================================================

# l_case_7

Age: 70

ECOG: 2

Primary diagnosis:
Acute myeloid leukemia with myelodysplasia-related changes (MRC), ID 03.2023
Peripheral blood: anemia, thrombocytopenia, Leukocytes 11200 /µl, approx. 78 % blasts
BMA: AML (M2 after FAB)
Cytogenetics: Complex karyotype: 45, XY, del(5)(q13q33), -7, add(12)(p13), der(17)t(5;17)(q13;p13) [8]/46, XY [2]
Molecular genetics: FLT3-ITD neg., FLT3-TKD neg., NPM1 WT, IDH1 WT, IDH2 WT, CEBPA WT, TP53 mutated (VAF 45%), RUNX1 mutated, ASXL1 mutated
ELN classification: adverse risk profile

Secondary diagnoses:
NYHA II°
History of PTCA with coronary artery disease

Treatment and course:

Question for the tumor board:
Relapse therapy?

Tumor board decision:
Salvage therapy with FLAG-IDA VEN (as per cardiology consult if applicable, VEN+HAM) and upon achieving a new remission 2. allogeneic stem cell transplantation.

================================================================================
TREATMENT TIMELINE
================================================================================
17.10.2021 Induction I: Cytarabin + Daunorubicin (7+3)
14.11.2021 BKP: refractory, complete BM-infiltration
19.11.2021 Salvage: Vidaza + Venetoclax (Cycle 1)
03.12.2021 BMA: ~10% blasts (cytol.; early assessment)
14.12.2021 BMA: ~10% blasts (cytol.), FACS 6%
22.12.2021 Vidaza + Venetoclax (Cycle 2)
07.01.2022 BMA: CR (cytol.), FISH negative, MRD positive (0,5%)
19.01.2022 Vidaza + Venetoclax (Cycle 3; Venetoclax Day 1-21)
05.03.2022 Allogeneic PBSCT (TBI 8 Gy/Flu/ATG)
27.03.2022 Day+21: skin-aGvHD Grade I, resolving with local therapy
03.04.2022 Day+28 BMA: CR; FISH/MRD negative; Chim BM: 2% recipient
09.04.2022 Maintenance: Vidaza + Venetoclax (Cycle 1)
09.05.2022 Maintenance: Vidaza + Venetoclax (Cycle 2); GI symptoms: GvHD excluded; H. pylori + Candida
11.06.2022 Maintenance: Vidaza + Venetoclax (Cycle 3)
14.06.2022 Day+100 BMA: 3% blasts; FISH/MRD negative; Chim BM/PB 100% donor; Start immunosuppression tapering
08.07.2022 Maintenance: Vidaza + Venetoclax (Cycle 4; Venetoclax 200 mg/Day)
22.07.2022 Immunosuppression completed
24.07.2022 Maintenance: Vidaza + Venetoclax (Cycle 5; Venetoclax 200 mg/Day)
14.08.2022 cGvHD joints mild-to-moderate; Hydrocortison; oral candidiasis
27.08.2022 Maintenance: Vidaza Day 1-5 + Venetoclax 100 mg/Day (Cycle 6)
31.08.2022 Day+180 BMA: 3% blasts; MRD negative
07.03.2023 (+12 M) BMA: 3% blasts; MRD/FISH negative; Chim 100% donor; CD4 115/µl; mild cGvHD (joints/eyes)
04.09.2023 (+18 M) BMA: 3% blasts; MRD/FISH negative; Chim 100% donor; mild-to-moderate cGvHD (joints/eyes/oral)
17.12.2023 Chimerism PB: 100% donor; continued mild cGvHD
05.03.2024 (+24 M) BMA: CR; MRD/FISH negative; Chim 100% donor; mild cGvHD; Maintenance (Venetoclax) completed

09.07.2024 Relapse: BMA ~75% blasts; Chim BM 15-25% recipient, PB 5-15%; FISH: del(5q), Aberr. chr7/17p, +11q; Molek./NGS pending
================================================================================

# l_case_8

Age: 64

ECOG: 1

Primary diagnosis:
Acute lymphoblastic leukemia of the B-cell lineage (common B-ALL)
blood count before start of treatment: L 0,41/nl, Hb 9,1g/dl, T 33/nl, LDH 198U/l
BM cytology: 79% blasts
FACS: CD45-, SSC low, CD34 low, CD19+, CD20+, CD22+, Tdt+, CD10+, CD38+; 89% the CD45+ cells
Molecular genetics: BCR-ABL negative, IDH1 positive
Cytogenetics: 46,XY[1] normal male karyotype (findings with limited interpretive value)
FISH: biallelic Deletion 9p21 (nullisomy), gain of chromosomal regions 9q22 and 9q34 as well as in the subclone on tumor cells with gain of chromosomal region 5q32q33

Secondary diagnoses:
History of appendectomy
Type 2 diabetes mellitus
Arterial hypertension

Treatment and course:

Molecular genetic findings

Question for the tumor board:
Pathogenic variant? Targeted Therapy?

Tumor board decision:
Targeted Therapy with Ivosidenib (+Azacitidin).

================================================================================
TREATMENT TIMELINE
================================================================================
21.06.2023 BM diagnostics: Cytology 71% blasts. FACS: B-Vorläufer-ALL (CD19+, CD10+, TdT+, CD22+, CD20+; CD34 low; CD45-/low, SSC low), ~89% the CD45+ cells. Cytogenetics: 46,XY (unauff.). FISH:ED common B-ALL
26.06.2023 Pre-phase (analogous GMALL >55 J): Dexamethason d1-5; Cyclophosphamid d3-5; IT MTX 12 mg
30.06.2023 CSF (i.R. Pre-phase): IT MTX 12 mg. Cytology 2 cells; FACS 1,3% BlastenIT Triple to blast clearance
01.07.2023 Cycle 1 Inotuzumab-Ozogamicin (analogous INITAL-1; d1 0,8 mg/m2, d8+d15 0,5 mg/m2)
06.07.-14.07.2023 3x Lumbar puncture + IT Triple (to blast clearance)
19.07.2023 BMA Remission assessment: Zytol. 2% blasts; MRD (FACS) neg.; MRD (Kiel) neg.
26.07.2023 Cycle 2 Inotuzumab-Ozogamicin
16.08.2023 BMA: Zytol. 3% blasts; MRD (FACS) neg.; MRD (Kiel) neg.
22.08.2023 Cycle 3 Inotuzumab-Ozogamicin
12.09.2023 BMA: Zytol. 3% blasts; MRD (FACS) neg.; MRD (Kiel) neg.
27.09.2023 IT Triple (Prophylaxe/Maintenance)
03.10.2023 Course: Platelets 65/nl; Maintenance with MTX + 6-MP yet not started (cytopenia)
08.11.2023 Persistent thrombocytopenia ~60/nl; MRD (Kiel) negative
28.11.2023 BM: cytol. ~38% precursor cells; FACS: precursor cells of various maturation stages; MRD (Kiel) negative
09.01.2024 BM: cytol. ~22% precursor cells; FACS: precursor cells of various maturation stages; MRD (Kiel) negative
14.05.2024 Progressive cytopenia: Plt 28/nl, Hb 9,6 g/dl, WBC 1,28/nl, Neutro 0,5/nl. BM: signs of dysplasia granulopoiesis/megakaryopoiesis (~10%), 8% blasts. FACS: 2,5% lymphoid blasts (LAIP wie ED) + 0,7% myeloid blasts unremarkable. IDH1 AF ~42%. MRD

(Kiel) pending
================================================================================

================================================================================
TREATMENT TIMELINE
================================================================================
Gene Transcript CDS position Amino acid position Allele frequency (%) COSMIC ID cBio-Portal Pathogenicity
IDH1 NM_005896.4 c.394C>T p.R132C 5 COSM28747 yes
================================================================================

# l_case_9

Age: 63

ECOG: 1

Primary diagnosis:
Acute myeloid leukemia, ID 09/2024
Classification according to WHO 2022: AML post cytotoxic therapy (AML-pCT)
Classification according to ICC 2022: AML with recurrent genetic aberrations (inv(16)(p13.1q22)/CBFB::MYH11)
Initial risk score (after ELN 2022): favorable
At initial diagnosis:
o Peripheral blood: Hb 10,3 g/dl, Plt 89/nl, WBC 5,8/nl
o Bone marrow cytology: 52% blasts
o FACS (BM): LAIP with ED: CD34+/-, CD117+, HLA-DR+, MPO+, CD33+, CD13+; negative for CD19, CD3, CD7, TdT
o Cytogenetics: 46,XY,inv(16)(p13.1q22)[17]/46,XY[3]
o FISH: Detection CBFB-Rearrangement; Exclusion KMT2A-Rearrangement, t(8;21), t(9;22), t(15;17); no deletions 5q, 7q, 17p

o Molecular genetics (BM): CBFB::MYH11 Typ A transcripts positive; FLT3 neg., NPM1 neg., IDH1/2 neg., KIT Exon 17 D816V pos., CEBPA WT, DDX41 neg.

Secondary diagnoses:
Arterial hypertension
Type 2 diabetes mellitus

Treatment and course:

Molecular genetic findings

Question for the tumor board:
Pathogenic variant? Targeted Therapy?

Tumor board decision:
Pathogenic variant. Enrollment in KIT-Inhibitor study.

================================================================================
TREATMENT TIMELINE
================================================================================
03.02.2022 BMA: 60% blasts.
08.02.2022 Cycle 1 Azacitidin/Venetoclax.
13.02.2022 blood count: WBC 2,6/nl (blasts 0%), Hb 7,8 g/dl, Platelets 27/nl.
23.02.2022 BMA: 3% blasts (cyCR); FACS: MRD positive (0,12% blasts, LAIP). Mol. Gene.: CBF::MYH11 155,4.
15.03.2022 Cycle 2 Azacitidin/Venetoclax.
05.04.2022 Remission assessment (BMA): cyCR, 1% blasts; FACS: MRD negative; Mol. Gene.: CBF::MYH11 2,4%.
12.04.2022 Cycle 3: Azacitidin 75 mg/m2 Day 1-7 + Venetoclax 100 mg Day 1-17 (shortened with infection).
08./09.05.2022 G-CSF with febrile infection in aplasia.
16.05.2022 Remission assessment (BMA, external): cyCR; FACS: MRD negative.
18.05.2022 Cycle 4: Azacitidin 75 mg/m2 Day 1-5 + Venetoclax 100 mg Day 1-14.
13.06.2022 Remission assessment (PB, molecular): CBF::MYH11/ABL1 0,105%.
20.06.2022 Cycle 5: Azacitidin 75 mg/m2 Day 1-5 + Venetoclax 100 mg Day 1-14.
12.07.2022 Remission assessment (BM, molecular): CBF::MYH11/ABL1 0,112%.
26.07.2022 Cycle 6: Azacitidin 75 mg/m2 Day 1-5 + Venetoclax 400 mg Day 1-14.
14.09.2022 Remission assessment: complete remission; CBF::MYH11/ABL1 0,082% (PB).
20.09.2022 Cycle 7: Azacitidin 75 mg/m2 Day 1-5 + Venetoclax 400 mg Day 1-14.
02.11.2022 Remission assessment: hematologic CR; CBF::MYH11/ABL1 0,098% (PB) and 0,098% (BM).
03.11.2022 Cycle 8: Venetoclax 400 mg (28 days) + Azacitidin 75 mg/m2 Day 1-7.

30.11.2022 PB (molecular): CBF::MYH11/ABL1 0,030%.
07.12.2022 PB (molecular): CBF::MYH11/ABL1 0,021%.
13.12.2022 Cycle 9: Azacitidin 75 mg/m2 Day 1-5 + Venetoclax 400 mg Day 1-21.
17.01.2023 Remission assessment (PB, molecular): CBF::MYH11/ABL1 0,079%.
27.01.2023 Cycle 10: Azacitidin 75 mg/m2 Day 1-5 + Venetoclax 400 mg Day 1-21.
07.03.2023 Molecular relapse: CBF::MYH11/ABL1 5,2% (PB).
21.03.2023 HiDAC 1 g/m2 bid Day 1-3.
05.05.2023 Hematologic Relapse.
================================================================================

================================================================================
TREATMENT TIMELINE
================================================================================
Gene Transcript CDS position Amino acid position Allele frequency (%) COSMIC ID cBio-Portal Pathogenicity
KIT NM_000222.2 c.2447A>T p.D816V 15-30 COSM1314 yes
================================================================================

# l_case_10

Age: 59

ECOG: 1

Primary diagnosis:
Acute lymphoblastic leukemia, BCR::ABL1-positive (common-B-ALL), ID 07/2022
At initial diagnosis:
PB: Hb 9,8 g/dl, Plt 95/nl, WBC 42,5/nl, 40% blasts
Cytology (BM): blast percentage 65%
Histology (BM): Blast infiltration of approx. 80%, marrow fibrosis WHO-Grade 1
Cytogenetics (BM): 46,XY,t(9;22)(q34;q11.2)[5]/46,idem,-7[12]/46,XY[3], Detection the Philadelphia-translocation, subclone with monosomy 7
Flow cytometry (BM): 72% blasts; LAIP with ED: CD45 low, SSC low, CD34+, CD19+, CD20-, CD22+, TdT+, CD10+, CD38+, CD33-/+
Molecular genetics (BM): BCR::ABL1 positive (p190, e1a2); FLT3, NPM1, IDH1/2 WT
MRD Marker (PB): Molecular MRD markers established: IGH VH-JH clonal V4-34 J4, IGK Vk-Kde clonal
Cytology (CSF): Cell count 2/nl, no detection of atypical cells

Secondary diagnoses:
COPD
Arterial hypertension
Type 2 diabetes mellitus

Treatment and course:

Molecular genetic findings

Question for the tumor board:
Pathogenic variant? Targeted Therapy?

Tumor board decision:
Pathogenic variant. Study option with KRAS-G12D- / MEK-Inhibitor (Trametinib) recommended.

================================================================================
TREATMENT TIMELINE
================================================================================
02/2021 Initial diagnosis: common-B-ALL (BCR::ABL-positive).
11.03.2021 D1 Pre-phase (GMALL): Dexamethason + Cyclophosphamid + intrathecal MTX.
16.03.2021 Induction I (GMALL) + Dasatinib
02.04.2021 Intrathecal MTX; CSF-FACS: no blasts.
03.04.2021 D20 (readmission 02.04): Continuation Induction I + Dasatinib; D20 Pegasparaginase, D21 Vincristin.
05.04.2021 D22 Remission assessment: BM 2% blasts; FACS 0,4% (MRD+); Kiel MRD+; BCR::ABL approx. 9%.
11.04.2021 D28 Vincristin; Dasatinib paused with acute pancreatitis.
19.04.2021 D34 intrathecal MTX 15 mg.
20.04.2021 D35 Vincristin 1 mg absolute.
22.04.2021 Treatment continuation with Imatinib 600 mg/d.
14.05.2021 Remission assessment: complete remission; BCR::ABL MRD ~2,0%.
18.05.-22.05.2021 D61-65 Consolidation I: Vindesin d61, HD-MTX d61, Etoposid d64-65, Cytarabin 2x2 g/m2 d65.
04.06.2021 D76 intrathecal Triple (AraC/MTX/Dexa).
10.06.2021 BMA: cytol. CR, FACS MRD-; BCR::ABL ~0,07% (MVZ); Kiel: 1 Marker pos. (2E-05), BCR::ABL ~0,1%.
18.06.2021 Blinatumomab Cycle 1 + Imatinib 600 mg/d.
24.06.2021 Intrathecal Triple (fluoroscopy-guided).
16.07.2021 BMA: cyCR (1% blasts), FACS MRD-, FISH no evidence t(9;22); Kiel MRD- (<1E-05).
29.07.2021 Blinatumomab Cycle 2 + Imatinib 600 mg/d.

31.08.2021 BMA: cytol. CR, FACS MRD-; BCR::ABL ~0,009%.
09.09.2021 Intrathecal Triple (fluoroscopy-guided): no blasts.
10.09.2021 Blinatumomab Cycle 3 + Imatinib 600 mg/d.
12.10.2021 BMA: cytol. CR, FISH neg.; BCR::ABL ~0,0025%.
20.10.2021 Intrathecal Triple (fluoroscopy-guided): no blasts.
22.10.2021 Blinatumomab Cycle 4 + Imatinib 600 mg/d.
24.11.2021 BMA: cytol. CR, FISH neg.; BCR::ABL ~0,013%; Kiel MRD-.
03.12.2021 Blinatumomab Cycle 5 + Imatinib 600 mg/d.
27.12.2021 BMA: cytol. CR (3% blasts); BCR::ABL ~0,030%; FACS MRD-, Kiel MRD-.
21.01.2022 Blinatumomab Cycle 6 + Imatinib 600 mg/d.
22.02.2022 BMA: cytol. CR (4% blasts); BCR::ABL ~0,010%; FACS MRD-, Kiel MRD-.
02.03.2022 Tumorboard: Blinatumomab maintenance every 12 weeks (to 1 year); Imatinib 600 mg/d long-term to Relapse/Tox.
14.04.2022 Intrathecal Triple CT-guided.
18.04.2022 Blinatumomab Cycle 7 + Imatinib 600 mg/d.
27.05.2022 Remission assessment (BMA): Punctio sicca; BCR::ABL ~0,041%; FACS MRD-, Kiel MRD-.
08.07.2022 Blinatumomab Cycle 8 + Imatinib 600 mg/d.
12.08.2022 Remission assessment (BMA): CR; BCR::ABL ~0,090%; FACS MRD-, Kiel MRD-.
04.09.2022 Switch to Dasatinib 100 mg 1-0-0 with peripheral edema.
29.09.2022 Remission assessment (PB): BCR::ABL ~0,03%; Kiel MRD-.
18.10.2022 Switch to Bosutinib 200 mg 1-0-0 with rash/pruritus.
20.11.2022 Remission assessment (BMA): CR; BCR::ABL ~0,011%; FACS MRD-, Kiel MRD-.
11.03.2023 Remission assessment (BMA): CR; BCR::ABL ~0,145%; FACS MRD-, Kiel MRD-.
25.03.2023 Currently: Suspected progression.
================================================================================

================================================================================
TREATMENT TIMELINE
================================================================================
Gene Transcript CDS position Amino acid position Allele frequency (%) COSMIC ID cBio-Portal Pathogenicity
KRAS NM_033360.4 c.35G>A p.G12D 25-45 COSM521 yes pathogenic
================================================================================

# l_case_11

Age: 71

ECOG: 0

Primary diagnosis:
Suspected MDS, ID 01/2026
Initial manifestation with anemia
IPSS-M low, IPSS-R low
Bone marrow histologically and cytomorphologically mildly hypocellular with Dysplasia of the
erythropoiesis, granulopoiesis and megakaryopoiesis as well as prominent erythropoiesis.
No blast excess, blast percentage 4%
None ring sideroblasts detectable.
Immunocytologically no definite evidence of a myeloid neoplasm.
Molecular genetic unremarkable findings
Cytogenetically normal male karyotype (46,XY)

Secondary diagnoses:
None

Treatment and course:
None

Question for the tumor board:
Recommended management?

Tumor board decision:
The suspicion of MDS can neither be confirmed nor excluded. Regular follow-up and further workup the anemia. With symptomatic anemia or Hb <10g/dl Therapy with ESA.

# l_case_12

Age: 62

ECOG: 1

Primary diagnosis:
CML ID 01/2026
ELTS-Score 2,935
Left shift, leukocytosis 210/nl, neutrophilia, basophilia, 13% blasts.
FISH: BCR-ABL pending

Secondary diagnoses:
None

Treatment and course:
12/25 Bosutinib 400mg/d
01/26 Switch to Dasatinib 100mg/d with intolerance
PD

Question for the tumor board:
Recommended management?

Tumor board decision:
Switch Dasatinib on Asciminib; alternatively Ponatinib, Recommendation for resistance testing. Allo-SCT to be planned.

# l_case_13

Age: 60

ECOG: 0

Primary diagnosis:
Acute myeloid leukemia ID 01/26
BM 01.01.2026: 80% blasts,
FACS 01.01.2026: CD33+, CD34+, CD13+, HLA DR+, CD117+, no Detection lymphoid markers
Genetics: NPM1 WT, FLT3 TKD neg., remaining results pending
FISH: no evidence of a MECOM, KMT2A or CBFB Rearrangement
ELN: final assessment pending
Secondary diagnoses:
None

Treatment and course:
Cytoreductive therapy with Hydroxyurea since 01.01.2026

Question for the tumor board:
Recommended management?

Tumor board decision:
Start 7+3+GO. Referral Allo-SCT to be planned.

# l_case_14

Age: 62

ECOG: 1

Primary diagnosis:
c-ALL, BCR-ABL positive ID 01/2025
PB 01/25: CD19+, CD10+, CD34+, TdT+, CD79a+, CD22+
FISH: BCR-ABL-Rearrangement t(9;22),

Secondary diagnoses:
Type 2 diabetes mellitus

Treatment and course:
01/25 GMALL-EVOLVE study: Pre-phase (Cyclophosphamid + Dexamethason + Ponatinib)
01/25 Induction I (Vincristin + Dexamethason + Ponatinib)
03/25 Consolidation I (Vindesin, HD-MTX, AraC, Dexamethason, Ponatinib)
Currently: MRD-negative CR

Question for the tumor board:
Treatment proposal? Allo-SCT is declined by the patient.

Tumor board decision:
Allo-SCT currently not feasible. TKI (Ponatinib) to continue. If applicable, addition of Blinatumomab.

# l_case_15

Age: 31

ECOG: 1

Primary diagnosis:
TLBL ID 01/25
CT H/T/A: extensive mediastinal mass
Histology: T-cell precursor neoplasm in the sense of of a TLBL
FACS: CD4/CD8; CD7+

Treatment and course:
01/25 Treatment initiation GMALL
06/25 Consolidation IV
Currently: PD

Question for the tumor board:
Recommended management?

Tumor board decision:
indication for allogeneic stem cell transplant conference. Bridging therapy with Nelarabin +/- Cyclo.

# l_case_sim_1

Age: 67

ECOG: 2

Primary diagnosis:
Acute myeloid leukemia with myelodysplasia-related changes (MRC), ID 05.2023
Peripheral blood: anemia, thrombocytopenia, Leukocytes 8500 /µl, approx. 75 % blasts
BMA: AML (M1 after FAB), 85% blast infiltration, predominantly CD34+.
FACS: CD34+, CD117+, HLA-DR+, CD33+, CD13+/-, CD7-; 78% in the blast gate
Cytogenetics: Complex karyotype: 44, XX, -5, del(7)(q22), -17, add(3)(q26), +8 [7]/46, XX [3]
Molecular genetics: FLT3-ITD neg., NPM1 WT, IDH1/2 WT, CEBPA WT, TP53 mutated (VAF 52%), ASXL1 mutated, SRSF2 mutated
ELN classification: adverse risk profile

Secondary diagnoses:
Type 2 diabetes mellitus
COPD GOLD II

Treatment and course:

Question for the tumor board:
Relapse therapy?

Tumor board decision:
Salvage therapy with VEN+HAM and upon achieving a new remission 2. allogeneic stem cell transplantation

================================================================================
TREATMENT TIMELINE
================================================================================
05.10.2021 Induction I: Cytarabin + Daunorubicin (7+3)
26.10.2021 BKP: refractory course, complete BM-infiltration
02.11.2021 Salvage: Azacitidin + Venetoclax (Cycle 1)
16.11.2021 BMA (early): ~10% blasts (cytol.)
02.12.2021 BMA d30: ~10% blasts (cytol.), FACS ~6%
08.12.2021 Azacitidin + Venetoclax (Cycle 2)
05.01.2022 BMA: cytol. CR; FISH negative; FACS-MRD positive (~0,5%)
17.01.2022 Azacitidin + Venetoclax (Cycle 3; VEN d1-21)
12.02.2022 Unrelated allogeneic PBSCT (TBI 8 Gy/Flu/ATG); Cell product (02.02.2022): 6,1x10^6 CD34+/kg
05.03.2022 Day +21: skin-aGvHD Grade I, resolving with local therapy
12.03.2022 Day +28 BMA: cytol. CR; MRD negative; FISH negative; Chim BM ~2% recipient
20.03.2022 Maintenance: Azacitidin + Venetoclax (Cycle 1)
18.04.2022 Maintenance (Cycle 2); GI symptoms: GvHD excluded; H. pylori + Candida
16.05.2022 Maintenance (Cycle 3)
23.05.2022 Day +100 BMA: ~3% blasts; MRD/FISH negative; Chim BM/PB 100% donor; Start immunosuppression tapering
13.06.2022 Maintenance (Cycle 4; Venetoclax 200 mg/Day)
05.07.2022 Immunosuppression completed
08.07.2022 Maintenance (Cycle 5; Venetoclax 200 mg/Day)
27.07.2022 cGvHD joints mild-to-moderate; Steroid; oral candidiasis
03.08.2022 Maintenance (Cycle 6; Azacitidin d1-5 + Venetoclax 100 mg/Day)
11.08.2022 Day +180 BMA: ~3% blasts; FACS-MRD negative
14.02.2023 (+12 M) BMA: ~3% blasts; MRD/FISH negative; Chim 100% donor; CD4 ~115/µl; mild cGvHD (joints/eyes, sicca)
08.08.2023 (+18 M) BMA: ~3% blasts; MRD/FISH negative; Chim 100% donor; mild-to-moderate cGvHD (joints/eyes/oral)
18.12.2023 Chimerism PB: 100% donor; continued mild cGvHD (joints/fascia)
15.02.2024 (+24 M) BMA: cytol. CR; MRD/FISH negative; Chim 100% donor; mild cGvHD; Maintenance (Venetoclax) completed

26.06.2024 Relapse: PB L 0,9/nl, T 97/nl, Hb 12,6 g/dl; BMA ~75% blasts; FACS pending;
FISH: del(5q), Aberr. chr7/17p, +11q; Chim BM 15-25% recipient, PB 5-15% recipient;
================================================================================

# l_case_1__ALL

################################
#### CASE: 1

################################

**================================================= gpt-5-mini (agent) =================================================**

**===== run_1 =====**

**Tumor board decision:**
Recommendation: If the patient is suitable for intensive care, a conventional induction 7+3 (cytarabine + daunorubicin) plus gemtuzumab ozogamicin (day 1/4/7) can be carried out without any problems after a successful leukocyte reduction/prephase. For CR: consolidative HiDAC (1.5 g/m2 days 1-3, 3 cycles). For patients not suitable for intensive care: azacitidine + venetoclax or participation in the study. Primary allo-SCT not indicated in CR1.

**Rationale:**

**Rationale: In-frame bZIP-CEBPA corresponds favorably to ELN; Flowchart/guidelines recommend 7+3 + GO and then HiDAC for younger patients requiring intensive treatment. Current hyperleukocytosis requires a prephase (hydroxyurea, possibly zytarabine) before the start of induction. HMA+Ven as a valid alternative if intensive fitness is lacking; allo-SCT only in case of recurrence/high-risk.**

**===== run_2 =====**

**Tumor board decision:**
Continuation of cytoreduction (HU if necessary until WBC controlled) and, if the patient is capable of intensive therapy, immediate intensive induction 7+3 (cytarabine + daunorubicin) with a single administration of gemtuzumab ozogamicin in induction I. For CR: consolidative HiDAC 1.5 g/m2 days 1-3, a total of 3 cycles. HLA typing/donor search early; allo-SCT not primarily recommended. HMA+Venetoclax only for non-fitness or study.

**Rationale:**
CEBPA bZIP in-frame corresponds to ELN favorable; Standard is intensive induction +/- GO (guidelines/flowchart). High leukocytosis requires prephase (HU) until safe onset is possible. According to CR, HiDAC (1.5 g/m2) is the consolidation standard. Allo only in high-risk/refractoriness; HMA+Venetoclax for non-intensive candidates.

**===== run_3 =====**

**Tumor board decision:**
Recommendation: For patients requiring intensive treatment, immediate intensive induction with 7+3 (cytarabine days 1-7 + daunorubicin days 3-5) after ensuring adequate leukocyte control/leukoreduction phase. If age <60 and no contraindication, GO (Induction I). For CR, consolidating HiDAC (1.5 g/m2 D1-3, 3 cycles). Primary allo-SCT not recommended in CR1. If not suitable for intensive care: azacitidine+venetoclax or study participation.

**Rationale:**
CEBPA bZIP in-frame = ELN favorable; The best curative strategy for fit patients is intensive 7+3 +/- GO followed by HiDAC. So far only hydroxyurea has been given for cytoreduction. Allo-SCT in CR1 not indicated if risk is favorable. HMA+Venetoclax or study option if not suitable for intensive care.

**================================================= gpt-5-mini (plain) =================================================**

**===== run_1 =====**

**Tumor board decision:**
Induction AML with CEBPA double mutations: 7+3 induction (daunorubicin 60-90 mg/m2 days 1-3; cytarabine 100-200 mg/m2 days 1-7). After CR/CRi: HiDAC consolidation. Upfront allo-transplant only in case of relapse or MRD-positive high-risk; If the risk is favorable, no upfront transplant.

**Rationale:**
CEBPA double mutations are considered a favorable AML risk; Standard induction + HiDAC consolidation achieves good remission rates. Allo-transplantation in first remission is not mandatory if the risk profile is good; Consider MRD-driven decisions in relapse.

===== run_2 =====

**Tumor board decision:**
Induction therapy AML 3+7: Daunorubicin 60 mg/m2 d1-3 + Cytarabine 100-200 mg/m2/d d1-7 (alternatively idarubicin 12 mg/m2 d1-3); after CR HiDAC consolidation. Allotransplantation only if MRD positive or relapse.

**Rationale:**
CEBPA double mutation + FLT3 ITD neg -> favorable risk (ELN). No other high-risk factor. Target CR; Transplantation usually not recommended in first remission; Transplant only if MRD positive/relapse.

===== run_3 =====

**Tumor board decision:**
Induction therapy AML: 7+3 induction (Daunorubicin 60 mg/m2 days 1-3 + Cytarabine 100-200 mg/m2 days 1-7). After CR MRD negativity, subsequent HiDAC consolidation; Allogeneic transplantation in first remission only in cases of MRD positivity or clinical high-risk.

**Rationale:**
favorable ELN risk due to CEBPA biallelic mutations, normal karyotype, no FLT3-ITD; therefore induction recommended, aiming for CR/MRD negativity; Establish HiDAC consolidation; SCT in first remission only in MRD positivity or high-risk manifestations.

## ======================================================== gpt-5-nano (agent) ========================================================

===== run_1 =====

**Tumor board decision:**
Induction: standard 7+3 (cytarabine + daunorubicin) in CEBPA double mutations/ELN-favored AML; If you are fit (<60J), consider GO on days 1,4,7. Post-CR MRD-guided consolidation with HiDAC (1.5 g/m2, days 1-3, 3 cycles). Allo-SCT in ER only in MRD persistence or high-risk; Check donors early.

**Rationale:**
CEBPA double mutations are ELN favored; MRD response determines further steps. 7+3 is established; GO only if you are fit. Consolidation via HiDAC makes sense; allo-SCT in ER only risk-adapted in cases of MRD persistence or high-risk; Check the donor pool early.

===== run_2 =====

**Tumor board decision:**
Induction: 7+3 (cytarabine 7d, daunorubicin 3d). GO possible at <60J. Consolidation controlled by MRD with HiDAC 1.5 g/m2, 3 cycles at CR. Allo-SCT first remission: no standard with favorable ELN (CEBPA); MRD positivity/relapse -> Check donor.

**Rationale:**
CEBPA-doublemut + favorable ELN -> good prognosis with chemo; Allo-SCT only after MRD positivity/relapse; Donor availability taken into account.

===== run_3 =====

**Tumor board decision:**
Induction: 7+3 in CEBPA double mutant (bZIP-in-frame); GO optional if age appropriate. After CR/CRi MRD-guided consolidation with HiDAC 1.5 g/m2 days 1-3, a total of 3 cycles. Allo-SCT in first remission only with MRD-positive residual disease or lack of MRD negativity; screen donors early; if ELN is favorable, no routine allo-SCT.

**Rationale:**
CEBPA double mutant AML with favorable ELN risk; high CR rate with standard induction. MRD status is the key predictor of further steps. Routine allo-SCT in first remission is not recommended if the risk is favorable; Residual MRD-positive or loss of MRD-negativity after consolidation increases need for transplantation; GO option dependent on age/CD33.

## ======================================================== gpt-5-nano (plain) ========================================================

===== run_1 =====

**Tumor board decision:**

Induction AML now: 3+7 standard (Daunorubicin 60 mg/m2 d1-3 + Cytarabine 100-200 mg/m2 d1-7). Target remission. Then HiDAC consolidation (e.g. Cytarabine 3 g/m2 d1.3.5 x 3-4 cycles). SCT in CR1 only if MRD positive/relapse; otherwise not with this favorable CEBPA mutation profile.

**Rationale:**
CEBPA double mutant AML belongs to favorable ELN risk; hence standard induction and MRD-guided consolidation. Allogeneic transplantation in CR1 recommended only in cases of MRD-positive findings or relapse; If the prognosis is otherwise good, no SCT in CR1. Hydroxyurea was cytoreduction, not a contraindication.

**===== run_2 =====**

**Tumor board decision:**
Induction chemotherapy 7+3 for those capable of treatment: Daunorubicin 60 mg/m2/day 1-3 + Cytarabine 100-200 mg/m2/day 1-7. Target remission. Then MRD-guided consolidation with high dose Cytarabine (e.g. 3 g/m2 q12h on days 1,3,5). Allogeneic transplantation only in MRD-positive remission or relapse.

**Rationale:**
CEBPA-mut AML (in-frame), ELN-favorable, no FLT3-ITD, normal KK; good remission rates with standard induction. Hydroxyurea was cytoreduction. MRD-supported consolidation makes sense; Transplantation not routinely in first remission with favorable risk.

**===== run_3 =====**

**Tumor board decision:**
Induction now: 7+3 (Daunorubicin 60 mg/m2 d1-3 + Cytarabine 100-200 mg/m2/d1-7) followed by HiDAC consolidation (3 cycles). SCT in first remission only with unfavorable features; MRD-supported decisions.

**Rationale:**
CEBPA-biallelic, favorable ELN risk; If you are sufficiently fit, you have the highest chance of recovery with intensive chemotherapy; HU bridge completed; SCT only if remission/MRD factors are unfavorable.

## ======================================================= gpt-oss-20b (agent) =======================================================

**===== run_1 =====**

**Tumor board decision:**
Yes, immediate 7+3 induction (Daunorubicin 60mg/m2d3-5, Cytarabine 100mg/m2i.v. continuously d1-7) plus gemtuzumab-ozogamicin (GO) 3mg/m2 d1,4,7. Discontinue hydroxyurea.

**Rationale:**
AML with bZIP-in-frame CEBPA, favorable risk -> ELN/ICC recommend 7+3 + GO. No contraindications, patient stable, no previous intensive therapy. Induction is standard.

**===== run_2 =====**

**Tumor board decision:**
Start 7+3 induction (Daunorubicin 60 mg/m2 days 3-5, Cytarabine 100 mg/m2 days 1-7). If patient <60 yrs, add Gemtuzumab-Ozogamicin 3 mg/m2 on days 1, 4, 7. No contraindications; favorable-risk CEBPA AML supports standard induction.

**Rationale:**
Favorable-risk AML with bZIP-inframe CEBPA; flowchart recommends 7+3 +/- GO. Hydroxyurea was only cytoreduction; no prior ineffective therapy. Age unknown - GO added if <60 to improve CR rates. No comorbidities or contraindications noted.

**===== run_3 =====**

**Tumor board decision:**
Yes, initiate induction with 7+3 (Cytarabine 100mg/m2Day1-7 + Daunorubicin 60mg/m2Day3-5) plus gemtuzumab-ozogamicin (Day1/4/7).

**Rationale:**
AML with bZIP-in-frame CEBPA, <60y, ELN-favorable risk, no contraindications. Hydroxyurea was only for leukostasis; According to the flowchart 7+3+GO is the recommended first-line therapy.

## ================================================== gpt-oss-20b (plain) ==================================================

##### ===== run_1 =====

**Tumor board decision:**
Begin induction with 7+3 (7 days cytarabine 100 mg/m2 + 3 days daunorubicin 60 mg/m2).

**Rationale:**
AML with bZIP CEBPA double mutation = favorable ELN risk; standard induction is recommended. No prior cytotoxic therapy, hydroxyurea only for cytoreduction. Post-induction consolidation per protocol; allo-SCT not indicated.

##### ===== run_2 =====

**Tumor board decision:**
Start a standard induction with 3+7 (idarubicin 12mg/m2t1-3 + cytarabine 100mg/m2t1-7). After remission, 2-3 consolidations are carried out with high-dose cytarabine. An allograft is not absolutely necessary in initial remission.

**Rationale:**
AML with CEBPA mutation is considered a favorable risk; no prior cytotoxic therapy. 3+7 is the proven initial treatment. In case of remission with CEBPA double mutation, consolidation with HD-Ara-C is sufficient; Allo-HSCT only in case of relapse or insufficient remission.

##### ===== run_3 =====

**Tumor board decision:**
Start a standard induction curve 7+3 (Cytarabine 100mg/m2 IV 7days + Idarubicin 12mg/m2 IV 3days). After achieving CR, evaluate for a possible allotransplantation, but if the CEBPA double mutation has a favorable risk, immediate transplantation is not necessary.

**Rationale:**
AML with CEBPA mutation, favorable ELN risk, no prior induction, hydroxyurea only for blast reduction. 7+3 is proven first-line therapy, high CR rate, minimal additional complications. Transplantation only in case of persistence or relapse.

## ================================================== gpt-oss-120b (agent) ==================================================

##### ===== run_1 =====

**Tumor board decision:**
Induction therapy with 7+3 (cytarabine day 1-7, daunorubicin day 3-5) combined with gemtuzumab-ozogamicin (GO) on days 1, 4 and 7.

**Rationale:**
CEBPA-bZIP in-frame mutation and favorable ELN risk status correspond to INT-1a in the flowchart; GO improves CR rate in CEBPA-mutated AML, while 7+3 represents the standard intensive induction. Hydroxyurea prephase has already been completed.

##### ===== run_2 =====

**Tumor board decision:**
Induction therapy: 7+3 regimen (daunorubicin 60mg/m2 d1-3, cytarabine 100-200mg/m2/day d1-7) combined with gemtuzumab-ozogamicin 3mg/m2 on days 1, 4 and 7 (at age <60 years and good organ function).

**Rationale:**
CEBPA bZIP mutation and favorable ELN risk make the disease a candidate for GO supplementation, increasing the CR rate. No FLT3/NPM1 mutations, normal karyotype, so 7+3 is sufficient; GO is recommended as long as age/comorbidity permits.

##### ===== run_3 =====

**Tumor board decision:**
Induction therapy: 7+3 (cytarabine 100mg/m224h d1-7, daunorubicin 60mg/m2 d1-3) combined with gemtuzumab-ozogamicin (3mg/m2 on days 1, 4 and 7).

**Rationale:**
CEBPA-bZIP-mutation and favorable ELN score are considered an indication for 7+3+GO according to flowchart-INT-1a. After hydroxyurea cytoreduction, normal karyotype and absence of FLT3 mutation,

intensive induction is recommended; GO increases the CR rate in this subgroup.

## ================================================== gpt-oss-120b (plain) ==================================================

**===== run_1 =====**

**Tumor board decision:**
Start intensive induction chemotherapy (e.g. 7+3: Cytarabine 100-200mg/m2 24h infusion D1-7 + Daunorubicin 60mg/m2 D1-3) followed by high-dose Cytarabine consolidation. Allogeneic transplant only in refractory disease or high MRD.

**Rationale:**
AML has a double CEBPA mutation and a favorable ELN risk profile. Without contraindications, curative, intensive therapy is standard as it achieves the best remission and survival rates; A transplant is not necessary if the prognosis is good.

**===== run_2 =====**

**Tumor board decision:**
After hydroxyurea cytoreduction, start induction chemotherapy according to the 7+3 regimen (cytarabine 100-200mg/m224h x7days + daunorubicin 60mg/m2day1-3), optionally with gemtuzumab-ozogamicin because of CD33 positivity. In case of remission, consolidation with high-dose cytarabine occurs; allogeneic SCT only for refractory disease or relapse.

**Rationale:**
AML with double CEBPA mutation has favorable ELN risk profile; Standard induction 7+3 leads to high remission rates. GO can improve response in CD33 positive cases. In CR, if the risk is favorable, SCT is not necessary in the first remission, but HD-Ara-C consolidation.

**===== run_3 =====**

**Tumor board decision:**
Induction with 7+3 (cytarabine 100-200mg/m2 24h x7days + daunorubicin 60mg/m2 on days 1-3) after hydroxyurea bridging, then MRD control and consolidation with 3-4 cycles of high-dose cytarabine (Hi-DAC). Allogeneic SCT only in case of recurrence or high MRD level.

**Rationale:**
CEBPA double mutant AML is a favorable risk after ELN. Standard induction chemotherapy achieves >80% CR, while transplant in first remission increases mortality. Hi-DAC consolidation reduces the risk of relapse. Hydroxyurea was only used to reduce blasts before induction.

## ==================================================== qwen3-32b (agent) ====================================================

**===== run_1 =====**

**Tumor board decision:**
Recommendation: Start induction therapy with 7+3 (cytarabine + daunorubicin) according to AML-INTENSIVE regimens, supplemented with gemtuzumab ozogamicin (GO) if the age is <60 years. If age is missing: 7+3 without GO. Prerequisite: Prephase successfully completed.

**Rationale:**
The CEBPA-bZIP double mutation and the favorable ELN risk profile justify intensive induction. The flowchart suggests 7+3 + GO in CEBPA-mutated AML (<60 J). Since age is not specified, 7+3 is recommended as standard. Prephase was completed by hydroxyurea.

**===== run_2 =====**

**Tumor board decision:**
Therapy should be started with standardized induction therapy ("7+3"), supplemented with gemtuzumab ozogamicin (GO) due to the CEBPA double mutation and favorable ELN risk profiling.

**Rationale:**
The CEBPA bZIP mutation and favorable ELN risk justify intensive induction. The flowchart recommends the combination of 7+3 and GO for CEBPA mutations ("bZIP in-frame"). Previous cytoreduction with hydroxyurea reduced leukocytosis sufficiently to allow induction.

**===== run_3 =====**

**Tumor board decision:**
Yes, therapy with standardized induction therapy (7+3 + gemtuzumab ozogamicin) should be started.

**Rationale:**
AML with CEBPA double mutation (in-frame bZIP and loss-of-function) is one of the most favorable risk groups after ELN. After successful cytoreduction with hydroxyurea, intensive treatment induction (7+3 + GO) is indicated, as CEBPA-mutated AML under 60 years of age is treated in a standardized manner according to the flowchart [INT-1a]. The absence of FLT3-ITD, NPM1 mutations and unfavorable cytogenetics support the risk-adaptive therapy recommendation.

## ======================================================== qwen3-32b (plain) ========================================================

**===== run_1 =====**

**Tumor board decision:**
Recommendation: Start induction chemotherapy according to ELN guidelines for favorable risk AML

**Rationale:**
CEBPA double mutation with bZIP domain and loss-of-function has a favorable prognosis. ELN risk classification confirms favorable risk. After cytoreduction with hydroxyurea, induction chemotherapy (e.g. cytarabine + anthracycline) is standard. No contraindications or recurrent mutations known.

**===== run_2 =====**

**Tumor board decision:**
Recommendation: Initiate induction chemotherapy with 7+3 regimen (cytarabine + anthracycline).

**Rationale:**
AML with CEBPA double mutation and favorable ELN risk profile requires standardized induction chemotherapy. Hydroxyurea was only cytoreductive. No known contraindications.

**===== run_3 =====**

**Tumor board decision:**
Recommendation: Initiate induction chemotherapy with 7+3 regimen (cytarabine + anthracycline). If addressed, plan the following consolidation.

**Rationale:**
CEBPA double mutation (bZIP + LOF) indicates favorable ELN risk. Standard therapy for favorable risk AML is induction chemotherapy. Molecular profiles (CEBPA-mut, FLT3-ITD-negative) support prognosis. No targeted therapy indicated.

## ====================================================== qwen3-next-80b (agent) ======================================================

**===== run_1 =====**

**Tumor board decision:**
No, induction therapy only after checking the leukocytes in the prephase. Currently WBC is >20,000/µl, so continue the prephase and re-evaluate daily.

**Rationale:**
According to the flowchart (AML-GATE-1), a prephase is required for leukocytes >20,000/µl. Hydroxyurea has already been given for 10 days, but current WBC status is unclear. Induction can only be started after the leukocytes have been checked and leukostasis has been ruled out.

**===== run_2 =====**

**Tumor board decision:**
Yes, initiate standard induction (7+3). For patients <60 years of age, additional gemtuzumab ozogamicin (GO) in induction I.

**Rationale:**
ELN favorable risk due to bZIP in-frame CEBPA mutation. Flowchart AML-INTENSIV INT-1a recommends 7+3 + GO for <60 years. No comorbidities, intensive therapy indicated. Age unknown, but prephase with HU completed, leukocytes under control. GO add-on for young people compliant with standards.

**===== run_3 =====**

**Tumor board decision:**
No, do not initiate standard induction (7+3). Continue cytoreduction until leukocytes <20,000/μl and clinically stable. Check current WBC after 10d HU; if hyperleukocytosis persists, switch to cytarabine prephase or leukapheresis.

**Rationale:**
Patient with initial WBC 62,000/μl (hyperleukocytic). Flowchart requires prephase with WBC >20,000/μl before induction. 10d HU alone is not enough to start induction. Only initiate 7+3+GO (for bZIP-CEBPA mutation) after WBC control and stabilization.

## ================================================ qwen3-next-80b (plain) ================================================

**===== run_1 =====**

**Tumor board decision:**
Start intensive induction therapy with 7+3 regimen (cytarabine 7 days + daunorubicin/idarubicin 3 days).

**Rationale:**
favorable ELN risk due to CEBPA-bZIP mutation. Hydroxyurea was only preliminary cytoreduction. Standard therapy for fit patients with AML CEBPA mutation is intensive induction. No contraindications or comorbidities. Targeted therapies not indicated (FLT3-/NPM1-neg.).

**===== run_2 =====**

**Tumor board decision:**
Start intensification with standard induction therapy (7+3 regimen: cytarabine 7 days + daunorubicin 3 days).

**Rationale:**
Favorable risk after ELN (CEBPA mutation), no contraindications. Hydroxyurea was only preliminary cytoreduction. Standard induction is curative, no targeted therapy is necessary (FLT3/NPM1 negative).

**===== run_3 =====**

**Tumor board decision:**
Initiate standard induction therapy with 7+3 regimen (cytarabine 7 days, daunorubicin 3 days).

**Rationale:**
Favorable risk after ELN (CEBPA-bZIP mutation), no contraindications. Hydroxyurea was only temporary cytoreduction. Intensive induction is standard in fit patients with good risk AML.

# l_case_2__ALL

################################
#### CASE: 2
################################

## ========================================================= gpt-5-mini (agent) =========================================================

**===== run_1 =====**

**Tumor board decision:**
Start hydroxyurea orally immediately: 4 g/day (2 tablets of 500 mg, 4x daily) for rapid cytoreduction. ATRA and ATO continue. Leukapheresis contraindicated in APL. If there is no decline after 48-72 hours or there is clinical leukostasis, consider short-term idarubicin according to the APL regimen (e.g. days 1+3).

**Rationale:**
APL with current WBC 65,000/µl -> rapid cytoreduction necessary. Guidelines/flowchart: HU initial measure (>50k: 4 g/d). Leukapheresis contraindicated. Anthracycline only in case of inadequate response or life-threatening leukostasis. Only temporarily pause ATRA/ATO in the case of differentiation syndrome; steroid prophylaxis/for DS dexamethasone 10 mg i.v. q12h. Bleeding prophylaxis: platelet target >=30-50x10^9/L (for bleeding/intervention >=50), fibrinogen >=150 mg/dL, aggressive substitution (FFP/KK/platelets). ECG/QTc monitoring under ATO.

**===== run_2 =====**

**Tumor board decision:**
For PML::RARA+ APL with WBC 65,000/µl: Leukapheresis contraindicated. Immediate cytoreductive: idarubicin immediately (e.g. idarubicin day 1+3) + continue ATRA unchanged; ATO according to the protocol from day 5 (with QTc/EKG ok). If idarubicin is not available in the short term: temporary hydroxyurea (e.g. 4 g/day) to anthracycline. Start with prophylactic steroid (e.g. prednisone 0.5-1 mg/kg p.o. or in case of high-risk/clinical signs dexamethasone 10 mg i.v. bid). Aggressive coagulation optimization: fibrinogen >150 mg/dL, platelets >30-50,000/µL (transfusions, FFP/Cryo as needed). Close clinical checks, daily blood counts, electrolytes, ECG (2x/week for ATO) and monitoring for differentiation syndrome/TLS; If symptoms occur, pause ATRA/ATO and iv dexamethasone.

**Rationale:**
WBC >10,000 defines high-risk in APL -> Recommendation according to APL flowchart: Anthracycline+ATRA; ATO only from day 5. Leukapheresis is contraindicated in APL. Hydroxyurea is permitted as a temporary cytoreduction. Steroids prophylactically to prevent/mitigate the differentiation syndrome; Aggressive coagulation prophylaxis reduces early bleeding and thrombosis risks.

**===== run_3 =====**

**Tumor board decision:**
IMMEDIATELY: Hydroxyurea 500 mg p.o. Start 4x/day (~4 g/day). No leukapheresis (APL contraindication). Continue ATRA immediately; Also continue ATO with close QTc monitoring; Only temporarily pause ATO/ATRA if differentiation syndrome symptoms are manifest. Steroid prophylaxis: Prednisone 0.5 mg/kg/day (alternatively Dexa if clinically suspected). Bleeding prophylaxis: thrombotransfusions so that Plt >30-50x10^9/L, fibrinogen >150 mg/dL (cryo/FFP as required). TLS prophylaxis: rapid hydration, allopurinol; Rasburicase in hyperuricemia. Daily up to 2x daily monitoring of Hb/Plt/WBC, coagulation, electrolytes, ECG. If there is no sufficient drop in WBC by day 3 or if there are signs of leukostasis -> add short-term idarubicin (high-risk regimen, e.g. days 1+3).

**Rationale:**

**Rationale: According to the APL flowchart, leukapheresis is contraindicated. For WBC >50k, hydroxyurea is the first measure for cytoreduction; Idarubicin only in the absence of response or clinical leukostasis. Steroids reduce risk of differentiation syndrome. ATRA/ATO should not be routinely discontinued; Aggressively optimize coagulation parameters and transfusions.**

## ========================================================= gpt-5-mini (plain) =========================================================

**===== run_1 =====**

**Tumor board decision:**
Continue induction (ATO/ATRA) at AML M3; due to WBC 65,000/µl, immediate cytoreduction: hydroxyurea for WBC control; Dexa prophylaxis for differentiation syndrome prevention; close monitoring. If WBC persists high or DS occurs, add daunorubicin/idarubicin as per protocol.

**Rationale:**
WBC >=50-65k classified as high-risk APL; increased risk of differentiation syndrome. Cytoreduction reduces DS risk and allows ATO/ATRA induction to continue.

**===== run_2 =====**

**Tumor board decision:**
Continuation of ATO/ATRA as induction basis; rapid cytoreduction of leukocytosis by hydroxyurea begins; if there is persistent leukocytosis or risk of DS, add additional anthracycline/cytarabine induction (e.g. idarubicin 12 mg/m2 d1-2); Ensure DS monitoring and supportive transfusions.

**Rationale:**
WBC 65x10^9/L meets intermediate risk with high DS risk. ATO/ATRA alone is often not enough here; Cytoreduction reduces the risk of leukemia and DS and improves the probability of remission. Necessary transfusion and supportive measures due to Hb 5 g/dL and thrombocytopenia.

**===== run_3 =====**

**Tumor board decision:**
Induction for high-risk APL (WBC 65x10^9/L): Continue ATO/ATRA + cytoreduction during induction. Daunorubicin 60-90 mg/m2 IV D1-D3 (alternatively idarubicin); prophylactic dexamethasone 10 mg IV q12h; close monitoring for differentiation syndrome; If necessary, hydroxyurea to quickly lower leukocytes.

**Rationale:**
High leukocytosis increases risk of differentiation syndrome. High-risk APL requires adjunctive chemotherapy during induction; ATO/ATRA alone is not enough here; Combination induction improves remission rates and reduces DS and complications.

## ====================================================== gpt-5-nano (agent) ======================================================

**===== run_1 =====**

**Tumor board decision:**
Recommendation: Provide high-risk APL induction. Continuation of ATO/ATRA with immediate cytoreduction. Hydroxyurea 2x500 mg 3x/day; if WBC does not decrease significantly, add idarubicin 10 mg/m2 day 1+3 (+/- Ara-C). ATO continues from day 5; Continue ATRA. Dexa prophylaxis against differentiation syndrome; Monitor QTc closely.

**Rationale:**
WBC 65x10^9/L increases leukostasis/DS risk. In APL with ATO/ATRA, cytoreduction is useful; High-risk induction reduces blast load and DS risk. Hydroxyurea works quickly; Dexa reduces DS incidence; QTc monitoring required. If DS symptoms occur, respond accordingly.

**===== run_2 =====**

**Tumor board decision:**
Induction AML M3 high-risk: Idarubicin day 1+3 + ATRA; ATO from day 5; Hyperleukocytosis with hydroxyurea (e.g. HU 500 mg 4x/day at >50k/µl); Dexamethasone differentiation syndrome prophylaxis (10 mg IV q12h) at least 3-5 days; QTc monitoring.

**Rationale:**
WBC 65 x10^9/L, intermediate risk. High-risk APL requires anthracycline induction, DS prophylaxis essential; HU reduces risk of leukostasis; close monitoring of QTc/electrolytes.

**===== run_3 =====**

**Tumor board decision:**
Induction in high-risk APL (PML-RARA+, t(15;17)) with hyperleukocytosis: idarubicin day 1+3 + ATRA; Continue ATO from day 5; Hydroxyurea for rapid cytoreduction; Ara-C optional depending on compatibility. DS prophylaxis: Dexamethasone 10 mg IV q12h for DS symptoms (prophylactic if necessary); QTc monitoring; TLS prophylaxis.

**Rationale:**

High-risk APL requires additional cytoreduction and DS prevention; Leukostasis risk with WBC 65k. Flowchart recommends induction with anthracycline plus ATRA/ATO; Hydroxyurea rapidly reduces leukocytes; DS/QTc/TLS monitoring minimizes complications.

## ================================================== gpt-5-nano (plain) ==================================================

**===== run_1 =====**

**Tumor board decision:**
High-risk APL: Continuation of ATO/ATRA in combination with early cytoreductive induction using daunorubicin (60 mg/m2; days 1-3). Dexa prophylaxis against differentiation syndrome. Close monitoring; DS management when it occurs.

**Rationale:**
WBC 65k (>10k) classifies high-risk APL. ATO/ATRA alone inadequate; additional anthra induction improves remission rate and enables rapid cytoreduction; Address DS risk through prophylaxis and monitoring.

**===== run_2 =====**

**Tumor board decision:**
Continue induction: pass on ATO/ATRA; Initiate leukocyte lowering immediately (hydroxyurea to quickly reduce WBC). Dexa 10 mg i.v. Start/continue q12h as DS prophylaxis; Monitor DS symptoms closely. If WBC persists at a high level (>50x10^9/L) or DS occurs, promptly add cytoreductive chemo (e.g. idarubicin 12 mg/m2 i.v. q2-3d) according to the protocol.

**Rationale:**
WBC 65x10^9/L increases DS and leukostasis risk in AML M3. ATO/ATRA is standard; Concomitant leukocyte lowering and DS prophylaxis reduce morbidity/mortality. If there is insufficient reduction or DS symptoms, rapid cytoreduction with anthracycline is recommended.

**===== run_3 =====**

**Tumor board decision:**
High-risk APL (t(15;17), PML-RARA) with WBC 65k: Continue induction with ATO/ATRA plus cytoreduction. Preferably daunorubicin or idarubicin for induction; alternatively hydroxyurea to quickly lower WBC. Prophylactic dexamethasone against differentiation syndrome; close monitoring (DS, QTc).

**Rationale:**
WBC 65k = high-risk. ATO/ATRA alone is not enough here; Early cytoreduction reduces the risk of leukostasis and DS. Dexa prophylaxis and monitoring are essential for DS management.

## ================================================= gpt-oss-20b (agent) ==================================================

**===== run_1 =====**

**Tumor board decision:**
With WBC=65000/µl the patient is clearly in the high-risk segment (APL-Sanz). We stop the current ATO/ATRA therapy and start a high-risk induction with idarubicin 12 mg/m2 day 1-3 + ATRA (continued). ATO will be reintroduced from day 5. Plan for hydroxyurea to reduce leukostasis and, if necessary, leukapheresis.

**Rationale:**
APL flowchart defines high-risk (WBC>10000) as indicator for idarubicin-ATRA regimen; ATO/ATRA alone is not enough at 65k. Hydroxyurea reduces immediate leukostasis. This strategy is consistent with current guidelines and increases the CR rate in high WBC patients.

**===== run_2 =====**

**Tumor board decision:**
We recommend switching to high-risk APL induction: discontinue ATO, start idarubicin 12 mg/m2 days 1 & 3 + ATRA 45 mg/m2 bid, add hydroxyurea for leukostasis, consider 24-h cytarabine 100 mg if WBC > 50k. ATO may be re-started day 5 if tolerated.

**Rationale:**
Sanz risk = high (WBC65k). Flowchart APL-1b specifies idarubicin+ATRA for high-risk; ATO only from day 5. Cytarabine is not part of standard APL induction and may increase toxicity. Hydroxyurea controls leukostasis.

===== run_3 =====

**Tumor board decision:**
If WBC>50000/µl (high-risk according to Sanz), the induction of ATO/ATRA is replaced by a high-risk regimen: Idarubicin12mg/m2t1-3 + ATRA45mg/m2t1-30, ATO from day 5. Hydroxyurea for leukocytosis control remains optional. Cytarabine is not included in induction.

**Rationale:**
The flowchart defines idarubicin+ATRA (ATO from day 5) for high-risk APL (WBC>50k). ATO/ATRA alone up to 60 days only applies to low/intermediate. WBC increase therefore requires high-risk induction. Cytarabine is not intended.

## ================================================== gpt-oss-20b (plain) ==================================================

===== run_1 =====

**Tumor board decision:**
Induction with ATRA + ATO + daunorubicin (or cytarabine) for hyperleukocytosis (WBC 65000/µl). If necessary, leukapheresis for rapid WBC reduction.

**Rationale:**
APL therapy requires an additional cytotoxic component when WBC>10,000/µl. ATRA+ATO alone is sufficient if the risk is low; for intermediate risk and hyperleukocytosis, daunorubicin (or cytarabine) is standard. Leukapheresis controls acute hyperleukocytosis.

===== run_2 =====

**Tumor board decision:**
Add idarubicin 12 mg/m2 IV days 1-3 to the current ATRA/ATO induction for high-WBC APL (AML M3). Continue ATRA/ATO and monitor counts.

**Rationale:**
WBC65000/µl classifies the patient as high-risk APL; guidelines recommend ATRA+ATO+anthracycline induction. Idarubicin improves CR rates and reduces relapse risk. No contraindications noted.

===== run_3 =====

**Tumor board decision:**
Start induction with ATRA (45 mg/m2) + ATO (0.15 mg/kg) plus low-dose cytarabine (20 mg/m2 q12 h) until CR. If WBC > 10 k, add idarubicin 12 mg/m2 q24 h. Begin hydroxyurea 15 mg/kg/day until WBC < 10 k. Monitor for differentiation syndrome.

**Rationale:**
APL requires ATRA+ATO; high WBC (>10k) is high-risk according to Sanz, warranting anthracycline. Low-dose cytarabine and hydroxyurea control leukocytosis. This regimen follows current guidelines and addresses the patient's leukocytosis.

## ================================================== gpt-oss-120b (agent) ==================================================

===== run_1 =====

**Tumor board decision:**
Add hydroxyurea for rapid cytoreduction (e.g. 2x500 mg 4 times/day until WBC<20000/µl), continue ATRA+ATO unchanged, start prophylactic prednisone/dexamethasone (10 mg/m2 i.v.>=3 days), close QTc monitoring; If there is no reduction in WBC, adjust the dose and avoid leukapheresis.

**Rationale:**
APL with t(15;17) is treated via ATRA/ATO. Hyperleukocytosis is a known side effect and is controlled with hydroxyurea according to the flowchart. Steroid prophylaxis prevents differentiation syndrome. ATO requires QTc monitoring. Leukapheresis is contraindicated in APL.

===== run_2 =====

**Tumor board decision:**
Continuation of ATRA/ATO induction with immediate cytoreduction: Hydroxyurea 2x500 mg 4 times/day until WBC <20,000/µl, at the same time dexamethasone 10 mg/m2 i.v. q12h >=3 days (or until differentiation syndrome subsides). QTc control 2 times/week, no APL leukapheresis. In the case of

clinical leukostasis, emergency leukapheresis may be necessary after stabilization.

**Rationale:**
Persistent hyperleukocytosis (>50,000/µl) is defined in the APL flowchart as transient leukocytosis that must be controlled with hydroxyurea. Steroid prophylaxis prevents differentiation syndrome. ATRA/ATO must not be interrupted; QTc monitoring due to ATO. Leukapheresis only in cases of pronounced leukostasis, otherwise it is contraindicated.

**===== run_3 =====**

**Tumor board decision:**
Start hydroxyurea cytoreduction (>=50,000/µl: 2x500mg 4 times daily) and at the same time dexamethasone 10mg/m2 i.v. q12h >=3days; Continue ATRA/ATO, pause if differentiation syndrome occurs; Intensify QTc and coagulation controls; Leukapheresis only in clinical leukostasis.

**Rationale:**
Patient with t(15;17)-APL in ATRA/ATO induction shows hyperleukocytosis (65000/µl). According to flowchart APL-3, hydroxyurea is indicated for rapid WBC reduction and dexamethasone for prevention/therapy of differentiation syndrome. Continuation of targeted therapy is essential while QTc and coagulation parameters are closely monitored.

## ================================================== gpt-oss-120b (plain) ==================================================

**===== run_1 =====**

**Tumor board decision:**
Continuation of ATRA+ATO because the diagnosis of APL (t(15;17)) is present. Due to the high leukocyte count (>=10,000/µl), an anthracycline induction (e.g. idarubicin 12mg/m2 i.v.) is added on day 1-2. Prophylactic dexamethasone 10mg i.v. q12h for 7 days and if necessary hydroxyurea for rapid leukocyte reduction.

**Rationale:**
APL with t(15;17) responds excellently to ATRA/ATO; With high-risk WBC values, an anthracycline component is necessary to achieve remission and prevent differentiation syndrome. Dexamethasone reduces the syndrome, hydroxyurea controls acute hyperleukocytosis.

**===== run_2 =====**

**Tumor board decision:**
Continuation of induction with ATRA+ATO. Immediate prophylactic dexamethasone therapy (10 mg IV q12h) due to high leukocyte count (65,000/µl) and risk of differentiation syndrome. Additionally hydroxyurea (50mg/kg/d) or low-dose cytarabine (100mg/m2 q12h) up to leukocytes <10,000/µl. Close clinical and hematological control.

**Rationale:**
APL with t(15;17) reacts excellently to ATRA/ATO. The current leukocyte explosion risk requires steroid prophylaxis and rapid leukocyte depletion to prevent differentiation syndrome. Hydroxyurea/cytarabine is standard for WBC>40000/µl. No contraindications available.

**===== run_3 =====**

**Tumor board decision:**
Continuation of induction with ATRA+ATO. Immediate prophylactic dexamethasone therapy (10 mg IV q12h) due to high leukocyte count and risk of differentiation syndrome. Rapid leukocyte reduction with hydroxyurea (50mg/kg p.o. q6h) to <10000/µl. If hyperleukocytosis persists, additional idarubicin 12 mg/m2 on days 1, 3, 5.

**Rationale:**
APL with t(15;17) responds excellently to ATRA/ATO; intermediate risk does not require routine chemotherapy, but leukocytosis increases the risk of differentiation syndrome. Steroid prophylaxis and rapid cytoreduction prevent complications; Idarubicin is only used for persistent hyperleukocytosis.

## ================================================== qwen3-32b (agent) ==================================================

**===== run_1 =====**

**Tumor board decision:**

Addition of hydroxyurea to current ATO/ATRA therapy for cytoreductive support in persistent hyperleukocytosis.

**Rationale:**
For APL-M3 with PML::RARA translocation, the combination of ATO and ATRA is standard. According to flowchart rule APL-3, persistent leukocytosis (>20,000/µl) requires additional measures with hydroxyurea to prevent leukostasis and differentiation syndrome. ATO/ATRA are retained because they enable molecular remission. QTc monitoring and coagulation management remain critical.

**===== run_2 =====**

**Tumor board decision:**
Continuation of ATO/ATRA with additive hydroxyurea cytoreduction for persistent leukocytosis. Closely monitor for differentiation syndrome and QTc prolongation.

**Rationale:**
For APL-M3 with PML::RARA translocation, ATO/ATRA is the gold standard therapy. Persistent leukocytosis (>20,000/µl) requires acute cytoreduction with hydroxyurea. Differentiation syndrome (fever, shortness of breath, pleural effusion) must be excluded. QTc monitoring under ATO is mandatory. No change in basic therapy as intermediate risk and no refractory proven.

**===== run_3 =====**

**Tumor board decision:**
Immediate cytoreduction with hydroxyurea (HU) according to the schedule (>50,000/µl: 2x500mg 4x/day) and close monitoring for differentiation syndrome. Continue ATO/ATRA therapy, consider cytarabine prephase if leukocytosis persists. Check QTc daily under ATO.

**Rationale:**
In APL-M3 with persistent hyperleukocytosis (65,000/µL), urgent cytoreduction is indicated. HU is first choice, cytarabine prephase if response is inadequate. ATO/ATRA remains basic therapy, differentiation syndrome prophylaxis with prednisone is already given. QTc monitoring is mandatory for ATO.

## ================================================== qwen3-32b (plain) ==================================================

**===== run_1 =====**

**Tumor board decision:**
Continue therapy with arsenic trioxide (ATO) and all-trans-retinoic acid (ATRA), supplement differentiation syndrome prophylaxis with dexamethasone. Intensify clinical and laboratory monitoring.

**Rationale:**
AML M3 with t(15;17) requires ATO/ATRA as gold standard. High leukocytes (65,000/µl) increase risk of differentiation syndrome. Dexamethasone minimizes complications. Treatment response has not yet been documented, but the basic regimen remains unchanged.

**===== run_2 =====**

**Tumor board decision:**
Continue therapy with ATRA and ATO, closely monitor for differentiation syndrome and plan consolidation therapy if the leukocyte count persists.

**Rationale:**
AML M3 (PML-RARA+) requires ATRA/ATO as the gold standard. High leukocytes (>65,000/µl) signal risk for differentiation syndrome. Continuation of therapy with close monitoring is crucial. Consolidation at response.

**===== run_3 =====**

**Tumor board decision:**
Continue ATO/ATRA therapy with intensive hematologic monitoring and preparation for possible differentiation syndrome. If leukocytosis persists after 7 days: consider combination with idarubicin (anthracycline basic therapy).

**Rationale:**
AML M3 with t(15;17) requires ATO/ATRA therapy as standard. Current leukocytosis (65,000/µl) requires close monitoring for differentiation syndrome. If the response is inadequate after 7 days,

combination with idarubicin is required as a high-risk profile due to intermediate risk and persistent blast load.

## ================================================== qwen3-next-80b (agent) ==================================================

**===== run_1 =====**

**Tumor board decision:**
Evaluate differentiation syndrome (DS) (fever, dyspnea, pulmonary infiltrates, hypotension). For DS: pause ATO/ATRA, dexamethasone 10 mg iv twice daily, supportive. Without DS: Start hydroxyurea 500 mg 4x daily.

**Rationale:**
According to the APL flowchart: Passage leukocytosis >10,000/µl requires hydroxyurea, DS requires ATO/ATRA break + dexamethasone. WBC 65,000/µl requires evaluation of DS symptoms. Without DS: HU start according to WBC thresholds (50,000+ -> 4x500 mg).

**===== run_2 =====**

**Tumor board decision:**
1. Immediate evaluation for differentiation syndrome (dyspnea, fever, pulmonary infiltrates, hypotension). 2. For DS: pause ATO/ATRA, dexamethasone 10 mg i.v. 2x daily >=3 days. 3. Without DS: Hydroxyurea 2000 mg/day (4x500 mg) for WBC reduction. 4. Continuous WBC and clinical monitoring. Leukapheresis contraindicated.

**Rationale:**
For APL with WBC >50,000/µl, hydroxyurea is indicated (Flowchart APL-3). Leukapheresis prohibited (lead risk). DS clarification prioritized. For DS: dexamethasone and a break from therapy. Sanz intermediate risk does not require chemotherapy add-on, only WBC control.

**===== run_3 =====**

**Tumor board decision:**
Evaluation for differentiation syndrome (dyspnea, hypoxia, pulmonary infiltrates, hypotension). If present: pause ATO/ATRA, dexamethasone 10 mg i.v. 2x daily. Without DS symptoms: Start hydroxyurea 2000 mg/day (4x500 mg), continue ATO/ATRA.

**Rationale:**
According to APL flowchart (APL-3): Hydroxyurea is indicated for leukocytosis >50,000/µl without DS symptoms. If DS symptoms occur, immediate ATO/ATRA break and dexamethasone. Prophylactic prednisone already given (APL-1b), DS clarification prioritized.

## ================================================== qwen3-next-80b (plain) ==================================================

**===== run_1 =====**

**Tumor board decision:**
Addition of idarubicin to ongoing ATRA/ATO therapy. Continuous monitoring for differentiation syndrome with dexamethasone administration if necessary.

**Rationale:**
High leukocyte count (65,000/µl) during ATRA/ATO therapy requires chemotherapy addition to avoid differentiation syndrome. Idarubicin is standard for leukocytic progression, even with initially intermediate risk.

**===== run_2 =====**

**Tumor board decision:**
Continuation of ATRA and ATO therapy as standard therapy for APL M3. No additional chemotherapy required. Close monitoring for differentiation syndrome, with leukocytosis >10,000/µl hydroxyurea to control leukocytes.

**Rationale:**
APL M3 is treated by ATRA+ATO, regardless of Sanz risk. Molecular genetics (PML::RARA+) confirmed diagnosis. Intermediate risk (WBC <=10,000 initial) does not require chemotherapy. Current leukocytosis is typical of differentiation syndrome; Hydroxyurea as needed, not a change in therapy.

**===== run_3 =====**

**Tumor board decision:**
Continuation of ATRA and ATO with addition of hydroxyurea to control leukocytosis

**Rationale:**
ATRA/ATO is standard therapy for APL (Sanz intermediate). Leukocytes >10,000/µL require hydroxyurea to prevent differentiation syndrome risks. No chemotherapy necessary as Sanz risk is intermediate.

# l_case_3__ALL

################################
#### CASE: 3

################################

## ======================================================== gpt-5-mini (agent) ========================================================

**===== run_1 =====**

**Tumor board decision:**
Recommendation: If transplantable, intensive induction with CPX-351 (Vyxeos®) and immediate HLA typing/donor search with planned allogeneic SCT in CR. If the patient is not suitable for intensive therapy (age/comorbidity): azacitidine + venetoclax.

**Rationale:**
Therapy-associated AML/AML-MR with SF3B1 and unfavorable risk profile: CPX-351 is guideline recommendation for AML-pCT/AML-MR and showed better results vs. 7+3. Due to adverse risk, allo-SCT is indicated for consolidation. Lack of fitness -> HMA+Venetoclax as an alternative.

**===== run_2 =====**

**Tumor board decision:**
For patients who are fit in terms of organ function and performance: intensive induction with CPX-351 (Vyxeos®) with immediate HLA typing/donor search and planned allogeneic SCT in CR. For non-intensive fit: Azacitidine +/- Venetoclax.

**Rationale:**
Therapy-associated/AML-MR with SF3B1 and unfavorable risk -> guideline favorite CPX-351 for patients requiring intensive treatment; allo-SCT is curatively indicated. Lack of fitness/high comorbidity: HMA+/-Venetoclax. Clarify age, organ functions and CM histology and quickly start HLA/donor search.

**===== run_3 =====**

**Tumor board decision:**
Recommendation: If sufficient fitness is required, transplantable - carry out intensive induction with CPX-351 (Vyxeos®) and initiate HLA typing/donor search at an early stage with planned allogeneic SCT in CR/CRi. If fitness is limited/comorbidity: low-intensity therapy azacitidine or decitabine +/- venetoclax.

**Rationale:**
AML-pCT (SF3B1, MDS-associated) is considered unfavorable; Guidelines/flowchart recommend CPX-351 for treatment-associated AML in patients requiring intensive treatment. Allo-SZT offers the best curative chance. If fitness is lacking/high comorbidity, HMA+/-Venetoclax is standard. Clarify CM histology and organ functions.

## ======================================================== gpt-5-mini (plain) ========================================================

**===== run_1 =====**

**Tumor board decision:**
Induction with CPX-351 (liposomal cytarabine/daunorubicin) in therapy-related AML with unfavorable risk. Remission: consider prompt allogeneic transplantation. If transplant is not possible: Venetoclax + azacitidine as an alternative.

**Rationale:**
T-AML with poor prognosis; no targetable mutations; CPX-351 Shows OS Benefit in t-AML/AML-MRC; transplantable remission offers curative approach.

**===== run_2 =====**

**Tumor board decision:**
Induction with venetoclax + azacitidine (or decitabine) in t-AML; then allogeneic SCT in remission, if transplantable. If transplantation is not possible, continuation of Venetoclax+AZA or AZA-Mono.

**Rationale:**

T-AML with unfavorable risk profile (SF3B1+, complex karyotype). No FLT3/NPM1/IDH1/2 mutations. Ven+Aza shows activity and serves as a bridge to potentially curative transplantation; Breast cancer history takes into account treatment choice and tolerability.

**===== run_3 =====**

**Tumor board decision:**
Azacitidine + venetoclax as first-line therapy for AML-pCT, 28-day cycle, remission goal; Transplant consultation in remission.

**Rationale:**
Unfavorable risk profile (SF3B1+, MDS-associated, therapy-related AML), no targetable mutations. HMA-venetoclax is the current standard for unfit/therapy-related AML; Remission enables allo-SCT as a potential curative option.

## ==================================================== gpt-5-nano (agent) ====================================================

**===== run_1 =====**

**Tumor board decision:**
Intensive inductive therapy with CPX-351 (Vyxeos) for AML-pCT, followed by allogeneic SCT if transplantable; Start donor search in parallel. If transplantation is not possible, check bridge with HMA-Veneto/cluster detection (e.g. Azacitidine/Decitabine +/- Venetoclax); Consider study options.

**Rationale:**
Therapy-associated AML with unfavorable karyotype (add(13)(q34)/add(11)(p15)) and SF3B1 mutation; CPX-351 Shows Benefit in pCT-AML; potentially curative by allo-SCT; Blasts 25-40% and MDS dysplasia reduce prognosis, hence first-line induction with transplantation as the goal.

**===== run_2 =====**

**Tumor board decision:**
Intensive induction with CPX-351 (Vyxeos) in therapy-related AML/MDS-AML with SF3B1+, unfavorable karyotype, BM blasts 25-40%; followed by allo-SCT if remission and donor availability. If no rapid transplant access, bridge therapy with azacitidine + venetoclax or clinical trial; Continue the donor search in parallel.

**Rationale:**
High-risk of treatment-associated AML with SF3B1+, MDS dysplasia and unfavorable cytogenetics. Goal curative through Allo-SCT. CPX-351 preferred in t-AML/AML-MDS equivalents; no targeted mutation present. If transplantation is delayed, bridging therapy makes sense, study recommendation if necessary.

**===== run_3 =====**

**Tumor board decision:**
Induction with CPX-351 Vyxeos in therapy-related AML/AML-MDS with unfavorable genetics; Target CR/CRi; then allo-SCT after donor matching. If no CR: check clinical trial or VEN/AZA bridge.

**Rationale:**
SF3B1+, MDS dysplasia, unf. karyotype, 25-40% blasts -> high-risk. CPX-351 has benefit in therapy-related AML/MDS; Allo-SCT potentially curative; Start donor matching early; Study option for non-CR.

## ==================================================== gpt-5-nano (plain) ====================================================

**===== run_1 =====**

**Tumor board decision:**
Inductive CPX-351 therapy (liposomal daunorubicin/cytarabine) for treatment-related AML with MDS changes and unfavorable karyotype, if performance is available. If not fit: Azacitidine +/- Venetoclax. Check for CR/CRi allogeneic transplantation; If necessary, consider clinical studies.

**Rationale:**
Unfavorable risk profile (therapy-related AML, MDS-AML, SF3B1+, complex karyotype) with high blasts. CPX-351 improves CR rate/survival vs standard induction and enables transplantation; HMA+/-Venetoclax as a backup option.

**===== run_2 =====**

**Tumor board decision:**
Induction therapy 7+3 (Cytarabine + Daunorubicin/Idarubicin) with goal of remission; after CR/CRi, early allogeneic SCT, if available and tolerable. In case of intolerance/unfitness alternative AZA/DEC + VEN.

**Rationale:**
Therapy-related AML with unfavorable risk profile (SF3B1+, add(13)(q34), add(11)(p15)); no targetable mutations; SCT potentially curative; AZA/DEC+VEN as an option for poor tolerance.

**===== run_3 =====**

**Tumor board decision:**
Initiation of therapy: Intensive AML induction (7+3) with sufficient fitness with the aim of remission and prompt SCT integration. If intolerance or low fitness is present, venetoclax + azacitidine as an alternative; post-remission SCT plan depending on response.

**Rationale:**
AML-pCT, SF3B1-mut, adverse risk profile; NPM1/FLT3/IDH1/2 negative; 25-40% blasts; no target therapy. SCT potentially curative; Prior Breast Cancer Reduces Options; Therapy options depend on fitness and response; rapid remission is desirable.

## ================================================== gpt-oss-20b (agent) ==================================================

**===== run_1 =====**

**Tumor board decision:**
Induction with CPX-351 (Vyxeos®) in therapy-associated AML (SF3B1-positive, post-chemotherapy, unfavorable risk). After CR/CRi, immediate allo-SCT planning; for non-transplant fit, azacitidine+venetoclax as maintenance therapy.

**Rationale:**
Flowchart-B1c has CPX-351 as the induction standard for AML-MR/therapy-associated AML. SF3B1 mutation is not a specific target, but high-risk status and post-chemotherapy history favor CPX-351. In CR/CRi, allo-SCT planning occurs; if not possible, hypomethylating therapy (azacitidine+venetoclax) is recommended.

**===== run_2 =====**

**Tumor board decision:**
Induction 7+3 (Cytarabine 100mg/m2IVq12hDay1-7 + Daunorubicin 60mg/m2IVDay3-5). For CR: 3 cycles of azacitidine 200 mg/m2 IV day 1-5 + venetoclax 400 mgqd day 1-28 (with TLS prophylaxis).

**Rationale:**
AML pCT with SF3B1, no FLT3/NPM1/IDH, therefore standard intensive 7+3 per flowchart. Azacitidine+Venetoclax as effective maintenance of remission in high-risk classes and possible unfit status; supports MRD reduction and extends OS.

**===== run_3 =====**

**Tumor board decision:**
Induction with CPX-351 (Vyxeos®) for therapy-associated AML. After CR/CRi, 2 consolidation cycles CPX-351 or HiDAC, followed by allo-SZT planning (if fit). If not suitable for transplantation, azacitidine+venetoclax as maintenance therapy.

**Rationale:**
AML pCT with SF3B1, unfavorable risk, no FLT3/NPM1/IDH mutations. Flowchart B1c recommends CPX-351 for induction in therapy-associated AML. CPX-351 is also suitable for consolidation; allo-SZT is ideal if suitable. In case of transplant ineligibility, hypomethylating combination as maintenance.

## ================================================== gpt-oss-20b (plain) ==================================================

**===== run_1 =====**

**Tumor board decision:**
Recommendation: Standard induction 7+3 (Cytarabine 100mg/m2 i.v.x7days + Daunorubicin 60mg/m2 i.v.x3days). After remission, evaluate for allo-hematopoietic SCT with appropriate remission and

transplantation approach. For unfitness alternative: Venetoclax + HMA.

**Rationale:**
Secondary AML with SF3B1, no targetable mutations, unfavorable risk. Intensive induction has the highest remission rate; allo-HSCT improves survival in secondary AML. At unfitness Venetoclax+HMA is evidence-based.

**===== run_2 =====**

**Tumor board decision:**
Induction 7+3 (Ara-C 100mg/m2 1-7d, Dauno 60mg/m2 1-3d) + gemtuzumab-ozogamicin 3mg/m2 1d. For CR1: high-dose Ara-C consolidation, if necessary allo-SCT if the condition is suitable.

**Rationale:**
AML CD33+, SF3B1-positive, unfavorable risk; 7+3+GO increases CR rate, GO approved for CD33+. After CR1, high-dose Ara-C + allo-SCT improves survival in treatment-relative AML. No contraindications.

**===== run_3 =====**

**Tumor board decision:**
Start AML induction with 7+3 (Cytarabine 100mg/m2 continuously 1-7days + Daunorubicin 60mg/m2 1-3days). If performance is impaired: low-dose cytarabine or azacitidine. After remission: check allo-SCT.

**Rationale:**
AML pCT, SF3B1 positive, unfavorable risk, no effective alternative markers. 7+3 is standard for fit, offers the best remission rate. Low-dose cytarabine/azacitidine are established for unfit patients. allo-SCT increases survival rate with remission.

## ================================================== gpt-oss-120b (agent) ==================================================

**===== run_1 =====**

**Tumor board decision:**
Recommendation: Azacitidine+Venetoclax as inductive therapy (hypomethylating combination). Intensive chemotherapy only if performance is clearly good and allotransplantation is planned.

**Rationale:**
Therapy-associated AML with SF3B1 mutation has no targeted agent; The unfavorable risk profile and lack of FLT3/IDH mutations speak in favor of the VIALE-A-based HMA-Ven combination, which significantly improves OS and CR rates in patients not suitable for intensive care. Intensive therapy only if a transplant is intended.

**===== run_2 =====**

**Tumor board decision:**
Induction treatment with CPX-351 (liposomal daunorubicin+cytarabine) in a 5-day regimen, followed by MRD control and allo-SCT planning when CR/CRi is achieved.

**Rationale:**
Therapy-associated AML with unfavorable karyotype, no targetable mutations and expected suitability for therapy; CPX-351 is guideline recommended for AML-MR/therapy-associated AML and improves survival. Hypomethylating therapies are reserved for patients not suitable for intensive care.

**===== run_3 =====**

**Tumor board decision:**
Induction treatment with CPX-351 (liposomal daunorubin-cytarabine combination drug) as intensive 1-cycle therapy, followed by Allo-SCT planning in remission, as this is a treatment-associated AML with unfavorable risk and there are no targetable mutations.

**Rationale:**
Therapy-associated AML (AML-MR) is recommended with CPX-351 according to the current AML flowchart; FLT3, IDH and NPM1 are negative, GO is not indicated and the presence of SF3B1 mutation does not provide a targeted approach. Intensive therapy is possible despite previous breast cancer therapy and improves the chances of remission and transplantation.

## ================================================== gpt-oss-120b (plain) ==================================================

===== run_1 =====

**Tumor board decision:**
Initiate CPX-351 induction (3-day daunorubicin+7-day cytarabine) as first-line therapy for therapy-associated AML with myelodysplastic features. If in remission, consider an allogeneic SCT in the first stage of remission. If intensity is not suitable, azacitidine +/- venetoclax.

**Rationale:**
Therapy-associated AML and AML-MRC are considered high-risk; CPX-351 is approved for these subgroups and improves survival compared to 7+3. The patient has early, curative breast cancer, so intensive therapy and, if necessary, transplantation are justifiable. If performance is poor, hypomethylating therapy with venetoclax is the alternative.

===== run_2 =====

**Tumor board decision:**
Initiation of therapy with induction treatment: If physically fit (age <70 years, ECOG0-1) 7+3 (Cytarabine+Anthracycline) followed by consolidation and allogeneic SCT in CR1. In unfit patients, azacitidine+venetoclax as a less intensive induction; then allo-SCT if possible.

**Rationale:**
Therapy-related AML with an unfavorable karyotype and SF3B1 mutation has a high-risk of recurrence; Intensive induction offers the highest chance of remission and transplantation. If fitness is lacking, HMA+Venetoclax is evidence-based and more tolerable. Before starting, carry out a fitness and transplant suitability check.

===== run_3 =====

**Tumor board decision:**
Induction with CPX-351 (5-day regimen) as standard intensive therapy for therapy-associated AML with myelodysplastic features. In case of remission: consolidation with CPX-351 or high-dose cytarabine and evaluation for allogeneic SCT, if suitable for age and comorbidity reasons. For patients not suitable for intensive care: azacitidine+venetoclax.

**Rationale:**
Therapy-associated AML and AML-MRC are considered high-risk; CPX-351 is approved for these subgroups and improves survival compared to 7+3. The patient has no contraindications (breast cancer was pT1b, N0, treated curatively). Allogeneic transplantation provides long-term remission in appropriate patients. If it is not suitable for intensive care, HMA+Venetoclax is an evidence-based alternative.

## ======================================================== qwen3-32b (agent) ========================================================

===== run_1 =====

**Tumor board decision:**
Recommendation: Induction therapy with CPX-351 (liposomal cytarabine + daunorubicin) according to SOP for therapy-associated AML. Subsequently, allo-SCT planning when CR/CRi is achieved.

**Rationale:**
AML-pCT with SF3B1 mutation and unfavorable karyotype (add(13)/add(11)) requires intensive therapy. CPX-351 is the guideline standard for treatment-associated AML. Allo-SCT after remission optimizes prognosis.

===== run_2 =====

**Tumor board decision:**
Recommendation: Azacitidine + venetoclax (non-intensive) as first-line therapy for AML-pCT with SF3B1 mutation and unfavorable risk profile.

**Rationale:**
Therapy-associated AML (AML-pCT) with SF3B1 mutation and complex karyotype (46,XX,add(13)(q34)[2]/46,XX,?add(11)(p15)[6]) have an unfavorable risk profile. CPX-351 is indicated for AML-pCT, but SF3B1 mutations are not primarily targeted for intensive therapies. Non-intensive regimens (azacitidine + venetoclax) are risk-adapted if the risk is unfavorable and there is possible age uncertainty (contrast histology pending). SF3B1 mutations benefit from hypomethylators. Consider allo-SCT planning in parallel.

===== run_3 =====

**Tumor board decision:**
Recommendation: Induction with CPX-351 (liposomal cytarabine + daunorubicin) as standard therapy for AML-pCT with an unfavorable risk profile. Postremission planning: high-dose cytarabine consolidation and early allo-SCT evaluation. SF3B1 mutation not relevant to therapy, but prognostic factor.

**Rationale:**
AML-pCT with SF3B1 mutation and trilinear dysplasia requires the most intensive therapy. CPX-351 is evidence-based for treatment-associated AML. Unfavorable karyotype (add(13)/add(11)) and dysplasia support risk profile. Allo-SCT remains an option post-CR. Venetoclax combinations are not indicated for intensive therapy.

## ==================================================== qwen3-32b (plain) ====================================================

**===== run_1 =====**

**Tumor board decision:**
Recommendation: Induction chemotherapy with azacitidine + venetoklax. If performance status is poor: hypomethylation agent monotherapy.

**Rationale:**
AML-pCT with unfavorable risk profile and SF3B1 mutation. Hypomethylation agents + venetoklax are evidence-based. Previously treated AML requires risk-adjusted therapy. Lack of age: Performance status determines intensity.

**===== run_2 =====**

**Tumor board decision:**
Induction chemotherapy with cytarabine + daunorubicin (7+3 regimen) is recommended. If intolerance, consider azacitidine-based therapy as an alternative.

**Rationale:**
Unfavorable risk profile (complex karyotype, SF3B1 mutation) warrants intensive induction. AML-pCT and trilinear dysplasia suggest myelodysplastic genesis. No contraindications due to breast cancer or comorbidities.

**===== run_3 =====**

**Tumor board decision:**
Induction chemotherapy with cytarabine and daunorubicin (7+3 regimen) is recommended. If the response is inadequate, a second induction with cytarabine + idarubicin is planned.

**Rationale:**
AML-pCT with SF3B1 mutation and unfavorable risk profile requires intensive induction. The blast load in the bone marrow (25-40%) warrants aggressive therapy. The favorable hemoglobin values and normal leukocytes enable standard chemotherapy. Breast cancer in remission is not a contraindication.

## ================================================ qwen3-next-80b (agent) ================================================

**===== run_1 =====**

**Tumor board decision:**
Empfehlung: CPX-351 (Vyxeos®) als first-linentherapie

**Rationale:**
Therapy-associated AML (AML-pCT) according to WHO 2022 with unfavorable risk and MDS-associated mutations (SF3B1) is to be treated with CPX-351 as standard (flowchart INT-1c). 7+3 is not indicated in therapy-related cases. Clinical trials can be considered as a supplement, but CPX-351 is currently the evidence-based first-line option.

**===== run_2 =====**

**Tumor board decision:**
Standard-Induktion mit CPX-351 (Vyxeos®) empfehlen

**Rationale:**
Therapy-associated AML (AML-pCT) according to WHO 2022 with an unfavorable risk is to be treated with CPX-351 as standard (flowchart [INT-1c]). SF3B1 mutation does not change this. Priority: review clinical trials, but CPX-351 is currently evidence-based first-line therapy. No contraindications due

to history of breast cancer (exposed to anthracycline, but CPX-351 dose adjusted).

**===== run_3 =====**

**Tumor board decision:**
Klinische Studie empfehlen

**Rationale:**
Treatment-associated AML (AML-pCT) with unfavorable risk and SF3B1 mutation requires risk-adapted therapy. Standard induction with CPX-351 is established, but due to the unfavorable risk profile and lack of specific targeting options for SF3B1, participation in a clinical trial (e.g. with new combinations or targeted therapies) is prioritized. Flowchart always emphasizes review of clinical trials in AML.

## ====================================================== qwen3-next-80b (plain) ======================================================

**===== run_1 =====**

**Tumor board decision:**
Initiation of intensive induction chemotherapy (7+3: cytarabine 100-200 mg/m2 d1-7 + daunorubicin 60-90 mg/m2 d1-3). Then aim for consolidation and allogeneic SCT (allo-SCT) in first remission.

**Rationale:**
AML-pCT with adverse risk (SF3B1 mutation, cytogenetic abnormalities) requires intensive therapy in fit patients. Previous breast cancer therapy without documented contraindications (e.g. cardiac toxicity). No targeted therapy options (IDH/FLT3 negative). Allo-SCT in CR1 is targeted.

**===== run_2 =====**

**Tumor board decision:**
Initiation of azacitidine in combination with venetoclax as first-line therapy

**Rationale:**
Therapy-associated AML (AML-pCT) with an unfavorable risk profile (karyotypic abnormalities, SF3B1 mutation). Age unknown, but AML-pCT commonly occurs in older patients. Breast cancer history (ED 2023) points to possible comorbidities. HMA + Venetoclax is standard in unfit or elderly patients at unfavorable risk. Intensive chemotherapy (7+3) would be risky if age is not specified.

**===== run_3 =====**

**Tumor board decision:**
Initiate intensive therapy with 7+3 regimen (cytarabine 100-200 mg/m2 d1-7 + daunorubicin 60-90 mg/m2 d1-3).

**Rationale:**
AML-pCT with unfavorable risk (SF3B1, karyotypic aberrations), no previous AML therapy. No indication of age/comorbidities -> assumption fit. Standard therapy for fit patients. Prior breast cancer success (2023) probably caused by t-AML, but anthracycline cumulative dose probably tolerable.

# l_case_4__ALL

###############################
#### CASE: 4
###############################

## ======================================================== gpt-5-mini (agent) ========================================================

**===== run_1 =====**

**Tumor board decision:**
Acute: Continue CNS hard stop - intrathecal triple therapy 2-3x/W until CSF clearance + immediate systemic CNS cytoreduction (HD-MTX or HD-AraC, depending on organ function). Organize immediate leukapheresis (CD19-CAR-T plan) before further lymphocyte-depleting doses. Bridging: Inotuzumab ozogamicin combined with IT/HD chemo for cytoreduction. Curative consolidation: with confirmed CD19 preservation, primarily CD19-CAR-T; if CAR-T is not possible/unsuitable -> Allo-SCT after reaching CR.

**Rationale:**
Massive CSF involvement with neurological deficit requires immediate IT and systemic CNS therapy (flowchart CNS gate). Preservation of CD19/CD22 enables both target strategies: inotuzumab (CD22) rapid cytoreduction; CD19-CAR-T offers a curative chance if the antigen is preserved. Urgent additional: central confirmation of CD19/CD22 (CM+CSF), NGS and classical cytogenetics, HLA typing + donor search, T cell fitness/CD3 count, organ baseline, viral sereologies and MRD markers before definitive choice of therapy.

**===== run_2 =====**

**Tumor board decision:**
Immediately: intrathecal triple therapy 3 times/week until CSF clearance; Continue dexamethasone prephase. Systemic bridging: inotuzumab ozogamicin (CD22+) +/- additional CNS-active therapy (HD-MTX or HD-AraC) for rapid control. Curative consolidation: primary CD19-CAR-T (if available) - urgent leukapheresis before lymphotoxic bridging; if CAR-T is not available/too late: INO-induced CR followed by Allo-SCT. Urgent: Confirmation of CD19/CD22 on CM+CSF, NGS/cytogenetics, Ig/TCR MRD, HLA typing/donor search, organ functions, brain MRI.

**Rationale:**
Massive CSF infestation with neurological symptoms requires immediate i.th. and systemic CNS-directed therapy. Blinatumomab has already been used and did not show a lasting effect -> do not repeat. Preservation of CD22 allows INO as effective bridging; Preservation of CD19 makes CAR-T the preferred curative option. Alternatively, allo-SCT after CR.

**===== run_3 =====**

**Tumor board decision:**
Immediately: continue intrathecal triple therapy 2-3x/W until CSF clearance + systemic prephase (dexamethasone). In parallel, immediate systemic CNS-effective bridging therapy: HD methotrexate +/- HD cytarabine (if organ functions are ok). Alternatively/in addition to rapid cytoreduction: inotuzumab (with careful planning to reduce SCT risk). Parallel: Start CAR-T (CD19) workup including leukapheresis; If CAR-T is not practical: plan Allo-SCT after CR has been achieved.

**Rationale:**
Massive CSF involvement with neurological deficit primarily requires rapid i.th. Clearance + systemic CNS cytoreduction. Persistent CD19+/CD22+ allows CD19-CAR-T as the preferred curative option (also effective in CNS). Blinatumomab earlier response does not exclude CAR-T in the presence of CD19 expression. Urgent additional diagnostics: fresh confirmation of CD19/CD22 on CM and CSF, NGS/cytogenetics, Ig/TCR clone for MRD, HLA typing/donor search, MRI brain/spine, complete organ functions, hepatitis/HIV/CMV serologies, fertility counseling.

## ======================================================== gpt-5-mini (plain) ========================================================

**===== run_1 =====**

**Tumor board decision:**
Systemic salvage chemotherapy (FLAG-IDA) plus intrathecal triple-T to achieve remission and CNS control; immediate donor clarification; Planning an allogeneic SCT in remission/MRD negativity. If

available, use CAR-T (CD19) or inotuzumab ozogamicin as a bridge.

**Rationale:**
Relapse with CNS involvement and 15% blasts in the BM; curative option requires remission followed by transplantation. Previous blinatumomab exposure with possible resistance. Salvage + Transplantation offers a chance of recovery; Bridge options make sense depending on availability and comorbidities.

**===== run_2 =====**

**Tumor board decision:**
Recommendation: CD19-CAR-T cell therapy (e.g. brexucabtagene autoleucel) as primary bridging therapy for relapsed B-ALL with CNS involvement; goal remission; check or carry out allo-HCT after remission; Continue intrathecal therapy.

**Rationale:**
Relapse after GMALL/blinatumomab treatment; CD19 expressed; few effective options remain. CAR-T shows high remission rates including CNS penetration; Transplantation potentially offers long-term cure after remission; CNS lesion requires rapid systemic therapy plus local treatment.

**===== run_3 =====**

**Tumor board decision:**
CAR-T cell therapy (ALL) as a salvage to induce remission, followed by allogeneic SCT if suitable. Continue intrathecal CNS treatment in parallel; CNS-specific measures depending on response (possibly CNS radiotherapy).

**Rationale:**
Relapse after GMALL with 15% contrast blasts and 90% blasts in the CSF; CD22 positive. CAR-T shows assured remission rates in refractory B-ALL; Transplant offers chance of healing; CNS findings require concurrent, ongoing CNS therapy.

## ================================================== gpt-5-nano (agent) ==================================================

**===== run_1 =====**

**Tumor board decision:**
CAR T-cell therapy Brexucabtagene autoleucel (CD19) as primary salvage for relapsed CD19+ B-ALL (Ph-) after GMALL/blinatumomab; continue intrathecal triple therapy; SCT registration early; CR/MRD- -> Allo-SCT; CAR-T failure -> alternative immunotherapy/salvage chemo.

**Rationale:**
CD19+ after blinatumomab; CAR-T provides rapid remission and bridge to transplantation; CNS involvement addressed; Salvage chemo is less curative.

**===== run_2 =====**

**Tumor board decision:**
CAR-T cell therapy with brexucabtagene autoleucel as primary therapy for relapsed, BCR-ABL1-negative common B-ALL after GMALL/blinatumomab. Bridge therapy for cerebrospinal findings; Lymphodepletion before infusion. Target CR/MRD-neg; then allo-SCT, provided the patient is fit and the donor is available; if there is no response, consider alternative CAR-T/salvage chemo.

**Rationale:**
High remission rates with CAR-T in r/r ALL (ZUMA-3/OBE-CEL); CR/MRD-neg after CAR-T facilitates engraftment and long-term control. CNS findings responsive; Blinatumomab history is not an exclusion. Sequence CAR-T -> CR -> allo-SCT is evidence-based; Early transplant planning makes sense.

**===== run_3 =====**

**Tumor board decision:**
CD19-CAR-T (Brexucabtagene autoleucel) as a bridge to allo-SCT, with CNS therapy. If CAR-T is not possible/delayed: salvage chemotherapy or alternative immunotherapy; Target SCT soon after remission.

**Rationale:**
CD19-CAR-T achieves high remissions in r/r ALL and creates transplantation opportunity; CNS involvement requires separate CNS therapy; Backup strategy in place.

## ================================================== gpt-5-nano (plain) ==================================================

**===== run_1 =====**

**Tumor board decision:**
Recommend CD19 CAR T cell therapy (e.g. Tecartus/tisagenlecleucel) as soon as possible. If CAR-T availability delayed/impossible, bridge with inotuzumab ozogamicin (CD22) or other salvage regimen. Goal: MRD-negative remission and subsequent allogeneic transplant; Continue CNS therapy.

**Rationale:**
Relapse after GMALL therapy and blinatumomab; CNS involvement; CD19+ ALL. CAR-T offers a chance of recovery; Transplantation curative. InO bridge useful to achieve rapid remission until CAR-T is available.

**===== run_2 =====**

**Tumor board decision:**
CAR T-cell therapy (CD19) as the next therapeutic step; Bridging therapy with reduced chemotherapy + intrathecal therapeutic measures; prompt allocation to CAR-T center; After remission, allogeneic SCT if necessary (assuming a donor is available).

**Rationale:**
Relapse after GMALL-based protocols, CNS involvement, CD19+ phenotype; Blinatumomab v. pretreatment; CAR-T offers high remission rates including CNS involvement and enables subsequent curative allo-SCT with sufficient remission; CNS symptoms addressable with intrathecal therapy.

**===== run_3 =====**

**Tumor board decision:**
CD19-targeted CAR-T cell therapy as salvage therapy for recurrent B-ALL with CNS involvement; subsequent allogeneic HSCT in remission. Continue parallel intrathecal triple therapy; Dexa prephase as a bridge.

**Rationale:**
RR-B-ALL with CNS infiltration requires potent, center-penetrating therapy. CAR-T has high remission rates and enables transplant-ready states; HSCT offers potential long-term control. Alternatives less effective in CNS relapse.

## ================================================== gpt-oss-20b (agent) ==================================================

**===== run_1 =====**

**Tumor board decision:**
Start CD19 CAR-T (e.g., tisagenlecleucel) with concurrent intrathecal triple therapy and high-dose MTX; if CAR-T not available, give Inotuzumab ozogamicin + intrathecal triple therapy. Aim for remission then allo-SCT.

**Rationale:**
Relapse after blinatumomab, CD20-negative, CNS disease; CAR-T is the most effective CNS-penetrant salvage for CD19+ ALL and can bridge to allo-SCT. Inotuzumab is alternative for CD22+ disease. Intrathecal triple therapy clears CSF. High-dose MTX supports CNS control.

**===== run_2 =====**

**Tumor board decision:**
CD19 CAR T therapy (e.g. tisagenlecleucel) after bridging therapy (intrathecal triple, HD-MTX) as the first salvage option; then allo-SCT for CR.

**Rationale:**
Successful in relapsed B-ALL with CNS involvement, CD19 positive, after blinatumomab failure. Bridging therapy reduces tumor burden, enables CAR-T infusion; allo-SCT in CR for long-term remission.

**===== run_3 =====**

**Tumor board decision:**
Recommendation: Inotuzumab ozogamicin (CD22) in combination with intrathecal triple therapy (MTX, Ara-C, steroid) as the first salvage option. If CR is achieved: allo-SCT. Alternatively, CAR-T (CD19) if CR is missing after 2-3 cycles.

**Rationale:**

Patient has already used Ph-B-ALL, CD22+, blinatumomab, CNS relapse requires intrathecal. Inotuzumab shows high remission rate, can be combined with intrathecal. CAR-T has high CR rates with CNS involvement but time to infusion - hence a second option.

## ================================================= gpt-oss-20b (plain) ==================================================

**===== run_1 =====**

**Tumor board decision:**
Further treatment with bridging therapy (e.g. FLAG-Ida or ICE) followed by CAR-T-cell therapy (tisagenlecleucel) including intrathecal triple therapy. Consider allo-SCT after remission.

**Rationale:**
Patient has a recurrence with CNS involvement after B-ALL induction, blinatumomab and several consolidations. FLAG/ICE offers rapid remission; CAR-T (tisagenlecleucel) has high remission rates in relapses after blinatumomab and can control CNS blasts. Allo-SCT after remission is the best long-term option.

**===== run_2 =====**

**Tumor board decision:**
Inotuzumab ozogamicin plus dexamethasone as salvage therapy; If there is remission, immediately initiate allo-HSCT.

**Rationale:**
Patient is CD22 positive after blinatumomab resistance and CNS relapse; Inotuzumab shows high remission rate in relapsed B-ALL, supported intrathecally. Long-term recovery is only possible via allo-SCT.

**===== run_3 =====**

**Tumor board decision:**
Start salvage therapy with inotuzumab-ozogamicin (30 mg/m2 1x/week) as bridging therapy to achieve remission, followed by an allogeneic SCT in the event of a positive remission. Continue intrathecal triple therapy (methotrexate, dexamethasone, cytarabine).

**Rationale:**
Patient has a bone marrow and CNS relapse after induction, consolidation, blinatumomab and several remission checks. Inotuzumab is the evidence-based salvage option after blinatumomab failure. If remission occurs, allo-HSCT is performed, which offers the best long-term survival rate. Intrathecal therapy remains mandatory due to CNS involvement.

## ================================================= gpt-oss-120b (agent) =================================================

**===== run_1 =====**

**Tumor board decision:**
Recommendation: Start an inotuzumab-ozogamicin-based salvage therapy (4 cycles, dose adjustment according to standard) in combination with continued intrathecal triple therapy until clearance. When a morphological and cytological remission is achieved, allogeneic SCT is carried out immediately, provided a suitable donor is available. Without a suitable donor, CD19-CAR-T (e.g. tisagenlecleucel) should be considered as an alternative.

**Rationale:**
The patient has Ph-negative common-B-ALL, previous blinatumomab failure and multiple chemotherapy failures, as well as CNS involvement. Inotuzumab shows high CR rates after blinatumomab failure and serves as a bridge therapy to allo-SCT, which is the only curative option. CAR-T offers another curative option when a donor is not available.

**===== run_2 =====**

**Tumor board decision:**
We recommend inotuzumab-ozogamicin (4 cycles) combined with intrathecal triple therapy (MTX+Ara-C+steroid) as the first salvage therapy to control the CNS infection, followed by allogeneic SCT when an MRD-negative remission is achieved. If there is no donor or after failed inotuzumab therapy, CD19-CAR-T (e.g. tisagenlecleucel) should be considered as an alternative.

**Rationale:**

The patient has Ph-negative B-ALL, is refractory to multiple GMALL induction and blinatumomab, shows CD22 positivity and CNS involvement. Inotuzumab works independently of CD19, achieves high remission rates in relapse and serves as a bridge therapy to allo-SCT. Intrathecal triple therapy addresses CNS relapse; allo-SCT remains curative, CAR-T is a reserve option.

**===== run_3 =====**

**Tumor board decision:**
We recommend CD19 CAR T cell therapy (e.g. tisagenlecleucel or KTE-X19) as salvage therapy after a short bridging therapy with dexamethasone and intrathecal triple therapy. If complete remission is achieved, allogeneic SCT should be performed if a suitable donor is available.

**Rationale:**
CAR-T achieves 70-80% CR rates even after blinatumomab and in CNS involvement; Inotuzumab carries an increased risk of VOD before transplantation. The patient is transplant eligible, CD19 positive and has CNS infiltration, so CAR-T represents the most effective bridging therapy, which is then consolidated in the long term with allo-SCT.

## ================================================== gpt-oss-120b (plain) ==================================================

**===== run_1 =====**

**Tumor board decision:**
As a next step, we recommend CD19-targeted CAR T cell therapy (e.g. tisagenlecleucel) after lymphocyte depletion, continued with intrathecal triple therapy and systemic dexamethasone prephase. The aim is to achieve an MRD-negative remission, followed by allo-SCT if a suitable donor is available.

**Rationale:**
Relapsed B-ALL with 15% BM blasts and CNS involvement, CD19 positive, blinatumomab therapy already unsuccessful, therefore CAR-T as the most effective option; Inotuzumab would be possible, but CD19 target remains; Allo-HSCT after remission provides long-term control.

**===== run_2 =====**

**Tumor board decision:**
Immediately initiate salvage therapy with inotuzumab ozogamicin (3 cycles) combined with a mini CVD backbone and ongoing intrathecal triple therapy (MTX, cytarabine, hydrocortisone) 3 times/week. Goal: MRD-negative remission, followed by allogeneic SCT. If there is no donor option, CAR-T (tisagenlecleucel) is an alternative.

**Rationale:**
The patient has a hematological and CNS relapse after GMALL and blinatumomab therapy. The recurrence is CD19 and CD22 positive; Repeated use of blinatumomab makes little sense due to possible resistance. Inotuzumab achieves high remission rates in CD22-positive ALL relapses and, together with IT therapy, can address the CNS disease pattern. A subsequent allo-SCT offers the best long-term chance of recovery.

**===== run_3 =====**

**Tumor board decision:**
Salvage therapy with inotuzumab ozogamicin in combination with mini-hyper-CVD (or FLAG-IDA) as bridging therapy, continued intrathecal triple therapy, followed by allogeneic SCT in a suitable donor; If there is no donor, consider CAR T cell therapy (CD19).

**Rationale:**
Relapse after intensive GMALL therapy with 15% BM blasts and CNS involvement. Previous blinatumomab treatment limited efficacy; blasts express CD22, so inotuzumab-ozogamicin is effective. Combination with low-intensity chemotherapy enables remission and bridge therapy to alloHSCT, which is curative in adult ALL relapses. CAR-T as an alternative if there is no donor.

## ================================================== qwen3-32b (agent) ==================================================

**===== run_1 =====**

**Tumor board decision:**

Start with inotuzumab ozogamicin as salvage therapy combined with HD-MTX (1.5 g/m2) and continued intrathecal triple therapy. Evaluate allo-SCT as a bridge to remission.

**Rationale:**
Recurrent common B-ALL with CNS involvement requires targeted salvage (inotuzumab vs. blinatumomab). HD-MTX supports CNS penetration. Allo-SCT remains a long-term goal if there is a response. Weak leg lift requires neurological evaluation.

**===== run_2 =====**

**Tumor board decision:**
Initiate inotuzumab as systemic salvage therapy for recurrent common B-ALL with CNS involvement. Continue intrathecal triple therapy until clearance. Planning allo-SCT after remission induction. Evaluate leg lift weakness for extramedullary involvement (MRI/spinal therapy).

**Rationale:**
Inotuzumab (CD22-targeted) is effective in CD22+ B-ALL after blinatumomab relapse. CNS involvement requires i.th. triple therapy and systemic CNS-active therapy. Allo-SCT remains the goal after remission. Leg lift weakness requires exclusion of spinal compression/extramedullary disease.

**===== run_3 =====**

**Tumor board decision:**
Initiation of salvage therapy with inotuzumab ozogamicin (CD22-targeted) for systemic therapy and continuation of intrathecal triple therapy (MTX/Ara-C/dexamethasone) for CNS involvement. At the same time, the leg lift weakness is clarified (neurology, MRI). If there is a response to inotuzumab, CAR T-cell therapy (CD19-targeted) or allo-SCT should be considered after further remission consolidation.

**Rationale:**
CNS involvement requires prioritized i.th. therapy. Inotuzumab is the salvage option in blinatumomab failure and CD22-positive phenotype. CAR-T/Allo-SCT will be relevant in the future for remission after salvage therapy. Leg lift weakness requires a neurological differential diagnosis (e.g. lesions, compression).

## ====================================================== qwen3-32b (plain) ======================================================

**===== run_1 =====**

**Tumor board decision:**
Recommendation: Salvage therapy with blinatumomab combination therapy (CD19-targeted) and continuation of intrathecal triple therapy. If no response: CAR T-cell therapy (e.g. tisagenlecleucel) or clinical trial.

**Rationale:**
Relapse with persistent blasts (2%) and severe CNS involvement (90% blasts). Blinatumomab has already been used, but MRD negativity remained unconfirmed. Combination with intrathecal therapy addresses systemic and cerebral. In case of resistance: CAR-T cells as the next step.

**===== run_2 =====**

**Tumor board decision:**
Recommendation: Continue systemic blinatumomab therapy in combination with intensive intratecal triple therapy. If the CNS blast load persists after 2 weeks: consider CD19-targeted CAR-T cell therapy.

**Rationale:**
CD19-positive expressing relapsed ALL with CNS relapse has so far responded to blinatumomab (MRD-negative in CM). The high CNS blast load (90%) requires continuous intratecal therapy. Blinatumomab penetrates the blood-brain barrier potential and combines synergistically with local therapy. If CNS progression persists, CAR T-cell therapy (e.g. tisagenlecleucel) would be an alternative.

**===== run_3 =====**

**Tumor board decision:**
Recommendation: Start with intensive salvage therapy combined with blinatumomab and intrathecal triple therapy. If blinatumomab does not work, rapid evaluation for CD19-targeted CAR T-cell therapy.

**Rationale:**
The patient has recurrent common B-ALL with CNS involvement. Blinatumomab has already been used in remission, but current progression suggests resistance. CAR-T cells (e.g. tisagenlecleucel) are established in CD19+ ALL. Intrathecal therapy is mandatory. Salvage chemotherapy alone is inadequate for recurrent disease.

## ================================================== qwen3-next-80b (agent) ==================================================

**===== run_1 =====**

**Tumor board decision:**
Intrathecal triple therapy (MTX/Ara-C/Dexamethasone) 3x/week until CSF clearance. Systemic therapy with CD19-targeted CAR T-cell therapy (e.g. tisagenlecleucel) as salvage, followed by allogeneic SCT in remission. No further blinatumomab administration due to previous treatment failure.

**Rationale:**
CNS involvement (90% CSF blasts) requires immediate intrathecal therapy. Prioritized CAR-T (CD19+) is more effective in CNS resistance than inotuzumab (VOD risk) and blinatumomab (prior treatment failure). Allo-SCT is standard after remission for long-term control. Leg lift weakness requires MRI evaluation for spinal compression.

**===== run_2 =====**

**Tumor board decision:**
Continue intrathecal triple therapy until CSF clearance. Start systemic therapy with inotuzumab ozogamicin (CD22-targeted). If in remission, plan allogeneic SCT (allo-SCT). CAR T-cell therapy as an alternative when inotuzumab is contraindicated or after failure.

**Rationale:**
CNS involvement requires intrathecal therapy until clearance. Blinatumomab (CD19) failed, hence inotuzumab (CD22+) as the next standard. CD19+ allows CAR-T, but inotuzumab can be used more quickly. In case of remission, allo-SCT for curation. Age unknown, but inotuzumab is first salvage option in Ph-B-ALL after blinatumomab failure.

**===== run_3 =====**

**Tumor board decision:**
Continuation of intrathecal triple therapy (MTX/Ara-C/Dexa) until CSF clearance, then CD19-CAR-T cell therapy (e.g. tisagenlecleucel) as systemic salvage therapy. After remission, check allo-SCT.

**Rationale:**
Blinatumomab failure (CD19 positive), CNS involvement (90% CSF blasts). CAR-T effective in CNS involution and after blinatumomab resistance. Inotuzumab with VOD risk, CAR-T preferred. Allo-SCT as a long-term curative option after remission.

## ================================================== qwen3-next-80b (plain) ==================================================

**===== run_1 =====**

**Tumor board decision:**
Initiate CAR-T therapy (e.g., tisagenlecleucel) after stabilization of CNS pathology with intrathecal therapy. Aim for systemic remission before CAR-T.

**Rationale:**
Relapse with CNS involvement after blinatumomab therapy. CAR-T is effective in CD19+ B-ALL, even after prior blinatumomab failure. CNS involvement can be treated with CAR-T. FACS confirms CD19 expression. Aim for allogeneic transplantation after remission.

**===== run_2 =====**

**Tumor board decision:**
Initiate inotuzumab-ozogamicin (0.8 mg/m2 day 1, 0.5 mg/m2 day 8/15) systemically, continue intrathecal triple therapy (MTX/cytarabine/dexamethasone), evaluation for allogeneic SCT.

**Rationale:**
Relapse after blinatumumab with CD22+ expression. Inotuzumab is standard therapeutic option for CD22+ B-ALL. Intrathecal therapy for CNS relapse. Aim for transplantation in remission. No known contraindications.

===== run_3 =====

**Tumor board decision:**
Systemic inotuzumab-ozogamicin (CD22-directed therapy), continue intrathecal triple therapy, evaluate transplant candidate

**Rationale:**
Relapse after blinatumumab with CD22+ expression. Inotuzumab is standard option for relapsed B-ALL after CD19-targeted therapy. Intrathecal therapy necessary for CNS involvement. Transplantation is the goal when remission is achieved.

# l_case_5__ALL

################################
#### CASE: 5
################################

## ==================================================== gpt-5-mini (agent) ====================================================

**===== run_1 =====**

**Tumor board decision:**
Yes. Preemptive therapy recommended. Immediate clarifications: current BM/PB immunophenotyping (CD19, CD22 on blasts), limiting MRD quantification (Ig/TCR + FACS), CSF cytology/FACS, whole body imaging (CNS/extramedullary), CAR-T-PCR copy number and organ status/transplantability. Therapy algorithm: for CD19+: blinatumomab for MRD eradication -> for MRD negativity, allo-SCT evaluation/planning (if transplantable). For CD19-/CD22+: CD22-targeted therapy (inotuzumab or CD22-CAR/study). If there is negative antigen loss on both sides: search for studies/experimental approaches (other CARs, BiTEs). Repeated infusion of the same CAR-T only in studies/individually.

**Rationale:**
MRD increases to Kiel 4x10^-4 or FACS ~0.01-0.04% (>=10^-4 -> high-risk). GMALL/Onkopedia: molecular recurrence -> Blinatumomab preferred for MRD reduction before consolidation/allo-SCT. CD19/CD22 status decides; Inotuzumab has already been given previously (limited), Repeat-CAR-T only on a case-dependent/experimental basis. Antigen status, CNS/extramedullary status and transplant eligibility are mandatory before therapy.

**===== run_2 =====**

**Tumor board decision:**
Yes. Preemptive therapy recommended. First, immediate clarifications: CM immunophenotype with quant. CD19/CD22, MRD confirmation (Kiel + FACS), CAR T-PCR / B cell aplasia, cerebrospinal fluid (CNS), extramedullary imaging, organ status and HLA donor search. Therapy recommendation: for CD19+ -> Blinatumomab MRD-directed; if blinatumomab refractoriness or lack of CAR-T function is documented, consider 2. CAR-T (or CD22-CAR-T if available). For CD19-/CD22+ -> inotuzumab or CD22-directed strategy. Allo-SCT only as consolidation in MRD negativation and transplant fitness.

**Rationale:**
MRD most recently >1x10^-4 and rising slightly - high-risk of recurrence according to GMALL logic. The standard is MRD-directed intensification (blinatumomab). Clarify antigen status and CAR-T functional status before making a decision: repeat CAR-T only if there is persistent CD19 and no CAR-T activity; Allo-SCT only in fit patients after achieving molecular remission.

**===== run_3 =====**

**Tumor board decision:**
Yes. If the MRD increase persists (Kiel 4x10^-4; FACS ~0.01-0.04%), we recommend preemptive blinatumomab (CD19-BiTE) - prerequisite: current detection of CD19 on residual blast cells. For CD19 negativity and CD22+: only inotuzumab if previously effective; otherwise studies/experimental approaches. Allo-SCT only as consolidation after MRD eradication and if the patient is suitable; renewed CAR-T only in individual cases/central setting.

**Rationale:**
According to MRD logic/GMALL, MRD>10^-4 or increasing MRD is an intensification indicator; Blinatumomab is standard treatment for molecular recurrence. Before therapy: current BM immunophenotyping (CD19/CD22), MRD (Ig/TCR), CSF, imaging (extramedullary), PCR quant. CAR-T, organ functions, infection test. Do not routinely repeat already ineffective classes.

## ==================================================== gpt-5-mini (plain) ====================================================

**===== run_1 =====**

**Tumor board decision:**
continue observation; no antileukemic therapy now. MRD monitoring PB/KM every 1-3 months; Check therapy for MRD increase/relapse (e.g. blinatumomab, inotuzumab or CAR-T reinfusion).

**Rationale:**

CR long-standing, MRD near detection limit, CAR-T persistence; no clinical relapse. Therapy risky; Monitoring is a priority; If there is progression/increase in MRD, consider further intervention.

**===== run_2 =====**

**Tumor board decision:**
No ALL therapy now. Close monitoring (KM/MRD Kiel/PB, FACS) every 6-8 weeks; Note CAR-T persistence. If there is an increase in MRD or evidence of blast: Allo-SCT or renewed targeted therapy depending on availability.

**Rationale:**
CR with MRD near borderline; clinically no relapses; CAR-T-PCR positive, no clear indication for further upfront therapy. The risks of further therapy now outweigh the risks; Transplantation/bridging only in case of progression.

**===== run_3 =====**

**Tumor board decision:**
No immediate therapy. Close MRD monitoring (every 4-6 weeks) and clinical-morphological controls. If MRD increases or constant MRD positivity is at risk of relapse, consider early consolidation with allogeneic SCT (CR/MRD negative).

**Rationale:**
The patient has been in morphological CR since CAR-T late 2022; MRD is currently extremely low (FACS ~0.01%; Kiel ~1E-4) and CAR-T cells persist as detected by PCR. No clear indication for immediate further therapy; Transplantation should only be considered in the case of MRD rise/relapse.

## ================================================== gpt-5-nano (agent) ==================================================

**===== run_1 =====**

**Tumor board decision:**
Allo-SCT as consolidation after CAR-T in MRD-positive remission; Bridge with inotuzumab-ozogamicin (CD22+); if necessary, consider blinatumomab; Clarify donor/performance; RED conditioning (e.g., fludarabine-melphalan); DLI post-SCT only for MRD recurrence.

**Rationale:**
MRD-positive status after CAR-T increases relapse risk; SCT can be curative if donor is available and patient is fit. Inotuzumab is a useful MRD bridge; DLI unclear; Planning dependent on donor/performance; Close MRD monitoring recommended.

**===== run_2 =====**

**Tumor board decision:**
Allo-SCT as consolidation after CAR-T in persistent MRD positivity; check donors; MRD-driven bridge therapy for MRD negativation before SCT (e.g. blinatumomab); Conditioning RI-SCT preferred; DLI per MRD course after SCT.

**Rationale:**
Evidence supports MRD-driven bridges before SCT; CAR-T achieves remission, but SCT planning varies; Consider donor availability and comorbidities; Early MRD negativation increases SCT success.

**===== run_3 =====**

**Tumor board decision:**
Allo-SCT recommended as consolidative therapy for persistent MRD after CAR-T despite CR. Donor clarification promptly; Conditioning depending on comorbidities (RIC preferred). Bridge medication for MRD progression (blinatumomab or InO) optional; Consider DLI after SCT if MRD persists or early return of MRD.

**Rationale:**
MRD detection despite CR after CAR-T poses a high-risk of relapse. SCT offers the best possible long-term control. Evidence from similar cases/literature supports SCT after CAR-T; Sequences vary, optimal timing unclear. Donor availability/comorbidities are important, consider DLI after SCT if necessary.

## ================================================== gpt-5-nano (plain) ==================================================

**===== run_1 =====**

**Tumor board decision:**
No acute therapy required. Continuation of observation with close MRD monitoring (PB/KM regularly). If MRD increases or clinical progression occurs, consider repeat MRD-directed therapy (e.g., blinatumomab or repeat CAR-T).

**Rationale:**
CR since CAR-T with persistent but very low MRD values; no progress; Therapy risk/burden higher than benefit. CAR-T persistence delivers control. Further action: observation; Intervention only if there is a significant increase in MRD or symptoms.

**===== run_2 =====**

**Tumor board decision:**
Observation without acute ALL therapy. MRD monitoring regularly (PB/KM, Kiel, FACS) every 1-2 months, then adjust follow-up intervals; clinical control. If MRD increases or relapses: consider allo-SCT; If necessary, re-therapy (blinatumomab or renewed CAR-T) depending on availability and patient condition.

**Rationale:**
CR since CAR-T; currently morphologically CR, MRD positivity (Kiel/PB); CAR-T persistence. No current relapse. Allo-SCT offers curative potential in MRD positivity. Avoid unnecessary toxicity now; close monitoring with preparation for transplantation if MRD increases.

**===== run_3 =====**

**Tumor board decision:**
No acute ALL therapy. Continuation of MRD surveillance (Kiel-MRD, FACS PB/KM, CAR-T-PCR). For MRD increase/relapse: consider blinatumomab or repeat ALL therapy; HSCT only in cases of clear relapse. Continue breast cancer management as planned.

**Rationale:**
CR, MRD near limit, CAR-T persists via PCR; no clinical relapse. Further ALL therapy is currently risky; Monitoring makes sense. Intervene quickly if MRD increases.

## ================================================== gpt-oss-20b (agent) ==================================================

**===== run_1 =====**

**Tumor board decision:**
Observation without additional ALL therapy, MRD monitoring every 3 months and immediate therapy in the event of clinical progression or MRD increase.

**Rationale:**
The patient has been in hematological CR for 19 months, MRD values are between 10■■-10■■, without clinical relapse. Guidelines (MRD logic, GMALL structure) allow observation of persistent MRD <10■■ after CAR-T. Further therapy (blinatumomab/inotuzumab) carries significant risks of side effects and is not absolutely necessary if the CR is stable. Regular MRD checks ensure early intervention in the event of an increase.

**===== run_2 =====**

**Tumor board decision:**
Observation without additional ALL therapy, regular MRD checks (every 4-6 weeks) and clinical monitoring. If increase >0.1% or clinical progression consider immediate reinduction (blinatumomab/inotuzumab).

**Rationale:**
Patient in hematologic CR, persistent very low MRD (<0.01%) after CAR-T; Current guidelines allow observation at such low MRD in order to avoid therapy-toxic risks. If there is a significant increase in MRD or clinical progress, immediate reinduction is indicated.

**===== run_3 =====**

**Tumor board decision:**
observation without additional ALL therapy; Close MRD monitoring (every 3-6 months) and clinical control.

**Rationale:**

Persistent MRD detection after CAR-T in CR, low values (<=10■■), no clinical progression; Literature and comparable cases show that additional blinatumomab/inotuzumab does not significantly increase the remission rate and increase the risk of infections/immunosuppression, especially in the presence of HER2+ breast cancer and already intensive therapy.

## ================================================== gpt-oss-20b (plain) ===================================================

**===== run_1 =====**

**Tumor board decision:**
No further ALL therapy required. Observation with regular MRD control and continuation of HER2 adjuvant therapy (trastuzumab, aromatase inhibitors, radiotherapy).

**Rationale:**
Patient in hematologic CR, MRD at limit of detection, no clinical progression. CAR T cells detectable, no indication for recurrence. Continue breast cancer therapy according to current guidelines.

**===== run_2 =====**

**Tumor board decision:**
No further ALL therapy. Observation with regular MRD control and continuation of HER2-positive breast cancer therapy (trastuzumab/pertuzumab, aromatase inhibitors, radiotherapy).

**Rationale:**
Patient in hematological CR, MRD only at the limit of detection, no clinical evidence of recurrence. After CAR-T therapy, there is no evidence-based indication for further ALL therapies. The focus remains on breast cancer treatment and MRD monitoring.

**===== run_3 =====**

**Tumor board decision:**
No further ALL therapy. Continue hematological observation, possibly light 6-MP/MTX maintenance if necessary. Continuation of adjuvant HER2 therapy (trastuzumab, aromatase inhibitor) and radiotherapy as planned.

**Rationale:**
Patient in hematological CR, MRD only at the limit of detection, no clinical signs of recurrence. CAR T cells detectable, but no indication for therapy. B-ALL therapy would increase side effects without any clear benefit. Focus on surveillance and adjuvant breast cancer treatment.

## ================================================== gpt-oss-120b (agent) ==================================================

**===== run_1 =====**

**Tumor board decision:**
Recommendation: Allogeneic SCT (allo-SCT) with a suitable donor and sufficient organ function. Before the transplant, 4-6 weeks of blinatumomab consolidation for MRD reduction. Without donor/transplant contraindication: Participation in a CD22 or dual-target CAR-T study.

**Rationale:**
Persistent MRD~4x10■■ after CD19-CAR-T greatly increases the risk of recurrence. Studies show that allo-SCT improves long-term survival, while blinatumomab reduces MRD rates. Previous inotuzumab increases the risk of VOD, so use defibrotide prophylactically. A second CAR-T therapy is only recommended in studies and is not excluded by the HER2-positive breast cancer history.

**===== run_2 =====**

**Tumor board decision:**
Allogeneic SCT (allo-HSCT) as definitive consolidation because persistent MRD~4x10■■ after CD19-CAR-T and high-risk of recurrence; In the absence of donor availability or contraindication, consider MRD-targeted therapy with blinatumomab (if CD19 expression) or CD22-based approach (inotuzumab or CD22-CAR-T).

**Rationale:**
Persistent MRD after CAR-T is associated with short survival; Guidelines recommend allo-SCT in MRD-positive adults if fit. Blinatumomab can reduce MRD but is limited by possible CD19 loss. CD22 therapies have already been used, note cumulative toxicity. HSCT offers the best long-term control

and is compatible with HER2-positive breast cancer.

**===== run_3 =====**

**Tumor board decision:**
Start MRD-targeted therapy with blinatumomab (28-day infusion, 2 cycles if necessary). After completion of MRD control; If MRD positivity persists or there is a high-risk of recurrence, consider allogeneic SCT (if suitable donor and fitness). Alternatively, participation in a CD22 or CD19/CD22 dual CAR-T study can be offered.

**Rationale:**
Persistent MRD~4x10■■ after CD19-CAR-T is significantly reduced by blinatumomab in phase 3 data and improves DFS. The patient only had a short exposure to blinatumomab, so it makes sense to use it again. Allogeneic HSCT offers a curative option when there is a high-risk of recurrence, but is only indicated if there is sufficient conditioning. CD22-CAR-T is an experimental rescue option for study patients.

## ==================================================== gpt-oss-120b (plain) ====================================================

**===== run_1 =====**

**Tumor board decision:**
There is currently no indication for therapy for B-ALL. The patient is in hematological CR, the persistent MRD signal is in the borderline range (PCR detection of CAR T cells, slight flow positivity). Recommendation: close MRD controls and clinical observation; If there is a significant increase in MRD or clinical recurrence, immediate salvage therapy (e.g. blinatumomab or renewed CAR-T).

**Rationale:**
After multiple remissions (GMALL-Elderly, Inotuzumab, CD19-CAR-T) and sustained CR, the low MRD signal is likely residual CAR-T cells, not active malignancy. Further therapy creates unnecessary toxicity, while close monitoring allows early intervention in the event of true progression.

**===== run_2 =====**

**Tumor board decision:**
There is currently no indication for therapy. The patient is in hematological CR, the persistent but low MRD (<=4x10■■) is explained by persistent CAR T cells. Recommendation: close clinical and molecular monitoring (e.g. q-3 months) and continuation of breast cancer therapy; Therapy only in the event of clinical relapse or MRD>10■3.

**Rationale:**
After CAR-T therapy, a low, slowly increasing MRD is often and alone is not a criterion for intervention. The patient is clinically remitted, has no therapy contraindications and is already receiving adjuvant breast cancer therapy. Intervening would cause unnecessary toxicity.

**===== run_3 =====**

**Tumor board decision:**
No immediate indication for therapy. Further close monitoring of the MRD (PCR, FACS) and the clinical situation; Continuation of adjuvant HER2-targeted therapy and local radiation. If MRD increases or clinical recurrence occurs, consider allogeneic SCT.

**Rationale:**
Patient is in hematological CR after CD19-CAR-T, only persistent very low MRD evidence without clinical activity. Previous therapies (inotuzumab, CAR-T) have been exhausted, Allo-SCT carries a high-risk if the age is unknown and breast cancer is already present. Observation is appropriate according to guidelines.

## ==================================================== qwen3-32b (agent) ====================================================

**===== run_1 =====**

**Tumor board decision:**
No therapeutic intervention is indicated. MRD positivity is at the limit of detection, CAR-T cells are detectable by PCR and FACS-MRD is negative. Clinical CR exists without blast proliferation.

**Rationale:**

The MRD dynamics (1E-4-8E-5) lie in the limit of detection. CAR T cells are still active (detectable by PCR), suggesting biological control of the disease. FACS-MRD is negative and there is no evidence of clinical progression. Therapeutic interventions at such low MRD burdens without clinical relevance are not evidence-based.

**===== run_2 =====**

**Tumor board decision:**
No therapeutic intervention for MRD positivity in hematological CR after CAR T cell therapy with persistent CAR T cell presence (PCR detectable) and borderline positive MRD dynamics (Kiel/FACS).

**Rationale:**
MRD dynamics are at the limit of detection (1E-4-4E-4), while CAR-T cells are detectable by PCR and FACS-MRD remains negative. No clinical progression, no cytopenias or extramedullary involvement. CAR-T cell therapy and GMALL protocol indicate no indication for further intervention. MRD positivity alone does not justify therapy in the absence of molecular or cytological progression.

**===== run_3 =====**

**Tumor board decision:**
Recommendation: Therapy intervention not indicated for the time being. MRD positivity remains below the detection limit, CAR-T cells are persistently detectable. Strict MRD surveillance (Kiel/FACS) and clinical observation. If MRD dynamics increase or clinical progression occurs, consider blinatumomab as initial intervention.

**Rationale:**
Current MRD dynamics are at the limit of detection with stable CAR T cells, suggesting controlled disease. Literature and similar cases confirm that if MRD does not increase and there is no clinical progression, immediate intervention is not required. Blinatumomab remains a reserve option in case of future MRD increase. Contraindications due to breast cancer therapy are taken into account.

## ======================================================= qwen3-32b (plain) =======================================================

**===== run_1 =====**

**Tumor board decision:**
No immediate indication for therapy. Continue MRD surveillance and monitor CAR T cell activity. In case of clinical progression or increase in MRD, consider therapy with blinatumomab or alternatively CD19/CD22-targeted therapy in combination with immuno-oncologic agents.

**Rationale:**
The patient is in hematologic CR with MRD at the limit of detection. CAR-T cells can be detected by PCR, suggesting a latent control. Aggressive therapy is not indicated unless there is clinical progression. MRD surveillance and observation remain primary. If there is an increase, alternatively evaluate blinatumomab or new targeted therapies.

**===== run_2 =====**

**Tumor board decision:**
No immediate indication for therapy. Controlled MRD monitoring with FACS and Kiel method. For clinical progression or confirmed MRD increase, consider both methods: targeted therapy (e.g. CD19/CD22-targeted approaches) or clinical trials.

**Rationale:**
The patient shows hematological CR with divergent MRD results (Kiel positive, FACS negative). CAR-T cells can be detected by PCR, which suggests control of the disease. Therapy is omitted due to the borderline MRD and lack of clinical progression. Neoadjuvant breast cancer treatment is completed.

**===== run_3 =====**

**Tumor board decision:**
No active indication for therapy despite MRD positivity. Continue hematologic monitoring with MRD studies every 3-6 months.

**Rationale:**
The patient is in hematologic CR with minimal MRD (0.001-0.008%) and PCR detectable CAR-T cells. No clinical progression or toxicities. CAR-T history and low MRD burden suggest stable remission. No new target structures or study paths. Breast cancer is well controlled postoperatively.

## ================================================== qwen3-next-80b (agent) ==================================================

**===== run_1 =====**

**Tumor board decision:**
Keine therapeutische Intervention indiziert. Kontinuierliche MRD-Monitoring (alle 1-2 Monate) und klinische Beobachtung.

**Rationale:**
Persistent low MRD (<=0.01%) in hematological CR after CAR-T with detectable CAR-T cells is often worth monitoring if the disease is stable. Antigen escape (FACS negative, Kiel positive) makes CD19/CD22-targeted therapies unlikely to be effective. Re-challenge with inotuzumab/blinatumomab contraindicated (prior efficacy). Dual-target CAR-T only makes sense in cases of MRD progression. Current data show stable remission without risk of progression.

**===== run_2 =====**

**Tumor board decision:**
Therapeutic intervention with blinatumomab indicated. Start of blinatumomab therapy according to FDA approval for MRD-positive B-ALL, also after CAR-T.

**Rationale:**
Persistent MRD positivity (Kiel MRD PB 4E-4) after CAR-T requires intervention as MRD+ is a risk factor for relapse. Blinatumomab is standard therapy for MRD+ B-ALL, even after CAR-T, and FDA-approved. Previous exposure to blinatumomab is not a contraindication as the indication currently exists and the therapy was effective in comparable cases. No relevant contraindications due to breast cancer therapy.

**===== run_3 =====**

**Tumor board decision:**
Continue to monitor with monthly MRD and blood count checks. No therapeutic intervention indicated.

**Rationale:**
Persistent MRD positivity under CAR T cell presence in hematologic CR is stable (no increase). Allo-SCT is contraindicated in older patients (GMALL-Elderly protocol) and prioritized comorbidity (breast cancer). Experimental therapies would introduce additional toxicity without clear benefit. According to flowchart V-4 in cytological CR without molecular CR: in stable MRD and CAR-T presence, observation is preferred.

## ================================================== qwen3-next-80b (plain) ==================================================

**===== run_1 =====**

**Tumor board decision:**
No current indication for therapy. Continuous MRD monitoring (monthly) and clinical observation.

**Rationale:**
Hematological CR with MRD at the limit of detection (0.004%) and stable CAR-T cells. MRD values below critical threshold (0.01%), no clinical progression. Intensification with further therapy is not indicated in the current situation due to increased risks of toxicity without clear benefit.

**===== run_2 =====**

**Tumor board decision:**
No current indication for therapy. Continuous MRD monitoring (every 1-2 months) and clinical observation. No systemic therapy in existing hematological CR and low-grade MRD.

**Rationale:**
MRD detection (max. 0.04%) is below critical threshold (0.1%), patient in CR with detectable CAR-T cells. No morphological signs of recurrence. Current breast cancer therapy (trastuzumab/aromatase inhibitor) is ongoing; additional therapy increases risk of toxicity. Monitoring is evidence-based and appropriate.

**===== run_3 =====**

**Tumor board decision:**
Keine therapeutische Intervention erforderlich. Kontinuierliche MRD-Monitoring (alle 1-3 Monate) und klinische Beobachtung.

**Rationale:**
Hematologic CR with persistent but very low MRD (<=0.01%) and detectable CAR-T cells. FACS-MRD negative. No morphological signs of recurrence. Therapeutic intervention not indicated for stable, minimal MRD values after CAR-T. Risk of toxicity outweighs benefit. Current breast cancer requires focused therapy that should not be interfered with by additional ALL therapies.

# l_case_6__ALL

################################
#### CASE: 6

################################

## ==================================================== gpt-5-mini (agent) ====================================================

**===== run_1 =====**

**Tumor board decision:**
Primary: inclusion in a clinical trial with menin inhibitor (e.g. revumenib) as the preferred option for remission induction and bridge-to-2. allo-SCT. If not available or patient is not eligible for inclusion: intensive salvage chemotherapy (e.g. FLAG-IDA or HAM) without venetoclax with the aim of 2nd allo-SCT. Palliative only in unfit patients.

**Rationale:**
KMT2A rearrangement shows promise for menin inhibitors; Venetoclax/azacitidine was ineffective and should not be repeated. In fit patients, the curative strategy is to achieve remission through intensive salvage followed by second SCT. MEK inhibitors/other options currently experimental-trials only.

**===== run_2 =====**

**Tumor board decision:**
Primary: rapid inclusion in a clinical trial with a menin inhibitor (e.g. revumenib) because of persistent KMT2A rearrangement. If the study is not accessible or if the tumor burden is immediately high: cytoreductive intensive salvage chemotherapy (e.g. FLAG-IDA or HAM) +/- venetoclax as a bridge to allogeneic SCT. Palliative for non-transplant candidacy.

**Rationale:**
For KMT2A-rearranged R/R-AML, the strongest option is currently menin inhibition (clinical remissions reported). Patient relapsed after venetoclax/azacitidine -> if there is no access to the study, intensive salvage regimens +/- venetoclax make sense as an evidence-based bridge to the 2nd allo-SCT. KRAS-targeted MEK inhibitors remain experimental.

**===== run_3 =====**

**Tumor board decision:**
Primary: Inclusion in a clinical trial with a menin inhibitor (e.g. revumenib) preferred. If trial cannot be achieved or there is a need for rapid cytoreduction: intensive salvage chemotherapy (e.g. FLAG-IDA or HAM) if necessary + venetoclax as a bridge to rapid 2nd allo-SCT if transplantable. MEK inhibitor only in study; palliative if neither trial nor transplant is possible.

**Rationale:**
Persistent KMT2A rearrangement makes menin inhibition the best targeted option. The patient relapsed after venetoclax/azacitidine (therapy failure), therefore no further VEN monotherapy alone. Intensive salvage may enable remission for allo-SCT. KRAS mutation justifies MEK inhibitor only experimentally.

## ==================================================== gpt-5-mini (plain) ====================================================

**===== run_1 =====**

**Tumor board decision:**
FLAG-IDA reinduction as salvage therapy to achieve CR/MRD negativity followed by allogeneic SCT; if there are contraindications, MEC as an alternative; examine clinical studies in parallel (KMT2A/menin inhibitors).

**Rationale:**
KMT2A-Rearranged AML has poor prognosis in relapse. Rapid, profound remission is necessary to make SCT possible. FLAG-IDA offers good remission rates; SCT potentially curative. Trials make sense due to new targeting approaches.

**===== run_2 =====**

**Tumor board decision:**

Salvage induction (FLAG-IDA or CLAG-M) targeting CR/MRD negative, followed by allogeneic HSCT if transplantable; Parallel review of clinical trials (menin inhibitors) in KMT2A rearrangement.

**Rationale:**
KMT2A-rearr AML relapses after VEN/AZA; so far no permanent remission. Allogeneic transplantation offers the best long-term perspective. Examination of clinical trials makes sense because new target therapies (menin inhibitors) potentially open up additional options. CD33+ risk protein supporting GO as a reserve.

**===== run_3 =====**

**Tumor board decision:**
Recommendation: Clinical trial with menin inhibitor or DOT1L inhibitor in KMT2A-rearranged AML. If not available, salvage induction chemotherapy (FLAG-IDA/CLAG-IDA) for remission and subsequent allotransplantation; in case of non-transplantability, palliative/trial targeting.

**Rationale:**
KMT2A-rearranged AML with relapse after VEN-AZA; prognostically unfavorable. The goal is remission and transplantation, trials provide molecular targeting in this entity; Default options limited.

## ======================================================== gpt-5-nano (agent) ========================================================

**===== run_1 =====**

**Tumor board decision:**
Bridge with menin inhibitors KO-539 or SNDX-5613 in clinical trial as a bridge to achieve MRD-negative remission and then aim for allogeneic transplantation. If no trial available, VEN-HMA salvage; MRD and chimerism monitoring closely.

**Rationale:**
Menin inhibitors enable remissions and transplantation as a curative goal; Study priority, bridging makes sense, MRD/chimerism central.

**===== run_2 =====**

**Tumor board decision:**
Allo-transplantation in first remission remains the goal. Bridge to MRD Negativity; preferred menin inhibitor trial (KO-539/SNDX-5613) or VEN/HMA as a bridge.

**Rationale:**
KMT2A-rearranged R/R AML relapsed after VEN/AZA; Transplant offers a chance of healing. Menin inhibitors active as a bridge; no RCT. Priority access to studies; Transplantation after remission.

**===== run_3 =====**

**Tumor board decision:**
Menin inhibitor bridge therapy in clinical trial (SNDX-5613/KO-539) for MRD-negative remission followed by allogeneic transplantation; If no study access, VEN+FLAG-IDA as a bridge; Consider transplantation only after remission/MRD negativity.

**Rationale:**
KMT2A-rearranged AML in RR after VEN/AZA; Allo-SCT offers the highest chance of cure. Menin inhibitors show activity as a transplant bridge; The goal is MRD negativity before transplant. If there is no access to study, VEN+FLAG-IDA as an alternative. Pay attention to CAR-T limitations.

## ======================================================== gpt-5-nano (plain) ========================================================

**===== run_1 =====**

**Tumor board decision:**
Recommendation: Enrollment in a clinical trial with menin inhibitors (e.g. SNDX-5613 or KO-539) in KMT2A-rearranged AML; alternatively salvage chemotherapy (FLAG-IDA/CLAG-M) to achieve remission; subsequent allogeneic SCT, if possible.

**Rationale:**
Relapse after VEN/AZA in KMT2A-AML with poor prognosis. Menin inhibitors show activity in KMT2A rearrangements; The aim is MRD-negative remission and subsequent transplantation as a curative option; Check transplant eligibility and consider risks.

**===== run_2 =====**

**Tumor board decision:**
Salvage induction with FLAG-IDA or MEC followed by allogeneic SCT if there is remission or sufficient transplant fitness; Parallel testing of clinical trials (e.g. menin inhibitors) in KMT2A rearrangement.

**Rationale:**
Relapse after VEN/AZA; KMT2A rearrangement, KRAS+, FLT3 neg.; current blast ~30%; MRD-negative CR 04/2023; Aim: MRD-negative remission and transplantation; intensive salvage makes sense; Trials make sense due to poor prognosis.

**===== run_3 =====**

**Tumor board decision:**
Salvage chemotherapy for remission/MRD negativity followed by allogeneic SCT (allo-SCT) as the standard goal. Appropriate regimens: FLAG-IDA, CLAG-M or CPX-351; depending on tolerability. If transplant is not possible in a timely manner or is delayed, participation in a clinical study with menin-MLL inhibitors.

**Rationale:**
High-risk MLL-rearranged AML (KMT2A) relapsed after VEN/AZA; so far short remissions. Allo-SCT offers the best chance of cure. The goal is MRD-negative CR before transplantation. Salvage regimen is intended to enable remission; clinical trials or alternative therapies are considered if transplant cannot be implemented in a timely manner.

## ======================================================= gpt-oss-20b (agent) =======================================================

**===== run_1 =====**

**Tumor board decision:**
Since persistent MRD after venetoclax/azacitidine and KMT2A rearrangement, the highest priority is allogeneic SCT (allo-SCT) if suitable donor availability is available. If there is no suitable donor or the risk is too high, participation in a clinical trial with revumenib (menin inhibitor) is recommended; alternatively Venetoclax+FLAG-IDA as a salvage option.

**Rationale:**
Patient has already undergone standard salvage therapies without MRD success. Literature (5 PubMed articles) identifies revumenib as first choice in KMT2A-AML; allo-SCT remains the only curative option. Similar cases (1 case) showed a second allo-SCT or targeted therapy in the absence of remission.

**===== run_2 =====**

**Tumor board decision:**
Start revumenib (KMT2A inhibitor) as salvage therapy. In case of remission (CR/CRi), immediately allo-SCT. If no remission or transplant risk, consider enrollment in CD123 CAR T study (e.g. NCT05412345).

**Rationale:**
Patient has KMT2A rearrangement and persistent MRD after venetoclax/azacitidine. Revumenib (NCT04065399) shows CR 55-60% in KMT2A-AML and is the preferred option after venetoclax-azacitidine. In case of remission, allo-SCT remains the standard. CD123-CAR-T is an ongoing study in KMT2A-AML and offers an alternative if revumenib does not work.

**===== run_3 =====**

**Tumor board decision:**
Intensive salvage with FLAG-IDA + venetoclax (off-label) as bridging therapy, parallel HLA search for allo-SCT. Alternatively, participation in a clinical trial with a menin inhibitor (e.g. revumenib) for KMT2A fusion. Goal: remission and allo-transplantation.

**Rationale:**
Patient shows persistent MRD despite venetoclax/azacitidine; KMT2A rearrangement has poor prognosis. FLAG-IDA + venetoclax has good remission rates in R/R AML and can serve as a bridge to allo-SCT. Menin inhibitors are specific for KMT2A fusion and are active in studies.

## ================================================== gpt-oss-20b (plain) ==================================================

**===== run_1 =====**

**Tumor board decision:**
Forwarding to allogeneic stem cell transplantation after bridging therapy (e.g. FLAG-IDA or CLAG-M).

**Rationale:**
Patient has AML relapse with KMT2A rearrangement, CEBPA-, KRAS+, FLT3-/NPM1-negative; already venetoclax/azacitidine unsuccessful. Allogeneic transplantation is the only curative option; Bridging regimens enable remission until transplantation.

**===== run_2 =====**

**Tumor board decision:**
Referral to allogeneic stem cell transplantation (allo-SCT) after minimal bridge therapy (e.g. low-dose cytarabine or decitabine).

**Rationale:**
AML with KMT2A rearrangement, CEBPA- and KRAS+ is refractory to 3 courses of venetoclax/azacitidine. No FLT3/IDH mutations, therefore no targeted therapy. Allo-SCT offers the only potentially curative option; Bridge therapy reduces tumor burden and improves transplant outcomes.

**===== run_3 =====**

**Tumor board decision:**
Further treatment with an allogeneic SCT (allo-SCT) as an initial option; If there is no suitable transplant option, use a salvage regimen (e.g. FLAG-Ida) or participate in a clinical trial.

**Rationale:**
AML with KMT2A rearrangement, CEBPA-, KRAS+ is aggressive; after failure of HMA/venetoclax, allo-SCT remains the only potentially curative option. In cases of transplant inability, FLAG-Ida is the next best salvage regimen.

## ================================================== gpt-oss-120b (agent) ==================================================

**===== run_1 =====**

**Tumor board decision:**
Salvage therapy with venetoclax+intensive FLAG-IDA (or comparable intensive AML regimens) for rapid MRD-negative remission, followed by allogeneic SCT. If a revumenib trial is available, a menin inhibitor-based concept should be preferred as a bridging therapy.

**Rationale:**
The patient has a KMT2A rearrangement (targeted menin inhibitor) and showed a short remission after venetoclax/azacitidine, so an intensive venetoclax-FLAG-IDA approach as a bridge to allo-SCT is evidence-based. Literature and a similar case support both options; Revumenib offers a targeted, potentially less toxic alternative if available for trial.

**===== run_2 =====**

**Tumor board decision:**
Implementation of an intensive venetoclax-based salvage regimen (e.g. venetoclax+FLAG-IDA or venetoclax+HAM) aiming at MRD-negative remission, followed by allogeneic SCT as soon as possible. If available, therapy in the revumenib (menin inhibitor) study protocol should also be considered.

**Rationale:**
The patient has a KMT2A rearrangement, KRAS mutations and failure of the venetoclax/azacitidine combination. Current literature and similar cases show that intensive venetoclax-based combinations (e.g. FLAG-IDA) achieve high remission rates and serve as a bridge to allogeneic SCT. Menin inhibitors (Revumenib) are targeted for KMT2A-AML and can be added as part of a study.

**===== run_3 =====**

**Tumor board decision:**
It is recommended that the patient be included in a menin inhibitor study arm (e.g. revumenib) or compassionate use. If this is not possible, intensive venetoclax-combined salvage therapy (e.g. FLAG-IDA+venetoclax) should be carried out as a bridge therapy up to allogeneic SCT.

**Rationale:**
KMT2A rearrangements respond strongly to menin inhibitors (Phase I/II data show high CR rates). After failure of Ven/azacitidine, intensive venetoclax chemotherapy is still effective and allows remission before allo-SCT. Similar cases use Venetoclax-Intensive-Salvage+allo-SCT.

## ================================================== gpt-oss-120b (plain) ==================================================

**===== run_1 =====**

**Tumor board decision:**
Start salvage therapy with the menin inhibitor revumenib (or participate in a corresponding clinical trial) and, if there is a response, immediately seek an allogeneic SCT.

**Rationale:**
The patient has an AML relapse with persistent KMT2A rearrangement after HMA+venetoclax. Revumenib is approved for relapsed/refractory KMT2A AML and demonstrates high response rates. Successful remission allows the only curative option - allogeneic HSCT.

**===== run_2 =====**

**Tumor board decision:**
Initiation of intensive salvage therapy (e.g. FLAG-IDA or CPX-351) to achieve a second remission, followed by allogeneic SCT if a suitable donor is available. Parallel evaluation of a clinical study with a menin inhibitor (revumenib) due to KMT2A rearrangement.

**Rationale:**
AML is refractory to venetoclax/azacitidine, KMT2A rearranged and CD33 positive - a very poor prognostic profile. A curative option requires remission and allo-HSCT. Intensive salvage regimen increases the remission rate; Menin inhibitors show promising activity in KMT2A-AML.

**===== run_3 =====**

**Tumor board decision:**
An intensive salvage regimen (e.g. CPX-351 or FLAG-IDA) to achieve an MRD-negative second remission, followed by allogeneic SCT, should be initiated as the next step. If a suitable donor is available, the transplant must be planned promptly; alternatively, a clinical trial with menin inhibitor (e.g. revumenib) for KMT2A-rearranged AML should be considered.

**Rationale:**
After line switching to KMT2A-AML, the patient had already achieved a short remission with venetoclax/azacitidine, but quickly experienced a recurrence - evidence of therapy resistance. KMT2A rearrangements are considered high-risk and hypomethylation therapy alone is not curative. Intensive re-induction to achieve deep remission followed by allogeneic HSCT offers the best chance of long-term survival. Menin inhibitors show promising activity in KMT2A-AML and should be used in a trial if available.

## ================================================== qwen3-32b (agent) ==================================================

**===== run_1 =====**

**Tumor board decision:**
Recommendation: Revumenib (menin inhibitor) as first line in recurrent AML with KMT2A rearrangement. If remission occurs, plan allo-SCT. Consider FLAG-IDA + Venetoclax as a more intensive option. CAR T cells as a last option.

**Rationale:**
KMT2A rearrangement is predictive of menin inhibitors such as revumenib. Venetoclax/azacitidine showed partial response but recurrence. Allo-SCT after remission is standard. FLAG-IDA + Venetoclax as a more intensive salvage option. CAR T cells when determining therapy.

**===== run_2 =====**

**Tumor board decision:**
Recommendation: Revumenib as first salvage therapy for recurrent AML with KMT2A rearrangement. If in remission, plan allo-SCT. Alternatively, check FLAG-IDA + venetoclax. CAR T cells (CD123 targeting) as a last option.

**Rationale:**

Based on evidence, revumenib (menin inhibitor) is the strongest targeted therapy for KMT2A rearrangement. Venetoclax/azacitidine showed only partial remission. Allo-SCT after remission is standard. CAR T cells as an experimental option in the event of treatment failure.

**===== run_3 =====**

**Tumor board decision:**
Recommendation: Revumenib (menin inhibitor) as the first salvage option for recurrent AML with KMT2A rearrangement. If in remission, evaluate allo-SCT. FLAG-IDA + Venetoclax as an intensive salvage alternative with good performance. Apply CD123 CAR T cell clinical trials.

**Rationale:**
Revumenib is evidence-based relevant to KMT2A rearrangements. Previous venetoclax/azacitidine treatment was only temporarily effective. FLAG-IDA + Venetoclax offers intensive salvage option. Allo-SCT after remission is standard. CAR-T trials address specific targets in relapsed AML.

## ================================================== qwen3-32b (plain) ==================================================

**===== run_1 =====**

**Tumor board decision:**
Recommendation: Participation in a clinical trial with combination therapy (e.g. KRAS-targeted) or intensive salvage chemotherapy (e.g. 7+3) with Allo-SCT preparation, if the patient is fit.

**Rationale:**
Recurrence after venetoclax/Aza and KMT2A rearrangement with poor prognosis. KRAS mutation and prior treatments limit options. Clinical trials or allo-SCT if appropriate are given priority.

**===== run_2 =====**

**Tumor board decision:**
Recommendation: Gemtuzumab ozogamicin (CD33-targeting) in combination with azacitidine or decitabine as the next therapeutic step. If responsive: evaluation for Allo-SCT.

**Rationale:**
The patient has AML with KMT2A rearrangement and CD33+ blasts after relapsed B-ALL. Gemtuzumab ozogamicin is evidence-based for CD33+ AML. Previous venetoclax/azacitidine showed partial response but relapse. Combination with hypomethylators could have a synergistic effect. Allo-SCT remains a long-term goal in remission.

**===== run_3 =====**

**Tumor board decision:**
Recommendation: Allogeneic SCT (SCT) after intensive conditioning therapy. In non-transplantable condition: Clinical trial with combination therapy (e.g. venetoclax + IDH inhibitor or FLT3 inhibitor) taking into account the KMT2A rearrangement and KRAS mutation.

**Rationale:**
The patient presents with relapsed AML with KMT2A rearrangement and KRAS mutation after line switch. Previous therapies (blinatumomab, venetoclax/azacitidine) showed only partial responses. Allogeneic SCT remains the gold standard for recurrent genomic aberrations. If non-transplantable, targeted combinations based on molecular profiles are a priority.

## ================================================ qwen3-next-80b (agent) ================================================

**===== run_1 =====**

**Tumor board decision:**
Menin inhibitor (e.g. revumenib) in clinical trial or off-label, followed by allo-SCT in remission. No intensive salvage therapy due to lack of tolerance and lack of FLT3 mutation. Priority: remission induction for transplantation.

**Rationale:**
KMT2A rearrangement is high-risk. Studies of menin inhibitors show high response rates in this subtype. Venetoclax/azacitidine failed after initial remission. Intensive salvage (FLAG-Ida) is high-risk in AHT and unknown age. Allo-SCT is the only curative option but requires remission. Menin inhibitor is targeted bridge to transplantation.

**===== run_2 =====**

**Tumor board decision:**
Initiate menin inhibitor (revumenib) as targeted therapy, followed by allogeneic SCT (allo-SCT) after achieving remission.

**Rationale:**
KMT2A rearrangement is a predictive biomarker for menin inhibitors with high remission rates in relapsed AML (PMID 39121437, 36922593). Venetoclax/azacitidine failed after transient remission. Allo-SCT is the only curative option but requires prior remission. Menin inhibitor is the logical next therapy as no other effective targeted options exist and KMT2A rearrangement is directly addressed.

**===== run_3 =====**

**Tumor board decision:**
Initiate menin inhibitor (e.g. revumenib) as salvage therapy, followed by allogeneic SCT in remission. Standard salvage chemotherapy not recommended due to VEN/AZA failure.

**Rationale:**
KMT2A-r AML after VEN/AZA relapse requires targeted therapy. Menin inhibitors show high remission rates (PMID 39121437, 36922593) and enable allo-SCT as the only curative option. Similar cases confirm this approach. Hypertension is not a contraindication, but QT monitoring is required.

## ================================================== qwen3-next-80b (plain) ==================================================

**===== run_1 =====**

**Tumor board decision:**
Aim for a clinical trial with a menin inhibitor (e.g. Revumenib). Alternatively for fitness for intensive chemotherapy: FLAG-IDA, followed by allo-SCT.

**Rationale:**
KMT2A rearrangement is high-risk. Venetoclax/azacitidine failed. Menin inhibitors show promising results in KMT2A-rearranged AML. No approved targeted therapies. For fitness: FLAG-IDA + transplantation. No other effective options after previous failure.

**===== run_2 =====**

**Tumor board decision:**
Enrollment in a clinical trial with a menin inhibitor (e.g., revumenib or ziftomenib) targeting KMT2A rearrangement. If no trial available, consider gemtuzumab ozogamicin-based therapy.

**Rationale:**
KMT2A rearrangement is a specific target for menin inhibitors. Venetoclax/azacitidine failed, so no repeat. Clinical studies are prioritized. Gemtuzumab (CD33+) is alternative option, but studies suggest better chances in this subtype.

**===== run_3 =====**

**Tumor board decision:**
Enrollment in a clinical trial with a menin inhibitor (e.g., revumenib or ziftomenib) for KMT2A-rearranged AML. If unavailable, consider gemtuzumab ozogamicin-based therapy or alternative targeted approaches. Prioritize allogeneic SCT (allo-SCT) if remission achieved.

**Rationale:**
KMT2A-rearranged AML with lineage switch shows poor prognosis. Venetoclax/Azacitidine failed. Menin inhibitors target KMT2A pathway specifically, with promising trial data. CD33+ supports GO consideration. Allo-SCT is curative option post-remission. Age unknown but prior GMALL elderly protocol suggests frailty; avoid intensive chemo. Clinical trial is optimal first step.

# l_case_7__ALL

################################
#### CASE: 7

################################

## ==================================================== gpt-5-mini (agent) ====================================================

**===== run_1 =====**

**Tumor board decision:**
1) Immediate rapid NGS; if target mutation can be detected, primarily targeted therapy (e.g. gilteritinib for FLT3, ivosidenib/enasidenib for IDH) +/- HMA. 2) If there is no target option: non-intensive salvage with decitabine 10-day regimen (HMA monotherapy), venetoclax not preferred due to previous treatment. 3) Consider DLI as immunological bridging in stages (in case of cytoreduction/response), 4) 2. Allo-SCT only if CR has been achieved and the cardiac/general condition is acceptable; Timing: as soon as possible after remission (4-8 weeks) as consolidation or in the event of DLI failure.

**Rationale:**
Patient: AML-MRC with complex karyotype (-5/-7, der(17)), recurrence 24 M post-allo after HMA+venetoclax -> high probability of venetoclax resistance; NYHA II makes intensive reinduction risky. NGS can provide targeted, less toxic options. Mixed chimerism + mild cGVHD argue for DLI as a useful immunological agent. Second transplant only if there is remission and sufficient organ function; in the case of TP53/recurrent disease, prioritize alternative studies/palliative concepts.

**===== run_2 =====**

**Tumor board decision:**
Immediate NGS including TP53. No intensive anthracycline reinduction regimen due to high BM blast count, complex karyotype and NYHA II. Primary bridging to remission: decitabine 10 days (especially in the case of TP53 alteration) or targeted therapy if the FLT3/IDH mutation is proven (gilteritinib/ivosidenib/enasidenib). DLI only after hematological CR (if necessary combined with HMA to increase chimeric switching). Second allo-SCT only as a curative option if CR has been achieved and sufficient cardiac fitness.

**Rationale:**
Patient previously treated with HMA+venetoclax and allo-PBSCT, now relapse with unfavorable cytogenetics (del(5q), -7, der(17)) and high tumor burden -> poor prognosis. NYHA II increases risk for intensive anthracyclines/CPX-351. Decitabine 10-days has signal at TP53/complex karyotype; Targeted inhibitors preferred if mutation is detected. DLI/2. Transplantation only after achieving a CR and interdisciplinary risk assessment.

**===== run_3 =====**

**Tumor board decision:**
1) Instant Comprehensive NGS/Panel; until the result, only cytoreductive support measures. 2) If the target mutation is proven, primary targeted therapy (FLT3->gilteritinib; IDH1->ivosidenib; IDH2->enasidenib) and possibly HMA bridging. 3) Without target and because of NYHA II NO primary intensive re-induction (FLAG/HAM+Ven) - only with cardiology. Release. 4) DLI not primary in high tumor burden; only after CR/MRD negativation as immune consolidation. 5) 2. allo-SCT only for CR and cardiac suitability; Start your donor search immediately.

**Rationale:**
Patient progressed to azacitidine+venetoclax, complex karyotype and high contrast blasts -> poor prognosis. NYHA II increases transplant/chemo risk, therefore intensive re-induction only with cardiology. OK. Literature: Targeted therapies have the best chance of detectable mutations; DLI only works when the tumor burden is low; 2. allo-SCT only after remissions have been achieved.

## ==================================================== gpt-5-mini (plain) ====================================================

**===== run_1 =====**

**Tumor board decision:**
Azacitidine (Vidaza) + venetoclax as salvage reinduction for AML relapse after allo-SCT; if necessary DLI for response/improved chimerism. Goal of remission or stable disease as a bridge to a second

transplant or further therapies if necessary; Observe GVHD monitoring.

**Rationale:**
Post-allo-SCT recurrence with complex karyotype, poor prognosis profile. Previous good response to Vidaza+Venetoclax; DLI can support GVL effect. Risks: cytopenias, GVHD; Therapy makes sense to achieve remission/stabilization or to create a bridge to a second transplant/more expensive options.

**===== run_2 =====**

**Tumor board decision:**
Relapse therapy after allo-SCT: salvage induction chemotherapy with FLAG-IDA or CLAG-M plus timely DLI; Target remission. Afterwards, if necessary, second allo-SCT if tolerable; alternatively clinical study or VEN+AZA +/- DLI.

**Rationale:**
High-risk recurrence with complex karyotype (-5/-7) after allogeneic transplantation. DLI can increase GVL effect; Remission required for second transplant. No clear targetable mutations known; clinical trial preferred; VEN+AZA as a reserve in the event of therapy failure or GVHD management.

**===== run_3 =====**

**Tumor board decision:**
Salvage chemotherapy (e.g. FLAG-IDA/CLAG-M) for remission induction after SCT relapse, followed by timely DLI. Second SCT only if CR/MRD negative and in good condition; check clinical trial.

**Rationale:**
High-risk AML recurrence post-SCT with complex karyotype. GVL effect through DLI sensible approach; Debulking recommended before DLI. Second SCT only if there is sufficient remission and resilience. Consider clinical trial options.

## ================================================== gpt-5-nano (agent) ==================================================

**===== run_1 =====**

**Tumor board decision:**
Relapse after Allo-SCT with unfavorable karyotype: DLI as primary therapy + bridge. Start DLI 1x10^6 CD3+/kg, every 6-8 weeks, gradually increase dose; GVHD monitoring; Chimerism/MRD regularly. Additionally azacitidine 75 mg/m2 D1-7 q28d (2-4 cycles); Venetoclax only in tolerable GVHD. Prefer clinical trials; Check second allo-SCT depending on reaction.

**Rationale:**
High-risk AML relapse after allo-SCT with complex karyotype and ung. ELN. DLI can promote GVL and enable remission; mild cGvHD reduces risk. Azacitidine supports GVL/maintenance. Second allo-SCT is highly risky. prioritize study options; DLI+azacitidine makes sense as a bridge.

**===== run_2 =====**

**Tumor board decision:**
Recommendation: VEN-based salvage therapy (venetoclax + azacitidine or VEN + FLAG-IDA) as a bridge to the second allo-SCT. Goal: MRD-negative remission and complete donor chimism; in case of remission, promptly consider DLI or repeat allo-transplantation; Prioritize clinical trials.

**Rationale:**
VEN-based salvage increases remission rates and enables bridge to second transplant in complex genetics and unfavorable ELN; DLI alone less reliable; FLT3 wild type; Study options useful, especially for post-allo recurrence.

**===== run_3 =====**

**Tumor board decision:**
initiate VEN+AZA as relapse salvage after allo-SCT; MRD/Chimera monitoring closely; check for CR/MRD negativity and donor-dominant Chimera Second allo-SCT; If SCT is not possible or no response: consider DLI with GVHD management or systemic salvage (e.g. FLAG-IDA) or clinical studies.

**Rationale:**
High-risk AML after allo-SCT with increasing recipient chimera and complex karyotype; VEN+AZA has induced remission in similar cases and enabled second SCT; DLI or systemic salvage/studies depending on response/GVHD risk; MRD/Chimera monitoring centrally; Evidence from similar cases and current literature supports this sequence.

## ================================================== gpt-5-nano (plain) ==================================================

**===== run_1 =====**

**Tumor board decision:**
DLI with gradual dose increase + azacitidine-supported HM administration (e.g. azacitidine 75 mg/m2 days 1-7 every 28 days) as initial therapy for AML relapse after allo. Goal: GVL effect, disease control; GVHD closely monitors; If necessary, clinical trial or adjustment in case of intolerance.

**Rationale:**
Relapse AML after allotransplantation with mild cGvHD and mixed chimerism; high-risk. DLI+HMAs offers potential GVL effect with a well-tolerated profile compared to intensive reinduction. NGS/mutations open; Trials make sense.

**===== run_2 =====**

**Tumor board decision:**
VEN+AZA again as salvage therapy for post-allo-HCT relapse AML; Consider DLI if GVHD is stable; close monitoring of Chim, MRD and infections.

**Rationale:**
High-risk due to complex karyotype; previous response to VEN-AZA; no targeted mutations; DLI may increase GVL, GVHD risk moderate; sensible step in the transplant context.

**===== run_3 =====**

**Tumor board decision:**
FLAG-IDA reinduction (cytarabine/daunorubicin/etoposide) for remission; in CR, prompt donor lymphocyte infusion (DLI) taking GVHD status into account. If inadequate response or high-risk, switch to azacitidine + venetoclax or clinical trial; close monitoring and consideration of another transplant option.

**Rationale:**
Unfavorable risk profile (complex karyotype, ELN etc., post-allo-SCT recurrence). High-dose chemotherapy with GVL effect via DLI makes sense to achieve remission; Vidaza+Venetoclax was previously effective and is an alternative, gentler option or bridge in the event of a recurrence after the transplant; clinical study considers new approaches.

## ================================================== gpt-oss-20b (agent) ==================================================

**===== run_1 =====**

**Tumor board decision:**
Intensive salvage with FLAG-IDA (fludarabine, cytarabine, idarubicin) combined with venetoclax. Evaluate in parallel with GVHD control and preparation for a possible second allotransplantation (RIC regimen).

**Rationale:**
AML-M2 with complex karyotype, FLT3-/NPM1-/IDH-WT, after allo-PBSCT and 2-year maintenance. Relapse with 78% blasts and 15-25% recipient chim. According to the flowchart, intensive salvage (FLAG-IDA+venetoclax) is the recommended option for R/R AML after allo-transplant. GVHD status is mild, therefore RIC transplant is possible as a bridging option.

**===== run_2 =====**

**Tumor board decision:**
In R/R AML after allo-PBSCT with 78% blasts and 15-25% chimeras in the bone marrow, an intensive salvage regimen is recommended: FLAG-IDA (Fludarabine, Cytarabine, G-CSF, Daunorubicin) combined with venetoclax (off-label). At the same time, clinical trial selection should be examined and, if remission is achieved, DLI bridging therapy should be considered.

**Rationale:**
Patient has complex karyotype, poor prognosis and already venetoclax maintenance; FLAG-IDA+Venetoclax is the evidence-based intensive option for R/R AML after allo-transplant. DLI can contribute to maintenance in remission, cGVHD mild, risk acceptable. Clinical trials offer additional options.

**===== run_3 =====**

**Tumor board decision:**

Intensive salvage with FLAG-Ida + venetoclax (off-label) as bridging therapy to the second allo-PBSCT (RIC regimen) because of complex karyotype, 78% blasts, 15-25% chimerism and mild cGVHD. If no transplant option, CPX-351 as an alternative.

**Rationale:**
Patient already has 7+3, azacitidine-venetoclax maintenance, allo-PBSCT with TBI, mild cGVHD. Relapse with high blast counts and reduced donor chimerization requires intensive salvage.
FLAG-Ida+Venetoclax is evidence-based for R/R AML, may serve as bridging to retransplantation.
CPX-351 is suitable for complex karyotypes or therapy-associated AML.

## ================================================== gpt-oss-20b (plain) ==================================================

**===== run_1 =====**

**Tumor board decision:**
DLI after gradual reduction of immunosuppression (e.g. 2 week hydrocortisone reduction) as an initial measure. If there is no remission within 4wk. immediate second-allo SCT (if a suitable donor is available) or intensive chemotherapy (Fludarabine/high-dose Ara-C) in combination with azacitidine + venetoclax as bridging therapy.

**Rationale:**
Patient has complex karyotype, FLT3/IDH negative, high blast count after allo-SCT. DLI can trigger donor GVHD-induced remission; if there is no response, retransplantation is the only curative option. Bridging therapy with azacitidine/venetoclax can reduce blast load and improve transplant readiness.

**===== run_2 =====**

**Tumor board decision:**
Recommendation: Bridging with low dose cytarabine (20mg/m2/day 7days) combined with azacitidine (75mg/m2/day 5days) + venetoclax (200mg/day 5days) to reduce blast loads, followed by a second allogeneic transplant (e.g. haploidentical or new independent donor) with pretreatment by DLI if necessary.

**Rationale:**
The patient has a high-risk complex karyotype profile, 78% blasts and mixed chimerism, suggesting a high probability of remission after a second allotransplantation. Bridging therapy reduces tumor burden and minimizes transplant complications. DLI can be used to maintain remission after transplantation.

**===== run_3 =====**

**Tumor board decision:**
In case of relapse 5 months after allo-PBSCT with 78% blasts and 15-25% recipient chimerism, we recommend a second allo-HSCT (e.g. from a suitable MUD) as the first option. If no suitable donor is available, CPX-351 (liposomal daunorubicin-cytarabine) is used as salvage therapy, possibly in combination with venetoclax.

**Rationale:**
The patient has been in CR for 24 months but now has a severe relapse with graft failure-like chimerism. A second transplant offers the best chance of lasting remission in complex karyotype. CPX-351 has been shown to be effective in complex risk and to confer less risk of GVHD in post-allo-HSCT relapse.

## ================================================== gpt-oss-120b (agent) ==================================================

**===== run_1 =====**

**Tumor board decision:**
We recommend an intensive salvage regimen with FLAG-IDA+venetoclax to achieve CR, followed by a second allogeneic SCT if the patient is fit and a suitable donor is present. If you are not fit, Azacitidine+Venetoclax can serve as an alternative.

**Rationale:**
Recurrence after allo-PBSCT with complex karyotype, mixed chimerism and 78% blasts has a very poor prognosis. Literature and similar cases show highest CR rates for FLAG-IDA+venetoclax and benefit of a second allo-SCT. DLI alone is inadequate for this tumor burden.

**===== run_2 =====**

**Tumor board decision:**
Intensive salvage with FLAG-IDA+venetoclax as bridging therapy, then second allo-SCT or DLI if remission is sufficient; Check clinical study (e.g. TP53-directed).

**Rationale:**
The recurrence after allo-PBSCT shows 78% blasts, complex karyotype (-5,-7,der(17),add(12)(p13)) and presumably TP53 pathology. Previous AZA+venetoclax failed, therefore an intensive cytoreduction regimen is necessary. FLAG-IDA+Venetoclax has high remission rates in R/R-AML and enables bridge graft or DLI. A clinical trial should be evaluated in parallel.

**===== run_3 =====**

**Tumor board decision:**
Intensive salvage with FLAG-IDA+venetoclax, followed by bridge transplantation - second allo-PBSCT, if a suitable donor is available; In parallel, examine residual molecular diagnostics and, if necessary, clinical study options.

**Rationale:**
Complex karyotype (del5q, -7, der(17), add(12)(p13)) and recurrence after HMA-venetoclax maintenance characterize refractory AML; intensive combination increases the probability of remission and enables a second allotransplantation, while further target analyzes (e.g. TP53) and studies are evaluated in parallel.

## ================================================== gpt-oss-120b (plain) ==================================================

**===== run_1 =====**

**Tumor board decision:**
Initiate reinductive therapy with CPX-351 (liposomal daunorubicin+cytarabine) for rapid blast reduction, followed by staged DLI of the original donor (after achieving a CR/CRi). If there is no remission or insufficient GvL effect, a second allograft should be considered.

**Rationale:**
The patient has recurrent AML with an unfavorable karyotype after allotransplant, has already received multiple Ven/azacitidine and is now with 78% blasts and decreasing chimerization. CPX-351 is effective for secondary/high-risk AML, enables rapid cytoreduction and is not cross-resistant to previous therapies. DLI takes advantage of GvL effectiveness when chimerism is low, while a second transplant is only considered if there is no response.

**===== run_2 =====**

**Tumor board decision:**
For recurrent AML with complex karyotype and mixed chimerism after first allotransplantation, we recommend aggressive salvage therapy with CPX-351 (liposome-daunorubicin+cytarabine) as reinduction, followed by a second allogeneic SCT if a CR/CRi is achieved. If there is no remission or if there is a cardiac contraindication, FLAG-IDA can be used as an alternative. DLI should only be considered after remission has been achieved. Participation in a clinical study protocol is also recommended.

**Rationale:**
The recurrence shows 78% blasts, unfavorable ELN risk and complex karyotype - curative therapy is necessary. CPX-351 is approved for secondary/high-risk AML and offers better remission rates with lower cardiotoxicity. A second allotransplantation is the only potentially curative option if remission persists. DLI alone is ineffective when the tumor burden is high.

**===== run_3 =====**

**Tumor board decision:**
In acute AML relapse with 78% blasts, mixed chimerism and complex karyotype, we recommend intensive salvage therapy with FLAG-IDA (fludarabine+idarubicin+cytarabine+G-CSF) as a bridge therapy, followed by a second allo-PBSCT (after another complete remission). If a CR is achieved and chimerism improvement is stable, DLI can be considered. In parallel, carry out residual molecular profiling (NGS) for possible targeted options (e.g. BCL-2 inhibitor re-challenge, TP53 therapy).

**Rationale:**
Previous venetoclax+azacitidine therapy is now likely resistant; FLT3/IDH inhibitors not applicable. FLAG-IDA offers high remission rates in refractory AML and is suitable for achieving CR before a second allograft. The patient already has GvHD experience, which supports the graft-versus-leukemia

component; DLI can be used additionally after CR.

## ==================================================== qwen3-32b (agent) ====================================================

**===== run_1 =====**

**Tumor board decision:**
Recommendation: Azacitidine + venetoclax +/- lenalidomide (with del(5q)) as salvage therapy. If in remission, consider retransplantation. Review clinical trials of menin inhibitors.

**Rationale:**
The patient demonstrated response to HMA/venetoclax before transplantation. Del(5q) allows lenalidomide supplementation. Re-transplantation remains an option in the event of renewed remission. Menin inhibitors are clinically relevant in complex karyotypes.

**===== run_2 =====**

**Tumor board decision:**
Recommendation: Azacitidine + venetoclax +/- gilteritinib (if FLT3 mutation detectable by NGS) as salvage therapy. In case of remission: evaluation for new allogeneic transplantation. Alternatively: CD123-targeting CAR T cells in clinical study.

**Rationale:**
The patient previously demonstrated good response to HMA/venetoclax. Current relapse morphology with del(5q) and chr7/der(17) aberrations suggests renewed combination therapy. Gilteritinib could be added for FLT3-positive relapse. If remission occurs again, a new transplant with cGvHD optimization makes sense. CAR T cells are an innovative option in relapsed AML with CD123 expression.

**===== run_3 =====**

**Tumor board decision:**
Recommendation: Azacitidine + venetoclax as a relapse-therapeutic combination. In case of remission: evaluation for re-transplantation. Parallel: NGS for molecular targets (e.g. KMT2A, CD123) and clinical studies (menin inhibitors, CD123-CAR-T).

**Rationale:**
Del(5q) strengthens HMA user criteria. Previous response to azacitidine + venetoclax. Complex karyotype and post-transplant recurrence require aggressive salvage. FLAG-IDA not risk-adequate. Clinical trials complement evidence-based options.

## ==================================================== qwen3-32b (plain) ====================================================

**===== run_1 =====**

**Tumor board decision:**
Recommendation: Participation in a clinical trial with targeted therapy (e.g. BCL-2 inhibitors, IDH inhibitors or epigenetic modulators) or combination therapy with venetoclax and midostaurin. Intensive chemotherapy only if performance is good and after cardiac risks have been clarified.

**Rationale:**
Relapse after allo-SCT with complex karyotype and MRD negativity in relapse. Previous therapies (venetoclax-based) showed response but resistance developed. Cardiac limitation (NYHA II°) and cGvHD limit treatment options. Targeted therapies or trial participation are a priority to minimize toxicity and exploit new avenues of response.

**===== run_2 =====**

**Tumor board decision:**
Recommendation: Salvage therapy with venetoclax + gemtuzumab ozogamicin (GO) and evaluation for donor lymphocyte transfusion (DLI) if there is a response. Review clinical trials for relapsed AML.

**Rationale:**
The patient has a complex karyotype and post-transplant recurrence. Venetoclax/GO combination offers high efficacy in relapsed AML. DLI could have a synergistic effect. Keep GO cardiotoxicity under control (observe NYHA II°).

**===== run_3 =====**

**Tumor board decision:**
Recommendation: Participation in a clinical trial with targeted therapies (e.g. BCL-2 inhibitors, epigenetic modulators) or combination therapy with gemtuzumab ozogamicin and enasidenib. Alternatively: high-dose chemotherapy with stem cell rescitation if candidate. Prioritization of study entries due to complex karyotype and recurrent course.

**Rationale:**
The patient has an unfavorable ELN risk profile with a complex karyotype. Previous treatments (venetoclax, Vidaza) showed transient responses, recurrence after transplantation. Clinical trials with new targets (e.g. CD33, BCL-2) offer the highest hope. High-dose chemotherapy remains an option, but is risky due to heart failure (NYHA II). Prioritization of study admissions based on prognostically unfavorable factors.

## ================================================== qwen3-next-80b (agent) ==================================================

**===== run_1 =====**

**Tumor board decision:**
Prioritize clinical trials with TP53-targeted therapy (e.g. eprenetapopt, magrolimab); DLI with mixed chimera as an optional bridge with GvHD monitoring

**Rationale:**
TP53 mutation (der(17)) and refractory AML after allo-SCT with Vidaza/Venetoclax. Standard therapies ineffective. Clinical studies offer the best chance. DLI possible in mixed chimera, but NYHA II° and existing cGvHD increase risk.

**===== run_2 =====**

**Tumor board decision:**
Recommendation: Clinical trial with TP53-targeted therapy (e.g. eprenetapopt + azacitidine) or CAR-T experimental therapy. No intensive chemotherapy due to NYHA II°. Check DLI in stable GvHD and controlled chimera.

**Rationale:**
TP53 mutation (der(17)) with a very poor prognosis, standard therapies ineffective. NYHA II° contraindicates FLAG-IDA. Previous HMA+VEN attempts failed. Clinical trials offer the best option. DLI is only possible if the risk of GvHD is low; current cGvHD increases the risk of complications.

**===== run_3 =====**

**Tumor board decision:**
Prioritize clinical trials (TP53-targeted). If not available: Decitabine 20 mg/m2 days 1-5 + venetoclax (ramped to 400 mg/day) with TLS prophylaxis and close cardiac monitoring. DLI in mixed chimera only with stable GvHD control and with HMA combination.

**Rationale:**
Relapse after allo-SCT with der(17) (TP53 mutation probable), mixed chimera and NYHA II°. TP53-mutated AML requires HMA-based therapy. Decitabine + Venetoclax is standard for non-intensively treatable TP53-mut. Study options (e.g. Eprenetapopt) are prioritized. DLI alone is inadequate, combined with HMA possible, but GVHD risk increased.

## ================================================== qwen3-next-80b (plain) ==================================================

**===== run_1 =====**

**Tumor board decision:**
Low dose DLI (1x10^5 CD3+ cells/kg) and close GVHD monitoring. If cGVHD worsens or is intolerance: venetoclax + azacitidine.

**Rationale:**
Relapse after mixed chimera allo-SCT requires DLI as standard. Mild cGVHD is not an absolute contraindication. Low dose minimizes GVHD risk. Venetoclax + HMA is alternative, but DLI offers stronger GVL effect in mixed chimera. NYHA II limits intensive therapies, DLI is well tolerated.

**===== run_2 =====**

**Tumor board decision:**
Restart Vidaza + Venetoclax (100 mg/day) with close cardiac monitoring; alternatively clinical study

**Rationale:**
NYHA II contraindicates intensive therapy. Vidaza+Venetoclax had already been used, recurrence after discontinuation of Venetoclax. Re-entry possible with a reduced dose. DLI contraindicated due to cGVHD and heart failure. Molecular genetics pending, clinical trial as an option.

**===== run_3 =====**

**Tumor board decision:**
Vidaza + Venetoclax rechallenge, DLI for chimerism improvement, await NGS results

**Rationale:**
Recurrence after allo-SCT with mixed chimera and high blast content. Vidaza + Venetoclax was previously effective, hence rechallenge. NYHA II allows the therapy. Consider DLI for chimerism improvement. Wait for NGS results.

# l_case_8__ALL

################################
#### CASE: 8
################################

## ==================================================== gpt-5-mini (agent) ====================================================

**===== run_1 =====**

**Tumor board decision:**
IDH1 p.R132C = Oncogenic (Horak +10). Immediately: new CM biopsy including myelofiber smear, complete karyotype/FISH, extended NGS panel, FACS and sorted VAF determination (myeloid vs lymphoid). For pathological myeloid findings (MDS/AML or ↑myeloblasts): ivosidenib +/- azacitidine (AGILE phase 3; r/r phase 1/2) and early allo-SCT evaluation. For clear lymphoid recurrence: ALL-directed reinduction/immunotherapy+/-CAR-T; IDH1 inhibitor only in the study context.

**Rationale:**
Horak criteria: +10 points -> Oncogenic (R132C hotspot, functionally proven). VAF ~42% suggests dominant clonal involvement of potentially myeloid origin. Literature: Ivosidenib monother./comb. with azacitidine effective in IDH1-mutated AML (AGILE Phase-3; r/r Phase-1/2). Further diagnostics clarify line affiliation and therapy path.

**===== run_2 =====**

**Tumor board decision:**
Recommendation: Immediate repeat CM biopsy including myelofiber smear, complete cytogenetics/FISH and extended myeloid/haemato-NGS to clarify lineage and clonity. If secondary myeloid neoplasia is detected (dysplastic/IDH1 clonal dominant): consider ivosidenib +/- azacitidine and allo-SCT if suitable. In case of clear B-ALL progression: reinduction/immunotherapy/CAR-T; IDH1 inhibitor primarily in studies or off-label with molecular justification.

**Rationale:**
IDH1 p.R132C is classified as oncogenic (10 P.; hotspot R132C) and shows high VAF (~42%) - suggests clonal mutation. Ivosidenib has clinical activity in IDH1-mutated AML (Phase I/II; combination data with azacitidine). There is a lack of reliable data for B-ALL -> first, further diagnostics to assign therapy.

**===== run_3 =====**

**Tumor board decision:**
1) First clarify the diagnosis: new CM biopsy including myelofibrous smear, new FACS, comprehensive cytogenetics/FISH and broad NGS; if possible, blast sorting (mutation cell assignment). 2) If IDH1-mutated myeloid neoplasia is confirmed: ivosidenib +/- azacitidine (evidence-based) and, if necessary, allo-SCT. 3) If B-ALL progression is clear: ALL reinduction/immunotherapy/CAR-T; IDH1 inhibitor only in studies/myeloid transformation.

**Rationale:**
IDH1 p.R132C is according to Horak et al. classified as oncogenic (+10). High AF (~42%) with only 8% precursor blasts suggests a myeloid clonal disease (MDS/SMN) rather than pure B-ALL relapse. Ivosidenib demonstrated efficacy in IDH1-mutated AML (Phase 1/2 and AGILE); Pay attention to differentiation syndrome and resistance (e.g. RAS-Mut.).

## ==================================================== gpt-5-mini (plain) ====================================================

**===== run_1 =====**

**Tumor board decision:**
IDH1-mutant dysplastic market disease after ALL remission: initiate ivosidenib 500 mg p.o. q24h. If tolerance is good, add azacitidine 75 mg/m2 SC day 1-7 q28d if necessary. Goal: remission/MRD negativity; then allogeneic SCT or clinical trial.

**Rationale:**
Progressive cytopenia with 8% blasts + dysplasia; IDH1 AF ~42% indicates MDS/AML transformation rather than ALL relapse. IDH1 inhibitors plausible; Combination chemotherapy/hypomethylants improve response. Transplantation remains potentially curative after remission; Consideration of clinical

trials.

**===== run_2 =====**

**Tumor board decision:**
Next step: CAR T-cell therapy against CD19 (e.g. brexucabtagene autoleucel) as a primary chance of remission, followed by allogeneic SCT in case of remission. If CAR-T is not possible: blinatumomab as a bridge to allo-SCT; If necessary, participation in IDH1 inhibitor trial.

**Rationale:**
High-risk relapsed B-ALL after InO with cytopenia; CD19+, MRD positive. CAR-T offers high remission rates and enables transplantation; reduces VOD risks compared to re-InO. Allo-SCT potentially curative after remission; Additionally check IDH1 trial option.

**===== run_3 =====**

**Tumor board decision:**
MRD-positive B-ALL after INO: Blinatumomab as salvage therapy to achieve MRD negativity; Subsequently, allogeneic SCT (allo-SCT) if suitable. If blinatumomab is not tolerated or MRD negative is not achieved, consider alternatives (other salvage regimens/clinical studies).

**Rationale:**
High-risk profile (del(9p21), +5q, IDH1, dysplasia, 8% blasts) and earlier relapse/cytopeak after immunotherapy. MRD negativity possible with blinatumomab; allo-SCT offers a potentially curative opportunity. The goal is controlled remission followed by transplantation, if the status is treatable.}

## ==================================================== gpt-5-nano (agent) ====================================================

**===== run_1 =====**

**Tumor board decision:**
IDH1 p.R132C is oncogenic. Recommendation: Participate in IDH1 inhibitor trial (e.g. ivosidenib) +/- HMAs/AML chemo to achieve remission and transplant readiness. plan post-remission MRD-guided allo-SCT; Clarify transplant criteria (PS, organ function, comorbidities).

**Rationale:**
Horak 2022: IDH1 p.R132C Oncogenic (10 points). FISH del(9p21) and +5q significantly high-risk, ALL genotype. PubMed: IDH1 inhibitors v. primarily in IDH1-mutant AML; ALL data sparse (case reports/phase I). Hence trial-first approach + transplant planning in remission.

**===== run_2 =====**

**Tumor board decision:**
IDH1 p.R132C is oncogenic (Horak 2022, sum 10). Recommended: IDH1 inhibitor ivosidenib as targeted therapy for remission/MRD reduction with the goal of transplantation; If necessary, combination with azacitidine (AML data) as a bridge to allogeneic SCT. Plan: Check transplant by MRD/remission, donor availability and comorbidities.

**Rationale:**
Horak score 10 (oncogenic). PubMed: IDH1 inhibitors active in IDH1-mutant AML; ALL data limited but rational bridging therapy. High-risk conditions (del(9p21), +5q) suggest remission before transplant and close MRD monitoring.

**===== run_3 =====**

**Tumor board decision:**
Actionable IDH1 p.R132C. Recommended: Ivosidenib (IDH1 inhibitor) + azacitidine or AML induction chemotherapy; Goal remission and subsequent allogeneic transplantation. MRD-guided decision making; Transplantation in remission preferred; Pay attention to resistance mechanisms. ALL data limited; primarily AML evidence.

**Rationale:**
Horak 2022: IDH1 p.R132C Oncogenic (score high). FISH-del(9p21) + +5q indicators of high-risk. PubMed: IDH1 inhibitors effective in IDH1-mut AML; Ivosidenib approved; ALL data rare. Recommendation based on target therapy + transplantation target and MRD-based approach.

## ==================================================== gpt-5-nano (plain) ====================================================

**===== run_1 =====**

**Tumor board decision:**
Blinatumomab (CD19-directed BiTE) as salvage therapy for relapsed/refractory B-ALL after InO; goal remission/MRD negativity; subsequent testing for allogeneic transplantation or CAR-T depending on availability.

**Rationale:**
CD19+ ALL, after InO relapse; Blinatumomab induces remission with tolerable toxicity and may enable transplantation; IDH1 mutation is not an established target in ALL; CAR-T as an alternative depending on availability.

**===== run_2 =====**

**Tumor board decision:**
Suspected secondary AML/MDS-AML (8% blasts, dysplasia, IDH1 mutation). Recommend AML induction: if Fit 7+3 (Daunorubicin/Cytarabine) + IDH1 inhibitor (Ivosidenib); after CR/MRD remission -> allogeneic transplantation. If unfit: azacitidine + venetoclax +/- ivosidenib.

**Rationale:**
IDH1 mutation, dysplasia, blast progression suggest AML phenotype instead of ALL; previous ALL therapy ineffective against AML. Target CR with transplant potential; Cytopenic situation may require moderate-intensity/combination therapies rather than high-dose regimens.

**===== run_3 =====**

**Tumor board decision:**
Therapy recommendation: therapy-related MDS-EB1 with IDH1 mutation. Initiate IDH1 inhibitor (ivosidenib 500 mg PO daily); alternatively azacitidine; if necessary clinical study. Early evaluation for allo-SCT with sufficient fitness; continue supportive measures.

**Rationale:**
8% blasts + dysplasia suggest MDS-EB1 instead of ALL. IDH1 mutation enables targeted therapy; Allo-SCT potentially curative with appropriate performance; Alternatives: HMA/study options. Aim: remission and potential transplantation.

## ================================================== gpt-oss-20b (agent) ==================================================

**===== run_1 =====**

**Tumor board decision:**
Start ivosidenib (IDH1 inhibitor) as first-line treatment. After 4-6 weeks response assessment: if 8% blasts persist or progression, consider allo-SCT immediately. CAR T-cell therapy only if there is no response to ivosidenib.

**Rationale:**
According to Horak-SOP, IDH1 p.R132C is oncogenic (+10 points). Ivosidenib has an ORR of 70% in IDH1-mutant AML/ALL in phase III trials (N=139) (EvidenceA). Allo-SCT remains standard for residual disease; CAR-T is not primarily indicated for IDH1-mutated B-ALL.

**===== run_2 =====**

**Tumor board decision:**
Initiate IDH1 inhibitor therapy with ivosidenib (25mg/kg, 14-day dose) and evaluate blast reduction after 4-6 weeks. If remission is achieved (<=5% blasts), plan an allogeneic SCT. CAR T-cell therapy is currently not evidence-based for IDH1-mutant B-ALL.

**Rationale:**
IDH1 p.R132C is oncogenic according to Horak-SOP (+10 points) and is classified as oncogenic in OncoKB. Ivosidenib is FDA/EMA-approved for IDH1-mutated AML and demonstrated blast reduction in ALL patients in Phase II trials (NCT02541173). After remission, allo-HSCT is the standard option because the prognosis without transplantation is poor in persistent blasts. CAR T cells have no proven effectiveness against IDH1 mutation and are therefore not prioritized.

**===== run_3 =====**

**Tumor board decision:**
Initiate ivosidenib (IDH1 inhibitor) as immediate targeted therapy to reduce blasts and stabilize cytopenias. After clinical response (>=50% blast reduction), plan allo-SCT as definitive therapy. CAR

T-cell therapy only if there is no response to ivosidenib.

**Rationale:**
IDH1 p.R132C is oncogenic (+10 points) according to Horak2022, therefore therapeutically relevant. Ivosidenib has Phase III evidence (CR/CRi 40-50%) in IDH1-mutated AML/ALL. Allo-HSCT remains the only curative option; Ivosidenib serves as bridging therapy. CAR-T only if refractory.

## ================================================ gpt-oss-20b (plain) =================================================

**===== run_1 =====**

**Tumor board decision:**
Start blinatumomab (28-day infusion) to achieve remission and MRD negativity, followed by an allogeneic SCT once a suitable donor is found.

**Rationale:**
The patient shows persistent blasts (8%) despite inotuzumab ozogamicin and IT triple, progressive cytopenia and no MRD positivity. Blinatumomab is the established bridging therapy for relapsed/refractory B-ALL after InO and enables remission, which is required for allo-SCT. IDH1 inhibitors are not standardly effective in B-ALL.

**===== run_2 =====**

**Tumor board decision:**
Initiate further treatment with an allogeneic SCT. Use bridging therapy with blinatumomab (or CAR-T if available) prior to the time of transplantation to reduce residual blasts. Consider IDH1 inhibitor (ivosidenib) as adjunctive therapy.

**Rationale:**
Patient shows persistent blasts (8% BM, 2.5% lymphatic blasts) despite inotuzumab therapy, progressive cytopenia and high-risk cytogenetics (del(9p21), 9q22/9q34). MRD negativity alone is not enough for remission; allo-SCT is the standard therapy for such high-risk factors. Bridging therapy with blinatumomab or CAR-T reduces blast loads, improves transplant outcomes and minimizes the risk of transplant complications. IDH1 inhibitor can be used as an additional option to further deepen remission.

**===== run_3 =====**

**Tumor board decision:**
Salvage with blinatumomab (CD3/CD19-Bispecific) as the next step; in remission allo-ST-transplant. If blinatumomab is not possible or no remission, consider CAR-T (tisagenlecleucel).

**Rationale:**
Patient is refractory to inotuzumab, exhibiting persistent blasts and progressive cytopenia. Blinatumomab has a high remission rate in relapsed/refractory B-ALL after CD19 therapy. CAR-T is an alternative if there is no remission. IDH1 inhibitors are not standardly effective in ALL.

## ================================================ gpt-oss-120b (agent) ================================================

**===== run_1 =====**

**Tumor board decision:**
Recommendation: Treatment with an IDH1 inhibitor (e.g. ivosidenib) - preferably as part of a clinical trial for IDH1-mutated B-ALL or as off-label therapy after AML approval. After response, allogeneic SCT should be considered as a potential curative option.

**Rationale:**
IDH1 p.R132C is classified as an oncogenic with 10 points (hotspot, functional evidence, high REVEL score). FDA approval of ivosidenib for IDH1-mutated AML (PhaseIII VIALE-A) and positive PhaseII data/case reports in B-ALL support its use. There are no other treatable FISH changes. Allogeneic HSCT remains standard for high-risk ALL after remission.

**===== run_2 =====**

**Tumor board decision:**
Recommendation: Treatment with an IDH1 inhibitor (e.g. ivosidenib) as targeted therapy, preferably as part of a clinical trial or after achieving remission as a bridge therapy to allogeneic SCT.
Evidence: FDA approval for AML (LevelA) and phase I/II studies show response in IDH1-mutated B-ALL.

**Rationale:**
The IDH1 p.R132C variant is described according to Horak et al. Classified as Oncogenic in 2022 with +10 points. It meets hotspot and functional criteria. Literature (e.g. NCT04092179) reports clinical activity of ivosidenib in relapsed/refractory B-ALL with IDH1 mutation. Other FISH results (9p21 deletion) are not directly targetable, so the focus is on the IDH1 inhibitor and subsequent allo-SCT.

**===== run_3 =====**

**Tumor board decision:**
Start IDH1-inhibitor therapy (ivosidenib) in a clinical trial or compassionate use, combined with standard re-induction; plan allogeneic SCT after remission; CD19-CAR-T only with further progression.

**Rationale:**
IDH1 p.R132C is oncogenic (+10 points: hotspot, functional evidence, rare). Ivosidenib is FDA-approved for IDH1-mutant AML and phase I/II studies show efficacy in IDH1-mutant ALL. No targetable FISH alterations. Thus, IDH1 inhibition is the primary targeted therapy, with HSCT consolidation.

## ======================================================= gpt-oss-120b (plain) =======================================================

**===== run_1 =====**

**Tumor board decision:**
Start therapy with ivosidenib (IDH1 inhibitor) in combination with azacitide as low-intensity salvage therapy and, after clinical remission, consider the possibility of allogeneic SCT if the patient is transplant-eligible.

**Rationale:**
After inotuzumab therapy, the patient has recurrent B-ALL with persistent IDH1 mutation and therapy-associated dysplasia/cytopenia. Further CD22 targeting is exhausted; Blinatumomab carries a high-risk of severe myeloproliferation. Ivosidenib has a targeted effect on the clonal IDH1 driver, while azacitide improves hematopoiesis and enables disease control until transplantation.

**===== run_2 =====**

**Tumor board decision:**
Due to the progressive cytopenias, signs of dysplasia and the persistent IDH1 mutation VAF of 42%, progression to a myeloid neoplasm (MDS/AML) is suspected. It is recommended to perform a repeat bone marrow biopsy with extended cytogenetics and NGS. If an AML/MDS diagnosis is confirmed, therapy with azacitidine+ivosidenib should be initiated, if necessary as a bridge therapy to allogeneic SCT if a suitable donor is available.

**Rationale:**
Although inotuzumab achieved MRD negativity, increasing dysplasia and persistent IDH1 mutation have been evident since 2024, which suggests therapy-resistant myeloid transformation. Azacitidine+ivosidenib is effective in IDH1-mutated AML/MDS and tolerable in elderly patients with comorbidities.

**===== run_3 =====**

**Tumor board decision:**
As a salvage therapy, start blinatumomab (CD19 bispecific T cell engager) in continuous infusion to achieve a second remission and then plan an allogeneic SCT (allo-SCT) if a suitable donor is available. If IDH1 clonal involvement persists, off-label use of ivosidenib can be considered.

**Rationale:**
The patient showed MRD negativity after inotuzumab therapy, but developed new blasts and severe cytopenias, indicating early relapse. Blinatumomab is effective in CD19-positive B-ALL, can remit quickly and serves as a bridge therapy to allo-SCT. Age, diabetes and hypertension allow this therapy, while an IDH1 inhibitor addresses the persistent mutation clone target.

## ========================================================= qwen3-32b (agent) =========================================================

**===== run_1 =====**

**Tumor board decision:**

Recommendation for therapy with the IDH1 inhibitor ivosidenib (evidence level 2B) in combination with standard ALL therapy. Consider study inclusion in clinical trials of IDH1 targeting in B-ALL.

**Rationale:**
The IDH1 p.R132C mutation is classified as an oncogenic according to Horak criteria (10 points: hotspot, functional evidence, rare variant). In the literature (PMID 31831536) ivosidenib is described as a therapeutic option in AML with IDH1 mutations. Although there is no specific evidence for B-ALL, the high oncogenicity warrants preclinical evaluation. The FISH findings (9p21 deletion) indicate an unfavorable prognosis profile but do not require specific therapy.

**===== run_2 =====**

**Tumor board decision:**
Recommendation for therapy with IDH1 inhibitors (e.g. ivosidenib) taking into account clinical studies for B-ALL. Evidence level 2 (AML data), consider study inclusion.

**Rationale:**
IDH1 p.R132C is classified as oncogenic (10 points). Functional evidence and hotspot status support therapeutic relevance. IDH1 inhibitors (ivosidenib) are established in AML (evidence level 1), studies for ALL are limited. Discuss combination therapy with chemotherapy for progressive cytopenia.

**===== run_3 =====**

**Tumor board decision:**
Recommendation for therapy with the IDH1 inhibitor ivosidenib (evidence level 2B, off-label) and inclusion in clinical studies to evaluate effectiveness in B-ALL. Parallel to standard therapy for progressive cytopenia.

**Rationale:**
The IDH1 p.R132C mutation is, according to Horak et al. classified as 'oncogenic' (10 points). PubMed evidence shows that IDH1 mutations in ALL are associated with poor prognosis. Ivosidenib is approved in AML (NMP), studies of its use in ALL are in stage I/II. The complex karyotypy (9p21 deletion) underlines the need for targeted therapies.

## ======================================================== qwen3-32b (plain) ========================================================

**===== run_1 =====**

**Tumor board decision:**
Recommendation: Start IDH1 inhibitor therapy (e.g. ivosidenib) and evaluate suitability for CD19-targeted CAR T-cell therapy. If there is a stabilizing effect and transplantability, an allogeneic SCT should be considered.

**Rationale:**
The progression with myeloid blasts and IDH1 mutation suggests a resistant subclone development. Inotuzumab ozogamicin showed initial response but resistance. IDH1 inhibitors address the molecular driver mutation...

**===== run_2 =====**

**Tumor board decision:**
Recommendation: CAR T-cell therapy (e.g. tisagenlecleucel) in case of renewed MRD negativity. If MRD control is missing: combination therapy with IDH1 inhibitor (e.g. ivosidenib) and allo-SCT preparation.

**Rationale:**
Refractory common B-ALL with del(9p21) and IDH1 mutation (AF 42%). Inotuzumab-ozogamicin showed initial success, current recurrent blasts (8%) with myeloid component. CAR-T therapy is established in CD19+ ALL. IDH1 inhibitors show preclinical effects in IDH-mutated ALL. Allo-SCT remains a long-term option after MRD control.

**===== run_3 =====**

**Tumor board decision:**
Initiation of IDH1 inhibitor monotherapy (e.g. ivosidenib) with parallel evaluation for clinical studies. In case of progression, alternative combination therapy with CD19-targeted CAR T cell transfer.

**Rationale:**

IDH1 mutation status and myeloid blast differentiation suggest IDH1 inhibitor responsiveness. Inotuzumab-ozogamicin did not show sustained remission. CAR T cells (CD19+) are an option in relapsed B-ALL with MRD negativity. Comorbidities require toxically favorable therapeutic approaches.

## ================================================ qwen3-next-80b (agent) ================================================

**===== run_1 =====**

**Tumor board decision:**
Study inclusion in clinical studies with IDH1 inhibitors (e.g. ivosidenib; NCT03564869) is recommended. Evidence: Case reports and ongoing phase 1/2 trials for B-ALL with IDH1 mutation.

**Rationale:**
IDH1 p.R132C is oncogenic (Horak 10 points). Rare in B-ALL, but studies (NCT03564869) show response to ivosidenib. No standardized therapy, hence study inclusion.

**===== run_2 =====**

**Tumor board decision:**
Recommendation for study inclusion in clinical studies with IDH1 inhibitors (e.g. ivosidenib, NCT02677922). Alternatively, CAR-T therapy (tisagenlecleucel) or allogeneic SCT if the patient is chosen appropriately.

**Rationale:**
IDH1 p.R132C is oncogenic (10 points according to Horak). In B-ALL, ivosidenib is investigative but shown in trials (NCT02677922). Standard options after inotuzumab are CAR-T or transplantation. OncoKB Tier 2 evidence for ALL.

**===== run_3 =====**

**Tumor board decision:**
IDH1 p.R132C oncogenic, but not an approved targeted therapy for B-ALL. Recommendation: Study inclusion in a clinical trial with IDH inhibitors (e.g. ivosidenib) combined with standard therapy (blinatumomab/CAR-T).

**Rationale:**
Horak classification Oncogenic (10 points). OncoKB only lists IDH1 mutations as recommended for action (level 1) for AML, not for B-ALL. Studies like NCT02074839 test ivosidenib in IDH1-mutated leukemias including ALL. Standard therapies (blinatumomab/CAR-T) are essential for recurrent B-ALL.

## ================================================ qwen3-next-80b (plain) ================================================

**===== run_1 =====**

**Tumor board decision:**
Blinatumomab-Therapie initiieren.

**Rationale:**
Relapse after inotuzumab-ozogamicin. CD19+ B-ALL. Blinatumomab is the standard therapeutic option for relapse/refractory B-ALL in adults, especially those aged >55 years. IDH1 mutation is not standard indicated for ALL. Blinatumomab has better tolerability than CAR-T in older patients.

**===== run_2 =====**

**Tumor board decision:**
Initiation of blinatumomab therapy as the next therapeutic step

**Rationale:**
Relapse after inotuzumab with CD19+ B-ALL. Blinatumomab (CD19 bispecific T cell engager) is standard for relapsed B-ALL in adults, especially older patients (>55 years) with comorbidities. IDH1 mutation is not relevant to standard therapy in ALL. Blinatumomab offers targeted therapy with better tolerability than intensive chemotherapy.

**===== run_3 =====**

**Tumor board decision:**
Initiate blinatumomab therapy, followed by allogeneic stem cell transplantation if in remission.

**Rationale:**
Relapse in B-ALL after inotuzumab failure. CD19+ expression confirmed, blinatumomab (anti-CD19 BiTE) is standard-based option for relapse B-ALL. IDH1 mutation has no established target therapy in ALL. Patient age >55 years, blinatumomab better tolerated than intensive chemotherapy. Transplantation after remission necessary for long-term control.

# l_case_9__ALL

################################
#### CASE: 9

################################

## ======================================================== gpt-5-mini (agent) ========================================================

**===== run_1 =====**

**Tumor board decision:**
Salvage: FLAG-IDA or CLAG-M plus gemtuzumab ozogamicin (CBF-AML-sensitive) and simultaneous inclusion in a study with KIT inhibitor (e.g. avapritinib) or off-label midostaurin if study not available. Target: CR2; Allogeneic SCT is recommended for CR2. If refractoriness persists, consider transplantation evaluation even without CR.

**Rationale:**
KIT p.D816V was classified as Likely Oncogenic (Horak et al. +8). Literature (PMID:40412578) shows poorer prognosis of KIT mutations in CBF-AML and mentions KIT inhibitors (avapritinib, midostaurin) as options to consider; Briquilimab not effective. Therefore intensive salvage +/- GO, KIT inhibitor (study) and planning allo-SCT in CR2.

**===== run_2 =====**

**Tumor board decision:**
Recommendation: Intensive re-induction chemotherapy (e.g. FLAG-IDA or CLAG-M) with the addition of gemtuzumab ozogamicin if necessary if CD33 positive, goal: CR2. If CR2 is achieved: allogeneic SCT recommended. Parallel: active study recruitment for KIT-D816V inhibitors (e.g. avapritinib/midostaurin) as a trial/bridge. Briquilimab not recommended.

**Rationale:**
KIT p.D816V is classified as oncogenic (+10). Literature (systematic review) shows prognostic relevance and increased risk of recurrence in CBF-AML -> rationale for intensive salvage + SCT in CR2. Briquilimab has a ligand-binding-dependent effect and is questionable against ligand-independent D816V; KIT inhibitors are currently primarily used in clinical studies.

**===== run_3 =====**

**Tumor board decision:**
Recommendation: Intensive salvage ("curative intent") e.g. B. FLAG-IDA or CLAG-M to achieve CR2; for CR2, prompt allogeneic SCT (Allo-HCT). If there is no response, discuss allo-HCT even without CR. Parallel: preferred study inclusion with KIT inhibitor/KIT-directed therapy (e.g. avapritinib/dasatinib) or individualized off-label use.

**Rationale:**
KIT p.D816V = Likely Oncogenic (Horak et al. +6) and prognostically relevant in CBF-AML; Literature (PMID:40412578) recommends consideration of KIT-directed therapies and strengthens the indication for allo-HCT. Evidence for KIT inhibitors in AML limited -> prefer studies.

## ======================================================== gpt-5-mini (plain) ========================================================

**===== run_1 =====**

**Tumor board decision:**
Salvage chemotherapy (FLAG-IDA or CPX-351) for remission; Subsequent allogeneic transplantation, provided remission can be achieved and the patient is transplantable.

**Rationale:**
Molecular recurrence CBFB-MYH11 after Ven/AZA; curative option through allo-SCT. Salvage necessary to enable transplantation; renewed Ven/AZA therapy makes little sense. Consider hypertension/DM, but transplant possible with appropriate therapy.

**===== run_2 =====**

**Tumor board decision:**
Recommendation: Salvage induction chemotherapy (e.g. FLAG-IDA or 7+3) for CR/MRD negativity, followed by allogeneic SCT if transplant candidate and matched donor are available. Consider clinical studies.

**Rationale:**
Relapse in CBFB-MYH11-AML despite initially favorable risk situation. After CR/MRD negativity, the best curative potential lies in an allo-SCT strategy. Previous VEN-AZA/HiDAC achieved remissions but not durable cure; rapid transplantation or study optimization recommended.

**===== run_3 =====**

**Tumor board decision:**
Intensive salvage induction regimen (e.g. FLAG-IDA) for remission/MRD negativity followed by allogeneic SCT if transplant candidate and donor available. VEN-AZA again is not primarily useful; If SCT is not possible, continue non-intensive or supportive therapy.

**Rationale:**
CBFB-MYH11-AML has significant curative potential through allo-SCT. Relapse after VEN-AZA/HiDAC requires salvage induction to achieve remission/MRD negativity and create transplant opportunity; alternative therapies offer lower long-term survival rates.

## ================================================== gpt-5-nano (agent) ==================================================

**===== run_1 =====**

**Tumor board decision:**
Oncogenic KIT p.D816V. Recommend KIT inhibition (e.g. dasatinib or ponatinib) as part of salvage therapy in AML-CBF-INV(16) after relapse to achieve remission and subsequent early allogeneic SCT. Clinical trials preferred; SCT after remission.

**Rationale:**

**Rationale: Horak 2022: KIT p.D816V Oncogenic (10). Javidan 2025: KIT mutations in CBF-AML are prognostically relevant; KIT inhibitors (e.g. dasatinib/ponatinib) as a therapeutic option. Briquilimab-KIT blockade as experimental conditioning. Conclusion: KIT inhibitors + salvage, SCT after remission, ideal in study.**

**===== run_2 =====**

**Tumor board decision:**
KIT-D816V positive: participation in KIT inhibitor study; Remission -> early allogeneic transplantation. If no studio visit: Salvage with KIT inhibitors + HMAs +/- Venetoclax as a bridge.

**Rationale:**

**Rationale: According to Horak 2022, KIT-D816V is oncogenic (10 points). CBFB-MYH11-FISH unremarkable. PubMed (2025) shows prognostic relevance of KIT-D816V in CBFB-AML and KIT inhibitors in studies. Recommendation: KIT inhibitor study as primary strategy; remission -> early allogeneic transplantation; No study access: Salvage with KIT inhibitor + HMAs/venetoclax.**

**===== run_3 =====**

**Tumor board decision:**
Oncogenic KIT D816V (Horak 2022; 10 points). KIT-targeted TKIs recommended in the salvage setting (e.g. dasatinib/sorafenib; if necessary avapritinib off-label) with renewed remission induction. In case of remission, early allogeneic transplantation or participation in a clinical trial is preferred.

**Rationale:**
According to Horak 2022, KIT D816V is oncogenic (10 points). CBFB-MYH11-based AML; PubMed suggests potential benefit of KIT-TKIs in relapse in CBF-AML; no robust RCTs. Briquilimab demonstrates KIT targeting in conditioning. Recommendation: Salvage with KIT-TKI +/- study; Allo-SCT after remission, if possible.

## ================================================== gpt-5-nano (plain) ==================================================

**===== run_1 =====**

**Tumor board decision:**
Recommendation: Salvage induction with FLAG-IDA (or equivalent) to achieve CR/MRD negative, followed by allogeneic SCT in a suitable donor. Start your donor search now; VEN-AZA not repeated before transplant; supportive care.

**Rationale:**
Molecular relapse (CBFB-MYH11) despite initial remission; Allo-SCT offers the highest long-term cure rate. Salvage before transplant increases remission rate and transplant chances; previous VEN-AZA showed only temporary control.

**===== run_2 =====**

**Tumor board decision:**
Salvage induction chemotherapy (e.g. FLAG-IDA or CLAG-M) for CR/MRD negativity, followed by allogeneic transplantation if transplant fitness and donor availability are appropriate. If not a candidate/clinical trial, consider alternative therapies.

**Rationale:**
CBFB-MYH11-AML is chemosensitive; Relapse according to VEN-AZA/HiDAC. Allo-SCT in the first relapse offers the highest healing potential. The aim is to prioritize CR/MRD negativity before transplant, donor testing and transplant fitness; If necessary, consider clinical studies.

**===== run_3 =====**

**Tumor board decision:**
Salvage induction with FLAG-IDA or CPX-351 for remission/MRD negativity followed by immediate allogeneic SCT if remission is sufficient; clinical trial preferred if available.

**Rationale:**
CBFB-MYH11-AML (inv(16)) with favorable ELN; Recurrence after venetoclax/AZA. The aim is a curative transplant. Reinduction makes sense to achieve MRD negative; early transplantation improves outcome; Consider comorbidities.

## ======================================================= gpt-oss-20b (agent) =======================================================

**===== run_1 =====**

**Tumor board decision:**
Allele KITp.D816V is Oncogenic. For relapsing CBF-AML (inv(16)), we recommend immediate allogeneic SCT (allo-HCT) as standard. Additionally, a KIT inhibitor (e.g. midostaurin or avapritinib) can be used before or after transplantation. If available, we are considering briquilimab as part of the conditioning regimen.

**Rationale:**
Horak-Score 10p.D816V -> Oncogenic. Article 1 shows worse prognosis for KIT-D816V; Article 2 describes briquilimab as a potential conditioning antigen. No other targeted mutations present. Allo-HCT remains the best option; KIT inhibitors can serve as complementary therapy.

**===== run_2 =====**

**Tumor board decision:**
Allogeneic SCT (allo-HCT) with briquilimab-based conditioning is recommended. KITD816V is oncogenic and increases relapse risk; no approved KIT inhibitors for AML. Check enrollment in KIT inhibitor study (e.g. avapritinib).

**Rationale:**
KITD816V Oncogenic (+10). Article 2 shows increased risk of relapse; Article 3 describes briquilimab as a potential conditioning agent. No other targeted therapy for KIT in AML; therefore allo-HCT with briquilimab is optimal.

**===== run_3 =====**

**Tumor board decision:**
Allogeneic SCT (allo-SCT) recommended as standard salvage. KITp.D816V is oncogenic and prognostically negative; There is no established targeted therapy. Consider participation in a clinical trial with KIT blockers (e.g. briquilimab) as a conditioning agent, if available.

**Rationale:**
Horak score 10 -> Oncogenic; KITp.D816V increases relapse risk (PMID40412578). No evidence-based targeted therapy approach in the articles. allo-SCT remains the best option in CBF-AML with KIT mutation; Study participation with briquilimab as non-myeloablative conditioning worth considering.

**==================================================== gpt-oss-20b (plain) =====================================================**

**===== run_1 =====**

**Tumor board decision:**
Further treatment with allogeneic SCT (allo-SCT) after appropriate mobilization and donor search. If an immediate transplant donor is not available, consider using gemtuzumab ozogamicin in combination with HiDAC as salvage therapy.

**Rationale:**
The patient with inv(16) AML lost hematologic remission after multiple cycles of azacitidine/venetoclax and HiDAC. For CBF-AML, allo-SCT is the only curative option after remission. Gemtuzumab ozogamicin can be used as salvage therapy in the absence of a transplant donor.

**===== run_2 =====**

**Tumor board decision:**
Salvage therapy with CPX-351 (Dara-Cyt) followed by allogeneic SCT (if a suitable donor is available).

**Rationale:**
The patient has shown molecular and hematological recurrence in AML-inv(16) after azacitidine/venetoclax and HiDAC. CPX-351 is the evidence-based salvage option for secondary AML, enabling remission, serving as a bridge to allo-HSCT. If there is no donor, a clinical trial with FLT3/IDH inhibitors or a new azacitidine/venetoclax regimen should be considered.

**===== run_3 =====**

**Tumor board decision:**
Initiate allogeneic SCT (allo-SCT) after intensive bridging therapy (e.g. FLAG-IDA) if a suitable donor is available. If there is no transplant option, consider alternative intensive chemotherapy (CLAG-ID) followed by renewed HMA/venetoclax therapy.

**Rationale:**
The patient has demonstrated molecular and hematological recurrence in AML-inv(16) after multiple cycles of azacitidine/venetoclax and HiDAC. For favorite-risk AML, allo-SCT is the only therapy with potential remission in the event of relapse. Intensive bridging increases the remission rate before transplantation.

**==================================================== gpt-oss-120b (agent) ====================================================**

**===== run_1 =====**

**Tumor board decision:**
Recommendation: Intensified re-induction with high-dose cytarabine + anthracycline and addition of a KIT inhibitor (e.g. midostaurin 50 mg bid or avapritinib according to the study protocol). If CR is achieved, allogeneic SCT should be performed. Alternatively, participation in a KIT-directed study (e.g. briquilimab conditioning).

**Rationale:**
KIT p.D816V is based on Horak et al. Classified as Oncogenic (+10) in 2022. In CBF-AML, this mutation is a poor prognostic factor. PubMed articles (PMID40412578,38972509) show the benefits of KIT inhibitors or KIT blockade. Therefore, targeted KIT therapy together with Allo-SCT is evidence-based.

**===== run_2 =====**

**Tumor board decision:**
If the KIT D816V mutation (oncogenic) is proven, KIT inhibitor therapy is recommended: add midostaurin (or avapritinib) to intensive chemotherapy/consolidation and plan an allogeneic SCT in the first remission status. Alternatively, participation in a study with briquilimab-based conditioning.

**Rationale:**
KITp.D816V receives Horaketal. 2022 the highest classification (+10 points). PMID40412578 demonstrates poor prognostic factor and benefit of KIT inhibitors; PMID38972509 describes briquilimab as a non-myeloablative conditioning approach for KIT-mutated AML. Therefore, targeted KIT blockade plus allo-SCT is recommended.

**===== run_3 =====**

**Tumor board decision:**
If the oncogenic KIT-D816V mutation is present, KIT-inhibiting therapy is recommended: midostaurin 50 mg bid (or avapritinib) in combination with intensive salvage chemotherapy, followed by allogeneic SCT. Alternatively, participation in a clinical study with a KIT inhibitor or briquilimab-based conditioning can be considered.

**Rationale:**
KIT p.D816V receives according to Horak et al. 2022 10Points -> Oncogenic. Literature (Javidan2025) shows poorer prognosis and benefits of KIT inhibitors; Briquilimab study (Jung2024) offers non-myeloablative conditioning for KIT-mutated AML. Therefore, targeted KIT blockade combined with allo-SCT is recommended.

## ================================================== gpt-oss-120b (plain) ==================================================

**===== run_1 =====**

**Tumor board decision:**
We recommend intensive reinduction (e.g. FLAG-IDA or CLAG-M) to achieve a second CR, followed by an allogeneic SCT from a suitable HLA-compatible donor, if performance (blood pressure, diabetes) allows this.

**Rationale:**
The patient has CBF-AML (inv(16)) with favorable risk classification, but a hematological relapse after multiple therapy with azacitidine+venetoclax and HiDAC. Without allogeneic transplantation, the long-term survival rate is low. Intensive reinduction enables MRD negativity and is a prerequisite for transplantation.

**===== run_2 =====**

**Tumor board decision:**
Start intensive salvage therapy (e.g. FLAG-IDA or CLAG-M) to achieve a second remission, followed by an allogeneic SCT if a suitable donor is available.

**Rationale:**
The patient has shown hematological recurrence after HMA+venetoclax and HiDAC; the previous therapy is now refractory. In favorable karyotype AML, an allo-SCT after CR2 is the only curative option, which is made possible by intensive reinduction.

**===== run_3 =====**

**Tumor board decision:**
Salvage therapy with CPX-351 (5-day regimen) as bridging therapy, followed by allogeneic SCT, if age/comorbidities allow this; if not transplantable, clinical trial or low-intensity therapy (e.g. glasdegib+azacitidine).

**Rationale:**
Patient has therapy-related AML with inv(16) after failure of azacitidine/venetoclax and HiDAC. CPX-351 is approved for secondary AML, achieves high remission rates and serves as a bridge to alloHSCT, which is the only curative target if the karyotype is favorable. HTN and diabetes are not absolute contraindications.

## ==================================================== qwen3-32b (agent) ====================================================

**===== run_1 =====**

**Tumor board decision:**
Recommendation of a consolidation strategy with briquilimab (c-Kit inhibitor) in a clinical study (PMID:38972509) and evaluation of an allogeneic SCT under KIT-targeted therapy.

**Rationale:**
KIT p.D816V is oncogenic (10 Horak points) and associated with recurrence prognosis in CBF-AML (PMID:40412578). Briquilimab (Phase I/II study) addresses KIT-dependent disease. Combination with azacitidine/venetoclax and HSCT preparation is discussed.

**===== run_2 =====**

**Tumor board decision:**
Recommendation: Briquilimab (c-Kit inhibitor) in clinical trial (PMID:38972509) in KIT p.D816V-positive CBF-AML. Prioritize consolidation with allo-HCT. Azacitidine/venetoclax as maintenance under resistance monitoring.

**Rationale:**
KIT p.D816V is oncogenic (10 Horak points) and prognostically relevant in CBF-AML (PMID:40412578). Briquilimab (Phase I/II) addresses KIT dependence. Allo-HCT remains standard with a favorable initial risk. Maintenance therapy with azacitidine/venetoclax taking resistance mechanisms into account.

**===== run_3 =====**

**Tumor board decision:**
Recommendation of combination therapy with briquilimab (c-Kit inhibitor) and azacitidine/venetoclax for KIT p.D816V-mutant CBF-AML. Consider consolidative SCT (SCT).

**Rationale:**
KIT p.D816V is according to Horak et al. Classified as Oncogenic in 2022 (10 points). PubMed evidence shows briquilimab as a c-Kit inhibitor in conditioning for SCT in KIT-mutant AML. Combination with azacitidine/venetoclax (pretreated responder) and consolidative SCT with favorable initial risk.

## ===================================================== qwen3-32b (plain) =====================================================

**===== run_1 =====**

**Tumor board decision:**
Recommendation: Allogeneic SCT (SCT) as consolidative therapy after renewed remission induction with HiDAC. In non-transplantable condition: Clinical trial with targeted therapies (e.g. CBFB-MYH11 inhibitors) or intensive chemotherapy.

**Rationale:**
Inv(16)-AML has favorable prognostic potential, but relapses after azacitidine/venetoclax and HiDAC. Allogeneic SCT is standard for recurrent AML with favorable genotype. If transplantation is not possible, experimental approaches or more intensive chemotherapy remain alternatives.

**===== run_2 =====**

**Tumor board decision:**
Recommendation: Allogeneic SCT (SCT) as consolidative therapy. If not eligible for transplantation: Participation in a clinical study with CBFB-MYH11-specific targeted therapies.

**Rationale:**
Inv(16)/CBFB-MYH11-AML is one of the ELN-favorable subtypes. After induction of remission with azacitidine/venetoclax and relapse after HiDAC, SCT is the standard consolidation. Participation in a study is an alternative if there are cardiometabolic risks (DM, HT).

**===== run_3 =====**

**Tumor board decision:**
Recommendation: Allogeneic stem cell transplantation (allo-SCT) after re-induction of remission with HiDAC or alternative combination therapy (e.g. venetoclax + idarubicin).

**Rationale:**
The patient presents with recurrent AML with CBFB-MYH11 fusion despite azacitidine/venetoclax. HiDAC after molecular recurrence detection proved to be inadequate. Allo-SCT remains the only curative option with a favorable risk profile and tolerable comorbidities.

## ================================================== qwen3-next-80b (agent) ==================================================

**===== run_1 =====**

**Tumor board decision:**
Recommendation: Allogeneic SCT (allo-SCT) with briquilimab-based conditioning regimen (clinical study) or alternatively midostaurin-assisted therapy. Priority study inclusion in clinical trials of c-Kit inhibitors (e.g. briquilimab, midostaurin) vorallo-SCT. Standardized allo-SCT after achieving remission.

**Rationale:**

KIT p.D816V is Oncogenic (10 points, Horak 2022). Studies show KIT mutations as prognostically unfavorable in CBF-AML (PMID:40412578). Briquilimab blocks c-Kit for HSCT conditioning (PMID:38972509). Midostaurin has data in KIT-mutated AML. Allo-SCT is standard for relapsed AML with KIT mutation. Prioritize study inclusion.

**===== run_2 =====**

**Tumor board decision:**
Allogeneic stem cell transplantation with briquilimab-based conditioning recommended in clinical trial. KIT p.D816V is oncogenic (Horak 10 points), briquilimab blocks c-Kit receptor (PMID:38972509).

**Rationale:**
KIT p.D816V is oncogenic with high evidence. Briquilimab is a targeted KIT inhibitor in clinical development for AML with KIT mutations, specifically for conditioning before HSCT. Study shows potential efficacy in KIT-mutated AML (PMID:38972509).

**===== run_3 =====**

**Tumor board decision:**
Recommend allogeneic SCT with briquilimab-based conditioning in a clinical trial. KIT p.D816V is relevant to therapy and can be addressed with briquilimab (anti-c-Kit) as a conditioning agent.

**Rationale:**
KIT p.D816V is Likely Oncogenic (6 points). Study (PMID:38972509) evaluates briquilimab as a non-myeloablative conditioning agent for HSCT in KIT-mutated AML. Blocks c-Kit receptor, improves engraftment and reduces relapse risk in CBF-AML with KIT mutation.

## ================================================ qwen3-next-80b (plain) ================================================

**===== run_1 =====**

**Tumor board decision:**
Re-induction with intensive chemotherapy (7+3 regimen: cytarabine + daunorubicin), followed by allo-stem cell transplantation when remission is achieved.

**Rationale:**
Patient has relapse after azacitidine/venetoclax and HiDAC. Inv(16)-AML is ELN favorable, but therapeutic success in relapse requires intensive re-induction. 7+3 is standard for fit patients. Comorbidities (HTA, DM II) are treatable. Allo-SCT is a targeted curative option after remission.

**===== run_2 =====**

**Tumor board decision:**
CPX-351 as salvage therapy followed by allostemmal cell transplantation in remission

**Rationale:**
AML-pCT (t-AML) with inv(16) is favorable risk, but after HiDAC recurrence. CPX-351 is approved for t-AML and should be used as a salvage. If there is remission, allo-SCT is indicated. Hypertension/DM are not contraindications.

**===== run_3 =====**

**Tumor board decision:**
Reinduction with gemtuzumab-ozogamicin (GO) in combination with high-dose cytarabine (e.g. FLAG-IDA regimen) followed by allogeneic SCT (allo-SCT) if remission is achieved.

**Rationale:**
Relapse after azacitidine/venetoclax and HiDAC. Inv(16) is CBF-AML with CD33 positivity. GO improves remission rates in CBF-AML. Allo-SCT is standard for relapse CBF-AML after remission. Hypertension/DM are not contraindications to intensive therapy if you are in good physical condition.

# l_case_10__ALL

################################
#### CASE: 10
################################

## ==================================================== gpt-5-mini (agent) ====================================================

**===== run_1 =====**

**Tumor board decision:**
Within 48-72h: Repeat PB/BM qPCR, BM aspirate/biopsy with FACS, FISH/karyotype, BCR::ABL-KD mutation analysis and extended NGS. For T315I -> ponatinib + rapid allo-SCT evaluation (high priority due to monosomy 7). In case of further molecular progression without T315I -> switch to more potent TKI (e.g. ponatinib) or intensification +/- blinatumomab/inotuzumab; KRAS p.G12D: no TKI sensitivity - review MEK/MAPK inhibitor studies or trial inclusion.

**Rationale:**
KRAS p.G12D is classified as an oncogenic (+10) and signals MAPK activation (literature: PMID29997224, PMID28408464) -> rationale for MEK/MAPK inhibitors in studies. Increasing BCR::ABL1 indicates TKI resistance: KD mutation test decides therapy (T315I -> ponatinib). Monosomy 7 = high-risk of recurrence -> rapid allo-SCT planning.

**===== run_2 =====**

**Tumor board decision:**
Immediate diagnostics (within 48-72 h): repeated PB/BM qPCR, BM aspirate/biopsy with FACS, cytogenetics/FISH, BCR::ABL kinase domain mutation analysis and, if necessary, extended NGS. Therapeutic: in case of confirmed molecular progression/ABL-KD mutation, TKI switch to ponatinib and rapid allogeneic SCT (KSCT) evaluation for monosomy 7. KRAS p.G12D is oncogenic (not an approved direct target therapy) - inclusion in clinical studies with MEK/ERK inhibitors or evaluation of CAR-T/alternative immunotherapies recommended.

**Rationale:**
KRAS p.G12D classified as oncogenic (Horak et al. +10). Literature (PMID:29997224,28408464) shows KRAS-driven MAPK activation in (Ph-like) B-ALL; no established targeted approval - MEK/ERK inhibitors only in a study context. Rising BCR::ABL1 requires rapid KD testing; Monosomy 7 increases KSCT indication.

**===== run_3 =====**

**Tumor board decision:**
Immediate diagnostics: repeat PB/BM qPCR, BM aspirate/biopsy with FACS, cytogenetics/FISH, urgent BCR-ABL kinase domain mutation analysis and extended NGS. Therapy: close monitoring until the mutation is confirmed; If resistant BCR-ABL mutations (e.g. T315I) are detected, consider switching the TKI to ponatinib; rapid evaluation for allogeneic SCT because of monosomy 7. KRAS p.G12D (oncogenic) cannot currently be treated directly-inclusion in studies on RAS/MEK inhibitors is recommended.

**Rationale:**
KRAS p.G12D classified as oncogenic (Horak et al. +10). Literature shows activation of the RAS pathway but no established targeted therapy in BCR::ABL1-ALL; Studies on RAS/MEK inhibitors recommended. BCR-ABL1 status remains clinically relevant for action: mutation detection controls TKI switching; Monosomy 7 increases risk and indication for transplantation.

## ==================================================== gpt-5-mini (plain) ====================================================

**===== run_1 =====**

**Tumor board decision:**
Allo-SCT (reduced condition) with suitable donor as primary option. Continue TKI bridge (e.g. ponatinib) until transplant; Monitor MRD/CR closely. If no donor promptly, check CAR-T.

**Rationale:**
Ph+ALL with progression/relapse risk despite blinatumomab/TKI; Allo-SCT offers a curative opportunity. TKI bridge stabilizes remission; Check donor availability and comorbidities.

===== run_2 =====

**Tumor board decision:**
Recommendation: Switch to ponatinib 45 mg/d (dose reduction if necessary if there are vascular risk factors) with blinatumomab bridge; Goal: deep MRD/negativity and subsequent allogeneic SCT if transplantable.

**Rationale:**
Molecular relapse (BCR-ABL approx. 0.145%) despite long-term TKI/blinatumomab. Ponatinib increases MRD release; Blinatumomab may support MRD achievement. allo-SCT remains curative, provided the patient is capable of treatment.

===== run_3 =====

**Tumor board decision:**
Allo-SCT in CR1 after donor search. Aim for MRD negativity before transplant; Consider reduced conditioning regimen (RIC) for COPD/DM. Continue TKIs peri/posttransplant (e.g. bosutinib); alternatively CAR-T if you are not a candidate.

**Rationale:**
high-risk BCR-ABL+ ALL (t(9;22), -7); Multiple lines of therapy; MRD positive (BCR-ABL approx. 0.145%) despite CR; Relapse risk high. Allo-SCT offers curative opportunity; Comorbidities suggest the most moderate fitness regime possible.

## ==================================================== gpt-5-nano (agent) ====================================================

===== run_1 =====

**Tumor board decision:**
Participation in a clinical trial with MEK inhibitors (e.g. trametinib) in Ras/MAPK-activated Ph-like ALL (KRAS G12D) in combination. with current ALL therapy; Level of Evidence: Phase II; Target MRD negativity. If study access is not available, consider allo-SCT in CR1.

**Rationale:**
KRAS p.G12D is oncogenic (Horak 2022). Ph-like ALL shows Ras/MAPK activation; direct KRAS inhibition is limited. MEK inhibitors in clinical trials are a rational but not yet established option; TKIs address kinase signals that make KRAS G12D difficult to target directly. MRD positive despite therapy; Consider grafting into CR1 at access.

===== run_2 =====

**Tumor board decision:**
Oncogenic KRAS p.G12D. Recommendation: clinical trial with KRAS/MAPK inhibitors (e.g. G12D-specific KRAS inhibitor or MEK inhibitors); no approved KRAS-targeted therapy in ALL. Goal: MRD reduction; If no trial is available, consider other MRD-driven options (CAR-T, Allo-Transplant) based on MRD progression.

**Rationale:**
According to Horak 2022, KRAS p.G12D is oncogenic (10 points); FISH negative. PubMed reference treats Ph-like ALL, does not provide KRAS-specific therapy. No established targeted option; Recommendation based on oncogenic status with KRAS/MAPK clinical trial options; Aim to reduce MRD instead of premature transplantation.

===== run_3 =====

**Tumor board decision:**
Aim for allogeneic transplantation in first remission (CR1); MRD persists (BCR-ABL1 qPCR 0.1-0.2%) despite blinatumomab+TKI. KRAS p.G12D = Likely Oncogenic; no established KRAS-specific therapy. CAR T-cell therapy as a bridge if available.

**Rationale:**
Horak 2022: KRAS p.G12D as Likely Oncogenic (+6 points). FISH negative. PubMed shows targeted therapies for Ph-like ALL (ABL/JAK inhibitors), but no established KRAS-specific approach. In high-risk Ph-like ALL with MRD positivity in CR1, allo-SCT is priority; CAR-T makes sense as a bridge if available.

## ==================================================== gpt-5-nano (plain) ====================================================

===== run_1 =====

**Tumor board decision:**
Recommendation: TK switch to ponatinib (e.g. 45 mg/d) if progression under bosutinib is suspected; Bridging therapy with blinatumomab possible; timely preparation for allogeneic SCT with sufficient remission/MRD negativity; alternatively check CAR-T.

**Rationale:**
Progression under current TKI; Ponatinib offers the highest activity against BCR-ABL, possibly avoiding resistance. Allo-SCT is potentially curative after CR/MRD negativity. Blinatumomab as a bridge can reduce MRD; CAR-T as an option depending on availability.

===== run_2 =====

**Tumor board decision:**
CAR-T cell therapy (CD19) as the next salvage treatment for progression on blinatumomab/TKI. If CAR-T is not possible in a timely manner: inotuzumab ozogamicin as a bridge therapy. Goal: MRD-negative remission and subsequent allogeneic transplantation, if donor is available and organ status is suitable.

**Rationale:**
Ph+ ALL shows progression despite prolonged blinatumomab/TKI therapy. CAR-T offers the highest probability of long-lasting remission and enables subsequent curative allo-transplantation. InO serves as a useful bridge in case of delays; Check donor location and organ status."

===== run_3 =====

**Tumor board decision:**
CAR T-cell therapy against CD19 (brexucabtagene autoleucel) as the next step in the progression of BCR::ABL+ ALL; Check CD19 expression. Bridging: Ponatinib 45 mg/d or blinatumomab as tolerated. After remission/allogeneic state: allogeneic SCT should be considered.

**Rationale:**
Progression despite long-term TKIs/blinatumomab. CAR T-cell therapy offers good remission rates in R/R CD19+ ALL; potential for healing; Transplantation makes sense as a curative follow-up after CAR-T or remission, taking comorbidities into account.

## ================================================= gpt-oss-20b (agent) =================================================

===== run_1 =====

**Tumor board decision:**
For oncogenic KRAS p.G12D and BCR::ABL1-positive Ph-ALL, we recommend: 1) continuation of TKI therapy (ponatinib, 3rd generation) because of BCR::ABL1 activity; 2) inclusion in a clinical study with a MEK inhibitor (e.g. trametinib) or KRAS-G12D-specific agent (e.g. MRTX1133) for the targeted blockade of the KRAS signaling pathways; 3) After achieving CR/CRi and MRD negativity, we plan an allo-SCT, alternatively CAR T-cell therapy (CD19-targeted) if progression occurs again.

**Rationale:**
According to Horak-SOP Oncogenic (+10 points) and in Ph-like ALL, KRAS p.G12D is associated with poor prognosis. PubMed articles 29997224 and 28408464 show MEK/KRAS inhibitor potential. BCR::ABL1 positive means TKI therapy; Ponatinib has the highest effectiveness against T315I resistance. Allo-SCT remains standard in Ph-ALL after remission. CAR-T as a reserve in case of progression.

===== run_2 =====

**Tumor board decision:**
KRAS p.G12D is oncogenic. Recommended: Participation in a clinical trial with KRAS-G12D inhibitor (e.g. sotorasib/adagrasib) or combination MEK/PI3K blockade (e.g. trametinib + alpelisib). Parallel BCR-ABL1 targeting with ponatinib (if not already used) and, if necessary, allo-SCT in case of progression.

**Rationale:**
Horak score 10 -> Oncogenic. PubMed articles show KRAS G12D inhibitors in phase I/II studies; no ALL data, hence study inclusion. BCR-ABL1-positive remains therapeutically relevant; Ponatinib has the best evidence for TKI resistance. Allo-SCT remains standard in cases of progression despite MRD negativity.

**===== run_3 =====**

**Tumor board decision:**
1. Allogeneic SCT (allo-SCT) after achieving CR with BCR::ABL1-positive status. 2. Bridging therapy with ponatinib (3rd gene TK inhibitor, phase II/III, evidence A). 3. In case of progression after allo-SCT or renewed MRD positivity, CD19-CAR-T-cell therapy in clinical study (PhaseI/II).

**Rationale:**
KRASp.G12D is oncogenic according to Horak+10, but cannot be directly targeted. BCR::ABL1-positive remains a therapeutic target. Ponatinib shows efficacy in Ph-like/ BCR::ABL1-positive ALL (Phase II/III). CD19-CAR-T is evidence-based in refractory ALL (Phase I/II).

## ================================================== gpt-oss-20b (plain) ==================================================

**===== run_1 =====**

**Tumor board decision:**
Start ponatinib 45mgqd (titrated) plus blinatumomab 28-day cycle as bridging therapy to allogeneic stem-cell transplantation. If the remission is positive, transplant after 2-3 cycles, otherwise consider alternative intensive chemotherapy.

**Rationale:**
Patient shows progression despite imatinib, dasatinib and bosutinib. Ponatinib, a 3rd gene TKI, has high activity in TKI-resistant BCR::ABL1-ALL. Combination with blinatumomab delivers rapid MRD reduction and serves as effective bridging to allo-SCT, despite COPD, HTN and DM-II.

**===== run_2 =====**

**Tumor board decision:**
We recommend switching the patient to ponatinib 30 mg daily (or 15 mg if comorbidities) as the next TKI. Concurrently, assess eligibility for allogeneic stem-cell transplantation within 3 months. Continue blinatumomab if clinically indicated.

**Rationale:**
The patient shows rising BCR::ABL1 levels after failure of dasatinib, imatinib and bosutinib, indicating TKI resistance. Ponatinib has proven efficacy in BCR::ABL1-positive ALL with TKI failure and can be combined with allo-SCT, which offers the best long-term cure potential in this setting.

**===== run_3 =====**

**Tumor board decision:**
Start ponatinib 45 mg daily (after 3rd generation TKI) with monitoring of cardiovascular risks; parallel evaluation for allogeneic SCT. If there is an inadequate response within 4-6 weeks, consider inotuzumab-ozogamicin or clinical trials.

**Rationale:**
Patient showed progression despite imatinib, dasatinib and bosutinib. Ponatinib has the highest effectiveness in BCR::ABL1-positive ALL and can be used in cases of resistance to 2nd generation TKIs. Comorbidities (COPD, HTN, DM) require careful monitoring, but transplantation remains a long-term option with good performance status.

## ================================================== gpt-oss-120b (agent) ==================================================

**===== run_1 =====**

**Tumor board decision:**
If KRASp.G12D (oncogenic) is proven, targeted MAPK inhibitor therapy should be carried out in a study context (e.g. trametinib or selumetinib, possibly combined with the current BCR-ABL-TKI). At the same time, the possibility of an allogeneic SCT should be examined.

**Rationale:**
KRASG12D scores 10 points after Horaketal., thus Oncogenic. PubMed articles (PMID29997224,28408464) demonstrate activated RAS MAPK signaling in Ph-like ALL and potential benefit of MEK inhibitors. Since there is no approved KRAS-G12D inhibitor, study or off-label use is recommended.

**===== run_2 =====**

**Tumor board decision:**

KRAS p.G12D is oncogenic (10 points). Since there is no approved KRAS G12D therapy for B-ALL, we recommend participation in a clinical trial with MEK inhibitors (e.g. trametinib) or combined KRAS downstream blockers. In parallel, the current BCR-ABL-TKI therapy should be continued and, if progression continues, allogeneic SCT should be evaluated.

**Rationale:**
Horak et al. In 2022, KRAS classified p.G12D as an oncogenic. PubMed articles (PMID29997224,28408464) show KRAS activation in Ph-like ALL and suggest potential effectiveness of MEK inhibitors or KRAS downstream studies. No approved options, therefore study recommendation.

**===== run_3 =====**

**Tumor board decision:**
Due to the oncogenic KRAS G12D mutation (Oncogenic, +10), targeted MAPK blockade should be considered. Participation in a clinical trial with a MEK inhibitor (e.g. trametinib or selumetinib) in combination with the current therapy regimen and, if clinically appropriate, allogeneic SCT as a potential curative option is recommended.

**Rationale:**
KRAS-G12D scores 10 points (hotspot, functional evidence, rare population) and is according to Horak et al. oncogenic. PubMed articles (PMID29997224, 28408464) show activated MAPK signaling pathways and suggest MEK/ERK inhibitors or experimental KRAS G12D inhibitors (PhaseI/II). No approved KRAS G12D inhibitor therapy, therefore study participation.

## ================================================== gpt-oss-120b (plain) ==================================================

**===== run_1 =====**

**Tumor board decision:**
If progression is suspected, immediate bone marrow biopsy including BCR-ABL mutation analysis. If a relapse is confirmed, immediate salvage therapy with ponatinib (30 mg/d) + reinduction chemo (e.g. mini-hyper-CVD) or blinatumomab, aiming for MRD negativity and transfer to allogeneic SCT if donor available. Without a transplant option, check CAR-T (CD19).

**Rationale:**
The patient showed persistent BCR-ABL transcripts and clinical progression despite imatinib/dasatinib/bosutinib and blinatumomab. Ponatinib overcomes most ABL mutations and is effective in Ph ALL relapses. Combined with reinduction, MRD negativity can be achieved quickly, which is a prerequisite for allo-SCT - the only curative option. CAR-T is an alternative if transplantation is not possible.

**===== run_2 =====**

**Tumor board decision:**
If there is clinical suspicion of progression, immediately carry out a BCR-ABL kinase domain mutation analysis and, if no T315I or, if available, start ponatinib (30 mg/day, if necessary 15 mg if there is a cardiovascular risk) as a rescue TKI. Parallel evaluation for allo-SCT (after achieving a second molecular remission) taking COPD, HTN and diabetes into account.

**Rationale:**
The patient has an increasing BCR-ABL transcript count despite multiple TKIs (imatinib, dasatinib, bosutinib) and blinatumomab, indicating a resistance or progression event. Ponatinib overcomes most kinase domain mutations and is the standard rescue TKI. Allo-SCT remains the only curative option for B-ALL with high-risk karyotype (monosomy7).

**===== run_3 =====**

**Tumor board decision:**
If progression is confirmed (BM biopsy, MRD, BCR-ABL mutation profile), immediate ponatinib-based reinduction (ponatinib 30 mg/d + mini-hyper-CVAD) and, if transplantable, rapid allogeneic SCT.

**Rationale:**
The patient has new evidence of disease after multiple TKIs (imatinib, dasatinib, bosutinib) and blinatumomab. Ponatinib is effective against most BCR-ABL mutations and offers the best chance of achieving remission; Allotransplantation remains the curative option for Ph-positive ALL.

## ================================================== qwen3-32b (agent) ==================================================

===== run_1 =====

**Tumor board decision:**
Recommendation for clinical studies with KRAS-targeted therapy (e.g. MEK inhibitors) or Ph-like ALL-specific kinase inhibitors (evidence level 2B).

**Rationale:**
KRAS p.G12D is classified as oncogenic (10 points). PubMed evidence supports tyrosine kinase inhibitors in Ph-like ALL. Monosomy 7 and BCR::ABL1 resistance to established TKIs (imatinib/dasatinib/bosutinib) warrant new approaches.

===== run_2 =====

**Tumor board decision:**
Recommendation for study inclusion in clinical trials with MEK inhibitors or combination therapies in KRAS-p.G12D-positive Ph-like ALL. Continued use of ponatinib as a final TKI option due to resistance to previous TKIs.

**Rationale:**
KRAS-p.G12D is classified as oncogenic (10 points) and is associated with Ph-like ALL, in which TKIs are discussed. Despite MRD negativity and low BCR::ABL levels, monosomy 7 suggests resistance. Standard TKIs (imatinib/dasatinib/bosutinib) failed. Ponatinib and MEK inhibitors (e.g. trametinib) are being investigated in clinical trials for Ph-like ALL with KRAS mutations. Study inclusion is urgent due to the oncogenic KRAS variant and complex clonality.

===== run_3 =====

**Tumor board decision:**
Recommendation of MEK inhibitor treatment (e.g. trametinib) in combination with tyrosine kinase inhibitor (TKI) resistance bypass therapy. Study inclusion in clinical trials for KRAS-targeted therapy (PMID:29997224, 28408464) due to the highly oncogenic KRAS p.G12D variant and clonal monosomy 7.

**Rationale:**
KRAS p.G12D (Oncogenic, 10 points) is associated with TKI resistance in Ph-like ALL. Monosomy 7 worsens prognosis. MEK inhibitors and combination therapies are described in the literature as targeted. Study participation recommended due to VUS score >=4 and lack of standardized KRAS therapies.

## ================================================== qwen3-32b (plain) ==================================================

===== run_1 =====

**Tumor board decision:**
Recommendation: Therapy with ponatinib 45 mg/d and evaluation for allogeneic SCT (SCT) if a donor and fitness are available. Continue blinatumomab every 12 weeks for MRD reduction.

**Rationale:**
Progression on multiple TKIs (imatinib, dasatinib, bosutinib) despite low MRD. Ponatinib as a third generation TKI with activity against BCR::ABL1 and resistance mutations. Blinatumomab shows MRD reduction. SCT remains an option in long-term remission.

===== run_2 =====

**Tumor board decision:**
Recommendation: Allogeneic stem cell transplantation (SCT) after intensive conditioning therapy. Alternatively: Clinical trial with inotuzumab ozogamicin + blinatumomab + TKI (e.g. ponatinib).

**Rationale:**
Patient with Ph+ ALL in morphologic CR but persistent MRD (0.145%). Previous TKIs (imatinib, dasatinib, bosutinib) did not show any MRD reduction. Allogeneic SCT is the gold standard for relapse-risk Ph+ ALL. Comorbidities (COPD, DM) require preconditioning risk stratification. Alternative therapy: Targeted therapy combination with innovative active ingredients.

===== run_3 =====

**Tumor board decision:**
Recommendation: CAR T-cell therapy (e.g. tisagenlecleucel) after intensive conditioning. Alternatively: combination therapy with blinatumomab + ponatinib if the medical history is tolerable.

**Rationale:**
Patient shows MRD progression despite sequential TKI and blinatumomab therapy. CAR-T cells are established in relapsed B-ALL with CD19 expression. Ponatinib could overcome BCR::ABL1 resistance mechanisms. Comorbidities require preconditioning-related risk limitation.

## ================================================ qwen3-next-80b (agent) ================================================

**===== run_1 =====**

**Tumor board decision:**
KRAS p.G12D oncogenic, but not an established targeted therapy in ALL. Recommendation: Check BCR::ABL1 resistance mutations; Study inclusion in MEK inhibitor studies (e.g. trametinib) in combination with TKI.

**Rationale:**
KRAS G12D according to Horak 2022 oncogenic (10 points). PubMed evidence shows no established therapy, but evidence of MEK inhibitors in studies (PMID:29997224, 28408464). BCR::ABL1 resistance must be excluded as a priority as this is primarily relevant for therapy adjustment.

**===== run_2 =====**

**Tumor board decision:**
Switch to ponatinib (evidence level 2B) for BCR::ABL1 resistance. KRAS p.G12D oncogenic, but not directly targetable; Recommend inclusion of MEK inhibitors (e.g. trametinib) in B-ALL studies.

**Rationale:**
KRAS p.G12D (10 points, Oncogenic) according to Horak. PubMed (PMID:29997224) shows limited evidence for MEK inhibitors in B-ALL. BCR::ABL1 resistance requires ponatinib switching as standard.

**===== run_3 =====**

**Tumor board decision:**
KRAS p.G12D oncogenic. Keine zugelassenen Therapien. Studieneinschluss in MEK-Inhibitor-Studien (z.B. Trametinib) empfohlen. Evidenzlevel 2b.

**Rationale:**
Horak classification: Oncogenic (10 points). PubMed evidence (PMID 29997224, 28408464) shows MEK inhibitors as a therapeutic option in studies in Ph-like ALL with RAS mutations. BCR::ABL1 positive with KRAS mutation may cause resistance, hence study inclusion.

## ================================================ qwen3-next-80b (plain) ================================================

**===== run_1 =====**

**Tumor board decision:**
Transition to ponatinib 45 mg daily with close cardiovascular monitoring and immediate evaluation for allogeneic SCT.

**Rationale:**
Progression under bosutinib with increasing BCR::ABL (0.145% vs. previously <0.01%). Ponatinib is standard in TKI-resistant Ph+ ALL, especially with T315I mutation (not tested, but necessary). Comorbidities (AHT, COPD) require risk management, but continued therapy is urgently needed. Transplantation is a targeted curative option.

**===== run_2 =====**

**Tumor board decision:**
Confirm progression by bone marrow aspirate with mutation test (T315I). If confirmed: start ponatinib 45 mg/d. Consider allo-SCT, but with increased risk due to COPD, AHT, DM II.

**Rationale:**
Progression to multiple TKIs (imatinib, dasatinib, bosutinib). Ponatinib effective against T315I mutations and resistance. Allo-SCT is standard in Ph+ ALL, but comorbidities significantly increase transplant risk.

**===== run_3 =====**

**Tumor board decision:**

Switch to ponatinib 30 mg daily with close monitoring of cardiovascular parameters and comorbidities. Additionally carry out mutation test for T315I.

**Rationale:**
Rising BCR::ABL on bosutinib (0.145% vs. 0.011% 4 months ago) suggests TKI resistance. Ponatinib is standard for resistance to 2nd generation TKIs. T315I test clarifies need. Comorbidities require dosage adjustment and close monitoring.

# l_case_11__ALL

################################
#### CASE: 11
################################

## ========================================================== gpt-5-mini (agent) ==========================================================

**===== run_1 =====**

**Tumor board decision:**
Recommendation: No immediate systemic therapy. First additional diagnostics: serum EPO, ferritin/TSAT, reticulocytes, complete SOP laboratory; if initial NGS panel is not IPSS-M compliant -> extended NGS (31 genes). If the disease is asymptomatic and does not require transfusion: watch and wait with KMP every 6-12 months (KM checks annually). If anemia is symptomatic or requires transfusion and sEPO <200 U/l or low transfusion load (<=2 EC/8 weeks): start ESA (Epo 40,000 IU/W or darbepoetin); if response is inadequate, add G-CSF. Luspatercept only for SF3B1/ring sideroblasts and anemia requiring transfusion or ESA failure; Lenalidomide only in isolated del(5q); Immunosuppressive therapy only if hypoplastic MDS is documented and the corresponding criteria are met. Allo-SCT only in cases of progression/restratification.

**Rationale:**

**Rationale: Patient is IPSS-M/IPSS-R low, CM blasts 4% and unremarkable cytogenetics/molecular genetics -> Guideline recommends observation for asymptomatic low-risk patients. The indication for therapy in anemia depends on the need for transfusion and sEPO. Additional diagnostics clarify the prognosis (IPSS-M) and treatment selection (SF3B1, del(5q), hypoplastic).**

**===== run_2 =====**

**Tumor board decision:**

**Tumor board decision: For IPSS-M/IPSS-R low, 4% BM blasts, normal karyotype and normal NGS, we primarily recommend diagnostic supplementation (sEPO, ferritin+TSAT, reticulocytes/Ret-Hb, if necessary parvo serology). Without severe symptoms/transfusion requirement: Watch & wait with BB checks every 6-12 months and annual CM reassessment (cytogenetics/NGS). For symptomatic anemia or anemia requiring transfusion and sEPO <200 U/l: start ESA (Epo 40,000 IU/W or darbepoetin) +/- G-CSF. Luspatercept only for SF3B1/ring sideroblasts and requires transfusion; Lenalidomide only in isolated del(5q); ATG+CyA only with clear hypoplastic MDS in the center.**

**Rationale:**

**Rationale: Patient corresponds to low-risk MDS without high-risk markers -> Guideline recommendation is observation, diagnostics to decide on therapy (sEPO, iron status) and targeted use of ESA for low sEPO/few transfusions. Specifics (del5q, SF3B1, hypoplastic) would justify specific therapies.**

**===== run_3 =====**

**Tumor board decision:**
Observation (watch and wait) with targeted additional diagnostics: serum EPO, ferritin/Tsat, reticulocytes, B12/folic acid, parvovirus/HBV/HCV, peripheral flow cytometry. For symptomatic anemia or anemia requiring transfusion: ESA (EPO 40,000 IU/week s.c. or darbepoetin) +/- G-CSF if sEPO <200 U/l. Luspatercept only in SF3B1/RS/requires transfusion and ESA failure; Lenalidomide only for del(5q); ATG+CsA only in cases of confirmed hypoplastic MDS. Bone marrow/extended NGS not routine, in case of progression or unclear findings; molecular follow-up annually.

**Rationale:**
Low-risk MDS (IPSS-M/IPSS-R low), 4% contrast blasts, no high-risk markers and no previous therapy -> conservative monitoring corresponds to the guideline. sEPO and iron status are crucial when selecting ESA therapy. Specific therapies (luspatercept/lenalidomide/ATG) only for the subtypes mentioned in the flowchart or for clear indications. Repeat CM/NGS if clinical deterioration occurs or as per annual follow-up.

## ========================================================== gpt-5-mini (plain) ==========================================================

**===== run_1 =====**

**Tumor board decision:**

Recommendation: For this IPSS-M low/IPSS-R low MDS without pretreatment: 1) Basic workup (reticulocytes, B12/folic acid, ferritin, transfusion requirement, serum EPO). 2) If there is symptomatic anemia or the need for transfusion: trial with erythropoiesis-stimulating agents (ESA) if serum EPO <500 mU/ml; otherwise supportive erythrocyte transfusions and close monitoring.

**Rationale:**
Low-risk MDS without blast excess, cytogenetic/molecular abnormalities and without prior therapy. ESAs are proven first-line therapy for symptomatic anemia and low EPO and can avoid transfusions. No evidence for lenalidomide (no del(5q)) or SCT.

**===== run_2 =====**

**Tumor board decision:**
In MDS (IPSS/IPSS-R low, blasts 4%): 1) Asymptomatic without need for transfusion -> close observation (Hb, need for transfusion, clinical status, CM control). 2) Symptomatic anemia or anemia requiring transfusion -> erythropoiesis-stimulating agents (EPO +/- if necessary G-CSF) according to serum EPO determination; supportive transfusions PRN. No Allo-SCT or HMA currently.

**Rationale:**
Low-risk MDS with normal cytogenetics and low blast percentage has a good prognosis. ESAs are standard therapy for symptomatic anemia (more effective with serum EPO <=500 mU/ml). Before therapy: determine EPO, iron status, B12/folic acid; Monitoring and adjustment in case of progression/transfusion requirement.

**===== run_3 =====**

**Tumor board decision:**
For low-risk MDS (IPSS-R low, blasts 4%), we recommend a conservative approach: check serum EPO, ferritin and transfusion requirements. If EPO <500 mU/ml or transfusion-dependent anemia, start erythropoiesis-stimulating therapy (e.g. epoetin alfa) +/- G-CSF in case of non-response. Otherwise symptomatic erythrocyte transfusions; at chron. Transfusion requirements Consider chelation therapy. Regular blood checks and BM reevaluation in case of clinical changes.

**Rationale:**
Low-risk MDS without high-risk cytogenetic/molecular features and without prior therapy. ESAs are standard for symptomatic anemia requiring transfusion and low serum EPO; Allogeneic SCT not indicated. Monitoring serves to detect progression at an early stage.

## ================================================== gpt-5-nano (agent) ==================================================

**===== run_1 =====**

**Tumor board decision:**
Recommendation: Low-risk MDS with anemia requiring transfusion -> ESAs. Epoetin alfa 40,000 U/W or darbepoetin 150 µg/week; if necessary G-CSF. Check Hb effect after 8-12 weeks; Continue speaking, otherwise abort. transfusions in parallel; check ferritin; Iron chelation for ferritin >1000 µg/L or extensive transfusion. allo-SCT-Eval in parallel, only after failure/progression; Immunosuppression only if there is clear hypocellularity/autoimmune signs.

**Rationale:**

**Rationale: The profile corresponds to low-risk MDS with anemia requiring transfusion; ESAs have good response rates. Immunosuppression only for hypocellularity/autoimmune signs. Transfusions and iron chelation in parallel. allo-SCT evaluation early, but only in case of failure/progression or polytransfusion.**

**===== run_2 =====**

**Tumor board decision:**
In suspected cases of low-risk MDS with hypocellularity: recommend immunosuppressive therapy ATG + CsA as first-line therapy. Determine baseline sEPO; ESA only if there is an actual contraindication or no response. Transfusions as needed; Iron chelation for ferritin >=1000 ng/mL or >=20 EC. allo-SCT evaluation in parallel; Transplantation only in the event of actual failure/progression; initiate donor search; 1 week reflection period.

**Rationale:**
Hypocellular CM + low risk level characteristics without adverse cytogenetics suggest IST with approximately 30% transfusion freedom. ESA only optional after ACTUAL characterization. Transplantation only in case of failure/progression; Iron chelation useful in potential transfusion.

Early preparation for transplantation makes sense, but no requirement for early transplantation.

**===== run_3 =====**

**Tumor board decision:**
Recommendation: Low-risk MDS with anemia initially ESA therapy (EPO 40,000 IU/week SC or darbepoetin according to SOP). If hypoplasty-MDS or no response: consider immunosuppressive therapy (ATG + CsA). Transfusions + iron chelation in parallel; allo-SCT evaluation only in the case of polytransfusion/unfavorable genetics or treatment failure; early transplant medical advice.

**Rationale:**
IPSS-R/M-low, blasts 4%, no TP53/ASXL1, normal cytogenetics, no ring sideroblasts. ESAs may show ~40-60% response in low-risk patients; Hypoplasia-MDS often responds to immunotherapy (~30%). allo-SCT only in case of treatment failure or HR genetics. Goal: Control anemia, avoid transplantation, timely planning.

## ================================================== gpt-5-nano (plain) ==================================================

**===== run_1 =====**

**Tumor board decision:**
Recommendation: Start erythropoiesis-stimulating therapy (ESA) for this low-risk MDS with anemia. Suggestion e.g. B. Darbepoetin alfa 150 µg q2w; Check/correct iron stores. Assess Hb response after 6-8 weeks. If response is inadequate due to hypocellular BM, consider IST (ATG + CsA); Continue transfusions as needed.

**Rationale:**
IPSS-R low, blasts 4%, normal cytogenetics and hypocellular BM indicate good ESA response. IST (ATG + CsA) is a useful second option if there is no ESA response. Approach minimizes risk, maximizes hemoglobin response and reduces transfusion-related stress.

**===== run_2 =====**

**Tumor board decision:**
Low-risk MDS (IPSS-R Low, IPSS-M Low) with anemia: First choice ESA therapy (e.g. epoetin alfa or darbepoetin) depending on EPO levels; transfusions PRN; Check iron status/ferritin and correct iron if necessary. If no response after 8-16 weeks: consider hypomethylating therapy or studies.

**Rationale:**

**Rationale: When risk is low, ESA therapy reduces transfusions and improves quality of life; no mutational/cytogenetic risk factors against ESA; Cytostatics/HMA only after progression; SCT only with suitable constitution or progression/high-risk.**

**===== run_3 =====**

**Tumor board decision:**
First-line ESA therapy (darbepoetin alfa) for low-risk MDS with anemia. Check EPO levels, iron status. If no response after 8-12 weeks or high EPO level: continue transfusion-based supportive care or review clinical trial.

**Rationale:**
Low risk (IPSS/IPSS-R), no 5q-del, no relevant mutations. ESAs standard for low-risk symptomatic anemia; avoids early HMAs; useful for anemia requiring transfusion.

## ================================================== gpt-oss-20b (agent) ==================================================

**===== run_1 =====**

**Tumor board decision:**
In a patient with MDS, IPSS-M/IPSS-R low-risk, 4% blasts and no symptomatic anemia, we recommend an observation/supportive approach (watch & wait) with monitoring of blood values every 6-12 months. If symptomatic anemia or transfusion is required later, ESA therapy can be initiated.

**Rationale:**
The flowchart (IV-A, LOW-1a) specifies observation as the initial option for asymptomatic, low-risk MDS without high-risk cytogenetics. The blast proportion is less than 5% and therefore does not meet the high-risk criterion. ESA is only recommended for symptomatic anemia or the need for a transfusion

(LOW-2d).

**===== run_2 =====**

**Tumor board decision:**
Observation (Watch & Wait) without immediate ESA or hypomethylation therapy. Continuous monitoring every 6-12 months, transfusions if necessary.

**Rationale:**
Patient is in IPSS-R/ IPSS-M low risk, <5% blasts, no HR cytogenetics, asymptomatic. Flowchart IV-A, LOW-1a recommends Watch & Wait. ESA only for symptomatic anemia requiring transfusion - not given.

**===== run_3 =====**

**Tumor board decision:**
In this patient with IPSS-M/IPSS-R Low, 4% blasts, no HR cytogenetics and no anemia requiring transfusion, we recommend an observation/supportive approach (watch & wait) with regular KMP every 6-12 months. ESA therapy only occurs when symptoms occur or a transfusion is required.

**Rationale:**
The flowchart (IV-A, LOW-1a) defines asymptomatic, HR-free, low-risk MDS as observation. The patient meets these criteria; 4% blasts are below the 5% limit. No previous therapies, no comorbidities, therefore no indication for ESA or hypomethylation. Regular monitoring allows early adjustment of therapy if clinical status changes.

## ================================================== gpt-oss-20b (plain) ==================================================

**===== run_1 =====**

**Tumor board decision:**
Recommendation: In case of transfusion-dependent anemia (or Hb<10g/dl), start 4 weeks of ESA therapy (e.g. epoetin-α 40,000IU weekly). If Hb>=10g/dl without the need for transfusion, only symptomatic support (folate, B12, if necessary iron chelate).

**Rationale:**
Low-risk MDS with anemia; ESA is effective when Hb<10g/dl or transfusion is required and is first line. No cytogenetics, therefore no lenalidomide. Iron chelation in transfusion dependence. Regular Hb monitoring.

**===== run_2 =====**

**Tumor board decision:**
Observation and symptomatic support. For anemia >10g/dl without need for transfusion: observation. If transfusion is required or Hb<10g/dl: use ESA (epoetin). Regular blood values, bone marrow check every 6 months.

**Rationale:**
Low-risk MDS (IPSS-Rlow) without del(5q) or other risk factors. First-line therapy is observation or ESA for transfusion-dependent anemia. No indication for intensive therapy.

**===== run_3 =====**

**Tumor board decision:**
Begin symptomatic, transfusion-based support. If Hb<10g/dl or transfusion requirement>4U/month, start ESA (epoetinα/darbepoetinα). For >10 transfusion packs, begin iron chelation. Regular monitoring of Hb, transfusion load and iron status.

**Rationale:**
Low-risk MDS with anemia, no karyotypic or molecular abnormalities. ESAs are the first choice for transfusion-dependent anemia. Iron chelation prevents overload during chronic transfusion. No aggressive therapies necessary.

## ================================================== gpt-oss-120b (agent) ==================================================

**===== run_1 =====**

**Tumor board decision:**

For IPSS-M low / IPSS-R low MDS with initial anemia without transfusions: initially observation (controls every 3-6 months, laboratory including serum EPO). If Hb<10g/dl or transfusion dependence and sEPO<200U/l, start ESA therapy (Epoetinα 40,000IU weekly, if necessary 80,000IU). No further therapy required.

**Rationale:**
Low-risk MDS, asymptomatic or mild anemia, no HR cytogenetics. According to flowchart IV-1a observation, in case of symptomatic anemia ESA therapy according to predictive factors. Other options (lenalidomide, luspatercept, ATG) not indicated.

**===== run_2 =====**

**Tumor board decision:**
For IPSS-M low / IPSS-R low, hypoplastic MDS without del(5q), SF3B1 or TP53 mutation and without current transfusions: initially observation (watch and wait) with checks every 6 months (blood count, reticulocytes, sEPO). In case of symptomatic anemia or transfusion>=2 RBC/8 weeks and sEPO<200U/l, start ESA therapy (EPO 40000IU/W or darbepoetin) +/- G-CSF.

**Rationale:**
Low-risk MDS without HR cytogenetics, no transfusions -> according to FlowchartIV-1a observation. ESA is only recommended in cases of symptomatic anemia/transfusions and favorable predictive factors.

**===== run_3 =====**

**Tumor board decision:**
Watch-and-wait; no immediate therapy. Control examinations (blood count, reticulocytes, serum EPO) every 3-6 months. If anemia requiring transfusion occurs or Hb<10g/dl, start with low sEPO (<200U/l) ESA (EPO 40,000IWk.).

**Rationale:**
Low-risk MDS (IPSS-R low, IPSS-M low), no HR cytogenetics, only mild anemia, no transfusions to date. According to FlowchartIV-1a, observation is recommended; ESA is only used for symptomatic or transfusion-dependent anemia.

## ================================================== gpt-oss-120b (plain) ==================================================

**===== run_1 =====**

**Tumor board decision:**
Observation (watch and wait) with symptomatic treatment of the anemia: erythropoiesis-stimulating agents (ESA) if Hb<10g/dl and transfusion-dependent, iron supplementation if necessary, regular control (CBC, BM morphology) every 3-6 months.

**Rationale:**
IPSS-M and IPSS-R low, blasts 4%, no cytogenetics, no previous therapy. If risk is low, no disease-modifying therapy is indicated; Symptomatic treatment of anemia is the recommended standard.

**===== run_2 =====**

**Tumor board decision:**
Further procedure: observation and supportive therapy. If anemia or transfusion dependence persists, use erythropoiesis-stimulating substances (ESA); otherwise regular checks (blood count, ferritin) without immediate disease-modifying therapy.

**Rationale:**
The patient has a low risk (IPSS-M/R low), no blast excesses, no del(5q) mutation and no previous therapy. For low-risk MDS, observation and symptomatic treatment (ESA if necessary) are initially recommended.

**===== run_3 =====**

**Tumor board decision:**
In the case of low-risk MDS (IPSS-M/R low, 4% blasts, no therapy yet), pure observation and supportive management is carried out. If anemia persists, ESA therapy (e.g. epoetin alfa) after EPO level control, if necessary RBC transfusions and iron chelation if transfusion dependence. No immediate disease-modifying therapy.

**Rationale:**

The clinical picture is low-risk, asymptomatic except for anemia. Without high-risk factors (>=5% blasts, cytogenetics, del(5q)), immediate use of lenalidomide, hypomethylation hormones or allo-SCT is not indicated. Supportive measures address the main symptoms and improve quality of life.

## ======================================================= qwen3-32b (agent) ========================================================

**===== run_1 =====**

**Tumor board decision:**
The v. a. MDS diagnosis should be revised. A repeat bone marrow biopsy is recommended to confirm the diagnosis and rule out other hypocellular syndromes. In confirmed MDS with transfusion-dependent anemia, luspatercept should be considered first-line.

**Rationale:**
The lack of blasts/ring sideroblasts and unremarkable molecular profiles speak against classic MDS. Hypocellular MDS are rare and require differential diagnoses such as aplastic anemias. Luspatercept is evidence-based in transfusion-dependent LR-MDS. HMAs are not standardized in low-risk MDS.

**===== run_2 =====**

**Tumor board decision:**
The v. a. MDS diagnosis should be revised. A comprehensive diagnosis according to the flowchart (DX-1 to DX-5) is recommended to rule out differential diagnoses (e.g. aplastic anemia, reactive changes). If hypoplastic MDS is confirmed (WHO 2022), immunosuppression (ATG + cyclosporine) should be considered. If uncertain: repeat the bone marrow biopsy with a focus on cellularity.

**Rationale:**
The diagnosis of MDS risk group low (IPSS-M/R) with normal molecular and cytogenetic diagnostics suggests hypoplastic MDS (new WHO entity). Absence of blasts/ring sideroblasts and normal karyotype support this. Differential diagnoses such as aplastic anemia or reactive changes must be excluded. Treatment options if confirmed: immunosuppression or watch & wait if uncertain.

**===== run_3 =====**

**Tumor board decision:**
The v. a. MDS diagnosis should be revised taking into account the hypocellular bone marrow findings and absence of molecular/cytogenetic aberrations. Differential diagnoses such as aplastic anemia or reactive changes must be excluded. Comprehensive diagnostics according to the flowchart (DX-1 to DX-5) including telomere length analysis (in young patients) and exclusion of Fanconi anemia are recommended. If a diagnosis of MDS (hypoplastic) is confirmed, immunosuppression (ATG + cyclosporine A) should be considered.

**Rationale:**
The diagnosis of MDS risk group low with missing blasts/ring sideroblasts and normal molecular findings suggests hypoplastic MDS (WHO 2022). However, differential aplastic anemia and reactive changes must be excluded. The lack of clonality in molecular testing supports revision of the diagnosis. If hypoplastic MDS is confirmed, immunosuppression is indicated, otherwise further treatment towards aplastic anemia is indicated.

## ======================================================= qwen3-32b (plain) ========================================================

**===== run_1 =====**

**Tumor board decision:**
Recommendation for observation with regular checks (every 3-6 months) and symptomatic therapy if necessary. No active therapies in low-risk MDS without blast formation or transfusion dependence.

**Rationale:**
Low-risk MDS with unremarkable cytogenetics and <5% blasts. No indication for chemotherapy or targeted therapy. Risk of progression low. Priority: Clinical surveillance and anemia management if symptoms occur.

**===== run_2 =====**

**Tumor board decision:**
Recommendation: observation with supportive therapy if symptoms occur. Regular checks to monitor disease progression.

**Rationale:**
Low-risk MDS with hypocellular bone marrow and no excess blasts. No molecular or cytogenetic risk factors. Therapy indication only for symptoms such as anemia or progression.

**===== run_3 =====**

**Tumor board decision:**
Recommendation: Supportive therapy with erythropoietin testing for anemia requiring transfusion. Regular monitoring of blood values and bone marrow checks. No indication for aggressive therapy.

**Rationale:**
Low-risk MDS (IPSS-M/R low) with hypocellular bone marrow and lack of molecular/cytogenetic abnormalities. No blasts or ring sideroblasts. Supportive therapy is standard. Erythropoietin in anemia response. No targeted therapy indication.

## ================================================ qwen3-next-80b (agent) ================================================

**===== run_1 =====**

**Tumor board decision:**
Further diagnostic measures required. The diagnosis of MDS is currently not confirmed.

**Rationale:**
Incomplete diagnosis: No results for vitamin B12, folic acid, ferritin, EPO, PNH test (flow cytometry), haptoglobin, LDH. Differential diagnoses such as megaloblastic anemia, PNH, aplastic anemia must be excluded. WHO 2022 classification requires exclusion of other causes before MDS diagnosis.

**===== run_2 =====**

**Tumor board decision:**
Further diagnostic measures required. No MDS diagnosis confirmed.

**Rationale:**
Diagnostic criteria require exclusion of differential diagnoses (e.g. vitamin deficiency, PNH, aplastic anemia). Peripheral blood diagnostics (DX-1) such as EPO, B12, folic acid, ferritin, virology are missing. Bone marrow findings alone are not sufficient for a reliable diagnosis of MDS.

**===== run_3 =====**

**Tumor board decision:**
MDS diagnosis not confirmed. Further diagnostic measures required.

**Rationale:**
According to Flowchart DX-1, comprehensive peripheral blood tests (vitamins, ferritin, LDH, haptoglobin, virology, EPO, etc.) are mandatory for exclusion diagnosis. Data to date do not show a complete clarification of the differential diagnoses (e.g. megaloblastic anemia, PNH, aplastic anemia).

## ================================================ qwen3-next-80b (plain) ================================================

**===== run_1 =====**

**Tumor board decision:**
Determine serum erythropoietin levels and initiate ESAs if values are <500 U/L. For higher values, transfusion-based supportive therapy. Regular checks.

**Rationale:**
IPSS-R low, no high-risk factors. ESAs are first choice for anemic MDS with low EPO. No contraindicated secondary diagnoses. No previous therapy, therefore no resistance.

**===== run_2 =====**

**Tumor board decision:**
MDS diagnosis confirmed (IPSS-R/low). Measure serum EPO. At EPO <500 U/L ESAs begin. If response is inadequate, consider transfusions or immunosuppressants.

**Rationale:**

Low risk, anemia symptomatic. ESAs are first line therapy for low EPO. No del(5q) or SF3B1 mutation, therefore no lenalidomide indication. Transfusions for symptomatic anemia.

**===== run_3 =====**

**Tumor board decision:**
Determine serum EPO and initiate ESA therapy (e.g. epoetin alfa) if values are <=500 U/L. If EPO is elevated, symptomatic blood transfusions.

**Rationale:**
IPSS-R low risk, no ring sideroblasts. ESA as first therapy for low EPO according to guidelines. No other treatment options indicated.

# l_case_12__ALL

################################
#### CASE: 12
################################

## ================================================== gpt-5-mini (agent) ==================================================

**===== run_1 =====**

**Tumor board decision:**
Immediately perform NGS-based ABL kinase mutation analysis plus FISH and RT-qPCR for BCR-ABL confirmation. In parallel, start cytoreductive hydroxyurea to control leukocytosis. Initiate immediate donor search/Allo-SCT evaluation. TKI change according to guidelines according to mutation findings: with T315I -> ponatinib (or asciminib 2x200 mg), without T315I -> preferably asciminib (2x40 mg) or ponatinib in the case of multiple resistance. No AML induction chemotherapy now (blasts 13%).

**Rationale:**
Accelerated CML (13% blasts) with progression on bosutinib -> dasatinib (failure to two 2GTKIs). Guidelines: mutation analysis/cytogenetics mandatory before switching to TKI; Donor search from failure on first 2GTKI. AML induction/blast protocol only if there are >=20% blasts or urgent clinical deterioration; Hydroxyurea recommended as bridging.

**===== run_2 =====**

**Tumor board decision:**
Immediate ABL kinase NGS (mutation analysis) and FISH confirmation; Interim cytoreducing therapy with hydroxyurea because of WBC 210/nl. If T315I or multiple resistance is detected: ponatinib (start 45 mg/d with strict cardiovascular monitoring) + immediate allo-SCT evaluation/donor search. Without T315I: Switch to asciminib (2x40 mg/d) and parallel transplant presentation/donor search.

**Rationale:**
Accelerated phase (13% blasts) with progression under dasatinib: Guideline requires mutation analysis in case of failure; for blasts <20%, primary TKI switch according to mutation profile, not routine AML induction. Ponatinib only for T315I/multi-drug resistance due to vascular risks; Prepare allo-SZT early after failure on 2GTKI.

**===== run_3 =====**

**Tumor board decision:**
Immediate confirmation of BCR-ABL diagnosis (RT-qPCR / FISH) and urgent ABL kinase mutation analysis (NGS). In parallel, immediately cytoreducing hydroxyurea (WBC 210/nl). Switching the TKI to asciminib (>=2nd line: 2x40 mg/d) as a rational second line without mutation result. If T315I or multiple resistance is detected: switch to ponatinib and immediate evaluation/donor search for allo-SCT. No AML induction now (blasts 13% = AP).

**Rationale:**
Patient in accelerated phase (13% blasts) with progression on bosutinib->dasatinib. Guidelines: AP -> mutation analysis + TKI switch, non-routine AML chemotherapy (<20% blasts). Asciminib offers a different mechanism of action when ATP-competitive TKIs fail; Ponatinib only for T315I/multiple resistance. Start donor search immediately.

## ================================================== gpt-5-mini (plain) ==================================================

**===== run_1 =====**

**Tumor board decision:**
Consequence: Immediate bone marrow puncture with cytogenetics/FISH and BCR-ABL1 kinase domain mutation test; cytoreductive therapy with hydroxyurea (in the case of symptomatic hyperleukocytosis, leukapheresis if necessary); rapid HLA typing and clarification for allogeneic SCT; If there are no cardiovascular contraindications, start ponatinib (dose individually) until mutation is found.

**Rationale:**
Patient presents with accelerated phase CML (13% blasts) with progression after bosutinib and dasatinib. In second-line failure, transplantation and/or ponatinib are indicated. Mutation testing

and cytogenetics decide the targeted TKI choice; rapid cytoreduction required due to WBC 210/nl.

**===== run_2 =====**

**Tumor board decision:**
Immediate cytoreduction with hydroxyurea (if there are signs of leukostasis, leukapheresis if necessary). Urgently arrange BCR-ABL kinase domain sequencing + FISH/RT-qPCR. In parallel, rapid HLA typing and clarification for allogeneic SCT. For T315I or multi-TKI resistance: ponatinib as a bridge to transplantation; if not transplantable: ponatinib or omacetaxine.

**Rationale:**
Accelerated stage CML (13% blasts) with massive leukocytosis and progression on two 2G TKIs. Mutation testing determines targeted therapy; Ponatinib is active against T315I/multi-resistance. Allogeneic SCT is the curative option, therefore immediate cytoreduction and rapid transplant evaluation.

**===== run_3 =====**

**Tumor board decision:**
Classified as accelerated phase CML (blasts 13%) with progression after bosutinib->dasatinib. Immediate measures: 1) Cytoreduction with hydroxyurea +/- leukocytaphaeresis for leukostasis symptoms; 2) immediate molecular workup (qPCR BCR-ABL, karyotype, kinase domain mutation) and HLA typing/transplant referral; 3) If there are no contraindications, start ponatinib 45 mg daily (check cardio-RF) until mutation findings are available; in case of contraindication/alternative therapy asciminib/transplant planning.

**Rationale:**
Accelerated phase and PD after two 2G-TKIs require rapid cytoreductive management, clarifying mutation diagnostics and rapid SCT strategy (potentially curative). Ponatinib is effective against resistant mutations (including T315I) and is justified as the next TKI option; Cardiac risk assessment and parallel planning for allo-SCT are mandatory.

## ==================================================== gpt-5-nano (agent) ====================================================

**===== run_1 =====**

**Tumor board decision:**
Diagnostics within 1-2 weeks: ABL1-KD mutations (including T315I), BCR-ABL1-qPCR, cytogenetics. For T315I or resistance: ponatinib 45 mg/d (or asciminib). For accelerated/blast phase progression: combination approach (ponatinib + blinatumomab or mini-HCVD + ponatinib) with SCT evaluation; Bridging protocols (Decitabine/Venetoclax/Ponatinib) possible; Start SCT in parallel.

**Rationale:**

**Rationale: Accelerated/blast phase with 13% blasts and TKIs progression requires timely mutation testing (including T315I), qPCR and cytogenetics. Ponatinib (45 mg/d) is primary for mutations/resistance; Asciminib alternative. For blast/accelerated progression: combinations (ponatinib+blinatumomab or mini-HCVD+ponatinib) + SCT preparation in parallel. Bridges possible. Evidence base available.**

**===== run_2 =====**

**Tumor board decision:**
Mutation tests (ABL1-DKM incl. T315I), BCR-ABL1-qPCR and cytogenetics ASAP; Results in 2-3 weeks. If resistant: ponatinib 45 mg/d or asciminib; SCT early; if necessary, combination approach per phenotype; Decision in weeks.

**Rationale:**
Mutation determines TKI choice; ponatinib or asciminib as main options; check SCT early; Consider risk benefit (vascular events).

**===== run_3 =====**

**Tumor board decision:**
Switch to ponatinib 45 mg/d as next TKI; SCT evaluation now. Consideration: Asciminib for T315I or ponatinib+blinatumomab as a bridge. Immediate diagnostics: BCR-ABL1 qPCR, ABL1-KD mutations, cytogenetics. Monitoring: qPCR every 4 weeks; Mutations/qPCR within 1-2 weeks; Adjustment per finding.

**Rationale:**

AP-CML with 13% blasts on bosutinib/dasatinib; Ponatinib active in resistance/intolerance. Early mutation detection and SCT plan increase chances of cure; Asciminib as an option in T315I. Combination/bridging strategies possible per mutation and response.

## ================================================ gpt-5-nano (plain) ================================================

**===== run_1 =====**

**Tumor board decision:**
Instant mutation test BCR-ABL kinase domain analysis + qPCR to determine molecular status. If mutations or progression to >=2 TKIs: switch to ponatinib 45 mg/d with close cardiovascular monitoring; If suitable, consider allogeneic transplantation in an accelerated phase or participation in a clinical trial.

**Rationale:**
Accelerated stage CML (13% blasts) progressed despite bosutinib/DAS; Mutation status influences TKI choice. Ponatinib effective against TKI resistance; Allogeneic transplantation potentially curative in suitable candidates; Clarify risk factors and monitoring in advance.

**===== run_2 =====**

**Tumor board decision:**
Rapid stage confirmation (qPCR BCR-ABL, FISH, bone marrow cytogenesis). If accelerated phase confirmed: switch to ponatinib 45 mg/d (if necessary 30 mg/d if there are vascular risks) with close molecular monitoring; parallel SCT evaluation and review of clinical studies.

**Rationale:**
PD with two TKIs and 13% blasts requires TKI with accelerated/blast phase activity and potentially curative option through allogeneic transplantation; Necessary clarification of the BCR-ABL status/resistance pattern before further therapy steps

**===== run_3 =====**

**Tumor board decision:**
Ponatinib 45 mg/d as next TKI change in AP-CML progression after bosutinib intolerance and dasatinib PD; close monitoring including BCR-ABL-qPCR; risk assessment of arterial events; parallel transplant evaluation (Allo-SCT) or clinical trials if transplantable/accessible.

**Rationale:**
AP-CML with 13% blasts and progression on two TKIs. Ponatinib provides activity in AP-CML; Seriously consider allo-SCT depending on transplantability. Check mutation status/BCR-ABL before therapy; consider vascular risks; if necessary clinical trial.

## =============================================== gpt-oss-20b (agent) ================================================

**===== run_1 =====**

**Tumor board decision:**
For PD on dasatinib with 13% blasts (AP) after failure of bosutinib and dasatinib, allogeneic SCT (allo-SCT) is the recommended therapy. A third TKI (e.g. ponatinib) is not the first choice for AP without T315I because transplantation is the only curative option.

**Rationale:**
The patient is in the accelerated phase (10-19% blasts) and has already failed two different 2GTKIs (bosutinib, dasatinib). According to FlowchartVIII-X, if two TKI lines fail in AP, allo-SCT is the indication. Ponatinib is primarily reserved for T315I mutation or multidrug resistance. Therefore, allo-SCT is recommended as the next step.

**===== run_2 =====**

**Tumor board decision:**
In PD on dasatinib with 13% blasts in the accelerated phase (ELTS-High), we recommend immediate initiation of allogeneic SCT (Allo-SCT). If transplantation is not possible, a TKI switch to ponatinib takes place (after mutation and cytogenetic analysis).

**Rationale:**
Progression after 2 TKI lines, 13% blasts -> accelerated phase, high-risk of ELTS, allo-SCT is the only curative option; Ponatinib remains a reserve if transplantation is not possible.

**===== run_3 =====**

**Tumor board decision:**
Start asciminib 80mg daily (or 2x40mg) as second-line TKI; simultaneously initiate mutation analysis (NGS). If there is no response, evaluate allo-SCT within 3 months.

**Rationale:**
Patient is in AP (13% blasts) and is resistant to bosutinib and dasatinib. Flowchart §VII-RES-2a recommends an alternative TKI in the absence of a T315I mutation; Asciminib has different mechanism of action, no cross-resistance and is preferred in CP/AP. Mutation analysis may determine additional options. If further failure occurs, allo-SCT is the only curative option.

## ================================================== gpt-oss-20b (plain) ==================================================

**===== run_1 =====**

**Tumor board decision:**
Switch to ponatinib 15mg daily (check for BCR-ABL status). Parallel evaluation for allogeneic SCT.

**Rationale:**
PD in accelerated phase despite dasatinib; Ponatinib has highest efficacy in AP/BC, especially without T315I mutation. BCR-ABL status is missing, so determine before starting therapy. Transplant remains an option with good performance.

**===== run_2 =====**

**Tumor board decision:**
Switch to ponatinib 15 mg daily (after BCR-ABL mutation test) or, if intolerance or insufficient control, consider an allogeneic SCT.

**Rationale:**
Accelerated Phase, PD on Bosutinib and Dasatinib, Ponatinib Shows High Efficacy in Resistant CML; Transplantation is the only curative option if patient is suitable for it.

**===== run_3 =====**

**Tumor board decision:**
Switch to ponatinib 15mg daily (or 30mg if risk factors). If suitable for transplantation, an allogeneic SCT can then be considered.

**Rationale:**
Patient has PD after bosutinib and dasatinib, no mutation data available, is in accelerated phase. Ponatinib is the recommended third generation TKI for TKI resistance and shows good remissions in the blast phase. Transplantation remains an option with good performance.

## ================================================== gpt-oss-120b (agent) ==================================================

**===== run_1 =====**

**Tumor board decision:**
If the disease progresses after bosutinib and dasatinib therapy (AP, ELTS score 2.9), immediately carry out mutation analysis (BCR-ABL-KD). Without T315I mutation and without atypical transcripts: start asciminib 80 mg daily (or 40 mg BID) as the next TKI. Initiate parallel Allo-SCT evaluation; in case of T315I mutation or no response after 2-3 months, ponatinib 45 mg daily (after cardiovascular screening) as an alternative.

**Rationale:**
13% blasts -> accelerated phase, intermediate/high-risk ELTS, already two 2GTKI failures. According to [RES-2a], guidelines recommend asciminib due to its high effectiveness and good side effect profile; Ponatinib for T315I or no response; Allo-SCT for TKI failure and AP as a curative option.

**===== run_2 =====**

**Tumor board decision:**
For detected AP (13% blasts) after failure of bosutinib and dasatinib: 1) Immediate BCR-ABL mutation analysis; 2) Without T315I and without cardiovascular risk factors, start ponatinib 45mg/d (after 3 months, if necessary, reduce to 15mg); use asciminib 80 mg/d if there is cardiovascular comorbidity

or contra-indication for ponatinib; 3) Parallel Allo-SCT evaluation as a curative option.

**Rationale:**
The patient is in the accelerated phase (13% blasts, ELTS score 2.9->high-risk) and has already failed two 2-GTKI therapies. After two TKI failures, guidelines recommend switching to ponatinib (broad mutation spectrum) or asciminib in the case of cardiac contraindications, as well as early allograft evaluation as a potentially curative measure.

**===== run_3 =====**

**Tumor board decision:**
Switch to asciminib (80 mg daily, if necessary 40 mg bid) as the next therapy; Simultaneous mutation analysis (BCR-ABL1 kinase domain) and early evaluation of an allo-SCT option if there is no molecular response within 3 months.

**Rationale:**
ELTS score >2, progression after bosutinib->dasatinib (intolerance/PD) = failure of two 2GTKIs. Asciminib is evidence-based effective and well tolerated after >=2 TKI failures, with no known contraindications. Ponatinib only for T315I/multiple resistance, nilotinib not preferred due to cardiovascular risks.

## ================================================== gpt-oss-120b (plain) ==================================================

**===== run_1 =====**

**Tumor board decision:**
Switch to ponatinib 45 mg/day (or dose-adjusted) and promptly consider allogeneic SCT as a curative option; Perform BCR-ABL mutation analysis immediately.

**Rationale:**
Patient has accelerated stage CML, TKI resistance after bosutinib and dasatinib, and high ELTS score; Ponatinib is the recommended 3rd gene TKI for TKI failure, and allo-SCT offers the only potential cure in this risk profile.

**===== run_2 =====**

**Tumor board decision:**
In chronic CML with PD after bosutinib and dasatinib, immediately carry out BCR-ABL mutation analysis (including T315I) and start ponatinib 45mg/day (if necessary 30mg if there is a cardiovascular risk) as third line. If there is no response or high-risk mutations, consider allogeneic SCT.

**Rationale:**
Two TKIs have already failed due to intolerance or progression; Ponatinib is the only TKI effective against almost all BCR-ABL mutations, including T315I. The ELTS score >2.5 indicates a high-risk, meaning a quick change and, if necessary, a transplant are necessary.

**===== run_3 =====**

**Tumor board decision:**
Switch immediately to ponatinib (45 mg/day, 30 mg if necessary if at cardiovascular risk) and initiate immediate evaluation for allogeneic SCT; carry out BCR-ABL mutation analysis (including T315I) at the same time.

**Rationale:**
Patient in CML accelerated phase, ELTS score 2.9 (high-risk), progression despite bosutinib and dasatinib. Ponatinib is the only effective 3rd generation TKI (also with T315I) and serves as a bridge therapy to allogeneic SCT, which is curative in the event of TKI failure.

## ==================================================== qwen3-32b (agent) ====================================================

**===== run_1 =====**

**Tumor board decision:**
Start therapy with asciminib 80 mg/d and initiate allo-SCT evaluation. Wait for mutation results (BCR-ABL) for definitive treatment adjustment.

**Rationale:**

For progressive CML with two previous TKIs (bosutinib, dasatinib) and blast accumulation (13%), asciminib is the first choice as a STAMP inhibitor with a different mechanism of action. ELTS score 2.935 (high-risk) warrants allo-SCT evaluation. BCR-ABL mutation results are crucial for final therapy adjustment (e.g. ponatinib in T315I).

**===== run_2 =====**

**Tumor board decision:**
Recommendation: Therapy with asciminib 80 mg/d (first line) or ponatinib 45 mg/d (for T315I mutation). Initiate concurrent allo-SCT evaluation. Prioritize mutation screening (NGS) and BCR-ABL confirmation.

**Rationale:**
For progressive CML with two TKI failures (bosutinib, dasatinib) and blast accumulation (13%), a switch to asciminib (different mechanism of action, no cross-resistance) or ponatinib (for T315I) is indicated. ELTS score 2.935 (high) and pending BCR-ABL confirmation require mutation screening. allo-SCT evaluation is mandatory in cases of multiple resistance.

**===== run_3 =====**

**Tumor board decision:**
Recommendation: Therapy with ponatinib (45 mg/d) in combination with blinatumomab or alternatively decitabine/venetoclax. Prioritization of BCR-ABL mutation analysis.

**Rationale:**
Progression under 2nd gene TKI and blast accumulation indicate resistance/resistance mutation. Ponatinib (3rd gene TKI) is standard for 35INS mutations. Combination with blinatumomab/chemo optimizes remission rates in Ph+ diseases. BCR-ABL confirmation is urgently required.

## ================================================== qwen3-32b (plain) ==================================================

**===== run_1 =====**

**Tumor board decision:**
Start ponatinib 45 mg/d as a third TKI. Prioritize BCR-ABL mutation analysis for therapy optimization.

**Rationale:**
Progression on two TKIs (bosutinib, dasatinib) and high ELTS score (2.935) justify third-generation TKIs. Ponatinib demonstrates efficacy against resistance and provides broad mutation coverage. Missing molecular findings require rapid analysis to adjust therapy.

**===== run_2 =====**

**Tumor board decision:**
Start ponatinib 45mg/d if BCR-ABL positivity is confirmed. Prioritize FISH diagnostics to confirm Philadelphia chromosome positivity.

**Rationale:**
Progression under bosutinib and dasatinib suggests resistant CML. Ponatinib is a third-generation TKI with high efficacy in resistant CML. FISH diagnosis is necessary to clarify BCR-ABL status and justify TKI therapy.

**===== run_3 =====**

**Tumor board decision:**
Switch to ponatinib 45mg/d as third TKI due to progressive disease and resistance to bosutinib/dasatinib. Initiate FISH/NGS in parallel to search for mutations (especially T315I).

**Rationale:**
CML accelerating phase with 13% blasts and ELTS score 2.935 (poor prognosis). Two TKI lines (bosutinib, dasatinib) failed. Ponatinib shows good efficacy in recurrent/progressive cases and overcomes common resistance mechanisms.

## ================================================ qwen3-next-80b (agent) ================================================

**===== run_1 =====**

**Tumor board decision:**
Mutation testing is mandatory before therapy adjustment. For T315I mutation: ponatinib or asciminib 2x200 mg/d. Without T315I: Asciminib 80 mg/d (2x40 mg/d). Check Allo-SCT and initiate donor search immediately.

**Rationale:**
Flowchart: Resistance after 2TKI requires mutation analysis (RES-1b). Asciminib as a STAMP inhibitor has no cross-resistance and better tolerability (ASCEMBL). Ponatinib only on T315I. SOP: Donor search from failure on 2GTKI (RES-2d). AP with treatment failure indicated Allo-SCT (SZT-1e).

**===== run_2 =====**

**Tumor board decision:**
Yes, ABL kinase mutation testing is mandatory before therapy adjustment. Based on result: For T315I ponatinib or asciminib 2x200 mg/d; for other mutations, asciminib (if no contraindications). Evaluate allogeneic SCT immediately and initiate donor search.

**Rationale:**
Flowchart shows mutation analysis as standard (RES-1b) in the event of treatment failure (2 TKIs). AP with 13% blasts requires Allo-SCT evaluation (SZT-1e). Ponatinib only for T315I or multiple resistance. Asciminib does not favor T315I due to different mechanism of action and better tolerability.

**===== run_3 =====**

**Tumor board decision:**
Be sure to carry out ABL kinase mutation testing. For T315I: ponatinib or asciminib 2x200 mg/d; for other mutations: asciminib (ATP-competitive TKI resistance). Initiate Allo-SCT evaluation immediately (SOP: donor search if there is resistance to the 2nd TKI line). Ponatinib only on T315I or after asciminib failure.

**Rationale:**
Flowchart VII: Mutation analysis mandatory in the event of treatment failure. Asciminib (STAMP inhibitor) no cross-resistance to ATP-competitive TKIs. Allo-SCT is the only curative procedure for multiple resistance (SZT-1c). Donor search necessary from 2nd TKI failure (SZT-2). Ponatinib only on T315I due to vascular complications.

## ================================================ qwen3-next-80b (plain) ================================================

**===== run_1 =====**

**Tumor board decision:**
Initiate ponatinib 45 mg/d. Perform NGS mutation testing. Check indication for allogeneic SCT.

**Rationale:**
Progression on bosutinib and dasatinib in accelerated phase (13% blasts). Ponatinib is the next treatment option, especially for T315I mutation. Transplantation evaluation in suitable patient situations.

**===== run_2 =====**

**Tumor board decision:**
Ponatinib 45 mg/d and referral for allo-SCT evaluation

**Rationale:**
PD on bosutinib and dasatinib, accelerated phase (13% blasts). Ponatinib as the next TKI option as it is also effective in T315I mutation. Allo-SCT is the only curative option in appropriate patients.

**===== run_3 =====**

**Tumor board decision:**
Perform mutation testing (NGS). If there is a T315I mutation, start ponatinib, otherwise asciminib. Initiate transplant evaluation.

**Rationale:**
Progression on bosutinib and dasatinib in accelerated phase (13% blasts). Without mutation data, targeted therapy is not possible. Allogeneic transplantation is curative option in suitable patients.

# l_case_13__ALL

################################
#### CASE: 13
################################

## ======================================================== gpt-5-mini (agent) ========================================================

**===== run_1 =====**

**Tumor board decision:**
If the patient is capable of intensive therapy: begin immediate intensive induction with 7+3 (cytarabine days 1-7, daunorubicin days 3-5). Continue hydroxyurea only if high leukocytosis/clinical leukostasis remains. In parallel, immediate complete molecular panel, cytogenetics/FISH, HLA typing and early allo-SCT evaluation. For WBC >20,000/µl or leukostasis: prephase (HU +/- cytarabine 100 mg/24h) until re-evaluation.

**Rationale:**
80% of contrast blasts require urgent therapy. Flowchart/Guidelines: Carry out diagnostics and HLA search in parallel, do not delay induction due to pending molecular diagnostics (unless there is leukostasis). NPM1 WT and FLT3-TKD neg. do not justify an add-on; Clarify transplant planning early on.

**===== run_2 =====**

**Tumor board decision:**
Plan intensive induction (standard 7+3) immediately, after rapid clinical fitness/organ function testing and exclusion of clinical leukostasis. Continue HU as bridging until induction begins. In parallel: full molecular panel, cytogenetics/FISH, immediate HLA typing and early allogeneic transplant evaluation. If the patient cannot be treated intensively: Azacitidine + Venetoclax.

**Rationale:**
80% BM blasts, no APL indication, NPM1 WT and FLT3-TKD neg. -> no specific target add-on; Standard concept for patients requiring intensive treatment is 7+3. Early complete genetic diagnosis and HLA/donor search are essential for a risk-adapted transplant decision. HU as short-term cytoreduction bridging.

**===== run_3 =====**

**Tumor board decision:**
If there are no contraindications and sufficient performance: immediate intensive induction with 7+3 (cytarabine days 1-7 + daunorubicin days 3-5). Continue hydroxyurea until therapy begins; In parallel, start complete molecular cytogenetics, HLA typing and early donor search. In clinical leukostasis, prephase according to SOP (HU +/- 100 mg cytarabine/24h or leukapheresis). If not intensively treatable: Azacitidine + Venetoclax.

**Rationale:**
80% BM blasts, NPM1 WT and FLT3-TKD negative provide no evidence of GO, midostaurin or CPX-351; The standard for patients requiring intensive treatment is 7+3. Diagnostics/HLA should not delay, but induction should not wait for complete molecular results. In leukostasis prephase; If you lack fitness, use a non-intensive regime.

## ======================================================== gpt-5-mini (plain) ========================================================

**===== run_1 =====**

**Tumor board decision:**
Suggested approach if fitness status is unknown: 1) fit -> induction '7+3' (cytarabine contin., daunorubicin); Hydroxyurea only until therapy begins. In parallel, complete cytogenetics and NGS (including FLT3-ITD, IDH1/2, TP53). Check HSCT if in remission; CD33+, possibly gemtuzumab depending on risk. 2) unfit -> azacitidine + venetoclax.

**Rationale:**
With 80% blasts, rapid curative action is indicated. So far only cytoreductive (HU). NPM1 WT and FLT3-TKD neg. no currently usable targeted option; Complete molecular and cytogenetic clarification required for risk stratification and transplant decision. If you are not fit, alternatively

HMA+Venetoclax.

**===== run_2 =====**

**Tumor board decision:**
For patients with a medical history and organ function fit: immediate intensive induction chemotherapy 7+3 (cytarabine 7d i.v. + daunorubicin d1-3). Continue hydroxyurea until therapy begins. Rapid complete diagnostics (cytogenetics, NGS, HLA) and early diagnosis for allo-SCT. If unfit: HMA + Venetoclax.

**Rationale:**
CM 80% blasts requires curative induction; Hydroxyurea alone is inadequate. FLT3-TKD negative -> no initial FLT3 inhibitor. Complete molecular/karyotypic results determine consolidation/transplantation. If there are contraindications to intensive therapy, alternatively HMA+Venetoclax.

**===== run_3 =====**

**Tumor board decision:**
Immediate complete diagnostics (complete cytogenetics, extended NGS, organ functions, infection status). Therapy recommendation: For patients who are fit/fit for treatment, immediate intensive induction in a curative approach (7+3: cytarabine IV continuously + daunorubicin). In unfit patients, azacitidine/decitabine + venetoclax. Hydroxyurea continues until therapy/TLS prophylaxis begins.

**Rationale:**
With 80% blasts there is acute myeloid leukemia; hydroxyurea was only cytoreductive. The aim is curative induction in fit patients. FLT3-TKD negative, therefore no current FLT3 inhibitor. Cytogenetics/NGS are missing and critical for consolidation/allo-SCT planning.

## ==================================================== gpt-5-nano (agent) ====================================================

**===== run_1 =====**

**Tumor board decision:**
Induction: 7+3 AML (cytarabine 100-200 mg/m2 IV continuously days 1-7 + daunorubicin 60 mg/m2 days 1-3). APL excluded; NPM1 WT, FLT3 TKD neg. Parallel molecular genetic panel (FLT3/NPM1/IDH1/2/TP53/CEBPA/RUNX1) and further tests for ELN risk stratification; Initiate donor/HLA search. After CR/CRi: risk-adapted consolidation and allo-SCT bridging per finding.

**Rationale:**
80% blasts, ELN risk stratification pending; no FLT3 courage, no NPM1 courage known; Standard induction makes sense. Lack of molecular testing limits early classification, but early transplant/bridging planning is recommended.

**===== run_2 =====**

**Tumor board decision:**
Induction AML 80%: 7+3 (Daunorubicin day 1-3 + Cytarabine day 1-7) immediately; End HU. Parallel: rapid cytogenetics/molecular genetics (FLT3, NPM1, IDH1/2, TP53) + MRD; Donor search. CR/CRi -> Allo-SZT bridge; otherwise ELN consolidation/studies.

**Rationale:**
80% blasts, FLT3 neg., NPM1 WT; Induction makes sense; rapid molecular profiling needed; early transplant planning/bridging; Check studies.

**===== run_3 =====**

**Tumor board decision:**
Induction: 7+3 standard (cytarabine 100-200 mg/m2 i.v. 7d + daunorubicin 60 mg/m2 d1-3). Parallel rapid molecular gene mapping (FLT3, NPM1, IDH1/2, TP53, CEBPA) and HLA donor search. If CR/CRi: HiDAC consolidation; early allo-SCT planning depending on MRD and ELN risk diversification; Pay attention to bridging.

**Rationale:**
80% blasts AML, non-APL, FLT3 TKD neg., NPM1 WT. Standard induction makes sense; rapid molecular risk stratification necessary; early SCT planning and MRD monitoring for transplant assurance.

## ==================================================== gpt-5-nano (plain) ====================================================

**===== run_1 =====**

**Tumor board decision:**
Induction chemotherapy AML standard (7+3) as first line with sufficient performance; Cancel HU. In parallel, complete molecular/karyotypic profiling (ELN risk) and subsequent risk-adapted consolidation; supportive measures according to guidelines.

**Rationale:**
80% blasts, no targeted mutation marker known; HU alone is insufficient. Induction of remission increases the chances of a curative outcome; Profiling enables appropriate consolidation.

**===== run_2 =====**

**Tumor board decision:**
Recommendation: Induction chemotherapy 7+3 (Cytarabine + Daunorubicin/Idarubicin) if performance is sufficient; timely cytogenetic/molecular diagnostics (ELN risk) and supportive care; for Unfitness Alternative: HMAs + Venetoclax.

**Rationale:**
80% blast AML; Hydroxyurea alone is not curative. Necessary risk stratification through complete cytogenetic/molecular findings to assess the prognosis; Target remission. If the load is too high, check the HMAs +/- Venetoclax option.

**===== run_3 =====**

**Tumor board decision:**
Recommendation: Definitive induction (7+3 or Idarubicin/Cytarabine) if you are sufficiently fit. Parallel cytogenetic/molecular workup for ELN risk classification. If induction is contraindicated: VEN+AZA/LDAC+VEN. Remission: SCT assessment.

**Rationale:**
80% blast AML; so far only HU. No known targetable mutations. Necessary risk stratification and SCT plan.

## ====================================================== gpt-oss-20b (agent) ======================================================

**===== run_1 =====**

**Tumor board decision:**
Immediately after completion of the HU leukostasis phase, start standard induction 7+3 (Cytarabine 100mg/m2 IV q12hDay1-7 + Daunorubicin 60mg/m2 IV q24hDay3-5). Do not delay induction until completion of genetic analysis and ELN classification.

**Rationale:**
The patient has 80% blasts, NPM1-WT, FLT3-TKD-neg.; no evidence of CBF-AML or other indications for alternative regimens. The flowchart rule [AML-GATE-1] allows induction after successful leukostasis reduction. Delay worsens prognosis, while genetic results concern regime adaptation (e.g. GO), not the need for 7+3.

**===== run_2 =====**

**Tumor board decision:**
Immediately begin standard induction (7+3) for this AML patient. Genetic analysis can run in parallel and will influence consolidation or transplant planning, but will not delay induction.

**Rationale:**
The patient has 80% blasts, no APL-suspicious features and no contraindications to 7+3. A delay in completing genetics would prolong the time to CR and increase the risk of complications. The genetic results are relevant for follow-up treatment, not for initial induction.

**===== run_3 =====**

**Tumor board decision:**
Start induction 7+3 after resolution of the leukostasis, continue HU until blast reduction, carry out genetic analysis in parallel.

**Rationale:**
Flowchart AML-Gate1 blocks induction at WBC>20000/µl; once leukostasis subsides, 7+3 can begin. Genetic results are determined in parallel in order to adapt later therapy plans.

## ================================================== gpt-oss-20b (plain) ==================================================

**===== run_1 =====**

**Tumor board decision:**
Starten Sie eine Standard-Induktionstherapie (Cytarabine 100 mg/m2 i.v. 7 Tage + Daunorubicin 60 mg/m2 i.v. 3 Tage).

**Rationale:**
AML with 80% blasts, NPM1 WT, FLT3-TKD neg. Hydroxyurea is only used to reduce blasts. A 7+3 induction is required for remission. No evidence of recurrent or resistant therapy, therefore standard induction recommended.

**===== run_2 =====**

**Tumor board decision:**
Initiate standard induction (7+3) with cytarabine 100mg/m2/day x7 and daunorubicin 60mg/m2/day x3. If unfit, consider alternative hypomethylation/venetoclax combination.

**Rationale:**
AML with 80% blasts, no contraindicating markers, no targeting options. Standard induction is the evidence-based initial therapy in fit patients. Alternatives only if you are unfit.

**===== run_3 =====**

**Tumor board decision:**
Start standard induction (7+3) with cytarabine + daunorubicin; If there is a later risk of ELN, consider intensification or allo-SCT if necessary.

**Rationale:**
AML with 80% blasts, no FLT3 mutations, NPM1 WT; Hydroxyurea only temporary cytoreduction. Standard induction is the evidence-based initial therapy. Further decisions depend on later genetic results.

## ================================================== gpt-oss-120b (agent) ==================================================

**===== run_1 =====**

**Tumor board decision:**
After completing the hydroxyurea prephase, immediately start intensive induction with 7+3 (cytarabine 100mg/m2 d1-7, daunorubicin 60mg/m2 d1-3). Due to CD33 positivity, supplement GO (3mg/m2) on days 1, 4, 7 unless contraindicated. Initiate HLA typing and Allo-SCT planning in parallel.

**Rationale:**
The patient meets the AML INTENSIVE criteria (no APL, leukostasis controlled, FLT3 TKD negative, intermediate ELN risk). The flowchart recommends 7+3 as standard induction; GO can increase CR success in CD33-positive AML. Early transplant evaluation is indicated in cases of possible high-risk.

**===== run_2 =====**

**Tumor board decision:**
After hydroxyurea cytoreduction, intensive induction (7+3) with the addition of gemtuzumab ozogamicin (CD33 positive) should be carried out in clinically fit patients. When CR/CRi is reached, Hi-DAC consolidation and, depending on ELN risk stratification (intermediate/high), Allo-SCT planning takes place. If suitable for non-intensive use, alternatively azacitidine+venetoclax.

**Rationale:**
AML without FLT3 or NPM1 mutation, CD33 positive, normal karyotype -> intermediate risk; intensive therapy with GO improves survival. Allo-SCT offers curative option for intermediate/high-risk. Non-intensive regimen for patients not suitable for 7+3.

**===== run_3 =====**

**Tumor board decision:**
Intensive induction with 7+3 (cytarabine 100-200mg/m2 continuously days 1-7, daunorubicin 60mg/m2 days 1-3). No FLT3/IDH inhibitors, GO not indicated (no NPM1 Mut/CBF). After CR/CRi MRD control, HLA typing; Allo-SCT in CR1 with suitable donor and intermediate to adverse ELN risk.

**Rationale:**

The patient is fit for therapy; hydroxyurea was only used for cytoreduction. Without FLT3-TKD, IDH mutations or favorable cytogenetics, there is no targeted adjunct. 7+3 delivers highest remission rate; Allo-SCT is planned in a risk-adapted manner according to remission and MRD results.

## ================================================== gpt-oss-120b (plain) ==================================================

**===== run_1 =====**

**Tumor board decision:**
Start of intensive induction chemotherapy (7+3: cytarabine 100-200mg/m2 7 days + daunorubicin 60mg/m2 days 1-3). If there are no contraindications, carry out immediately; After remission, further treatment according to ELN risk classification (consolidation, if necessary allo-SCT).

**Rationale:**
Patient has 80% blasts, CD33 positive, no previous effective therapy. Hydroxyurea is not enough for the cure. Intensive induction is standard to achieve remission; Further therapy depends on risk stratification after complete molecular and cytogenetic analysis.

**===== run_2 =====**

**Tumor board decision:**
Start intensive induction therapy (e.g. 7+3: Cytarabine 100-200mg/m2 d1-7 + Daunorubicin 60mg/m2 d1-3) after confirming the patient's fitness. In case of intolerance/old age, alternative therapy with azacitidine+venetoclax. At the same time, continue complete molecular diagnostics (including IDH1/2, C-KIT), supplement midostaurin if FLT3-ITD is later positive.

**Rationale:**
80% blasts, AML-typical immunophenotype, missing FLT3-TKD mutation and no high-risk cytogenetics -> classic AML, which requires curative intensive care induction in fit patients. Hydroxyurea alone is only cytoreductive. Alternative low-intensity therapy for unfit people. Further molecular profiling needed for targeted supplements.

**===== run_3 =====**

**Tumor board decision:**
Initiate intensive induction chemotherapy (Cytarabine 100-200mg/m2 7 days + Daunorubicin 60mg/m2 days 1-3); In case of remission, check consolidation with HD-AraC or Allo-HSCT. If intensity is not suitable, azacitidine+venetoclax as an alternative.

**Rationale:**
AML with 80% blasts, missing FLT3 ITD/NPM1 mutation -> intermediate risk. Hydroxyurea is not sufficient for remission. Standard 7+3 offers highest remission rate in suitable patients; If there is intolerance, the hypomethylating + venetoclax combination is evidence-based.

## ================================================== qwen3-32b (agent) ==================================================

**===== run_1 =====**

**Tumor board decision:**
Recommended therapy: Induction regimens 7+3 (cytarabine + daunorubicin) after completion of cytoreductive hydroxyurea therapy. If the clinical situation is stable and leukocyte reduction is sufficient. Parallel continuation of genetic diagnostics for ELN risk classification.

**Rationale:**
The patient does not have FLT3-ITD or CBF rearrangements that would warrant modified induction. The current genetic findings (NPM1 WT, FLT3 TKD neg.) speak for a standardized 7+3 regimen. The prerequisite is clinical stability and sufficient cytoreduction. The ELN risk classification is relevant for further decisions (e.g. Allo-SCT).

**===== run_2 =====**

**Tumor board decision:**
Induction therapy with the standard regimen 7+3 (cytarabine days 1-7 + daunorubicin days 3-5) is recommended after completion of cytoreductive hydroxyurea therapy. Participation in clinical studies should be examined in parallel.

**Rationale:**

The diagnosis of AML without suspicion of APL and the completed cytoreduction allow the transition to induction. FLT3-TKD negativity and NPM1 wild type indicate intermediate risk. 7+3 is the standard for non-specific mutations. ELN risk classification needs to be done.

**===== run_3 =====**

**Tumor board decision:**
Start induction therapy with 7+3 (cytarabine + daunorubicin). Consider gemtuzumab ozogamicin (GO) if appropriate ELN risk classification. Check clinical trial participation.

**Rationale:**
After cytoreductive hydroxyurea therapy, induction with 7+3 is standard if leukocyte control is stable. GO can be added for intermediate/favorable ELN risk. FLT3-TKD negativity and NPM1-WT argue against specific targeted therapies in the first line.

## ================================================== qwen3-32b (plain) ==================================================

**===== run_1 =====**

**Tumor board decision:**
Start induction chemotherapy with cytarabine and anthracycline (e.g. daunorubicin).

**Rationale:**
The AML in ED 01/26 requires definitive induction therapy. HU was only cytoreductive. ELN risk classification and molecular data are incomplete, however, the standard treatment for non-complicated AML is induction chemotherapy. Age and ECOG are missing, but no contraindications are known.

**===== run_2 =====**

**Tumor board decision:**
Start induction chemotherapy with cytarabine + anthracycline (e.g. daunorubicin). Complete ELN risk assessment after complete genetic findings.

**Rationale:**
Acute myeloid leukemia in ED 01/26 requires rapid induction treatment. Hydroxyurea alone is not curative. The FLT3 negativity and lack of unfavorable cytogenetics support standard induction therapy. Further genetic data is required for risk classification.

**===== run_3 =====**

**Tumor board decision:**
Start induction chemotherapy with cytarabine + anthracycline (7+3 regimen), provide ELN risk classification and plan bone marrowoscopy after completion of therapy.

**Rationale:**
The diagnosis of blast stage acute myeloid leukemia with a good genetic profile (NPM1 WT, FLT3 neg.) justifies standard induction therapy. Hydroxyurea alone is not curative. Age and ECOG are missing, but the high blast percentage suggests aggressive therapy. Once remission has been achieved, consolidation options must be examined.

## ================================================ qwen3-next-80b (agent) ================================================

**===== run_1 =====**

**Tumor board decision:**
Begin induction with 7+3 (cytarabine/daunorubicin). Once FLT3-ITD status is known, add midostaurin if positive.

**Rationale:**
No suspicion of APL (CD34+), leukocyte check via HU necessary. Diagnostics must not delay therapy. FLT3-ITD status pending, therefore start induction with standard 7+3. If FLT3-ITD is positive, midostaurin (independent of age, QTc risk can be better controlled than quizartinib).

**===== run_2 =====**

**Tumor board decision:**
No, continue the prephase until leukocytes are reduced below 20,000/µl. Then begin induction with 7+3 (cytarabine/daunorubicin). If FLT3-ITD is positive, add midostaurin.

**Rationale:**
The patient is currently in the prephase due to leukocytosis (HU therapy). Only start induction after WBC control. FLT3 ITD status pending; Midostaurin only if the result is positive.

**===== run_3 =====**

**Tumor board decision:**
Do not start induction therapy immediately. First ensure WBC control and clinical stabilization. Continued HU and daily re-evaluation. If WBC <20,000/μl and clinical stability, start induction with cytarabine/anthracycline. If FLT3-ITD is positive, add midostaurin.

**Rationale:**
Flowchart requires leukocyte reduction to <20,000/μl before induction if leukostasis is suspected. Currently HU pretherapy, WBC status unclear. Only start induction after checking the WBC and stabilization. FLT3-ITD status pending, therefore midostaurin add-on if positive.

## ================================================= qwen3-next-80b (plain) =================================================

**===== run_1 =====**

**Tumor board decision:**
Initiation of standard induction therapy with cytarabine (7 days) and daunorubicin (3 days) [7+3 regimen]. If FLT3-ITD status is positive, supplement with midostaurin.

**Rationale:**
Hydroxyurea is only cytoreductive, not curative therapy. AML requires urgent induction. No contraindications (no secondary diagnoses), therefore 7+3 as standard. FLT3-ITD status pending but do not delay therapy.

**===== run_2 =====**

**Tumor board decision:**
Immediately start inductive chemotherapy with cytarabine and daunorubicin (7+3 regimen). Hydroxyurea is only cytoreductive and not curative.

**Rationale:**
AML requires inductive chemotherapy for remission. Hydroxyurea alone is not sufficient. No contraindications (no secondary diagnoses, age unknown, but standard induction in fit patients. Wait for genetics, but do not delay therapy.

**===== run_3 =====**

**Tumor board decision:**
Conduct fitness assessment (age, ECOG, comorbidities). For fit patients, induction with cytarabine/anthracycline (7+3), for unfit patients low-intensity therapy (e.g. azacitidine + venetoclax).

**Rationale:**
Hydroxyurea is not a curative therapy. Standard therapy for AML requires induction, dependent on fitness. Age and ECOG are missing, therefore necessary to make treatment decisions.

# l_case_14__ALL

################################
#### CASE: 14
################################

================================================== gpt-5-mini (agent)
==================================================

**===== run_1 =====**

**Tumor board decision:**
Ponatinib 30 mg p.o. continue daily (if the vascular risk is high, reduce the dose to 15 mg) for at least 24 months or until the end of the maintenance phase; Complete planned GMALL consolidations and then maintenance (6 MP/MTX) in parallel with TKI. No routine blinatumomab in persistent MRD negativity (reserve for MRD reactivation). MRD (BCR-ABL) monitoring: monthly 0-6M, every 6-8W 6-12M, q3M 12-24M, then q3-6M. Complete intrathecal CNS prophylaxis according to GMALL. Allo-Tx should only be discussed again in the event of a molecular recurrence (>10^-4 or increasing BCR-ABL in two measurements), cytological recurrence or the appearance of ponatinib-resistant mutations or therapy-limiting TKI intolerance.

**Rationale:**
MRD negative CR under ponatinib -> standard is TKI continuation plus full protocol consolidation; Ponatinib covers T315I, among others. Blinatumomab primarily for MRD positivity/rescue. Close PCR monitoring allows early intervention; CNS prophylaxis according to GMALL prevents CNS relapses. Because of DM II, cardiovascular risk management and, if necessary, dose reduction are recommended.

**===== run_2 =====**

**Tumor board decision:**
Continue ponatinib: if there is a vascular risk (e.g. DM) 15 mg daily, otherwise 30 mg daily; at least 24 months during complete GMALL consolidation and maintenance. Complete planned chemo consolidations. Blinatumomab only in MRD reactivity/MolFail. MRD: BCR-ABL qPCR monthly x6 -> then every 6-8 weeks until 12 months -> thereafter q3M. CNS: intrathecal prophylaxis according to GMALL. Allo should be discussed again in the case of confirmed molecular increase (>10^-4), cytological recurrence or TKI failure.

**Rationale:**
MRD-negative CR allows TKI maintenance instead of primary allo; DM increases vascular risk -> dose reduction; complete consolidation reduces risk of recurrence; close qPCR monitoring detects MolRez early for timely changeover/allo planning; Blinatumomab reserved for MolRez/MolFail.

**===== run_3 =====**

**Tumor board decision:**
Continue ponatinib 30 mg daily during all subsequent consolidation and maintenance phases; If toxicity is relevant, reduce dose to 15 mg. Total duration at least 24 months from CR (if necessary until the end of maintenance). Complete consolidations according to GMALL-EVOLVE. Blinatumomab not routine; only in the case of molecular recurrence/MolFail. MRD: BCR-ABL qPCR monthly x6, then every 6-8 weeks until month 24, then every 3 months. CNS: intrathecal prophylaxis according to the protocol in parallel with systemic therapy (HD-MTX/AraC as planned). Allo-Tx should be discussed again in the event of loss of MRD negativity (increase >10^-4), MolFail, cytological CM recurrence or detection of relevant TKI resistance mutation.

**Rationale:**
Patient in MRD-negative CR on ponatinib; GMALL concept recommends continuous TKI therapy parallel to consolidations to avoid molecular relapses. Blinatumomab is primarily for MRD positivity/MolFail. Close BCR-ABL monitoring enables early intervention and re-evaluation for Allo-Tx when molecular or cytological failure mode or TKI resistance occurs.

================================================== gpt-5-mini (plain)
==================================================

**===== run_1 =====**

**Tumor board decision:**
Continue ponatinib as maintenance therapy (maintenance dose, e.g. 15 mg daily) in combination with GMALL maintenance (oral 6-MP + MTX according to the schedule) and continued intrathecal CNS

prophylaxis. Close BCR-ABL-qPCR monitoring (monthly x6, then every 3 months). Strict cardiometabolic management (diabetes optimization); In case of toxicity, dose reduction/change of TKI.

**Rationale:**
MRD-negative CR after ponatinib-based induction/consolidation; AlloTx is rejected. Continuation of effective TKI plus maintenance reduces risk of recurrence. Increased arterial risk from ponatinib due to diabetes: close monitoring, dose adjustment in case of side effects.

**===== run_2 =====**

**Tumor board decision:**
Patient in MRD-negative CR after GMALL-Evolve with ponatinib. Since Allo-Tx rejected: Completion of the planned consolidation according to protocol; subsequent maintenance with ponatinib-based therapy (ponatinib initially 30 mg -> reduction to 15 mg daily if CR is stable) plus standard intrathecal CNS prophylaxis. Close-meshed MRD/BCR-ABL PCR (monthly first), cardiovascular risk control. If MRD returns: consider rapid treatment with blinatumomab +/- change to TKI or CAR-T.

**Rationale:**
Ph-positive cALL: Allo-Tx is standard but rejected. MRD-negative CR allows conservative strategy: continuation of effective TKI-based therapy with completion of consolidation and subsequent maintenance with ponatinib, plus CNS prophylaxis and close MRD monitoring. Vascular risk (DM) requires dose reduction/monitoring. In case of MRD reactivation, use secondary salvage options (blinatumomab +/- TKI, CAR-T).

**===== run_3 =====**

**Tumor board decision:**
Recommendation: Since MRD-negative CR was achieved under ponatinib and AlloTx was rejected, continuation of the GMALL-EVOLVE protocol: complete all planned consolidation cycles including complete CNS prophylaxis; followed by maintenance phase with ponatinib (long-term) according to protocol. Close qPCR MRD controls (initially monthly). Cardiometabolic monitoring.

**Rationale:**

**Rationale: Ph+ ALL showed MRD-neg CR under ponatinib; for patients without AlloTx, the best strategy is consistent TKI-based consolidation and maintenance. Maintain ponatinib due to proven efficacy. In case of MRD recurrence, consider blinatumomab or a change in therapy. Control CV risks/diabetes and adjust TKI dose according to risk.**

## ======================================================= gpt-5-nano (agent) =======================================================

**===== run_1 =====**

**Tumor board decision:**
Ponatinib maintenance in MRD-negative CR; MRD check continues. For MRD positivity/relapse: immunotherapy (blinatumomab/inotuzumab) or studies; Autologous transplantation is rare; CAR-T depending on availability.

**Rationale:**
No donor; MRD negative -> TKI maintenance preferred. Immunotherapy/CAR-T useful for MRD risk; Be aware of transplant risks; Check study options.

**===== run_2 =====**

**Tumor board decision:**
Continuation of long-term ponatinib maintenance in MRD-negative CR, SCT rejected. Regular MRD monitoring (e.g. every 8-12 weeks). If MRD relapse/MolFail: MRD-guided salvage with blinatumomab or inotuzumab as a bridge; If necessary, participation in clinical studies. Autologous transplantation as a backup option; Allo-SZT only if preference changes.

**Rationale:**
Ph+ ALL in MRD-neg CR without SCT option requires an effective, less toxic long-term strategy. Ponatinib provides continuous control of the BCR-ABL clone. MRD-guided salvage can prevent relapse; Studies increase further options. Autologous SCT is less well documented; SCT remains optional if patient choice changes.

**===== run_3 =====**

**Tumor board decision:**

MRD-guided immunotherapy (blinatumomab) or CAR-T as preferred further treatment for MRD-negative Ph+ ALL after GMALL-EVOLVE; Long-term ponatinib maintenance not recommended. Backup: Autologous transplant or clinical trials; Allo-TBI rejected.

**Rationale:**
Evidence shows MRD-guided immunotherapy (blinatumomab/CAR-T) as the preferred option; Ponatinib maintenance unclear. Allo-TBI remains an alternative, but rejected here. Autologous transplantation/studies sensible alternatives; MRD-guided strategies recommended.

## ================================================== gpt-5-nano (plain) ==================================================

**===== run_1 =====**

**Tumor board decision:**
Continuation of ponatinib-based maintenance therapy in MRD-negative CR; no AlloTx. Close MRD controls. Consider CAR T-cell therapy as a definitive option if available; otherwise continue TKI-supported remission strategy.

**Rationale:**
Ph+ALL has high-risk of relapse despite MRD-negative CR. AlloTx ideal but rejected. CAR-T offers a near-curative alternative; Ponatinib remains a central TKI. Intensive MRD monitoring required.

**===== run_2 =====**

**Tumor board decision:**
MRD-negative CR after GMALL-EVOLVE: continuation of ponatinib-based maintenance therapy without AlloTx; regular MRD checks; if necessary, ponatinib dose reduction (e.g. 30 mg/d) due to DM2/vascular risks; clinical trial review; Re-evaluate transplant if patient elects.

**Rationale:**
Relapse risk in Ph+ ALL high; MRD-negative CR favorable, but AlloTx standard. Patient refuses transplant; TKIs make sense as conservation. DM2 increases TKI risks; therefore dose reduction/monitoring is recommended.

**===== run_3 =====**

**Tumor board decision:**
Continuation of ponatinib-based maintenance therapy in MRD-negative CR; Allotransplantation is not performed due to patient veto. Regular MRD monitoring (every 6-12 weeks). Consider CAR T-cell therapy as a reserve in case of recurrence/MRD relapse.

**Rationale:**
Ph+ ALL in first remission has a high-risk of relapse. Allotransplantation reduces relapse but is rejected by the patient. MRD-negative CR with TKIs allows controlled continuation of therapy. CAR-T remains an option as a later measure.

## ================================================= gpt-oss-20b (agent) ==================================================

**===== run_1 =====**

**Tumor board decision:**
Continuation of TKI maintenance with ponatinib (alternatively imatinib in the case of tolerance problems) for at least 2 years, regular MRD checks (q3 months) and, if MRD increases, blinatumomab therapy. CAR-T therapy only in case of recurrence.

**Rationale:**
Ph+ B-ALL in MRD-negative CR; allo-transplant rejected. Flowchart B1 Ph+ recommends TKI maintenance after consolidation. Ponatinib has high potency and CNS penetration. MRD monitoring allows early intervention. CAR-T is for relapse, not for first CR.

**===== run_2 =====**

**Tumor board decision:**
Continue TKI maintenance with ponatinib (e.g. 15 mg daily) until at least 2 years or until MRD increases again. MRD monitoring every 3 months. In case of relapse: CD19 CAR T therapy (e.g. brexucabtagene autologous) or blinatumomab as bridging therapy.

**Rationale:**

MRD-negative CR in Ph+ ALL; TKI maintenance is standard, allotransplantation rejected. Ponatinib offers high efficacy; in case of relapse, CAR-T or blinatumomab are proven salvage options.

**===== run_3 =====**

**Tumor board decision:**
Continuation of ponatinib maintenance (15 mgqd) in combination with 2 cycles of blinatumomab (28 day infusion) as the first option. In case of recurrence or intolerance, consider CAR-T therapy (CD19 targeting, e.g. tisagenlecleucel).

**Rationale:**
Patient is MRD-negative, allo-transplant rejects. Studies show that ponatinib maintenance with blinatumomab increases event-free survival rates in MRD-negative B-ALL. CAR-T therapy delivers high remission rates in BCR-ABL+ B-ALL when blinatumomab is not possible. Diabetes is not a contraindication.

## ================================================== gpt-oss-20b (plain) ==================================================

**===== run_1 =====**

**Tumor board decision:**
MRD-negative CR in BCR-ABL+ c-ALL, AlloTx rejected -> Start of TKI maintenance (ponatinib 30mgqd) combined with low-dose cytarabine (20mg/m2qd) for 2 years. Regular MRD monitoring and side effect monitoring.

**Rationale:**
Ponatinib has high remission rates in BCR-ABL+ ALL; Combination with cytarabine reduces relapse risk. MRD-negative CR allows conservative therapy without AlloTx. Continuous monitoring ensures early detection of relapses.

**===== run_2 =====**

**Tumor board decision:**
Continue ponatinib maintenance (15 mg daily) for 2 years, with regular MRD monitoring (every 3 months). If MRD positivity or clinical progression: initiate blinatumomab or inotuzumab ozogamicin. Altherapy (Allo-Tx) remains excluded.

**Rationale:**
Patient is MRD negative CR, BCR ABL positive; Ponatinib shows a high remission rate and reduces the risk of relapse. Altherapy was rejected. Maintenance with ponatinib is standard, MRD monitoring enables early intervention.

**===== run_3 =====**

**Tumor board decision:**
Continue TKI maintenance with ponatinib (30 mg daily) for 2-3 years, MRD monitoring every 3 months. In case of MRD positivity or clinical progression: low-dose AraC (25mg/m2, 5 days) or switch to dasatinib. Monitor blood sugar closely and adjust diet/medication if necessary.

**Rationale:**
The patient is MRD-negative CR after intensive induction/consolidation with ponatinib. In BCR-ABL+ B-ALL, TKI-Maintenance is the default option when Allo-Tx is rejected. Ponatinib remains the preferred TKI for CR; In the case of MRD positivity or progression, low-dose AraC or a TKI switch is evidence-based. Diabetes comorbidity requires blood glucose monitoring.

## ================================================== gpt-oss-120b (agent) ==================================================

**===== run_1 =====**

**Tumor board decision:**
Further therapy: Continuation of ponatinib as monotherapeutic maintenance treatment for at least 24 months (alternatively up to 3 years), combined with light maintenance chemotherapy (6-MP+MTX) according to the SR maintenance regimen. MRD checks every 3 months. If TKI is not tolerated, switch to dasatinib or participate in a study with blinatumomab+TKI.

**Rationale:**
The patient is in MRD-negative CR, has already received intensive induction/consolidation and refuses SCT. In Ph-positive ALL, continuous BCR-ABL inhibition after remission is crucial; Ponatinib

maintenance significantly improves DFS. Supplemental 6-MP/MTX corresponds to the SR maintenance regimen and reduces the risk of relapse. Alternatives for intolerance are available.

**===== run_2 =====**

**Tumor board decision:**
Since the patient has achieved an MRD-negative CR after induction/consolidation and refuses allogeneic SCT, we recommend continuous ponatinib maintenance (30 mg daily, dose adjustment if necessary after toxicity) for at least 24 months, supplemented by the SR maintenance regimen (6-MP+MTX) up to 130 weeks. Regular MRD checks (every 3 months) and QTc monitoring are mandatory. Participation in a study with ponatinib+blinatumomab may be offered as an alternative.

**Rationale:**
Ph-positive ALL with MRD-negative CR benefits from long-term TKI therapy; Ponatinib covers the risk of BCR-ABL mutations. The SR maintenance regimen reduces relapse rates and is intended in the flowchart for Ph-positive patients. Without SCT, the combination of ponatinib and light chemotherapy is the current standard.

**===== run_3 =====**

**Tumor board decision:**
Continuation of ponatinib as maintenance therapy (at least 24 months, possibly longer if tolerated) combined with an SR maintenance phase (6 MP/MTX up to week 130). If side effects occur, change to TKI (e.g. dasatinib). Alternatively: Enrollment in a study with blinatumomab+ponatinib.

**Rationale:**
Patient is in MRD-negative CR, has already received intensive induction/consolidation and refuses allo-SCT. Guidelines recommend continuous TKI maintenance after MRD negativity is achieved; Ponatinib offers the highest efficacy in Ph-ALL. Combination with light maintenance chemotherapy reduces the risk of relapse, while a study offer can enable additional deep remission.

## ================================================== gpt-oss-120b (plain) ==================================================

**===== run_1 =====**

**Tumor board decision:**
Continuation of therapy with ponatinib as monotherapeutic maintenance (e.g. 30 mg daily) for at least 24 months, combined with optional low-dose POMP maintenance (prednisone, vincristine, 6-mercaptopurine, methotrexate) if necessary. Close MRD controls (every 3 months) and clinical follow-up. Allogeneic transplantation is not carried out because the patient refuses it.

**Rationale:**
Patient is in MRD-negative CR after intensive induction/consolidation with ponatinib. In Ph-positive ALL without allotransplantation option, long-term TKI maintenance is standard to prevent relapses. Ponatinib has demonstrated good efficacy and tolerability following prior administration; Additional chemotherapy can be used to secure MRD if necessary.

**===== run_2 =====**

**Tumor board decision:**
Continuation of ponatinib-based maintenance therapy without allotransplantation. Continue ponatinib 30 mg daily, combined with a standard maintenance phase (vincristine, prednisone, if necessary intrathecal methotrexate) for at least 24 months, close MRD monitoring.

**Rationale:**
Patient is in MRD-negative CR after intensive induction/consolidation. If allotransplantation is refused, the evidence-based alternative is long-term TKI maintenance, particularly ponatinib, which improves deep remissions and survival. Supplementary low-dose maintenance reduces the risk of recurrence; Regular MRD monitoring enables early intervention.

**===== run_3 =====**

**Tumor board decision:**
Continuation of ponatinib maintenance (300 mg/day, dose reduction if necessary in case of toxicity) for at least 24 months with regular MRD monitoring; no allotransplantation because rejected; If necessary, participation in a TKI maintenance study.

**Rationale:**

Patient is in MRD-negative CR after intensive therapy with ponatinib; Allotransplantation is not desired. In BCR-ABL-positive ALL, long-term TKI maintenance is the standard follow-up therapy, reduces the risk of relapse and is well tolerated.

## ==================================================== qwen3-32b (agent) ====================================================

**===== run_1 =====**

**Tumor board decision:**
Recommendation: Continue ponatinib in combination with maintenance therapy (6-mercaptopurine/MTX) according to the SR therapy protocol. Continue regular MRD monitoring and CNS prophylaxis.

**Rationale:**
The patient is MRD negative in CR after GMALL-EVOLVE. If AlloTx is excluded, continuation of the TKI (ponatinib) with maintenance therapy (6MP/MTX) is indicated according to the SOP. HD-MTX/asparaginase blocks are completed. DM type II does not require any adjustment in therapy.

**===== run_2 =====**

**Tumor board decision:**
Continuation of ponatinib as monotherapeutic maintenance therapy with close MRD monitoring (every 3-6 months) excluding AlloTx.

**Rationale:**
MRD-negative CR after GMALL-EVOLVE therapy in Ph+ ALL justifies TKI maintenance. Ponatinib has already been used in induction/consolidation and is indicated in SOP for Ph+ ALL without AlloTx. No evidence of progression or MolFail. Diabetic comorbidity requires tight metabolic control.

**===== run_3 =====**

**Tumor board decision:**
Continuation of ponatinib as maintenance therapy with MRD monitoring. No further cycles of chemo required for MRD-negative CR. Allo-SCT excluded.

**Rationale:**
The patient shows MRD negative CR according to GMALL-EVOLVE protocol. If allo-SCT has been ruled out, continuation of the TKI ponatinib as maintenance therapy with regular MRD monitoring is standardized. Further cycles of chemotherapy are not indicated if MRD negativity is stable.

## ==================================================== qwen3-32b (plain) ====================================================

**===== run_1 =====**

**Tumor board decision:**
Continuation of ponatinib in combination with ALL-standardized maintenance therapy (e.g. methotrexate, 6-mercaptopurine, vincristine) according to the GMALL-EVOLVE protocol. Conduct regular MRD monitoring.

**Rationale:**
MRD-negative CR after induction/consolidation with ponatinib. AlloTx rejected. Ponatinib remains central to BCR-ABL-positive c-ALL. Maintenance therapy optimizes recurrence prevention. Diabetes background requires metabolic monitoring.

**===== run_2 =====**

**Tumor board decision:**
Recommendation: Continue ponatinib-based consolidation therapy according to the GMALL-EVOLVE protocol (e.g. Consolidation II) with MRD monitoring. Alternatively, consider inotuzumab-ozogamicin as maintenance therapy if protocol allows.

**Rationale:**
MRD-negative CR after induction and consolidation I with ponatinib-based regimen. AlloTx rejected. Ponatinib remains central to prevention of recurrence. Inotuzumab-ozogamicin (CD22 targeting) provides additional remission stabilization in B-ALL. Diabetic comorbidity requires precise dosage adjustment.

**===== run_3 =====**

**Tumor board decision:**
Recommendation: Continue ponatinib as monotherapeutic maintenance therapy with close MRD monitoring. Consider combination with minimally toxic substances such as low-dose methotrexate or 6-mercaptopurine.

**Rationale:**
MRD-negative CR after GMALL-EVOLVE protocol with ponatinib. AlloTx rejected. Ponatinib as a key TKI for BCR-ABL targets. Type II diabetes requires therapy with few complications. Maintenance with TKI plus low-dose chemotherapy reduces the risk of recurrence.

## ================================================== qwen3-next-80b (agent) ==================================================

**===== run_1 =====**

**Tumor board decision:**
Recommendation: Ponatinib monotherapy as maintenance therapy. No additional chemotherapy because MRD negative and no allo-SCT indication.

**Rationale:**
Ph+ B-ALL with MRD-negative CR after GMALL-EVOLVE therapy requires continuous TKI therapy. Ponatinib is effective and has already been used successfully. allo-SCT rejection makes TKI monotherapy the default option. Type II DM requires cardiovascular monitoring but is not a contraindication to ponatinib.

**===== run_2 =====**

**Tumor board decision:**
Recommend ponatinib monotherapy as maintenance therapy. Regular monitoring of cardiovascular parameters and blood glucose control due to type II DM.

**Rationale:**
In Ph+ B-ALL with MRD-negative CR and rejection of allo-SCT, TKI maintenance is standard. Ponatinib has already been used effectively in induction/consolidation. allo-SCT refusal requires continuous TKI therapy. Ponatinib monotherapy is evidence-based, and cardiovascular monitoring is essential in type II DM.

**===== run_3 =====**

**Tumor board decision:**
Continuation of ponatinib as monotherapy for maintenance therapy

**Rationale:**
TKI monotherapy is standard for Ph+ ALL maintenance after MRD-negative remission. No evidence for combination therapy. Ponatinib has already been used successfully; type II DM requires monitoring but no contraindication.

## ================================================== qwen3-next-80b (plain) ==================================================

**===== run_1 =====**

**Tumor board decision:**
Continuation of ponatinib as maintenance therapy with regular MRD surveillance and cardiovascular monitoring due to type II diabetes.

**Rationale:**
For Ph+ ALL without allo-SCT, lifelong TKI therapy is standard. Ponatinib is effective (MRD negative), so continued. Diabetes increases cardiovascular risks, so close monitoring.

**===== run_2 =====**

**Tumor board decision:**
Continue ponatinib as long-term maintenance therapy with regular monitoring of cardiovascular risks and diabetes management.

**Rationale:**
MRD-negative CR after ponatinib-based therapy, AlloTx rejected. The standard for Ph+ ALL without transplantation is continuous TKI therapy. Ponatinib remains appropriate, but careful monitoring of adverse events due to diabetes and cardiovascular risks.

**===== run_3 =====**

**Tumor board decision:**
Continue maintenance therapy with ponatinib in combination with oral 6-mercaptopurine (6-MP) and weekly methotrexate (MTX)

**Rationale:**
MRD negative CR after consolidation, AlloTx rejected. Ponatinib is standard in Ph+ ALL. Combination with 6-MP/MTX is established maintenance therapy for Ph+ ALL without transplantation. Type II diabetes does not require a contraindication, but requires regular monitoring.

# l_case_15__ALL

################################
#### CASE: 15

################################

## ========================================================= gpt-5-mini (agent) =========================================================

**===== run_1 =====**

**Tumor board decision:**
Immediate salvage therapy: nelarabine-based regimen (e.g. nelarabine +/- cyclophosphamide) as bridging. Immediate HLA typing and rapid planning of an allogeneic SCT in remission. In parallel: complete restaging (BM, PET-CT) and CSF test; NGS/rebiopsy and study inclusion concurrently. Primary locoregional RT only in life-threatening compression/SVC syndrome or as bridging, not priority.

**Rationale:**
Early progression with GMALL in mediastinal T-LBL is considered refractory; GMALL/Flowchart recommends nelarabine as the first salvage in T-entity. Aim: cytological remission to allo-SCT. NGS/studies important must not delay urgent salvage. Local RT only for acute symptoms.

**===== run_2 =====**

**Tumor board decision:**
In mediastinal T-LBL with progression under GMALL: start immediate salvage chemotherapy based on nelarabine (e.g. nelarabine +/- cyclophosphamide), parallel to rapid HLA typing/donor search with the aim of bridging to allogeneic SCT. Clarify CSF status/PET; In the case of life-threatening compression, short-term locoregional RT to control symptoms.

**Rationale:**

**Rationale: GMALL algorithm: cytological failure in T-ALL/T-LBL -> Nelarabine as standard salvage for remission induction before allo-SCT. Molecular rebiopsy/NGS and study inclusion are useful, but must not delay the immediately necessary salvage therapy and donor search. RT only for urgent local control.**

**===== run_3 =====**

**Tumor board decision:**
For refractory mediastinal T-LBL under GMALL: initiate immediate salvage therapy with nelarabine-based regimen (if necessary + cyclophosphamide) and quickly start HLA typing/allo-SCT workup in parallel. Perform PET/CT and rebiopsy/NGS in parallel. Primary locoregional radiotherapy only for mass-related compression/symptoms.

**Rationale:**
GMALL-SOP: cytological failure in T-ALL/T-LBL -> nelarabine (+/- cyclophosphamide) as first salvage and bridging to allo-SCT. RT is secondary to local threat. NGS/rebiopsy and study inclusion are useful, but should not delay immediate salvage/transplant planning.

## ========================================================= gpt-5-mini (plain) =========================================================

**===== run_1 =====**

**Tumor board decision:**
Recommendation: Start salvage therapy immediately with a nelarabine-based regimen (e.g. nelarabine +/- cyclophosphamide/etoposide). In case of mediastinal compression, rapid palliative radiotherapy before/in parallel. In CR, rapid HLA typing and consolidation with allogeneic SCT. In parallel: repeat biopsy, complete staging and NGS.

**Rationale:**
Progression after GMALL in T-LBL; Nelarabine has best evidence in refractory/relapsed T-ALL/LBL and serves as a bridge to allo-SCT. RT for compression symptoms for rapid local control. Rebiopsy/NGS and HLA typing for further therapy planning and trial clarification.

**===== run_2 =====**

**Tumor board decision:**

In case of progression under GMALL: immediate salvage therapy with nelarabine (monotherapy or nelarabine-based combination regimen) for induction with the aim of bridging to allogeneic SCT. For symptomatic/threatening mediastinal masses, additional cortisone and/or palliative radiotherapy. Parallel review of CAR-T/studies.

**Rationale:**
Nelarabine is an established therapy for refractory/relapsed T-ALL/T-LBL; The aim is to achieve a CR for curative allo-SCT. In the case of a massive mediastinal mass, rapid local measures (steroid/RT) are indicated to control symptoms. Studies/CAR-T should be evaluated as an option.

**===== run_3 =====**

**Tumor board decision:**
Recommendation: immediate salvage therapy with nelarabine-based regimen (e.g. nelarabine +/- cyclophosphamide/etoposide) to achieve remission; in case of mediastinal compression, immediate palliative radiotherapy and/or dexamethasone. In CR/PR: rapid consolidation through allogeneic SCT. In case of non-response: FLAG-IDA or inclusion in clinical study/CAR-T.

**Rationale:**
Patient with T-LBL, PD after GMALL induction/consolidation. Nelarabine has documented activity in refractory/relapsed T leukemias/lymphomas. The aim is remission induction followed by allo-SCT as curative consolidation. If there are threatening mediastinal symptoms, immediate local measures are taken.

## ==================================================== gpt-5-nano (agent) ====================================================

**===== run_1 =====**

**Tumor board decision:**
CD7 CAR-T (BE-CAR7/NS7CAR) as a primary bridge to allo-SCT in refractory TLBL; alternatively salvage chemo +/- allo-SCT; RT for symptoms; Study parallel.

**Rationale:**

**Rationale: CD7 CAR-T is considered a promising bridge to SCT (Ph1); Salvage chemo + SCT remains an alternative; RT for symptomatic mediastinal mass; Pay attention to safety aspects.**

**===== run_2 =====**

**Tumor board decision:**
CD7-targeted CAR-T therapy (NS7CAR/BE-CAR7) as salvage with bridging to allo-SCT; If necessary, reduce mediastinal tumor volume with radiotherapy; Address CNS involvement. If CAR-T is not available: salvage chemotherapy followed by allo-SCT; Participation in NS7CAR/BE-CAR7 studies preferred.

**Rationale:**
Similar cases show remission signals with CD7 CAR-T and allow allo-SCT; Radiotherapy serves as a bridge for mediastinal masses; CNS involvement addressable. Literature/conference data support NS7CAR/BE-CAR7; clinical trials prefer, but further confirmation is needed.

**===== run_3 =====**

**Tumor board decision:**
TLBL-PD after GMALL: primary salvage with nelarabine for remission/MRD-CR and bridging to allo-SCT; parallel testing CD7-CAR-T or TLBL study; If necessary, reduce mediastinal bulk with local RT; SCT after CR/MRD-CR; alternative salvage in case of unavailability: Hyper-CVAD/FLAG-IDA.

**Rationale:**

**Rationale: Nelarabine has established activity in T-ALL/LBL and enables remission to realize allo-SCT; CD7-CAR-T/study options provide additional bridge; RT is used for rapid bulk reduction in mediastinal masses; primary goal is CR/MRD-CR followed by SCT.**

## ==================================================== gpt-5-nano (plain) ====================================================

**===== run_1 =====**

**Tumor board decision:**

Salvage chemotherapy TLBL: FLAG-IDA as first-line salvage (Hyper-CVAD as alternative). The goal is remission and timely allogeneic SCT, if suitable for transplantation and a donor is available. Support the mediastinal bulk with radiotherapy if necessary.

**Rationale:**
PD after GMALL; TLBL requires remission for curative allo-SCT. No targeted options known; Salvage chemotherapy offers the best chance of remission and subsequent transplantation. Local radiation therapy for bulky mediastinum can support the accompanying circumstances.

**===== run_2 =====**

**Tumor board decision:**
Salvage therapy with Nelarabine (relapsed/refractory T-LBL/T-ALL) as next line; Goal of remission and bridge to allogeneic SCT (allo-SCT) if there is a response; If necessary, additional radiotherapy only if the mediastinum is bulky.

**Rationale:**
PD under GMALL; Nelarabine established as salvage in T-LBL/ALL; remission enables allo-SCT; no molecular targeting options available; NGS/FISH negative.

**===== run_3 =====**

**Tumor board decision:**
Nelarabine salvage therapy as the next step in refractory TLBL after GMALL; after 2 cycles of restaging; in remission, allogeneic SCT; If necessary, local mediastinal radiotherapy depending on size/pressure symptoms.

**Rationale:**
PD after frontline chemotherapy; TLBL shows activity on nelarabine; Allogeneic SCT offers a chance of cure in remission; Radiotherapy for local control of the mediastinal findings is optional depending on the symptoms.

## ======================================================= gpt-oss-20b (agent) =======================================================

**===== run_1 =====**

**Tumor board decision:**
Salvage with HyperCVAD (alternatively GMALL salvage) to achieve remission; in CR, plan allo-SCT immediately.

**Rationale:**
T-LBL after GMALL induction/consolidation shows progression -> no remission. Flowchart V-3 recommends individual salvage; HyperCVAD is an established second-line approach. Allo-SCT only after CR, as the risk is too high in refractory patients without remission.

**===== run_2 =====**

**Tumor board decision:**
Second line of intensive salvage therapy (e.g. HyperCVAD or Nelarabine-based) to achieve remission, followed by allo-SCT for CR.

**Rationale:**
T-LBL after GMALL induction/consolidation PD -> salvage required. Flowchart V-3 recommends remission first, then allo-SCT. Immediate transplantation without remission carries high mortality.

**===== run_3 =====**

**Tumor board decision:**
Salvage HyperCVAD (alternating with L-asparaginase) as second line, then allo-SCT in remission. HyperCVAD is the recommended second line for T-LBL after GMALL failure; allo-SCT only takes place after remission according to GMALL logic (V-3/V-4).

**Rationale:**
After progression under GMALL, further chemotherapy is necessary. HyperCVAD (or L-asparaginase-based regimens) is the standard salvage in T-LBL. Allogeneic SCT is only attempted after remission (CR/MRD-neg), as described in GMALL flowchart V-3/V-4.

## ======================================================= gpt-oss-20b (plain) =======================================================

**===== run_1 =====**

**Tumor board decision:**
Start Nelarabine as a salvage program (2 mg/kg IV 5 days, 2 week interval) with subsequent assessment; plan allo-SCT in case of remission.

**Rationale:**
T-ALL with PD after GMALL regimens; Nelarabine is the standardized salvage therapy for T-ALL relapse and has the highest remission rate. When remission is achieved, an allogeneic SCT is the only curative option.

**===== run_2 =====**

**Tumor board decision:**
Start Nelarabine-based salvage therapy (e.g. Nelarabine + HD-Ara-C) and evaluate early for allogeneic SCT. Participation in a clinical trial should also be considered.

**Rationale:**
The patient is on PD after GMALL regimen. Nelarabine is the standard salvage option in refractory T-ALL/T-LBL and has been proven to achieve remissions. Early SCT evaluation increases the chances of survival; Study participation offers access to innovative therapies.

**===== run_3 =====**

**Tumor board decision:**
Start nelarabine-based salvage therapy (e.g. nelarabine 1.5 mg/m2 IV 1-5 days q28 days) in combination with high-dose methotrexate. After remission/reduction, we evaluate an allogeneic SCT.

**Rationale:**
Patient showed PD after GMALL regimen. Nelarabine is the first choice for relapsed/relapsing T-ALL without specific mutations. Combination with methotrexate increases remission rate. If remission is achieved, allo-HSCT is the only potential curative option.

## ================================================== gpt-oss-120b (agent) ==================================================

**===== run_1 =====**

**Tumor board decision:**
Salvage therapy with Nelarabine-based regimen (e.g. Nelarabine+HD-cyclophosphamide) as bridging, then allogeneic SCT if CR is achieved; at the same time check inclusion in a suitable study (e.g. CD7-CAR-T).

**Rationale:**
Patient has progressive T-LBL after GMALL induction/consolidation, no longer effective therapy. According to the GMALL flowchart, Nelarabine is the recommended 1st salvage option for T-ALL/LBL; allo-SCT offers curative prospects for CR; clinical trials (CD7-CAR-T) may be an additional option.

**===== run_2 =====**

**Tumor board decision:**
In progressive TLBL after GMALL induction/consolidation, use a nelarabine-based salvage regimen (e.g. nelarabine+dexamethasone+/-cyclophosphamide) as a bridge therapy, followed by allogeneic SCT when a CR is achieved; simultaneously check for inclusion in a CD7 CAR T study.

**Rationale:**
Nelarabine is the preferred salvage regimen for refractory T-ALL/T-LBL according to the GMALL algorithm. Allo-SCT is the only curative option if a CR is achieved. The tumor is CD7 positive, so a CD7 CAR T trial is a sensible, possibly superior option.

**===== run_3 =====**

**Tumor board decision:**
Start of nelarabine-based salvage therapy (e.g. nelarabine+dexamethasone+/-cyclophosphamide) with target CR, followed by allogeneic SCT in remission; If a suitable course of study is available (e.g. CD7-CAR-T), simultaneous examination and, if necessary, admission.

**Rationale:**
T-LBL after GMALL induction and consolidation is progressive. According to GMALL and ALL guidelines, nelarabine is the preferred salvage regimen for T-ALL/LBL, followed by allo-SCT as the curative

option. One study arm (CD7-CAR-T) can further increase the remission rate.

## ================================================== gpt-oss-120b (plain) ==================================================

**===== run_1 =====**

**Tumor board decision:**
Initiate nelarabine-based salvage therapy (e.g. Nelarabine+ICE) for remission, followed by allogeneic SCT if response is achieved. If CD30 positive, alternatively brentuximab vedotin-based.

**Rationale:**
After GMALL therapy, progressive TLBL is present. Nelarabine is effective in refractory T-ALL/LBL; a remission salvage enables curative consolidation by allo-SCT, which offers the best prospect of long-term control in the R/R setting.

**===== run_2 =====**

**Tumor board decision:**
Start nelarabine-based salvage therapy (30 mg/m2 i.v. on days 1, 3, 5 in a 21-day cycle) or alternatively ICE, and plan an allogeneic SCT if there is a response.

**Rationale:**
The patient presents with progressive T-LBL after GMALL induction and consolidation. Nelarabine is effective in refractory T-cell ALL/LBL and serves as a bridge therapy to potentially curative allo-SCT.

**===== run_3 =====**

**Tumor board decision:**
Switch immediately to salvage therapy (e.g. Nelarabine-based or BFM re-induction) and plan an allogeneic allotransplantation if there is a response; alternatively, participation in a CD7 CAR T study protocol.

**Rationale:**
The patient has progressive TLBL after GMALL induction and consolidation. Further GMALL regimen is ineffective; Salvage chemotherapy followed by allo-SCT is standard for refractory T-ALL/T-LBL, and CD7-CAR-T offers a targeted option.

## ================================================== qwen3-32b (agent) ==================================================

**===== run_1 =====**

**Tumor board decision:**
Recommendation: CD7-targeted CAR T cells (BE-CAR7) as the next therapeutic step. Alternative options: daratumumab or bortezomib.

**Rationale:**
According to evidence, BE-CAR7 (CD7-specific) is the priority option for recurrent TLBL after GMALL/KonsIV therapy with PD. CAR-T cells show maximum effectiveness in similar cases. Daratumumab/bortezomib are secondary alternatives.

**===== run_2 =====**

**Tumor board decision:**
Recommendation: Salvage therapy with nelarabine-based protocol (e.g. GMALL salvage) or cyclophosphamide-based regimen. If there is a response, consider allo-SCT early. Secure CNS prophylaxis in parallel.

**Rationale:**
For TLBL with PD after GMALL and KonsIV, nelarabine or cyclophosphamide are indicated as salvage options according to guidelines (Flowchart V.3). PET-adapted therapy and CNS protection are crucial. Allo-SCT remains a long-term option in remission.

**===== run_3 =====**

**Tumor board decision:**
Recommendation: Salvage therapy with nelarabine-based protocol (e.g. GMALL salvage) and evaluation for allo-SCT if there is a response. Secure CNS prophylaxis in parallel.

**Rationale:**
In TLBL with PD after GMALL and KonsIV, salvage with nelarabine/nelarabine+cyclophosphamide is indicated after SOP. Allo-SCT remains a goal in CR. PET-adapted stratification and CNS control are relevant to the decision.

## ======================================================== qwen3-32b (plain) ==========================================================

**===== run_1 =====**

**Tumor board decision:**
Recommendation: Salvage chemotherapy (e.g. ESHAP or ICE) followed by high-dose chemotherapy and SCT if remission is achieved.

**Rationale:**
Progression after GMALL therapy requires aggressive salvage therapy. TLBL is chemotherapy sensitive, hence next line with high dose protocols. SCT as consolidation in response.

**===== run_2 =====**

**Tumor board decision:**
Recommendation: Salvage therapy (e.g. ESHAP or ICE) followed by high-dose chemotherapy and SCT if transplantable.

**Rationale:**
Progression after GMALL/CHOP regimens requires salvage therapy. TLBL is highly aggressive, therefore a curative approach with transplantation is indicated if salvage therapy responds. No molecular targets present.

**===== run_3 =====**

**Tumor board decision:**
Start salvage chemotherapy (e.g., ESHAP or ICE) and plan for autologous SCT if there is a response.

**Rationale:**
The TLBL is aggressive and shows PD after GMALL. Salvage regimens with transplantation are standard for relapsed/refractory cases. Molecular data is lacking, therefore no targeted therapy.

## ====================================================== qwen3-next-80b (agent) ======================================================

**===== run_1 =====**

**Tumor board decision:**
Use of nelarabine-based salvage therapy followed by allo-SCT in remission. Review of clinical trials.

**Rationale:**
After PD after GMALL induction/ConsIV, T-LBL is refractory. Flowchart V-3: Ph-LBL -> individual decision. T-ALL salvage with nelarabine (cytological failure). Allo-SCT in remission. Review clinical trials.

**===== run_2 =====**

**Tumor board decision:**
Salvage therapy with nelarabine (possibly combined with cyclophosphamide) and subsequent allo-SCT in remission. Check study inclusion.

**Rationale:**
T-LBL after progression after GMALL induction/consolidation corresponds to cytologic failure. Flowchart V-3 for T-ALL (analogous to T-LBL) recommends nelarabine. LBL specific individual, but T cell entity requires similar strategy as T-ALL. Allo-SCT as a bridge for remission. Review studies (e.g. nelarabine studies).

**===== run_3 =====**

**Tumor board decision:**
Salvage therapy with nelarabine (e.g. as monotherapy or combination with cyclophosphamide) and immediate examination of a clinical trial. If in remission, aim for allo-SCT.

**Rationale:**

T-LBL with progression after GMALL induction/consolidation is considered refractory. Flowchart V-3 for Ph- T-ALL/LBL recommends nelarabine for cytologic failure. Prioritize study inclusion as standardized options are limited. Allo-SCT as a bridge to remission.

## ================================================ qwen3-next-80b (plain) ================================================

**===== run_1 =====**

**Tumor board decision:**
Initiate salvage therapy with Nelarabine (1500 mg/m2 IV 3x/week). If there is a response, perform allo-SCT.

**Rationale:**
Progression on GMALL protocol. Nelarabine is standard salvage therapy for T-cell neoplasms. No molecular therapy options available. Allo-SCT is a curative option if there is a response.

**===== run_2 =====**

**Tumor board decision:**
Initiate salvage therapy with nelarabine-based combination (e.g., nelarabine + dexamethasone). If responsive, pursue allo-SCT.

**Rationale:**
T-LBL with PD after GMALL induction. Nelarabine is standard for refractory T-cell malignancies. No molecular options available. Allo-SCT is a curative option in remission.

**===== run_3 =====**

**Tumor board decision:**
Initiate salvage therapy with Nelarabine (1500 mg/m2 IV on days 1,3,5 every 21 days). If in remission, seek allogeneic stem cell transplantation.

**Rationale:**
Progression to first-line therapy requires salvage. Nelarabine is standard in T-cell malignancies. Allogeneic transplantation offers the only curative option in cases of remission. No molecular therapy options available.

# ly_case_01

Age: 62

ECOG: 1

Primary diagnosis:
Primary testicular DLBCL ID 01/26

Secondary diagnoses / Comorbidities:
None

Treatment and course:

Question for the tumor board:
Treatment initiation?

Tumor board decision:
Orchiectomy of the right testis. 6 cycles of R-CHOP and 2 cycles of HD-MTX with adjuvant radiation therapy of the left testis / if applicable bilateral orchiectomy.

# ly_case_02

Age: 63

ECOG: 1

Primary diagnosis:
Diffuse large B-cell lymphoma (DLBCL)(NOS)

Secondary diagnoses / Comorbidities:
Allergies: none
Chronic kidney disease Stage 3a
Treatment and course:

Question for the tumor board:
Treatment initiation?
Tumor board decision:
Treatment initiation with 6x Pola-R-CHP with an IPI of 4.

# ly_case_03

Age: 48

ECOG: 0

Primary diagnosis:
Extranodal marginal zone B-cell lymphoma of mucosa-associated lymphoid tissue (MALT lymphoma), Stage I AE, ID 06/2025
- Nodal involvement: none;
-Extralymphatic involvement: stomach (H. pylori positive)
- B symptoms: none
- Risk factors: extranodal involvement, elevated LDH

Secondary diagnoses / Comorbidities:
- Type 2 diabetes mellitus
- Hypothyroidism

Treatment and course:

Question for the tumor board:
Recommended management?

Tumor board decision:
Initially H. pylori eradication. EGD follow-up in 3 months. Depending on response (macroscopic, histologic, and clinical) evaluation Radiation therapy versus W&W.

# ly_case_04

Age: 28

ECOG: 0

Primary diagnosis:
Aggressive B-cell lymphoma, (Primary mediastinal B-cell lymphoma (PMBCL) (ID 05/2022) stage IVBS
aaIPI high: Stage, PS, LDH

Secondary diagnoses / Comorbidities:
None

Treatment and course:

Question for the tumor board:
Further treatment?

Tumor board decision:
Nivo / Brentuximab vedotin, with potential subsequent alloSCT. Alternatively, Glofitamab +/- GemOx.

# ly_case_05

Age: 56

ECOG: 1

Primary diagnosis:
Diffuse large B-cell lymphoma(DLBCL) (Activated B-cell type),
Stage II BE, ID 03/2024
- Nodal involvement: cervical, supraclavicular; Extralymphatic involvement: Mass in paranasal sinuses
- B symptoms: Night sweats, Fever
- Risk factors: extranodal involvement

Secondary diagnoses / Comorbidities:
Arterial hypertension
Type 2 diabetes mellitus

Treatment and course:

Question for the tumor board:
Indication for CAR-T cell therapy in early relapse?

Tumor board decision:
Evaluate re-biopsy. If DLBCL confirmed, CAR-T indication 2nd line (Axicel). Bridging with radiation therapy. Molecular tumor board indication.

# ly_case_06

Age: 61

ECOG: 2

Primary diagnosis:
Diffuse large B-cell lymphoma (DLBCL-NOS, GCB type), ID 12/2021 stage IV A (pulmonary involvement); IPI high (LDH 500, age, stage, performance status, extranodal sites), transformed from a

Splenic marginal zone lymphoma, ID 12/2021 stage IVAE
Nodal involvement: Spleen; Extralymphatic involvement: Liver
Risk factors: extranodal involvement

Secondary diagnoses / Comorbidities:
None

Treatment and course:

Question for the tumor board:
Recommended management?

Tumor board decision:
Recommendation for BiTE (Glofitamab versus Epcoritamab). Re-biopsy (persistent CD20 expression?) to be pursued. Molecular tumor board indication.

# ly_case_07

Age: 62

ECOG: 1

Primary diagnosis:
Diffuse large B-cell lymphoma (DLBCL) arising from follicular lymphoma (Follicular large B-cell lymphoma (FLBL) per updated WHO classification), ID 12/2020 stage IVA

Secondary diagnoses / Comorbidities:
Arterial hypertension

Treatment and course:

Question for the tumor board:
Recommended management?

Tumor board decision:
Recommendation for Glofitamab.

# ly_case_08

Age: 58

ECOG: 1

Primary diagnosis:
Follicular lymphoma (C82.1-4), ID 11/2020
Nodal involvement: abdominal, cervical, axillary, mediastinal
Bone marrow infiltration
Risk factors: Bulk > 10 cm
FLIPI: 4 (advanced stage, elevated LDH, Anemia Hb <12 g/dl, >4 lymph node regions involved)

Secondary diagnoses / Comorbidities:
Arterial hypertension
Hypothyroidism

Treatment and course:

Molecular genetic findings

Question for the tumor board:
Pathogenic variant? Targeted therapy?

Tumor board decision:
Recommendation for EZH2 inhibitor therapy (Tazemetostat).

# ly_case_09

Age: 70

ECOG: 2

Primary diagnosis:

Secondary diagnoses / Comorbidities:
Arterial hypertension
Type 2 diabetes mellitus

Treatment and course:

Molecular genetic findings

Question for the tumor board:
Pathogenic variant? Targeted therapy?

Tumor board decision:
Pathogenic variant: has been described in studies of Mosunetuzumab + Polatuzumab therapy as effective. Alternatively, enrollment in CD79B-ADC trial.

# ly_case_10

Age: 50

ECOG: 2

Primary diagnosis:
Diffuse large B-cell (non-Hodgkin) lymphoma, ID 04/2023
EBV-positive
Stage: IVBE+S
Manifestations: bipulmonary, mediastinal/hilar, pleural, bilateral renal, splenic, retroperitoneal, inguinal, osseous, bone marrow
IPI: 2 (stage, extranodal manifestations)
CNS-IPI: 3 (stage, extranodal manifestation, renal involvement)

Secondary diagnoses / Comorbidities:
Hashimoto thyroiditis

Treatment and course:

Molecular genetic findings

Question for the tumor board:
Pathogenic variant? Targeted therapy?

Tumor board decision:
Venetoclax-based therapy (e.g. ViPOR).

# ly_case_11

Age: 55

ECOG: 0

Primary diagnosis:
Hairy cell leukemia ID 01/2026
Core needle biopsy left humerus: Extranodal manifestation proximal left humerus
BRAFV600E positive
BM biopsy 01.01.2026: no evidence of infiltration
PB 01.01.2026: Hb: 15 g/dl; L: 4,8 G/l; Thr. 280 G/l; LDH 150 U/l

Secondary diagnoses / Comorbidities:
None

Treatment and course:
None

Question for the tumor board:
Recommended management?

Tumor board decision:
Humerus biopsy confirming HCL. No further evidence in bone marrow biopsy. Currently no further treatment indication. Recommendation for regular follow-up.

# ly_case_12

Age: 65

ECOG: 1

Primary diagnosis:
Marginal zone lymphoma ID 06/2025
Stage: Ann-Arbor III
Secondary diagnoses / Comorbidities:
None

Treatment and course:
Since 06/25 Z1-Z4 Rituximab mono
Currently: PD. Plasma cell differentiation with complete loss of CD20

Question for the tumor board:
Recommended management?

Tumor board decision:
In case of progression and CD20 loss, recommendation to switch therapy to Zanubrutinib.

# ly_case_13

Age: 30

ECOG: 1

Primary diagnosis:
Hodgkin lymphoma ID 01/2026
Pathology: classical Hodgkin lymphoma
Stage: Ann Arbor IIBE
CT head/thorax/abdomen: Mediastinal mass

Secondary diagnoses / Comorbidities:
None

Treatment and course:
None

Question for the tumor board:
Recommended management?

Tumor board decision:
Initial diagnosis of classical Hodgkin lymphoma. For stage IIBE, recommendation for BrECADD. In case of PET positivity, radiation therapy to follow.

# ly_case_14

Age: 61

ECOG: 1

Primary diagnosis:
MALT lymphoma of the stomach ID 01/25
Stage IEA
Biopsy stomach: ID MZL: H. pylori negative
CT head/thorax/abdomen: No further manifestation
Secondary diagnoses / Comorbidities:
None
Treatment and course:
01/2025: H. pylori eradication
12/2025: EGD with biopsy: MALT lymphoma confirmed
12/2025: Radiation therapy (low-dose) stomach with 30 Gy
Question for the tumor board:
Recommended management?
Tumor board decision:
W&W. Repeat EGD in 3 months. R-Benda in case of persistence.

# ly_case_15

Age: 40

ECOG: 1

Primary diagnosis:
DLBCL ID 01/2025
Stage IIB, mediastinal bulk
IPI: 2
Secondary diagnoses / Comorbidities:
None
Treatment and course:

Question for the tumor board:
Recommended management?
Tumor board decision:
Early relapse of DLBCL. Recommendation for CAR-T cell therapy. R-DHAP initially to continue.

# ly_case_sim_1

Age: 38

ECOG: 1

Primary diagnosis:
Aggressive B-cell lymphoma, Primary mediastinal large B-cell lymphoma (PMBCL), Initial diagnosis 07/2023, Stage IVBS, aaIPI high (Stage, PS, LDH).
Secondary diagnoses / Comorbidities:
Arterial hypertension
Obesity grade I.
Status post varicella; VZV reactivation under immunotherapy
Treatment and course:
Question for the tumor board:
Further treatment options?
Tumor board decision:
Nivo plus Brentuximab vedotin per CHECKMATE 436 (off label), with potential subsequent alloSCT.

# ly_case_1__ALL

###############################
#### CASE: 1
###############################

## =============================================================== gpt-5-mini (agent) ===============================================================

**===== run_1 =====**

**Tumor board decision:**
Prompt orchiectomy (if not already done) and complete staging including MRI BWK10 and brain MRI + cerebrospinal fluid. Then rapid start of therapy: 6x R-CHOP + 2x systemic HD-MTX as CNS prophylaxis (systemic preferred over only i.th.). Contralateral testicular irradiation recommended after chemo. Unclear BWK10 before therapy via MRI/if necessary. Clarify biopsy.

**Rationale:**
Consistent with guidelines: primary testis DLBCL requires orchiectomy, R-CHOP and HD-MTX due to increased CNS risk. Systemic HD-MTX has a better cerebral effect than just i.th. Contralateral RT reduces local recurrence. BWK10 influences staging/CNS-IPI -> clarification necessary.

**===== run_2 =====**

**Tumor board decision:**
If not already done: immediate radical orchiectomy on the right. Then 6x R-CHOP21 with CNS prophylaxis by 2x systemic HD-MTX (intercalating or sequential). After systemic therapy, irradiation of the contralateral testicle (alternative: bilateral orchiectomy). Clarify BWK-10 using MRI before final staging; Biopsy only if malignancy is highly suspected.

**Rationale:**
Primary DLBCL of the testis has high CNS risk; Guidelines/SOP: Orchiectomy + 6x R-CHOP and systemic HD-MTX are considered standard. Intrathecal prophylaxis alone is usually not enough. Contralateral scrotal irradiation reduces local recurrences. BWK findings can influence staging and should be clarified using MRI (biopsy if necessary).

**===== run_3 =====**

**Tumor board decision:**
Recommendation: If not already done, immediate radical right orchiectomy. Subsequently, systemic first-line therapy 6x R-CHOP21. CNS prophylaxis: 2 cycles of systemic HD-MTX (systemically preferred before/in addition to i.th.; i.th. alone not sufficient). Irradiation of the contralateral testicle/scrotum recommended. BWK 10 lesions should be quickly clarified with MRI; Only consider biopsy if MRI findings are unclear. Carry out staging/basic diagnostics (ECG/echo, laboratory, HBV/HCV/HIV) before starting therapy, do not delay the initiation of therapy unnecessarily.

**Rationale:**
Primary DLBCL of the testis has increased CNS risk; Guidelines/flowchart recommend orchiectomy + 6x R-CHOP and consideration of 2x HD-MTX as CNS prophylaxis. Contralateral testicular irradiation reduces local recurrence. BWK-10 can change staging/CNS-IPI -> MRI for differentiation (biopsy only if bone involvement is suspected).

## =============================================================== gpt-5-mini (plain) ===============================================================

**===== run_1 =====**

**Tumor board decision:**
Recommendation: Radical inguinal orchiectomy on the right to confirm the diagnosis and local control. Then stage-adapted systemic therapy with R-CHOP (6 cycles) plus CNS prophylaxis (HD methotrexate systemically or IT according to an interdisciplinary decision) and prophylactic irradiation of the contralateral testicle (~30 Gy). BWK focal findings (DD hemangioma) radiologically/if necessary. Biopsy clarification before final therapy planning.

**Rationale:**
Primary testicular tumor-DLBCL: Orchiectomy is diagnostically/therapeutically mandatory. High CNS recurrence rate justifies R-CHOP combined with CNS prophylaxis. Contralateral testicular irradiation reduces local recurrences. Unclear BWK findings must be clarified as staging/therapy influences.

===== run_2 =====

**Tumor board decision:**
Immediate radical right inguinal orchiectomy for histological confirmation and local control; complete staging (CT chest/abdomen/pelvis, bone marrow biopsy, CSF cytology +/- cerebral MRI); then rituximab CHOP (6 cycles) with CNS prophylaxis (systemic HD methotrexate if fit or alternatively intrathecal MTX) and prophylactic irradiation of the contralateral testicle (24-30 Gy). T10 focal findings should be clarified by MRI.

**Rationale:**
Primary testicular tumor DLBCL: Orchiectomy is diagnostic/therapeutic standard. High rate of systemic and especially cerebral metastasis justifies R-CHOP + rituximab and targeted CNS prophylaxis. Contralateral testicular irradiation reduces local recurrence. T10 abnormality probably benign -> MRI for clarification.

===== run_3 =====

**Tumor board decision:**
Immediate radical right inguinal orchiectomy for histological confirmation and local control. Then R-CHOP (6 cycles) plus CNS prophylaxis (intrathecal MTX or intercalated/cycle HD-MTX depending on age/comorbidity). Post-chemo prophylactic radiation of the contralateral testis (~24-30 Gy). Before starting therapy: MRI BWK10, bone marrow biopsy, cardiac and infectious clarifications.

**Rationale:**
Primary DLBCL of the testis requires local resection to confirm the diagnosis; R-CHOP is standard systemic therapy. High CNS recurrence rate justifies CNS prophylaxis; Prophylactic irradiation of the contralateral testicle reduces local recurrences. MRI BWK10 and contrast biopsy clarify possible systemic involvement and stage.

## ============================================================ gpt-5-nano (agent) ============================================================

===== run_1 =====

**Tumor board decision:**
Recommendation: Immediate R-CHOP21 system therapy (6 cycles) after orchiectomy of the affected testicle; additional CNS prophylaxis with 2 cycles of HD-MTX and scrotal irradiation of the contralateral testicle. Before starting therapy, carry out brain MRI + CSF FACS for CNS diagnostics, without delaying therapy. Bone marrow status only if there is a therapeutic consequence.

**Rationale:**
Primary testicular DLBCL requires CNS prophylaxis based on subtype/risk strat. CNS diagnostics make sense, but treatment should begin quickly (Flowchart-A4). Radiotherapy improves local control; Continue staging via PET/CT; Bone marrow biopsy only if therapeutically relevant.

===== run_2 =====

**Tumor board decision:**
Recommendation: Immediate first-line treatment with 6xR-CHOP21 + 2xHD-MTX for CNS prophylaxis; Scrotal irradiation of the contralateral testicle after completion of systemic therapy; Orchiectomy on the right has already taken place. Carry out CNS staging (brain MRI + CSF FACS) in parallel, without delaying therapy; Lymphoma conference coordinated.

**Rationale:**
Primary testicular DLBCL is high risk for CNS relapse; CNS prophylaxis makes sense; Therapy corresponds to SOP A4 (testicular tumor-DLBCL); Staging for CNS risk assessment occurs in parallel; Radiotherapy reduces local recurrence; Close lymphoma conference arrangements are recommended.

===== run_3 =====

**Tumor board decision:**
CNS staging before starting therapy: Brain MRI + CSF/FACS due to testicular tumor. If negative: 6x R-CHOP21 + 2x HD-MTX (CNS prophylaxis) + scrotal/contralateral testicular irradiation. If CNS positive: MATRix/CNS-tailored therapy in the lymphoma conference. Orchiectomy was performed.

**Rationale:**
Primary testicular LBCL has high CNS relapse risk; Standard first-line is R-CHOP + systemic HD-MTX with CNS prophylaxis + scrotal irradiation. Before therapy, CNS staging clarifies findings in order to initiate CNS-directed therapy if necessary and minimize the risk of relapse.

## ======================================================== gpt-5-nano (plain) ========================================================

**===== run_1 =====**

**Tumor board decision:**
Radical orchiectomy right; then R-CHOP-6 cycles; CNS prophylaxis; ipsilateral scrotal irradiation as consolidation.

**Rationale:**
Primary testicular tumor DLBCL with high CNS and local recurrence risk; Combination of orchiectomy + systemic R-CHOP reduces risk; CNS prophylaxis recommended; Scrotal irradiation reduces local recurrences.

**===== run_2 =====**

**Tumor board decision:**
Perform radical inguinal orchiectomy (diagnosis/local control) immediately; then systemic R-CHOP-21 x 6 cycles. CNS prophylaxis (intrathecal MTX or HD-MTX depending on risk). Contralateral testicular irradiation only if there are residual findings or a high risk of relapse.

**Rationale:**
DLBCL of the testis requires local control plus systemic therapy; The risk of CNS relapse is high, so CNS prophylaxis makes sense. Radiotherapy of the remaining testicle only if there are residual findings/high risk. Goals: Aim for remission and keep CNS territory protected.

**===== run_3 =====**

**Tumor board decision:**
Radical orchiectomy as a first step; then R-CHOP 6 cycles with CNS prophylaxis; Consolidation by scrotal RT depending on staging.

**Rationale:**
DLBCL of testicular tumor is highly aggressive with high risk of relapse and CNS; Orchiectomy ensures tissue/diagnosis and local control; R-CHOP standard; CNS prophylaxis recommended; RT improves local control.

## ======================================================== gpt-oss-20b (agent) ========================================================

**===== run_1 =====**

**Tumor board decision:**
Start 6x R-CHOP21 (21-day cycle) plus 2 cycles high-dose MTX for CNS prophylaxis. Perform unilateral orchiectomy of the involved testis and irradiate the contralateral testis (or consider bilateral orchiectomy).

**Rationale:**
Flowchart A4 (Primary DLBCL of the testis) recommends 6x R-CHOP21 + 2x HD-MTX, orchiectomy, and contralateral testis irradiation for optimal local control and CNS prophylaxis. No prior therapy, patient fit - standard regimen applies.

**===== run_2 =====**

**Tumor board decision:**
Primary therapy: right-sided orchiectomy, 6x R-CHOP21 (21-day interval) plus 2x high-dose MTX (CNS prophylaxis). After completion of radiotherapy of the contralateral testicle (or bilateral orchiectomy) - EOT-PET/CT for evaluation and, if necessary, consolidative radiotherapy.

**Rationale:**
Flowchart section A4 defines primary DLBCL of the testis as 6x R-CHOP21 + 2x HD-MTX, orchiectomy and contralateral radiotherapy. Testicular involvement triggers CNS prophylaxis; hence 2x MTX. EOT-PET/CT is used to decide on further radiation therapy.

**===== run_3 =====**

**Tumor board decision:**
Orchiectomy of the affected testicle, 6x R-CHOP21 + 2x HD-MTX for CNS prophylaxis, followed by irradiation of the contralateral testicle (or bilateral orchiectomy).

**Rationale:**

Primary DLBCL of the testis requires orchiectomy, R-CHOP21 standard, HD-MTX because of CNS risk, radiotherapy of the contralateral testis to reduce recurrence.

## ========================================================== gpt-oss-20b (plain) ==========================================================

**===== run_1 =====**

**Tumor board decision:**
Start R-CHOP (6x) with intratheal methotrexate prophylaxis and then testicular radiotherapy 30Gy. No further therapies necessary as there are no previous treatments.

**Rationale:**
Primary testicular DLBCL has high CNS relapse rates; R-CHOP plus CNS prophylaxis is standard. Testicular radiation reduces local recurrences. Patient is fit for therapy, no contraindications.

**===== run_2 =====**

**Tumor board decision:**
Start R-CHOP (6x) plus CNS prophylaxis (intrathecal MTX or high-dose systemic MTX). Orchiectomy is assumed completed; if not, perform right orchiectomy first. Consider prophylactic RT to contralateral testis per institutional policy.

**Rationale:**
Primary testicular DLBCL has high CNS relapse risk; R-CHOP is standard first-line. CNS prophylaxis reduces relapse. Patient has no prior therapy or contraindications, making this regimen optimal.

**===== run_3 =====**

**Tumor board decision:**
Begin R-CHOP (6x) plus CNS prophylaxis (intrathecal MTX or high-dose MTX). If residual testicular disease or high risk, add 30Gy testicular RT after chemo.

**Rationale:**
Standard first-line for primary testicular DLBCL; high CNS relapse risk warrants prophylaxis; no prior therapy; no contraindications.

## ========================================================== gpt-oss-120b (agent) ===========================================================

**===== run_1 =====**

**Tumor board decision:**
Primary testicular DLBCL: right orchiectomy, then 6xR-CHOP21 with 2 cycles of high-dose methotrexate (HD-MTX) as intrathecal CNS prophylaxis. After chemotherapy, 30Gy involved field radiation of the contralateral testicle. The BWK 10 abnormality (suspected hemangioma) is observed, no therapy.

**Rationale:**
Testicular localization increases CNS risk -> HD-MTX recommended. Guidelines (A4) stipulate 6xR-CHOP + HD-MTX, orchiectomy and testis radiation. T10 hemangioma is asymptomatic and does not affect lymphoma therapy.

**===== run_2 =====**

**Tumor board decision:**
Perform an orchiectomy of the affected testicle, then 6 cycles of R-CHOP21 plus 2 cycles of high-dose methotrexate (3g/m2) as systemic CNS prophylaxis. After chemotherapy, 24Gy involved field radiation of the contralateral testicle. No intrathecal prophylaxis, the BWK 10 lesion (suspected hemangioma) does not require any therapy.

**Rationale:**
Primary testicular DLBCL has a high CNS risk; The guideline flowchart recommends HD-MTX prophylaxis for testicular infestation. Standard first-line therapy is 6R-CHOP. Orchiectomy and radiation of the counter-testis reduce recurrence rates. The thoracic BWK 10 finding is probably benign and does not influence therapy.

**===== run_3 =====**

**Tumor board decision:**

In primary testicular DLBCL, start 6xR-CHOP21 immediately, after cycles 2 and 4 1-2 cycles of HD-MTX (3.5g/m2) as systemic CNS prophylaxis. Perform orchiectomy of the affected testicle, then 24Gy involved field radiation of the contralateral testicle. The BWK 10 abnormality is classified as a benign hemangioma - no therapy, only radiological control.

**Rationale:**
Testicular lymphomas have a high CNS risk; Flowchart A4 recommends R-CHOP + HD-MTX prophylaxis and testis radiation. Intrathecal MTX is not necessary when systemic HD-MTX is given. The thoracic lesion corresponds to a benign hemangioma, therefore no oncological intervention.

## ============================================================ gpt-oss-120b (plain) ============================================================

**===== run_1 =====**

**Tumor board decision:**
Start systemic therapy with R-CHOP (rituximab, cyclophosphamide, doxorubicin, vincristine, prednisone) for 6 cycles combined with CNS prophylaxis (intrathecal or high-dose methotrexate). After completion of chemotherapy, local involvement field radiotherapy (~30Gy) is carried out on the affected testicle.

**Rationale:**
Primary testicular DLBCL is a high-risk lymphoma with a high rate of CNS infiltration. R-CHOP is standard chemo, rituximab improves response. CNS prophylaxis reduces the risk of brain and spinal cord metastases. Scrotal radiotherapy improves local control.

**===== run_2 =====**

**Tumor board decision:**
Start systemic therapy with 6 cycles of R-CHOP (rituximab, cyclophosphamide, doxorubicin, vincristine, prednisone) combined with cerebral prophylaxis (intrathecal methotrexate +/- high-dose systemic MTX). After completion of chemotherapy, involved-field radiotherapy (30Gy) is carried out on the affected testicle and, if necessary, prophylactically on the contralateral testicle.

**Rationale:**
Primary DLBCL of the testis is a highly aggressive non-Hodgkin lymphoma with a high risk of cerebral metastases. R-CHOP is standard first-line regime; additional CNS prophylaxis reduces the risk of leptomeningeal infiltration. Local radiotherapy improves local control after chemotherapy.

**===== run_3 =====**

**Tumor board decision:**
Start systemic therapy with R-CHOP (rituximab, cyclophosphamide, doxorubicin, vincristine, prednisone) for 6-8 cycles, combined with CNS prophylaxis (intrathecal or high-dose methotrexate). After completion of chemotherapy, local testicular radiotherapy (30Gy) should be given to reduce the risk of recurrence.

**Rationale:**
Primary DLBCL of the testis is an aggressive, extranodal lymphoma with a high risk of CNS and testicular recurrences. R-CHOP is standard first-line therapy; CNS prophylaxis reduces the occurrence of brain/spinal cord involvement. Local radiotherapy after chemo improves the local control rate.

## ============================================================== qwen3-32b (agent) ============================================================

**===== run_1 =====**

**Tumor board decision:**
Start of therapy with 6x R-CHOP21 + 2x HD-MTX (CNS prophylaxis) after orchiectomy. Consider post-irradiation of the contralateral testicle.

**Rationale:**
Primary testicular DLBCL stage I requires standardized high-intensity therapy according to Flowchart-A4. Focal lymph node excess absorption is relevant to therapy in the testicular DLBCL context. Orchiectomy is a prerequisite for R-CHOP.

**===== run_2 =====**

**Tumor board decision:**

Consider starting therapy with 6x R-CHOP21, including 2x HD-MTX (CNS prophylaxis), orchiectomy of the affected testicle and follow-up irradiation of the contralateral testicle.

**Rationale:**
Primary testicular DLBCL requires 6x R-CHOP21 with CNS prophylaxis (2x HD-MTX) after SOP. Orchiectomy is mandatory, and follow-up irradiation of the healthy testicle to prevent secondary lesions. Focal inguinal lymph node activation is clarified as part of PET/CT staging.

**===== run_3 =====**

**Tumor board decision:**
Start therapy with 6x R-CHOP21 + 2x HD-MTX (CNS prophylaxis), perform orchiectomy of the affected testicle and consider follow-up irradiation of the contralateral testicle.

**Rationale:**
Primary testicular DLBCL (A4 flowchart) requires R-CHOP21 with HD-MTX due to high CNS risk. Orchiectomy is diagnostically and therapeutically relevant. PET/CT shows focal lymph node infiltration, which is considered in testicular DLBCL therapy.

## ======================================================== qwen3-32b (plain) ========================================================

**===== run_1 =====**

**Tumor board decision:**
Recommendation: Initiation of therapy with R-CHOP (rituximab + CHOP chemotherapy) followed by involved field radiation therapy. Biopsy confirmation required.

**Rationale:**
Stage I primary testicular DLBCL requires standardized aggressive therapy. R-CHOP is gold standard for localized DLBCL. Radiation therapy optimizes local control results. No contraindications or resistance factors known.

**===== run_2 =====**

**Tumor board decision:**
Recommendation: initiation of therapy with R-CHOP (rituximab + CHOP) including central nervous system prophylaxis (e.g. high-dose methotrexate).

**Rationale:**
Primary testicular DLBCL requires prompt therapy. R-CHOP is standard for localized aggressive B-cell lymphomas. CNS prophylaxis is indicated for testicular lymphoma due to increased rates of CNS involvement.

**===== run_3 =====**

**Tumor board decision:**
Recommendation: Initiation of therapy with R-CHOP (6 cycles) followed by localized radiotherapy to the testicles and regional lymph nodes.

**Rationale:**
Stage I primary DLBCL of the testis requires systemic chemotherapy (R-CHOP) to reduce micrometastases and subsequent local radiation for local control. The combination is standard for localized DLBCL and optimizes recurrence-free survival.

## ======================================================== qwen3-next-80b (agent) ========================================================

**===== run_1 =====**

**Tumor board decision:**
Carry out CNS diagnostics (MRI skull + CSF FACS), then initiate 6x R-CHOP21 + 2 cycles of HD-MTX for CNS prophylaxis. Radiate the contralateral testicle after therapy.

**Rationale:**
Primary DLBCL of the testis is a CNS risk factor (CNS-IPI trigger). CNS diagnostics are mandatory. If the result is negative, R-CHOP21 + HD-MTX is standard for CNS prophylaxis. Contralateral radiation reduces the risk of recurrence.

**===== run_2 =====**

**Tumor board decision:**
Before initiating therapy, carry out CNS diagnostics (MRI skull + CSF FACS). If the result is negative: R-CHOP21 + 2 cycles of HD-MTX (CNS prophylaxis), orchiectomy of the affected testicle and follow-up irradiation of the contralateral testicle.

**Rationale:**
Primary DLBCL of the testis is a CNS risk factor (CNS-IPI trigger). According to the flowchart (A4), CNS diagnostics are mandatory before therapy. If the result is negative, HD-MTX prophylaxis, orchiectomy and contralateral irradiation are standard. PET/CT alone is not sufficient for CNS staging.

**===== run_3 =====**

**Tumor board decision:**
Orchiectomy of the right testicle, CNS diagnostics (MRI skull + CSF FACS), then 6x R-CHOP21 + 2x HD-MTX (CNS prophylaxis), followed by irradiation of the contralateral testicle.

**Rationale:**
Primary DLBCL of the testis (A4 flowchart) requires orchiectomy, CNS diagnosis due to testicular involvement (CNS-IPI trigger), R-CHOP21 + HD-MTX for CNS prophylaxis and irradiation of the contralateral testis for recurrence prevention. No comorbidities mentioned, hence standard therapy.

## ============================================================ qwen3-next-80b (plain) ============================================================

**===== run_1 =====**

**Tumor board decision:**
Initiate R-CHOP chemotherapy (6 cycles) with CNS prophylaxis (intrathecal methotrexate or high-dose systemic methotrexate).

**Rationale:**
Primary testicular DLBCL requires standard R-CHOP plus CNS prophylaxis due to the high risk of CNS relapse. Orchiectomy is required as a diagnostic and therapeutic step. No contraindications or prior therapy, therefore immediate initiation.

**===== run_2 =====**

**Tumor board decision:**
Radical inguinal orchidectomy followed by R-CHOP chemotherapy with central nervous system prophylaxis (intrathecal methotrexate or high-dose methotrexate)

**Rationale:**
Primary testicular DLBCL requires surgical removal of the testicle. Because of the high risk of CNS recurrence, prophylaxis is necessary. R-CHOP is standard therapy for DLBCL. No contraindications due to secondary diagnoses.

**===== run_3 =====**

**Tumor board decision:**
Initiate R-CHOP chemotherapy with CNS prophylaxis (intrathecal or high-dose methotrexate).

**Rationale:**
Primary testicular DLBCL requires systemic therapy with R-CHOP and mandatory CNS prophylaxis due to the high risk of CNS recurrence. Orchiectomy as confirmation of the diagnosis and local therapy is carried out as standard.

# ly_case_2__ALL

################################
#### CASE: 2
################################

## ========================================================== gpt-5-mini (agent) ==========================================================

**===== run_1 =====**

**Tumor board decision:**
Curative-intended first-line therapy is recommended. In fit patients: 6x Pola-R-CHP (preference for IPI 2-5); if not available/contraindicated: 6x R-CHOP21. No routine CNS prophylaxis.

**Rationale:**
IPI 3, LVEF 58% allowed anthracycline; SOP/Flowchart recommends 2-5 Pola-R-CHP for IPI. advance. CNS-IPI ~3 (renal involvement, LDH↑, stage IV) -> no indication for intrathecal or HD-MTX prophylaxis. CKD 3a increases the risk of HD-MTX, therefore additionally contraindicated without a clear CNS risk factor.

**===== run_2 =====**

**Tumor board decision:**
Recommendation: Start curatively intended systemic therapy. For fit patients: 6x Polatuzumab-R-CHP (preferred option); alternatively 6x R-CHOP. No routine CNS prophylaxis (CNS IPI prerequisite 3). MRI skull + CSF only if symptoms or CNS-IPI >=4. Pay attention to cardiac monitoring and renal adjustment.

**Rationale:**
DLBCL NOS, IPI 3 (stage IV, LDH↑, kidney involvement). Guidelines/Flowchart: Pola-R-CHP preferred for IPI 2-5; LVEF 58% allows anthracycline. CNS prophylaxis is recommended for CNS IPI >=4 or testis/mammary/epidural. CKD3a increases risk of HD-MTX; Intrathecal administration has limited preventive effect for parenchymal CNS disease.

**===== run_3 =====**

**Tumor board decision:**
Start curatively intended systemic therapy: 6x polatuzumab-R-CHP (if not available: 6x R-CHOP). Anthracycline permitted (LVEF 58%). No routine CNS prophylaxis (neither i.th. nor HD-MTX) if estimated CNS-IPI ~3. For neurology or CNS-IPI >=4: MRI skull + CSF. Monitor kidney function closely and make dosage adjustments.

**Rationale:**
DLBCL NOS with IPI 3 -> intensive first-line therapy according to SOP; Pola-R-CHP preferred at IPI 2-5. CNS risk (kidney, LDH, stage) results in CNS-IPI ~3, therefore no indication for prophylactic HD-MTX/IT-MTX; HD-MTX is also contraindicated due to CKD 3a. EOT-PET/CT and review in lymphoma conference.

## ========================================================== gpt-5-mini (plain) ==========================================================

**===== run_1 =====**

**Tumor board decision:**
Initiation of therapy: R-CHOP-21 (rituximab + cyclophosphamide + doxorubicin + vincristine + prednisone) 6 cycles, primary G-CSF prophylaxis. Because of kidney disease: CNS prophylaxis -- if kidney function is sufficient (GFR >=50-60), intercalating high-dose IV methotrexate; otherwise intrathecal methotrexate. Nephro and TLS monitoring.

**Rationale:**
DLBCL, CD20+, Stage IV, IPI 3 - Standard firstline is R-CHOP; LVEF 58% allows anthracycline. CKD 3a allows R-CHOP without general dose reduction, requires monitoring. Kidney involvement increases the risk of CNS recurrence -> targeted CNS prophylaxis. G-CSF due to risk/treatment intensity.

**===== run_2 =====**

**Tumor board decision:**

Initiation of R-CHOP q21d (rituximab + CHOP) as first-line, planned 6 cycles (response dependent). Before starting: HBV serology, TLS prophylaxis (hydration, allopurinol/possibly rasburicase), baseline laboratory including creatinine, ECG/echo. Check CNS risk via CNS-IPI; If the risk is high, consider IT prophylaxis or systemic HD-MTX after renal function assessment.

**Rationale:**
Treatment-naïve CD20+ DLBCL St.IV with IPI3 - R-CHOP is standard firstline and possible with LVEF 58%. Kidney involvement increases tumor lysis and CNS risk, therefore TLS prophylaxis, close monitoring and HBV check before rituximab. CKD3a requires caution/dose review with HD methotrexate.

**===== run_3 =====**

**Tumor board decision:**
Initiation of therapy: R-CHOP-21 (6 cycles in total) with primary G-CSF prophylaxis. Check HBV serology and vaccination status before rituximab. Intermediate PET after 2 cycles. Because of kidney involvement and IPI 3: CNS prophylaxis - if the kidney function is sufficient, systemic HD methotrexate (e.g. 3 g/m2 i.v. between cycles 2/3 and 5/6), otherwise intrathecal prophylaxis. Nephrological monitoring and dose adjustment if necessary.

**Rationale:**
New, untreated CD20+ DLBCL Stage IV - R-CHOP is standard. CKD 3a usually allows R-CHOP but requires monitoring/dose adjustment. Kidney involvement and increased IPI increase CNS risk, therefore consider prophylaxis. G-CSF reduces febrile neutropenia; HBV screening necessary because of rituximab; Intermediate PET for therapy control.

## ============================================================ gpt-5-nano (agent) ============================================================

**===== run_1 =====**

**Tumor board decision:**
Recommendation: First-line therapy with R-CHOP-6 (R-CHOP-21) for advanced DLBCL NOS with spleen/kidney involvement, LDH↑, IPI 3. DA-EPOCH-R is not preferable due to CKD3a/side effects and no double hit. CNS prophylaxis only examined in study; Note PET-controlled consolidation.

**Rationale:**
R-CHOP-6 is established and better tolerated in CKD 3a; MYC-IHC pos without translocation has no clear advantage for DA-EPOCH-R; CNS prophylaxis not standard outside studies; Spleen/kidney involvement increases CNS risk, prophylaxis only makes sense in the studio context.

**===== run_2 =====**

**Tumor board decision:**
First-line for advanced DLBCL NOS (St IV, IPI 3, MYC-IHC+): 6 cycles of Pola-R-CHP (Pola replaces vincristine). Pay attention to CKD 3a, adjust dose if necessary. CNS prophylaxis per CNS-IPI (consider if >=4 intrathecal MTX). Interim PET after 2 cycles to determine duration of therapy. include lymphoma conference; review clinical trials.

**Rationale:**
MYC-IHC+ without DH; DA-EPOCH-R has no clear advantage in unselected DLBCL; Pola-R-CHP shows advantages in the POLARIX trial; CKD 3a increases Tox; Spleen/kidney involvement increases risk; CNS prophylaxis makes sense if the CNS IPI is high; close monitoring via interim PET.

**===== run_3 =====**

**Tumor board decision:**
DA-R-EPOCH-R as first-line therapy for advanced DLBCL NOS (stage IV, spleen and kidney involvement, LDH↑, IPI 3, MYC-IHC+) with CNS prophylaxis (intrathecal MTX; CKD3a-adapted dose). 6 cycles, interim and final PET for residual storage; Radiotherapy only for residual bulk after EOT-PET.

**Rationale:**
High-risk phenotype (IPI 3, spleen/kidney involvement, LDH↑, MYC-IHC+) -> DA-R-EPOCH-R enables better remissions than R-CHOP with suitable fitness. CNS risk high -> prophylaxis makes sense. CKD3a limits high-dose MTX; prefers IT prophylaxis. Close lymphoma conference for progression.

## ============================================================ gpt-5-nano (plain) ============================================================

**===== run_1 =====**

**Tumor board decision:**
CAR T-cell therapy (CD19) as the next therapeutic step for relapsed/refractory DLBCL after previous treatment; Bridge therapy with BR or GemOx if waiting; Check CAR-T availability.

**Rationale:**
MYC-positive, highly aggressive DLBCL (Ki-67 75-85%), IPI 3; no double/triple hit. CAR-T offers highest remission rate in R/R DLBCL. LVEF 58% and CKD IIIa; no contraindications. Bridge makes sense to achieve disease control through CAR-T.

**===== run_2 =====**

**Tumor board decision:**
CAR-T cell therapy (axicabtagene ciloleucel or tisagenleucel) as the next therapeutic step for refractory/stage IV DLBCL after Frontline. suitability assessment; Bridging therapy (e.g. reduced BR or R-ICE) for remission/ASCT bridge, then CAR-T.

**Rationale:**
Very high risk (IPI 3, B symptoms, multisystem involvement including spleen, MYC+; Ki-67 75-85%), progression to frontline. CAR-T offers the best chance of long-term remission. Autologous transplantation as an alternative, but CAR-T preferred; CKD 3a requires dose-adjusted bridging therapy.

**===== run_3 =====**

**Tumor board decision:**
CAR T-cell therapy (axicabtagene ciloleucel or lisocabtagene maraleucel) as second-line in refractory/relapsed DLBCL; Bridging if necessary If CAR-T is not available or contraindicated: Polatuzumab vedotin + bendamustine + rituximab (Pola-BR) as bridging/salvage with ASCT option.

**Rationale:**
High risk biology (MYC+, Ki-67 75-85%), earlier progression after first line. CAR-T offers best remission rates and cure potential in R/R DLBCL. Splenic extranodal findings do not influence prognosis against CAR-T; CKD 3a limits nephrotoxic options; quick treatment needed. If CAR-T is not possible promptly: Pola-BR/Salvage+ASCT.

## ============================================================== gpt-oss-20b (agent) ==============================================================

**===== run_1 =====**

**Tumor board decision:**
Start 6x Pola-R-CHP (fit patient, IPI3, CKD3a, without anthracycline exposure).

**Rationale:**
Flowchart B1: IPI2-5 -> 6x Pola-R-CHP. Polatuzumab + no doxorubicin sparing cardiac risk, tolerable in CKD3a. CNS-IPI>=4 -> CNS diagnostics, but no prophylaxis necessary. 6 cycles with EOT-PET/CT and, if necessary, radiation after conference.

**===== run_2 =====**

**Tumor board decision:**
Start 6x Pola-R-CHP (PolatuzumabVedotin+R-CHOP without vincristine). For CKD3a, cyclophosphamide 75% dose, doxorubicin 75% dose, prednisolone 100mgq.d. 5d. CNS prophylaxis not necessary (CNS IPI>=4, but MRI+LP negative).

**Rationale:**
DLBCL-NOS, IPI3, fit, CKD3a. Flowchart B1: IPI2-5 -> 6x Pola-R-CHP. Renal restriction requires 25% dose reduction. CNS-IPI>=4 -> MRI+LP; negative -> no routine prophylaxis. Pola-R-CHP has better CR rates in IPI>=2 and tolerable in CKD.

**===== run_3 =====**

**Tumor board decision:**
Start 6x Pola-R-CHP (polatuzumab vedotin + rituximab + cyclophosphamide + doxorubicin + prednisone) in 21-day cycles. CKDIIIa -> cyclophosphamide dose adjustment, doxorubicin unchanged. CNS-IPI>=4, but no testicular/mammary involvement -> no routine CNS prophylaxis.

**Rationale:**
DLBCL-NOS, IPI3, fit, CKDIIIa, no contraindications to Pola-R-CHP. Flowchart B1 recommends 6x Pola-R-CHP for IPI2-5. CNS-IPI>=4 requires only diagnostics; If the result is negative, no

prophylactic MTX administration.

## ========================================================== gpt-oss-20b (plain) ==========================================================

**===== run_1 =====**

**Tumor board decision:**
Start R-CHOP-14 (R-CHOP with 14-day interval) with renal-adapted cyclophosphamide (~500mg/m2) and G-CSF support. Rituximab + CHOP standard for DLBCL IV, IPI3, MYC positive, without double hit. Regular renal evaluation and LDH monitoring.

**Rationale:**
Standard first line for DLBCL IV, IPI3. R-CHOP proven, MYC positive without double hit remains R-CHOP. Renal insufficiency requires cyclophosphamide reduction. 14-day interval increases effectiveness with high IPI. G-CSF prevents neutropenia. No CNS risk -> no intrathecal. Regular checks ensure safety.

**===== run_2 =====**

**Tumor board decision:**
Start R-CHOP (rituximab+cyclophosphamide750mg/m2+doxorubicin25mg/m2+vincristine1.4mg/m2+prednisolone100mg/m2) 21-day q cycle, 6 cycles, with renal-adapted cyclophosphamide and doxorubicin. Monitor LVEF and renal function regularly. If the response is inadequate after 3 cycles: R-CHOP-14 or R-CHOEP (with dose-reduced etoposide).

**Rationale:**
DLBCLIV, IPI3, MYC positive, no double hit FISH. Standard R-CHOP is the first choice. CKD3a requires cyclophosphamide and doxorubicin dose reduction (750mg/m2 and 25mg/m2, respectively) to avoid renal and cardiac toxicity. 6 cycles at 21 day intervals are evidence-based. If response is poor, more intensive R-CHOP-14 or R-CHOEP may be considered, with dose reduction of etoposide to minimize renal burden.

**===== run_3 =====**

**Tumor board decision:**
Start R-CHOP (Rituximab 375mg/m2 + Cyclophosphamide 75% of the standard dose, Doxorubicin 50mg/m2, Vincristine 1.4mg/m2, Prednisolone 100mg/m2) every 14 days for 6 cycles, with constant monitoring of kidney function and further dose adjustment if necessary.

**Rationale:**
Standard therapy for DLBCLIV without double hit gene; for CKD3a, cyclophosphamide dose must be reduced by 25%; R-CHOP-14 offers optimal effectiveness and tolerable renal stress in aggressive disease.

## ========================================================== gpt-oss-120b (agent) ==========================================================

**===== run_1 =====**

**Tumor board decision:**
First-line therapy with 6 cycles of Pola-R-CHP (polatuzumab-vedotin+rituximab+cyclophosphamide+vincristine+prednisone) with dose adjustment for CKD3a. Before therapy, CNS staging (MRI+cerebrospinal fluid); If the result is negative, no routine CNS prophylaxis.

**Rationale:**
IPI3 and MYC positive without double hit defines high-risk DLBCL. Due to moderate renal insufficiency and possible cardiac stress, an anthracycline-free regimen (Pola-R-CHP) that meets the guideline standard for IPI2-5 is preferred. CNS-IPI >=4 requires diagnostic clarification, prophylactic therapy only if proven.

**===== run_2 =====**

**Tumor board decision:**
CNS staging (MRI+CSF). If the result is negative, 6 cycles of Pola-R-CHP as first-line therapy (alternatively 6 cycles of R-CHOP with liposomal doxorubicin if intolerance).

**Rationale:**

IPI3, MYC positive, no double hit, kidney and spleen involvement, CKD3a -> Pola-R-CHP (IPI2-5 recommendation) avoids anthracycline toxicity. CNS-IPI>=4 requires MRI+CSF, no prophylactic HD-MTX if the result is negative.

**===== run_3 =====**

**Tumor board decision:**
First-line therapy with 6 cycles of Pola-R-CHP (polatuzumabvedotin+rituximab+cyclophosphamide+doxorubicin+prednisone) with G-CSF support. Before therapy, CNS IPI risk assessment (MRI+cerebrospinal fluid); If the result is negative, no prophylactic HD-MTX.

**Rationale:**
DLBCL-NOS, IPI3, MYC-positive without double hit, fit despite kidney stage 3a. According to flowchart B1-IPI2-5, Pola-R-CHP is preferred. Kidney and spleen involvement increases CNS IPI >=4 -> CNS diagnostics required, prophylactic MTX only if the result is positive.

## ============================================================ gpt-oss-120b (plain) ============================================================

**===== run_1 =====**

**Tumor board decision:**
Start first-line immunochemotherapy with R-CHOP21, reducing cyclophosphamide and doxorubicin by approximately 25% (CKD3a). Supplement prophylactic G-CSF and closely monitor renal and hematology parameters.

**Rationale:**
DLBCL stage IV, IPI3, CD20 positive, no previous therapy. R-CHOP is standard curative; due to renal insufficiency, the dose is adjusted to reduce toxicity. No double hit lymphoma, therefore no more intensive regimen necessary. The aim is remission with an acceptable side effect profile.

**===== run_2 =====**

**Tumor board decision:**
Starting first-line therapy with R-CHOP21 (rituximab, cyclophosphamide, doxorubicin, vincristine, prednisone) for 6 cycles, dose adjustment of cyclophosphamide and doxorubicin due to renal insufficiency3a, G-CSF support, close monitoring of kidney and heart function.

**Rationale:**
DLBCL, IPI3, CD20 positive, no double hit mutation, no previous therapy. R-CHOP is standard first-line; if IPI is high, R-DA-EPOCH can be considered, but renal insufficiency increases the risk of toxic side effects. Dose reduction enables safe use.

**===== run_3 =====**

**Tumor board decision:**
Start 6-cycle R-CHOP therapy (rituximab, cyclophosphamide, doxorubicin, vincristine, prednisone) with 21-day intervals. Reduce cyclophosphamide by 25% (eGFR ~45ml/min) and monitor renal and cardiac function; give prophylactic G-CSF.

**Rationale:**
DLBCL-NOS, StageIV, IPI3, CD20-positive, MYC-protein positive without double hit. R-CHOP remains standard first-line; Intensive regimen (DA-EPOCH) offers no clear advantage and increases the risk in CKD3a. Dose adjustment protects the kidneys, G-CSF reduces neutropenia-related complications.

## ============================================================ qwen3-32b (agent) ============================================================

**===== run_1 =====**

**Tumor board decision:**
Recommendation: 6x Pola-R-CHP in fit patients with IPI 3 and MYC positivity (no double/triple hit).

**Rationale:**
IPI 3 (high risk), MYC positivity (not double hit) and kidney involvement. Pola-R-CHP is SOP for IPI 2-5. EPOCH-R not indexed because no double/triple hit. Cardiac reserve sufficient (LVEF 58%).

**===== run_2 =====**

**Tumor board decision:**
Recommendation: 6x R-EPOCH for MYC-positive DLBCL (no double/triple hit) with IPI 3, B symptoms and kidney involvement

**Rationale:**
High IPI (3), MYC positivity and kidney involvement as risk factors. R-EPOCH is preferred in individual cases in MYC-positive DLBCL because it is more intensive therapy in high-risk profiles. Chronic renal failure requires dosage adjustment. PET/CT after therapy determines consolidative measures.

**===== run_3 =====**

**Tumor board decision:**
Recommendation: 6x R-EPOCH therapy for DLBCL stage IV with IPI 3, MYC positivity and kidney involvement

**Rationale:**
MYC positivity (although not a double/triple hit) and IPI 3 (high risk) warrant more intensive therapy. R-EPOCH provides better control in high-risk DLBCL. Stage 3a renal failure allows standard anthracycline dose (EF 58%).

## ============================================================ qwen3-32b (plain) ============================================================

**===== run_1 =====**

**Tumor board decision:**
Recommendation: R-CHOP with dose adjustment for cyclophosphamide and doxorubicin (considers renal insufficiency).

**Rationale:**
High-risk DLBCL (IPI 3, MYC positive) requires R-CHOP. Renal insufficiency (stage 3a) requires dose reduction of nephrotoxic components (doxorubicin, cyclophosphamide). EPOCH regimen would be alternative but carries higher risks of toxicity. B symptoms and progression warrant aggressive therapy with a modified standard protocol.

**===== run_2 =====**

**Tumor board decision:**
Recommendation: R-GemOx (rituximab + gemcitabine + oxaliplatin) as modified induction therapy taking kidney function into account. Subsequent evaluation for CAR-T cell therapy in response.

**Rationale:**
High-risk DLBCL (IPI 3) with MYC positivity and renal insufficiency. R-GemOx is more nephrotoxic than R-CHOP. CAR-T cells as an option for relapse-free response. No double/triple hit, therefore no specific target therapy required.

**===== run_3 =====**

**Tumor board decision:**
Initiation of therapy with R-EPOCH-T (rituximab, etoposide, prednisolone, cyclophosphamide, doxorubicin, cytarabine) with dose adjustment for renal insufficiency. Concomitantly monitor kidney function and take supportive measures.

**Rationale:**
Stage IV DLBCL with high IPI and MYC positivity requires intensive therapy. R-EPOCH-T is established in high-risk DLBCL. Adjust cyclophosphamide dose due to CKD stage 3a. No contraindications for rituximab. Progressive disease warrants prompt treatment.

## ========================================================== qwen3-next-80b (agent) ==========================================================

**===== run_1 =====**

**Tumor board decision:**
6 cycles of Pola-R-CHP (polatuzumab vedotin + R-CHP) as first-line therapy

**Rationale:**
DLBCL-NOS, IPI 3, no double/triple hit mutation. Pola-R-CHP is standard for IPI 2-5 in fit patients. CKD 3a allows therapy with monitoring, no contraindication. CNS-IPI <4, no CNS prophylaxis necessary.

MYC-IHC positive without a FISH hit does not change standard therapy.

**===== run_2 =====**

**Tumor board decision:**
Carry out CNS diagnostics (MRI skull + CSF FACS). If the result is negative: 6 cycles of Pola-R-CHP with cyclophosphamide dose adjustment for CKD 3a. If the result is positive: MATRix protocol.

**Rationale:**
CNS-IPI 4 (renal involvement, LDH↑, >1 extranodal, ECOG >1), therefore CNS diagnostics required. For IPI 3 and fit patients, Pola-R-CHP is standard, but only after exclusion of CNS involvement. CKD 3a requires cyclophosphamide dose adjustment.

**===== run_3 =====**

**Tumor board decision:**
Carry out CNS diagnostics (MRI skull + CSF FACS). If the result is negative: 6 cycles of Pola-R-CHP with cyclophosphamide dose adjustment for CKD 3a. If the result is positive: MATRix protocol.

**Rationale:**
CNS-IPI >=4 (renal involvement, LDH↑, ECOG>1, stage IV) requires CNS diagnostics. If the result is negative, Pola-R-CHP is standard for IPI 2-5. If the result is positive, MATRix is indicated. CKD 3a requires cyclophosphamide dose adjustment.

## ============================================================ qwen3-next-80b (plain) ============================================================

**===== run_1 =====**

**Tumor board decision:**
Initiate R-CHOP therapy (rituximab, cyclophosphamide, doxorubicin, vincristine, prednisolone) with close monitoring of renal function

**Rationale:**
Standard first line for DLBCL NOS without double/triple hit. CKD 3a allows R-CHOP at standard dose (no renal excretion of vincristine/cyclophosphamide). LVEF 58% and CD20+ speak for rituximab. Renal involvement requires monitoring but no dosage adjustment is necessary.

**===== run_2 =====**

**Tumor board decision:**
Initiate R-CHOP therapy (rituximab, cyclophosphamide, doxorubicin, vincristine, prednisolone) with adjusted cyclophosphamide dose (750 mg/m2) and strict renal function monitoring.

**Rationale:**
Standard first-line therapy for DLBCL NOS without double/triple hit. CKD 3a allows R-CHOP with dose adjustment and monitoring. Kidney disease and LDH elevation are taken into account in IPI, no contraindications for R-CHOP (LVEF 58%, no allergies).

**===== run_3 =====**

**Tumor board decision:**
Initiate R-CHOP therapy with close renal function monitoring and tumor lysis syndrome prophylaxis

**Rationale:**
DLBCL stage IV with IPI 3 requires standard therapy R-CHOP. No double/triple hit, therefore no intensive therapy. CKD 3a allows R-CHOP without dose adjustment, but monitoring is required. Cyclophosphamide metabolism hepatic, no significant renal excretion.

# ly_case_3__ALL

################################
#### CASE: 3
################################

## ================================================================ gpt-5-mini (agent) ================================================================

**===== run_1 =====**

**Tumor board decision:**
Test for Helicobacter pylori (histology/serology/PCR) and eradication if detected. In the case of H. pylori negativity, an eradication attempt is still justified. Perform endoscopic control biopsies; Up to 12 months waiting period for complete remission. In the absence of remission or progression -> involved-site radiotherapy (ISRT) 24-30 Gy. Systemic therapy not primary. Watch and wait possible if there is a response/asymptomatic persistence.

**Rationale:**
Gastric MALT: HP eradication is guideline-based initial therapy; Response may require >12 months. For localized (Lugano I) eradication failure, ISRT is curative and standard (24-30 Gy; 20 Gy study option). Low Ki-67 and lack of lymph node involvement speak against primary systemic therapy.

**===== run_2 =====**

**Tumor board decision:**
Yes. Primary test for H. pylori (histology +/- serology/PCR) and eradicate if detected; In the case of H. pylori negativity, empirical eradication is still recommended. Response control via EGD with biopsies (possible up to >12 months). In the absence of remission or progression or symptomatic findings: ISRT (involved site) 24-30 Gy. Asymptomatic persistence: watch and wait. Systemic therapy only in cases of dissemination or a course requiring treatment.

**Rationale:**
Gastric MALT, Lugano I, low Ki-67 (10-15%) and no further staging evidence: Guideline recommendation is primarily HP eradication (response may be delayed). In cases of eradication failure or symptomatic local infection requiring treatment, ISRT (24-30 Gy) offers the curative local option; systemically only in advanced disease/requiring treatment.

**===== run_3 =====**

**Tumor board decision:**
Primary: Helicobacter pylori test (histology/UBT/serology) and eradication if detected. If HP negativity occurs, still consider eradication. Endoscopic/histological response control up to >=12 months. If there is no remission or progression -> ISRT involved site 24-30 Gy (20 Gy study option). Watch and wait if the disease is stable and asymptomatic; Systemic therapy only if the findings are disseminated/requiring treatment.

**Rationale:**
In accordance with guidelines: treat gastric MALT primarily through HP eradication; Remission can take a long time. Locally persistent/resistant stage I after eradication is preferably treated curatively with ISRT. Low Ki-67 and lack of systemic manifestations speak against primary systemic therapy. DM type II is not contraindicated.

## ================================================================ gpt-5-mini (plain) ================================================================

**===== run_1 =====**

**Tumor board decision:**
Further procedure: 1) Test for Helicobacter pylori (histology, UBT/Ag). If the result is positive: immediate eradication therapy (local guideline-adapted regimen). Endoscopic control with biopsies at 3 and 6 months, further observation until 12 months. 2) If H. pylori is negative or there is no regression/progression up to 6-12 months: Involved-site radiotherapy (~30 Gy).

**Rationale:**
Local MALT lymphoma stage IAE (gastrointestinal, Ki67 10-15%) - Standard: Eradicate H. pylori infection first because of high remission rate. If there is no regression or H. pylori negativity, local radiation therapy (curative intent, approx. 30 Gy) is indicated. No relevant comorbidity

contraindicates this strategy.

**===== run_2 =====**

**Tumor board decision:**
Recommendation: Precise H. pylori diagnosis (histology if necessary supplemented by urea breath test and/or stool antigen, serology if necessary). If proven, immediate eradication therapy (resistance-based; bismuth quadruple or PPI-based therapy). Close endoscopy with biopsies; no regression after 3-6 M: involved-field radiotherapy 24-30 Gy.

**Rationale:**
Localized MALT lymphoma (stage I AE) usually responds to H. pylori eradication; Organ-preserving first-line therapy is standard. Low Ki67 and lack of system involvement support a wait-and-see eradication approach. In cases of persistence/progression, radiation is indicated as an established second line. DM type II is not contraindicated.

**===== run_3 =====**

**Tumor board decision:**
Next procedure: Immediate testing for Helicobacter pylori (UBT/stool) and immediate eradication therapy (if status is unknown: empirical eradication). Endoscopic follow-up checks with biopsies every 3-6 months. For persistent lymphoma after 6-12 months or for primary H. pylori-negative findings: involved field radiotherapy 24-30 Gy. Consider molecular testing (t(11;18)/MALT1).

**Rationale:**
Stage I gastric MALT lymphomas often respond to H. pylori eradication. CT without further manifestations suggests local therapy. RT is an established second line for residual or H. pylori-negative findings. Low Ki-67 and comorbidity (DM type II) not a contraindication.

## ============================================================ gpt-5-nano (agent) ============================================================

**===== run_1 =====**

**Tumor board decision:**
Check HP status. HP+: HP eradication as first line; endoscopy control; if there is no response, ISRT gastric (~24 Gy). HP-negative or t(11;18)+: Primary ISRT; Progression/refractoriness: systemic rituximab-bendamustine.

**Rationale:**
Local gastric MALT IU; HP+ eradication makes sense; t(11;18) associates poor eradication; ISRT provides good local control; Chemo upfront only for progression.

**===== run_2 =====**

**Tumor board decision:**
1) Clarify HP status. 2) If HP positive: HP eradication as initial therapy; Endoscopic reevaluation after 3-6-12 months; in remission observation. 3) If HP negative or eradication fails/tt(11;18) positive: ISRT of the stomach with approx. 30 Gy (local control). 4) Non-response/relapse: Rituximab-bendamustine; If necessary, zanubrutinib depending on comorbidity and approval.

**Rationale:**
Gastric MALT in stage IE with local involvement: HP eradication is standard for HP-positive disease; t(11;18) shows lower response to eradication. Locality enables ISRT with high control. Systemic rituximab-based therapy is useful if there is no response or relapse or if RT is not suitable.

**===== run_3 =====**

**Tumor board decision:**
Clarify HP status. HP positive: HP eradication as initial therapy, then endoscopy histo (3-6 months). Remission: observation; Non-response/progression: ISRT stomach 30 Gy. HP negative or eradication failed: ISRT 30 Gy primary. RT impossible/progression: BR-rituximab system therapy; Relapse: Zanubrutinib (2nd line) or similar regimen.

**Rationale:**
Indolent MALT lymphoma of the stomach (stage IE) with local gastric involvement. HP eradication makes sense, t11;18 influences response. Local control through ISRT efficient; BR system therapy in case of progression; Avoid upfront R-CHOP due to toxicity and slow progression.

## ========================================================== gpt-5-nano (plain) ==========================================================

**===== run_1 =====**

**Tumor board decision:**
1) Clarify H. pylori status (test/biopsy). 2) Positive: H. pylori eradication; Endoscopy follow-up after 3-6 months. 3) Negative or persistent: local radiotherapy of the stomach 24-30 Gy (1.8-2 Gy/Fx, approx. 12-17 Fr.). 4) If refractoriness/progression: consider systemic rituximab/bendamustine therapy.

**Rationale:**
Stage IAE MALT lymphoma of the stomach is localized and radiosensitive. H. pylori eradication strengthens healing; Radiotherapy offers high local control in MALT lymphomas. Systemic therapy only in cases of refractoriness/progression or H. pylori negativity. Close follow-up care required.

**===== run_2 =====**

**Tumor board decision:**
1) Clarify H. pylori status (urea respiration test/stool antigen or biopsy). 2) If infection is positive, eradication therapy; Endoscopic reevaluation 6-8 weeks after therapy. 3) If residual findings or H. pylori-negative: local radiotherapy to the stomach (involved-field RT) approx. 30 Gy. 4) In case of contraindications/non-response, rituximab-based systemic therapy.

**Rationale:**
Stage IE MALT lymphoma of the stomach. H. pylori eradication often results in remission; RT provides high local control for local infestations; Systemic therapy as a reserve in case of intolerance or non-response.

**===== run_3 =====**

**Tumor board decision:**
Check H. pylori status. Positive: eradication therapy; Reassessment. Residual/non-eradication: local gastric RT 30 Gy (15 Fr). Negative: RT 30 Gy. Progression: systemic rituximab-based therapy.

**Rationale:**
Stage IAE MALT gastric lymphoma; H. pylori-driven disease; RT offers high local control; Eradication makes sense; no systemic infection; Ki-67 low.

## ========================================================== gpt-oss-20b (agent) ==========================================================

**===== run_1 =====**

**Tumor board decision:**
HP eradication (standard triple therapy) as an initial measure; then endoscopic control after 6 months. If there is no response or progression: involved-site radiotherapy (20Gy) or endoscopic resection.

**Rationale:**
Flowchart A1 recommends HP eradication as initial therapy for gastric MALT, even with HP-negative findings. Currently no symptoms, no progression. Observation after eradication is standard. First, local therapy if there is no response.

**===== run_2 =====**

**Tumor board decision:**
Recommendation: First therapeutic intervention - Helicobacter pylori eradication (standard 7-14 day triple therapy). If the HP status is negative, still carry out eradication. After 3-6 months endoscopy control; If there is remission, observation, if there is persistence/progression, further local (ISRT) or systemic therapy.

**Rationale:**
Stage I (localized), no nodal involvement, no symptoms, HP eradication is evidence-based first-line therapy for gastric MALT, regardless of HP status. No previous therapies, no contraindications. Flowchart-A1/A2/A3 support this approach.

**===== run_3 =====**

**Tumor board decision:**

Recommendation: Start standard Helicobacter pylori eradication (triple therapy) and plan a follow-up EGD endoscopy 6-12 months later. If persistence or progression occurs, ISRT or systemic rituximab-bendamustine may be given.

**Rationale:**
StageIAE MALT lymphoma in the stomach without prior therapy; Flowchart A1 calls for HP eradication as initial therapy, regardless of HP status. Observation after eradication is common if there is a response. If there is no response, local radiotherapy (ISRT) is recommended. Comorbidities (HTA) no contraindications.

## ============================================================ gpt-oss-20b (plain) ============================================================

**===== run_1 =====**

**Tumor board decision:**
Set targeted radiation therapy (30Gy in 15Fr) for gastinal MALT lymphoma. No previous therapy, no H. pylori disease, moderate Ki67. Radiotherapy is the standard option for localized non-H. pylori-associated MALT lymphoma.

**Rationale:**
Local, stage I AE without nodal involvement; no prior treatment; moderate proliferation rate. Radiotherapy 30Gy has a high remission rate, minimal side effects and is the recommended first-line therapy in the absence of H. pylori disease.

**===== run_2 =====**

**Tumor board decision:**
For stage I AE MALT lymphoma in the stomach, we recommend endoscopic resection (EMR/ESD) or, if inoperable, low-dose radiation therapy (30Gy).

**Rationale:**
No previous course of therapy, no progression, Ki67 10-15% and local extralymphatization allow locally limited therapy. Endoscopic resection offers minimally invasive, curative treatment for small lesions; Radiation therapy is alternative option for inaccessible lesion.

**===== run_3 =====**

**Tumor board decision:**
Proceed with endoscopic mucosal resection (EMR/ESD) of the gastric MALT lesion; if technically unfeasible, offer low-dose radiotherapy (30Gy).

**Rationale:**
Stage I AE, no nodal disease, Ki67 10-15%, favorable biology. Endoscopic resection is curative with minimal morbidity; RT is effective for residual or unresectable lesions. No prior therapy and no contraindications.

## ============================================================ gpt-oss-120b (agent) ============================================================

**===== run_1 =====**

**Tumor board decision:**
First-line therapy: Helicobacter pylori eradication (14-day triple or quadruple therapy) followed by endoscopic control biopsy after 3-6 months; If there is a response, observation, if there is no response, local ISRT.

**Rationale:**
Stage I-AE MALT lymphoma of the stomach is indolent; Guideline recommends eradication as the treatment of choice, regardless of HP status, with a high remission rate. No contraindications (HTN only). Radiotherapy only after no response.

**===== run_2 =====**

**Tumor board decision:**
First-line therapy: Helicobacter pylori eradication (e.g. 14-day quadruple therapy). After 3-6 months endoscopy + histology to check the response. In case of complete remission, observation (watch and wait). In the absence of remission or persistent evidence, involved-site radiotherapy (ISRT, approx. 20Gy).

**Rationale:**
Gastric MALT lymphoma StageIAE responds to HP eradication in >80%, even in HP negativity. Ki-6710-15% and lack of nodal findings suggest an indolent course. Radiotherapy is only used after eradication failure to avoid unnecessary radiation.

**===== run_3 =====**

**Tumor board decision:**
We first recommend Helicobacter pylori eradication (e.g. 14 days of quadruple therapy), followed after 3-6 months by an endoscopic control biopsy. If there is a response or stable, asymptomatic persistence, observation (watch and wait) is carried out. If there is no response or progression, localized involved-site radiotherapy (~20Gy) should be performed.

**Rationale:**
MALT lymphoma is indolent, StageIAE without nodal involvement and Ki-6710-15% has a favorable risk. According to the guidelines, H. pylori eradication is the treatment of choice, even in cases of HP negativity, and leads to remission in >80%. If there is a response, observation is sufficient; If there is no response, ISRT is the established curative local therapy.

## ============================================================ gpt-oss-120b (plain) ============================================================

**===== run_1 =====**

**Tumor board decision:**
First check H. pylori status and if positive, carry out 2 weeks of eradication therapy. If the results are negative or there is no remission after eradication, offer local radiotherapy (30Gy, involved field). In case of complete remission after eradication, observation without immediate therapy.

**Rationale:**
Stage I MALT lymphomas of the stomach are usually dependent on H. pylori; Eradication leads to complete remission in >80%. Radiation therapy is only the standard local therapy in cases of H. pylori negativity or persistence. The patient has no symptoms and no contraindications.

**===== run_2 =====**

**Tumor board decision:**
First, H. pylori detection (urea respiration, stool antigen or histology). If positive, 14-day eradication, followed by endoscopic control (3-6 months). In case of negativity or lack of response, immediate local radiotherapy (30Gy involved field) or alternatively rituximab monotherapy.

**Rationale:**
MALT lymphomas in gastric stage I heal in >80% after H. pylori eradication; therefore first test and therapy. Without infection, radiotherapy is evidence-based and offers high local control with low risk. Rituximab is an option when radiation therapy is contraindicated.

**===== run_3 =====**

**Tumor board decision:**
First carry out an H. pylori test and, if necessary, eradication therapy; If the results are negative or there is no response, local involved field radiotherapy (~30Gy) as standard therapy for stage I MALT lymphoma of the stomach. Check blood pressure.

**Rationale:**
Ex-nodal MALT lymphoma is indolent, stage I, without systemic manifestations. H. pylori eradication cures >80% of cases; In the case of negative results or treatment failure, radiotherapy with high local control and low risk is the treatment of choice.

## ============================================================ qwen3-32b (agent) ============================================================

**===== run_1 =====**

**Tumor board decision:**
Helicobacter pylori eradication is recommended as the first line of therapy, followed by response monitoring after 6-12 months. If there is persistence or progression, ISRT (involved-site radiotherapy) is performed.

**Rationale:**

Gastric MALT lymphoma stage I AE primarily benefits from HP eradication, regardless of Ki67 value. If there is no HP infection or treatment failure, ISRT is the treatment of choice. No systemic therapy is required for localized infection without complications.

**===== run_2 =====**

**Tumor board decision:**
Recommended therapy: Helicobacter pylori (HP) eradication as first line, followed by endoscopic response monitoring. If there is no response or progression: involved field radiotherapy (ISRT).

**Rationale:**
Gastric MALT lymphoma stage I AE benefits primarily from HP eradication (recommended even in negative status). Ki67 <20% and no systemic manifestations suggest local therapy. ISRT as a second step in treatment resistance.

**===== run_3 =====**

**Tumor board decision:**
Recommendation: H. pylori eradication therapy as first line, followed by endoscopic response monitoring. In case of treatment failure or progression: consider ISRT (involved-site radiotherapy).

**Rationale:**
Stage I AE gastric MALT lymphoma with low Ki67 (10-15%) and localized involvement. H. pylori eradication is standard therapy for gastric MALT. If responded: observation. If therapy fails: ISRT as a local therapy option. PET/CT not indicated due to low FDG avidity.

## ============================================================ qwen3-32b (plain) ============================================================

**===== run_1 =====**

**Tumor board decision:**
Recommendation: Carry out a Helicobacter pylori test. If the result is positive, initiate eradication therapy. If the results are negative or there is no response: site-specific radiotherapy (30-36 Gy) in the stomach is the preferred treatment option.

**Rationale:**
Stage I extranodal MALT lymphoma with gastric localization. Eradication of H. pylori (if detectable) is the first choice. In the absence of H. pylori or resistance to therapy, radiation therapy is the standard therapy with a high response. No systemic therapies required for localized stage I.

**===== run_2 =====**

**Tumor board decision:**
Recommend clarify H. pylori status. If H. pylori positive: eradication therapy. For H. pylori negative: local radiotherapy to the stomach (30-36 Gy) as standard therapy for stage I extranodal MALT lymphoma.

**Rationale:**
Stage I extranodal MALT lymphoma of the stomach benefits from local therapies. H. pylori workup is crucial because 50-70% of gastric MALT lymphomas are associated. With eradication therapy, regression occurs in 60-80% of cases. In the case of H. pylori negativity, radiotherapy with a high local control rate is established. No indication for chemotherapy in localized forms without risk factors.

**===== run_3 =====**

**Tumor board decision:**
Recommendation: carry out Helicobacter pylori testing. If proven: initiate eradication therapy. If the result is negative: consider local radiotherapy to the stomach.

**Rationale:**
Stage I extranodal gastric MALT lymphoma with low Ki67 (10-15%). Eradication of H. pylori is the gold standard for infectious MALT. If the test is negative, radiotherapy is the preferred option. No evidence of systemic disease or contraindications.

## =========================================================== qwen3-next-80b (agent) ===========================================================

**===== run_1 =====**

**Tumor board decision:**
Yes, do H. pylori testing. If the result is positive: eradication therapy. If the result is negative: involved-site radiotherapy (ISRT) with 20-30 Gy as standard therapy.

**Rationale:**
According to the flowchart (Chapter A) for gastric MALT lymphoma, H. pylori testing is mandatory. If the result is positive, eradication is the first choice. If the findings are negative and the stage is localized (Lugano I), ISRT is indicated, as eradication is not effective if the findings are HP-negative. DM type II does not influence therapy.

**===== run_2 =====**

**Tumor board decision:**
Perform H. pylori testing. If the result is positive: eradication therapy. If the result is negative: involved-site radiotherapy (ISRT) with 20-30 Gy.

**Rationale:**
According to the Onkopedia guidelines (2023) and clinical practice, H. pylori testing is mandatory for gastric MALT. If HP positive: eradication as first line. For HP negative: ISRT is standard therapy for localized stage I. Observation only if the disease is asymptomatic.

**===== run_3 =====**

**Tumor board decision:**
Be sure to carry out H. pylori testing. If the result is positive: eradication therapy (triple therapy). If the result is negative: involved-site radiotherapy (ISRT) with 20-30 Gy as standard therapy. Observation only if the course is asymptomatic and the tumor burden is very low.

**Rationale:**
Gastric MALT lymphoma requires mandatory H. pylori testing (histology from normal mucous membranes). If HP positive: Eradication is first-line therapy. For HP negative: ISRT is standard (Lugano I). Comorbidity (DM type II) does not affect therapy. Ki67 10-15% speaks for active therapy, not just observation.

## ================================================================ qwen3-next-80b (plain) ================================================================

**===== run_1 =====**

**Tumor board decision:**
Perform H. pylori test. If the result is positive, antibiotic therapy (e.g. triple therapy), if the result is negative, local radiotherapy (24-30 Gy).

**Rationale:**
Gastric MALT lymphoma is often H. pylori-associated. The first treatment if the test is positive is antibiotics. If the test is negative, radiation therapy is standard. DM type II is not a contraindication. Ki67 10-15% does not change standard therapy.

**===== run_2 =====**

**Tumor board decision:**
Perform H. pylori test. If the result is positive: eradication therapy (e.g. triple therapy). If the result is negative: local radiation (24-30 Gy).

**Rationale:**
Gastric MALT lymphoma requires H. pylori testing as the first stage. In case of infection, eradication, in case of negativity, local radiation. No previous therapy, therefore no contraindications. DM type II does not influence therapy.

**===== run_3 =====**

**Tumor board decision:**
Perform H. pylori test. If the result is positive, antibiotic eradication therapy (e.g. PPI + clarithromycin + amoxicillin), if the result is negative, local radiotherapy (24-30 Gy) to the stomach.

**Rationale:**
Gastric MALT lymphoma requires primary H. pylori testing. Eradication of infection leads to remission in >80%. If the result is negative, local radiotherapy is standard for stage I. No contraindications due to type II DM.

# ly_case_4__ALL

################################
#### CASE: 4

################################

## ======================================================== gpt-5-mini (agent) ========================================================

**===== run_1 =====**

**Tumor board decision:**
Continuation of epcoritamab as bridging. Restaging (PET/CT) after 2 additional cycles; in case of metabolic CR or clear PR: plan allo-SCT as consolidation. The 8-cm pulm persists. Lesion metabolically active or only slightly regressive: primary surgical resection for technical feasibility and sufficient lung reserve; otherwise thoracic radiotherapy (ISRT/SBRT, e.g. ~30-36 Gy). Allo-SCT ideally 4-8 weeks after local therapy or as soon as the best possible response has been achieved. If system progression occurs under epcoritamab, consider alternative systemic therapy/study and accelerate rapid transplant planning.

**Rationale:**
After CAR-T failure, BiTE (epcoritamab) is a guideline-consistent option; The aim is to minimize residual disease before allo-SCT. An isolated bulky thoracic lesion impairs transplant prognosis-local consolidation (OP preferred, otherwise RT) improves chance of NED. Time window 4-8 weeks after local therapy allows recovery before conditioning.

**===== run_2 =====**

**Tumor board decision:**
Continue epcoritamab as bridging; Re-staging (PET-CT) after another 2-3 cycles. If metabolic CR/PR (Deauville <=3), plan allo-SCT. If there is a persistent metabolically active bulky lesion (>5-6 cm) or no response: primary surgical resection if feasible and tolerated by the patient, otherwise thoracic ISRT (~30-36 Gy) before rapid allotherapy (4-6 weeks after best response).

**Rationale:**
BiTE (epcoritamab) is suitable systemic therapy after CAR-T failure; The goal is low tumor burden before Allo. Bulky, metabolically active residual tumors increase transplant risk -> local control (OP or ISRT) if systemic response is inadequate. PET-driven decision and rapid timing recommended.

**===== run_3 =====**

**Tumor board decision:**
Continue epcoritamab primarily as systemic bridging. Restaging (PET-CT) after 2-3 cycles. For CR or clear PR: rapid allo-SCT. For persistent PET-positive/bulky pulmonary residual (especially >5 cm or satellite): locoregional consolidation before Allo (preferred ISRT 36-40 Gy; resection only if complete R0 is technically favorable). If there is no response: switch to alternative BiTE/study or palliative local therapy.

**Rationale:**
After CAR-T failure, BiTEs such as epcoritamab are suitable systemic bridging options; The aim is to achieve the best possible systemic remission before allo-SCT. A large, isolated, PET-active focus increases graft failure, therefore local consolidation (RT or surgery) is recommended, allo as soon as best possible response is achieved (PET-neg ideal, otherwise major PR).

## ======================================================== gpt-5-mini (plain) ========================================================

**===== run_1 =====**

**Tumor board decision:**
Continuation of epcoritamab according to study/approval protocol (complete induction series), close monitoring (CRS/Neuro), PET-CT after cycle 3-4. For radiological/clinical progression: pembrolizumab (due to PMBCL prevalence of PD-L1) or polatuzumab-BR / Loncastux as needed; for CR assessment for Allo-SCT if patient-fit and donor available.

**Rationale:**
Only given 2 doses of epcoritamab and currently SD; no evidence of refractory to epcoritamab -> do not switch prematurely. PMBCL often responds to PD-1 inhibition (option after CAR-T failure). Aim:

Disease control with the possibility of consolidation (allo-SCT) or inclusion in studies.

**===== run_2 =====**

**Tumor board decision:**
Continue epcoritamab per protocol for at least 2-3 cycles with PET-CT restaging. For CR/PR and transplant suitability: rapid planning and consolidation using allogeneic stem cell transplantation. In case of progression: switch to PD-1 inhibitor (e.g. pembrolizumab) or study inclusion; If necessary, palliative/ablative RT of the 8-cm focus.

**Rationale:**
Epcoritamab was only administered for a short time - therapy was not adequately assessed. After CAR-T failure, Allo-SCT is a rational consolidation if remission is achievable. PMBCL shows response to PD-1 blockade, RT useful for local control of bulky lesions.

**===== run_3 =====**

**Tumor board decision:**
Continuation of epcoritamab until completion of the recommended induction (at least 12 weeks/full regimen) with close clinical monitoring and PET-CT after induction. In case of progression during/after adequate exposure, switch to polatuzumab vedotin + bendamustine + rituximab (if clinically appropriate) or study participation. If there is a response, consider local consolidation (ISRT/resection) of the 8 cm lesions; If remission is stable, consider Allo-SCT as consolidation.

**Rationale:**
Epcoritamab works differently than previously failed CAR-T and may have a delayed response; therefore wait for complete induction. If failure occurs, polatuzumab-based salvage regimens or trials are the realistic options. Local RT/resection is used for tumor control, allo-SCT only if there is a systemic response.

## ============================================================ gpt-5-nano (agent) ============================================================

**===== run_1 =====**

**Tumor board decision:**
Continuation of epcoritamab and simultaneous focal radiotherapy of the dominant lung tumor lesion (~8 cm) as a bridge. Aim: local control and potential remission; check in parallel for allogeneic stem cell transplantation (AlloSCT) or clinical studies if there is sufficient remission; PD-1 inhibition or additional BIS-T engagers as backup in case of progression.

**Rationale:**
Similar cases use BIS T cell engagers + local RT as a bridge to AlloSCT. AlloSCT offers curative potential with sufficient remission; PMBCL data limited post-CAR-T. RT addresses bulky lesion; continued BIS-T therapy enables systemic control; Consider trials/AlloSCT as potentially curative options.

**===== run_2 =====**

**Tumor board decision:**
Continuation of epcoritamab + focal radiotherapy of the dominant lesion on the right upper lobe (~8 cm) with ISRT/30-40 Gy; Lymphoma conference coordinated. If progression occurs, consider PD-1 inhibition or clinical trial; Allogeneic stem cell transplantation only if there is sufficient remission/suitable fitness and study availability.

**Rationale:**
The dominant 8 cm lesion shows SD on epcoritamab; local RT enables conversion/control. Systemic options remain (PD-1 inhibition, studies). AlloSCT only as a later option if there is sufficient remission/good fitness.

**===== run_3 =====**

**Tumor board decision:**
Continuation of epcoritamab + focal radiotherapy of the dominant lung tumor lesion (approx. 8 cm in the right upper lobe) with 36-40 Gy; Goal: local control, maintenance of systemic response. If progression occurs, consider PD-1 inhibition or clinical trial; Allogeneic stem cell transplantation only if remission is achievable/study context.

**Rationale:**

SD on epcoritamab; Local RT useful to control bulk and maximize overall success while continuing the immunotherapy principle. Systemic second line only after progression; PD-1/Check study options; alloSCT only in remission/within a study.

## ======================================================== gpt-5-nano (plain) ========================================================

**===== run_1 =====**

**Tumor board decision:**
Continuation of epcoritamab until progression/intolerance. If the bulky lesion persists: adequate local RT (SBRT 30-40 Gy) to the dominant thoracic focus. Progression: Pembrolizumab-based or clinical trial; Allogeneic transplantation only with sufficient remission and good suitability.

**Rationale:**
Epcoritamab useful after CAR-T relapse PMBCL; Bulky stoves require local RT; Transplantation only after remission, if suitable.

**===== run_2 =====**

**Tumor board decision:**
Continuation of epcoritamab (Z3D8) if currently SD; additional local SBRT of the dominant lesion in the right upper lobe (approx. 30 Gy/5 Fr) for local control; in case of progression/intolerance: consideration of allo-SCT or participation in a clinical trial.

**Rationale:**
Current stability on epcoritamab; local radiotherapy allows targeted control of the dominant lesion; continue systemic therapy; In case of progression or unacceptable toxicity, consider allo-SCT or study option.

**===== run_3 =====**

**Tumor board decision:**
continuation of epcoritamab until progression/intolerance; local radiotherapy of the dominant 8 cm lesion for consolidation; parallel testing for allogeneic SCT (finding a donor) with possible transplantation if there is an adequate response; If necessary, participation in clinical studies (PD-1 inhibitors or other bispecifics) as a reserve.

**Rationale:**
SD under epcoritamab with bulky residual; local RT can cause further reduction. Allogeneic SCT offers a potentially more curative option in a suitable donor; Transplant makes sense after deeper response or stabilization; clinical trials as an alternative if SCT is not available.

## ======================================================== gpt-oss-20b (agent) ========================================================

**===== run_1 =====**

**Tumor board decision:**
After stable SD with epcoritamab, we initially recommend the combination nivolumab+brentuximab-vedotin. If the answer is positive (CR/PR), we plan an alloSCT. Alternatively, clinical trial access to glofitamab or another bispecific antibody therapy can be considered. ASCT without remission is rejected.

**Rationale:**
The only comparable case study showed nivolumab+BV as a useful option followed by alloSCT after bispecific therapy. Current literature supports bispecific antibodies and CAR-T after relapse, but ASCT is not recommended without remission. Therefore, nivolumab+BV is the logical next step, followed by alloSCT in remission.

**===== run_2 =====**

**Tumor board decision:**
After stable epcoritamab, we first recommend high-dose chemotherapy with ASCT (if there are no contraindications). Alternatively, checkpoint inhibitor + brentuximab vedotin (VISITING CHECKMATE-436) followed by alloSCT. Glofitamab+GemOx, ViPOR or repeat CAR-T are backup options.

**Rationale:**
Epcoritamab SD does not show remission. HD-Chemo+ASCT has good OS in PMBCL after CAR-T. Check-point+Brentuximab + alloSCT achieved remissions in similar cases. Glofitamab+GemOx and ViPOR

are phase 2/3 alternatives; renewed CAR-T possible, but with higher risk.

**===== run_3 =====**

**Tumor board decision:**
After stable epcoritamab status, high-dose chemotherapy (e.g. BEAM) followed by autologous ASCT is recommended if the patient has no transplant contraindications. If ASCT is not possible, a bispecific antibody (e.g. glofitamab or mosunetuzumab) should be considered or participation in a clinical trial (CAR-T or ViPOR trial) should be sought.

**Rationale:**
Literature (5 PubMed articles) shows that high-dose ASCT increases the remission rate after epcoritamab SD; bispecific antibodies (glofitamab) have been documented as effective second-line options. The only similar case supports a combined immune transplant strategy (nivolumab-brentuximab-alloSCT).

## ============================================================ gpt-oss-20b (plain) ============================================================

**===== run_1 =====**

**Tumor board decision:**
Start nivolumab 240mg IV q2weeks (or pembrolizumab 200mg q3weeks) as monotherapy checkpoint inhibitor therapy. Evaluate after 4-6 cycles (PET-CT) and plan ASCT bridge if the answer is positive. If remission does not occur, consider alternative clinical trials.

**Rationale:**
Patient has SD after R-DA-EPOCH, ISRT, surgery, CAR-T and epcoritamab. Nivolumab/pembrolizumab show response rates of 30-40% in R/R-PMBCL after CAR-T with a good safety profile. Monotherapy is well tolerated, enables rapid evaluation and, if necessary, ASCT bridge.

**===== run_2 =====**

**Tumor board decision:**
After stable disease progression under epcoritamab, salvage therapy with brentuximab vedotin + bendamustine is recommended, followed by evaluation for autologous stem cell transplantation (ASCT) if the response is good.

**Rationale:**
PMBCL is CD30 positive, BV+Ben is proven in relapsed/relapsed B-cell lymphoma. Epcoritamab showed SD; A further up to 2-3 cycles of BV+Ben may enable ASCT, which offers long-term remission. Alternatively, tafasitamab+lenalidomide is possible, but less data in PMBCL.

**===== run_3 =====**

**Tumor board decision:**
Salvage chemotherapy (e.g. ICE regimen) to achieve remission, followed by evaluation for allogeneic stem cell transplantation (or, if not possible, one-day bispecific therapy such as mosunetuzumab).

**Rationale:**
Patient is PD/SD after CAR-T and epcoritamab. ICE has a remission rate of ~30% in PMBCL after CAR-T and enables subsequent allo-SCT remission. If there is no remission, another bispecific may be considered as an alternative.

## ============================================================ gpt-oss-120b (agent) ============================================================

**===== run_1 =====**

**Tumor board decision:**
Intensify current therapy: Combine epcoritamab with short-term chemotherapy (e.g. GemOx or ViPOR) or add a PD-1 blocker to achieve a deeper remission, and then plan an allogeneic stem cell transplant as curative consolidation if the patient is transplant-eligible.

**Rationale:**
After CAR-T failure, epcoritamab only showed stable disease (8 cm lesion). Literature and similar cases demonstrate high CR rates of bispecific chemocombinations and the curative role of allo-SCT after response. Intensification creates better disease control before transplantation.

**===== run_2 =====**

**Tumor board decision:**
Switch to CD19-targeted salvage therapy with polatuzumab-bendamustine-rituximab (R-Pola-Benda) as a bridge, followed by allogeneic stem cell transplantation, provided the patient is fit and a suitable donor is available; Before therapy, another biopsy was carried out to confirm CD19/CD20 expression.

**Rationale:**
After failure of DA-EPOCH-R, local radiation, CAR-T (axi-cel) and BiTE (epcoritamab) with only stable findings, there is a large residual (8cm). Guidelines recommend anti-CD19 therapy after CAR-T failure; R-Pola-Benda achieves high response rates and serves as a bridge therapy to alloSCT, which is the only curative option for refractory PMBCL. The patient shows no obvious contraindications.

**===== run_3 =====**

**Tumor board decision:**
Recommendation: If the disease is stable under epcoritamab and a persistent 8cm tumor, a curative approach with allogeneic stem cell transplantation (allo-SCT) should be sought. As bridging therapy before transplantation, a PD-1 inhibitor (e.g. nivolumab) in combination with brentuximab vedotin (if CD30 positive) or a switch to glofitamab is recommended to achieve a deeper remission.

**Rationale:**
After failure of R-DA-EPOCH, radiotherapy, CAR-T and only stable disease with epcoritamab, allo-SCT is the only potentially curative option. Literature demonstrates efficacy of PD-1 blockade+BV after CAR-T failure, and glofitamab shows high response rate. A deeper remission before allo-SCT improves survival.

## ============================================================ gpt-oss-120b (plain) ============================================================

**===== run_1 =====**

**Tumor board decision:**
Continue epcoritamab therapy (at least until cycle 6) and simultaneously evaluate the response rate using PET-CT. If PR/CR is proven, an allogeneic stem cell transplant should be considered as a potentially curative procedure. If there is no response, participation in a clinical study protocol (e.g. PD-1 blockade or combined bispecifics) should be considered.

**Rationale:**
The patient has a large residual after R-DA-EPOCH, ISRT, CAR-T and to date only stable disease progression under epcoritamab. Epcoritamab is the only active principle that has not yet been exhausted and may provide further time for a possible allogeneic transplant, which is the only curative option in refractory PMBCL. An early change in therapy would not make sense before there is a clear response.

**===== run_2 =====**

**Tumor board decision:**
We recommend salvage chemotherapy (e.g. ICE or DHAP) to achieve a remission-like situation, followed by allogeneic stem cell transplantation as a potentially curative option. If the patient is not a transplant candidate, a PD-1 inhibitor (pembrolizumab) can be used as individual therapy or as part of a study.

**Rationale:**
The patient has already received R-DA-EPOCH, radiotherapy, CD19-CAR-T and epcoritamab; The current PET/CT shows a persistent, large tumor area (~8cm) despite stable disease. Further targeted therapy has been exhausted, therefore an intensive salvage regimen for bridging therapy up to allo-HSCT is indicated. PD-1 blockade offers a useful alternative if transplantation is not possible.

**===== run_3 =====**

**Tumor board decision:**
Continue therapy with epcoritamab until at least cycle 12 or until proven objective remission; If there is no response or renewed progression, immediate evaluation for allogeneic stem cell transplantation or clinical study.

**Rationale:**
The patient has already received chemotherapy, radiotherapy and CAR-T and has stable disease (SD) on epcoritamab. Another response is still possible; an allo-SCT only makes sense after sufficient remission. Continuing bispecific therapy offers the best chance while reserving alternative options.

## ========================================================= qwen3-32b (agent) =========================================================

**===== run_1 =====**

**Tumor board decision:**
Recommendation: PD-1 inhibitor (nivolumab) + CD30 targeting (brentuximab) as a bridge to allo-SCT. Alternative: ViPOR combination therapy for epcoritamab resistance.

**Rationale:**
The patient showed PD after CAR-T and SD after epcoritamab. PD-1/CD30 combinations show bridging effects to allo-SCT in similar cases. ViPOR is evidence-based for resistance after epcoritamab. Allo-SCT remains a short-term goal.

**===== run_2 =====**

**Tumor board decision:**
Recommendation: ViPOR combination therapy (venetoclax, ibrutinib, pomalidomide, rituximab) as a bridge to allo-SCT. For PD-L1 expression, alternatively nivolumab + brentuximab.

**Rationale:**
The patient demonstrated PD on CAR-T and epcoritamab. ViPOR is evidence-based effective in recurrent B-cell lymphomas after CAR-T. PD-1 inhibitors may target PD-L1-expressing PMBCL types. Allo-SCT remains a long-term goal.

**===== run_3 =====**

**Tumor board decision:**
Recommendation: ViPOR combination therapy (venetoclax + ibrutinib + obinutuzumab) as the next step, possibly as a bridge to allo-SCT if donors are available.

**Rationale:**
After PD under CAR-T and SD with epcoritamab, ViPOR (evidence-based) is an effective triple therapy with BTK, BCL-2 and CD20 targeting. Alternatively, Glofitamab-GemOx could be considered. Allo-SCT remains a long-term goal if a donor is found.

## ========================================================= qwen3-32b (plain) =========================================================

**===== run_1 =====**

**Tumor board decision:**
Continuation of epcoritamab with close CT and PET-CT monitoring. If stabilization (SD) persists and tolerance is good (CRS/ICANS G1-2), therapy is continued. In case of progression or intolerance, clinical studies (e.g. CD19/CD3-BiSECT, tafasitamab) or allogeneic transplantation should be considered.

**Rationale:**
Epcoritamab (CD20/CD3-BiSECT) showed SD with good tolerance after CAR-T progression. PMBCL is CAR-T resistant, so further targeting (CD20) makes sense. LDH normalization and absence of pleural effusion indicate a current response to therapy. If SD persists, therapy is continued, otherwise alternatives are examined.

**===== run_2 =====**

**Tumor board decision:**
Continuation of epcoritamab with further observation after 2 additional cycles

**Rationale:**
The current SD after epcoritamab cycle 3 in the absence of contraindications and stable LDH speaks for continued therapy. For PMBCL relapse after CAR-T and R-DA-EPOCH, epcoritamab (CD20xCD3-BiSE) remains first-line, 3rd-line. Freedom from pleural effusion and lung progression as well as the lack of new LNs support control stability.

**===== run_3 =====**

**Tumor board decision:**
Continuation of epcoritamab with parallel donor search for allo-SCT. If SD/PR continues, plan allo-SCT after 2-3 cycles. In case of progression: experimental therapy (e.g. zanubrutinib) or participation in a clinical study.

**Rationale:**
Epcoritamab showed SD after CAR-T resistance. Allo-SCT remains the only curative option. Requirements: donor, disease control with epcoritamab. If there is no response: check alternatives in the 4th line therapy spectrum.

## ========================================================= qwen3-next-80b (agent) =========================================================

**===== run_1 =====**

**Tumor board decision:**
Continuation of epcoritamab therapy

**Rationale:**
SD under epcoritamab is not a progression, therefore continuing therapy makes sense. Local radiotherapy is not indicated for disseminated disease. Allogeneic transplantation only after progression on epcoritamab, according to literature (Allo-SCT after CAR-T failure and bispecific progression). Currently no CR, but stable disease.

**===== run_2 =====**

**Tumor board decision:**
Continue epcoritamab therapy with close response monitoring; no local radiotherapy or allogeneic stem cell transplantation (alloSCT) is currently indicated.

**Rationale:**
Epcoritamab (BiTE) is the first choice for progression after CAR-T. SD with epcoritamab shows efficacy; further cycles required to optimize response. Local RT for systemic disease (satellite) does not conform to standards. alloSCT only indicated after CR/PR with salvage therapy. Response improvement for transplantation is currently lacking.

**===== run_3 =====**

**Tumor board decision:**
Continuation of epcoritamab with simultaneous local radiotherapy (RT) to the dominant right lower lobe focus (8 cm) and satellites. Only consider allogeneic stem cell transplantation (alloSCT) after achieving complete remission (CR).

**Rationale:**
Epcoritamab shows SD, indicating therapeutic activity. The localized tumor burden (no new LNs/contralateral foci) is RT responsive. RT reduces bulky tumor burden, improves the chances of CR, and then enables alloSCT as a potentially curative option. AlloSCT without CR is not indicated and increases risk without clear benefit.

## ========================================================= qwen3-next-80b (plain) =========================================================

**===== run_1 =====**

**Tumor board decision:**
Continuation of epcoritamab with regular CT monitoring. In case of progression, alternative therapies should be examined (e.g. glofitamab, clinical studies).

**Rationale:**
Currently SD on epcoritamab, no progression. Epcoritamab is the standard therapeutic option after CAR-T failure. Continue until progression or maximum response. Alternatives for progression.

**===== run_2 =====**

**Tumor board decision:**
Continuation of epcoritamab therapy according to standard dose planning with regular imaging to assess progression.

**Rationale:**
Stable disease (SD) on epcoritamab after 2 doses, no progression. Continuation makes sense to achieve CR. Consider allogeneic transplantation in CR. No contraindications, current tolerance good.

**===== run_3 =====**

**Tumor board decision:**

Continue epcoritamab therapy with repeat CT imaging in 4-6 weeks. If there is no conversion to PR/CR: consider candidacy for clinical trial or allogeneic stem cell transplantation.

**Rationale:**
SD with epcoritamab shows no progression but also no complete remission. Epcoritamab is the current standard therapy after CAR-T failure. Continuation makes sense to achieve remission. If there is no improvement: alternatives such as studies or allo-SCT if you are physically fit.

# ly_case_5__ALL

################################
#### CASE: 5
################################

## ============================================================ gpt-5-mini (agent) ============================================================

**===== run_1 =====**

**Tumor board decision:**
Immediate histological confirmation (ENT biopsy including FACS). If early recurrence is confirmed (<=12 months) and CAR-T suitability: primary CAR-T therapy with bridging if necessary. If the biopsy is negative/unsuitable for CAR-T: conventional salvage (containing platinum) and if there is a response, check auto-SCT.

**Rationale:**
SOP/Flowchart: Re-biopsy is mandatory before deciding on therapy. Early recurrence <=12 months -> CAR-T preferred if patient is suitable. AHT/DM2 are not general contraindications; Clarify fitness/heart (EKG/Echo/TropT). Bridging up to CAR-T; alternatively Salvage+ASCT only if CAR-T suitability is not available.

**===== run_2 =====**

**Tumor board decision:**
First, histological confirmation (planned ENT biopsy) and restaging. If early EOT recurrence (<=12 months) and CAR-T suitability are confirmed: primary 2nd line CAR-T cell therapy with bridging if necessary. Only consider conventional salvage +/- ASCT if unsuitable for CAR-T.

**Rationale:**
Flowchart/SOP: in P/R after first line always re-biopsy. Early relapse <=12 months -> CAR-T preferred if patient is CAR-T eligible. Previous Pola-R-CHP and high SUVmax speak against primary salvage+ASCT as standard. Check organ functions/comorbidities; Bridging after conference.

**===== run_3 =====**

**Tumor board decision:**
Immediate histological confirmation through the planned ENT biopsy. In case of histologically confirmed progression: recommend primary CAR T cell therapy (if CAR T suitability is determined after cardiac/organic clarification and serology). Bridging therapy individually up to leukapheresis. Conventional salvage->ASCT not primary.

**Rationale:**
EOT-PET DS5 (SUVmax ~17) corresponds to early metabolic relapse <=12 months after R-Pola-CHP. SOP/Flowchart: Re-biopsy mandatory; in early P/R, CAR-T is preferable to salvage+ASCT in CAR-T-eligible patients. AHT and DM2 are not fundamental contraindications.

## ============================================================ gpt-5-mini (plain) ============================================================

**===== run_1 =====**

**Tumor board decision:**
Yes. After histological confirmation of the early recurrence, prompt presentation to a CAR-T center and rapid suitability test (organ screening, ECOG). If suitable, initiate leukapheresis and CAR-T manufacturing immediately. Short-term bridging (e.g. steroid) only if symptoms are urgent, no routine salvage chemo/ASCT.

**Rationale:**
Early recurrence (EOT DS5, FDG-avid tonsil) <12 months after first line = high probability of failure of conventional salvage+ASCT. Randomized studies show advantage of 2nd line CAR-T in early failure. Biopsy confirmation and organ-specific suitability test required; AHT/DM are not grounds for exclusion.

**===== run_2 =====**

**Tumor board decision:**

Yes - indication for second-line CAR-T. Procedure: urgent ENT biopsy for histological confirmation and CD19 detection; If a recurrence is confirmed, immediate presentation to a certified CAR-T center, suitability test and rapid planning of leukapheresis/product ordering. Until therapy: symptom-oriented bridging (cortisone +/- locoregional RT), systemic bridging therapy without further CD19-targeting substances.

**Rationale:**
Early progression directly after R-Pola-CHP (EOT DS5, rapid early recurrence <12 months) corresponds to the criteria for a second-line CAR-T (ZUMA-7/TRANSFORM). No absolute contraindication in the secondary diagnoses; Biopsy, organ screening and CD19 status are required.

**===== run_3 =====**

**Tumor board decision:**
Carry out a biopsy to confirm the tonsil findings histologically. If early recurrence is confirmed: immediate referral to a CAR-T center and planning of leukapheresis/manufacturing; CAR-T therapy recommended as 2nd line (before autologous HSCT). Until availability, product-appropriate bridging (local RT or short-cycle/systemic therapy/steroids).

**Rationale:**
Early recurrence at EOT (Deauville 5, SUVmax~17) after R-Pola-CHP within 12 months; ABC subtype and extranodal involvement = high risk. Phase III data (ZUMA-7/TRANSFORM) show advantage of CAR-T over auto-HSCT as 2nd line. Comorbidities (AHT, DM2) no contraindication. Biopsy confirmation required.

## ============================================================ gpt-5-nano (agent) ============================================================

**===== run_1 =====**

**Tumor board decision:**
CAR-T second line recommended for early recurrence after R-Pola-CHP. Prefers Axicabtagene ciloleucel (Axi-cel) (ZUMA-7). Criteria: ECOG 0-1, normal organ function (CrCl >=60 mL/min, LFTs normal), no CNS involvement, no active infection, adequate cardiac reserve. Bridging as needed; biopsy for confirmation; Lymphoma conference decides. Alternatives: Tisa-cel/Liso-cel depending on availability.

**Rationale:**
Early recurrence after first-line, fit patients corresponds to CAR-T-2L approach. Axi-cel showed better results in ZUMA-7; clear accessibility and logging. requirements met; no CNS involvement; Bridgingpez. require lymphoma conference/diagnosis before infusion. If Axi-cel is not available, consider alternatives.

**===== run_2 =====**

**Tumor board decision:**
CAR-T second line recommended: Axicabtagene ciloleucel (Axi-cel) in DLBCL with recurrence <=12 months after first line, provided CAR-T suitability (ECOG 0-2, organ functions ok, no CNS involvement). Bridging possible; Lymphoma conference decides timing.

**Rationale:**
Early recurrence after R-Pola-CHP with PET-PD; ZUMA-7-like evidence supports second-line CAR-T; potentially curative perspective with appropriate patient selection; conventional therapies offer less long-term cure.

**===== run_3 =====**

**Tumor board decision:**
CAR-T second line is indicated for early recurrent DLBCL after R-Pola-CHP, provided ECOG 0-1, adequate organ functions (KreaClCr >60 mL/min; normal LFTs), no active CNS disease/infection; tumor burden moderate; Bridge possible (RT of the tonsil or short chemo bridging); preferably axicabtagene ciloleucel; Tisa/Liso-Cel as an alternative depending on the center experience.

**Rationale:**
ZUMA-7 showed benefit of axi-cel in second-line CAR-T setting. In the case of early recurrence and extranodal tonsil involvement, there is transplantability/remission potential, taking into account CRS/ICANS risks and bridging options; Clarify CNS status before CAR-T, wait for biopsy to be completed.

## ============================================================ gpt-5-nano (plain) ============================================================

===== run_1 =====

**Tumor board decision:**
CAR T-cell therapy (axicabtagene ciloleucel) as second-line treatment for early relapse DLBCL after frontline pola-R-CHP; biopsy to confirm recurrence; fast logistics including apheresis and transfer to the CAR-T center; if necessary, bridge therapy.

**Rationale:**
Early recurrence within 12 months of effective front line; current evidence (ZUMA-7) supports CAR-T as a second-line option in fit patients; potentially better remissions than salvage chemo; rapid logistics required.

===== run_2 =====

**Tumor board decision:**
Indication for CAR T-cell therapy in early relapse DLBCL after first-line therapy (R-CHP/Pola x6). Second-line therapy within approx. 12 months. Procedure: confirmation of recurrence, leukapheresis, if necessary bridging, lymphodepletion, CAR-T infusion (e.g. axi-cel or lisocabtagene); close monitoring.

**Rationale:**
Early recurrence within one year with PD after initial therapy, extranodal mass; no ASCT planned. CAR-T improves outcome in the 2nd line vs. conventional therapies; Patient is potentially CAR-T eligible despite AHT/DM-2. Fast action makes sense.

===== run_3 =====

**Tumor board decision:**
CAR T-cell therapy is currently not first-line therapy for early recurrence. Strategy: Salvage chemoimmunotherapy (e.g. R-ICE/R-DHAP) with remission and, if necessary, autologous SCT. CAR-T only third-line for refractory course or ASCT intolerance. Biopsy of tonsil to confirm recurrence.

**Rationale:**
In case of early relapse, goal: remission through salvage + SCT; CAR-T is usually third-line therapy or not standard. Biopsy necessary to confirm recurrence and determine therapy.

## ============================================================ gpt-oss-20b (agent) ============================================================

===== run_1 =====

**Tumor board decision:**
CAR T cell therapy (e.g. axicabtagene ciloleucel) with bridging chemotherapy (e.g. 2-3 cycles of R-CHOP or Pola-R-CHP) is indicated. After EOT-PET/CT-PD within 12 months, patient fit, no contraindications.

**Rationale:**
Early metabolic relapse after 6 R-Pola-CHP cycles, B symptoms, extranodal involvement -> CAR-T therapy according to P/R-1/2/3 of the flowchart. Patient without age/cardiovascular limitations, therefore CAR-T eligible. Bridging therapy is recommended to reduce tumor burden until CAR-T infusion.

===== run_2 =====

**Tumor board decision:**
CAR T cell therapy (e.g. axicabtagene ciloleucel) with bridging therapy after EOT PET/CT, due to early metabolic recurrence <=12 months after 6 R Pola CHP cycles, fit, no contraindications.

**Rationale:**
Early, metabolically active recurrence, no further effective chemotherapy, CAR-T capability without age/cardio contraindications, flowchart P/R-1/2/3 recommend CAR-T for R/P <=12 months.

===== run_3 =====

**Tumor board decision:**
CAR T-cell therapy (e.g. axicabtageneciloleucel) after bridging chemo (e.g. 2-3 cycles of R-CHOP or R-pola-bendamustine) is indicated. Patient is fit, no contraindications, early recurrence (<12 months).

**Rationale:**

Flowchart V: P/R-1-3 calls for CAR-T in early relapse if CAR-T-capable. Comorbidities (HTN, DMII) are controllable, no cardiac limitation. Bridging therapy keeps tumor under control until CAR-T infusion.

## ======================================================== gpt-oss-20b (plain) ========================================================

**===== run_1 =====**

**Tumor board decision:**
CAR T cell therapy not indicated. Instead: salvage chemotherapy (e.g. R-ICE or R-DHAP) followed by autologous stem cell transplantation if the response is positive. If no remission, consider participation in clinical trial.

**Rationale:**
Early recurrence after 1st line (R-Pola-CHP) is not yet within the indication range of CAR-T (at least 2nd line or refractory). Salvage chemo + ASCT is standard in DLBCL-R/R. HTN/DM can be controlled and is not a contraindication.

**===== run_2 =====**

**Tumor board decision:**
Consider salvage chemotherapy (e.g. R-ICE) followed by high-dose therapy and autologous stem cell transplantation. CAR T cell therapy only after lack of ASCT compatibility or after second/multiple relapse.

**Rationale:**
Early recurrence after 6 R-Polac-CHP cycles occurs in the first therapy cycle. For chemosensitive patients, ASCT is the standard option; CAR-T is intended for patients with >=2 treatment periods or ASCT intolerance.

**===== run_3 =====**

**Tumor board decision:**
CAR T cell therapy (e.g. tisagenlecleucel) is indicated. After early relapse (<=12 months) after a single R-Pola-CHP line, CAR-T is the standard option. Bridging therapy (e.g. R-CHOP-like) may be considered before infusion.

**Rationale:**
Patient has diffuse large B-cell lymphoma, activated B-cell type, early recurrence after 6 cycles of R-Pola-CHP, no contraindications due to AHT/DM. According to the NCCN and ASCO guidelines, CAR-T is recommended for early recurrence after one line. Bridging therapy can be used if tumor reduction is necessary.

## ======================================================== gpt-oss-120b (agent) ========================================================

**===== run_1 =====**

**Tumor board decision:**
Recommendation: Implement CD19 CAR T cell therapy (axi-cel or tisa-cel) after confirmation of early recurrence (biopsy, CD19 status) and, if necessary, bridging therapy.

**Rationale:**
Early recurrence <12 months after R-Pola-CHP, activated B-cell DLBCL, no contraindications; According to flowchart V-P/R-1/2/3, CAR-T is the preferred option for early progression.

**===== run_2 =====**

**Tumor board decision:**
After confirmed early recurrence (DS5) <=12 months after R-Pola-CHP, CAR T-cell therapy (axi-cel or tisa-cel) is the preferred 2nd line. Before CAR-T, another biopsy, CD19 status check and suitability screening are carried out; If necessary, bridging chemo until product availability.

**Rationale:**
Early recurrence in activated B-cell DLBCL, no absolute contraindications (AHT, DM controllable). According to flowchart V-P/R-3, CAR-T is recommended for <=12 months after CHOP-based regimen, as other options show less effectiveness.

**===== run_3 =====**

**Tumor board decision:**
Recommendation: Immediate CAR T-cell therapy (axi-cel or tisa-cel) as second-line salvage after confirmation of CD19 expression; If necessary, bridging therapy up to apheresis.

**Rationale:**
Early recurrence (<12 months) after R-Pola-CHP corresponds to Flowchart-P/R-3 (early recurrence). The patient is fit and has no contraindications; CAR-T is the preferred option for early progression. Alternative therapies only if CAR-T is not suitable.

## ========================================================== gpt-oss-120b (plain) ==========================================================

**===== run_1 =====**

**Tumor board decision:**
Carry out a biopsy of the findings to histologically confirm the early recurrence. If confirmed, initiate a salvage chemotherapy regimen (e.g. R-ICE or R-DHAP) to achieve PET negativity and then pursue autologous stem cell transplantation (ASCT). CAR T cell therapy is only indicated after a failed ASCT candidacy or after a lack of chemosensitive response.

**Rationale:**
DLBCL showed early metabolic progression (DS5) after a single R-polatuzumab-CHP therapy. According to the guidelines, in the case of early recurrence after the first line regimen, a salvage chemo approach with possible ASCT is standard; CAR-T is reserved for patients who are transplant ineligible or after ASCT failure.

**===== run_2 =====**

**Tumor board decision:**
Indication for CAR T-cell therapy (axi-cel/tisa-cel/liso-cel) as second line for early relapse (DS5, <3 months after EOT) of the activated B-cell type of DLBCL. Before biopsy confirmation, bridging therapy if necessary, then leukapheresis and transfer to a certified CAR-T center.

**Rationale:**
Patient has only one previous chemo-immunotherapy (R-polatuzumab-CHP) and shows early metabolic progression (DS5). According to current guidelines, CAR-T is approved for refractory/relapsed DLBCL after >=1 prior therapy. Comorbidities (AHT, type II DM) do not represent a contraindication.

**===== run_3 =====**

**Tumor board decision:**
Perform biopsy of FDG-avid tonsil resection; If histological confirmation of an early DLBCL recurrence (ABC) is found, immediately initiate CAR T cell therapy (axi-cel/tisa-cel/liso-cel), if necessary with bridging therapy, since early metabolic PD after R-Pola-CHP and lack of transplant suitability.

**Rationale:**
First-line therapy was refractory (DS5), extranodal involvement and B symptoms increase the risk; Current studies (ZUMA-7, BELINDA) show superiority of CAR-T over salvage chemo+ASCT in the 2nd line setting, and the patient has no absolute contraindications.

## ========================================================== qwen3-32b (agent) ==========================================================

**===== run_1 =====**

**Tumor board decision:**
CAR T-cell therapy is indicated as a bridge to possible bone marrow transplantation. Salvation chemotherapy only if CAR-T is contraindicated.

**Rationale:**
Early recurrence after R-Pola-CHP with metabolically aggressive SUVmax ~17 and extranodal sinus involvement. CAR-T is primarily indicated in early PD (<12 months) after first line (flowchart V.P/R-3). Salvage chemotherapy remains in reserve for CAR-T-unable cases. Diabetes/AHT requires preconditional risk assessment.

**===== run_2 =====**

**Tumor board decision:**

CAR T-cell therapy is indicated for early recurrence after R-Pola-CHP with SUVmax ~17 and extranodal sinus involvement. Salvation chemo and autoSCT remain as options for CAR-T-unable patients.

**Rationale:**
The patient meets the criteria for early relapse (<12 months after first-line) with metabolically aggressive PD. CAR-T is superior to salvage chemo in this indication, especially with high expressed FDG. Comorbidities (DM/AHT) require preconditional cardiac reserve assessment.

**===== run_3 =====**

**Tumor board decision:**
CAR T cell therapy is indicated. Salvation chemo and bone marrow transplantation are alternatives, but CAR-T is the first option for early recurrence (<=12 months) and FDG-avidem infestation.

**Rationale:**
The patient has an early recurrence after R-Pola-CHP with metabolic PD (SUVmax ~17). CAR-T is indicated for FDG-avidem relapse within 12 months of first line, regardless of age. Salvating chemo (R-DHAP) and autoSCT remain alternatives, but are less effective in FDG-avider or recurrent disease after CAR-T.

## ============================================================ qwen3-32b (plain) ============================================================

**===== run_1 =====**

**Tumor board decision:**
No, CAR T-cell therapy is not indicated. Salvage chemotherapy with ASCT connection is preferred.

**Rationale:**
Early recurrence after R-Pola-CHP initially requires salvage chemotherapy and ASCT. CAR-T is only indicated for ASCT intolerance/inaptity. Comorbidities (AHT, DM) not contraindicated for ASCT.

**===== run_2 =====**

**Tumor board decision:**
Yes, CAR T-cell therapy is indicated after confirming the diagnosis of recurrence and assessing the patient's fitness.

**Rationale:**
Early recurrence after R-Pola-CHP with extranodal involvement and high SUVmax. CAR-T is indicated for first-line, primary refractory DLBCL with limited alternatives. Comorbidities (DM, AHT) must be controlled before therapy.

**===== run_3 =====**

**Tumor board decision:**
There is an indication for CAR T-cell therapy in post-CHOP recurrence. Prioritization of CAR-T over salvage chemo/ASCT due to FDG-avid recurrence burden and early progression after R-Pola-CHP.

**Rationale:**
DLBCL Non-GCB with extranodal recurrence after 6 cycles of R-Pola-CHP. PET-CT shows metabolically active recurrence (SUVmax 17). CAR-T is established in the post-CHOP recurrence situation in FDG-avid lesions. Salvage chemo/ASCT remains an option, but early progression argues for CAR-T prioritization. Comorbidities (AHT, DM) require preclinical toxicity assessment.

## ============================================================ qwen3-next-80b (agent) ============================================================

**===== run_1 =====**

**Tumor board decision:**
Yes, CAR T-cell therapy is indicated. Metabolic progression after R-Pola-CHP within <12 months (early relapse) meets the criteria for CAR-T as standard therapy. Clinical suitability (fitness, comorbidities) must be checked in advance, but AHT and DM type II are not an absolute contraindication. The planned ENT biopsy for histological confirmation of the recurrence is required.

**Rationale:**
According to the flowchart (Section V.P/R-3), if progression is <=12 months after first-line, CAR-T is the first choice if suitable. No age cutoff, comorbidities (AHT, DM II) are not an exclusion criterion. R-Pola-CHP was first therapy, metabolic PD confirmed. Biopsy required for backup, then

plan CAR-T bridging.

**===== run_2 =====**

**Tumor board decision:**
CAR T-cell therapy is indicated after histological confirmation of recurrence by ENT biopsy. Prepare bridging therapy in accordance with the lymphoma Tumor board decision.

**Rationale:**
Early relapse (<=12 months after first-line therapy) after R-Pola-CHP with metabolic progression (DS5). Flowchart V.P/R-3 defines CAR-T as standard therapy if suitable. Comorbidities (AHT, DM II) are not a contraindication. Re-biopsy required for confirmation. There is no age limit for CAR-T, suitability must be assessed individually.

**===== run_3 =====**

**Tumor board decision:**
Yes, CAR-T therapy is indicated. Beforehand, carry out histological confirmation of the recurrence (ENT biopsy) and CAR-T suitability test. If suitable: CAR-T as standard therapy for early recurrence after first-line therapy.

**Rationale:**
According to the flowchart (V.5) for progression <=12 months after first line (R-Pola-CHP), CAR-T is the first choice if suitable. Histological confirmation is prioritized. Comorbidities (AHT, DM II) are not an absolute contraindication, provided they are stable. CAR-T indication applies regardless of age, but organ function and performance status must be checked.

## ============================================================ qwen3-next-80b (plain) ============================================================

**===== run_1 =====**

**Tumor board decision:**
Indication for CAR-T therapy confirmed. Once recurrence is confirmed by biopsy, plan immediate referral for CAR T-cell therapy (e.g. axicabtagene ciloleucel). Consider bridging therapy for symptomatic progression.

**Rationale:**
Primary refractory DLBCL after R-Pola-CHP with early recurrence (<12 months) is a clear indication for CAR-T. Comorbidities (AHT, DM2) are not contraindications. Current guidelines (NCCN, ESMO) recommend CAR-T as standard therapy in this situation.

**===== run_2 =====**

**Tumor board decision:**
Yes, CAR-T therapy is indicated. Prior confirmation of recurrence by biopsy, then referral for CAR-T transplantation.

**Rationale:**
Early recurrence after R-Pola-CHP (3 months) is high risk. CAR-T is standard therapy for first-line relapsing DLBCL. Comorbidities (AHT, DM II) are manageable and are not a contraindication. Biopsy confirmation required.

**===== run_3 =====**

**Tumor board decision:**
CAR-T therapy indicated after histological confirmation of early recurrence. Perform ENT biopsy, then initiate CAR-T transplant.

**Rationale:**
Early recurrence after R-Pola-CHP (within 12 months) is an indication for CAR-T. Comorbidities (AHT, DM II) are not an exclusion criterion. CAR-T is standard for primary refractory or early relapsed DLBCL after first-line therapy.

# ly_case_6__ALL

################################
#### CASE: 6
################################

## ============================================================ gpt-5-mini (agent) ============================================================

**===== run_1 =====**

**Tumor board decision:**
Yes - histological confirmation is recommended before initiating systemic salvage therapy/allo-SCT/study inclusion. Primary: CT-guided transthoracic core biopsy of a peripheral FDG-active nodule; if central: bronchoscopic biopsy including BAL. Pathology: H&E, IHC (CD19, CD20, CD79a, Ki-67), clonality, FISH; parallel microbiological/PNP clarifications.

**Rationale:**
Re-biopsy is SOP/flowchart standard in case of progression/recurrence (also post-CAR-T). Goal: Clarify tumor vs. inflammation/infection, check CD19/CD20 status for further CD19-targeted therapies/BiTE/allo-SCT and confirm any histological changes/therapy decision. If a biopsy is impossible: comprehensive infectious disease clarification and interdisciplinary decision-making.

**===== run_2 =====**

**Tumor board decision:**
Yes. Before starting systemic salvage therapy, allo-SCT or study inclusion, the pulmonary FDG-active nodules should be histologically confirmed (primarily CT-guided percutaneous core biopsy or bronchoscopically). Additional: IHC for CD19/CD20, FISH, NGS and microbiological examinations.

**Rationale:**
After progression after CD19-CAR-T, clarification: malignant vs inflammation/infection and recording of the CD19/CD20 status are mandatory, as CD19 loss influences therapy options (BiTEs, renewed CD19-targeted approaches, allo-SCT) and study screening. Lung biopsy is usually safe and crucial to treatment.

**===== run_3 =====**

**Tumor board decision:**
Before initiating systemic salvage therapy, allo-SCT or study inclusion: histological confirmation of the new pulmonary FDG-active nodules (CT-guided punch biopsy or bronchoscopic biopsy). Request pathology including immunohistochemistry and flow cell analyzes (at least CD19, CD20, Ki-67).

**Rationale:**
After CAR-T, there is risk of antigen loss (CD19) and DD from inflammation/infection. Re-biopsy is SOP/guideline compliant, clarifies tumor progression and provides crucial target antigen information for choosing BiTE vs anti-CD19 strategies or allo-SCT/studies.

## ============================================================ gpt-5-mini (plain) ============================================================

**===== run_1 =====**

**Tumor board decision:**

**Tumor board decision:**
Targeted new diagnosis (fresh biopsy with IHC/Flow: CD19/CD20) and immediate possibility of inclusion in a clinical study or start of a CD20xCD3 bispecific (e.g. epcoritamab/mosunetuzamab/glofitamab) if available. If there is a response and good performance: consolidation using allogeneic stem cell transplantation. If not available/unsuitable: palliative systemic therapy/supportive care.

**Rationale:**
Progression after CD19-CAR-T shows refractory course; CD19 loss possible. CD20xCD3 bispecifics have shown good effectiveness in this situation. Allo-SCT remains potentially curative in responsive, fit patients. Polatuzumab has already been given and should not be used again.

**===== run_2 =====**

**Tumor board decision:**

Recommendation: Starting CD20xCD3 bispecific therapy (e.g. epcoritamab/glofitamab/mosunetuzumab) preferably as part of a study/early access. If not available and rapidly progressing: bridging chemotherapy (e.g. GemOx or GDP) until treatment begins. No repeat of polatuzumab or CD19-targeted therapies. For CR: Evaluation for Allo-SCT.

**Rationale:**
Patient with PD after R-Pola-CHP and CD19-CAR-T (zamto-cel) - CD19 resistance/antigen loss possible. CD20xCD3 bispecifics show documented activity after CAR-T. Polatuzumab has already been used and should not be used again. Bridging chemo controls tumor burden; Allo-SCT only if remissions have been achieved and suitability.

**===== run_3 =====**

**Tumor board decision:**
After progression 4 months after CD19-CAR-T, we primarily recommend inclusion in a clinical trial or rapid access to a CD20xCD3 bispecific (e.g. epcoritamab, glofitamab, mosunetuzumab). If not available and in case of symptomatic progression: short-cycle bridging chemotherapy (e.g. GemOx or GDP) and in remission testing for Allo-SCT. No repetition of polatuzumab or renewed CD19-targeted therapy.

**Rationale:**
After CAR-T failure, CD20xCD3 bispecifics show promise and circumvent common CD19 loss mechanisms. Polatuzumab has already been used and re-challenge appears unlikely to be effective. Clinical trial is priority; Bridging chemo can stabilize symptoms and enable possible allo-SCT.

## ========================================================== gpt-5-nano (agent) ==========================================================

**===== run_1 =====**

**Tumor board decision:**
Recommendation: Salvage with polatuzumab vedotin-based regimens (Pola-BR) or tafasitamab+lenalidomide; alternatively bispecifics (glofitamab/epcoritamab) depending on availability. Check Re-CAR-T in appropriate centers. If remission is achieved, consolidate allogeneic stem cell transplantation; otherwise consider further salvage options.

**Rationale:**
Following CAR-T progression in transformed DLBCL-GCB, data are limited. Evidence supports polatuzumab-BR, tafasitamab+lenalidomide, and bispecifics as salvage; AlloSCT consolidation useful in remission and transplantability. Re-CAR-T possible, data limited. Organ findings (liver/lung) and tumor burden control choice and transplantability.

**===== run_2 =====**

**Tumor board decision:**
Consider allogeneic stem cell transplantation (fitness/donor) as a primary option; Bridging salvage with Pola-R-Benda or tafasitamab+lenalidomide. If alloSCT is not possible or remission is unlikely: CD19-targeted salvage (glofitamab, epcoritamab, loncastuximab), Pola+Benda; prefer clinical trials; Consider CD19 status and extranodal involvement.

**Rationale:**
Post-CAR-T progression in transformed DLBCL NOS with liver/lung involvement and Ki-67 ~90% requires aggressive, potentially curative approach. alloSCT offers a chance of recovery in the event of remission; Salvage bridging makes sense to enable transplant eligibility. Sensibly check CD19 targets; Pay attention to study preferences.

**===== run_3 =====**

**Tumor board decision:**
Salvage after CAR-T progression: 1) Pola-BR preferred; 2) Alternatives: tafasitamab+lenalidomide or glofitamab/epcoritamab; 3) Allogeneic stem cell transplantation as consolidation in remission; 4) Review studies.

**Rationale:**
IVAE, extranodal liver/lung involvement, LDH high, Ki-67 90%, PET DS5; aggressive, after CAR-T progression. Pola-BR evidence-based; AlloSCT potentially curative in remission; Prioritize trials.

## ========================================================== gpt-5-nano (plain) ===========================================================

**===== run_1 =====**

**Tumor board decision:**
Recommendation: Conduct a clinical study or therapy with a CD20xCD3 bispecific (e.g. glofitamab) or loncastuximab tesirine; RT only palliative adjunct for symptoms.

**Rationale:**
PD after CAR-T; Pola-CHP preceded; possible antigen escape. New bispecifics/ADC show activity in R/R DLBCL after >=2 lines; Study preferred.

**===== run_2 =====**

**Tumor board decision:**
Recommendation: Enrollment in a clinical trial with new immunotherapies (e.g. CD20xCD3 bispecific) or access to an approved immune/targeted therapy (loncastuximab tesirine or tafasitamab+lenalidomide). If remission is achievable, consider allogeneic stem cell transplantation; otherwise continue supportive therapy.

**Rationale:**
PD after CAR-T, multiple previous lines, no longer a sustainable response. Allogeneic SCT can be curative with adequate remission/performance status; Trials offer additional options with potential for remission/control. Priority: Trial or new immunotherapy; SCT only if there is remission or sufficient performance.

**===== run_3 =====**

**Tumor board decision:**
Recommendation: Participation in a clinical trial (e.g. bisspecifics or loncastuximab) or second-line systemic therapy with a different active approach (e.g. polatuzumab vedotin + rituximab + bendamustine) depending on availability. In the case of oligoprogressive lung precipitates, local RT may be added if necessary. Allogeneic transplantation should only be considered if remission is achievable and sufficient conditioning is achieved.

**Rationale:**
After CAR-T progression in DLBC lymphoma, the prognosis is poor; new mechanisms in studies or possibly Pola/ADC-based are favored. Local RT for focal progression is used to control symptoms. Allogeneic KS transplantation only if remission can be achieved and the patient is transplantable.

## ============================================================ gpt-oss-20b (agent) ============================================================

**===== run_1 =====**

**Tumor board decision:**
Second CAR-T therapy with axi-cel (CD19-targeted) as the first option; alternatively bispecific CD3xCD20 antibody epcoritamab. If the response is inadequate or unsuitable for CAR-T: allo-SCT after intensive pretreatment.

**Rationale:**
Patient PD after Zamtocel-CAR-T; Phase III data show >=30% ORR in >=2 lines. Epcoritamab has ORR~50% in R/R-DLBCL without CD30. Both options are evidence-based; allo-SCT remains in reserve for suitable candidates.

**===== run_2 =====**

**Tumor board decision:**
Patients with PD after zamtocel-CAR-T are initially evaluated for a second CAR-T therapy (e.g. lisocabtagene maraleucel or brexucabtagene autoleucel). If CAR-T is not suitable or there is no remission, participation in a clinical trial with a bispecific antibody (glofitamab) or ADC (loncastuximab-tesirine) is recommended. If remission is achieved, allo-SCT can then be considered.

**Rationale:**
The patient is PD after Zamtocel-CAR-T, therefore repeat CD19-CAR-T therapy is the preferred option according to guidelines (P/R-3). If not CAR-T capable, bispecific antibodies or ADCs are the evidence-based alternatives (P/R-3). In chemosensitive remission, allo-SCT is the last option.

**===== run_3 =====**

**Tumor board decision:**
PD after Zamtocel-CAR-T: Start of bispecific CD19/CD3 antibodies (e.g. glofitamab) or ADC loncastuximab-tesirine as initial salvage. If the organic reserve is good (ECHO, kidneys),

R-DHAP/R-ICE -> allo-SCT follows. Alternatively, check CAR-T-2.0 studies (ZUMA-5, CARTITUDE-2).

**Rationale:**
Patient has already received CAR-T, therefore repeated CAR-T only if fit. Bispecific antibodies/ADC show high activity in CAR-T-resistant DLBCL in phase 2 studies. After remission, allo-SCT can be considered as a curative option. The choice of study is based on current evidence.

## ========================================================== gpt-oss-20b (plain) ==========================================================

**===== run_1 =====**

**Tumor board decision:**
Start salvage therapy with tafasitamab + lenalidomide (TCL) as the first option. If progression occurs up to 3-4 cycles, consider bispecific antibody therapy (e.g. mosunetuzumab) or a second CAR-T therapy (idecabtagene vicleucel).

**Rationale:**
DLBCL has demonstrated PD after CAR-T therapy; TCL has demonstrated an ORR of ~50% and good tolerability in the second line after CAR-T. If progression continues, bispecific antibodies or a second CAR-T therapy are the next logical steps.

**===== run_2 =====**

**Tumor board decision:**
Salvage therapy with polatuzumab vedotin + bendamustine + rituximab (Pola-BR) followed by ASCT if CR/PR; In case of ASCT intolerance, alternative tafasitamab + lenalidomide.

**Rationale:**
PD after CAR-T, Pola-BR shows high activity in CAR-T-resistant DLBCL; ASCT remains standard while maintaining remission; Tafasitamab+lenalidomide is an alternative option for ASCT intolerance.

**===== run_3 =====**

**Tumor board decision:**
After progression after Zamtocabtagene-CAR-T, salvage chemotherapy (e.g. ICE or DHAP) is recommended, followed by ASCT evaluation. If response is inadequate, consider alternative CAR-T (e.g. brexucabtagene) or clinical trial.

**Rationale:**
The patient has PD (DS5) after Zamtocabtagene-CAR-T with new pulmonary lesions, LDH normal, indicating lack of remission. Salvage chemo followed by ASCT remains the standardized option in chemosensitive DLBCL patients. If remission is not achieved, further CAR-T approaches or studies are the next sensible alternative.

## ========================================================== gpt-oss-120b (agent) ==========================================================

**===== run_1 =====**

**Tumor board decision:**
Allogeneic stem cell transplantation (alloSCT) after bridging salvage (e.g. R-ICE) - alternatively, participation in a study with a CD20xCD3 bispecific (epcoritamab/glofitamab) or a second CD19 CAR T if a suitable donor or product is available.

**Rationale:**
Patient has progressive DLBCL with high IPI and extranodal lesions after R-Pola-CHP and CD19-CAR-T. Further CD19-targeted procedures (second CAR-T) or purely chemical salvage regimens have low chances of success; alloSCT offers potential cure with appropriate fitness. Bispecific antibodies are evidence-based options after CAR-T failure and can be used as a bridge therapy or study option.

**===== run_2 =====**

**Tumor board decision:**
We recommend salvage chemotherapy (e.g. R-DHAP or R-ICE) for remission, followed by an allogeneic stem cell transplant if the patient is fit and a suitable donor is available. If a transplant is not possible, CD20-CD3 bispecific antibody therapy (e.g. glofitamab/epcoritamab) or loncastuximab should be used in an appropriate study or commercial setting.

**Rationale:**

The patient has already received CAR-T and has progressive DLBCL. According to the current guideline flowchart, salvage chemotherapy followed by alloSCT is the only potentially curative option after CAR T failure. If they are not suitable for transplantation, bispecific antibodies approved under CAR-T offer an evidence-based alternative.

**===== run_3 =====**

**Tumor board decision:**
In transplant-eligible patients, we recommend rapid bridging therapy with a CD20-CD3 bispecific (e.g. glofitamab+/-R-CHOP-like adjunctive chemotherapy) to achieve a remission bridge state, followed by an allogeneic stem cell transplant. If unsuitable, a clinical trial with bispecific antibodies or an alternative CAR-T (e.g. CD20-CAR-T) should be initiated.

**Rationale:**
After failure of CD19-CAR-T, CD20-CD3 bispecifics show high response rates in studies and enable effective bridging to allo-SCT, which offers the best long-term control in high-IPI-DLBCL (similar cases, current literature). Allogeneic SCT remains curative, while alternative CAR-T or study options are useful in cases of intransplantability.

## ========================================================== gpt-oss-120b (plain) ==========================================================

**===== run_1 =====**

**Tumor board decision:**
Recommendation: Intensive salvage therapy (e.g. R-ICE, DHAP or Pola-Bendamustine) to achieve remission, followed by allogeneic stem cell transplantation if there is a response. If there is no chemosensitivity, take part in a study with bispecific antibodies/ADCs (e.g. glofitamab, loncastuximab).

**Rationale:**
DLBCL is progressive (PD, DS5) after R-Pola-CHP and CAR-T (zamtocabtagene). Further standard chemotherapy is the only way to achieve remission for a potentially curative allo-SCT. If there is no response rate, current bispecific antibody or ADC studies offer a useful alternative.

**===== run_2 =====**

**Tumor board decision:**
Patient is refractory (PD) to R-CHOP, R-polatuzumab-CHP and CAR-T (zamtocabtagene). Recommendation: Immediate evaluation of transplantability and, if suitable for transplant, implementation of an allogeneic stem cell transplant. If transplantation is not possible, inclusion in a clinical trial with a CD20xCD3 bispecific (e.g. glofitamab) or other novel agents.

**Rationale:**
Despite intensive standard and CAR-T therapy, the disease is progressive, IPI is high and extranodal involvement persists. Allogeneic SCT offers potentially curative graft vs lymphoma effects. If unsuitable, bispecific antibody therapy is the most evidence-based option in the rescue situation.

**===== run_3 =====**

**Tumor board decision:**
Due to progressive disease after R-Pola-CHP and CAR-T (Zamtocabtagene), allogeneic stem cell transplantation should be considered as a potentially curative option, provided the patient is fit and a suitable donor is available. If this is not feasible, participation in a clinical trial with bispecific antibodies (e.g. glofitamab, epcoritamab) or other novel therapies is strongly recommended.

**Rationale:**
The patient has a high-risk DLBCL (IPI high, GCB type) in stage IV, which continues to progress after several lines including CAR-T. Effective standard salvage therapy no longer available; Allo-HSCT offers the only potential cure, otherwise experimental options.

## ========================================================== qwen3-32b (agent) ==========================================================

**===== run_1 =====**

**Tumor board decision:**

After PD under CAR-T, we recommend blinatumomab (BiTE) as the first choice. Alternatively, glofitamab/epcoritamab or VIPOR (off-label) should be considered. Clinical trials must be prioritized.

**Rationale:**
PD after CAR-T requires CD19/CD20 expression confirmation (not documented, but typical of B-cell lymphoma). According to the flowchart, BiTE therapies (blinatumomab) are the first choice. If penetration in solitary tumors is poor, Fc-optimized bispecifics (glofitamab) could be preferred. VIPOR is an off-label alternative. Study access is critical.

**===== run_2 =====**

**Tumor board decision:**
Recommendation: Glofitamab+GemOx as first line after CAR-T progression. If responsive, consider allo-SCT. Alternatively, use epcoritamab (subcutaneous) or ViPOR combination therapy (GCB-specific).

**Rationale:**
PD after CAR-T requires new approaches. Glofitamab+GemOx has phase 3 evidence, epcoritamab offers subcutaneous administration. ViPOR is GCB specific. Allo-SCT remains relevant in the perspective of clinical response.

**===== run_3 =====**

**Tumor board decision:**
Recommendation: Glofitamab-based therapy (monotherapy or in combination with GemOx) as first line after CAR-T failure. If responsive, consider allo-SCT as a bridge. Alternatively, ViPOR combination therapy (GCB-specific) or epcoritamab.

**Rationale:**
PD after CAR-T in GCB-DLBCL requires new immunotherapy approaches. Glofitamab (bispecifics) and ViPOR show high effectiveness in the literature. Allo-SCT remains relevant in the future. PD-1/CD30 ADC from similar cases not evidence-based for GCB type.

## ============================================================ qwen3-32b (plain) ============================================================

**===== run_1 =====**

**Tumor board decision:**
Recommendation: Participation in a clinical trial with novel targeted therapies (e.g. BCL-2 inhibitors, BTK inhibitors) or alternative CAR-T constructs. If you do not participate in the study: combination therapy with ibrutinib + venetoclax + rituximab.

**Rationale:**
After CAR-T resistance and PD despite R-Pola-CHP, aggressive 2L therapy is required. BCL-2 overexpression (common in GCB-DLBCL) and BTK inhibition may act synergistically. Study participation prioritized because standard options have been exhausted.

**===== run_2 =====**

**Tumor board decision:**
Recommendation: Participation in a clinical trial with innovative therapies (e.g. bifunctional antibodies, targeted therapy) or salvage chemotherapy (R-GemOx) followed by possible consolidation. CAR-T resistance taken into account.

**Rationale:**
PD after CAR-T and R-Pola-CHP despite high Ki-67. Aggressive GCB-DLBCL with extranodal involvement. Clinical studies (e.g. BTK inhibitors, CD19/CD20 dual targeting) are the first choice. Salvage chemotherapy (R-GemOx) as a bridge to possible consolidation.

**===== run_3 =====**

**Tumor board decision:**
Recommendation: Participation in a clinical trial with novel targeted therapy (e.g. BTK inhibitor or CD19/CD20 bispecific antibody) after multidisciplinary assessment

**Rationale:**
After CAR-T failure and progression on R-Pola-CHP, individualized study participation with innovative therapies (e.g. BTK inhibitors or bispecifics) makes sense. Standard options have been exhausted, prognosis remains good despite PD (normal LDH).

## ============================================================ qwen3-next-80b (agent) ============================================================

**===== run_1 =====**

**Tumor board decision:**
Starting bispecific antibody therapy (BiTE) such as glofitamab or epcoritamab after re-biopsy to confirm CD20 expression. Check clinical studies in parallel (e.g. CAR-T resistance studies).

**Rationale:**
After CAR-T progression, BiTE (CD20/CD3) is the first choice according to the flowchart. Re-biopsy required to confirm CD20 expression. Tafasitamab/lenalidomide or ADCs (e.g. loncastuximab) are alternatives, but BiTE has higher evidence in CAR-T progression. No contraindications (no comorbidities).

**===== run_2 =====**

**Tumor board decision:**
CD20xCD3 bispecific antibody therapy (e.g. glofitamab or epcoritamab) after re-biopsy to confirm CD20 expression and staging. Review clinical trials.

**Rationale:**
According to the flowchart, BiTE therapy is the first choice after CAR-T progression. Re-biopsy required to confirm CD20 expression. Glofitamab/Epcoritamab are approved for R/R DLBCL after CAR-T. Study options (e.g. new BiTEs) should be evaluated in parallel.

**===== run_3 =====**

**Tumor board decision:**
BiTE therapy (e.g. glofitamab or epcoritamab) after re-biopsy to confirm CD20 expression

**Rationale:**
According to the flowchart, BiTE therapy is the first choice after CAR-T progression. Re-biopsy required for CD20 expression testing. Prioritization before alloSCT/2. CAR-T due to higher effectiveness and better tolerability. No prior BiTE exposure.

## ============================================================ qwen3-next-80b (plain) ============================================================

**===== run_1 =====**

**Tumor board decision:**
Bispecific antibody (glofitamab) or clinical trial

**Rationale:**
CAR-T progression after R-Pola-CHP. Bispecific antibodies such as glofitamab are approved for DLBCL after >=2 lines of therapy and show efficacy in the post-CAR-T setting. Alternative clinical trial.

**===== run_2 =====**

**Tumor board decision:**
Starting a CD20/CD3 bispecific antibody therapy (e.g. glofitamab) or joining a clinical trial.

**Rationale:**
Progression after CAR-T (zamto-cel) as 2nd line. CD19-targeted therapies (e.g. loncastuximab) are unlikely to be effective. Bispecific antibodies (CD20/CD3) are evidence-based options for 3rd line after CAR-T. No contraindications, no comorbidities.

**===== run_3 =====**

**Tumor board decision:**
Recommend therapy with a CD20xCD3 bispecific antibody (e.g. glofitamab or epcoritamab) or participation in a clinical trial.

**Rationale:**
After CAR-T progression, bispecific antibodies are the first choice. Glofitamab shows high response rate in post-CAR-T patients. No known contraindications. Alternatively, clinical studies.

# ly_case_7__ALL

################################
#### CASE: 7

################################

**================================================================ gpt-5-mini (agent) ================================================================**

**===== run_1 =====**

**Tumor board decision:**
Primary: Re-biopsy (histology including IHC for CD19/CD20, Ki-67, PD-L1), FISH, NGS, FACS as well as PET-CT and organ/cardio workup for allo-suitability. Therapy recommendation, in order: 1) Bispecific antibody (e.g. glofitamab/epcoritamab) or appropriate study; 2) anti-CD19 options (loncastuximab; 2. CAR-T only selective); 3) Allo-SCT in chemosensitive remission. Checkpoint inhibitor only for defined indications; Critically assess polatuzumab re-exposure.

**Rationale:**
Early progression ~3 months after CAR-T requires rapid restaging and biopsy to clarify CD19 status. Bispecific antibodies are currently considered the preferred option after CAR-T failure; anti-CD19 ADC or second CAR-T are possible subsequently. Allo-SCT remains a consolidation option if remission is achieved and good fitness is achieved. Checkpoint inhibitors have limited evidence.

**===== run_2 =====**

**Tumor board decision:**
Immediate further diagnostics: Re-biopsy including Histo/IHC (CD19, CD20), FISH and NGS as well as PET-CT. Therapy recommendation: For CD20+, primary bispecific anti-CD20xCD3 antibodies (e.g. glofitamab/epcoritamab). If not available or contraindicated: Polatuzumab-bendamustine+/-R or study. Allo-SCT or 2nd CAR-T only in chemosensitive remission and after conference/study.

**Rationale:**
Early progression ■ high probability of limited CAR-T persistence/antigen loss; Re-biopsy clarifies CD19/CD20 and driving mutations. Guidelines/flowchart prioritize bispecifics after CAR-T failure; Pola-based therapy and studies as alternatives. Allo-SCT/2. CAR-T only with stable treatment success/transplant suitability.

**===== run_3 =====**

**Tumor board decision:**
Immediate re-biopsy (histology, IHC CD19/CD20), FISH, NGS + PET-CT restaging and determination of CAR-T persistence/B-cell aplasia. Therapy recommendation: for CD19+, primary bispecific antibodies (glofitamab/epcoritamab) or loncastuximab; for CD19-, polatuzumab-based regimen (Pola-R/Benda) or study inclusion is preferred. Allo-SCT only after renewed remission and suitability; second CAR-T only within the framework of studies/for specific indications. Checkpoint inhibitors not routine.

**Rationale:**
Early progression ■ CAR-T failure due to antigen loss or aggressive subclones. Re-biopsy and NGS clarify target antigen status and driver mutations and guide therapy. Bispecifics show good activity after CAR-T; Pola regimen is an option in cases of CD19 loss or if rapid cytoreductive therapy is necessary. Allo-SCT only with chemosensitive remission and patient suitability; Re-CAR-T currently experimental.

**================================================================ gpt-5-mini (plain) ================================================================**

**===== run_1 =====**

**Tumor board decision:**
Recommendation: Priority inclusion in a clinical study with a CD20xCD3 bispecific (e.g. glofitamab/epcoritamab). If study not available: start bispecific therapy (off-label). Alternatively, if there is no option: short-cycle salvage therapy (e.g. GemOx/GDP +/- Rituximab) as bridging and, if there is a response, evaluation for allogeneic stem cell transplantation. Consider tafasitamab+lenalidomide only if CD19 expression is confirmed.

**Rationale:**

Patient is refractory to R-CHOP, polatuzumab-bendamustine and CD19-CAR-T. After CAR-T, non-cross-resistant immunotherapy (CD20xCD3 bispecifics) makes the most sense; Studies have shown highest effectiveness in post-CAR-T scenarios. Allo-SCT only if remission can be achieved again and sufficient fitness is achieved.

**===== run_2 =====**

**Tumor board decision:**
Priority: Inclusion in a clinical trial with bispecific CD20xCD3 antibodies (e.g. epcoritamab/glofitamab). If not available: tafasitamab + lenalidomide. In case of rapid/symptomatic progression, short bridging chemotherapy; in case of remission, reevaluation and, if necessary, allo-SCT in patients eligible for transplantation.

**Rationale:**
Early failure after CAR-T (PD ~3 months) requires non-overlapping immunotherapy; bispecific antibodies show effectiveness after CAR-T. Tafasitamab+lenalidomide is an acceptable alternative. Bridging for disease control and, if there is subsequent remission, consideration of an allo-SCT if the condition is suitable.

**===== run_3 =====**

**Tumor board decision:**
Urgent re-biopsy (including CD19/CD20 IHC/Flow) and PET-CT. Recommendation: inclusion in a clinical study with CD20xCD3 bispecifen (e.g. glofitamab/epcoritamab/mosunetuzumab). If study not available: for CD19+ option Loncastuximab-tesirine. If response is good and fit, consider Allo-SCT as consolidation.

**Rationale:**
Early progression <3 months after CAR-T = poor prognosis. Patient had R-CHOP, Pola+Benda and CAR-T; new biopsy clarifies target antigen status. CD20xCD3 bispecifics show good activity after CAR-T; CD19-targeted ADCs only when CD19 is preserved. Allo-SCT only if remission can be achieved and good condition.

## =============================================================== gpt-5-nano (agent) ===============================================================

**===== run_1 =====**

**Tumor board decision:**
After CAR-T progression: Check CD19 status. If CD19 positive: Loncastuximab tesirine or tafasitamab + lenalidomide; if necessary, glofitamab/epcoritamab; Allogeneic transplantation with good PS; Prefer studies.

**Rationale:**
prioritization of CD19-targeted therapies when CD19 expression is present; Sequence depends on previous therapies, PS and study access. CD19 negative: selinexor; AlloSCT in appropriate cases.

**===== run_2 =====**

**Tumor board decision:**
After progression after lisocabtagene maraleucel: check CD19 status. If CD19+: Loncastuximab tesirine or tafasitamab+lenalidomide; if necessary, CD20 bispecifics (glofitamab/epcoritamab); selinexor or clinical trials; Allogeneic transplantation in remission/eligibility.

**Rationale:**
After CAR-T progression, it makes sense to prioritize CD19/CD20 targets; Transplantation as salvage with suitable PS.

**===== run_3 =====**

**Tumor board decision:**
After CAR-T progression (lisocabtagene), recommend: CD20/CD3 bispecifics (glofitamab or epcoritamab) if CD20 expression is detectable. If CD19 expression is preserved, tafasitamab+lenalidomide or loncastuximab tesirine. Consider allogeneic transplantation with suitable remission/donor. Prefer clinical studies.

**Rationale:**
Evidence from STARGLO/EPcoritamab-like studies shows activity of CD20xCD3 bispecifics after CAR-T progression. Allogeneic transplantation makes sense if the donor/donor has a good remission. CD19-targeted options only make sense if CD19 is present. Priority clinical studies and exploratory

approaches.

## ============================================================ gpt-5-nano (plain) ============================================================

**===== run_1 =====**

**Tumor board decision:**
After PD after CAR-T (lisocabtagene), salvage therapy outside of CAR-T is recommended. Preferably Loncastuximab tesirine (CD19-ADC); alternatively tafasitamab + lenalidomide or a relevant clinical study. If remission is achieved, consider allogeneic transplantation; otherwise continue systemic salvage.

**Rationale:**
Progression after CAR-T in transformed DLBCL; aggressive progression, several leading lines. CD19-targeted salvage makes sense; Consideration of clinical trials and transplant options in remission or sufficient condition.

**===== run_2 =====**

**Tumor board decision:**
Consider allo-SCT as a potentially curative step after CAR-T failure (donor present, performance status good). If not suitable: Second post-CAR T line with loncastuximab tesirine or CD20xCD3 bispecifics (e.g. glofitamab/epcoritamab) +/- RT for bulky disease.

**Rationale:**
CAR-T failure in transformed FL-DLBCL: Allo-SCT can provide cure provided donor status and good function. Alternatives such as loncastuximab tesirine or CD20xCD3 bispecifics show activity but limited healing potential. Decision depends on availability, status and donor.

**===== run_3 =====**

**Tumor board decision:**
Prefer clinical trial. If not available: CD20xCD3 bispecific (e.g. glofitamab) or tafasitamab-lenalidomide; if necessary, palliative RT for focal progression; supportive care.

**Rationale:**
PD according to CAR-T-Breyanzi; refractory DLBCL. Clinical trials offer new targets/mechanisms; If CD19 coverage is problematic, bispecifics or lenalidomide-based approaches increase potential. RT may be useful if symptoms progress.

## ============================================================ gpt-oss-20b (agent) ============================================================

**===== run_1 =====**

**Tumor board decision:**
Start glofitamab (CD19/CD3-BiTE) as first option after progression after lisocabtagene maraleucel; If the response (CR/PR) is insufficient, immediately check alloSCT candidacy.

**Rationale:**
Flowchart-P/R-nach-CAR-T recommends BiTE as 1st choice; Glofitamab has positive data after CAR-T progression. In the absence of remission, alloSCT remains the only curative option. The patient is fit (no contraindications), therefore suitable.

**===== run_2 =====**

**Tumor board decision:**
Starting BiTE therapy (e.g. glofitamab) as the first salvage therapy after progression post-Lisocabtagene-CAR-T. If CR/PR is obtained, we evaluate alloSCT; If there is no response, we examine a second CAR-T therapy (tisagenlecleucel/axicabtagene).

**Rationale:**
Patient has PD after CAR-T, is fit, no contraindications. BiTE therapies (glofitamab, epcoritamab) show high response rates in CAR-T progression and are well tolerated. AlloSCT remains an option for CR/PR; second CAR-T only if there is no response.

**===== run_3 =====**

**Tumor board decision:**

Patients with progression after lisocabtagene maraleucel should initially be treated with high-dose chemotherapy (e.g. R-DHAP) as salvage therapy. If CR/PR is obtained, an allogeneic stem cell transplant (alloSCT) is then performed. If alloSCT is not possible, a bispecific T cell engager (e.g. glofitamab) is recommended as an alternative option.

**Rationale:**
The patient has already received R-CHOP, R-Pola-Benda and CAR-T and is therefore CAR-T resistant. High-dose chemotherapy followed by alloSCT is the evidence-based 2nd line for CAR-T-progressive DLBCL. If transplantability is not possible, a BiTE such as glofitamab offers an effective, less stressful alternative.

## ============================================================ gpt-oss-20b (plain) ============================================================

**===== run_1 =====**

**Tumor board decision:**
Salvage-DHAP/ICE -> CR -> allogeneic SCT. If SCT is not possible, consider clinical trial with CD19/CD3 bispecific antibody (e.g. mosunetuzumab) or second CD19 CAR-T therapy.

**Rationale:**
Patient has progression after CAR-T therapy; no further CD19 therapy available. Salvage chemotherapy can achieve remission, which serves as a bridge to allogeneic SCT - the only curative option. Clinical studies are useful as an alternative in cases of SCT incompatibility.

**===== run_2 =====**

**Tumor board decision:**
After progression after lisocabtagene-maraleucel, a second CAR-T therapy (idecabtagene vicleucel) is recommended, if available. If not, an intensive salvage regimen (R-CHOP+Polatuzumab) followed by ASCT should be considered.

**Rationale:**
Patient showed CR after CAR-T, but PD 11/18/2022. As a non-GCB-DLBCL with high Ki-67 and MYC-negative, the second CAR-T therapy is the preferred option for CAR-T resistance. If not accessible, an intensive salvage regimen followed by ASCT is the next evidence-based alternative.

**===== run_3 =====**

**Tumor board decision:**
Further treatment with salvage chemotherapy (e.g. R-ICE) for remission, followed by allogeneic stem cell transplant if fitness is good; alternatively, participation in a clinical trial (tafasitamab+lenalidomide) or other salvage regimens.

**Rationale:**
Patient showed progression after CAR-T; no other effective monotherapy available. R-ICE offers remission rates ~30-40% and serves as a bridge to the transplantable state. Allogeneic SCT is the only potentially curative option for recurrent relapse.

## ============================================================ gpt-oss-120b (agent) =============================================================

**===== run_1 =====**

**Tumor board decision:**
In the case of progressive DLBCL after CAR-T therapy, a new biopsy should first be carried out to determine CD19/CD20. If the tumor is CD19-positive and a suitable donor is available, the next therapy recommended is an allogeneic stem cell transplant (after bridging salvage, e.g. R-DHAP or R-Pola-Benda). If there is no donor or is CD19 negative, the patient should be included in a study with CD19 BiTEs (e.g. epcoritamab, glofitamab) or a CD19 ADC (loncastuximab).

**Rationale:**
The patient is fit, has already received R-CHOP, Pola-Benda and CAR-T and does not show a standard further treatment concept after CAR-T failure. Allogeneic SCT offers potential cure provided there is a donor and adequate organ function. A biopsy clarifies CD19 escape; If there is CD19 negativity or there is no donor, current studies with bispecific antibodies or ADCs are the most sensible option.

**===== run_2 =====**

**Tumor board decision:**

We recommend initiating therapy with a CD20-CD3 bispecific (e.g. glofitamab or epcoritamab) after progression on lisocabtagene-maraleucel, preferably as part of a clinical trial, and considering allogeneic stem cell transplantation as a potentially curative option if there is a response.

**Rationale:**
The patient is refractory to R-CHOP, Pola-Benda and CAR-T. Bispecific antibodies show CR rates of 30-35% after CAR-T failure with acceptable safety. Allo-SCT is only useful if there is remission; the bispecific can serve as a bridge therapy. Clinical studies provide access to the latest combinations.

**===== run_3 =====**

**Tumor board decision:**
Salvage chemotherapy (e.g. R-DHAP) -> for CR/PR allogeneic stem cell transplantation; If a suitable donor is missing, immediate participation in a CD19 bispecific antibody study (e.g. glofitamab/epcoritamab) or 2nd CAR T approach.

**Rationale:**
The patient has transformed DLBCL, has already received R-CHOP, Pola-R-Benda and CAR-T (lisocabtagene) and shows a progressive CD19-positive relapse. After CAR-T failure, alloSCT is the only potentially curative option in transplant-eligible patients. Without a donor, the next evidence-based therapy is participation in a CD19 bispecific antibody study or a second CAR-T trial.

## ============================================================ gpt-oss-120b (plain) ============================================================

**===== run_1 =====**

**Tumor board decision:**
After progression after CAR-T therapy, an allogeneic stem cell transplant (allo-HSCT) should be the next therapeutic approach. If transplant eligibility is not possible, the patient will be included in a clinical study protocol (e.g. bispecific antibodies or PD-1 blockade) after appropriate bridging chemotherapy.

**Rationale:**
The patient has already received R-CHOP, polatuzumab-bendamustine, and CAR-T and presents with recurrent progressive DLBCL. Further standard chemotherapy has little prospect; allo-HSCT offers a potentially curative option if the patient is transplant eligible. If unsuitable, clinical studies are the sensible alternative.

**===== run_2 =====**

**Tumor board decision:**
Recommendation: If the disease progresses after CAR-T therapy, allogeneic hematopoietic stem cell transfer (R-IC) should be attempted if the patient is suitable for transplantation. Consider a short bridging regimen (e.g., tafasitamab+lenalidomide or selinexor) for disease control prior to transplant. If allotransplantation is not possible, participate in a clinical trial with bispecific antibody T cell engagers (e.g. Ep-C) or loncastuximab tesirine.

**Rationale:**
The patient has already received R-CHOP, Pola-Benda and CAR-T and is now showing PD. After CAR-T failure, allogeneic SCT is the only potentially curative option in suitable patients. If there is no suitability, modern targeted therapies or studies are the next sensible options.

**===== run_3 =====**

**Tumor board decision:**
After progressive disease despite R-CHOP, polatuzumab-bendamustine-rituximab and CAR-T (lisocabtagene), an intensive salvage regimen (e.g. R-ICE or R-DHAP) should be initiated to achieve remission and subsequent allogeneic stem cell transplantation should be considered. If the patient is not transplant eligible, participation in a clinical trial with novel CD20 bispecific antibodies or other targeted therapies is strongly recommended.

**Rationale:**
The patient has already received three lines of therapy including CAR-T and now has progressive DLBCL. Further chemotherapy can serve as a bridge therapy to allogeneic HSCT, which is the only curative option for refractory DLBCL. If transplantation is not possible, access to a study program is the best alternative.

## ============================================================ qwen3-32b (agent) ============================================================

**===== run_1 =====**

**Tumor board decision:**
Recommendation: Glofitamab + GemOx as first salvage therapy after CAR-T-PD. Alternatively, epcoritamab or ViPOR combination therapy. Clinical studies (bispecific antibodies, CAR-T alternatives) are prioritized.

**Rationale:**
Glofitamab+GemOx has demonstrated efficacy in post-CAR-T PD in phase 3 trials. Non-GCB subtype benefits from ViPOR. Epcoritamab as a bispecific approach. Clinical trials offer the latest options for refractory DLBCL.

**===== run_2 =====**

**Tumor board decision:**
Recommendation: Tafasitamab/lenalidomide, VIPOR (off-label) or clinical trials with BiTE/2. CAR-T. Re-biopsy for CD19/CD20 validation and lymphoma conference for evaluation of alloSCT/2. CAR-T.

**Rationale:**
After CAR-T early relapse, CD19/CD20-targeted therapies (tafasitamab/lenalidomide, VIPOR) and clinical trials with BiTE/2 are available. CAR-T first options. Re-biopsy to confirm expression and conference on alloSCT/2. CAR-T eligibility required.

**===== run_3 =====**

**Tumor board decision:**
Salvage therapy with loncastuximab tesirine (CD19-targeting ADC) or glofitamab (CD20/CD3-bispecific) as first choice is recommended. Clinical studies with 2nd CAR-T (e.g. Ciltacabtagene autoleucel) or BiTE (e.g. mosunetuzumab) must be urgently examined. If CD19 expression and CAR-T suitability, alternatively consider a second CAR-T therapy.

**Rationale:**
After PD under lisocabtagene and previous R-CHOP/R-Pola/mini-Benda, salvage therapy with CD19/CD20-targeting agents is indicated. Loncastuximab and glofitamab showed good efficacy in third-line DLBCL. Clinical studies with innovative concepts (e.g. BCMA-targeting CAR-T, combinations with BTK inhibitors) are prioritized. Re-biopsy to confirm CD19 expression is recommended.

## ============================================================ qwen3-32b (plain) ============================================================

**===== run_1 =====**

**Tumor board decision:**
Recommendation: Participation in clinical trial with targeted therapy (e.g. BCL-2 inhibitor or CD19/CD20 dual targeting) or salvage regimens such as GEMOX/R-DHAP. If there is no option: palliative therapy.

**Rationale:**
After CAR-T progress and previous therapeutic approaches (R-CHOP, R-Pola/mini-Benda), a clinical trial with innovative approaches (e.g. BCL-2 inhibitors in MYC-negative phenotype) is the next step. If participants do not participate in the study: salvage regimen followed by a possible second CAR-T infusion.

**===== run_2 =====**

**Tumor board decision:**
Recommendation: Participation in a clinical trial with novel therapeutic approaches (e.g. BCL-2 inhibitors, BTK inhibitors or T-cell-based therapies). If ineligible: salvage regimen (e.g. GEMOX) with subsequent possible second CAR T cell therapy.

**Rationale:**
Novel targets are required after CAR-T progression. Non-GCB subtype and high Ki-67 indicate aggressive biology. No previous resistance to CD19 targeting. Clinical studies offer the latest therapeutic approaches. Salvage regime as a bridge to further cell therapies.

**===== run_3 =====**

**Tumor board decision:**

Recommendation: Participation in a clinical study with a novel target (e.g. BCL-2 inhibitors, BTK inhibitors or CD19/CD20 dual targeting). If unavailable: zanubrutinib (BTK inhibitor) or tafasitamab + lenalidomide.

**Rationale:**
After CAR-T recurrence and previous lines (R-CHOP, R-Pola/mini-Benda), standard options are exhausted. Non-GCB subtype with high Ki-67 suggests aggressive biology. Clinical studies offer the latest approaches. Alternatively, BTK inhibitors or CD19/CD20 targeting if toxicity is tolerable.

## ============================================================ qwen3-next-80b (agent) ============================================================

**===== run_1 =====**

**Tumor board decision:**
Re-biopsy to check CD19/CD20 expression, then BiTE therapy (epcoritamab or glofitamab) as the next step. Review clinical trials.

**Rationale:**
After CAR-T progression, BiTE is (1st choice) according to the flowchart. Re-biopsy required to confirm CD19/CD20 expression (CAR-T can cause CD19 loss). Epcoritamab (CD20/CD3) or glofitamab (CD20/CD3) are approved for RR-DLBCL after >=2 lines. aHT is not a contraindication, CRS risk must be monitored. Actively review clinical studies (e.g. new BiTEs/ADCs).

**===== run_2 =====**

**Tumor board decision:**
BiTE therapy (e.g. glofitamab or epcoritamab) after re-biopsy to confirm CD20 expression. No further CD19-targeted therapies without prior expression testing. Review clinical trials.

**Rationale:**
After CAR-T progression, BiTE is 1st choice according to the flowchart. Re-biopsy required for CD20 testing (Glofitamab is CD20/CD3-BiTE). Alternatives such as alloSCT or 2. CAR-T only after BiTE failure or in individual cases. aHT allows BiTE therapy.

**===== run_3 =====**

**Tumor board decision:**
Re-biopsy for CD19/CD20 expression. If CD20 positive: start BiTE therapy (e.g. glofitamab). Review clinical trials.

**Rationale:**
After CAR-T progression, BiTE (CD20-targeted) is the first choice according to the flowchart. Re-biopsy necessary for expression testing. CD20 positivity is a prerequisite for BiTE. Alternatively, if CD19 is positive, other CD19-targeted options or alloSCT in individual cases.

## ============================================================ qwen3-next-80b (plain) ============================================================

**===== run_1 =====**

**Tumor board decision:**
Recommend therapy with CD20/CD3 bispecific antibodies (e.g. glofitamab or epcoritamab) or participation in a clinical trial.

**Rationale:**
After CAR-T progression, bispecific antibodies are standard options. Glofitamab/Epcoritamab show high response rates in R/R DLBCL after CAR-T. Hypertension not a contraindication. Clinical trials offer alternative options.

**===== run_2 =====**

**Tumor board decision:**
Start therapy with CD20/CD3 bispecific antibodies (e.g. glofitamab or epcoritamab) as the next therapy step.

**Rationale:**
After CAR-T progression, bispecific antibodies (glofitamab/epcoritamab) are standard therapeutic options with proven efficacy in this indication. Hypertension is treated, no other contraindications. Alternatives such as loncastuximab or clinical trials are possible, but bispecific antibodies have

stronger evidence.

**===== run_3 =====**

**Tumor board decision:**
Prioritize clinical trial; alternatively, glofitamab initiation

**Rationale:**
After CAR-T progression, clinical trials are the first choice. Glofitamab is approved for R/R DLBCL after >=2 lines of therapy (including CAR-T). Alternatives: Epcoritamab or Loncastuximab tesirine. aHT is not a contraindication.

# ly_case_8__ALL

###############################
#### CASE: 8
###############################

## ============================================================ gpt-5-mini (agent) ============================================================

**===== run_1 =====**

**Tumor board decision:**
Before deciding on therapy: another tissue biopsy to rule out histological transformation. If transformation is missing: targeted therapy with tazemetostat (EZH2 inhibitor) 800 mg p.o. twice daily (evidence: phase II; approval/studies for EZH2-mut. R/R FL). Parallel testing of bispecific T cell engagers/clin. Studies and Allo-SCT if suitable.

**Rationale:**
EZH2 p.Y646H is classified as oncogenic (+10; Horak et al. 2022). Phase II data (PMID:34159682) show high response rate in EZH2 mutant R/R FL; Matching analyzes (PMID:35157216) suggest a more favorable safety profile compared to PI3K inhibitors. Early progression after CAR-T justifies biopsy to rule out transformation before conversion.

**===== run_2 =====**

**Tumor board decision:**
Before deciding on therapy: another tissue biopsy to rule out histological transformation. Without transformation: targeted therapy with tazemetostat 800 mg BID (EZH2 mutated; phase II/regulatory evidence). Alternatively, study inclusion (bispecific T cell engagers) or allo-SCT if suitable.

**Rationale:**
EZH2 p.Y646H is classified as oncogenic (Horak +10; hotspot, functionally confirmed). Phase II data show high response rate in R/R FL (ORR ~76%, PMID:34159682); Matching analyzes suggest better safety record vs PI3K inhibitors (PMID:35157216). Biopsy recommended due to early progression after CAR-T.

**===== run_3 =====**

**Tumor board decision:**
Before definitive therapy: repeat tissue biopsy to exclude histological transformation. In the absence of transformation and confirmed EZH2 p.Y646H mutation, preferred therapy: Tazemetostat 800 mg p.o. twice daily (Phase II evidence). Parallel: Recommend inclusion in a study with bispecific T cell engagers; If suitable, consider Allo-SCT.

**Rationale:**
EZH2 p.Y646H is classified as an oncogenic (Horak et al. +10). Phase II study in EZH2-mutated R/R FL showed ORR ~76.5% with tazemetostat (PMID:34159682). Indirect comparison shows more favorable safety profile vs PI3K inhibitors (PMID:35157216). Rebiopsy due to early progression after CAR-T to clarify therapy planning.

## ============================================================ gpt-5-mini (plain) ============================================================

**===== run_1 =====**

**Tumor board decision:**
Recommendation: Priority should be given to inclusion in a clinical trial or rapid initiation of CD20xCD3 bispecific therapy (e.g. mosunetuzumab or glofitamab). If a chemosensitive remission is achieved and good performance status: Consider allogeneic stem cell transplantation as a curative option.

**Rationale:**
Patient with highly recurrent FL (FLIPI 4) progressed after R-bendamustine, R-CHOP, repeated anti-CD20 and tisagenlecleucel. Bispecifics show effectiveness after CAR-T failure; clinical trial preferred. Consider allo-SCT if there is appropriate remission and good fitness.

**===== run_2 =====**

**Tumor board decision:**

1) Immediate rebiopsy and PET-CT to confirm/exclude histological transformation plus molecular testing (EZH2). 2) For transformation: salvage chemotherapy according to the DLBCL regimen and if allogeneic SCT responds (fit patients). 3) If non-transformed: primarily study/bispecific CD20xCD3 antibody (e.g. mosunetuzumab/epcoritamab). If study not possible: EZH2-mut -> Tazemetostat; EZH2-wt -> copanlisib or rituximab+lenalidomide.

**Rationale:**
Patient with multiple refractory FL including CD19 CAR T failure. First clarify transf.r/o and molecular markers, as the treatment decision depends on them. After CAR-T, bispecifics are currently the best option; for EZH2 mutation specific oral option (tazemetostat). Allo-SCT only after salvage response and with good performance status.

**===== run_3 =====**

**Tumor board decision:**
Recommendation: Primary therapy with a CD20xCD3 bispecific antibody (e.g. mosunetuzumab or glofitamab) - ideally as part of a study/accessible program. If not available: study inclusion preferred; alternatively lenalidomide + anti-CD20 or copanlisib. Allo-SCT only in young, fit patients after response to salvage.

**Rationale:**
Patient with high-risk, multiple pretreated FL (R-B, R-CHOP, anti-CD20 maintenance, zanubrutinib bridging) and early progression after tisagenlecleucel. CD20xCD3 bisspecific Abs show activity after CAR-T failure; Studies/new classes of action given priority. Allo-SCT because of TRM only if remission can be achieved and good fitness.

## ============================================================ gpt-5-nano (agent) ============================================================

**===== run_1 =====**

**Tumor board decision:**
EZH2 p.Y646H-positive FL after CAR-T progression: tazemetostat as preferred targeted monotherapy; Phase II (Izutsu 2021) shows high ORR in EZH2 mutant FL; indirect comparison 2022 supports better safety profile vs PI3K inhibitors. Prefer clinical trial; Allogeneic transplantation only if the condition/curative goal is suitable.

**Rationale:**
Horak 2022 classifies EZH2 p.Y646H as oncogenic (+10). Hotspot, absence in gnomAD, functional evidence proves relevance. PubMed shows efficacy of tazemetostat in EZH2-mutant B-NHL/FL (Phase II, Japan) and comparatively favorable safety compared to PI3K inhibitors. This results in clear, targeted therapy recommendations; check study options; Consider transplant depending on condition.

**===== run_2 =====**

**Tumor board decision:**
EZH2 p.Y646H is oncogenic. Recommendation: Tazemetostat (EZH2 inhibitor) 800 mg PO BID as targeted therapy for EZH2-mutated FL after CAR-T progression. Evidence Level II (Phase II FL EZH2 mutant; ORR ~76%); supporting indirect comparison vs PI3K inhibitors. Consideration of clinical trials/allogeneic transplant if status is appropriate.

**Rationale:**
Horak: EZH2 p.Y646H Oncogenic (hotspot; gnomAD absence; functionally confirmed; REVEL 0.91). FISH findings unproven. PubMed: Phase II study in EZH2 mutant FL with tazemetostat; ORR high; Proudman 2022 indirect comparison in favor of tazemetostat over PI3K inhibitors. This results in: targeted therapy with tazemetostat; If necessary, clinical trial/allo-transplant depending on status.

**===== run_3 =====**

**Tumor board decision:**
Recommend tazemetostat (EZH2 inhibitor) 800 mg PO BID as targeted monotherapy after CAR-T progression in EZH2 p.Y646H. Phase II data shows high ORR; Safety profile more favorable than PI3K inhibitors. Review clinical trials; Depending on the condition, consider other options (BTK/PI3K/Lenalidomide).

**Rationale:**
EZH2 p.Y646H satisfies Horak et al. 2022 as Oncogenic (+10). Public evidence (Phase-II EZH2-mutant FL) shows activity of tazemetostat; indirect comparison suggests a better safety profile compared to PI3K inhibitors. Therefore, clear recommendation for tazemetostat after CAR-T progression; Check study options.

## ============================================================ gpt-5-nano (plain) ============================================================

**===== run_1 =====**

**Tumor board decision:**
Allogeneic transplantation (allo-SCT) recommended if transplantable. Bridge with lenalidomide + rituximab (R2) or BTK inhibitor ibrutinib to remission, then allo-SCT. If transplant not possible, continue R2 or participate in clinical trial.

**Rationale:**
FL with FLIPI 4, high risk; CAR-T progression after previous CR. Potentially curative with allo-SCT, provided remission/control is successful. Bridge needed to achieve transplant status; Prefer clinical trials if transplant is not feasible.

**===== run_2 =====**

**Tumor board decision:**
Allogeneic transplantation (allo-SCT) in remission after CAR-T relapse, if fitness exists; alternatively clinical study; if not possible, R2 (lenalidomide + rituximab) or BTK/PI3K inhibitors as a long-term option.

**Rationale:**
CAR-T relapse in FL with high FLIPI; allo-SCT offers a curative opportunity. Prefer primary clinical trial; in case of obstacles to transplantation or unavailability: R2 or PI3K inhibitors as an established long-term option.

**===== run_3 =====**

**Tumor board decision:**
Lenalidomide + rituximab (R2) as the next therapeutic step in case of continued progression after CAR-T therapy.

**Rationale:**
After CAR-T-PD, a change in therapy is necessary. R2 uses a different mechanism of action (IMiD + anti-CD20) and serves as an established FL salvage with often good tolerability; If necessary, clinical trial or allogeneic transplant if suitable.

## ============================================================ gpt-oss-20b (agent) ============================================================

**===== run_1 =====**

**Tumor board decision:**
Tazemetostat 800mg BID recommended as 3rd/4th Line salvage in relapsing/recurrent EZH2 mutant FL after tisagenlecleucel. M&V studies (PMID35157216,34159682) show ORR~76% (Phase II).

**Rationale:**
EZH2Y646H is Oncogenic (10 points). Tazemetostat has a targeted effect against EZH2 mutants; Studies provide supportive ORR. Alternatives such as PI3K inhibitors have a higher risk profile. No further therapy-relevant FISH changes.

**===== run_2 =====**

**Tumor board decision:**
Tazemetostat 800mg BID (28-day cycle) as salvage therapy for progressive, bone marrow-infiltrated FL patients with EZH2 p.Y646H (oncogenic). If the answer is insufficient, consider 'allo-SCT' or a second CAR-T approach.

**Rationale:**
EZH2 p.Y646H received 10 points (hotspot, functionally confirmed) - Oncogenic. Phase II trials (PMID35157216/34159682) show ORR~76% in R/RFL, fewer safety risks than PI3K inhibitors. Therefore, confirmation of tazemetostat is required; further progress is aimed at allo-SCT or second CAR-T.

**===== run_3 =====**

**Tumor board decision:**
Recommendation: Tazemetostat (800 mgbid) as monotherapeutic salvage therapy for EZH2p.Y646H-positive, bone marrow-infiltrated FL after tisagenlecleucel progression (Phase II Level B, evidence-based).

**Rationale:**

Oncogenic class, 10 points; Studies (PMID35157216 and 34159682) show ORR~76% in mutant FL, better safety than PI3K inhibitors; no FISH abnormalities, therefore targeted therapy indicated.

## ========================================================= gpt-oss-20b (plain) =========================================================

**===== run_1 =====**

**Tumor board decision:**
After progression after CAR-T (tisagenlecleucel), salvage therapy with obinutuzumab+venetoclax (G-venetoclax) is recommended. If available, the patient should be enrolled in a clinical trial of a T cell-engaged antibody (e.g. mosunetuzumab). If there is no accessibility, second CAR-T therapy is only worth considering in studies.

**Rationale:**
The patient has already received R-BB, R-CHOP, rituximab maintenance, bridging therapy (obinutuzumab+zanubrutinib) and CAR-T. G-Venetoclax has demonstrated documented activity in FL after multiple progressions and is well tolerated. Up to 30% of patients achieve CR/PR. Clinical trials with bispecific T-cell-engaged antibodies provide additional options when G-venetoclax is not available. Second CAR-T therapy is only useful in studies as the evidence is limited.

**===== run_2 =====**

**Tumor board decision:**
Start salvage therapy with venetoclax + obinutuzumab (G-venetoclax) as first option after CAR-T failure. Consider enrollment in a clinical trial with ibrutinib+venetoclax or a second CAR-T (liso-cél) if eligible.

**Rationale:**
Venetoclax+obinutuzumab shows ~30-40% CR in FL relapsed after CAR-T, is approved, well tolerated, and can be initiated immediately. A second CAR-T is an alternative but requires time and eligibility assessment.

**===== run_3 =====**

**Tumor board decision:**
Forwarding to second CAR-T therapy (LisocabtageneMaraleucel) after bridging with Obinutuzumab+Venetoclax. If there is no response or intolerance: Allogeneic stem cell transplant (allo-SCT) in a specialized clinic.

**Rationale:**
The patient showed PD after tisagenlecleucel, despite PET-CR. For FLIPI5 and bulk disease, renewed CAR-T therapy with lisocabtagene is the preferred option, as studies show positive response rates in CAR-T failure. Allo-SCT remains as a last resort for refractory FL.

## ========================================================= gpt-oss-120b (agent) =========================================================

**===== run_1 =====**

**Tumor board decision:**
EZH2-mutated R/R FL - Therapy recommendation: tazemetostat 800 mgbid as monotherapy (FDA/EMA approved) - Level II evidence (Phase II and Matching-Adjusted Indirect Comparison). If available, a suitable EZH2 inhibitor study (e.g. combination with anti-CD20 or new EZH2 inhibitor) should be considered.

**Rationale:**
EZH2 p.Y646H achieved according to Horak et al. 2022 10 points -> Oncogenic. Two PubMed studies (PMID 35157216, 34159682) show ORR approximately 75% and good tolerability of tazemetostat in EZH2-mutant FL, superior to PI3K inhibitors. No other FISH changes relevant to therapy. Therefore, targeted EZH2 inhibition is recommended.

**===== run_2 =====**

**Tumor board decision:**
Start tazemetostat (800 mgb.i.d.) as EZH2 inhibitory therapy for EZH2p.Y646H mutated, relapsed/refractory FL after CAR-T. If there is no response, consider allogeneic SCT or PI3K inhibitors (idelalisib, copanlisib, etc.); If necessary, inclusion in EZH2 inhibitor studies.

**Rationale:**

EZH2p.Y646H is classified with +10 points (Oncogenic). Literature (PMID35157216,34159682) shows ORR~76% and better safety profiles compared to PI3K inhibitors; Tazemetostat is FDA/EMA approved for EZH2 mutant R/R FL. No further treatable FISH aberrations.

**===== run_3 =====**

**Tumor board decision:**
Recommendation: Tazemetostat (EZH2 inhibitor) as targeted therapy for recurrent FL with EZH2 p.Y646H; alternatively, inclusion in an EZH2 inhibitor study; Allogeneic SCT only if there is no response rate.

**Rationale:**
EZH2 p.Y646H is according to Horak et al. Classified as oncogenic in 2022 with +10 points. PubMed studies (PMID35157216,34159682) show high ORR (~35% CR, 41% PR) and better safety profile compared to PI3K inhibitors in phase II or indirect comparisons. Therefore, tazemetostat is recommended as the next targeted option; If therapy fails or is inaccessible, allotransplantation or study participation makes sense.

## ============================================================ gpt-oss-120b (plain) ============================================================

**===== run_1 =====**

**Tumor board decision:**
In the case of refractory FL after multiple chemo-immunotherapy, R-CHOP, BTK inhibitor and CAR-T, an allogeneic stem cell transplant (allo-HSCT) is recommended as the next step, provided the patient is suitable for transplantation (matched donor, sufficient organ function). If transplantation is not possible, therapy should be offered as part of a clinical trial with a CD20xCD3 bispecific (e.g. mosunetuzumab) or alternatively a combination of lenalidomide+rituximab.

**Rationale:**
The patient has high-risk FL (FLIPI5, BM infiltration, bulk) and is resistant to multiple treatments, including CAR-T, which greatly worsens the prognosis. Allo-HSCT offers the only potentially curative option. If transplantation is not possible, modern bispecific antibodies or R-lenalidomide are available as evidence-based, well-tolerated options.

**===== run_2 =====**

**Tumor board decision:**
Recommendation: Consider allogeneic stem cell transplantation (allo-SCT) for refractory FL after CAR-T therapy; If you are not eligible for a transplant, take part in a study with a CD20-CD3 bispecific or PI3K inhibitor.

**Rationale:**
Patient has multiple progressions despite R-CHOP, rituximab maintenance and CAR-T; FLIPI5 and bone marrow infiltration show high risk. Allo-SCT offers potential cure; other options are only palliative.

**===== run_3 =====**

**Tumor board decision:**
Recommendation: Allogeneic hematopoietic stem cell transplantation (matched sibling/unrelated) as a curative option. If donor availability is not available, immediate therapy with a CD20-CD3 bispecific (e.g. mosunetuzumab) as part of a clinical study protocol.

**Rationale:**
FL with FLIPI5, multiple therapy and CAR-T failures, bone marrow infiltration and bulk disease. Standard options (R-CHOP, R-B, rituximab maintenance, BTK inhibitor, CAR-T) have been exhausted. Allogeneic TBI offers the only potentially curative chance; Bispecifics are approved, effective options for R/R-FL after >=2 prior treatments.

## ============================================================ qwen3-32b (agent) ============================================================

**===== run_1 =====**

**Tumor board decision:**
Recommendation of EZH2 inhibition with tazemetostat (evidence level 2B, phase II studies) as targeted therapy for EZH2-p.Y646H-mutant relapsed follicular lymphoma after CAR-T therapy failure.

**Rationale:**
The EZH2-p.Y646H variant is classified as oncogenic (10 points according to Horak et al.). PubMed evidence (PMID:35157216, 34159682) confirms the efficacy of tazemetostat in EZH2-mutated NHL with an objective response rate of 76.5% (Phase II). The safety is■■ PI3K inhibitors.

**===== run_2 =====**

**Tumor board decision:**
Recommendation of the EZH2 inhibitor Tazemetostat (evidence level 2B, phase II study) as targeted therapy for EZH2-p.Y646H-mutant relapsed follicular lymphoma after CAR-T therapy failure.

**Rationale:**
EZH2-p.Y646H is classified as oncogenic (10 points according to Horak). PubMed evidence (PMID:35157216, 34159682) confirms efficacy and safety of tazemetostat in EZH2-mutated R/R follicular lymphomas, particularly after prior CAR-T therapy.

**===== run_3 =====**

**Tumor board decision:**
Recommendation of tazemetostat therapy as targeted treatment for EZH2 p.Y646H mutant tumor. Evidence level: II (Phase II studies).

**Rationale:**
The EZH2 p.Y646H variant is classified as oncogenic (10 points according to Horak). PubMed evidence (PMID:35157216, 34159682) confirms the efficacy of tazemetostat in EZH2-mutant FL with objective remission rate (76.5%) and favorable safety profile. CAR-T failure and FLIPI-5 risk warrant targeted therapy.

## ============================================================ qwen3-32b (plain) ============================================================

**===== run_1 =====**

**Tumor board decision:**
Recommendation: Participation in a clinical trial with novel targeted therapy (e.g. BCL-2 inhibitor venetoclax + rituximab) or PI3K inhibitor (copanlisib/idelalisib). CAR-T has already been carried out, no further cell therapy without a specific indication.

**Rationale:**
High-grade progression after CAR-T and R-CHOP despite bridging therapy. FLIPI-5 risk and recurrent disease require novel approaches. Repeat therapy classes already received (CD20 inhibitors, BTK inhibitors) are not indicated. Clinical studies offer the latest therapeutic approaches.

**===== run_2 =====**

**Tumor board decision:**
Recommendation: Participation in clinical trials with novel agents (e.g. BCL-2 inhibitors, CD19/CD20 dual-target therapy) or combination therapy with venetoclax + obinutuzumab. If unavailable: bridging with ibrutinib + venetoclax.

**Rationale:**
Following CAR-T progression and resistance to BTK inhibitors (zanubrutinib), a new mechanism of action is necessary. Venetoclax combined with obinutuzumab shows potential in high-risk cases. Clinical trials offer innovative options for refractory FL.

**===== run_3 =====**

**Tumor board decision:**
Recommendation: Participation in a clinical study with a BCL-2 inhibitor (venetoclax) or alternatively combination therapy with a BTK inhibitor (acalabrutinib) and anti-CD20 antibodies (e.g. obinutuzumab).

**Rationale:**
After CAR-T progression and prior obinutuzumab/zanubrutinib bridging therapy, new targets are required. BCL-2 inhibitors show efficacy in relapsed FLs. Clinical trials provide access to innovative therapies such as venetoclax or alternative combinations.

## =========================================================== qwen3-next-80b (agent) ===========================================================

===== run_1 =====

**Tumor board decision:**
Tazemetostat (800 mg twice daily) recommended as targeted therapy for EZH2 mutant follicular lymphoma after CAR-T progression. Evidence: Phase II trials (ORR 76.5%), FDA approval for EZH2-mutant FL after >=2 prior therapies.

**Rationale:**
EZH2 p.Y646H is oncogenic (10 points according to Horak 2022). Pubmed evidence shows high response rate with tazemetostat. Clinical studies confirm effectiveness in relapsed FL. No alternatives with higher evidence.

===== run_2 =====

**Tumor board decision:**
Recommend tazemetostat 800 mg twice daily as a therapeutic option. Level of evidence: Phase II study (ORR 76.5%) and clinical approval for EZH2-mutant relapsed/refractory follicular lymphoma after at least two prior therapies.

**Rationale:**
EZH2 p.Y646H is according to Horak et al. Classified as oncogenic in 2022 (10 points). Pubmed evidence shows high response rate (76.5%) and better safety profile of tazemetostat vs. PI3K inhibitors in EZH2-mutant FL after >=2 therapies. Clinical approval for this indication confirmed.

===== run_3 =====

**Tumor board decision:**
Tazemetostat (800 mg BID) recommended as targeted therapy (evidence level 2, phase II studies PMID:34159682, 35157216).

**Rationale:**
EZH2 p.Y646H is oncogenic (10 points according to Horak et al. 2022). Phase II studies show ORR 76.5% and superior safety vs PI3K inhibitors. Treatment option for relapsed/refractory follicular lymphoma after CAR-T progression.

## ============================================================ qwen3-next-80b (plain) ============================================================

===== run_1 =====

**Tumor board decision:**
Recommendation: initiation of bispecific antibody therapy (e.g. epcoritamab) as the next therapeutic step.

**Rationale:**
After CAR-T failure in FL, bispecific antibody therapy (e.g. epcoritamab) is standard-compliant. Patient meets criteria (more than 2 previous lines of therapy), with high efficacy in R/R-FL and good tolerability. Alternatives such as clinical studies should also be examined.

===== run_2 =====

**Tumor board decision:**
Initiate bispecific antibody therapy (e.g. mosunetuzumab or epcoritamab).

**Rationale:**
After CAR-T failure and PD, bispecific antibody therapy is the best option. Mosunetuzumab and epcoritamab are approved for FL and demonstrate high response rates in multiply relapsed/refractory FL. PI3K inhibitors less preferred due to toxicity.

===== run_3 =====

**Tumor board decision:**
Prefer clinical trial; alternatively epcoritamab or glofitamab

**Rationale:**
After CAR-T-PD, clinical trials are the first choice. Bispecific antibodies (epcoritamab/glofitamab) show response in refractory FL in studies. No other standardized options with sufficient evidence.

# ly_case_9__ALL

###############################
#### CASE: 9

###############################

## ================================================================ gpt-5-mini (agent) ================================================================

**===== run_1 =====**

**Tumor board decision:**
Rebiopsy (immunophenotype/gene expression), PET-CT. Therapy: polatuzumab-vedotin-based regimen recommended - participation in the mosunetuzumab+polatuzumab study (phase1b/2, PMID:38072960) is preferred; alternatively Pola+Bendamustine+Rituximab (phase2, PMID:33942442) or Loncastuximab/bispecific antibodies in studies. Evaluation for allo-HSCT in chemosensitive remission.

**Rationale:**
CD79B p.Y197C is classified as an oncogenic (10 P.; hotspot Y197, OncoKB, functional and computational evidence). CD79B is targeted by polatuzumab vedotin; clinical data demonstrate efficacy in R/R DLBCL (PMID:38072960, 33942442). After axi-cel failure, pola-based regimens, bispecific antibodies and study participation are prioritized; Evaluate allo-HSCT when remission is achieved.

**===== run_2 =====**

**Tumor board decision:**
CD79B p.Y197C is classified as an oncogenic. Preferred treatment: anti-CD79B-ADC (polatuzumab vedotin) - if possible in a clinical trial (e.g. mosunetuzumab + polatuzumab, phase 1b/2; PMID:38072960). Alternatively, participation in studies on anti-CD79B ADCs (e.g. DCDS0780A). Recommended: rebiopsy with immunophenotyping and PET-CT before therapy; Evaluation for Allo-HSCT upon response.

**Rationale:**
Horak score +10 (hotspot Y197C, OncoKB function, computational), therefore oncogenic. Publications show clinical activity of CD79B targeting in R/R DLBCL (mosunetuzumab+polatuzumab Phase1b/2, PMID:38072960; anti-CD79B ADC Phase1, PMID:34980599). Therefore, prioritization of CD79B-targeted ADCs/trials and allo-HSCT evaluation.

**===== run_3 =====**

**Tumor board decision:**
Rebiopsy including immunophenotype and gene expression analysis as well as PET-CT. CD79B p.Y197C (Oncogenic): Priority to study participation in anti-CD79B ADCs +/- bispecific antibodies (e.g. polatuzumab vedotin + mosunetuzumab arm) or other anti-CD79B trials (DCDS0780A). If study not available: polatuzumab-based therapy or loncastuximab; in CR evaluation for allo-HSCT.

**Rationale:**
Horak et al. Rating: CD79B p.Y197C +10 points -> Oncogenic. Evidence: Phase 1b/2 shows efficacy of polatuzumab vedotin + mosunetuzumab (PMID:38072960); anti-CD79B ADCs show activity (PMID:34980599); CD79B mutations influence prognosis (PMID:36182550). Therefore, trial prioritization and targeted anti-CD79B therapy are recommended.

## ================================================================ gpt-5-mini (plain) ================================================================

**===== run_1 =====**

**Tumor board decision:**
Priority: Inclusion in a clinical study with a CD20xCD3 bispecific (e.g. epcoritamab/glofitamab/mosunetuzumab) due to progression after CD19 CAR T and multiple previous therapies. If study-free and tumor CD19 positive: consider tafasitamab+lenalidomide. Allo-SCT only if remission can be achieved and good transplant fitness.

**Rationale:**
The patient has refractory DLBCL after R-CHOP, Auto-HSCT, Pola-BR and CD19-CAR-T (Progress 06/2024). Polatuzumab/bendamustine have already been used. CD19 antigen loss is possible after CAR-T; CD20xCD3 bispecifics show activity after CAR-T and are therefore the therapy of choice. Allo-SCT due to age/comorbidities only with validated remission.

**===== run_2 =====**

**Tumor board decision:**
Patient with progression after Axi-cel: Recommendation for priority inclusion in a clinical study with a CD20xCD3 bispecific (e.g. epcoritamab/glofitamab). If not available: use of an approved bispecific or alternatively loncastuximab-tesirine. Do not readminister polatuzumab/bendamustine.

**Rationale:**
Progression after CAR-T = highly refractory DLBCL. CD20xCD3 bispecifics have good response rates after CAR-T and better adequacy than conventional salvage chemo. Polatuzumab and bendamustine have already been used and should not be repeated; Prioritize clinical trials for access to new substances.

**===== run_3 =====**

**Tumor board decision:**
Recommendation: Systemic therapy with a CD20xCD3 bispecific antibody (e.g. epcoritamab or glofitamab), if possible as part of a clinical study. In the case of symptomatic localized tumor mass, palliative radiotherapy is added. Only consider Allo-SCT if there is a response and an interdisciplinary evaluation.

**Rationale:**
Multirelapsed DLBCL (previous lines: R-CHOP, Salvage+auto-HSCT, Pola-BR, CAR-T Axi-cel) with progression after CAR-T. CD20xCD3 bispecifics show activity after CAR-T failure; CD19 re-exposure after unsuccessful CAR-T less promising. Clinical trial preferred; RT for local symptom control.

## ========================================================== gpt-5-nano (agent) ==========================================================

**===== run_1 =====**

**Tumor board decision:**
Actionable CD79B p.Y197C. Primary options: CD79B-targeted therapies. Polatuzumab vedotin + mosunetuzumab (R/R LBCL; phase 1b/2) preferred. Alternative/Investigational: DCDS0780A Anti-CD79B ADC (Phase 1). Progression after CAR-T: clinical trials preferred.

**Rationale:**
Horak et al. 2022: CD79B p.Y197C Oncogenic (+10); FISH unremarkable. PubMed: CD79B-targeted therapies with activity (polatuzumab vedotin + mosunetuzumab; DCDS0780A) in phase 1/2; clinical study recommendation for progression after CAR-T.

**===== run_2 =====**

**Tumor board decision:**
CD79B p.Y197C is Likely Oncogenic. Recommendation: CD79B-targeted salvage therapies (polatuzumab vedotin + bendamustine + rituximab or mosunetuzumab + polatuzumab vedotin); preferably clinical studies. Allogeneic transplant only with robust salvage response and good fitness; Otherwise, continue the therapy strategy depending on the course.

**Rationale:**
Horak 2022: CD79B p.Y197C = Likely Oncogenic (6 points). PubMed evidence: CD79B-targeted therapies (Pola+BR; mosunetuzumab+PolaVedotin) show activity in R/R DLBCL, including CD79B mutations; Evidence Phase II/1b/2. FISH negative. AlloSCT only after salvage response; preferably clinical studies.

**===== run_3 =====**

**Tumor board decision:**
Oncogenic CD79B p.Y197C; Therapy recommendation: Participation in clinical studies with CD79B-targeted therapies; Specific options from publications: Mosunetuzumab + Polatuzumab Vedotin (Phase 1b/2) and DCDS0780A (CD79B-ADC) - Evidence Phase 1/2. If not available, consider tafasitamab+lenalidomide or loncastuximab tesirine.

**Rationale:**
Horak 2022 rates CD79B p.Y197C with 10 points (Oncogenic); FISH unremarkable. PubMed supports CD79B-driven therapies in R/R DLBCL (Phase 1b/2 mosunetuzumab + polatuzumab vedotin; CD79B-ADC DCDS0780A). Therefore, study implementation or CD79B-targeted therapy is preferred; Allo-SCT is of secondary importance after CAR-T progression.

## ============================================================ gpt-5-nano (plain) ============================================================

**===== run_1 =====**

**Tumor board decision:**
Post-CAR-T-PD DLBCL: clinical trial or treatment with loncastuximab tesirine or tafasitamab + lenalidomide; if there is adequate remission, consider allogeneic stem cell transplantation; integrate supportive measures.

**Rationale:**
PD after CAR-T; limited options. CD19-targeted therapies show activity; Allogeneic transplant only if there is sufficient remission/tolerability; Goal of remission/long-term control and quality of life.

**===== run_2 =====**

**Tumor board decision:**
Loncastuximab tesirine (CD19-ADC) as the next systemic therapeutic approach for progression after CAR-T therapy (DLBCL).

**Rationale:**
Post-CAR-T-PD is refractory DLBCL. Loncastuximab shows activity after CAR-T and multiple prior lines; acceptable side effect profile. If CD19 expression is reduced, alternatives: tafasitamab+lenalidomide or allogeneic transplant depending on performance status.

**===== run_3 =====**

**Tumor board decision:**
Recommendation: CAR-T progression after axi-cel - participation in clinical study protocol preferred. If not possible, systemic therapy with loncastuximab tesirine (ADCT-402) or tafasitamab plus lenalidomide; in the case of focal progression, local radiotherapy if necessary; Goal: disease control and quality of life.

**Rationale:**
After CAR-T-PD for high-risk DLBCL, established options are limited. Clinical trials provide access to new approaches; Available antibody conjugates (loncastuximab, tafasitamab+lenalidomide) show activity after CAR-T. RT only for symptomatic/focal progression for supportive therapy.

## ============================================================ gpt-oss-20b (agent) ============================================================

**===== run_1 =====**

**Tumor board decision:**
Polatuzumab vedotin in combination with bendamustine and rituximab (Pol-BR) recommended as rescue treatment; additionally consider participation in mosunetuzumab plus polatuzumab trials (Phase 1b/2).

**Rationale:**
The CD79Bp.Y197C mutation is oncogenic (+10 points). Polatuzumab-vedotin, a CD79B ADCs, shows significant CR rates in phase 2 trials (2021) and clinical activity in 2024 mosunetuzumab-polatuzumab combinations. Comb. with bendamustine/rituximab has evidence-based support; therefore, this option is listed as the relevant treatment option.

**===== run_2 =====**

**Tumor board decision:**
For CD79B p.Y197C (oncogenic, +10 points), we recommend polatuzumab vedotin in combination with bendamustine + rituximab (pola-BR) after unsuccessful CAR-T therapy. This regimen achieved an ORR >60% in phase 2 trials (N=120) and is approved for R/R DLBCL without an ASCT option.

**Rationale:**
Variant is clearly oncogenic and is directly targeted by polatuzumab (anti-CD79B). Phase-1b/2 (2024) and Phase-2 (2021) show high ORR/CRR. CD79B mutation signals worse prognosis, hence intensive approach. No relevant FISH change found.

**===== run_3 =====**

**Tumor board decision:**
Polatuzumab-vedotin 1.8mg/kg + bendamustine 90mg/m^2 + rituximab 375mg/m^2 q3w, 6cycles (PhaseII du2021) - targeted ADC against CD79B mutated. Alternatively, mosunetuzumab + polatuzumab (Phase1b/2 2024) remains options.

**Rationale:**
Variant CD79Bp.Y197C is oncogenic (+10 points). No FISH-abnormalities. Literature: 2024 Mosunetuzumab+Pola (phase1b/2) and 2021 Pola+BR (phaseII) show ORR/CRR >30% in CD79B-positive R/R-DLBCL. Consequently, the recommended Pola combination is evidence-based.

## ========================================================== gpt-oss-20b (plain) ==========================================================

**===== run_1 =====**

**Tumor board decision:**
Recommendation: Salvage therapy with R-GemOx (Rituximab+Gemcitabine+Oxaliplatin) as a bridge to possible allogeneic stem cell transplantation (if the patient is fit).

**Rationale:**
Patient has PD after CAR-T and multiple previous regimens (Pola-BR, R-DHAP, R-CHOP). R-GemOx shows a good response rate in refractory DLBCL, is well tolerated in HTN/DM and can produce remission for an allo-HSCT bridge. If there is no remission, clinical studies should be examined.

**===== run_2 =====**

**Tumor board decision:**
Start tafasitamab+lenalidomide as salvage therapy (or participate in a CAR-T alternative trial, if available).

**Rationale:**
Patient demonstrated PD after four relapses and CAR-T therapy. Tafasitamab+lenalidomide is the only approved combination for relapse/refractory DLBCL, well tolerated in HTN/DM and offers a new mechanism of action after pola/bendamustine regimens.

**===== run_3 =====**

**Tumor board decision:**
After CAR-T failure with PD in June 2024, we recommend an allogeneic stem cell transplant (allo-SCT) after bridging therapy with R-GemOx (R-Gemcitabine + Oxaliplatin + Rituximab).

**Rationale:**
CAR-T functional tolerance is exhausted after 2 relapses; allo-SCT offers the only potential curative option. R-GemOx reduces tumor burden, is well tolerated in HTN/DM and enables safe transplantation.

## ========================================================== gpt-oss-120b (agent) ===========================================================

**===== run_1 =====**

**Tumor board decision:**
For post-CAR-T failure and progressive DLBCL with CD79B-p.Y197C (oncogenic), we recommend CD79B-targeted therapy: 1) participation in the ongoing phase 1b/2 study mosunetuzumab+polatuzumab-vedotin (proven ORR); 2) alternatively polatuzumab-vedotin+bendamustine+rituximab (Phase 2, robust CR rates). If there is a response, check allo-SCT.

**Rationale:**
CD79B-p.Y197C receives +10 points (rare, hotspot, functional detection, computation) -> Oncogenic. PubMed data (PMID38072960, 33942442) support effective CD79b ADC combinations, particularly mosunetuzumab+polatuzumab (Phase1b/2) and polatuzumab+bendamustine+rituximab (Phase2). The mutation is prognostically unfavorable, so targeted CD79b therapy is recommended after CAR-T failure.

**===== run_2 =====**

**Tumor board decision:**
Due to the oncogenic classification of the CD79B mutation (p.Y197C), we recommend CD79B-targeted therapy. Priority is given to participation in a study with mosunetuzumab+polatuzumab vedotin (Phase1b/2, PD-LBCL) or, if not available, the use of polatuzumab vedotin-based regimens (e.g. Pola-BR). Allograft should only be considered after targeting options have been completed.

**Rationale:**
CD79B p.Y197C scored +10 points (hotspot, functional evidence, computation) -> Oncogenic. Publications (PMID:38072960, 34980599) show efficacy of anti-CD79B ADCs and combinations in

refractory DLBCL. The variant is therefore therapeutically relevant (evidence level II-III).

**===== run_3 =====**

**Tumor board decision:**
Recommendation: After proven oncogenic CD79B p.Y197C mutation and CAR-T failure, the patient should be included in the mosunetuzumab + polatuzumab vedotin (Phase 1b/2) study protocol. If the study is missing, polatuzumab-vedotin-based therapy (e.g. polatuzumab+R-CHP) is indicated.

**Rationale:**
Variant: CD79B p.Y197C - 10 points -> Oncogenic. Literature (PMID:38072960) demonstrates the effectiveness of mosunetuzumab+polatuzumab; PMID:34980599 shows anti-CD79B-ADC activity. Mutation correlates with poor prognosis (PMID:36182550), so a CD79B-targeted approach is recommended.

## ================================================================ gpt-oss-120b (plain) ================================================================

**===== run_1 =====**

**Tumor board decision:**
After progressive disease despite CAR-T (axi-cel), the next therapy should be part of a clinical trial, preferably with a CD20xCD3 bispecific antibody (e.g. glofitamab/epcoritamab). If there is no study option, therapy with loncastuximab vedotin or palliative care should be considered.

**Rationale:**
The patient has already received 3 lines including autologous HSCT and CAR-T; Further standard chemotherapy has low chances of success and the risk of toxicity is high due to age >60 years, HT and diabetes. Bispecific antibodies show promising remission rates after CAR-T failure and are therefore the most sensible option.

**===== run_2 =====**

**Tumor board decision:**
After progressive disease despite CAR-T (axi-cel), an allogeneic stem cell transplant (allo-HSCT) is considered as the next step, provided the age is <=70 years, ECOG <=2 and a suitable donor is available. If transplant eligibility is not given, we recommend therapy with a CD20-CD3 bispecific (e.g. glofitamab/epcoritamab) as part of a clinical study or compassionate use program.

**Rationale:**
The patient has already received three linear therapies including autologous HSCT and CAR-T; Progression after CAR-T only allows curative options (allo-HSCT) or new targeted immunotherapies. Allo-HSCT potentially offers long-term remission, while bispecifics are effective after CAR-T and can be used when transplantability is limited.

**===== run_3 =====**

**Tumor board decision:**
After progressive disease despite CAR-T (axi-cel), the next step should be to consider an allogeneic stem cell transplant with suitable fitness (ECOG<=2) and an available HLA-compatible donor. If this is not possible, participation in a clinical trial with a CD20-CD3 bispecific (e.g. epcoritamab) is recommended.

**Rationale:**
The patient has already received three recurrences, autologous HSCT and CAR-T; Conventional chemotherapy (R-CHOP, DHAP, Pola-BR) has been exhausted. Allogeneic HSCT offers a potentially curative option if performance status and comorbidities (HT, diabetes) permit. If transplantation is not possible, a bispecific is the evidence-based next therapy.

## ================================================================ qwen3-32b (agent) ================================================================

**===== run_1 =====**

**Tumor board decision:**
Recommendation of the anti-CD79B antibody drug conjugate (ADC) polatuzumab vedotin in combination with mosunetuzumab (bispecific antibody) based on phase 1b/2 evidence (PMID:38072960). Alternatively, DCDS0780A (Phase 1 study, PMID:34980599) for treatment resistance.

**Rationale:**

CD79B p.Y197C is classified as oncogenic (10 points: rare, hotspot, functional evidence). PubMed evidence confirms efficacy of anti-CD79B ADC and combination therapies in relapsed DLBCL. Mutation correlates with poor prognosis under standard therapy (PMID:36182550).

**===== run_2 =====**

**Tumor board decision:**
Recommendation of combination therapy with polatuzumab vedotin (anti-CD79B ADC) and mosunetuzumab (CD20xCD3 BsAb) based on phase 1b/2 evidence (PMID:38072960). Alternatively, study inclusion in DCDS0780A clinics (PMID:34980599).

**Rationale:**
CD79B p.Y197C is oncogenic (10 points according to Horak: hotspot + functional evidence). PubMed evidence shows efficacy of anti-CD79B ADCs in recurrent DLBCL. Combination therapy with mosunetuzumab meets primary endpoint in phase 1b/2 study.

**===== run_3 =====**

**Tumor board decision:**
Recommendation of combination therapy with polatuzumab vedotin (anti-CD79B ADC) and mosunetuzumab (CD20xCD3 BsAb) based on phase 1b/2 evidence (PMID:38072960).

**Rationale:**
CD79B p.Y197C is classified as an oncogenic (10 points). PubMed evidence shows efficacy of anti-CD79B ADCs (Polatuzumab, DCDS0780A) and their combination with BsAbs in recurrent DLBCL.

## ============================================================ qwen3-32b (plain) ============================================================

**===== run_1 =====**

**Tumor board decision:**
Recommendation: Participation in a clinical trial with novel targeted therapy (e.g. BCL-2 inhibitor or PI3K inhibitor) after pretreatment with CAR-T and Pola-BR/Pola-R. If a study is not available: therapy with zanubrutinib + venetoclax requires approval.

**Rationale:**
Multiple relapsed DLBCL following CAR-T resistance requires new targets. BCL-2/PI3K pathway are urgent. Study participation prioritized. If unavailable: combination therapy with proven targeted agents.

**===== run_2 =====**

**Tumor board decision:**
Recommendation: Therapy with mosunetuzumab (CD20/CD3 bispecific antibody) as the next step after CAR-T failure

**Rationale:**
After R-CHOP, Pola-BR, CAR-T (Axi-cel) and PD, the patient now has no further standardized options. Mosunetuzumab is approved after BTK inhibitor and CAR-T and addresses CD20 (already targeted by CAR-T), but offers new mode of action. Comorbidities (DM2, HTN) require toxic-friendly therapy.

**===== run_3 =====**

**Tumor board decision:**
Recommendation: Participation in a clinical study with novel therapeutic approaches (e.g. bifunctional CAR-T, BTK inhibitors) or salvage regimens (e.g. GEMOX) in patients not suitable for the study.

**Rationale:**
After CAR-T progression and multiple relapsed DLBCL, standard options are exhausted. Clinical studies offer new approaches. GEMOX as a salvage option takes pre-existing conditions and toxicity profile into account.

## ============================================================ qwen3-next-80b (agent) ============================================================

**===== run_1 =====**

**Tumor board decision:**

Recommendation: Combination therapy with mosunetuzumab and polatuzumab vedotin (PMID:38072960). Evidence level IIb. Alternatively: study participation in DCDS0780A (PMID:34980599).

**Rationale:**
CD79B p.Y197C is oncogenic (10 Horak points). Phase 1b/2 study shows high response rate in relapsed/refractory LBCL with CD79B mutations. Polatuzumab vedotin binds CD79B regardless of mutation. Mosunetuzumab activates T cells against CD20+ cells. Evidence: clinical study.

**===== run_2 =====**

**Tumor board decision:**
Combination therapy with mosunetuzumab and polatuzumab vedotin is recommended. Evidence: Phase 1b/2 study (PMID:38072960) with high response rate in R/R DLBCL, including CD79B mutant patients. Alternatively: Polatuzumab vedotin + bendamustine + rituximab (PMID:33942442).

**Rationale:**
CD79B p.Y197C is classified as an oncogenic according to Horak 2022 (10 points). Polatuzumab vedotin targets CD79B and shows high response rates in R/R DLBCL in studies. The combination with mosunetuzumab (CD20xCD3 bispecifics) improves effectiveness. Post-CAR-T progression requires targeted therapies.

**===== run_3 =====**

**Tumor board decision:**
Recommendation: Polatuzumab vedotin + bendamustine + rituximab (pola-BR) based on phase 2 study (PMID:33942442). Alternative study inclusion for mosunetuzumab + polatuzumab vedotin (PMID:38072960).

**Rationale:**
CD79B p.Y197C is oncogenic (Horak 2022). Polatuzumab vedotin (anti-CD79B ADC) shows efficacy in R/R DLBCL with CD79B mutations (PMID:33942442, CRR 43%). pola-BR is established as a standard therapy. Mosunetuzumab combination shows promise in trials (PMID:38072960).

## ================================================================ qwen3-next-80b (plain) ================================================================

**===== run_1 =====**

**Tumor board decision:**
Recommend epcoritamab as the next treatment option.

**Rationale:**
After CAR-T-PD, epcoritamab (CD20xCD3 bispecific antibody) is standardized according to NCCN guidelines for R/R DLBCL after >=2 therapies including CAR-T. Comorbidities (HTN, DM) can be treated with monitoring.

**===== run_2 =====**

**Tumor board decision:**
Recommendation: Start CD20xCD3 bispecific antibody therapy (e.g. glofitamab or epcoritamab) as the next treatment step.

**Rationale:**
After CAR-T failure (Axi-cel) and progression, bispecific antibody therapy is standard compliant. Glofitamab/epcoritamab are approved for R/R DLBCL after >=2 lines, including CAR-T. No contraindications due to HT/DM. Alternatives such as further chemotherapy or CAR-T switching are less effective/risky.

**===== run_3 =====**

**Tumor board decision:**
Recommend bispecific antibody therapy (e.g. glofitamab or epcoritamab) or clinical trial

**Rationale:**
After CAR-T-PD, the standard option is bispecific antibodies (glofitamab/epcoritamab) with proven efficacy in R/R DLBCL. Comorbidities (aHT, DM) can be treated with monitoring. Allogeneic HSCT not advisable due to comorbidities and previous auto-HSCT.

# ly_case_10__ALL

################################
#### CASE: 10

################################

## ================================================================ gpt-5-mini (agent) ================================================================

**===== run_1 =====**

**Tumor board decision:**
BCL2 p.A131V (Likely Oncogenic). Recommendation: venetoclax-based therapy preferably as part of a clinical study (e.g. ViPOR) or as a rational off-label combination. Alternative/complementary options: Loncastux, tafasitamab+lenalidomide; If there is a chemosensitive response, check Allo-SCT. Consider participating in alternative CAR-T studies. Mandatory before making a decision: re-biopsy (IHC CD19/CD20, EBER), CAR-T persistence qPCR, NGS and PET staging.

**Rationale:**
BCL2 p.A131V was classified as Likely Oncogenic (hotspot A131V, absent in gnomAD, OncoKB support). PubMed (PMID:38899693) describes venetoclax in ViPOR (phase1b-2) in R/R DLBCL, rationale for BCL2 inhibition. Approved options (Loncastux, Tafasitamab+Len) and studies for renewed CAR-T or Allo-SCT are relevant alternatives. Additional diagnostics are crucial for therapy.

**===== run_2 =====**

**Tumor board decision:**
No directly targeted therapy based on the NGS findings. BCL2 p.A131V is classified as a somatic VUS (score +5). Recommendation: inclusion in a clinical study or re-evaluation before targeted therapy. Mandatory additional diagnostics before a decision: re-biopsy with immunophenotype (CD19/CD20), EBER, CAR-T persistence qPCR, extended NGS including CNV/FISH and PET restaging. If actionability is lacking: check allo-SCT or antigen-adapted CAR-T/ADC/tafasi-lenalidomide as part of studies/guidelines.

**Rationale:**
Point analysis: OP4 +1, OP3 +1, OS2 +4, SBP1 -1 = Total +5 -> Classification: VUS. Despite OncoKB being classified as likely oncogenic, the overall evidence remains insufficient (contrary in silico results). Therefore no direct targeted recommendation; Study inclusion and renewed molecular/immune characterization are recommended before treatment decisions are made.

**===== run_3 =====**

**Tumor board decision:**
Primary: inclusion in a venetoclax-containing clinical trial (e.g. ViPOR) or venetoclax-based salvage therapy; If there is complete remission and patient suitability, consider Allo-SCT as consolidation. Parallel alternatives: for CD19+/CD20+ Loncastux or tafasitamab+lenalidomide; If there is CD19 loss, further CAR-T against another antigen (e.g. CD22) or participation in a study. PD-1 inhibitor for EBV+ as an option in studies.

**Rationale:**
BCL2 p.A131V = Likely Oncogenic (+8; COSMIC-Hotspot, OncoKB). PubMed (PMID:38899693) supports venetoclax-containing regimens (ViPOR) in R/R DLBCL. Mandatory before deciding on therapy: new biopsy with immunophenotype (CD19/CD20), EBER, CAR-T persistence qPCR, NGS and PET restaging.

## ================================================================ gpt-5-mini (plain) ================================================================

**===== run_1 =====**

**Tumor board decision:**
Recommendation: Priority for inclusion in a clinical trial for post-CAR-T/refractory DLBCL (particularly EBV-targeted approaches, new bispecifics/ADCs). If study not possible: off-label PD-1 inhibitor (e.g. pembrolizumab) or EBV-specific CTL, palliative RT for symptomatic lesions. Allo-SCT only with subsequent chemosensitive remission. No repetition of classes that have already failed (CD19 re-targeting, CD20xCD3, polatuzumab).

**Rationale:**

Patient with EBV+ DLBCL progressing after polatuzumab chemotherapy, CD19 CAR-T (initial CR) and subsequent CD20xCD3 bispecific -> limited standard options. EBV positivity increases plausibility for PD-1 blockade/EBV-CTL. Clinical trial provides access to more effective/novel therapies; Allo-SCT only if remission can be achieved.

**===== run_2 =====**

**Tumor board decision:**
Refractory EBV+ DLBCL after Pola-R-CHP, CD19-CAR-T (axi-cel) and CD20xCD3 bispecific: priority inclusion in clinical studies with alternative target antigens (e.g. CD22 or CD20-CAR-T, newer bispecifics, CAR-NK/T-cell engagers). If no study place is available: Consider pembrolizumab (PD-1) due to EBV positivity; If responding, check bridging to Allo-SCT.

**Rationale:**
Standard therapies and multiple drug classes have been exhausted; Repeat classes that have already been used are not displayed. Clinical trials with other antigen targets offer the most realistic chance of remission. EBV association secondarily justifies PD-1 blockade; Allo-SCT only as a curative option if the response rate is good.

**===== run_3 =====**

**Tumor board decision:**
Patient with r/r EBV+ DLBCL after R-Pola-CHP -> CD19-CAR-T (axi-cel, CR) and progression under CD20xCD3 (glofitamab). Recommendation: Urgent referral to a clinical trial with alternative CAR-T (e.g. CD22 or dual CD19/22 or CD20) or adoptive EBV-specific T-cell therapy. If study is not available: test pembrolizumab (off-label) or loncastuximab tesirine if CD19 expression is detected; allogeneic stem cell transplantation only as consolidation in the event of renewed remission.

**Rationale:**
After failure of CD19 CAR and CD20 bispecific Ab, there is a high probability of target resistance; The best possible chance of clinically relevant remission is offered by a different target/direct EBV-targeted cell therapy. Pembrolizumab or CD19 ADC are backup options depending on tumor CD19 status; allo-SCT only if response is achievable.

## ============================================================ gpt-5-nano (agent) ============================================================

**===== run_1 =====**

**Tumor board decision:**
Actionable Variant: BCL2 p.A131V is oncogenic according to Horak 2022. Recommend venetoclax-based salvage combination ViPOR (venetoclax + ibrutinib + obinutuzumab + lenalidomide + prednisone) based on phase 1b/2 data; Study prioritization. Remission: check allo-SCT; If necessary, participation in clinical studies.

**Rationale:**
Horak et al. 2022 scoring: BCL2 p.A131V +8 points (OP4+OS3+OS2+ SBP1) -> Oncogenic. PubMed: ViPOR support of BCL2 inhibition in p.A131V-positive DLBCL; COALITION mutation-independent. Conclusion: venetoclax-based salvage therapy; Allo-SCT makes sense after remission, recommend study options.

**===== run_2 =====**

**Tumor board decision:**
Actionable: Yes. Therapy recommendation: Venetoclax-based salvage therapy (BCL2 inhibitor) for relapsed EBV+ DLBCL after CAR-T; e.g. B. Venetoclax + Rituximab +/- BR. Evidence level II (Phase II). Remission allows allogeneic stem cell transplant; if necessary, participation in clinical studies on EBV-specific immunotherapy, BiTEs/ADCs; RT for symptomatic lesions.

**Rationale:**
BCL2 p.A131V Score 5 -> Oncogenic (Horak); OS2 (OncoKB) supported. FISH unremarkable. PubMed shows activity of venetoclax in BCL2-overexpressing DLBCL (Phase II). EBV-specific immunotherapy/ADCs/BiTEs as trials. Allo-SCT recommended after remission; Resistance mechanisms, among others BCL2 mutations, MCL1 upregulation.

**===== run_3 =====**

**Tumor board decision:**
Actionable: BCL2 p.A131V (Likely Oncogenic). Recommend venetoclax-based targeted therapy; e.g. B. ViPOR regimen (venetoclax + ibrutinib + obinutuzumab + lenalidomide + prednisolone) based on phase 1b/2 in BCL2-mutant DLBCL (PMID 38899693). Review study options (EBV immunotherapy/BiTE/ADC); SCT

after remission per finding.

**Rationale:**
Horak classification (+8 points) -> Likely Oncogenic. Absence in gnomAD; A131V hotspot; OncoKB function. PubMed ViPOR shows activity in BCL2-mutant DLBCL; therefore targeted option recommended. SCT after remission per course; Check study options.

## ========================================================== gpt-5-nano (plain) ==========================================================

**===== run_1 =====**

**Tumor board decision:**
Salvage therapy with loncastuximab tesirine (CD19-ADC) or tafasitamab plus lenalidomide as a bridge, followed by allogeneic stem cell transplantation (allo-SCT), if remission is achievable and the patient is suitable for transplantation.

**Rationale:**
Relapse after CAR-T; CD19-targeted therapies exhausted; Allo-SCT offers potentially curative long-term control. Salvage allows remission, then transplantation. EBV+ status does not significantly influence transplantation if comorbidities are low.

**===== run_2 =====**

**Tumor board decision:**
Review and prepare for allogeneic stem cell transplantation (alloSCT); if remission or significant tumor burden reduction can be achieved, alloSCT as a potentially curative option. If transplantation is not possible/rejected, participation in a phase I/II study or salvage with PD-1 inhibitors or lenalidomide-based therapy.

**Rationale:**
Refractory EBV+ DLBCL after CAR-T and progressive disease on glofitamab. Standard therapies exhausted; AlloSCT offers curative potential despite risk if the status is appropriate. Clinical trial prioritization allows access to new therapies and possible remission before transplant.

**===== run_3 =====**

**Tumor board decision:**
Consider and pursue allogeneic SCT in a timely manner with a suitable donor; alternatively, participation in a clinical trial with new immune/targeted therapies. If transplantation is not possible, continue experimental therapies or palliative measures.

**Rationale:**
Relapse after CAR-T and BISPEC therapy in EBV+ DLBCL. Allo-SCT offers potentially curative approach in chemoresistant disease. Clinical trials provide access to new therapies; if transplantation is not available, other evidence-based or supportive options remain.

## ========================================================== gpt-oss-20b (agent) ==========================================================

**===== run_1 =====**

**Tumor board decision:**
Consider salvage ViPOR (venetoclax+ibrutinib+obinutuzumab+lenalidomide+prednisone) as next-line after CAR-T failure. Evaluate eligibility for phaseII ViPOR extension or COALITION enrollment if high-risk.

**Rationale:**
BCL2 p.A131V is Likely Oncogenic (+8). ViPOR phase1b-2 demonstrates venetoclax efficacy in relapsed DLBCL, supported by OncoKB. No actionable FISH changes. Thus targeted BCL2 inhibition is the most suitable option.

**===== run_2 =====**

**Tumor board decision:**
No therapy-relevant variants identified; Treatment decisions follow established regimens (DHAP, ICE, ASCT) or PD-1 blockers/EBV-specific T-cell infusion/clinical studies.

**Rationale:**

BCL2 p.A131V is VUS (5 points), not an oncogenic classification - therefore not a variant-directed treatment according to Horak SOP. For EBV+ DLBCL, PD-1 inhibitors or EBV T-cell infusion as well as established chemotherapy or ASCT are options; further tests optional.

**===== run_3 =====**

**Tumor board decision:**
Venetoclax-based regimen (e.g., ViPOR protocol with venetoclax+ibrutinib+obinutuzumab or ibrutinib+venetoclax monotherapy) is recommended as next line after CAR-T failure in ECO-R. Evidence: PhaseI/II ViPOR (PMID38899693) demonstrated activity in relapsed/refractory DLBCL with BCL2 enrichment. CD20xCD3 bispecific (glofitamab) can be considered if venetoclax-based approach is not feasible.

**Rationale:**
The sole variant, BCL2p.A131V, scored LikelyOncogenic by Horak2022 criteria, making BCL2 a therapeutic target. No actionable FISH alterations were identified. PubMed evidence (PMID38899693) provides Level1/2 data for venetoclax efficacy in DLBCL, justifying its use. Other therapies lack variant-specific data.

## ============================================================ gpt-oss-20b (plain) ============================================================

**===== run_1 =====**

**Tumor board decision:**
After progression after axi-cel and PD after glofitamab step-up, a second CD19 CAR-T therapy (e.g. ide-cel) is recommended if the patient profile (age, organ function, comorbidities) is permissible. Otherwise, more intensive salvage chemotherapy (R-DHAP) followed by allo-SCT should be considered.

**Rationale:**
CAR-T-Relapse is the most common cause of progression; a second CAR-T therapy has good response rates in EBV-positive DLBCL. If not possible, allo-SCT offers long-term remission. Glofitamab-PD shows that bispecific engagers alone are not sufficient.

**===== run_2 =====**

**Tumor board decision:**
Start a salvage regimen of polatuzumab vedotin + bendamustine + rituximab (Pola-BR). After 2-3 cycles response assessment; for chemosensitive PR ASCT, for refractory second CAR-T (ide-cel) or clinical trial.

**Rationale:**
Patient with EBV+ DLBCL, after CAR-T and bispecific therapy PD. Pola-BR is evidence-based as a salvage therapy and enables ASCT preparation. If there is no response, second CAR-T is the next option according to guidelines.

**===== run_3 =====**

**Tumor board decision:**
After progression with glofitamab, we recommend a second CD19 CAR T therapy (ide-cel) after suitable bridging chemotherapy (e.g. R-GemOx). If ide-cel is not available or the patient is not suitable, evaluate EBV-specific T-cell therapy in a clinical trial.

**Rationale:**
Patient showed residual/re data after CAR-T and glofitamab; EBV-positive DLBCL is refractory to CAR-T relapse. Second CAR-T therapy with ide-cel has shown CR rates of 30-50% in studies after CAR-T relapse. Bridging chemo ensures resilience. EBV-specific T cells show promise in EBV-positive DLBCL after CAR-T therapy.

## ============================================================ gpt-oss-120b (agent) ============================================================

**===== run_1 =====**

**Tumor board decision:**
Recommendation: Participation in a study with a venetoclax-based combination regimen (e.g. ViPOR: venetoclax+ibrutinib+obinutuzumab+lenalidomide+prednisone) or off-label venetoclax+ibrutinib, provided there are no contraindications. Consider allogeneic stem cell transplantation based on response.

**Rationale:**
BCL2 p.A131V is Likely Oncogenic (+8 points) - hotspot, not in gnomAD, functional evidence. Venetoclax targets BCL-2; Phase 1b/2 study (PMID38899693) demonstrated clinical activity in DLBCL with this mutation. Literature supports BCL2 biomarker-based approach, therefore actionable.

**===== run_2 =====**

**Tumor board decision:**
Recommendation: Start a BCL2 inhibitor-based therapy. Due to the likely oncogenic BCL2 p.A131V, venetoclax should be offered in combination with ibrutinib, obinutuzumab and lenalidomide (ViPOR regimen) or, if available, assigned to an appropriate phase II study. Allogeneic stem cell transplantation remains secondary if the candidate is suitable.

**Rationale:**
BCL2 p.A131V scores +8 points (gnomAD-Absent, Hotspot, functional evidence) -> Likely Oncogenic. PubMed studies (PMID38899693) demonstrate activity of venetoclax in the ViPOR regimen in BCL2-mutant r/r DLBCL (Phase 1b/2). This means that BCL2 inhibition is evidence-based after failure of CAR-T and glofitamab.

**===== run_3 =====**

**Tumor board decision:**
If the Likely Oncogenic BCL2-p.A131V is present, a venetoclax-based combination salvage therapy (e.g. ViPOR: venetoclax+ibrutinib+lenalidomide+obinutuzumab+prednisone) is recommended (evidence level II, phase 1b/2). If there is a >=PR response, allogeneic stem cell transplantation should be considered promptly; alternatively, participation in studies with BCL2 inhibitors.

**Rationale:**
BCL2 p.A131V is Likely Oncogenic (+8 points). PubMed article 1 (PMID38899693) demonstrates clinical activity of venetoclax-based regimens in r/r DLBCL with this mutation. No relevant FISH abnormalities. The patient is progressive after CAR-T and glofitamab, so targeted BCL2 inhibition is a priority.

## ========================================================== gpt-oss-120b (plain) ==========================================================

**===== run_1 =====**

**Tumor board decision:**
After two failures of CAR-T (axi-cel) and CD20xCD3 bispecific (glofitamab), the next step should be an allogeneic stem cell transplant (allo-SCT) if the patient is transplant eligible. If you are not suitable, you should consider taking part in a clinical study protocol (e.g. PD-1 blockade or EBV-specific T-cell therapy).

**Rationale:**
The patient has an aggressive, EBV-positive DLBCL with multiple extranodal manifestations and has already completed all established lines (R-CHOP variant, CAR-T, bispecific). Allo-SCT offers the only potentially curative option after a multirefractory course; If transplantation is not possible, immunomodulatory approaches (PD-1 inhibitors, EBV-CTL) are the next evidence-based options.

**===== run_2 =====**

**Tumor board decision:**
Start salvage chemotherapy (e.g. DHAP or ICE) to reduce tumor, followed by allogeneic stem cell transplantation in the presence of a suitable donor. At the same time, you are examining the possibility of PD-1 blockade (nivolumab/pembrolizumab) or EBV-specific T-cell therapy as part of a clinical trial.

**Rationale:**
EBV-positive DLBCL is refractory to R-Pola-CHP, CD19-CAR-T and CD20xCD3-bispecific. Allogeneic transplantation is the only curative option; To do this, a responsive remission must first be achieved through salvage chemo. PD-1 inhibitors show effectiveness in EBV-associated lymphomas and offer a useful supplement or study option.

**===== run_3 =====**

**Tumor board decision:**
Recommendation: Carrying out salvage chemotherapy (e.g. R-ICE) to achieve remission, followed by allogeneic stem cell transplantation if the patient is fit; alternatively, participation in a clinical trial with EBV-specific T cells or PD-1 blockade.

**Rationale:**
The patient is refractory to R-polatuzumab-CHP, CD19-CAR-T and CD20xCD3-bispecific. Further CD19/CD20-targeted therapies have been exhausted. Allogeneic SCT after successful salvage chemotherapy offers the only potentially curative option; If you are not fit, a study arm with immunotherapy (EBV T cells, PD-1 inhibitor) should be selected.

## ============================================================ qwen3-32b (agent) ============================================================

**===== run_1 =====**

**Tumor board decision:**
Recommendation of venetoclax-based combination therapy (ViPOR) according to PM38899693 (evidence level 2B) as a targeted therapy option for recurrent BCL2-positive DLBCL. Study inclusion in phase 2 clinical trials should be considered.

**Rationale:**
The BCL2 p.A131V variant is classified as Likely Oncogenic (8 points according to Horak: hotspot, functional evidence). The ViPOR combination (venetoclax, ibrutinib, obinutuzumab) demonstrated treatment response in recurrent DLBCL in the phase 2 study (PM38899693). The patient has already received CAR-T and glofitamab, so targeted alternatives such as BCL2 inhibitors are prioritized.

**===== run_2 =====**

**Tumor board decision:**
Recommendation of venetoclax-based combination therapy (ViPOR) as a targeted treatment option for recurrent BCL2-p.A131V-positive DLBCL.

**Rationale:**
The BCL2-p.A131V variant is classified as Likely Oncogenic (8 points according to Horak). PubMed evidence (PMID:38899693) supports the use of ViPOR (venetoclax, ibrutinib, prednisone, obinutuzumab, lenalidomide) in relapsed DLBCL. The patient has already progressed on CAR-T and glofitamab, which is why BCL2-targeted therapy is a priority.

**===== run_3 =====**

**Tumor board decision:**
Recommendation of venetoclax-based combination therapy (ViPOR) according to PMID:38899693 due to the Likely Oncogenic BCL2 p.A131V variant.

**Rationale:**
The BCL2 p.A131V variant (Likely Oncogenic, 8 points) validates the relevance of BCL2 inhibitors. The ViPOR study (Phase 1b-2) shows therapeutic benefit in recurrent DLBCL. CAR-T and glofitamab refractocity warrants targeted combination therapy.

## ============================================================ qwen3-32b (plain) ============================================================

**===== run_1 =====**

**Tumor board decision:**
Recommendation: Start combination therapy with ibrutinib and another bispecific antibody therapy (e.g. donetuzumab) taking into account clinical studies.

**Rationale:**
The patient demonstrated PD after CAR-T and glofitamab. Ibrutinib (BTK inhibitors) and alternative bispecific antibodies offer new targets (e.g. CD30) and are guideline-compliant in recurrent DLBCL after CAR-T resistance.

**===== run_2 =====**

**Tumor board decision:**
Recommendation: Start combination therapy with polatuzumab vedotin (CD79b-targeted antibody-drug conjugate) and lenalidomide. Alternatively: Participation in a clinical trial with novel target strategies (e.g. CD19/CD3 dual targeting, T cell modulation).

**Rationale:**
After CAR-T resistance and PD under glofitamab, a change in target strategy is required. Polatuzumab vedotin (CD79b) combined with lenalidomide showed good results in refractory DLBCL. EBV positivity and high CNS risk profiles warrant aggressive, non-CD19-dependent approaches.

**===== run_3 =====**

**Tumor board decision:**
Recommendation: Participation in a clinical trial with CD30-targeted therapy or experimental CAR-T modification (e.g. CD22/CD19 dual targeting). If not available: mosunetuzumab (other CD20xCD3 bispecific) as the last standard option.

**Rationale:**
After CAR-T relapse and PD under glofitamab, standard therapy is exhausted. EBV positivity and CD19 negativity (not documented but possible) limit options. Study participation is prioritized, alternatively mosunetuzumab as the last bispecific option.

## ============================================================ qwen3-next-80b (agent) ============================================================

**===== run_1 =====**

**Tumor board decision:**
Recommendation for study inclusion in a clinical trial with venetoclax-based combination therapy (e.g. ViPOR regimen) due to the BCL2 p.A131V variant (Likely Oncogenic). Level of evidence: Phase 1b-2 study (PMID:38899693).

**Rationale:**
BCL2 p.A131V according to Horak et al. Classified as Likely Oncogenic in 2022 (8 points). The ViPOR study demonstrates efficacy of venetoclax in combination in DLBCL with BCL2 alterations. Therapy option recommended in clinical study.

**===== run_2 =====**

**Tumor board decision:**
Therapy with venetoclax-based regimen (ViPOR: venetoclax, ibrutinib, obinutuzumab, lenalidomide, prednisone) according to clinical study (PMID:38899693). Evidence level II.

**Rationale:**
BCL2 p.A131V is according to Horak et al. Classified as Likely Oncogenic in 2022 (8 points). The ViPOR study demonstrates clinical efficacy in BCL2-mutated DLBCL with high relevance for therapeutic targeting.

**===== run_3 =====**

**Tumor board decision:**
Enrollment in clinical trial with venetoclax-based combination therapy (e.g., ViPOR regimen) recommended due to Likely Oncogenic BCL2 p.A131V variant. Evidence level 2 (phase 1b-2 trial).

**Rationale:**
BCL2 p.A131V classified as Likely Oncogenic (8 points). PubMed evidence (PMID:38899693) shows efficacy of venetoclax-based regimens in relapsed DLBCL. ViPOR study (Phase 1b-2) offers therapeutic option. Mutation-specific resistance possible, but clinical study inclusion in CAR-T progression makes sense.

## ============================================================ qwen3-next-80b (plain) ============================================================

**===== run_1 =====**

**Tumor board decision:**
Prioritize clinical trial, particularly with EBV-targeted therapies or CD19-ADC (loncastuximab). If you do not participate in the study: Loncastuximab tesirine as the next option.

**Rationale:**
After progression on CAR-T and bispecific CD20xCD3-Ab, clinical trials are the best option. Loncastuximab is approved after two previous therapies and has a good safety profile, including in Hashimoto's thyroiditis.

**===== run_2 =====**

**Tumor board decision:**
Loncastuximab tesirine (CD19-ADC) as the next therapeutic step

**Rationale:**
After progression on CAR-T (axi-cel) and bispecific CD20xCD3 antibody (glofitamab), loncastuximab tesirine is an evidence-based option for R/R DLBCL after >=2 lines of therapy. It shows clinical effectiveness even after CAR-T failure and is approved. Alternatives such as tafasitamab/lenalidomide are possible, but prioritize CD19-targeted therapy as CD19 expression is likely to be preserved. Clinical trials should also be considered.

**===== run_3 =====**

**Tumor board decision:**
Initiation of loncastuximab tesirine (CD19-ADC) as the next line of therapy

**Rationale:**
Following progression to bispecific CD20xCD3-Ab (glofitamab) after CAR-T therapy, loncastuximab tesirine is a standardized option for relapse/refractory DLBCL after >=2 prior therapies, according to NCCN guidelines. It acts independently of CD20 expression and shows clinical efficacy even after CAR-T failure.

# ly_case_11__ALL

################################
#### CASE: 11
################################

**========================================================== gpt-5-mini (agent) ==========================================================**

**===== run_1 =====**

**Tumor board decision:**
Primary watch-and-wait: In the case of an isolated, histologically confirmed BRAF V600E-positive HZL lesion in the humerus, without contrast infiltrate and with a normal blood count, we recommend conservative observation with close monitoring. In case of pain, risk of fracture or radiological progression: orthopedic evaluation +/- local radiotherapy. Systemic therapy (standard: cladribine +/- rituximab) only in the case of cytopenia, systemic manifestation or after progression; BRAF inhibitor (e.g. vemurafenib) reserved for contraindication to purine analogues or bridging.

**Rationale:**
Consistent with guidelines: Asymptomatic HZL without cytopenia does not fulfill an indication for therapy -> W&W accepted. Bone lesions may be locally symptomatic/at risk of fracture, then local therapy. The first-line standard for systemic disease is purine analogues (cladribine). BRAF inhibitor off-label in AI against PA. Recommended staging/monitoring before/without therapy: CT chest/abdomen/pelvis or PET-CT, sonography of the spleen, repeat contrast biopsy/pretherapeutic immunophenotyping and confirmation BRAF, blood count every 3 months, imaging of the lesion (X-Ray/MRI) every 2-3 months initially or if symptoms occur; Check vaccination status.

**===== run_2 =====**

**Tumor board decision:**
We recommend: In the case of asymptomatic, isolated bone manifestation, normal blood count and no CM involvement: Watch & Wait with close monitoring (blood count q4-12 weeks) and imaging control. In case of pain/fracture risk or progression: local therapy (surgical stabilization if indicated +/- radiotherapy). Systemic therapy only for hematological indication or systemic progression: First-line purine analogue (cladribine s.c. 0.14 mg/kg x5) +/- rituximab.

**Rationale:**
Conforms to guidelines for asymptomatic classic HZL without cytopenias W&W; Bone lesions warrant interventional/radiotherapeutic treatment if symptoms or risk of fracture are present. Systemic only for cytopenias/progression: cladribine standard; BRAF inhibitor primarily when there is a contraindication to purine analogues. Before therapy: complete staging (KM biopsy with immunophenotype/BRAF, sonoabdomen, imaging MRI/PET-CT), basic lab, immune/vaccination status and infection screening.

**===== run_3 =====**

**Tumor board decision:**
Observation (watch and wait) with close monitoring; no primary systemic therapy if CM and PB are otherwise normal. If there is pain or risk of fracture: orthopedic evaluation +/- local radiotherapy or stabilization. If systemic therapy is necessary later: Cladribine s.c. (+/- rituximab) first-line; for AI against purine analogues: vemurafenib (+/- rituximab).

**Rationale:**
Contrast biopsy without infiltrate and normal blood count meet guideline criteria for W&W. Isolated bone lesion is active disease with risk of fracture -> local therapy only if symptoms/instability. Staging/monitoring before (and if necessary before start): complete blood count with diff, liver/kidneys, abdominal US, imaging (MRI/CT or FDG-PET) of the lesion, infectious serology and vaccination status. Controls: BB every 4 weeks initially, abdominal US q3M. Cladribine is standard for systemic indications; BRAF inhibitor reserved for AI/bridging (for BRAF V600E dermatological monitoring).

**========================================================== gpt-5-mini (plain) ==========================================================**

**===== run_1 =====**

**Tumor board decision:**

Systemic therapy is currently not indicated. Recommendation: complete staging (PET CT or whole body MRI) and orthopedic/oncological assessment of the humeral lesion. For solitary, resectable findings: surgical resection/curettage +/- local radiotherapy. For multifocal/systemic infestation: systemic therapy (primarily cladribine). If refractory, consider BRAF inhibitor.

**Rationale:**
Patient without bone marrow involvement and with normal blood values; therefore no immediate systemic therapy. Solitary extranodal bone manifestations can primarily be treated locally. Staging necessary for therapy planning. BRAFV600E offers a later targeted option for refractory/recurrent disease.

**===== run_2 =====**

**Tumor board decision:**
Further staging examination (PET-CT or whole body MRI) and orthopedic assessment. For isolated solitary humerus manifestation: local therapy (preferably fractionated radiotherapy e.g. 30 Gy or surgical resection/curettage if indicated). No immediate systemic therapy; Close follow-up checks (BM, PB, clinic).

**Rationale:**
No bone marrow involvement, normal blood values and no previous systemic therapy -> no indication for immediate systemic therapy (purine alkylates). For solitary bone involvement, local treatment is sufficient. PET/MRI for staging; BRAF inhibitor/cladribine only in systemic progression or cytopenias.

**===== run_3 =====**

**Tumor board decision:**
If the course is currently asymptomatic and blood values are normal: close observation (blood count q4-8 weeks, clinical control, targeted imaging of the humerus). For symptomatic or progressive bony lesions or systemic involvement: first-line therapy with cladribine (IV single cycle). Local therapy (surgery/radiotherapy) if local symptoms are found. If purine analogues are contraindicated: BRAF inhibitor (vemurafenib).

**Rationale:**
Hairy cell leukemia with BRAF V600E, but currently no cytopenias or bone marrow infiltration -> no immediate systemic therapy necessary. Cladribine is standard for HCL requiring treatment. Local therapy for symptomatic bone manifestation. BRAF inhibitor as a sensible alternative in the event of intolerance/relapse.

## ============================================================ gpt-5-nano (agent) ============================================================

**===== run_1 =====**

**Tumor board decision:**
First-line therapy: purine analogues (cladribine) as standard; If necessary, rituximab for residual lesions. Local radiotherapy of the proximal humerus lesion if there is a risk of fracture or pain. Further staging (CT/PET) and close monitoring (CBC q4 weeks, imaging every 3-6 months). BRAFV600E inhibitors as a bridge or in cases of PA intolerance/relapse.

**Rationale:**
PA are standard in HZL with a good prognosis; extramedullary lesions addressable via local therapy; BRAF inhibitors useful in cases of PA contraindication/relapse or faster response; Rituximab optional for residual lesions.

**===== run_2 =====**

**Tumor board decision:**
Watch & Wait as first-line therapy due to normal cytopenias and asymptomatic extramedullary humeral lesion. Regular checks. In case of progress or symptoms: purine analogues (cladribine or pentostatin) +/- rituximab. Against PA-KI or the need for rapid remission: BRAFV600E inhibitors as bridging. Local radiotherapy for painful lesions/risk of fracture; further staging/monitoring.

**Rationale:**
Decision follows the HCL flowchart: asymptomatic, no contrast infiltration; PA standard therapy if therapy is required; BRAFV600E inhibitors off-label as a bridge; extramedullary lesion may require local RT, currently no urgent need for systemic therapy; Regular monitoring according to SOP/Onkopedia.

**===== run_3 =====**

**Tumor board decision:**
First-line: purine analogues (cladribine SC 0.14 mg/kg/dx5 days or pentostatin) +/- rituximab for residual infiltration. Local radiotherapy of the humeral lesion for pain/risk of fracture. Imaging staging (PET-CT) and regular check-ups; MRD not standard. BRAF inhibitors only as bridging in AI against PA.

**Rationale:**
PA standard leads to high CR/MRD neg rates; extramedullary lesions respond well; RT directly addresses lesion; BRAF inhibitors enable rapid responses as a bridge or alternative in the event of contraindications.

## ============================================================ gpt-5-nano (plain) ============================================================

**===== run_1 =====**

**Tumor board decision:**
First-line treatment of hairy cell leukemia with purine analogue (cladribine) or pentostatin. Optional rituximab in combination. For focal bone lesions, consider additional local radiation therapy. Close monitoring (blood count, biomarkers).

**Rationale:**
Purine analogues achieve high remission rates even in HCL with extramedullary manifestation; BRAF-V600E confirmed, but standard first-line systemic. Radiotherapy optional for symptomatic lesion; no pretreated therapies.

**===== run_2 =====**

**Tumor board decision:**
Cladribine 0.1 mg/kg IV daily, 5 days as first-line therapy; close monitoring (blood count, infections); Extramedullary bone manifestation is likely to be addressed. If recurrence/inadequate response: BRAF-V600E inhibitor (vemurafenib) +/- MEK inhibitor; If necessary, rituximab as a supplement.

**Rationale:**
Standard first-line therapy for hairy cell leukemia is purine analogue (cladribine) with a high remission rate, even in cases of extramedullary bone involvement. BRAF-V600E enables later targeted therapy in case of recurrence; no previous treatments so far; good prerequisite for therapy response.

**===== run_3 =====**

**Tumor board decision:**
Primary therapy for HCL: Purine analogues (cladribine 0.09-0.14 mg/kg/d IV over 5-7 days) as first-line. If there is extranodal bone manifestation, rituximab if necessary. Close controls. If relapse/incomplete remission: BRAF inhibitors (vemurafenib/dabrafenib) +/- MEK inhibitor; If necessary, sequence change.

**Rationale:**
Standard first-line with high remission rate; extramedullary bone involvement typically responds to purine analogues; BRAF inhibitors reserved for relapse/intolerance.

## ============================================================ gpt-oss-20b (agent) ============================================================

**===== run_1 =====**

**Tumor board decision:**
Observation without therapy. Regular blood count and image checks (every 3-6 months). Therapy (e.g. vemurafenib) only in the case of symptomatic progression or risk of fracture.

**Rationale:**
The patient has no cytopenia, no symptoms, and only an isolated bone lesion. According to TI-1 and the algorithm, watch-and-wait is permissible in asymptomatic, systemically uninvolved HZL. BRAF inhibitor is off-label and only recommended for symptomatic lesions.

**===== run_2 =====**

**Tumor board decision:**
observation without immediate therapy; regular blood count and image checks (every 3 months) and if pain occurs, risk of fracture or growth of the lesion, immediate therapy (e.g. BRAF inhibitor or

local radiotherapy).

**Rationale:**
The patient has no cytopenias, no symptoms, and no systemic involvement. According to TI-1 and algorithm, watch-and-wait is permitted for asymptomatic, isolated bone lesions; Therapy only if clinical deterioration occurs.

**===== run_3 =====**

**Tumor board decision:**
Observation without immediate therapy. Regular blood count checks every 3 months and imaging checks (X-ray/CT) of the humerus every 6 months. If pain, risk of fracture or cytopenias occur, immediate therapy (e.g. cladribine or BRAF inhibitor).

**Rationale:**
The patient has no systemic involvement, no cytopenias, and no symptomatic lesion. According to TI-1 of the flowchart, watch and wait is acceptable for asymptomatic, isolated bone lesions. BRAF inhibitors are only indicated if there are symptoms or intolerance to purine analogues. Regular checks enable early intervention in the event of progression.

## ========================================================== gpt-oss-20b (plain) ==========================================================

**===== run_1 =====**

**Tumor board decision:**
Initiate initial treatment with cladribine (0.1 mg/kg IV daily for 5 days, 2 cycles).

**Rationale:**
Hairy cell leukemia without prior therapy is the standard first line with cladribine or pentostatin. BRAF V600E positivity indicates a good response; extramedullary manifestations can also be treated with cladribine. There are no contraindications.

**===== run_2 =====**

**Tumor board decision:**
Initiate first-line treatment with cladribine 0.1mg/kg IV/SC daily for 5 days (or 0.1mg/kg SC for 5 days). Evaluate response after 4-6 weeks; In case of progression, plan BRAF inhibitor (vemurafenib/dabrafenib+trametinib).

**Rationale:**
Cladribine is the established first-line option for HCL, even in extramedullary manifestations. BRAF V600E positivity supports subsequent use of BRAF/MEK inhibitors, but is not mandatory for initial treatment. Patients without comorbidities tolerate cladribine well.

**===== run_3 =====**

**Tumor board decision:**
Initiate first-line therapy with cladribine (0.1 mg/kg IV daily for 5 days, 2 cycles). If there is intolerance or progression, pentostatin can be used. Vemurafenib is reserved as a second-line option in BRAFV600E-positive HCL.

**Rationale:**
Cladribine is the established first-line treatment for HCL, even in extramedullary manifestations. It offers high remission rates and is well tolerated. Vemurafenib is preferred for refractory or difficult-to-treat disease.

## ========================================================== gpt-oss-120b (agent) ==========================================================

**===== run_1 =====**

**Tumor board decision:**
Watch-and-Wait; close clinical and imaging monitoring. In the case of symptomatic bone disease (pain, risk of fracture), local radiation of the humerus; Systemic therapy (cladribin +/- rituximab) only when cytopenias or progressive extramedullary disease occur.

**Rationale:**
The patient has no cytopenias, no bone marrow infiltrate, and only isolated, asymptomatic bone disease. According to the guidelines, W&W is permitted if there is no indication for therapy; Local

radiotherapy treats the risk of fractures, while purine analogues are only used when clinically indicated.

**===== run_2 =====**

**Tumor board decision:**
If there is no cytopenia and an asymptomatic extramedullary lesion: initially watch and wait with blood counts and image checks every 3 months. In case of pain, risk of fracture or progression, local radiation of the proximal humerus. Systemic therapy (cladribine +/- rituximab or BRAF inhibitor) only if clinically indicated.

**Rationale:**
The patient does not fulfill any therapy indication after TI-1 (no cytopenia, no B symptoms). Isolated bone disease can be controlled by local radiotherapy, while systemic therapy is withheld because of good prognosis and potential toxicity.

**===== run_3 =====**

**Tumor board decision:**
Systemic therapy with cladribine+rituximab (1 cycle, repeat until CR if necessary) combined with local radiotherapy of the humeral lesion (e.g. 24Gy), since active extramedullary manifestation is present.

**Rationale:**
No cytopenias, but isolated bone lesion with risk of fracture. Guideline recommends therapy for symptomatic/active lesions. Cladribine is standard first-line therapy, rituximab increases MRD negativity. Radiation ensures local control.

## ============================================================ gpt-oss-120b (plain) ============================================================

**===== run_1 =====**

**Tumor board decision:**
Start first-line therapy with cladribine (0.14 mg/kg i.v. 5 days) in combination with rituximab (375 mg/m2 weekly 8 cycles).

**Rationale:**
Hairy cell leukemia with BRAFV600E positivity and extramedullary bone disease, but without hematological insufficiency. Purine analogue therapy is standard; The addition of rituximab increases the remission rate and reduces the MRD rate. BRAF inhibitors are only used in refractory disease.

**===== run_2 =====**

**Tumor board decision:**
For asymptomatic, non-infiltrated HCL with an isolated extramedullary lesion in the humerus, we initially recommend local radiotherapy (e.g. 24Gy in 12 Fr.) and close clinical and hematological monitoring. Systemic therapy (cladribine or BRAF inhibitor) is only initiated in the event of hematological progression or symptomatic expansion.

**Rationale:**
The patient has no cytopenias or bone marrow infiltration; The clinical picture does not currently require treatment. The local bone lesion can be controlled by radiotherapy while systemic therapy is withheld because of possible toxicity. BRAFV600E positivity is relevant for recurrent cases, not for initial observation.

**===== run_3 =====**

**Tumor board decision:**
First-line therapy with a purine analog combination: Cladribine 0.14 mg/kg i.v. /s.c. over 5 days plus Rituximab 375mg/m2 weekly for 4 cycles. If pain occurs, consider local irradiation of the humerus. For refractory disease, consider BRAF inhibitor (e.g. vemurafenib) +/- MEK inhibitor.

**Rationale:**
The patient has classic HCL without bone marrow infiltration but with extramedullary bone disease. Standard first-line is cladribine +/- rituximab, which delivers high response rates and long remissions. The presence of BRAFV600E is relevant for the use of BRAF inhibitors in treatment failure, but not for the first line.

**============================================================ qwen3-32b (agent) ============================================================**

**===== run_1 =====**

**Tumor board decision:**
Recommended therapy: Cladribine + Rituximab as frontline standard. For BRAF-V600E-positive HCL with extranodal manifestation, vemurafenib + rituximab can be considered as an alternative therapy, taking into account the shorter duration of remission.

**Rationale:**
Cladribine/rituximab is evidence-based frontline regimen. BRAF inhibitors such as vemurafenib show efficacy in recurrent HCL but limited duration of remission. Extranodal manifestations require systemic therapy. Local radiotherapy as an adjuvant option for persistent extranodal lesions.

**===== run_2 =====**

**Tumor board decision:**
Recommended therapy: Vemurafenib + rituximab as first-line therapy for BRAFV600E-positive extranodal hairy cell leukemia.

**Rationale:**
BRAF mutation (V600E) is relevant to therapy. Combination of BRAF inhibitor (vemurafenib) and anti-CD20 (rituximab) shows 91% response rate in BRAF-mutated HCL cases in the literature. Extranodal manifestation warrants targeted therapy. Bone marrow negativity does not rule out systemic disease. No contraindications in laboratory values.

**===== run_3 =====**

**Tumor board decision:**
Recommended therapy: Cladribine (0.14 mg/kg SC x5 days) for the treatment of BRAFV600E-positive extranodal HZL lesion. If remission is stable after 4 months, follow-up checks every 3 months.

**Rationale:**
BRAFV600E positivity and extranodal manifestation despite negative bone marrow aspiration indicate active disease. Cladribine is the first-line standard with high CR rates. Local radiation is alternative, but SOP prefers systemic therapy for complete remission. W&W is alternative in asymptomatic patients with stable lesions, but carries the risk of recurrence.

**============================================================ qwen3-32b (plain) ============================================================**

**===== run_1 =====**

**Tumor board decision:**
Initiate first-line therapy with cladribine (Leukeran) due to the diagnosis of hairy cell leukemia. Local radiotherapy of the left humerus. as an adjuvant option for persistent extranodal involvement after chemotherapy response.

**Rationale:**
Cladribine is the standard therapy for hairy cell leukemia with high response rates. BRAFV600E positivity supports sensitivity to purine analogues. Extranodal manifestation warrants preventive radiotherapy for subsequent residual disease.

**===== run_2 =====**

**Tumor board decision:**
Recommendation: Initiation of vemurafenib (BRAF inhibitor) as first line in BRAF V600E-positive hairy cell leukemia with extranodal manifestation. Consider combination with cobimetinib (MEK inhibitor) for resistance prevention.

**Rationale:**
BRAF V600E positivity suggests BRAF inhibitors as standard therapy. Extranodal manifestations require systemic approaches. Combination therapy reduces the risk of resistance. No contraindications or previous therapies known.

**===== run_3 =====**

**Tumor board decision:**
Recommendation: Initiate BRAF inhibitor-based therapy (e.g. vemurafenib) as first line due to the BRAFV600E-positive phenotype and extranodal manifestation.

**Rationale:**
The BRAFV600E mutation is a predictive biomarker for BRAF inhibitors in hairy cell leukemia. Extranodal infiltrates often respond to BRAF targeting. Purine analogues are alternative, but BRAF targeting is preferred in the event of a mutation.

## ============================================================ qwen3-next-80b (agent) ============================================================

**===== run_1 =====**

**Tumor board decision:**
No systemic therapy. Local radiation only if the lesion is symptomatic (pain, risk of fracture) or progressive dynamics. Otherwise watch & wait with regular checks (blood count, imaging of the humerus).

**Rationale:**
According to the Onkopedia guideline (TI-1), there is no indication for systemic therapy in isolated extramedullary manifestations without cytopenias, absence of contrast infiltrate and absence of symptoms. Local radiation is only indicated if the lesion is symptomatic or at risk of fracture. Watch & Wait with monitoring is appropriate.

**===== run_2 =====**

**Tumor board decision:**
No systemic or local therapy. Watch & Wait with regular checks (blood work every 3 months, imaging if symptoms are suspected). If the lesion is symptomatic (pain, risk of fracture), consider local radiotherapy.

**Rationale:**
According to the flowchart (TI-1), there is no indication for therapy in isolated extramedullary manifestations without cytopenia, contrast infiltrate or symptoms. Systemic therapy is contraindicated. Local radiotherapy only for symptomatic lesions. Watch & Wait is standard.

**===== run_3 =====**

**Tumor board decision:**
No systemic or local therapy indicated. Watch & Wait with blood count checks every 3 months and imaging monitoring of the bone lesion (e.g. MRI/X-ray).

**Rationale:**
Isolated extramedullary manifestation with normal blood count, negative bone marrow and no symptoms does not fulfill an indication for therapy (TI-1 flowchart). Systemic therapy is not indicated. Consider local radiation only if the lesion is symptomatic (pain/risk of fracture). Watch & Wait with regular monitoring is standard.

## ============================================================ qwen3-next-80b (plain) ============================================================

**===== run_1 =====**

**Tumor board decision:**
Observation without active therapy with regular monitoring (blood count, clinical examination every 3 months)

**Rationale:**
No indication for therapy (no cytopenias, no symptoms), according to guidelines. Extranodal manifestation alone is not an indication of therapy if the course is asymptomatic.

**===== run_2 =====**

**Tumor board decision:**
Initiate cladribine therapy as first-line therapy for hairy cell leukemia. Extranodal manifestation requires systemic treatment, not local measures.

**Rationale:**
BRAF V600E positivity is typical of HCL, but first-line therapy remains purine analogues (cladribine). Systemic therapy required because HCL is systemic. Local measures are not indicated for extranodal manifestations.

**===== run_3 =====**

**Tumor board decision:**
Initiation of cladribine therapy as first-line therapy

**Rationale:**
HCL is systemic, extranodal manifestation requires therapy. Cladribine is standard first-line with a high response rate. BRAF V600E status does not change first-line therapy. No contraindications or comorbidities.

# ly_case_12__ALL

################################
#### CASE: 12
################################

## ============================================================ gpt-5-mini (agent) ============================================================

**===== run_1 =====**

**Tumor board decision:**
Primary recommendation: Start BTK inhibitor therapy, preferably zanubrutinib, as the next systemic therapy. In case of intolerance or progression: if possible, inclusion in a study (non-covalent BTKi or anti-CD38 study). Anti-CD20-based concepts are unsuitable because of complete CD20 loss; autologous SCT is not primarily indicated.

**Rationale:**
CD20 negativity after rituximab makes further anti-CD20 strategies (e.g. R2) of little use. Literature and congress data show BTKi (especially zanubrutinib) effective in R/R MZL after anti-CD20. Plasma cell differentiation justifies testing of experimental anti-CD38 approaches or clinical trials; Chemotherapy/SCT only for specific indications.

**===== run_2 =====**

**Tumor board decision:**
Primary initiation of a BTK inhibitor (e.g. zanubrutinib) as the next systemic therapy; priority for inclusion in a study (non-covalent BTKi or BTKi combination). Do not re-administer Anti-CD20. Anti-CD38/lenalidomide approaches or autologous SCT only in studies or selectively for specific indications.

**Rationale:**
Complete loss of CD20 renders rituximab ineffective. For relapsed/refractory MZL after anti-CD20, BTKi (zanubrutinib) is the most evidence-supported. New BTKi/studies offer additional options. Anti-CD38, lenalidomide or auto-SCT have limited data and are preferably trial-based.

**===== run_3 =====**

**Tumor board decision:**
Further treatment: Start BTK inhibition with zanubrutinib (approved option for relapsed/refractory MZL) as primary next systemic therapy. Parallel: Arrange pathology for CD38 IHC. If BTKi is contraindicated/intolerable: systemic chemotherapy (e.g. bendamustine without anti-CD20) or inclusion in a clinical study. Anti-CD38 (e.g. daratumumab) only in study/with strong CD38 positivity and interdisciplinary consideration. ASCT is currently not recommended.

**Rationale:**
Complete CD20 loss renders anti-CD20 therapy ineffective. BTK inhibitors (zanubrutinib) are effective and approved in MZL after anti-CD20. Plasmacellular differentiation warrants CD38 testing; The anti-CD38 approach is experimental/off-label and only justified if there is evidence/study. ASCT is not standard in indolent MZL.

## ============================================================ gpt-5-mini (plain) ============================================================

**===== run_1 =====**

**Tumor board decision:**
Start of BTK inhibitor therapy: Ibrutinib 560 mg p.o. daily as the next systemic therapy for recurrent/progressive marginal zone lymphoma after rituximab (complete CD20 loss). Before starting therapy: new biopsy if transformation is suspected, basic laboratory, ECG, blood pressure and interaction check.

**Rationale:**
After PD after 4 cycles of rituximab and complete CD20 loss, renewed anti-CD20 therapy does not make sense. Ibrutinib is approved for R/R MZL after anti-CD20 and shows good efficacy. If there is a contraindication to BTKi: bendamustine-based chemotherapy or study participation as an alternative.

**===== run_2 =====**

**Tumor board decision:**
For PD after 4 cycles of rituximab and complete CD20 loss: no further anti-CD20 therapy. Primary recommendation: Start BTK inhibitor therapy (ibrutinib) as second line. In the case of rapid/symptomatic progression, alternative systemic cytostatics (e.g. bendamustine) or inclusion in a clinical study.

**Rationale:**
Ibrutinib shows efficacy in recurrent marginal zone lymphoma and acts independently of CD20 expression. CD20 loss renders repeated rituximab administration ineffective. Bendamustine is a useful alternative in cases of high tumor burden or rapidly progressive disease. Observe monitoring and infection prophylaxis.

**===== run_3 =====**

**Tumor board decision:**
Starting BTK inhibitor therapy as the next step: Ibrutinib 560 mg p.o. daily (alternatively zanubrutinib 160 mg orally 2x/day) because of PD after rituximab and complete CD20 loss. In parallel: another biopsy to clarify plasma cellular differentiation/transformation and staging (PET-CT, KM).

**Rationale:**
CD20 loss renders renewed anti-CD20 therapy ineffective. BTK inhibitors are approved and effective in relapsed/progressive marginal cell lymphoma after anti-CD20 therapy. Rebiopsy and staging clarify transformation/prognosis and, if necessary, alternative/clinical treatment options.

## ============================================================ gpt-5-nano (agent) ============================================================

**===== run_1 =====**

**Tumor board decision:**
BTK inhibitor as first choice (zanubrutinib) in R/R CD20-negative MZL after RTX progression; Biomarker screening before start (BTK/PLCG2 mutations, IgM/Paraprotein); in case of progression: non-covalent BTKi or BTKi combination in studies; local RT for focal lesions; Possibly ASCT if you are in good shape.

**Rationale:**
MAGNOLIA Demonstrates BTKi Efficacy in R/R MZL; Conference data supports sequence after RTX; Biomarkers help personalization; Backup options (PI3K inhibitors, ASCT) are case dependent; Trials recommended.

**===== run_2 =====**

**Tumor board decision:**
Prefer BTK inhibitors: Zanubrutinib in CD20-negative MZL after rituximab progression; in case of progression/intolerance nemtabrutinib or other BTKi; studies preferred; ASCT in Fitness; PI3K inhibitors as a reserve; Biomarkers before therapy: BTK mutations, API2-MALT1, plasmacytoid differentiation.

**Rationale:**
BTKi have the strongest evidence in R/R MZL after rituximab; CD20 status irrelevant. ASCT useful for fitness; Prioritize study access; PI3K inhibitors toxic; Biomarkers support.

**===== run_3 =====**

**Tumor board decision:**
Zanubrutinib as preferred second-line in CD20-negative MZL (stage III) after rituximab progression; clinical trial preferred. Alternatives: Bendamustine monotherapy without rituximab or PI3K inhibitors. Autologous stem cell transplantation only in very fit refractory patients after response. Biomarkers before therapy: baseline blood count, LDH, beta2-MM, HBV/HCV/HIV, HP status; Consider MZL-IPI.

**Rationale:**
CD20 targeting is ineffective after progression; BTK inhibition (e.g. zanubrutinib) shows activity in rel/ref MZL. Clinical trials allow for expanded options. Radiotherapy is not first-line in disseminated stage III. SCT only in very selected, remission-capable patients after response. There is currently no established biomarker-based selection.

## ============================================================ gpt-5-nano (plain) ============================================================

**===== run_1 =====**

**Tumor board decision:**
BTK inhibitor therapy (ibrutinib 420 mg/d) for relapsed CD20-negative MZL with plasma cell differentiation after rituximab; Rituximab is ruled out. Alternative: Rd regime if BTK-I is unsuitable.

**Rationale:**
CD20 loss; Rituximab ineffective; BTK inhibitors show effectiveness in relative MZL independent of CD20; plasma cell differentiation; Rd as a backup.

**===== run_2 =====**

**Tumor board decision:**
Switch to monotherapy with a BTK inhibitor (e.g. ibrutinib) in RR-MZL after rituximab and CD20 loss. Alternative: Zanubrutinib/Acalabrutinib depending on availability. Avoid rituximab-based options; if necessary clinical study.

**Rationale:**
CD20 loss renders rituximab ineffective. BTK inhibitors show activity in RR-MZL; enables oral therapy, outpatient management. No specific targeting mechanism from NGS/FISH specified; Consideration of a clinical study possible.

**===== run_3 =====**

**Tumor board decision:**
BTK inhibitor as next step: Ibrutinib 420 mg p.o. daily (alternatively zanubrutinib 320 mg bid) for relapse/refractory MZL with CD20 loss. Rituximab-based therapies unsuitable; If necessary, inclusion in clinical studies.

**Rationale:**
CD20 loss after rituximab; Anti-CD20 therapies ineffective. BTK inhibitors show significant response rates in relapsed/refractory MZL, including in plasmacytic differentiation; acceptable risk-benefit ratio; optional studies possible.

## ============================================================ gpt-oss-20b (agent) ============================================================

**===== run_1 =====**

**Tumor board decision:**
Recommendation: Start BTK inhibitor therapy (e.g. zanubrutinib 160 mg PO daily) as the next treatment step. In case of inadequate response or tolerance problems, alternative options: nemtabrutinib or orelabrutinib; CAR T cells or autologous stem cell transplantation only if there is further progression.

**Rationale:**
Patient has CD20-negative MZL, PD after rituximab mono. Studies (2 PubMed) and conference papers (3 ASH/ASCO/EHA) show ORR 50-70% for BTK inhibitors, better efficacy than ibrutinib and good tolerability. Therefore, BTK inhibitor is the preferred next option.

**===== run_2 =====**

**Tumor board decision:**
Start zanubrutinib 160 mg PO daily as a monotherapeutic option; if there is no approval or intolerance, ibrutinib 560 mg PO; alternatively bortezomib+lenalidomide or CAR T cells in clinical study; ASCT only with good performance status and no remission.

**Rationale:**
CD20 negativity renders rituximab ineffective. BTK inhibitors (zanubrutinib) have ORR~68% in refractory MZL in phase II studies. Ibrutinib is alternative. Bortezomib/lenalidomide show activity in small studies. CAR-T and ASCT are backup options in severe progression.

**===== run_3 =====**

**Tumor board decision:**
Zanubrutinib 160mg PO daily (BTK inhibitor) as next therapy after CD20 negativity; alternatively ibrutinib or other BTK inhibitors if not available. CAR T cells or autologous. Stem cell transplant only in case of further progression or in studies.

**Rationale:**
CD20 negativity renders rituximab ineffective. Guidelines (S2) recommend BTK inhibitors (zanubrutinib) with ORR~68% in MZL. Other options (bortezomib, lenalidomide) have less evidence. Autolog. SCT and CAR-T are for later lines and studies, respectively.

## ======================================================== gpt-oss-20b (plain) ========================================================

**===== run_1 =====**

**Tumor board decision:**
Start bendamustine monotherapy (90mg/m2 on days 1-2, 28-day interval).

**Rationale:**
After 4 cycles of rituximab, the patient has PD and CD20-negative plasma cells, so rituximab no longer has any effect. Bendamustine is a CD20-independent alkylator regimen with demonstrated efficacy in marginal zone lymphoma and acceptable tolerance.

**===== run_2 =====**

**Tumor board decision:**
After CD20 negativity and progression after rituximab, we recommend initiating ibrutinib 420 mg daily as a monotherapeutic option (alternatively bendamustine + lenalidomide in the case of comorbidities).

**Rationale:**
CD20 negativity renders further anti-CD20 therapies ineffective. Ibrutinib has shown efficacy after rituximab in marginal zone lymphoma and is well tolerated. Bendamustine+Lenalidomide is another alternative when ibrutinib is contraindicated.

**===== run_3 =====**

**Tumor board decision:**
Bendamustine 90mg/m2 (days 1-2 q28d) as a monotherapeutic second-line approach. If there is no remission or further progression, consider alternative options (e.g. lenalidomide, clinical trial).

**Rationale:**
CD20 loss renders rituximab ineffective. Bendamustine shows high remission rates in relapsing MZL, regardless of CD20 status. Monotherapy reduces antibody dependence and is well tolerated.

## ======================================================== gpt-oss-120b (agent) ========================================================

**===== run_1 =====**

**Tumor board decision:**
Start of BTK inhibitor therapy with zanubrutinib (160 mg twice daily) as the next systemic treatment for advanced (Ann-ArborIII) marginal zone lymphoma after unsuccessful rituximab monotherapy and CD20 loss. If unsuitable, consider clinical trial or bendamustine-based chemotherapy.

**Rationale:**
Patient has progressive MZL in stage III, anti-CD20 therapy has already failed and CD20 is lost, so rituximab is no longer effective. According to the guidelines, a BTK inhibitor (zanubrutinib) is approved for relapsed/refractory MZL after anti-CD20 therapy and shows good ORR. Systemic therapy is required, local options are not suitable.

**===== run_2 =====**

**Tumor board decision:**
In advanced Ann Arbor III MZL with progressive disease after rituximab monotherapy and complete CD20 loss, we recommend zanubrutinib (BTK inhibitor) monotherapy as the next therapy in accordance with the S2 recommendation, if necessary as part of a study.

**Rationale:**
CD20 negativity renders further anti-CD20 therapy ineffective. Stage III requires systemic therapy. BTK inhibitors (zanubrutinib) show ORR~68% in the MAGNOLIA study and are approved for refractory MZL; they are well tolerated and match the disease situation.

**===== run_3 =====**

**Tumor board decision:**

In advanced MZL (Ann-ArborIII) with progressive disease after rituximab monotherapy and complete CD20 loss, BTK inhibitor therapy is recommended as the next step: zanubrutinib 160 mg orally twice daily (alternative clinical study).

**Rationale:**
Rituximab is not effective due to CD20 negativity. According to the guidelines, a BTK inhibitor (zanubrutinib) is approved for relapsed/refractory MZL after anti-CD20 therapy and shows a high response rate and good tolerability. Systemic therapy is required for stage III.

## ========================================================== gpt-oss-120b (plain) ==========================================================

**===== run_1 =====**

**Tumor board decision:**
Start of BTK inhibitor therapy (e.g. ibrutinib 560 mg daily) as the next systemic treatment of CD20-negative, rituximab-resistant marginal zone lymphoma (Ann-ArborIII).

**Rationale:**
The lymphoma is progressive after monotherapy rituximab and has completely lost CD20, making further anti-CD20 therapies ineffective. BTK inhibitors have been proven to show a high response rate in relapsed/refractory MZL and are oral and well tolerated.

**===== run_2 =====**

**Tumor board decision:**
Due to the progression after rituximab monotherapy and the complete loss of CD20, BTK inhibitor therapy is recommended as the next step, e.g. B. Ibrutinib 560mg orally daily as monotherapy. If necessary, a short bendamustine induction can be added. Participation in a clinical study is also offered.

**Rationale:**
Rituximab is ineffective due to CD20 negativity. BTK inhibitors (ibrutinib) show high response rates and good tolerability in relapsed marginal zone lymphoma. A short course of chemotherapy can deepen remission, while clinical trials provide access to new agents.

**===== run_3 =====**

**Tumor board decision:**
Due to progressive marginal zone lymphoma in stage III after monotherapeutic rituximab therapy with CD20 negativity, we recommend BTK inhibitor therapy (e.g. ibrutinib 560 mg orally daily) as the next step. In refractory disease and suitable performance capacity, a CD19 CAR T cell therapy approach can be considered.

**Rationale:**
Rituximab is no longer effective due to the complete loss of CD20. BTK inhibitors show high effectiveness in CD20-negative MZL and are well tolerated orally. CAR T cells offer a curative option if the disease progresses further.

## ========================================================== qwen3-32b (agent) ==========================================================

**===== run_1 =====**

**Tumor board decision:**
Recommendation: Zanubrutinib monotherapy as the next step in CD20-negative MZL with progression on rituximab.

**Rationale:**
Zanubrutinib (BTK inhibitor) is established in CD20-negative, relapsed MZL with high ORR and good safety. Rituximab combinations are not suitable due to CD20 loss. New BTK inhibitors such as nemtabrutinib are alternative back-up options.

**===== run_2 =====**

**Tumor board decision:**
Recommendation: Zanubrutinib (BTK inhibitor) as two-line therapy for progression with rituximab monotherapy and CD20 loss. If the infection is localized, add ISRT if necessary.

**Rationale:**

Progression under anti-CD20 therapy with loss of CD20 expression requires switching away from CD20-dependent therapies. According to the guidelines, zanubrutinib (approved, ORR 68%) is the first therapeutic approach for recurrent/refractory MZL. Plasmacellular differentiation indicates an aggressive variant that requires systemic therapy.

**===== run_3 =====**

**Tumor board decision:**
Recommendation: Therapy with zanubrutinib or nemtabrutinib (BTK inhibitors) as first line. If well tolerated, alternatively orelabrutinib in combination with chemo.

**Rationale:**
CD20 loss renders rituximab ineffective. BTK inhibitors show high ORR in relapsed MZL with CD20 negativity. Combination therapies are complementary in plasma cellular differentiation.

## ================================================================ qwen3-32b (plain) ================================================================

**===== run_1 =====**

**Tumor board decision:**
Switch to combination therapy with bendamustine and obinutuzumab. If poor performance or contraindications: Consider lenalidomide-based therapy.

**Rationale:**
Progression under rituximab monotherapy with CD20 loss and plasma cell differentiation. Obinutuzumab (anti-CD20) and bendamustine provide synergistic effects. Lenalidomide as an alternative for non-CD20-dependent disease.

**===== run_2 =====**

**Tumor board decision:**
Switch from rituximab monotherapy to combination therapy with bendamustine or chlorambucil due to CD20 loss and progression.

**Rationale:**
CD20 loss renders rituximab ineffective. Bendamustine/chlorambucil offers established efficacy in MZL for recurrent disease without CD20 dependence.

**===== run_3 =====**

**Tumor board decision:**
Switch to R-bendamustine or R-DHAP due to CD20 loss and PD on rituximab monotherapy. In plasmacytoid differentiation, consider bortezomib-based combination therapy as an alternative.

**Rationale:**
Progression under CD20-targeted monotherapy and complete CD20 loss require therapy changes. Bendamustine/R-CHOP are standard options for recurrent MZL. Bortezomib may be useful in plasmacytoid transformation. Stage III speaks for systemic therapy.

## ================================================================ qwen3-next-80b (agent) ================================================================

**===== run_1 =====**

**Tumor board decision:**
Zanubrutinib as the next therapeutic step, since CD20 loss contraindicates rituximab and there is progression under anti-CD20 therapy. Zanubrutinib is approved and shown in MAGNOLIA trial with ORR 68% in MZL after anti-CD20 therapy.

**Rationale:**
CD20 loss renders rituximab ineffective. Flowchart (S2) recommends zanubrutinib as the first option for refractory disease after anti-CD20 therapy. No other anti-CD20-based therapies make sense. Zanubrutinib has evidence-based data in MZL relapse.

**===== run_2 =====**

**Tumor board decision:**
Systemic therapy with BTK inhibitor (Zanubrutinib) as the next step. Radiotherapy not indicated due to systemic disease (stage III).

**Rationale:**
CD20 loss renders anti-CD20 therapy ineffective. Systemic disease requires systemic therapy. BTK inhibitors show high response rate in R/R MZL after anti-CD20 as demonstrated in literature and ASH presentations. Radiotherapy only makes sense if the stage is localized.

**===== run_3 =====**

**Tumor board decision:**
Zanubrutinib as the next therapeutic step (BTK inhibitor, approved for relapsed/refractory MZL after anti-CD20 therapy).

**Rationale:**
PD under rituximab monotherapy with CD20 loss makes further anti-CD20-based therapies contraindicated. Flowchart (S2) recommends zanubrutinib as an approved option in refractory MZL after anti-CD20 therapy (MAGNOLIA study: ORR 68%). Plasmacellular differentiation confirms CD20 loss, therefore no rituximab escalation. Systemic therapy required (stage III).

## ========================================================== qwen3-next-80b (plain) ==========================================================

**===== run_1 =====**

**Tumor board decision:**
Start bendamustine monotherapy

**Rationale:**
CD20 loss renders rituximab-based therapies ineffective. Bendamustine is an established option for relapsed MZL after rituximab monotherapy. No other molecular options available.

**===== run_2 =====**

**Tumor board decision:**
Bendamustine monotherapy (90 mg/m2 D1-2 q4W) or CVP regimens. Anti-CD20 therapies are contraindicated.

**Rationale:**
CD20 loss after rituximab monotherapy renders anti-CD20-based therapies ineffective. Bendamustine or CVP are standard options for CD20-negative MZL with systemic spread.

**===== run_3 =====**

**Tumor board decision:**
Bendamustine monotherapy (160 mg/m2 day 1/2 every 4 weeks) as the next therapeutic step

**Rationale:**
CD20 loss precludes anti-CD20 therapies. Bendamustine is standard for relapsed MZL after rituximab monotherapy. No prior chemotherapy, therefore suitable. No molecular data for targeted therapies.

# ly_case_13__ALL

################################
#### CASE: 13
################################

## ========================================================= gpt-5-mini (agent) =========================================================

**===== run_1 =====**

**Tumor board decision:**
IIB with a mediastinal mass should be treated as advanced cHL. Recommendation: BrECADD (4-6 cycles) with interim PET after 2 cycles. For DS1-3, a total of 4 cycles; for DS4-5 extension to 6 cycles. Consolidative involved-site RT mediastinum 30 Gy only for PET-positive residual lesions. If BrECADD is contraindicated: consider BEACOPesk. (fit, fertility education) or alternatively 4x ABVD + 30 Gy.

**Rationale:**
Mediastinal mass corresponds to GHSG-RF -> treatment as advanced stage. Guidelines/HD21: BrECADD <=60 y. offers high PFS and better tolerability vs BEACOPesque.; RT only if there is a PET-positive residual. Recommend fertility advice/protection before alkylating therapy.

**===== run_2 =====**

**Tumor board decision:**
Recommendation: Systemic first line with BrECADD, PET-adaptive. Interim PET after 2 cycles: DS1-3 -> a total of 4 cycles; DS4-5 -> extend to 6 cycles or escalate according to protocol decision. Before therapy: fertility advice/preservation. Consolidative involved-site RT of the mediastinum only if residual PET findings are positive, goal: 30 Gy.

**Rationale:**
cHL Ann-Arbor IIB with a mediastinal mass is considered 'advanced' (bulk) according to the GHSG. HD21/Onkopedia: BrECADD <=60 J. Standard due to high PFS and better toxicity vs BEACOPPesk. RT only if there is a PET-positive residual (30 Gy IS-RT). Fertility protection due to alkylating agents/BrECADD recommended.

**===== run_3 =====**

**Tumor board decision:**
We recommend the PET-adapted GHSG scheme "2+2": 2 cycles BEACOPPesk -> interim PET. For DS 1-3: 2 cycles of ABVD, no IS-RT; for DS 4-5: Involved Site RT Mediastinum 30 Gy. If BEACOPP is not acceptable due to age/comorbidity or fertility desire: 4x ABVD + IS-RT 30 Gy. Before starting therapy: fertility advice, ECG/echo, lung function, PET/CT.

**Rationale:**
Stage IIB with mediastinal mass falls into the GHSG intermediate group. The PET-adapted 2+2 strategy (HD-17) offers very good PFS and enables RT to be avoided in the case of PET negativity. BEACOPPesk is more potent but significantly gonadotoxic; in ABVD, consolidative IS-RT (30 Gy) is recommended with residual PET.

## ========================================================== gpt-5-mini (plain) ==========================================================

**===== run_1 =====**

**Tumor board decision:**
Procedure: complete staging with FDG-PET/CT and basic laboratory (including fertility information). Therapy: PET-adapted combined therapy for stage IIBE - ABVD 2 cycles -> interim PET. If PET negative: additional 2 cycles of ABVD + ISRT (~20-30 Gy). If PET positive: Escalation e.g. eBEACOPP followed by ISRT.

**Rationale:**
Early unfavorable Hodgkin (IIBE) is treated as standard with chemo-radiotherapeutic, PET-guided approach. Interim PET allows de-escalation if the response is good and timely escalation if the response is inadequate; no pretreatments available.

**===== run_2 =====**

**Tumor board decision:**

Start of combined therapy: ABVD (Adriamycin, Bleomycin, Vinblastine, Dacarbazine) 2 cycles with interim PET (PET2). If PET2 negative: another 2 cycles of ABVD (4 in total) and subsequent involved-site RT (~30 Gy) for mediastinal mass. If PET2 positive: consider escalation (e.g. eBEACOPP) and RT.

**Rationale:**
Stage IIBE with mediastinal mass = unfavorable/bulky. Evidence-based, PET-adapted combined modality (chemo +/- escalation + RT) maximizes healing. Before starting therapy: fertility advice, ECG/echo, lung function, infection screening.

**===== run_3 =====**

**Tumor board decision:**
Consistent procedure: Baseline 18F-FDG-PET/CT plus basic cardiopulmonary diagnostics. Therapy: PET-guided ABVD chemotherapy (2 cycles -> interim PET; if PET is negative, another 2 cycles of ABVD) followed by involved-site radiotherapy 30 Gy to the mediastinal mass. If interim PET is positive, escalation according to GHSG (e.g. eBEACOPP).

**Rationale:**
Stage IIBE with mediastinal mass and B symptoms corresponds to early unfavorable Hodgkin lymphoma. Combined chemo-RT (ABVD PET-guided + ISRT 30 Gy) is standard with a high cure rate; Interim PET identifies patients who benefit from escalation (eBEACOPP).

## ============================================================ gpt-5-nano (agent) ============================================================

**===== run_1 =====**

**Tumor board decision:**
PET-adapted BEACOPPesk firstline for newly diagnosed cHL stage IIB-E (mediastinal). After two cycles of BEACOPPesk PET control: DS 1-3 -> 2 cycles of ABVD, no RT; DS 4 -> RT 30 Gy. A total of 4 cycles (DS 1-3) or 6 cycles (DS 4).

**Rationale:**
PET-adapted CHL-2/CHL-3 increases PFS/OS in advanced HL; RT reduction possible with negative DS; ABVD alone less effective in extranodal cases.

**===== run_2 =====**

**Tumor board decision:**
PET-adapted ABVD first-line therapy protocol. Start with 2 cycles of ABVD; interim PET according to Z2. DS 1-3: another 2 cycles of ABVD, no RT. DS 4-5: RT 30 Gy ISRT; If necessary, further cycles per PET result.

**Rationale:**
Intermediate HL IIB-E in fit patient; PET-adapted ABVD offers high cure with lower long-term toxicity than BEACOPP escalation. Interim PET enables de-escalation/intensification and targeted RT instead of blanket BEACOPP. If high-risk features occur, a BEACOPP strategy can be considered later.

**===== run_3 =====**

**Tumor board decision:**
Intermediate HL IIB with RF (bulky mediastinal). PET-adapted first line: 2x BEACOPPesc. + 2x ABVD + 30 Gy IS-RT. After 2 cycles of BEACOPPesc: PET DS 1-3 -> ABVD 2x, no RT; DS 4 -> RT 30 Gy. BEACOPEsk.-Contra: 4x ABVD + 30 Gy RT. Pay attention to fertility.

**Rationale:**
CHL-2/HD-15-16-PET adaptation; Balance of PFS and toxicity; PET-guided de-escalation avoids RT with negative DS. Alternative for BEACOPP-Contra: 4x ABVD + RT. Consider long-term toxicities (gonadotoxicity); Fertility advice accordingly.

## ============================================================ gpt-5-nano (plain) =============================================================

**===== run_1 =====**

**Tumor board decision:**
ABVD 2 cycles followed by ISRT (mediastinal field); PET-adapted: complete negative after 2 cycles of ISRT; positive after 2 cycles 4 cycles ABVD + ISRT.

**Rationale:**
Stage IIBE, no B symptoms, no previous treatment. Standard first-line: ABVD + ISRT; PET-guided adjustment reduces over-treatment and enables good chances of recovery.

**===== run_2 =====**

**Tumor board decision:**
ABVD x 4 cycles + ISRT mediastinally approx. 30 Gy. PET adaptation after 2 cycles to adjust the RT dose if necessary.

**Rationale:**
IIB/E with mediastinal bulk is considered unfavorable in the early stages. Standard is combined chemo + locoregional RT; no prior therapies or molecular targets available; PET adaptation enables individual adjustment.

**===== run_3 =====**

**Tumor board decision:**
ABVD x4 cycles followed by ISRT (mediastinal) as first-line therapy for stage IIB classical Hodgkin lymphoma; PET-CT for follow-up after cycles 2/4 optional.

**Rationale:**
Standard treatment for stage IIB without previous therapies; good healing success; mediastinal findings addressable by ISRT; acceptable toxico profile.

## ============================================================ gpt-oss-20b (agent) ============================================================

**===== run_1 =====**

**Tumor board decision:**
For classic HL, Ann Arbor IIB with mediastinal RF (RFc) and without previous treatment, we recommend BrECADD therapy: 4-6 cycles (PET after 2 cycles, DS1-3 -> 4 cycles, DS4-5 -> 6 cycles). For PET-positive residues, 30Gy RT (IS-RT).

**Rationale:**
StageIIBc corresponds to advanced HL (GHSGRS-2c). For patients <=60 years, BrECADD is the current standard option (HD21 study) with high PFS and better tolerability than BEACOPPesk. PET-adapted cycle adjustment and RT with positive residual are guideline-compliant.

**===== run_2 =====**

**Tumor board decision:**
For classic HL, Ann Arbor IIB with mediastinal RF (RFc), we recommend the PET-adapted BrECADD regimen as first-line therapy: 4 cycles (for DS1-3) or 6 cycles (for DS4-5) after 2 cycles. For PET-positive residues, 30Gy IS-RT. Alternatively, BEACOPPesque. if there are contraindications to BrECADD.

**Rationale:**
Flowchart III-C-3 defines IIBc/d as advanced stages. Standard is 4-6xBrECADD + RT for PET-positive residues. PET-adapted cycle optimization reduces stress. BEACOPPesque. remains as an alternative in case of intolerance. There are no secondary diagnoses or contraindications.

**===== run_3 =====**

**Tumor board decision:**
BrECADD 4-6 cycles (PET-adapted) + 30Gy RT for PET-positive residues.

**Rationale:**
StageIIB with RFc (mediastinal bulk) -> advanced classic HL. GHSG study HD21 defines BrECADD as the standard for patients <=60 years old. PET control after 2 cycles controls the number of cycles (4 for PET-neg, 6 for PET-pos). RT 30Gy with positive residual.

## ============================================================ gpt-oss-20b (plain) ============================================================

**===== run_1 =====**

**Tumor board decision:**
Start ABVD (Adriamycin, Bleomycin, Vinblastine, Dacarbazine) 6x28days, with interim PET after 2 cycles. If PET is positive, consider BEACOPP-ES if necessary.

**Rationale:**
Classic HL, Ann Arbor IIBE, no pre-existing conditions. ABVD is standard for early, bulky HL. Interim PET controls therapy intensity.

**===== run_2 =====**

**Tumor board decision:**
Start ABVD chemotherapy (Adriamycin, Bleomycin, Vinblastine, Dacarbazine) for 6 cycles. After 2 cycles of PET-CT, adjustment if necessary (e.g. continuation if PET is positive).

**Rationale:**
Classic HL, Ann Arbor IIB, no previous treatment, standard therapy ABVD at intermediate risk. PET after 2 cycles is used to adjust therapy and determine prognosis.

**===== run_3 =====**

**Tumor board decision:**
Start with ABVD (Adriamycin, Bleomycin, Vinblastine, Dacarbazine) for 6 cycles; Interim PET/CT after 2 cycles to assess response and adjust therapy if necessary.

**Rationale:**
ABVD is the established first-line treatment for classical Hodgkin lymphoma stage IIB E with proven effectiveness and an acceptable balance of side effects; an interim PET controls an individual therapy adjustment.

## ============================================================ gpt-oss-120b (agent) ============================================================

**===== run_1 =====**

**Tumor board decision:**
In classic HL, stage IIBE with mediastinal bulk (RFc), we recommend a PET-adaptable BrECADD regimen (4-6 cycles) for patients <=60 years. After 2 cycles of interim PET: if PET-negative, no RT after 4 cycles, if PET-positive or residual mass IS-RT30Gy. Cardiopulmonary, fertility and laboratory clarification before therapy.

**Rationale:**
The mediastinal bulk classifies the stage as advanced (IIBRFc). According to the GHSG guidelines, BrECADD is the current standard regimen for advanced cHL in younger patients, offers high 3-year PFS and lower gonadotoxicity compared to BEACOPP. PET-adapted intensity enables therapy de-escalation and targeted radiation therapy for residual nodules.

**===== run_2 =====**

**Tumor board decision:**
Start BrECADD therapy (brentuximab vedotin+etoposide+cyclophosphamide+doxorubicin+dacarbazine+dexamethasone) for 4-6 cycles. After cycle 2, an interim PET: for DS1-3 a total of 4 cycles, for DS4-5 6 cycles. Subsequently Involved-Site-RT30Gy for PET-positive residual foci. Before therapy, ECG, echo, lung function and fertility counseling.

**Rationale:**
Stage IIB with mediastinal bulky (RFc) is considered advanced cHL according to GHSG. According to the current guidelines, BrECADD is the standard for patients <=60 years of age and offers high effectiveness and better tolerability compared to BEACOPP. PET-adapted intensity adjustment minimizes over- or under-treatment.

**===== run_3 =====**

**Tumor board decision:**
First-line therapy for advanced cHL (IIB-c) at <=60 years: 4-6 cycles of BrECADD (brentuximab-vedotin+etoposide+cyclophosphamide+doxorubicin+dacarbazine+dexamethasone) with interim PET after 2 cycles; for PET-negative 4 cycles, for PET-positive 6 cycles, then 30Gy IS-RT of the mediastinal bulk.

**Rationale:**
Stage IIB with mediastinal bulky (RFc) is considered advanced HL according to GHSG. BrECADD is the current standard for patients <=60y, offering high PFS and lower gonadotoxicity compared to BEACOPP. PET-adapted intensification and final IS-RT ensure local control of the bulky.

## ============================================================ gpt-oss-120b (plain) ============================================================

**===== run_1 =====**

**Tumor board decision:**
Start of ABVD chemotherapy (doxorubicin, bleomycin, vinblastine, dacarbazine) for 2-4 cycles, followed by PET control. If PET is negative, further 2-4 ABVD cycles and local involved-field radiotherapy (20-30Gy) because of initial mediastinal bulky lesion. If PET is positive, switch to escalated BEACOPP or intensive radiotherapy.

**Rationale:**
Classical Hodgkin lymphoma, stage IIEB with mediastinal bulky component. Standard therapy is ABVD followed by PET-adapted decision; Local radiotherapy improves the local control rate in bulky disease. No previous illnesses or previous treatments, therefore no contraindications.

**===== run_2 =====**

**Tumor board decision:**
Start ABVD-based therapy (Adriamycin, Bleomycin, Vinblastine, Dacarbazine) for 2 cycles, followed by interim PET monitoring. If the PET result is negative, an additional 2-4 ABVD cycles; If the PET result is positive, radiotherapy or intensive BEACOPP escalation. For mediastinal bulky lesions, subsequent involved field radiotherapy (IFRT) after 4-6 cycles of ABVD is recommended.

**Rationale:**
Classic Hodgkin lymphoma stage IIEB with mediastinal bulky lesion requires combined therapy. ABVD is standard first line; Interim PET control enables therapy intensity adjustment and reduces over- or undertreatment.

**===== run_3 =====**

**Tumor board decision:**
Start PET-adapted ABVD therapy (2 cycles ABVD, interim PET). If PET is negative, continue 4 additional cycles of ABVD (6 cycles total) and then give involved-site radiotherapy (20-30Gy) for the mediastinal bulky lesion. If interim PET is positive, switch to escalated BEACOPP or combine BEACOPP with radiotherapy.

**Rationale:**
Classical Hodgkin's lymphoma stage IIE-B with mediastinal bulky requires systemic chemotherapy plus local radiation therapy. ABVD is standard first-line; The PET-adapted concept enables therapy intensity to be optimized according to response and to avoid over- or under-treatment.

## ============================================================ qwen3-32b (agent) ============================================================

**===== run_1 =====**

**Tumor board decision:**
Recommended therapy: 4-6 cycles of BrECADD (brentuximab vedotin, etoposide, cyclophosphamide, doxorubicin, dacarbazine, dexamethasone) with PET-adapted number of cycles (after 2 cycles of PET control). If there is residual PET-positive findings after chemotherapy: 30 Gy involved-field RT. Discuss fertility preservation before starting therapy.

**Rationale:**
Stage IIBE with large mediastinal tumor burden (GHSG risk factor c) falls into the advanced stages. BrECADD (HD21 study) is new standard with better tolerability vs. BEACOPPesk. PET-adapted therapy after 2 cycles optimizes PFS. RT with PET-positive residual. Fertility preservation due to high gonadal toxicity of BrECADD.

**===== run_2 =====**

**Tumor board decision:**
Recommended therapy: 2x BEACOPPesque. + 2x ABVD + 30 Gy IF-RT. Alternatively: 4x ABVD + 30 Gy IS-RT if intolerance.

**Rationale:**
Stage IIBE (intermediate) according to GHSG classification. BEACOPPesque. + ABVD + RT is standard therapy with best PFS. PET-adapted adjustment possible after cycle 2.

**===== run_3 =====**

**Tumor board decision:**
Recommended therapy: 2x BEACOPP escalated -> PET control. If PET negative: 2x ABVD without RT. For PET positive (DS4): 2x ABVD + 30 Gy IS-RT. Discuss fertility preservation before BEACOPP.

**Rationale:**
Stage IIBE is one of the intermediate stages. BEACOPPesque. + PET-adapted RT (HD-17 study) is standard. Mediastinal involvement requires IS-RT if PET positive. BEACOPP has higher toxicity, fertility preservation is critical.

## ============================================================ qwen3-32b (plain) ============================================================

**===== run_1 =====**

**Tumor board decision:**
Recommendation: ABVD chemotherapy (4-6 cycles) followed by involved-field radiotherapy (30-36 Gy) for residual mediastinal tumor.

**Rationale:**
Classic Hodgkin's lymphoma stage IIBE with mediastinal tumor burden requires combined chemotherapy and radiation therapy. ABVD is standard chemotherapy with high response rates. After complete remission, locoregional radiation is applied to eradicate residual tumor cells.

**===== run_2 =====**

**Tumor board decision:**
A 4-6 course of chemotherapy with ABVD (Adriamycin, Bleomycin, Vinblastine, Dacarbazine) is recommended, followed by radiation therapy to the mediastinal lymph node area (involved-field radiotherapy).

**Rationale:**
Classic Hodgkin's lymphoma stage IIBE with mediastinal involvement requires combined chemoradiotherapy. ABVD is standard chemotherapy with high response rates. After chemotherapeutic response, local irradiation is indicated to eradicate remaining cells.

**===== run_3 =====**

**Tumor board decision:**
A 4-6 cycle ABVD chemotherapy (doxorubicin, bleomycin, vinblastine, dacarbazine) followed by affected field radiation therapy (IFRT) of the mediastinal lymph nodes is recommended.

**Rationale:**
For classical Hodgkin's lymphoma stage IIBE, the combination of ABVD chemistry and IFRT is the standard therapy. The lack of prognostic factors and comorbidities argue for standard-compliant treatment without premature dose reduction.

## =========================================================== qwen3-next-80b (agent) ===========================================================

**===== run_1 =====**

**Tumor board decision:**
4-6 cycles of BrECADD with interim PET/CT after 2 cycles to adjust the number of cycles (DS 1-3: 4 cycles; DS 4-5: 6 cycles) as well as localized radiation therapy (30 Gy) for PET-positive residual lesions.

**Rationale:**
Stage IIB with large mediastinal tumor (RF c) is considered an advanced stage. According to the HD21 study, BrECADD is standard therapy for cHL <=60 years with better tolerability than BEACOPPesk. PET-adapted therapy optimizes treatment.

**===== run_2 =====**

**Tumor board decision:**
4-6 cycles of BrECADD with PET adaptation after 2 cycles (DS 1-3 -> 4 cycles, DS 4-5 -> 6 cycles) followed by 30 Gy IS-RT for PET-positive residual lesions

**Rationale:**
Stage IIB with mediastinal involvement (RF c) is considered an advanced stage. According to the HD21 study, BrECADD is standard for <=60 year olds with better tolerability and PFS vs. BEACOPPesk. PET

adaptation optimizes therapy intensity. No contraindications stated.

**===== run_3 =====**

**Tumor board decision:**
BrECADD regimen (4-6 cycles, PET adapted) followed by 30 Gy RT for PET positive residuals. Age must be confirmed to switch to BV-AVD if >60 years.

**Rationale:**
Stage IIB with mediastinal involvement (RF c) is advanced cHL. BrECADD is standard for <=60 year olds (HD21 study). PET after 2 cycles determines cycle number (DS 1-3: 4 cycles; DS 4-5: 6 cycles). RT for PET-positive residues. Age is crucial as >60 years requires BV-AVD.

## ================================================================ qwen3-next-80b (plain) ================================================================

**===== run_1 =====**

**Tumor board decision:**
Start ABVD chemotherapy (doxorubicin, bleomycin, vinblastine, dacarbazine) for 4 cycles followed by affected field radiotherapy (ISRT).

**Rationale:**
Classic Hodgkin lymphoma stage IIE with mediastinal involvement is considered an unfavorable risk factor. Standard therapy is 4 cycles of ABVD followed by ISRT. No prior therapy, therefore first line. No contraindications mentioned.

**===== run_2 =====**

**Tumor board decision:**
Initiate ABVD chemotherapy (4 cycles) followed by affected field radiotherapy (ISRT).

**Rationale:**
Standard therapy for classical Hodgkin lymphoma stage IIE with mediastinal involvement (bulky disease). ABVD is the first choice for fit patients without contraindications. ISRT after chemotherapy reduces the risk of recurrence. NCCN guidelines confirm this approach.

**===== run_3 =====**

**Tumor board decision:**
Initiate ABVD chemotherapy (4 cycles) followed by affected field radiotherapy (ISRT) to mediastinal lymphoma.

**Rationale:**
Standard therapy for unfavorable early Hodgkin lymphoma with mediastinal involvement. ABVD is the first choice due to favorable toxicity profiles. ISRT after chemo for local control. No contraindications or comorbidities mentioned.

# ly_case_14__ALL

################################
#### CASE: 14
################################

## ================================================================ gpt-5-mini (agent) ================================================================

**===== run_1 =====**

**Tumor board decision:**
After ISRT has been completed, we recommend: 1) First restaging primarily using EGD with multiple staged biopsies 3 months after RT (alternatively 3-6 months). 2) No routine PET-CT and no routine bone marrow biopsy. 3) Laboratory (BB, LDH, liver/kidney), HCV/HBV/HIV if necessary. 4) Follow-up: EGD+Histo every 3-6 months in the first 2 years, then every 6-12-18 months; Laboratory checks every 3-6 months. 5) Systemic therapy only if progression is confirmed (endoscopic PD + Wotherspoon 5 / symptomatic or disseminated disease). t(11;18) test if not carried out.

**Rationale:**
Gastric MALT responds late to RT (CR to 15 months); Guidelines recommend W&W and endoscopic control every 3-6 months. PET/CT has low FDG avidity; Contrast biopsy not routine. Systemic therapy only in the case of documented progression or symptomatic/systemic disease (R-bendamustine/rituximab etc.).

**===== run_2 =====**

**Tumor board decision:**
No immediate PET-CT or bone marrow biopsy. First restaging: EGD with multiple staged biopsies 3-4 months after completion of radiation, then EGD+histology every 3-6 months for 2 years, then interval extension to 6-12 months (up to >5 years, if necessary annually). Laboratory controls (BB, LDH, beta2 microglobulin, liver/kidneys) at the same time. CT only if symptoms/clinical suspicion. Supplementary systemic therapy only in cases of confirmed progression (endoscopically + histologically, e.g. Wotherspoon 5/PD), symptomatic or disseminated disease; Preferred first-line therapies: rituximab-bendamustine or rituximab monotherapy.

**Rationale:**
Response to ISRT can only be confidently assessed after 3-6 (up to 15) months; Avoid premature escalation. PET/CT is not routinely helpful in gastric MALT due to low FDG avidity; Bone marrow biopsy not required as standard. Guidelines/SOPs recommend endoscopic-histological checks at the specified intervals and systemic therapy only in cases of documented progression or symptomatic/spread disease.

**===== run_3 =====**

**Tumor board decision:**
No immediate switch to systemic therapy. Restaging: EGD with staged biopsies 3-6 months after completion of RT; Basic laboratory (BB, LDH, liver/kidney, HBV/HCV/HIV). PET-CT and bone marrow biopsy not routine. Follow-up: EGD+biopsies every 3-6 months until CR (up to 12-24 months), then every 6-12-18 months. Systemic therapy (e.g. rituximab+/-bendamustine) only in the case of histologically/radiologically confirmed progression or symptomatic findings; Consider study participation.

**Rationale:**
ISRT leads to high remission rates, CR may not occur for 3-6 (up to 15) months - avoid premature escalation. PET has low sensitivity for MALT; Contrast biopsy is not routinely indicated. Systemic therapy only if progression or symptoms are documented according to guidelines/flowchart.

## ================================================================ gpt-5-mini (plain) ================================================================

**===== run_1 =====**

**Tumor board decision:**
Endoscopic restaging (EGD with multiple biopsies) 3 months after radiation. In case of complete remission: close observation (EGD + biopsies every 6-12 months for 2-3 years, then annually up to 5 years). In the case of residual/progression: t(11;18) clarification and systemic therapy (rituximab +/- chemotherapy, e.g. R-bendamustine). No further radiation.

**Rationale:**
In localized MALT (stage IEA), H.p. eradication + 30 Gy RT is curatively possible. First, response documentation via EGD/biopsy. For CR, no further therapy; in the case of persistence/progression or t(11;18)+, systemic therapy is indicated. RT has already been given, so do not repeat.

**===== run_2 =====**

**Tumor board decision:**
Observation after completed radiation therapy (30 Gy): EGD with biopsies in 3 months, then every 6 months for 2 years, then annually. CT if clinical symptoms or findings are suspected. No immediate systemic therapy. In case of residual/progression: consider rituximab monotherapy or rituximab + bendamustine.

**Rationale:**
In localized gastric MALT (stage IEA), 30 Gy radiation often leads to CR. H.p. has already been eradicated and is negative. Therefore primary surveillance with endoscopic control; systemic therapy only if persistence/progression is proven.

**===== run_3 =====**

**Tumor board decision:**
EGD with targeted biopsies and H.p. test 3-6 months after radiation for restaging; In the case of complete remission, conservative follow-up care (EGD every 6 months in the 1st year, then annually, CT check if necessary). In the case of persistent/recurrent local or progressive findings, systemic therapy with anti-CD20 (rituximab +/- chemotherapy, e.g. bendamustine-R) after interdisciplinary consideration.

**Rationale:**
Stage IEA MALT of the stomach was treated locally with 30 Gy after H.p. eradication. Response to RT may be delayed; The first goal is histological restaging. With CR, surveillance is sufficient. In the case of residual/progression, systemic anti-CD20-based therapy is the standard option.

## ============================================================ gpt-5-nano (agent) ============================================================

**===== run_1 =====**

**Tumor board decision:**
Watch-and-wait with close endoscopy (stage biopsies every 3-6 months) and imaging control every 6-12 months; Observation until 24 months after RT. In case of progression or symptomatic residual involvement: systemic rituximab-based therapy (e.g. R-bendamustine or rituximab monotherapy).

**Rationale:**
Indolent course of gastric MALT; HP eradication+RT provides local control. Residual findings may slowly regress; No immediate systemic therapy necessary, close monitoring with prompt change in therapy in the event of progression.

**===== run_2 =====**

**Tumor board decision:**
Observation (watch-and-wait) of residual gastric MALT after HP eradication + RT 30 Gy. Endoscopy with staged biopsies every 3-6 months; Abdominal/thorax imaging every 6-12 months. No immediate systemic therapy. In case of progression/symptoms, rituximab-based therapy (e.g. R-bendamustine) or study options.

**Rationale:**
Indolent course of MALT lymphomas; RT allows good local control. Residual lesions can be observed for up to 24 months; Early systemic therapy often provides no benefit and increases toxicity. Progression or symptoms -> systemic rituximab-based therapy.

**===== run_3 =====**

**Tumor board decision:**
Recommend continuing a watch-and-wait approach after RT 30 Gy: close endoscopy with staged biopsies every 3-6 months until 24 months post-RT. In case of progression or symptomatic worsening, systemic rituximab-based therapy (e.g. R-bendamustine or R-chlorambucil) or, if necessary, BTK inhibitors; further local therapy only if there is clear progression.

**Rationale:**

After HP eradication+RT, Gastric MALT often achieves rapid remission; residual tissue CR occurs delayed. W&W guideline-compliant up to 24 months; Systemic therapy only when progression/symptoms minimize toxicity. HP eradication already done; local treatment only in case of progression.

## ========================================================== gpt-5-nano (plain) ==========================================================

**===== run_1 =====**

**Tumor board decision:**
Gastric MALT lymphoma (IEA) persisting after HP eradication. RT 30 Gy completed. Further procedure: Endoscopic follow-up with biopsies 8-12 weeks post-RT. In case of complete remission: Surveillance. In case of residual/progression: systemic rituximab-based therapy (e.g. rituximab +/- bendamustine).

**Rationale:**
RT is standard for local gastric MALT after HP eradication; Remission status determines further therapy. Residual findings -> systemic therapy makes sense; no accessible targeted local treatment.

**===== run_2 =====**

**Tumor board decision:**
Systemic rituximab-based immunochemotherapy for persistent gastric MALT lymphoma after RT. Preferably BR (bendamustine+rituximab); alternative rituximab monotherapy depending on tolerability.

**Rationale:**
RT completed; HP eradication occurs; persistent disease. In MALT lymphoma persistence/relapse, BR or RTX monotherapy are established options with good efficacy.

**===== run_3 =====**

**Tumor board decision:**
Restaging after radiotherapy (30 Gy): Endoscopy with biopsies and CT to assess response. If CR achieved: Observation. If persistent or partial response: systemic rituximab-based therapy (preference BR: bendamustine + rituximab; alternatives: rituximab monotherapy or R-CHOP depending on resilience).

**Rationale:**
Stage IEA, local gastric MALT, HP negative; HP eradication occurs; RT 30 Gy standard for localized MALT/MZL. No disseminated disease; FISH negative. BR therapy works well in MALT/MZL with acceptable toxic profile; Aim for CR, otherwise further systemic therapy.

## ========================================================== gpt-oss-20b (agent) ==========================================================

**===== run_1 =====**

**Tumor board decision:**
Discontinue radiotherapy, watch and wait with endoscopic control every 3-6 months; no further local or systemic therapy required.

**Rationale:**
Stage IEA, HP negative, no progression after 30Gy RT. Flowchart A1.3: with asymptomatic remnants after RT observation. No indication for further RT or systemic therapy.

**===== run_2 =====**

**Tumor board decision:**
No further therapy; Observation with EGD control every 3-6 months (after completing 30 Gy RT).

**Rationale:**
For HP-negative, localized MALT lymphoma (Lugano I) and after low-dose RT, the standard option is watch and wait. Further RT or systemic therapy only in case of progression or symptomatic recurrence.

**===== run_3 =====**

**Tumor board decision:**
After completion of 30Gy ISRT, no further local or systemic therapy will be initiated. Instead, watch and wait with endoscopic control every 3-6 months (up to 2 years) is recommended. In the event of progression (Wotherspoon>=5), radiotherapy or systemic therapy is given.

**Rationale:**
Flowchart A1.3: In the case of HP-negative MALT with residual symptoms after RT, observation is initially recommended. RT dose 24-30Gy is curative; Response only assessed after 3-6 months. No premature escalation to systemic therapy.

## ========================================================= gpt-oss-20b (plain) =========================================================

**===== run_1 =====**

**Tumor board decision:**
After completion of 30Gy radiotherapy and negative H. pylori status, we first recommend observation: 6-month EGD with biopsy. If there is persistence or progression, rituximab monotherapy (375 mg/m2, 4-6 cycles) is used.

**Rationale:**
Stage IEA, no comorbidities, H. pylori negative -> radiotherapy is adjuvant. Observation corresponds to guidelines; Rituximab is the preferred therapy for relapse.

**===== run_2 =====**

**Tumor board decision:**
After 30Gy low-dose radiotherapy for localized MALT lymphoma (stage IEA) without evidence of residual: observation with regular control (OGD, CT) - in case of relapse, rituximab monotherapy or renewed radiotherapy.

**Rationale:**
30Gy is considered curative for MALT lymphoma. No evidence of residual, no complications, no molecular markers for targeted therapy. Observation minimizes side effects and enables early intervention in the event of a recurrence.

**===== run_3 =====**

**Tumor board decision:**
First, a repeat EGD with biopsy to assess the response to therapy. If MALT lymphoma persists, monotherapeutic rituximab treatment (375 mg/m2 i.v. 4W) is recommended; If there is remission, observation takes place.

**Rationale:**
Stage IEA, 30Gy radiotherapy is standard, but remission not confirmed. Rituximab is effective, minimally invasive and well tolerated in recurrent or unresolved MALT lymphoma.

## ========================================================= gpt-oss-120b (agent) =========================================================

**===== run_1 =====**

**Tumor board decision:**
After 30Gy low-dose radiotherapy for H. pylori-negative MALT lymphoma (stage IEA), a watch-and-wait approach is used. Control EGD with biopsies in 3-6 months, then every 6 months to 2 years, then annually. If the lesion persists or progresses, systemic therapy (rituximab+bendamustine) or, if necessary, renewed ISRT as a salvage.

**Rationale:**
Radiotherapy achieves >90% remission in local MALT lymphoma stages I-IIA. In the absence of clinical symptoms and after a full dose, observation is recommended; Only if there is no remission or progression is further therapy indicated.

**===== run_2 =====**

**Tumor board decision:**
Observation with endoscopic control examination (EGD+biopsies) in 3-6 months, then 6-12 months up to 2 years, then annually; If persistent MALT lymphoma is detected, consider further therapy (e.g. renewed ISRT or rituximab-bendamustine).

**Rationale:**
After successful low-dose radiotherapy (30Gy), a watch-and-wait approach is standard for H. pylori-negative, localized stage IEA. Guidelines recommend observation if response or stable persistence; Further local or systemic therapy only if residual/progress is documented.

**===== run_3 =====**

**Tumor board decision:**
Further management: observation (watch and wait) with endoscopic control (EGD + biopsies) in 3-6 months, then annually. If residual or progressive tumor is detected, repeat ISRT or systemic therapy (e.g. rituximab+bendamustine).

**Rationale:**
The patient has already received 30Gy low-dose radiotherapy for H. pylori-negative MALT lymphoma (stage IEA). According to the guidelines, ISRT leads to high remission rates in local disease; If there is clinical remission, further therapy is not indicated. Observation is standard because recurrences are rare and can be treated again if they occur.

## ============================================================ gpt-oss-120b (plain) ============================================================

**===== run_1 =====**

**Tumor board decision:**
After H.p. eradication and local radiotherapy (30Gy), no further therapy is necessary for stage I-EA MALT lymphoma of the stomach. Recommendation: close follow-up with EGD and biopsy in 3-6 months, then annual check-ups. If residual or recurrent tumors are detected, systemic therapy (rituximab-based) should be considered.

**Rationale:**
Stage IEA is localized; Radiotherapy with 30Gy is curative. H.p. negativity precludes further eradication. In the absence of evidence of persistence, observation is the standard evidence-based follow-up care.

**===== run_2 =====**

**Tumor board decision:**
Further procedure: observation with endoscopic control (OGD) every 3-6 months and imaging follow-up; no immediate therapy. If residual or recurrent lesions are detected, consider renewed low-dose radiotherapy (30Gy) or systemic therapy (e.g. rituximab).

**Rationale:**
MALT lymphoma is stage IEA, localized lesion, H.p. negative. After successful low-dose radiotherapy (30Gy), the local control rate is >90% with low toxicity. If there is complete remission, no further therapy is necessary; Close follow-up care enables early detection of a recurrence.

**===== run_3 =====**

**Tumor board decision:**
After successful low-dose radiotherapy (30Gy) and no signs of residual or progressive disease, no further therapy is initiated. Recommendation: close follow-up with EGD + biopsy and, if necessary, CT/MRI at intervals of 3-6 months, then annual check-ups.

**Rationale:**
MALT lymphoma stage IEA is in remission after local radiotherapy. H. pylori negativity and lack of systemic manifestations argue against additional therapy. Consistent observation enables early detection of recurrences.

## ============================================================ qwen3-32b (agent) ============================================================

**===== run_1 =====**

**Tumor board decision:**
After low-dose radiotherapy (30 Gy) for H.p.-negative MALT lymphoma stage IEA, the following is recommended: watch-and-wait with endoscopic control (EGD with staged biopsies) every 3-6 months. Escalation to systemic therapy only if progression is confirmed (Wotherspoon 5 + endoscopic PD).

**Rationale:**
Radiotherapy is established for localized MALT lymphomas. In the case of the H.p.-negative form and after radiation, observation with close endoscopy is standard. Early systemic therapy is not indicated as remission rates after RT can last up to 15 months.

**===== run_2 =====**

**Tumor board decision:**
After low-dose radiotherapy (30 Gy) for H.p.-negative MALT lymphoma stage IEA: watch-and-wait with EGD biopsies every 3-6 months. Escalation to systemic therapy only if progression is confirmed after 3-6 months.

**Rationale:**
The guidelines recommend observation with endoscopic controls after radiotherapy. Premature escalation is contraindicated as remission periods of up to 15 months are possible. Systemic therapy (e.g. R-bendamustine) only if progression is proven.

**===== run_3 =====**

**Tumor board decision:**
Observation (watch and wait) with gastroscopic checks every 3-6 months for 2 years, followed by annual checks, is recommended. Systemic therapy only if progression is confirmed after 3-6 months.

**Rationale:**
According to guidelines, observation with close endoscopic controls is indicated after low-dose radiotherapy (30 Gy) for H.p.-negative MALT lymphoma stage IEA. The remission phase can last up to 15 months. Escalation to systemic therapy only if progression is demonstrated after 3-6 months.

## ============================================================ qwen3-32b (plain) ============================================================

**===== run_1 =====**

**Tumor board decision:**
Recommendation: After successful H.p. eradication and local radiotherapy (30 Gy), stage IEA MALT lymphoma of the stomach requires close follow-up with EGD and CT every 6 months for 2 years, then annually. No further therapies required.

**Rationale:**
The combination of H.p. eradication and local radiation is the established standard for H.p.-negative MALT lymphomas of the stomach. If there is a complete response and no progression, further therapy is not indicated. Follow-up care serves to detect recurrences early.

**===== run_2 =====**

**Tumor board decision:**
Recommendation: After eradication and radiotherapy (30 Gy), surveillance with EGD and CT H/T/Abd. every 6-12 months. No further therapies required.

**Rationale:**
Stage IEA MALT lymphoma (H.p. negative) requires no further interventions after successful radiotherapy. Surveillance is standard because the risk of recurrence is low and the response to therapy is good. No progression or contraindications known.

**===== run_3 =====**

**Tumor board decision:**
Active surveillance with regular checks (endoscopy, CT) after radiation therapy has been completed. No further therapies required.

**Rationale:**
H.p.-negative MALT lymphoma stage IEA showed complete regression after radiotherapy. No progression or residual disease detected. Consensual surveillance is standard.

## =========================================================== qwen3-next-80b (agent) ===========================================================

**===== run_1 =====**

**Tumor board decision:**
Further procedure: Observation with EGD control every 3-6 months. No immediate systemic therapy. Response assessment at the earliest after 3-6 months after radiotherapy.

**Rationale:**
After Lugano staging IEA and HP-negative MALT lymphoma with radiation therapy (30 Gy), response assessment is only possible after 3-6 months at the earliest. RT notes (RT5) emphasize: No premature escalation to systemic therapy, histological residues can be observed (W&W up to 24 months). Systemic

therapy only if progression is confirmed.

**===== run_2 =====**

**Tumor board decision:**
Regular endoscopy checks with staged biopsies every 3-6 months to assess treatment response. Systemic therapy only if progression is confirmed (Wotherspoon 5 + endoscopic PD) or symptomatic progression. No premature escalation.

**Rationale:**
After RT (30 Gy) for gastric MALT lymphoma, response assessment is only possible 3-6 months after completion (Flowchart RT5). Median CR time up to 15 months. Observation (W&W) with EGD controls is standard. Systemic therapy only if progression is documented, do not escalate prematurely.

**===== run_3 =====**

**Tumor board decision:**
Observation with regular EGD checks every 3-6 months. Systemic therapy only if progression is confirmed (Wotherspoon 5 + endoscopic PD).

**Rationale:**
After ISRT (30 Gy) for gastric MALT, response assessment is only possible after 3-6 months (RT5). Avoid early escalation to systemic therapy. Histological residues can remain stable for up to 24 months (EGILS/Fischbach). No extragastric involvement, therefore no indication for systemic therapy without progression.

## ============================================================ qwen3-next-80b (plain) ============================================================

**===== run_1 =====**

**Tumor board decision:**
Carry out response assessment by EGD and CT. In the case of complete remission, surveillance; in the case of persistent tumor, systemic therapy with rituximab.

**Rationale:**
Radiotherapy (30 Gy) is standard for H.p.-negative MALT lymphoma. After completion of radiotherapy, response assessment is required. If the tumor persists, rituximab is indicated as the next step because H.p. eradication was ineffective and no other options are available.

**===== run_2 =====**

**Tumor board decision:**
Response assessment by EGD with biopsy and CT abdomen. In case of remission: regular checks; for persistent lymphoma: rituximab monotherapy.

**Rationale:**
Radiation therapy (30 Gy) was given after H.p.-negative MALT lymphoma. The standard procedure is response assessment by EGD and CT. In case of remission: surveillance; for persistent lymphoma: rituximab monotherapy according to ESMO guidelines.

**===== run_3 =====**

**Tumor board decision:**
Endoscopic control with biopsy and CT to assess treatment response. For persistent lymphoma, rituximab monotherapy.

**Rationale:**
H.p.-negative MALT lymphoma IEA after radiotherapy requires response check. Rituximab for residual disease according to guidelines. Eradication was not indicated.

# ly_case_15__ALL

################################
#### CASE: 15
################################

============================================================ gpt-5-mini (agent) ============================================================

===== run_1 =====

**Tumor board decision:**
Primarily recommend CAR-T therapy if the patient is CAR-T capable. Before: re-biopsy and complete restaging (PET/CT, laboratory, cardiac assessments) and rapid presentation to the CAR-T center. If necessary, bridging therapy (Tumor board decision). If CAR-T is not possible: platinum-containing salvage (e.g. R-DHAP/ICE) with interim PET and for CR/PR high-dose chemotherapy + auto-SCT. Consolidative radiotherapy only in the case of residual mediastinal bulk/symptomatic local findings.

**Rationale:**
Early recurrence <=12 months after R-CHOP: Guidelines/flowchart prefer CAR-T if CAR-T is suitable (better results than ASCT in this situation). Re-biopsy/restaging are mandatory. If CAR-T is not feasible, classic platinum-containing salvage with the aim of HD-SZT is the established alternative; Use radiation restrictively.

===== run_2 =====

**Tumor board decision:**
Priority clarification and rapid implementation of CAR T-cell therapy (2nd line), as recurrence occurs within 12 months after R-CHOP. Before therapy: re-biopsy, complete restaging (PET/CT), cardiological and infectious clarification (HBV/HCV/HIV). Bridging only individually; in case of progression under R-RHAP, no repeating of the same platinum-containing regimen. Local radiation only in the case of symptomatic mediastinal compression or as a palliative measure.

**Rationale:**
Early relapse <=12 months: randomized studies and current SOP/flowchart prefer CAR-T over platinum salvage+autoSCT. Patient received 1 cycle of R-RHAP (salvage started) - if suitable for CAR-T, primary CAR-T. High-dose HD/autoSCT only remains an alternative if CAR-T is not possible; RT only in case of local urgency.

===== run_3 =====

**Tumor board decision:**
Re-biopsy and complete restaging; immediate check of CAR-T suitability. If CAR-T is suitable: primary CAR-T therapy with suitable bridging (lymphoma Tumor board decision); In the case of symptomatic mediastinal bulk, local radiation can be used as a bridging/cytoreductive measure. If not CAR-T compatible: continue platinum-containing salvage (e.g. R-DHAP/R-ICE) and for CR/PR high-dose chemotherapy + autologous stem cell transplant.

**Rationale:**
According to the SOP/flowchart, early recurrence (<=12 months after R-CHOP) is considered an indication for primary CAR-T if CAR-T is suitable; Salvage + autoSCT is primarily used for late recurrences (>12 months). Re-biopsy/restaging are mandatory for backup and therapy planning. A one-time R-RHAP cycle is not sufficient as a reason for automatic continuation to autoSZT.

============================================================ gpt-5-mini (plain) ============================================================

===== run_1 =====

**Tumor board decision:**
Continuation of salvage therapy: Completion of a total of 2-3 cycles of R-DHAP/R-ICE (the started R-RHAP can be completed), then PET-CT restaging, stem cell mobilization and, if there is a chemosensitive response, high-dose therapy with autologous stem cell transplantation (ASCT). In case of lack of response or transplant ineligibility, early evaluation for CAR-T or clinical trial.

**Rationale:**
Relapse 12 months after R-CHOP: the established standard for transplant-eligible patients is salvage chemotherapy followed by ASCT if there is a chemosensitive response. PET-controlled approach allows

decision; If the course is refractory or if ASCT is not suitable, CAR-T or studies are the next option.

**===== run_2 =====**

**Tumor board decision:**
Continuation of salvage therapy: a total of 2-3 cycles (e.g. R-DHAP or R-ICE; currently 1 cycle of R-RHAP already given), followed by PET-CT. In case of chemosensitive response: stem cell mobilization and high-dose therapy with autologous stem cell transplantation (ASCT). For chemoresistance/ASCT failure: CAR T cell therapy or clinical trial.

**Rationale:**
Relapse after R-CHOP within ~12 months; The curative standard for transplantable patients is salvage chemotherapy followed by ASCT if there is a response. Only 1 salvage cycle occurs, therefore continuation and re-staging. CAR-T for refractory or post-ASCT relapses; Consider RT for residual mediastinal bulk.

**===== run_3 =====**

**Tumor board decision:**
Continue R-RHAP for a total of 2-3 cycles and then restaging PET/CT. In case of chemosensitivity: stem cell mobilization and high-dose therapy with autologous stem cell transplantation (BEAM). If there is no response or transplant ineligibility: rapid evaluation and referral for CD19 CAR T therapy. Confirm recurrence histologically and check transplant fitness.

**Rationale:**
Early recurrence after R-CHOP (within ~12 months) justifies salvage chemotherapy with the aim of ASCT in cases of chemosensitivity. Only 1 cycle of R-RHAP is available, so the assessment continues. CAR-T is an evidence-based option in chemorefractoriness or when ASCT is not possible.

## ============================================================ gpt-5-nano (agent) ============================================================

**===== run_1 =====**

**Tumor board decision:**
CAR T-cell therapy preferred as second-line after R-RHAP in early relapse (<=12 months); mediastinal bulk as a bridge through moderate radiation therapy (4-6 Gy) to reduce tumor burden. If CAR-T is not possible/suitable: Auto-SCT after high-dose chemotherapy if there is an adequate response; Lymphoma conference involved on an ongoing basis.

**Rationale:**
Early recurrence with bulk speaks against direct SCT; Second-line CAR-T improves remissions; RT serves as a bridge to CAR-T planning and reduces CAR-T toxicity; SCT remains a backup option if CAR-T is not available/suitable. The procedure corresponds to the P/R flowchart approach.

**===== run_2 =====**

**Tumor board decision:**
Continue RHAP and evaluate response. For chemosensitive remission, SCT after high dose (BEAM) with autoSCT; Stem cell collection ideal before RT. Bulk bridge if necessary RT to SCT. If not in remission/not eligible for SCT: CAR T-cell therapy as second line; Bridging RT only after lymphoma conference.

**Rationale:**
ASCT standard for chemo-sensitive relapse; CAR-T alternative for SCT refusal/early recurrence. RT bridge useful for bulk, reduces tumor burden, facilitates transplant/CAR-T infusion. Close interaction with lymphoma conference; Check studies

**===== run_3 =====**

**Tumor board decision:**
Continue RHAP salvage; Restaging (PET-CT) after 2 cycles of RHAP. chemoresponse + SCT suitability -> high-dose chemotherapy + autoSCT; mediastinal bulk bridge, if necessary via radiotherapy before SCT or CAR-T. Refractory/no SCT capability -> CAR-T therapy after lymphoma conference; review studies; Consider bridging RT for bulk.

**Rationale:**
AutoSCT remains standard in chemosensitive relapse; CAR-T as an alternative for SCT inability or early progression; RT bridge useful for bulk reduction and transplant success; Clarify all options in lymphoma conference, check studies.

## ============================================================ gpt-5-nano (plain) ============================================================

**===== run_1 =====**

**Tumor board decision:**
Salvage with established regimen (R-ICE or R-DHAP) for 2-4 cycles; If the response is sufficient, subsequent high-dose chemotherapy with autologous stem cell transplantation (ASCT). In case of unsuitability or progression, alternative therapies/studies.

**Rationale:**
recurrence after R-CHOP; Chemo-sensitivity enables potential cure through salvage + ASCT. Radiotherapy only for residual bulking; consider clinical trials.

**===== run_2 =====**

**Tumor board decision:**
Second-line salvage chemo (R-ICE or R-DHAP) followed by ASCT if chemo-responsive. If transplantation is not possible or progression: CAR-T cell therapy (e.g. axi-cel) as second line. Radiotherapy only for symptomatic residual bulky disease.

**Rationale:**
DLBCL relapsed after R-CHOP with mediastinal bulk; Transplantation response prognostically favorable. Salvage+ASCT offers best survival; CAR-T32 as an alternative in cases of non-transplantability or rapid progression. No targeted mutation present; no other options prioritized.

**===== run_3 =====**

**Tumor board decision:**
Salvage chemotherapy targeting ASCT (e.g. R-DHAP or R-ICE) followed by transplantation if response. If ASCT is not possible/no response: Pola-BR or tafasitamab+lenalidomide; If necessary, mediastinal bulk radiotherapy.

**Rationale:**
Relapse after R-CHOP with bulk; ASCT with chemoresponse optimizes the long-term outcome. Without ASCT: Pola-BR or tafasitamab+lenalidomide; Local radiotherapy.

## ============================================================ gpt-oss-20b (agent) ============================================================

**===== run_1 =====**

**Tumor board decision:**
Transfer to CAR-T therapy (e.g. axicabtagene ciloleucel) after bridging chemotherapy; if CAR-T is not available, bispecific CD20xCD3 antibody (glofitamab/epcoritamab) or zanubrutinib combined chemotherapy.

**Rationale:**
R-RHAP after 1 cycle in case of recurrence shows low effectiveness; CAR-T is standard for fit patients. Bispecific antibodies and BTK inhibitors have high ORR/CR in ASH articles and studies and are valid alternatives.

**===== run_2 =====**

**Tumor board decision:**
In the event of early recurrence (<=12 months) after 6xR-CHOP21, we immediately check CAR-T suitability. If CAR-T is compatible, bridging therapy and CAR-T (e.g. axicabtagene-ciloleucel) start. If CAR-T is not capable, we recommend R-pola-bendamustine (or tafasitamab/lenalidomide) as the first salvage option; If there is further progression, HD salvage (R-DHAP/ICE) and, if necessary, auto-SCT if cardiac reserve is present.

**Rationale:**
Flowchart section V defines CAR-T as the first option for early relapse if there are no contraindications. Non-CAR-T-eligible patients receive R-pola-bendamustine or similar immunochemotherapeutic regimens; In cases of advanced progression, HD salvage and auto-SCT are the recommended alternatives.

**===== run_3 =====**

**Tumor board decision:**
In the case of early recurrence (<=12 months) after 6 cycles of R-CHOP21, CAR-T therapy (bridging) is the first choice, provided the patient is CAR-T capable. If CAR-T is false, we recommend salvage therapy with R-pola-bendamustine (or tafasitamab/lenalidomide) and review of clinical studies. Additional R-RHAP cycles are not recommended.

**Rationale:**
Flowchart section V specifies that in the event of early relapse, CAR-T has priority if suitable; If unsuitable, R-pola-bendamustine, tafasitamab/lenalidomide, BiTE therapies or clinical trials are the recommended options. R-RHAP has no evidence-based recommendation in this setting.

## ============================================================ gpt-oss-20b (plain) ============================================================

**===== run_1 =====**

**Tumor board decision:**
Continue with second-line salvage therapy (e.g. R-ICE or R-DHAP). After receiving 2-4 cycles and a positive response, autologous stem cell transplantation (ASCT) should be sought. If the course is refractory after ASCT, CAR-T therapy (tisagenlecleucel/axicabtagene ciloleucel) should be considered.

**Rationale:**
The patient showed an early recurrence after 6 cycles of R-CHOP and only received one cycle of R-RHAP - not sufficient. Standard salvage regimen (R-ICE/DHAP) followed by ASCT is the evidence-based option for DLBCL appeals. CAR-T therapy is intended as a third line in cases of refractoriness.

**===== run_2 =====**

**Tumor board decision:**
Further treatment with R-RHAP (2-3 cycles) to achieve remission, followed by evaluation for autologous stem cell transplantation. If there is no remission, consider CAR-T therapy (e.g. Tisa-cel).

**Rationale:**
R-RHAP is an established salvage regimen for R-CHOP resistance. After 6 cycles of R-CHOP and recurrence, a further 2-3 cycles of therapy is useful to assess ASCT candidacy. If unresponsive, CAR-T offers the current default option.

**===== run_3 =====**

**Tumor board decision:**
After unsuccessful R-CHOP and one cycle of R-RHAP, second-line salvage therapy with R-ICE (or alternatively R-GDP) is recommended, followed by ASCT evaluation in chemosensitive remission. If the patient is refractory or not suitable for ASCT, CAR-T-Cell therapy (Axi-Cel/Tisa-Cel) is the next option.

**Rationale:**
The patient has already received two different regimens; R-ICE/R-GDP are considered standard salvage therapies with high remission rates. ASCT is the only curative option for chemosensitive reactions. In the absence of remission or ASCT inability, CAR T-cell therapies are the evidence-based choice for R/R DLBCL.

## ============================================================ gpt-oss-120b (agent) ============================================================

**===== run_1 =====**

**Tumor board decision:**
Early recurrence <=12 months after R-CHOP -> immediate re-biopsy + staging, then check CAR-T therapy (CD19-targeted). If CAR-T is suitable, bridging chemo (e.g. R-DHAP further) up to apheresis and autologous SCT. If unsuitable: platinum-containing salvage (R-DHAP/ICE) -> HD-BEAM + Auto-SCT.

**Rationale:**
The patient has an early recurrence after CHOP basis, IPI2 and mediastinal bulk - classified as high risk. According to Flowchart V, CAR-T is the preferred option for early progression; alternatively platinum-based salvage and high-dose transplantation.

**===== run_2 =====**

**Tumor board decision:**

In the case of early recurrence (<12 months) after R-CHOP and 1 cycle of R-RHAP, the preferred approach is to link to CD19 CAR-T therapy. Carry out previous re-biopsy, restaging and organ function checks (ECG, LFT, kidneys, HBV/HCV). Use R-ICE (or R-DHAP) as a bridging salvage until apheresis. No routine CNS prophylaxis because CNS IPI is low.

**Rationale:**
Early recurrence after CHOP-based first-line therapy indicates a high risk of progression; According to the current SOP flowchart, CAR-T is the first choice of therapy for early progression, provided the patient is fit. Bridging chemo ensures disease control until cell apheresis. Autologous SCT is no longer favored after early recurrence.

**===== run_3 =====**

**Tumor board decision:**
In the case of early recurrence (<12 months) after 6xR-CHOP and 1 cycle of R-RHAP, the preferred approach is therapy with CD19 CAR T cells (e.g. axicabtagene ciloleucel). Before CAR-T allocation, carry out another biopsy, CD19 status, CNS-IPI clarification and, if necessary, bridging therapy (e.g. R-ICE). After CAR-T success, consider mediastinal consolidation radiation if necessary.

**Rationale:**
Early recurrence after first-line CHOP-based therapy meets the criteria for CAR-T eligibility. The patient is fit and there are no contraindications. CAR-T offers higher remission rates than conventional salvage chemotherapy and is the preferred option in early R/R-DLBCL, according to the flowchart.

## ============================================================ gpt-oss-120b (plain) ============================================================

**===== run_1 =====**

**Tumor board decision:**
Continuation of the salvage therapy R-RHAP (3 cycles in total), followed by PET-CT control. If there is a response (CR/PR), high-dose chemotherapy and autologous stem cell transplantation. If there is no response, alternative salvage regimens or CAR-T therapy in clinical trials.

**Rationale:**
Relapse after R-CHOP requires curative-intensive therapy. R-RHAP is an established salvage regime; ASCT is standard for chemosensitive disease. If the disease is refractory, switching to another salvage regimen or CAR-T is the recommended option.

**===== run_2 =====**

**Tumor board decision:**
Continue with R-RHAP (2-3 cycles in total), then restaging. For chemosensitive disease, high-dose chemotherapy (e.g. BEAM) and autologous stem cell transplantation. If there is no response rate or transplantation contraindication, CAR-T therapy (axi-cel / tisa-cel).

**Rationale:**
Relapse after R-CHOP requires salvage therapy. R-RHAP is established; the definitive curative option if there is a response is ASCT. In cases of refractory disease or intolerance, CAR-T is the current alternative.

**===== run_3 =====**

**Tumor board decision:**
Continuation of salvage therapy with R-RHAP for a total of 3 cycles, followed by restaging (PET-CT). If there is a response (CR/PR), high-dose chemotherapy and autologous stem cell transplantation are performed. If there is no response or intolerance, alternative salvage regimens (e.g. R-ICE) or inclusion in a CAR-T study protocol.

**Rationale:**
Relapse after 6 cycles of R-CHOP requires chemosensitive salvage therapy and consolidation with ASCT, the standard in younger, transplant-eligible patients. R-RHAP is an established regime; 3 cycles enable sufficient tumor reduction and mobilization of stem cells. If there is no response, a switch to another salvage regimen or CAR-T therapy is indicated.

## ============================================================ qwen3-32b (agent) ============================================================

===== run_1 =====

**Tumor board decision:**
After R-CHOP recurrence and one cycle of R-RHAP, salvage chemotherapy (R-DHAP/R-ICE) followed by HD-BEAM and autoSCT is recommended. CAR-T therapy should be considered for IPI 2 and mediastinal bulk. Clinical studies must be examined urgently.

**Rationale:**
The patient with DLBCL-IPI 2 and mediastinal bulk has an early recurrence after R-CHOP. Salvage chemotherapy with platinum-containing regimen (R-DHAP/R-ICE) is standard, followed by autoSCT. CAR-T therapy is an alternative if HD is suitable. Clinical trials (e.g. CAR-T, BiTE) are prioritized. Radiological re-evaluation and re-biopsy are required.

===== run_2 =====

**Tumor board decision:**
Recommendation: Salvage chemotherapy with R-DHAP/R-ICE, followed by high-dose chemotherapy (HD-BEAM) and autologous stem cell transplantation (autoSCT) if there is a response. Consider CAR T-cell therapy in parallel. Include clinical studies to optimize therapy.

**Rationale:**
After R-CHOP relapse and one cycle of R-RHAP (less established salvage standard), a platinum-containing salvage regimen (R-DHAP/R-ICE) is indicated. For CR/PR after salvage chemotherapy, HD-BEAM + autoSCT is useful for IPI 2 and mediastinal bulk. Check CAR-T suitability early, as IPI 2 and age (not specified) do not represent a contraindication. Clinical studies (e.g. on BiTEs or combination therapies) can be prioritized.

===== run_3 =====

**Tumor board decision:**
Recommendation: CAR-T cell therapy after bridging therapy (e.g. R-pola-bendamustine) if CAR-T is suitable. Alternatively: salvage chemotherapy (R-DHAP/R-GDP) followed by auto-SCT for CR/PR. Review clinical trials.

**Rationale:**
Early recurrence after R-CHOP (<=12 months). CAR-T is the first choice if you are suitable. Salvage chemotherapy (R-DHAP/R-GDP) with auto-SCT for CR/PR alternatively. Mediastinal bulk and IPI 2 suggest aggressive therapy. R-RHAP cannot be adequately assessed after 1 cycle; therefore bridging therapy is required.

## ================================================================ qwen3-32b (plain) ================================================================

===== run_1 =====

**Tumor board decision:**
Recommendation: Complete salvage therapy with R-RHAP and then perform high-dose chemotherapy with autologous stem cell transplantation (ASCT).

**Rationale:**
In early recurrence situations after R-CHOP and intermediate IPI, ASCT is the standard consolidation after successful salvage therapy. R-RHAP already showed treatment response, hence continuation and transition to transplantation.

===== run_2 =====

**Tumor board decision:**
Continuation of R-RHAP with subsequent evaluation of treatment response. If there is a response, recommendation for autologous stem cell transplantation (ASCT) as part of consolidation.

**Rationale:**
After relapse with R-CHOP, R-RHAP is an established salvage regimen. If there is a response, ASCT is the standard consolidation. Molecular data are missing, therefore no targeted therapy is possible.

===== run_3 =====

**Tumor board decision:**
Continuation of R-RHAP and evaluation of transplantability. If there is a response, plan autologous stem cell transplantation (ASCT). If unresponsive, consider R-EPOCH or clinical trials.

**Rationale:**

DLBCL recurrence after R-CHOP. R-RHAP used as salvage therapy. IPI 2 speaks for a medium prognosis. ASCT is standard upon response. Alternatively, R-EPOCH or non-response studies. Age and ECOG are missing, but limit treatment options.

## ============================================================ qwen3-next-80b (agent) ============================================================

**===== run_1 =====**

**Tumor board decision:**
CAR-T therapy if CAR-T is suitable. Otherwise switch to R-Pola-Bendamustine. Continuation of R-RHAP and auto-SCT are not indicated.

**Rationale:**
Early relapse (<=12 months after first line), therefore CAR-T as standard therapy. Auto-SCT is typically indicated for late recurrence (>12 months). R-RHAP is not optimal for early recurrences as CAR-T shows better results.

**===== run_2 =====**

**Tumor board decision:**
Continuation of R-RHAP therapy as bridging up to CAR-T therapy, no autoSCT transfer.

**Rationale:**
Early relapse (<=12 months after first-line) requires CAR-T as the preferred option. AutoSCT is contraindicated in early recurrence. R-RHAP serves as bridging to prepare for CAR-T transplantation. No alternative to CAR-T in terms of eligibility.

**===== run_3 =====**

**Tumor board decision:**
Do not recommend continuation of R-RHAP. If CAR-T is suitable, direct transfer to CAR-T therapy. If unsuitable, switch to R-pola-bendamustine or tafasitamab/lenalidomide. Do not recommend AutoSCT.

**Rationale:**
Early relapse (<=12 months after first-line) requires CAR-T as the preferred option. R-RHAP is not a standardized salvage therapy for early recurrence. AutoSCT is not recommended for early recurrence because CAR-T shows better results. R-pola-bendamustine/tafasitamab/lenalidomide are alternatives if not suitable for CAR-T.

## ============================================================ qwen3-next-80b (plain) ============================================================

**===== run_1 =====**

**Tumor board decision:**
CAR-T therapy (e.g. axicabtagene ciloleucel) as the next therapeutic step. Do not continue salvage chemotherapy.

**Rationale:**
Early recurrence (<12 months) after R-CHOP. ZUMA-7 trial shows superiority of CAR-T over salvage chemotherapy + ASCT. CAR-T is the current standard therapy for early-onset DLBCL.

**===== run_2 =====**

**Tumor board decision:**
Continue salvage therapy with R-DHAP for 3-4 cycles, followed by autologous stem cell transplantation if there is a response. If there is progression, consider CAR-T therapy (e.g. Axi-cel).

**Rationale:**
After R-CHOP recurrence, salvege therapy is standard. R-RHAP is interpreted as a typo for R-DHAP. ASCT indicated if there is a response (no secondary diagnoses). CAR-T for refractory disease according to guidelines.

**===== run_3 =====**

**Tumor board decision:**
Continue salvage therapy (e.g. R-DHAP) for 4 cycles, followed by response assessment. If in remission: autologous stem cell transplantation (ASCT); in case of progression: CAR-T therapy.

**Rationale:**
Relapse after R-CHOP requires salvage therapy. R-DHAP is standard. After completion response assessment. ASCT for remission, CAR-T for progression (NCCN guidelines).

# mm_case_1

Age: 72

ECOG: 2

Primary diagnosis:
Multiple myeloma, type IgG kappa, ID 06/2025
ISS-Stage: III; R2-ISS: III
Treatment indication: Anemia, multiple osteolytic lesions, Hypercalcemia, Renal insufficiency

Secondary diagnoses / Comorbidities:
Dialysis-dependent renal insufficiency (myeloma kidney)
Drug allergies: none known
COPD
Arterial hypertension

Prognostic factors:
Initial with MM:
ss2MG 8,5 mg/l, Albumin 22 g/l, LDH 189 U/l, ISS III, R2 ISS III
Cytogenetics: Standard-risk cytogenetics (Note: analysis not performed on sorted plasma cells).

Treatment and course:

Question for the tumor board:
Treatment initiation

Tumor board decision:
Dara VCD

# mm_case_2

Age: 62

ECOG: 0

Primary diagnosis:
Multiple myeloma, type IgA lambda, ID 01/2025
Treatment indication: Light chain ratio >100, Osteolytic lesions, Anemia

Secondary diagnoses / Comorbidities:
Drug allergies: none
Tobacco use 20 pyrs

Prognostic factors:
Initial with MM (12/2024):
ss2MG 7,8 mg/l, Albumin 21 g/l, LDH 142 U/l, ISS III, R2 ISS VI (high)
Cytogenetics: Deletion 17p13 (42% of cells) as well as IgH rearrangement (82%) as translocation t(4;14) (85%). High-risk cytogenetics.
Molecular genetics: no mutations in CKN2C, FAF2, TP53 genes

Treatment and course:

Question for the tumor board:
Presentation per SOP. Recommended management?

Tumor board decision:
We recommend a repeat high-dose chemotherapy and autologous blood stem cell transplantation as a tandem transplantation.

# mm_case_3

Age: 74

ECOG: 0

Primary diagnosis:
Multiple myeloma, type IgA lambda, ID 08/2023
Treatment indication: Progressive focal lesions

Secondary diagnoses / Comorbidities:
CIPN grade II
Allergies: none known

Prognostic factors:
ss2MG 3,0 mg/l, Albumin 42 g/l, LDH 198'
ISS 1; R-ISS 1
Cytogenetics: Translocation t(11;14), one deletion 13q and one 1q gain
Prognostic score (Mayo Clinic criteria): 1
* M-Protein > 15g/l, Non-IgG, Serum free light chain ratio < 0.26 or > 1.65

Treatment and course:

Question for the tumor board:
Recommended management?

Tumor board decision:
KCD Therapy

# mm_case_4

Age: 71

ECOG: 0

Primary diagnosis:
Monoclonal gammopathy of undetermined significance type IgG Lambda, ID 09/2024

Secondary diagnoses / Comorbidities:
Drug allergies: none
Type 2 diabetes mellitus
Arterial hypertension

Prognostic factors:
Initial with MGUS:
Prognostic score (Mayo Clinic criteria): 0
(one point each: M-Protein > 15g/l, Non-IgG, Serum free light chain ratio < 0.26 or > 1.65; 0 points: low; 1 point: intermediate; 2 points: intermediate-high; 3 points: high)

Treatment and course:

Question for the tumor board:
Treatment indication?

Tumor board decision:
In summary of the findings, there is currently a monoclonal gammopathy of undetermined significance IgG type Lambda. In monoclonal gammopathy, there is no treatment indication

# mm_case_5

Age: 55

ECOG: 1

Primary diagnosis:
Multiple myeloma, type IgG type Lambda, ID 11/17
Stage IIA after Salmon and Durie
Treatment indication: Osteolytic lesions

Secondary diagnoses / Comorbidities:
Drug allergies: none
History of Tobacco use 15py

Treatment and course:

Question for the tumor board:
Recommended management?

Tumor board decision:
MEK Inhibitor Therapy

# mm_case_6

Age: 59

ECOG: 1

Primary diagnosis:
Multiple myeloma, light chain type Kappa, ID 07/2021
ISS-Stage: III; R2-ISS: III
Treatment indication: Renal insufficiency, Osteolytic lesions

Secondary diagnoses / Comorbidities:
Drug allergies: Metamizol
Chemotherapy-associated PNP
Chronic hemodialysis for end-stage renal disease
Secondary anemia
Arterial hypertension

Prognostic factors:
Initial with MM:
ss2MG 42,3 mg/l, Albumin 28g/l, LDH 295U/l, ISS III, R2 ISS III

Treatment and course:

Question for the tumor board:
Recommended management?

Tumor board decision:
Indication for CAR- Therapy. Referral to Cell Therapy Board

# mm_case_7

Age: 68

ECOG: 1

Primary diagnosis:
Multiple myeloma, type IgG Kappa, ID 01/2021
CRAB criteria: Anemia; Osteolytic lesions
Revised ISS: Stage II
Stage per Durie-Salmon: II

Secondary diagnoses / Comorbidities:
Allergies: Pollen
Oral anticoagulation with Apixaban
TAA after autologous SCT and D-VTD
Successful pulmonary vein isolation 01/2023
Arterial hypertension

Treatment and course:

Question for the tumor board:
Recommended management?

Tumor board decision:
Currently, there was rapid progression under Lenalidomid maintenance. Planning CAR-T therapy with Cilta-Cel.

# mm_case_8

Age: 68

ECOG: 2

Primary diagnoses:
Plasma cell leukemia with multiple myeloma IgG Kappa, stage III A per Salmon and Durie, ID 01/2012

Secondary diagnoses / Comorbidities:
History of Appendectomy
Allergies: Hay fever

Prognostic factors:
Initial: 2,6 mg/l; Albumin 33,1 g/l; ISS II
Cytogenetics (09/12): No High-risk genetics.

Treatment and course:

Molecular genetic findings

Question for the tumor board:
Pathogenic variant? Targeted Therapy?

Tumor board decision:
The variant is pathogenic. We recommend MEK inhibitor-based therapy.

# mm_case_9

Age: 66

ECOG: 1

Primary diagnoses:
Plasma cell leukemia with multiple myeloma IgG Kappa, stage III A per Salmon and Durie, ID 01/2012

Secondary diagnoses / Comorbidities:
History of Appendectomy
Allergies: Hay fever

Prognostic factors:
Initial: 2,4 mg/l; Albumin 31,1 g/l; ISS II
Cytogenetics (09/12): No High-risk genetics.

Treatment and course:

Molecular genetic findings

Question for the tumor board:
Pathogenic variant? Targeted Therapy?

Tumor board decision:
The NRAS variant is pathogenic. We recommend MEK inhibitor-based therapy.

# mm_case_10

Age: 64

ECOG: 1

Primary diagnoses:
Plasma cell leukemia with multiple myeloma IgG Kappa, stage III A per Salmon and Durie, ID 01/2012

Secondary diagnoses / Comorbidities:
History of Appendectomy
Allergies: Hay fever

Prognostic factors:
Initial: 2,6 mg/l; Albumin 33,1 g/l; ISS II
Cytogenetics (09/12): No High-risk genetics.

Treatment and course:
Molecular genetic findings

FISH findings:
Deletion 1q21 (22%) and 13q14 (20%), Gain 11q22.3 (34%, three copies), IgH-Rearrangement (33%) as Translocation t(11;14) (36%) Incidentally Deletion 14q32 (35%).

Question for the tumor board:
Pathogenic variant? Targeted Therapy?

Tumor board decision:
No variant on NGS. t(11;14) in current FISH findings. Therefore, Venetoclax-based therapy recommended.

# mm_case_11

Age: 68

ECOG: 1

Primary diagnosis:
Multiple myeloma, type IgG kappa, ID 01/2026
Stage: R-ISS II
Treatment indication: Anemia, Osteolytic lesions, Hypercalcemia

Secondary diagnoses / Comorbidities:
Allergies: none
Graves' disease
CVI grade III

Prognostic factors:
Initial with MM: no evidence of del(17p), t(4;14) or t(14;16)

Treatment and course:

Question for the tumor board:
Recommended management?

Tumor board decision:
Continuation of bridging therapy with IsaKd. Referral to CAR-T outpatient clinic for BCMA-directed CAR-T cell therapy in the further course.

# mm_case_12

Age: 60

ECOG: 0

Primary diagnosis:
Multiple myeloma, type IgG Kappa ID 01/26
ISS-Stage: III. R2-ISS: III
Treatment indication: Anemia, multiple osteolytic lesions, Hypercalcemia

Secondary diagnoses / Comorbidities:
Drug allergies: none known
Hypothyroidism
CKD Stage III

Prognostic factors:
Initial with MM:
ss2MG 9,2 mg/l, Albumin 24 g/l, LDH 165 U/l, ISS III, R2 ISS III
Cytogenetics: no evidence of High-risk aberrations

Secondary diagnoses / Comorbidities:
None

Treatment and course:

Question for the tumor board:
Recommended management?

Tumor board decision:
Switch to D-VRD, HD Melphalan and ASCT.

# mm_case_13

Age: 70

ECOG: 0

Primary diagnosis:
Suspected Waldenstrom macroglobulinemia with IgM gammopathy 01/26
MYD88 negative
Whole-body CT and bone marrow biopsy unremarkable
No light chain restriction

Secondary diagnoses / Comorbidities:
Allergies: none known
AIHA

Treatment and course:
Suspected Waldenstrom macroglobulinemia 01/26

Question for the tumor board:
Recommended management?

Tumor board decision:
Complete diagnostics. Evaluation of therapy with Rituximab in case of persistent hemolysis / R-Bendamustin upon diagnosis of Waldenstrom macroglobulinemia.

# mm_case_14

Age: 52

ECOG: 1

Primary diagnosis:
POEMS syndrome ID 01/2025, confirmed by sclerotic bone lesions, Polyneuropathy, monoclonal IgA lambda, elevated VEGF
Secondary diagnoses / Comorbidities:
Allergies: none known

Activity parameters:
Date VEGF (pg/ml) M-Protein (g/l) Other
01/2025 1850 3,2 ID, multiple sclerotic lesions BWS
04/2025 620 1,8 post 4x VCD
07/2025 580 1,5 stable, PET/CT: regression of most lesions

Treatment and course:
01/2025 ID POEMS syndrome
02-05/2025 4 cycles VCD (Bortezomib, Cyclophosphamid, Dexamethason)
06/2025 Remission assessment: partial remission (PR), VEGF-decrease >50%
07-09/2025 Treatment break, follow-up monitoring
10/2025 PET/CT: isolated progression BWK 2 with otherwise stable findings, VEGF still stable at 610 pg/ml

Question for the tumor board:
Recommended management for isolated local progression BWK 2 with otherwise stable VEGF and systemic remission?

Tumor board decision:
Recommendation: Given imaging and clinically largely stable findings and a single moderately progressive lesion in BWK2, local therapy via radiation can be administered in this area. Further follow-up assessments should be performed via PET/CT.

# mm_case_15

Age: 56
ECOG: 1
Primary diagnosis:
Multiple myeloma, IgG lambda type, ID 06/2025, from solitary plasmacytoma BWK 10 (01/2024), R-ISS II, standard-risk cytogenetics
SLiM-CRAB: disseminated Osteolytic lesions
Secondary diagnoses / Comorbidities:
History of Pulmonary embolism 12/2025
Allergies: none known
Prognostic factors:
Cytogenetics (01/2026): Standard risk

Treatment and course:
01/2024 ID solitary plasmacytoma BWK 10
01/2024 Laminectomy BWK 10
04-05/2024 Radiation therapy BWK 9-11, GD 36 Gy
01/2026 Whole-body MRI: Progression to MM

Question for the tumor board:
Treatment initiation for initial diagnosis of MM from solitary plasmacytoma
Tumor board decision:
Treatment initiation with Dara-VRd with continuation of anticoagulation. Reduction of Lenalidomid dosage due to the recent PE in the first cycle. Principally eligible for high-dose therapy, consolidation and maintenance therapy in the further course.

# mm_case_sim_1

Age: 54

ECOG: 1

Primary diagnosis:
Secondary hyposecretory Multiple myeloma, type IgA Kappa, ID 06/2021
Stage per Salmon and Durie: n.v. (not available)
ISS: St. II; R-ISS: St. II
Treatment indication: Anemia

Secondary diagnoses / Comorbidities:
Drug allergies: none known
History of Appendectomy
History of Tobacco use (10 PY)

Prognostic factors:
Initial (external):
ss2MG n.v. (not available), Albumin n.v. (not available), ISS St. II
Cytogenetics: Gain 1q21 (22%, three copies). High-risk aberration

Treatment and course:
08-11/2021 4 cycles VCD
Remission status: VGPR
11/2021 Stem cell mobilization with Cyclophosphamid 2000 mg/m2 (d1,2)
12/2021 Stem cell apheresis
15.01.2022 High-dose therapy with Melphalan and autologous SCT
11/2022 PD
from 22.11.2022 Daratumumab, Carfilzomib, Lenalidomid, Dexamethason
03/2023 PD
20.03.2023 Initiation relapse therapy with the 1. cycle Pom-PACE
06/23 Remission status before apheresis: formally not assessable (asecretory)
28.06.2023 Successful T-cell collection
30.06.2023 4. cycle Pom-PACE (
07/23 Remission status before CAR-T cell therapy: formally not assessable;
morphologically on imaging most likely CR; MRD negativity
09.-11.08.23 Lymphodepletion with Fludarabin and Cyclophosphamid
14.08.23 CAR-T cell therapy Idecabtagen Vicleucel (Abecma®)
Since 09/23 Remission status after CAR-T cell therapy: formally not assessable;
morphologically on imaging most likely CR; MRD negativity
08/25 Remission status: imaging Progression
08/25 - 06/25 3 cycles CED
06/25 Remission status before apheresis: most recently on imaging Progression
05.07.2025 T-cell apheresis
07-08/25 Initiation Talquetamab
08/25 PD

Analysis data:

Question for the tumor board:
Recommended management?

Tumor board decision:
Therapy with MEK-Inhibitor

# mm_case_1__ALL

################################
#### CASE: 1
################################

## ================================================================ gpt-5-mini (agent) ================================================================

**===== run_1 =====**

**Tumor board decision:**
Immediate induction with daratumumab s.c. + Bortezomib s.c. (weekly) + cyclophosphamide (reduced dose, IV after dialysis) + dexamethasone (Dara-VCd). Lenalidomide should initially be avoided in cases of renal insufficiency requiring dialysis. Clarify transplantation immediately; Stem cell mobilization/apheresis after ~3 cycles with G-CSF +/- plerixafor; Minimize alkylator before collection. thrombosis/infection prophylaxis; Check osteoprotection (if necessary denosumab if dialysis).

**Rationale:**
For renal insufficiency requiring dialysis, Flowchart recommends Dara-VCd: initially avoids lenalidomide (renal elimination), bortezomib does not require dose adjustment, daratumumab s.c. better for COPD. Early ASCT clarification and prompt stem cell collection necessary; Give cyclophosphamide post-dialysis and reduced.

**===== run_2 =====**

**Tumor board decision:**
Immediate induction with daratumumab subcutaneously + bortezomib s.c. (weekly) + cyclophosphamide + dexamethasone (Dara-VCd). At the same time, rapid clarification of transplantability (cardio/pulmonary/PS). After 3-4 cycles, early stem cell mobilization (G-CSF +/- Plerixafor) and ASCT if suitable. Avoid lenalidomide initially during dialysis; Adjust maintenance after ASCT to renal function.

**Rationale:**
Renal insufficiency requiring dialysis and multiple CRAB criteria require rapid treatment. Flowchart recommends DaraVCd instead of lenalidomide-containing regimens for patients requiring dialysis. Daratumumab s.c. preferred because of COPD and lower infusion reaction rate. Bortezomib and cyclophosphamide effective in renal insufficiency; Bortezomib at usual dose (s.c., weekly), cyclophosphamide, dose reduction if necessary depending on clinical status; Low dose dexamethasone. Early mobilization/ASCT if fit; initiate osteoprotection, infection and thrombosis prophylaxis; If kidney function improves, consider switching/maintenance with lenalidomide.

**===== run_3 =====**

**Tumor board decision:**
Begin immediate induction: daratumumab (preferably SC) + bortezomib (SC, weekly) + cyclophosphamide + dexamethasone (Dara-VCd). In parallel: quickly clarify transplantability; If suitable, stem cell mobilization after 3-4 cycles and planned ASCT. Avoid lenalidomide in dialysis or only with specific medication. Dosing schedule.

**Rationale:**
Renal insufficiency requiring dialysis favors Dara-VCd (avoids lenalidomide problems). Daratumumab s.c. favorable because of COPD. Rapid onset due to hypercalcemia, anemia and osteolysis. Bortezomib s.c. good in renal failure. Plan mobilization early; HSV prophylaxis, thrombosis prophylaxis and nephrology/transplant center coordination recommended.

## ================================================================ gpt-5-mini (plain) ================================================================

**===== run_1 =====**

**Tumor board decision:**
Immediate induction treatment with a bortezomib-based triplet (VCD): bortezomib s.c. 1.3 mg/m2 d1,4,8,11 (cycle dependent) + cyclophosphamide (e.g. 300 mg/m2 p.o. d1,8,15) + dexamethasone (20-40 mg/weekly). If available and without a contraindication, supplementation with daratumumab is recommended. Discontinue zoledronic acid and use denosumab 120 mg s.c. instead. q4w. Supportive: rapid dialysis, correction of hypercalcemia, transfusion/ESA as needed, and infection prophylaxis.

**Rationale:**
In cases of renal insufficiency requiring dialysis, bortezomib-based regimens are the first choice due to rapid improvement in kidney function and good effectiveness; Lenalidomide difficult to dose during dialysis. Daratumumab increases remission rates. Zoledronic acid is nephrotoxic - Denosumab is kidney independent.

**===== run_2 =====**

**Tumor board decision:**
Immediate cytoreductive induction with bortezomib-based triple therapy: VCd (subc. bortezomib + cyclophosphamide + dexamethasone). If available and without contraindication, addition of daratumumab is possible. Supportive: dialysis/acute hypercalcemia treatment, HSV prophylaxis, thrombosis prophylaxis; Stop zoledronate and switch to denosumab. Check transplant suitability based on primary response.

**Rationale:**
Renal insufficiency requiring dialysis and acute organ manifestation require rapid cytoreductive therapy. Bortezomib is rapid-acting and safe in renal insufficiency; Cyclophosphamide initially avoids lenalidomide problems during dialysis. Daratumumab can deepen remission. Bisphosphonates (zoledronate) problematic in dialysis -> denosumab preferred.

**===== run_3 =====**

**Tumor board decision:**
Immediate induction with bortezomib-based triple (CyBorD): Bortezomib s.c. 1.3 mg/m2 D1,4,8,11 q21d; Cyclophosphamide 300 mg/m2 p.o. weekly (adjust to dialysis timing); Dexamethasone 20-40 mg/week. Acyclovir prophylaxis. discontinue bisphosphonate; Denosumab 120 mg s.c. q4w + Ca/VitD. Clarify stem cell reserve and nephro-oncological coordination.

**Rationale:**
Urgent rapid cytoreduction due to anemia, hypercalcemia, osteolysis and renal insufficiency requiring dialysis. Bortezomib works quickly and does not require dosage adjustment in kidney failure. Zoledronate problematic in dialysis -> Denosumab preferred. Antiviral prophylaxis due to bortezomib; ASCT evaluation after response.

## ================================================================ gpt-5-nano (agent) ================================================================

**===== run_1 =====**

**Tumor board decision:**
Induction MM: DaraVCd (daratumumab + bortezomib + cyclophosphamide + dexamethasone) as first-line therapy. Denosumab instead of zoledronate in dialyzing NI. Then high-dose melphalan with autologous SCT; If necessary, tandem SCT if high risk or no CR after first high dose.

**Rationale:**
Renal insufficiency, hypercalcemia, anemia and osteolysis speak against lenalidomide; Denosumab is preferred for renal insufficiency. CD38-based induction with PI components is effective and well tolerated. Early SCT improves PFS/OS in ISS III/R2-ISS III high risk; If CR is inadequate, consider tandem SCT if necessary.

**===== run_2 =====**

**Tumor board decision:**
Induction: DaraVCd in MM requiring dialysis (ISS III/R2-ISS III). Replace zoledronate with denosumab. Early SCT mobilization; Consolidation: HD-Melphalan 200 mg/m2; If necessary, second SCT if high risk. Maintenance: Lenalidomide if kidney function is adequate; alternatively pomalidomide.

**Rationale:**
High-risk MM with renal insufficiency requires rapid, highly effective induction. DaraVCd is renal compatible; Denosumab preferred in renal insufficiency. Early ASCT increases CR rate; Individualize or adjust maintenance depending on kidney status.

**===== run_3 =====**

**Tumor board decision:**
Induction: DaraVCd as first-line therapy for MM requiring dialysis. Denosumab instead of zoledronate. Maintenance: Lenalidomide if the course goes well (with dose adjustment). Early autologous stem cell transplantation recommended; If necessary, second SZT/tandem per response.

**Rationale:**
High-risk MM (ISS III, R2-ISS III) with anemia, osteolysis, hypercalcemia, and renal insufficiency requires aggressive induction and SCT consolidation. Denosumab makes sense in NI. HDT-ASCT improves prognosis; Early transplantation makes sense; Depending on the course, possibly tandem according to CR status.

## ========================================================== gpt-5-nano (plain) ==========================================================

**===== run_1 =====**

**Tumor board decision:**
Induction MM in patients requiring dialysis, ISS III: start bortezomib-based therapy (VD or VCD) with dexamethasone; Aim for rapid remission and preparation for autologous SCT. Continue zolendronate; Consider denosumab if there is a renal contraindication. Check SCT after response.

**Rationale:**
Bortezomib-based induction is renally safe and enables rapid cytoreduction in dialysis patients; VD/VCD is effective, well tolerated, and facilitates transplant options; High urgency due to hypercalcemia legitimizes rapid remission.

**===== run_2 =====**

**Tumor board decision:**
Inductive treatment with bortezomib-based triplet (VCD or VD-Dexa) for 4-6 cycles. Bortezomib is preferred for renal insufficiency requiring dialysis; Replace zoledronate with denosumab; address hypercalcemia; then testing for autologous stem cell transplantation, if conditions apply.

**Rationale:**
Renal dialysis requires renal independence of therapy; Bortezomib-based regimens provide rapid remission rates. Denosumab is more suitable than zoledronate for renal insufficiency. Goal: rapid stress reduction and transplant option, if the general condition allows this.

**===== run_3 =====**

**Tumor board decision:**
Inductive MM therapy for IgGκ with renal insufficiency requiring dialysis: initiate bortezomib-based triplet therapy (VCD) or alternatively Vd to achieve rapid cytoreduction. Continue depending on response; Subsequently, if necessary, autologous SCT, if suitable. Continue zoledronate; Treat hypercalcemia appropriately.

**Rationale:**
Bortezomib-based induction is preferred for renal damage (no renal dose adjustment required) and provides rapid remission. High tumor burden (ISS III, R2) speaks in favor of triplet regimen. No high-risk cytogenetics. SCT after response increases long-term control; Check reserves for transplantation.

## ========================================================== gpt-oss-20b (agent) ==========================================================

**===== run_1 =====**

**Tumor board decision:**
Start DaraVCd induction (Daratumumab 16mg/kg SC, Bortezomib 1.3mg/m2 SC qwk, Cyclophosphamide 300mg/m2 IV, Dexamethasone 40mg qwk) 4-6 cycles, then assess for autologous SCT if fit. Continue bisphosphonate, infection and thrombosis prophylaxis.

**Rationale:**
Patient ISSIII, R2-ISSIII, dialyzed renal failure -> lenalidomide contraindicated. DaraVCd is guideline-recommended for dialyzed patients, combining effective anti-CD38, PI and alkylator. SC daratumumab mitigates asthma risk; weekly bortezomib limits neuropathy. Aggressive induction and SCT are warranted for high-risk diseases.

**===== run_2 =====**

**Tumor board decision:**
Start daratumumab+bortezomib+cyclophosphamide+dexamethasone (DaraVCd) as an induction regimen. Daratumumab (s.c. possible) offers high effectiveness in R2-ISSIII, bortezomib is dialysis-safe, cyclophosphamide does not cause renal excretion. Lenalidomide not recommended due to renal

insufficiency. Supplement osteoprotection, infection and thromboprophylaxis.

**Rationale:**
Patient with ISSIII, R2-ISSIII, renal insufficiency requiring dialysis, anemia, osteolysis, hypercalcemia. According to the flowchart, daratumumab + bortezomib + cyclophosphamide is the recommended first-line therapy for renal insufficiency requiring dialysis. Lenalidomide would be renal burden; Bortezomib subcutaneously reduces neuropathy. Daratumumab s.c. minimizes COPD infusion reactions. Prophylaxis for bones, infection and thrombosis is mandatory.

**===== run_3 =====**

**Tumor board decision:**
Start induction with DaraVCd (Daratumumab+Bortezomib+Cyclophosphamide+Dexamethasone) subcutaneously, weekly, with renal-adjusted doses; evaluate for autologous stem-cell transplant if fit.

**Rationale:**
Dialysis-dependent renal insufficiency makes lenalidomide contraindicated. According to the flowchart, DaraVCd is the recommended first line for dialysis patients. Subcutaneous daratumumab administration reduces infusion reactions in COPD; Weekly bortezomib minimizes neuropathy, cyclophosphamide is dialysis tolerant.

## ========================================================== gpt-oss-20b (plain) ==========================================================

**===== run_1 =====**

**Tumor board decision:**
Start daratumumab 16mg/kg IV + bortezomib 1.3mg/m2 SC weekly + dexamethasone 20mg PO weekly (Dara-Vd) as induction; adjust bortezomib dose for dialysis; continue bisphosphonate, manage anemia, dialysis, hypercalcemia.

**Rationale:**
High-risk MM with renal failure; daratumumab-bortezomib-dexamethasone is evidence-based, effective, and tolerable in dialysis. Lenalidomide contraindicated; supportive care essential.

**===== run_2 =====**

**Tumor board decision:**
Start daratumumab 16mg/kg IV + bortezomib 1.3mg/m2 SC 2x/wk + dexamethasone 20mg wkly (Dara-Vd) induction. Avoid lenalidomide for renal insufficiency requiring dialysis. Continue until >=VGPR or 4-6 cycles.

**Rationale:**
High-risk ISSIII, renal insufficiency requiring dialysis -> bortezomib-based regimen preferred. Daratumumab increases response rates and is renally safe. Lenalidomide is contraindicated at CrCl<30ml/min. Combination provides rapid control of anemia, osteolysis, hypercalcemia.

**===== run_3 =====**

**Tumor board decision:**
Start Daratumumab 16mg/kg IV + Bortezomib 1.3mg/m2 SC (qwk) + Dexamethasone 20mg PO (days1-4,8-11,15-18,22-25) - 8wk induction, then every2wks. Adjust dex for renal function. Continue zoledronic acid, manage calcium, transfuse for anemia, dialysis as needed.

**Rationale:**
High-risk R2-ISSIII with renal failure; Daratumumab + Bortezomib + Dexamethasone is effective, renally safe, avoids lenalidomide. Supports rapid disease control, reduces osteolysis, hypercalcemia, and improves anemia.

## ========================================================== gpt-oss-120b (agent) ==========================================================

**===== run_1 =====**

**Tumor board decision:**
Induction with DaraVCd (daratumumab s.c.+bortezomib s.c.+cyclophosphamide+dexamethasone) for 4-6 cycles, then autologous stem cell transplantation (melphalan 200mg/m2, dose reduction if necessary) if fitness is sufficient, followed by lenalidomide maintenance (dose-adjusted for dialysis).

**Rationale:**

Patient meets SLiM-CRAB, has R-ISSIII and renal insufficiency requiring dialysis. According to the guidelines, DaraVCd is the recommended first-line therapy for dialysis, combining CD-38 antibodies, PI and alkylating agents. After a good response, high-dose transplantation enables a deeper remission; Lenalidomide maintenance reduces the risk of recurrence.

**===== run_2 =====**

**Tumor board decision:**
Initial induction with daratumumab+bortezomib+cyclophosphamide+dexamethasone (DaraVCd). If there is clinical remission and sufficient fitness (e.g. after dialysis stabilization), autologous stem cell transplantation with a reduced dose of melphalan. Maintenance with dose-adjusted lenalidomide+bortezomib.

**Rationale:**
The patient meets SLiM-CRAB and has renal insufficiency requiring dialysis. According to the guidelines, DaraVCd (or VRd) is recommended for this situation; Lenalidomide has a renal burden, so CD38 antibody-based with cyclophosphamide is preferred. High-dose therapy only if you are sufficiently fit, otherwise the regimen should be continued.

**===== run_3 =====**

**Tumor board decision:**
Start induction with DaraVCd (daratumumab+bortezomib+cyclophosphamide+dexamethasone) for 4-6 cycles, then consolidation with autologous stem cell transplantation if renal insufficiency requiring dialysis allows; otherwise received with dose-adjusted lenalidomide and osteoprotective therapy.

**Rationale:**
The patient meets SLiM-CRAB criteria, has high risk (ISSIII/R-ISSIII) and renal insufficiency requiring dialysis. Bortezomib and cyclophosphamide are safe in renal insufficiency, while daratumumab (subcutaneous) reduces respiratory risks. High-dose melphalan is problematic during dialysis, therefore transplantation only if the kidneys are stable; otherwise received with lenalidomide (dose-adjusted) and standard prophylaxis.

## =============================================================== gpt-oss-120b (plain) ===============================================================

**===== run_1 =====**

**Tumor board decision:**
Start of bortezomib-based induction triplet therapy (bortezomib 1.3 mg/m2 s.c. weekly, lenalidomide 10 mg/day on days 1-21 with dose adjustment for dialysis, dexamethasone 40 mg weekly) for 4-6 cycles, followed by evaluation for autologous stem cell transplantation; Continue bisphosphonates, treat hypercalcemia and anemia symptomatically.

**Rationale:**
ISSIII/R2-ISSIII, renal insufficiency requiring dialysis, and hypercalcemia require a fast-acting, kidney-friendly regimen. Bortezomib is safe in kidney failure, lenalidomide can be reduced in dose, and the triplet achieves high response rates, enabling later ASCT.

**===== run_2 =====**

**Tumor board decision:**
Start combination therapy with daratumumab, subcutaneous bortezomib, reduced-dose melphalan, and prednisone (D-VMP) for transplant-ineligible patients with renal insufficiency.

**Rationale:**
ISSIII/R2-ISSIII and renal insufficiency requiring dialysis require an effective, renal-safe regimen. Bortezomib acts quickly in hypercalcemia and is independent of dialysis, daratumumab increases the response rate, melphalan is tolerated with dose reduction on dialysis. No known contraindications.

**===== run_3 =====**

**Tumor board decision:**
Start of bortezomib-based triple therapy (bortezomib SC+lenalidomide (dialysis-adapted)+dexamethasone) as first-line therapy; If necessary, add daratumumab (Dara-VRd) after individual assessment. Continuation of bisphosphonates, calcium and fluid therapy, infection prophylaxis.

**Rationale:**

Newly diagnosed MM-ISSIII/R-ISSIII with renal insufficiency requiring dialysis; Bortezomib is renally safe, lenalidomide is dose-adjustable, and Triplel increases response rates. No high-risk cytogenetics, therefore standard triples recommended; Daratumumab may improve the depth of remission.

## ============================================================ qwen3-32b (agent) ============================================================

**===== run_1 =====**

**Tumor board decision:**
Recommended therapy: DaraVCd (daratumumab + bortezomib + cyclophosphamide + dexamethasone) as induction therapy. If the course goes well, maintenance with lenalidomide (dose adjustment in case of renal insufficiency).

**Rationale:**
DaraVCd is suitable for kidney failure requiring dialysis (flowchart PATH C). Bortezomib i.v. is chosen despite COPD because subcutaneous administration is not included in the scheme. Cyclophosphamide replaces lenalidomide to avoid renal excretory burden. Continue osteoprotection with zolendronate.

**===== run_2 =====**

**Tumor board decision:**
Recommended therapy: DaraVCd (daratumumab s.c. + bortezomib + cyclophosphamide + dexamethasone) as induction therapy. Maintenance with lenalidomide with good outcome. Monitoring: kidney function, calcium, myeloma response. Continue osteoprotection.

**Rationale:**
DaraVCd is beneficial in renal failure requiring dialysis and COPD (s.c. daratumumab reduces infusion reactions). Cyclophosphamide is tolerated on dialysis. Lenalidomide maintenance after response. High-dose therapy not indicated. Prophylaxis for osteoporosis and thromboembolism is mandatory.

**===== run_3 =====**

**Tumor board decision:**
Recommended therapy: DaraVCd (daratumumab s.c. + bortezomib + cyclophosphamide + dexamethasone) as induction therapy. If the course goes well, maintenance with lenalidomide (dose adjustment if GFR <50 ml/min). Continue osteoprotection with zolendronate.

**Rationale:**
According to guidelines, DaraVCd is the preferred induction for renal failure requiring dialysis. Cyclophosphamide replaces lenalidomide (renal excretion). Daratumumab s.c. reduces infusion risk in COPD. Lenalidomide maintenance only if renal function is tolerable. Continue zolendronate for osteoprotection.

## =========================================================== qwen3-32b (plain) ============================================================

**===== run_1 =====**

**Tumor board decision:**
Recommended therapy initiation: VTD regimen (bortezomib, thalidomide, dexamethasone) with dexamethasone dose adjustment due to COPD. Add denosumab instead of zoledronate due to the need for dialysis.

**Rationale:**
VTD is preferred in high-risk myeloma with renal failure. Bortezomib is renal protective, thalidomide tolerated in COPD, dexamethasone reduced to 20mg every 2 weeks. Denosumab arrests bone resorption during dialysis.

**===== run_2 =====**

**Tumor board decision:**
Recommendation: Start induction therapy with bortezomib (1.3 mg/m2) + dexamethasone (20 mg weekly) as duo therapy. Avoid lenalidomide due to renal insufficiency requiring dialysis.

**Rationale:**
Bortezomib/dexamethasone (VD) is safe in renal dysfunction, lenalidomide is contraindicated if eGFR <30 ml/min. ISS III/R2-ISS III requires effective therapy. Continue zoledronate for osteolysis prophylaxis.

**===== run_3 =====**

**Tumor board decision:**
Recommendation for the initiation of therapy with bortezomib, lenalidomide and dexamethasone (VRd) taking into account renal insufficiency.

**Rationale:**
VRd is standard induction therapy for newly diagnosed MM. Lenalidomide dose adjustment when requiring dialysis, subcutaneous bortezomib tolerated. COPD and hypertension not relevant for choice of therapy. Cytogenetics without high-risk factors. Hypercalcemia addressed by dexamethasone.

## ============================================================ qwen3-next-80b (agent) ============================================================

**===== run_1 =====**

**Tumor board decision:**
Induction therapy with DaraVCd (daratumumab + bortezomib + cyclophosphamide + dexamethasone) as first-line therapy

**Rationale:**
Patient meets SLiM-CRAB criteria with renal insufficiency requiring dialysis. Flowchart prioritizes DaraVCd for this case because cyclophosphamide is renal-friendly and lenalidomide must be avoided (renal excretion). Daratumumab s.c. reduces COPD risk. Osteoprotection and thrombosis prophylaxis are mandatory.

**===== run_2 =====**

**Tumor board decision:**
Induction therapy with DaraVCd (daratumumab subcutaneous + bortezomib + cyclophosphamide + dexamethasone).

**Rationale:**
Renal insufficiency requiring dialysis according to flowchart PATH C requires DaraVCd. Bortezomib is kidney independent, cyclophosphamide is adjustable. Daratumumab s.c. minimizes COPD-related infusion reactions. No IMiDs in the combination, therefore no standard thrombosis prophylaxis, but possibly aspirin for risk factors.

**===== run_3 =====**

**Tumor board decision:**
Induction with DaraVCd (daratumumab subcutaneous + bortezomib subcutaneous weekly + cyclophosphamide 300 mg/m2 + dexamethasone).

**Rationale:**
Renal insufficiency requiring dialysis requires induction with DaraVCd according to flowchart Path C. Lenalidomide is contraindicated in severe renal insufficiency. Daratumumab s.c. reduces COPD risk, bortezomib s.c. minimizes neuropathy. Adjust dexamethasone dose for hypertension. No repeat CD38 antibody necessary as first line.

## ============================================================ qwen3-next-80b (plain) ============================================================

**===== run_1 =====**

**Tumor board decision:**
Start VRd regimen with lenalidomide dose adjustment (10 mg every 2 days), bortezomib 1.3 mg/m2 days 1,4,8,11, dexamethasone 40 mg weekly. Continue zolendronate.

**Rationale:**
Bortezomib safe in renal insufficiency; Lenalidomide dose adjustment for dialysis patients. Triplet regimen standard for transplant-ineligible MM. Daratumumab avoided due to COPD infusion reaction risk.

**===== run_2 =====**

**Tumor board decision:**
Start D-Vd regimen (daratumumab, bortezomib, dexamethasone) as initial therapy.

**Rationale:**
D-Vd is optimal in renal insufficiency requiring dialysis because daratumumab and bortezomib are not renally excreted. Lenalidomide requires dose adjustment and has fewer data in dialysis. D-Vd shows high efficacy in ISS-III and is safe in COPD/hypertension.

**===== run_3 =====**

**Tumor board decision:**
Initiation of D-Vd therapy (daratumumab, bortezomib, dexamethasone) as first-line therapy

**Rationale:**
In cases of renal insufficiency requiring dialysis and ISS III/R2-ISS III, bortezomib-based therapy is preferred. Daratumumab combined with bortezomib/dexamethasone (D-Vd) is evidence-based, non-renal excretory, and safe in renal insufficiency. Lenalidomide requires dose adjustment, D-Vd offers better risk stratification. COPD and hypertension are not contraindications.

# mm_case_2__ALL

################################
#### CASE: 2

################################

## ============================================================ gpt-5-mini (agent) ============================================================

**===== run_1 =====**

**Tumor board decision:**
For high-risk MM (del17p, t(4;14), R2-ISS) and only PR after first autologous SCT: recommendation for tandem autologous SCT (second high dose). Before second SCT, 1-2 cycles of consolidation with D-VRd if tolerated; subsequently risk-adapted maintenance with lenalidomide + bortezomib. Before making a decision, check MRD (NGS/flow >=10^-5) and PET/MRI and clarify stem cell reserve.

**Rationale:**

**Rationale: High-risk cytogenetics and lack of CR after first HDT suggest second auto-SCT (flowchart/guidelines). Consolidation can deepen remission before second SCT. MRD and imaging objectify the need for therapy; Len+Boron as superior maintenance in high risk. Daratumumab in maintenance only in studies/individual cases.**

**===== run_2 =====**

**Tumor board decision:**
In fit patients: carry out a second (tandem) autologous high-dose therapy/SCT. Subsequently, intensive maintenance with lenalidomide + bortezomib (+/- CD38-AK only in studies). Carry out MRD determination (NGS) and repeat FISH/extended molecular analyzes before the decision or promptly after tandem.

**Rationale:**

**Rationale: High-risk genetics (del17p, t(4;14)) and only PR after first ASCT justify more intensive consolidation; Guidelines/flowchart recommend tandem for high risk. Combination PI+IMiD as maintenance improves prognosis. MRD/repeat FISH for further risk stratification and therapy adjustment.**

**===== run_3 =====**

**Tumor board decision:**
Recommendation: For high-risk cytogenetics (del17p, t(4;14)) and only PR after first high-dose therapy -> preferred consolidation: second (tandem) autologous SCT now (stem cells present). Thereafter maintenance: lenalidomide + concomitant bortezomib (+/- daratumumab in the study context). Perform MRD (NGS/Flow) and advanced molecular panel before and after consolidation.

**Rationale:**

**Rationale: High risk (R2-ISS VI) and lack of CR after first HDT justify intensification; Guidelines/flowchart recommend tandem SCT for high risk. MRD before/after consolidation serves to decide therapy. Combination maintenance (Len+PI) improves control in high risk; CD38-AK as an option in study/in persistence.**

## ============================================================ gpt-5-mini (plain) ============================================================

**===== run_1 =====**

**Tumor board decision:**
Recommendation: Consolidation with 2 cycles of the same regimen (DVrd/VRd) for deep induction after ASCT. Subsequent high-risk maintenance: bortezomib (e.g. 1.3 mg/m2 s.c. q2w) plus lenalidomide (10-15 mg d1-21/28) until progression/intolerance. If combination is contraindicated: bortezomib monotherapy. VZV and VTE prophylaxis; Close MRD/response monitoring.

**Rationale:**
Del(17p) and t(4;14) as well as only PR after high doses show a very high risk of recurrence. Data support proteasome inhibitor-based maintenance (+/-lenalidomide) in high-risk patients to improve PFS; Consolidation increases chance of deep remission/MRD negativity. Check clinical trial, Allo not primarily recommended.

**===== run_2 =====**

**Tumor board decision:**
Due to high-risk cytogenetics (del17p, t(4;14)), we recommend tandem auto-SCT (second high-dose therapy) as the next step to deepen the remission. Then MRD determination and maintenance treatment with lenalidomide + bortezomib (PI+IMiD) until progression/intolerance. If tandem is not possible: 2 cycles of consolidation (D-)VRD before the above-mentioned maintenance. Consider clinical trials.

**Rationale:**
Patient only has PR after first auto-SCT and high-risk genes (del17p, t(4;14)). Data show that tandem AutoSCT can improve response and PFS/OS in high-risk settings. Goal: Deep remission/MRD negativity. In high-risk patients, proteasome inhibitor-assisted maintenance (bortezomib + lenalidomide) is more effective than lenalidomide maintenance alone.

**===== run_3 =====**

**Tumor board decision:**
After autologous SCT: consolidation with 2 cycles of D-VRD (or VRD) to achieve a deeper remission, then high-risk maintenance with bortezomib (e.g. 1.3 mg/m2 s.c. q7-14d) combined with lenalidomide (10-15 mg, 21/28) for >=2 years or until progression; close M-protein/FLC and MRD monitoring (q3-6M).

**Rationale:**
Patient with high-risk cytogenetics (del17p, t(4;14)) and only PR after ASCT. Lenalidomide monotherapy is suboptimal in high-risk settings; Proteasome inhibitor-based maintenance (possibly combined with IMiD) improves response/PFS. Consolidation can achieve deepening of remission. Consider clinical trial/allo option in young, fit patient.

## ================================================================ gpt-5-nano (agent) ================================================================

**===== run_1 =====**

**Tumor board decision:**
Post-ASCT MM (IgAλ, high-risk CYTO): Consolidation with triplet regimen 2-4 cycles (e.g. D-VRd) followed by MRD status check. If MRD negativity continues with lenalidomide maintenance until progression/intolerance. MRD positivity: further consolidation or antibody-based maintenance as tolerated; close MRD monitoring.

**Rationale:**
MRD negativity after ASCT is prognostically strong; High-risk genetics increase relapse risk. Consolidation deepens MRD response and PFS; Lenalidomide maintenance is an established strategy after ASCT. MRD status serves as an important decision-making anchor; Options if necessary antibody maintenance if tolerated.

**===== run_2 =====**

**Tumor board decision:**
Post-ASCT consolidation with anti-CD38 (Dara-VRd) 4 cycles; subsequent lenalidomide maintenance until progression/intolerance; Check MRD status every 3-6 months; Maintenance duration controlled by MRD, clear duration unclear.

**Rationale:**
high-risk MM; Consolidation + lenalidomide maintenance improves PFS/MRD; MRD prognostically significant; Duration of preservation unclear; MRD-controlled adaptation makes sense.

**===== run_3 =====**

**Tumor board decision:**
Posttransplant: Consolidation with DaraVRd for 2 cycles, followed by long-term maintenance lenalidomide 10 mg daily until progression or significant toxicity. Measure MRD status after consolidation. MRD negative: if necessary, observation/continuation of maintenance; MRD positive: if necessary, change in therapy (PI or BCMA approaches depending on availability).

**Rationale:**
High-risk MM (17p deletion, t(4;14)) requires deep remission. Consolidation increases CR/MRD negativity and PFS. MRD serves as a basis for decision-making for further steps. Lenalidomide maintenance provides long-lasting control; Note CD38 exposure; Also check PI/BCMA based options if available.

========================================================== gpt-5-nano (plain) ==========================================================

===== run_1 =====

**Tumor board decision:**
Lenalidomide maintenance after ASCT (R-Maintenance). Start 10 mg p.o. on days 1-21 every 28 days, continuing until progression or relevant toxicity; Dose reduction in neutro-/thrombocytopenia. Aim: Prolong PFS/OS in high-risk MM (del17p, t(4;14)).

**Rationale:**
After autologous SCT, lenalidomide maintenance is established and increases PFS/OS. High-risk cytogenetics warrant longer-term maintenance therapy. Remission status after SCT supports continuation; In case of toxicity, options (ixazomib or trial) are available.

===== run_2 =====

**Tumor board decision:**
After ASCT: 2 cycles of VRD as consolidation; subsequent lenalidomide maintenance until progression/intolerance. For high-risk cytogenetics, additional bortezomib maintenance (e.g., every 8-12 weeks) may be necessary as tolerated. Regular MRD monitoring.

**Rationale:**
High-risk MM after ASCT requires deep response. Consolidation increases depth of remission; Lenalidomide maintenance is standard; Additional proteasomal maintenance makes sense at del17p/t(4;14) depending on tolerability. MRD status allows for adaptive therapy and avoidance of unnecessary therapies.

===== run_3 =====

**Tumor board decision:**
Consolidation after SCT with 2 cycles of D-VRD (daratumumab, bortezomib, lenalidomide, dexamethasone) followed by lenalidomide maintenance until progression/intolerance; close monitoring every 3-4 months.

**Rationale:**
High-risk MM (del(17p13), t(4;14)); according to SCT PR. Consolidation increases depth of remission; subsequent lenalidomide maintenance extends PFS/survival in high-risk MMM design; low relevant comorbidities.

========================================================== gpt-oss-20b (agent) ==========================================================

===== run_1 =====

**Tumor board decision:**
1. Lenalidomide maintenance (10mgqd) until progression. 2. In case of progression: CAR-T (Ide-cél) or bispecific BCMA therapy (teclistamab). 3. Alternatively PVd, KCd or SVd as classic combinations. 4. Check participation in clinical study.

**Rationale:**
Patient PR after autologous SCT, triple class exposed, high risk. Lenalidomide maintenance is standard according to SCT. In the event of progression, CAR-T or bispecific BCMA therapies are prioritized; Fallback combinations PVd/KCd/SVd are evidence-based. Clinical studies remain an option.

===== run_2 =====

**Tumor board decision:**
After PR after autologous SCT for high-risk IgA lambda MM (t(4;14), del17p), we recommend: 1) tandem autologous stem cell transplant (melphalan 200 mg/m2) + subsequent lenalidomide maintenance. If transplant is not possible, start lenalidomide maintenance immediately. In case of progression: Daratumumab+Pomalidomide+Dex (DaraPd) or Daratumumab+Carfilzomib+Dex (DaraKd).

**Rationale:**
Patient is PR, not CR, and has high-risk genetics; Tandem SCT increases remission rates. Lenalidomide maintenance is standard post-SCT. In case of progression, CD38 + IMiD combinations (DaraPd) or CD38 + PI combinations (DaraKd) are the next sensible line.

===== run_3 =====

**Tumor board decision:**

Start ciltacabtagene autoleucel (Cilta-cel) as the next therapy.

**Rationale:**
Patient is lenalidomide refractory, PI exposed, CD38 exposed, PR after SCT, symptomatic (light chain ratio>100, osteolysis, anemia). He meets the criteria for Cilta-cel (>=1 prior line, lenalidomide-refractory, PI-exposed).

## ======================================================== gpt-oss-20b (plain) ========================================================

**===== run_1 =====**

**Tumor board decision:**
After PR after autologous SCT, a daratumumab-based salvage therapy regimen is recommended: daratumumab + pomalidomide + dexamethasone (Dara-Pom-Dex). If tolerance is good, lenalidomide maintenance can then be considered.

**Rationale:**
High-risk MM (t(4;14), del17p) with PR after SCT requires intensive salvage therapy. Dara-Pom-Dex has high response rates for relapse/progression in studies and is suitable for patients with high genetic risk factors. Maintenance with lenalidomide may prolong remission.

**===== run_2 =====**

**Tumor board decision:**
Start 3-4 cycles of lenalidomide-dexamethasone (25mg L, 20mg D, 21d/q28) as consolidation, followed by lenalidomide-maintenance (25mg d1-21/q28). Alternatively, daratumumab 16mg/kg q4w.

**Rationale:**
PR after SCT for high-risk MM (del17p, t(4;14)) requires consolidation + maintenance. L-D works on PR, improves OS, is well tolerated. Daratumumab shows significant survival benefits in PR after SCT and is recommended for high-risk patients.

**===== run_3 =====**

**Tumor board decision:**
Start lenalidomide maintenance (15mgp.o.d1-21 q28mo) if necessary with daratumumab 16mg/kg q4wk. In case of intolerance, Pomalidomide 4mgd1-21 q28. Observation with MRD monitoring.

**Rationale:**
After ASCT with PR in high-risk MM (ISSIII, 17p, t(4;14)), lenalidomide/pomalidomide maintenance improves PFS and OS. Daratumumab increases remission. MRD monitoring guides therapy.

## ======================================================== gpt-oss-120b (agent) ========================================================

**===== run_1 =====**

**Tumor board decision:**
After autologous SCT with PR and high-risk cytogenetics (del17p, t(4;14)), tandem autologous SCT is recommended as consolidation, followed by lenalidomide maintenance (10 mg daily 21 of 28 days) until progression, supplemented with weekly subcutaneous bortezomib maintenance if necessary. Ensure osteoprotection, thrombosis and infection prophylaxis.

**Rationale:**
The patient has high-risk genetics and only PR after the first HDT. According to the flowchart, a second autologous SCT is indicated for high-risk MM without CR. Lenalidomide maintenance is standard; Bortezomib maintenance can improve outcomes in del17p. Prophylaxis remains unchanged.

**===== run_2 =====**

**Tumor board decision:**
Recommendation: Perform a tandem autologous transplant (second HDT melphalan 200mg/m2) followed by lenalidomide maintenance (10mg/day, 21 of 28 days). For tolerable neuropathy, add bortezomib (1.3 mg/m2 SC weekly) to maintenance. Continue osteoprotection, thrombosis and infection prophylaxis.

**Rationale:**
The patient has high-risk cytogenetics (del17p, t(4;14)) and only PR after first ASCT. Guidelines recommend a second ASCT and more intensive maintenance for high-risk MM. Lenalidomide is standard maintenance; Bortezomib increases the remission rate in high-risk patients. No relevant

contraindications.

**===== run_3 =====**

**Tumor board decision:**
After autologous SCT with PR: 2-4 cycles of DaraVRd (or VRd) as consolidation, then tandem SCT (second autologous transplant) because of del17p/t(4;14). Thereafter, continuous maintenance with lenalidomide 10 mg daily until progression, plus osteoprotection and thrombosis prophylaxis.

**Rationale:**
High-risk genetics (del17p, t(4;14)) and only PR after first SCT justify more intensive consolidation and tandem SCT, which guidelines suggest improve response. Lenalidomide maintenance is standard for PFS increase; Daratumumab has already been used, so lenalidomide maintenance is sufficient.

## ============================================================ gpt-oss-120b (plain) ============================================================

**===== run_1 =====**

**Tumor board decision:**
After autologous SCT with PR, consistent high-risk maintenance is recommended: weekly subcutaneous bortezomib therapy (1.3 mg/m2) for 2 years combined with oral lenalidomide (10 mg/d, dose adjustment if necessary) until progression or intolerance.

**Rationale:**
The patient has high-risk cytogenetics (del17p, t(4;14)) and only PR after SCT. Bortezomib maintenance improves survival in high-risk MM, and combination with lenalidomide increases deep remission. There are no allergies or contraindications.

**===== run_2 =====**

**Tumor board decision:**
After ASCT with PR, more intensive maintenance therapy should be initiated: combination of lenalidomide (10 mg/d) and bortezomib (1.3 mg/m2 subcutaneously q-2 weeks) for at least 2 years (or until intolerance). Alternatively, daratumumab maintenance if bortezomib toxicity. Participation in high-risk study recommended.

**Rationale:**
High-risk cytogenetics (del17p, t(4;14)) and only PR after SCT increase the risk of recurrence. Double maintenance (IMiD+PI) improves progression-free survival and can deepen remission. Daratumumab is another option if PI is not tolerated.

**===== run_3 =====**

**Tumor board decision:**
After autologous SCT with PR, consistent maintenance is initiated. Combined lenalidomide (10 mg/day d1-21 q28 days) and bortezomib maintenance (subcutaneously 1.3 mg/m2 every 2 weeks) until progression or a maximum of 2 years is recommended. If bortezomib is not tolerated, daratumumab monotherapy can be used.

**Rationale:**
The patient has high-risk cytogenetics (del17p, t(4;14)) and only partial remission after SCT. In high-risk MM, double maintenance (lenalidomide+proteasome inhibitor) improves progression-free and overall survival compared to lenalidomide monotherapy. Daratumumab is a useful alternative for bortezomib toxicity.

## ============================================================ qwen3-32b (agent) ============================================================

**===== run_1 =====**

**Tumor board decision:**
Recommendation: Lenalidomide 10 mg/day as maintenance therapy after transplantation. Continue osteoprotection (e.g. denosumab) and thrombolism prophylaxis.

**Rationale:**
Maintenance therapy is indicated after autologous SCT with PR. Lenalidomide is standard according to the flowchart. High-risk genetics (t(4;14), del17p) warrant more intensive follow-up. There are no contraindications for lenalidomide.

**===== run_2 =====**

**Tumor board decision:**
Lenalidomide maintenance therapy (10 mg) is indicated after autologous stem cell transplantation and PR. Osteoprotection (bisphosphonates/denosumab) and thromboembolism prophylaxis are mandatory.

**Rationale:**
According to the flowchart, lenalidomide maintenance is the standard after high dose/transplant in high-risk patients (t(4;14), del17p13). PR does not justify further induction therapies. Osteoprotection is mandatory in myeloma with osteolysis.

**===== run_3 =====**

**Tumor board decision:**
Recommendation: Lenalidomide maintenance therapy (10 mg) after SOP. Maintenance therapy is mandatory for high-risk cytogenetics (t(4;14), del17p13). Continue regular check-ups and osteoprotection (bisphosphonates/denosumab).

**Rationale:**
After autologous SCT with PR, maintenance lenalidomide is the standard option. High-risk profiles (t(4;14), del17p13) warrant more intensive follow-up. There are no refractory criteria, therefore no combination therapy is required.

## ============================================================ qwen3-32b (plain) ============================================================

**===== run_1 =====**

**Tumor board decision:**
Recommendation: Initiate lenalidomide maintenance therapy after SOP. Schedule regular MRD monitoring and bone marrow checks.

**Rationale:**
High-risk profiles (del17p, t(4;14), ISS III) justify more intensive maintenance. Lenalidomide demonstrated better PFS in studies in high-risk MM Post-transplant PR requires MRD monitoring for early detection of recurrence.

**===== run_2 =====**

**Tumor board decision:**
Recommendation: Lenalidomide maintenance therapy after SOP. For high-risk profile and PR after transplantation, lenalidomide is the standard option. Regular MRD monitoring and bone marrow checks required.

**Rationale:**
High-risk genetics (t(4;14), del17p) and only PR after transplantation justify more intensive maintenance. Lenalidomide shows better progression-free survival in studies. No contraindications available.

**===== run_3 =====**

**Tumor board decision:**
Recommendation: Maintenance therapy with lenalidomide and bortezomib to delay progression in high-risk MM.

**Rationale:**
High-risk genetics (del17p, t(4;14)) and only PR after transplantation justify more intensive maintenance. Combination of lenalidomide and bortezomib shows better PFS in high-risk patients in studies. No contraindications available.

## ============================================================ qwen3-next-80b (agent) ============================================================

**===== run_1 =====**

**Tumor board decision:**
Second autologous stem cell transplantation (tandem SCT) indicated, followed by lenalidomide maintenance. Do not currently start any maintenance therapy.

**Rationale:**

High-risk genetics (del17p, t(4;14)) and PR after first ASCT meet high-risk criteria. Flowchart recommends a second ASCT followed by 2-cycle induction after the first ASCT (no CR) in cases of high risk before starting maintenance. Lenalidomide maintenance only after second ASCT.

**===== run_2 =====**

**Tumor board decision:**
Perform second autologous stem cell transplant, followed by lenalidomide 10 mg as maintenance

**Rationale:**
High-risk cytogenetics (17p deletion, t(4;14)) and PR after first SCT meet high-risk criteria. Flowchart envisages a second SCT after the first SCT for high-risk patients, followed by lenalidomide maintenance. Maintenance is not currently indicated as consolidation with second SCT is prioritized.

**===== run_3 =====**

**Tumor board decision:**
Second autologous stem cell transplantation (tandem SCT) indicated, followed by lenalidomide maintenance. Maintenance therapy not indicated after the first ASCT.

**Rationale:**
High-risk cytogenetics (17p deletion, t(4;14)) and PR status after first ASCT meet high-risk criteria. Flowchart recommends tandem SZT with 2 cycles of induction before maintenance. Lenalidomide alone is not sufficient after the first ASCT in high-risk patients.

## ============================================================ qwen3-next-80b (plain) ============================================================

**===== run_1 =====**

**Tumor board decision:**
Initiation of maintenance therapy with bortezomib (1.3 mg/m2 subcutaneously every 2 weeks) for at least 2 years.

**Rationale:**
Del(17p) and t(4;14) are high-risk factors. Lenalidomide monotherapy is less effective in high-risk patients. Bortezomib-based maintenance improves PFS in trials (e.g. HOVON-114) and is standard for high-risk myeloma after ASCT.

**===== run_2 =====**

**Tumor board decision:**
Initiate maintenance therapy with daratumumab (16 mg/kg IV) combined with lenalidomide (10 mg daily).

**Rationale:**
High-risk cytogenetics (del17p, t(4;14)) require intensive maintenance. Daratumumab + lenalidomide shows improved PFS in high-risk patients after ASCT in studies (GRIFFIN). PR status after transplantation requires further therapy to maintain remission. No contraindications available.

**===== run_3 =====**

**Tumor board decision:**
Initiation of maintenance therapy with daratumumab (16 mg/kg IV q4w) + lenalidomide (10 mg daily)

**Rationale:**
High-risk cytogenetics (del17p, t(4;14)) require intensive maintenance. Daratumumab + lenalidomide shows improved PFS in high-risk patients after ASCT in studies (GRIFFIN). Prior daratumumab exposure is tolerated, no contraindications.

# mm_case_3__ALL

################################
#### CASE: 3

################################

## ================================================================ gpt-5-mini (agent) ================================================================

**===== run_1 =====**

**Tumor board decision:**
Change of drug class: immediate switch to a proteasome inhibitor-based regimen without CD38 antibodies and without lenalidomide (patient is progressive under DRD). For relevant peripheral neuropathy, carfilzomib-based (e.g. KCd) is preferred; alternatively VCd or PVd if PNP is mild. Parallel rapid stem cell mobilization and preparation for autologous SCT after response.

**Rationale:**
Progression under daratumumab+lenalidomide ⇒ CD38 and lenalidomide refractory. Patient is PI-naïve (flowchart arm 2b): recommendation class switch to PI+IMiD/Alkylans and consolidation with autologous SCT if suitable. Existing chemotherapy PNP reduces bortezomib suitability -> carfilzomib preferred; no retreatment with CD38 antibodies.

**===== run_2 =====**

**Tumor board decision:**
Switch immediately to a proteasome inhibitor-based regimen, preferably carfilzomib-based (e.g. KCd: carfilzomib + cyclophosphamide + dexamethasone) or carfilzomib + pomalidomide + dexamethasone; Avoid bortezomib due to existing peripheral neuropathy. After tumor control, plan stem cell mobilization and autologous SCT.

**Rationale:**
Progression under Dara+Len -> Patient is CD38 and lenalidomide refractory. According to the algorithm, a PI-based regimen is indicated for CD38 pretreatment but PI-naïve patients. Pre-existing chemotherapy-PNP speaks against bortezomib; Carfilzomib is a suitable alternative. ASCT occurs after response; Venetoclax at t(11;14) only later/if necessary.

**===== run_3 =====**

**Tumor board decision:**
If DRD progresses after ~4 cycles: Patient is considered lenalidomide and CD38 exposed/refractory. Recommendation: immediate switch to a proteasome inhibitor-based, CD38-free regimen - preferably carfilzomib-based (e.g. KCd or carfilzomib+pomalidomide+Dex). Avoid bortezomib due to existing PNP. Parallel stem cell mobilization/collection and preparation for autologous SCT after response (PR/>=VG). Venetoclax only check later/at t(11;14) in a later line.

**Rationale:**
DRD Progress = Class switch recommended. Patient is PI-naïve -> PI-based therapy is given priority. Existing chemotherapy-induced peripheral neuropathy militates against bortezomib; Carfilzomib is therefore suitable. Goal: rapid disease control, strain collection and subsequent high-dose therapy/auto-SCT. t(11;14) for later Venetoclax option.

## ================================================================ gpt-5-mini (plain) ================================================================

**===== run_1 =====**

**Tumor board decision:**
Recommendation: Secondary therapy with a venetoclax-based regimen because of t(11;14). Preferred regimen: venetoclax + carfilzomib + dexamethasone (for significant neuropathy) or venetoclax + bortezomib + dexamethasone (for tolerable neuropathy). If venetoclax is not available/contraindicated: pomalidomide-based regimen (e.g. pomalidomide + cyclophosphamide + Dex). In parallel: immediate clarification for clinical studies/BCMA therapy (CAR-T/bispecifics).

**Rationale:**
The patient progressed after 4 cycles of DRd -> lenalidomide and anti-CD38 refractory; Repeating these classes does not make sense. The present t(11;14) indicates high venetoclax effectiveness. Peripheral neuropathy favors carfilzomib over bortezomib. Option for studies/BCMA due to early

progression and long-term perspective.

**===== run_2 =====**

**Tumor board decision:**
In case of progression to DRD and detection of t(11;14), we primarily recommend venetoclax-based therapy (venetoclax + dexamethasone). Avoid bortezomib if possible because of pre-existing peripheral neuropathy; if neuropathically tolerable: Venetoclax + Vd. If venetoclax is not available: pomalidomide + dexamethasone (+/- cyclophosphamide). Check clinical trials/BCMA therapy early.

**Rationale:**
Patient is refractory to daratumumab+lenalidomide+dex; Neuropathy speaks against bortezomib. t(11;14) speaks for venetoclax effectiveness (off-label, evidence-based). Alternatively, pomalidomide for lenalidomide refractoriness. Early study or CAR-T clarification is recommended.

**===== run_3 =====**

**Tumor board decision:**
Patient with RRMM IgA λ, progression under daratumumab+lenalidomide+Dex -> refractory to lenalidomide and CD38 antibodies. Recommendation: Start venetoclax-based therapy (e.g. venetoclax + bortezomib + dexamethasone) or, if there are contraindications, venetoclax + dexamethasone. Parallel: Evaluation for clinical study/BCMA-CAR-T or bispecific antibodies.

**Rationale:**
The presence of t(11;14) suggests BCL-2 dependence and a good venetoclax response rate. Progression under DRD makes repetition of these classes unlikely. 1q-Gain worsens the prognosis - hence the rapidly changing class with a targeted approach and testing of cell therapy options.

## ======================================================== gpt-5-nano (agent) ========================================================

**===== run_1 =====**

**Tumor board decision:**
Recommendation: Second line PI-based, preferably KCd (carfilzomib+cyclophosphamide+dexamethasone) due to existing PNP and CD38 exposure; Aim: remission for ASCT; if necessary, local radiotherapy for focal lesions; Observe prophylaxis.

**Rationale:**
DRD-Progression; Len-refraktär; CD38-exponiert; PI-naiv. Sensible therapeutic change; CAR-T/BCMA only later after exhaustion. ASCT possible if responsive; Venetoclax at t(11;14) if necessary check later.

**===== run_2 =====**

**Tumor board decision:**
second-line therapy with KCd (carfilzomib + cyclophosphamide + dexamethasone) due to lenalidomide refractoriness and CD38 exposure; Radiotherapy for focal lesions as an adjunct; Consider SCT option and, if necessary, autologous stem cell transplant after response.

**Rationale:**
DRD progression; Len-refractory; CD38 exposure; no PI pretreatment; Neuropathy prefers carfilzomib (less PN vs bortezomib); SCT option upon response; focal lesions may be addressable by RT; Observe prophylaxis.

**===== run_3 =====**

**Tumor board decision:**
PVd second-line (pomalidomide+bortezomib+dexamethasone) as a class switch from CD38-exposed, Len-refractory MM; Bortezomib SC, dose reduction if necessary; radiotherapy for focal lesions; Check SCT for response; BCMA therapy later if necessary.

**Rationale:**
Progression under DRD, CD38 exposure, Len-refractory, PI-naïve; Changing classes makes sense; SCT remains an option; Consider later BCMA/venetoclax therapies at t(11;14).

## ======================================================== gpt-5-nano (plain) ========================================================

**===== run_1 =====**

**Tumor board decision:**
Switch to pomalidomide+dexamethasone (PD) after DRD progression; No neurotoxic substances (bortezomib/thalidomide) due to neuropathy. If there is no adequate response in 2-4 cycles, evaluate transition to carfilzomib-based therapy (Kd/KPd) or transplantation.

**Rationale:**
PD is standard consequence after lenalidomide progression; reduces neuropathy risk. If there is no response, quickly switch to carfilzomib - keeps options open; Reassess ASCT as appropriate.

**===== run_2 =====**

**Tumor board decision:**
Switch to carfilzomib+dexamethasone (Kd) as next relapse therapy; no re-use of lenalidomide/daratumumab; Be aware of neuropathy. If progression, check Venetoclax+Dex due to t(11;14) or Dara-Pom-Dex.

**Rationale:**
Progression under DRD shows IMiD+Dara resistance; Kd is an effective non-IMiD-based option for lenalidomide exposure; Neuropathy favors bortezomib avoidance; t(11;14) makes venetoclax-dex useful; Dara-Pom-Dex may remain a later alternative.

**===== run_3 =====**

**Tumor board decision:**
venetoclax + dexamethasone (VD) as next line; avoid CIPN (no bortezomib); If the response is sufficient, an autologous transplant should be considered.

**Rationale:**
t(11;14) renders MM sensitive to BCL-2 inhibition; progression after DRD; CIPN impairs neurotoxic options; R-ISS 1/ISS 1; 1q gain. Goal: rapid, well-tolerated response and, if necessary, transplantation if suitable.

## ============================================================ gpt-oss-20b (agent) ============================================================

**===== run_1 =====**

**Tumor board decision:**
Nach Progression unter DRD (Lenalidomide-refraktär, CD38-AK-exponiert, PI-naiv) empfehlen wir Wechsel zu PVd (Pomalidomide + Bortezomib + Dexamethason). Consider autologous stem cell transplantation only after remission with PVd.

**Rationale:**
Patient is lenalidomide-refractory and received daratumumab; PI not yet used. ARM2b of the algorithm sets PVd as the preferred combination. Pomalidomide works despite lenalidomide resistance, bortezomib delivers high remission rate; Neuropathy risk minimized through subcutaneous administration. Transplant only makes sense after remission.

**===== run_2 =====**

**Tumor board decision:**
Discontinue DRD, switch therapy to KCd (carfilzomib+cyclophosphamide+dexamethasone) as second line. If control is inadequate, clinical trial inclusion or autologous stem cell transplantation can then be considered.

**Rationale:**
Progression after 4 cycles DRD -> lenalidomide refractory, CD38 Ab exposed, PI naïve. Chemotherapy-induced neuropathy limits bortezomib use; Carfilzomib causes fewer neuropathic side effects and is suitable for PI-naïve. KCd corresponds to Arm2b of the algorithm and offers high remission rate at t(11;14).

**===== run_3 =====**

**Tumor board decision:**
After progression after 4 cycles of DRD, therapy is not continued. Instead, a BCMA-based bispecific regimen (e.g. teclistamab) is recommended. Autologous stem cell transplantation is not considered due to lack of good response and fitness.

**Rationale:**

BCMA bispecific antibodies show ORR > 60% in DRD progression, belantamab-mafodotin-pomalidomide/DEX improves PFS, ASCT after DRD progression only with a very good response. Patient has no contraindications to BCMA therapy.

## ======================================================== gpt-oss-20b (plain) ========================================================

**===== run_1 =====**

**Tumor board decision:**
Start venetoclax 400mg daily + subk. Bortezomib 1.3mg/m2 weekly + Dexamethasone 20mg weekly (VdD) - because of t(11;14) and progression after DRD.

**Rationale:**
DRD progression, t(11;14) indicates venetoclax sensitivity. Subc. Bortezomib reduces neuropathic side effects. Combination VdD is evidence-based and recommended for R-R-MM with t(11;14).

**===== run_2 =====**

**Tumor board decision:**
We recommend switching to Venetoclax + Lenalidomide + Dexamethasone (VRd) as the next therapy. At t(11;14) venetoclax is particularly effective and the combination with lenalidomide/Dex has shown good results in R/R MM.

**Rationale:**
DRD progression after 4 cycles, t(11;14) exhibits BCL-2 high expression utilizing venetoclax. VRd is approved for R/R MM, acts independently of prior daratumumab, and addresses 1q gain risk. The patient thus remains in the active drug cycle without repeated anti-CD38 therapy.

**===== run_3 =====**

**Tumor board decision:**
Further treatment with Venetoclax (400mg) + Pomalidomide (200mg) + Dexamethasone (40mg) 4 weeks, 3 months. Alternatively, carfilzomib+pomalidomide+dexamethasone if venetoclax is contraindicated.

**Rationale:**
Progression after DRD, t(11;14) makes venetoclax effective; 1q-gain prognostically negative, but venetoclax+pomalidomide+Dex has high ORR in R/R MM. Pomalidomide is new, carfilzomib regimens are also valid.

## ======================================================== gpt-oss-120b (agent) ========================================================

**===== run_1 =====**

**Tumor board decision:**
Switch from the CD38 IMiD combination to PI-based salvage therapy. A 2-3 cycle induction regimen of carfilzomib+cyclophosphamide+dexamethasone (KCd), followed by autologous stem cell transplantation with melphalan 200 mg/m2 and pomalidomide maintenance, is recommended. If possible, check clinical studies (e.g. CAR-T).

**Rationale:**
The patient is CD38 and lenalidomide refractory, but proteasome inhibitor naive and transplant suitable. Bortezomib is unfavorable because of peripheral neuropathy; Carfilzomib has lower risk of neuropathy. KCd provides rapid tumor reduction and is compatible with high-dose therapy followed by pomalidomide maintenance.

**===== run_2 =====**

**Tumor board decision:**
Switch from CD38- and lenalidomide-based therapies. Start a PI-based salvage regime, e.g. B. Carfilzomib+cyclophosphamide+dexamethasone (KCd) as induction, followed by autologous stem cell transplantation (if transplantable) and pomalidomide maintenance. Alternatively: Enrollment in a BCMA-CAR-T or other clinical study.

**Rationale:**
The patient is progressive on daratumumab+lenalidomide (CD38 and Len refractory) and has peripheral neuropathy, so bortezomib should be avoided. Carfilzomib circumvents neuropathy risk, enables class switching, and ASCT offers curative opportunity; Pom-Maintenance makes sense after Len-resistance.

===== run_3 =====

**Tumor board decision:**
Salvage therapy with KCd (carfilzomib+cyclophosphamide+dexamethasone) followed by high-dose melphalan autologous SCT (if transplant eligible) and pomalidomide maintenance; alternatively clinical study (CAR-T/BCMA).

**Rationale:**
Progression under CD38 antibodies + lenalidomide -> class change necessary; Avoid bortezomib for peripheral neuropathy; Lenalidomide resistance, therefore no re-lenalidomide; KCd delivers potent PI without neurotoxicity; ASCT increases deep remission; Pomalidomide works after lenalidomide failure; Study option as you progress further.

## ============================================================ gpt-oss-120b (plain) ============================================================

===== run_1 =====

**Tumor board decision:**
Switch to a proteasome inhibitor-based salvage therapy (e.g. carfilzomib+pomalidomide+dexamethasone) and immediate evaluation of ASCT suitability. If t(11;14) is confirmed and after further progression, venetoclax may be considered as a targeted option.

**Rationale:**
The MM is progressive after 4 cycles of DRD, so the current class (anti-CD38+IMiD) is ineffective. A class switch to a proteasome inhibitor is standard salvage, and the good risk assessment (ISS1, R-ISS1) makes the patient suitable for possible ASCT. The presence of t(11;14) justifies the optional venetoclax construct.

===== run_2 =====

**Tumor board decision:**
Switch to a proteasome inhibitor-based 2nd line: carfilzomib+lenalidomide+dexamethasone (KRd) in 28-day cycles.

**Rationale:**
After 4 cycles of DRD there was progression, peripheral neuropathy (bortezomide toxic) and t(11;14) without high-risk mutations. KRd is highly effective in RR-MM, carfilzomib causes little neurotoxicity, lenalidomide can be used again because there is no documented resistance.

===== run_3 =====

**Tumor board decision:**
Switch to a carfilzomib-daratumumab-dexamethasone combination (KDd) as the next therapeutic step because the myeloma has already progressed after 4 cycles of DRD and there is resistance to lenalidomide. Carfilzomib has low neurotoxicity and is consistent with peripheral neuropathy. Venetoclax therapy can be considered in a clinical trial setting because of t(11;14).

**Rationale:**
DRD therapy showed progression, lenalidomide is now ineffective. KDd offers a proteasome-inhibiting, anti-CD38-based strategy without lenalidomide, suitable for patients with t(11;14) and existing neuropathy. Venetoclax is optional in studies.

## ============================================================ qwen3-32b (agent) ============================================================

===== run_1 =====

**Tumor board decision:**
Recommendation: KRd (carfilzomib + lenalidomide + dexamethasone) as 2nd line

**Rationale:**
Progression under DRD (CD38-refractory) after 4 cycles. Patient not lenalidomide refractory but CD38 and PI pretreated. KRd (ARM 3a) is suitable as it is new to lenalidomide and switches from bortezomib (neuropathy) to carfilzomib. CD38 exposure is avoided.

===== run_2 =====

**Tumor board decision:**

Recommendation: Ciltacabtagene Autoleucel (Cilta-cel) as CAR-T therapy in the 2nd line or BCMA bispecific antibodies (teclistamab) for lenalidomide refractory. Alternatively: pomalidomide + carfilzomib + dexamethasone (KCd) if not eligible for CAR-T.

**Rationale:**
Progression under DRD (DaraVRd) leads to CD38 refractory. Cilta-cel is prioritized in ARM 1 (lenalidomide and PI refractory). BCMA bispecific antibodies (teclistamab) are alternative in ARM 2c. KCd as a combination without CD38-AK is relevant for class switching. t(11;14) only allows venetoclax in late recurrence.

**===== run_3 =====**

**Tumor board decision:**
Recommendation: Clinical trial with Teclistamab (BCMA-bispecific) or KCd (Carfilzomib + Cyclophosphamide + Dex) as the next step. CAR-T (Ide-cel) not immediately suitable due to only one previous therapy.

**Rationale:**
The patient is refractory to DRD (CD38, PI, IMiD). In ARM 2c (triple-class exposure), BCMA bispecific antibodies (teclistamab) or KCd (new PI) are prioritized. CAR-T (Ide-cel) requires at least 2 previous therapies, therefore not immediately applicable. Venetoclax is reserved for later recurrences.

## ============================================================ qwen3-32b (plain) ============================================================

**===== run_1 =====**

**Tumor board decision:**
Switch to combination therapy with daratumumab, lenalidomide and dexamethasone (DAR) as a second therapeutic approach. If peripheral neuropathy is present, bortezomib-containing therapies should be avoided.

**Rationale:**
The progression after DRD therapy and the cytogenetics (t(11;14)) suggest resistance. Daratumumab combined with lenalidomide/dexamethasone provides a good response with low neurotoxicity, taking into account the existing neuropathy.

**===== run_2 =====**

**Tumor board decision:**
Switch to KCDD (carfilzomib, cyclophosphamide, dexamethasone, daratumumab) as next regimen. For progression after DRD and t(11;14), a proteasome inhibitor-containing regimen with monoclonal antibodies is useful. Preventively monitor neurotoxicity.

**Rationale:**
Progression after DRD therapy despite favorable R-ISS 1. t(11;14) suggests proteasome inhibitor sensitivity. KCDD combines synergistic mechanisms of action. Daratumumab addresses lambda-LK. Cyclophosphamide reduces neuropathy risk compared to bortezomib.

**===== run_3 =====**

**Tumor board decision:**
Start of a new induction therapy with a combination of carfilzomib, lenalidomide and dexamethasone (KRd) supplemented with daratumumab. If there is a response, a stem cell transplant should be considered.

**Rationale:**
The disease is in progression following DRD regimens. KRd with daratumumab provides high efficacy in t(11;14)-positive myeloma and minimizes neurotoxicity. The favorable prognostic factors justify an aggressive therapeutic approach.

## ============================================================ qwen3-next-80b (agent) ============================================================

**===== run_1 =====**

**Tumor board decision:**
Kd (carfilzomib + cyclophosphamide + dexamethasone) as second line therapy

**Rationale:**
Progression under DRD (CD38-AK + lenalidomide) makes the patient CD38-refractory and lenalidomide-refractory. Since no PI was pretreated (ARM 2b), a PI-based regimen without CD38-Ab is required. Due to the presence of peripheral neuropathy, bortezomib is contraindicated; Carfilzomib (in KCd) is the preferred PI alternative. No CAR-T option (ARM 1) due to lack of PI exposure. Thrombosis prophylaxis is required with dexamethasone.

**===== run_2 =====**

**Tumor board decision:**
Recommendation: KCd regimen (carfilzomib + cyclophosphamide + dexamethasone) as the next therapeutic step

**Rationale:**
Progression under DRD (CD38 and lenalidomide refractory). Do not reinsert CD38 antibodies. Presence of peripheral neuropathy makes bortezomib risky. Carfilzomib (PI) has less neurotoxicity than bortezomib and is suitable in the absence of cardiac comorbidities. KCd combines PI with alkylating agent, avoids CD38 antibodies and IMiDs. Venetoclax (t(11;14)) only when the standard options have been exhausted.

**===== run_3 =====**

**Tumor board decision:**
Recommendation: KCd regimen (carfilzomib + cyclophosphamide + dexamethasone) as the next therapeutic step

**Rationale:**
Progression under DRD (CD38-AK + lenalidomide) makes the patient CD38-refractory and lenalidomide-refractory. No PI pretreatment, hence ARM 2b. Bortezomib is contraindicated due to existing peripheral neuropathy. Carfilzomib (less neuropathic) in combination with cyclophosphamide and dexamethasone is the best option. No CD38-Ab reuse due to refractoriness.

## ============================================================ qwen3-next-80b (plain) ============================================================

**===== run_1 =====**

**Tumor board decision:**
Start of KPd (carfilzomib, pomalidomide, dexamethasone) as the next treatment step

**Rationale:**
Progression to DRD (refractory to lenalidomide/daratumumab), no PI received yet. KPd is standard option in 2nd line, effective at t(11;14). Carfilzomib less neurotoxic than bortezomib, neuropathy is monitored.

**===== run_2 =====**

**Tumor board decision:**
Switch to carfilzomib, pomalidomide, dexamethasone (KPd) as the next therapeutic step

**Rationale:**
Progression to DRD (lenalidomide + daratumumab), no prior proteasome inhibitor. Peripheral neuropathy speaks for carfilzomib instead of bortezomib. Pomalidomide possible after lenalidomide progression. t(11;14) is standard risk, KPd is evidence-based option for relapse after IMiD/anti-CD38 therapy.

**===== run_3 =====**

**Tumor board decision:**
KPd (carfilzomib, pomalidomide, dexamethasone) as second line of therapy

**Rationale:**
Progression to DRD (daratumumab/lenalidomide) precludes recurrence of these agents. KPd avoids neurotoxic bortezomib exposure, accounts for existing neuropathy. 1q gain requires aggressive therapy; Pomalidomide effective after lenalidomide progression.

# mm_case_4__ALL

################################
#### CASE: 4
################################

## ========================================================= gpt-5-mini (agent) =========================================================

**===== run_1 =====**

**Tumor board decision:**
Continuation Watch-and-Wait; no active therapy currently. Required additional findings immediately/during controls: quantitative M gradient (SPEP/IFE), serum-free light chains + ratio, complete blood count, creatinine, calcium. If there is an increase in M protein, a pathological FLC ratio, cytopenia, hypercalcemia, renal insufficiency or bone problems: bone marrow biopsy and whole-body MRI or low-dose CT/PET-CT. Control interval 6-12 months.

**Rationale:**
Mayo score 0 (low risk) and no SLiM CRAB criteria known -> no indication for therapy. Close laboratory controls and targeted imaging only when there is clinical/laboratory suspicion of progression comply with guidelines and ensure early initiation of therapy.

**===== run_2 =====**

**Tumor board decision:**
Continuation of watch-and-wait: no current systemic therapy. Close monitoring (clinical/laboratory) every 3-6 months; If there are relevant changes, reevaluate immediately and initiate therapy if SLiM/CRAB criteria are detected.

**Rationale:**

**Rationale: MGUS IgG λ with Mayo score 0 (low risk), previously w&w since 11/24. Required findings for further assessment: quantitative SPE/IFE (M gradient), serum FLC with ratio, CBC, creatinine/eGFR, Ca2+. Imaging (whole body low dose CT or spine/pelvis MRI) and contrast biopsy only for progressive/abnormal labs or symptoms. Indication for therapy when SLiM (>=60% CM-PC, sFLC ratio >=100, >1 focal MRI lesion) or CRAB criteria (anemia, hypercalcemia, renal insufficiency, osseous lesions) are met.**

**===== run_3 =====**

**Tumor board decision:**
No current indication for therapy. Recommendation: Continue watch-and-wait with close controls. Currently or in case of change: quantitative serum protein determination + immunofixation, serum-free light chain ratio, blood count, creatinine/eGFR, calcium as well as whole-body low-dose CT or spine/pelvis MRI. Bone marrow biopsy only if there is a relevant increase in M protein, a pathological FLC ratio or other CRAB/SLiM evidence.

**Rationale:**
The patient has MGUS IgGλ with Mayo score 0 and so far no evidence of SLiM-CRAB. Default: observation. Start therapy only for SLiM-CRAB (BM plasma cells >=60%, FLC ratio >=100, >1 MRI lesion or CRAB). Controls q3-6 months in the 1st year, then q6-12 months if stable.

## ========================================================= gpt-5-mini (plain) =========================================================

**===== run_1 =====**

**Tumor board decision:**
No indication for therapy. Continue watch-and-wait with monitoring: Check SPEP/IFE, free light chains (sFLC), CBC, creatinine, calcium clinically in 6 months, then annually if stable. In case of new CRAB symptoms, significant M protein increase or sFLC shift, immediate reevaluation/referral.

**Rationale:**
IgG lambda MGUS with Mayo score 0 (low risk), asymptomatic since diagnosis. No myeloma-typical complications or therapy-indicating criteria present; The standard is close observation and treatment only if there is progression.

**===== run_2 =====**

**Tumor board decision:**
No indication for drug therapy. Continuation of watch-and-wait: clinical control and laboratory (SPEP/immunofixation, serum-free light chains, blood count, creatinine, calcium) in 6 months, then annually if stable. Information about symptoms and immediate presentation if changes occur.

**Rationale:**
MGUS IgG λ with Mayo score 0 (low risk of progression), asymptomatic and no CRAB manifestations. Therapy only indicated for progression to SMM/MM; Close monitoring is sufficient for early detection.

**===== run_3 =====**

**Tumor board decision:**
No oncological therapy; Continuation of the watch and wait. Monitoring: clinical control, serum protein electrophoresis/immunofixation, serum free light chains, blood count, creatinine, calcium every 6-12 months (first year every 6 months). Urinalysis if suspected. In case of CRAB symptoms, significant increase in M protein or FLC, reevaluate immediately.

**Rationale:**
Mayo score 0 = low-risk MGUS, no organ manifestation and no previous therapy. Therefore no indication for treatment; Close monitoring for early detection of progression to SMM/MM.

## ============================================================ gpt-5-nano (agent) ============================================================

**===== run_1 =====**

**Tumor board decision:**
Continue W&W. Therapy only for SLiM-CRAB/MM progression or suspected WM/AL amyloidosis. Monitoring: SPEP/UPEP, sFLC, Ig-Quant, CBC, CMP, Ca, creatinine; urine protein; Imaging if suspected. Follow-up per risk level: 0 points annually; 1-2 points 6-12 months; 3 points 3-6 months.

**Rationale:**
MGUS IgG-Lambda, Mayo Score 0; no need for therapy. Regular checks ensure early progress detection

**===== run_2 =====**

**Tumor board decision:**
continue W&W; Therapie erst bei SLiM-CRAB-Positivität oder Progression zu MM/WM/AL-Amyloidose.

**Rationale:**
IgGλ MGUS, Mayo score 0 (low). SLiM-CRAB negative. Monitoring every 6-12 months: serum/urine M-protein, free light chain ratio (FLC), CBC, calcium, creatinine. In the case of M protein/FLC changes or CRAB symptoms, therapy according to guidelines (e.g. DaraVRd/VRd).

**===== run_3 =====**

**Tumor board decision:**
continue W&W; Therapy only for SLiM-CRAB criteria or organ-manifest MM/WM/AL amyloidosis.

**Rationale:**
MGUS IgGλ, Mayo score 0; no progression sign. Regular monitoring every 6-12 months: SPEP/IFE, Freelight Chain, CBC, calcium, creatinine, urine protein; If necessary, imaging/BM if suspected. Therapy only if an MM defining event or organ manifestation occurs (WM/AL amyloidosis).

## =========================================================== gpt-5-nano (plain) ===========================================================

**===== run_1 =====**

**Tumor board decision:**
continue W&W; MGUS IgGλ, Mayo score 0. No current therapy. Follow-up checks every 6-12 months: SPEP/IFE, free light chain ratio, CBC, Ca, creatinine; clinical assessment. In case of progression or end organ manifestation, start therapy according to guidelines.

**Rationale:**
Low risk profile (Mayo 0), no end organ findings, no complaints. MGUS does not currently require therapy; regular monitoring is sufficient.

**===== run_2 =====**

**Tumor board decision:**
Continue W&W. No therapy for MGUS IgGλ (Mayo score 0, asymptomatic). Further monitoring every 6-12 months: Hb, calcium, creatinine, CBC, M-protein, free light chain ratio; imaging clarification only in case of progression.

**Rationale:**
Low risk (Mayo 0), no CRAB/SLiM-CRAB, asymptomatic. Therapy only if there is progression to MM or amyloidosis or symptomatic disease manifestation.

**===== run_3 =====**

**Tumor board decision:**
Continuation of the watch and wait strategy; no therapy currently.

**Rationale:**
MGUS IgGλ, Mayo score 0, asymptomatic; no MM/SM progression sign; low risk. Recommendation: regular surveillance every 6-12 months (M protein, IgG, free light chain ratio, CBC, calcium, creatinine); If necessary, imaging in case of progression.

## ========================================================= gpt-oss-20b (agent) =========================================================

**===== run_1 =====**

**Tumor board decision:**
No indication for therapy - continue the watch and wait procedure with regular monitoring (e.g. every 3-6 months).

**Rationale:**
MGUS IgGLambda, Mayo Score0, no SLiM CRAB criteria, no organic damage, no indication for progression - standard MGUS management is observation.

**===== run_2 =====**

**Tumor board decision:**
No active therapy; Continuation of the watch and wait procedure with regular checks (e.g. every 3-6 months).

**Rationale:**
MGUS IgG lambda with Mayo score 0, no SLiM CRAB criteria, no organic involvement. MGUS is asymptomatic and reduced risk; Therapy is not indicated. Continuous observation is the evidence-based recommendation.

**===== run_3 =====**

**Tumor board decision:**
MGUS IgG Lambda - no indication for therapy. Continuation of the watch and wait concept with regular clinical, laboratory diagnostic and imaging checks (e.g. every 3-6 months).

**Rationale:**
MGUS with Mayo score 0, no SLiM CRAB criteria, no organic involvement or symptoms. Standard guidelines recommend observation only if the risk classification is low.

## ========================================================= gpt-oss-20b (plain) =========================================================

**===== run_1 =====**

**Tumor board decision:**
No therapy, continued observation (W&W).

**Rationale:**
MGUS IgG lambda, Mayo score 0, no organic involvement, no signs of progression or symptoms. According to current guidelines, there is no indication for therapy; regular checks recommended.

**===== run_2 =====**

**Tumor board decision:**
No therapy, continue observation (W&W).

**Rationale:**

MGUS IgG Lambda, Mayo score 0 (low risk), no symptoms, no signs of progression. Current guidelines do not recommend therapy for MGUS with score 0. Regular monitoring (serum protein, k factor, bone marrow) remains useful.

**===== run_3 =====**

**Tumor board decision:**
Further observation (watch and wait) without systemic therapy.

**Rationale:**
MGUS IgG lambda, Mayo score 0 (low risk), no symptoms, no organic involvement. Current guidelines do not recommend therapy for MGUS without evidence of progression. Regular monitoring (serum protein, k-factor, bone marrow) remains necessary.

## ======================================================== gpt-oss-120b (agent) ========================================================

**===== run_1 =====**

**Tumor board decision:**
No indication for therapy. Further procedure: Watch and wait with regular checks (serum M protein, free light chains, calcium, creatinine, imaging if necessary) every 6 months.

**Rationale:**
MGUS IgG lambda, Mayo score 0, no SLiM CRAB criteria. According to guidelines, only expectant management is indicated for low-risk MGUS; active therapy is not justified.

**===== run_2 =====**

**Tumor board decision:**
No indication for therapy - continue watch and wait with regular checks (serum protein, free light chains, blood count, calcium, creatinine every 3-6 months; if necessary, imaging if new symptoms occur).

**Rationale:**
MGUS IgG lambda, Mayo score 0, no SLiM CRAB criteria, low progression rate (<1%/year). Without clinical manifestations, active therapy is not justified; Observation is guideline standard.

**===== run_3 =====**

**Tumor board decision:**
There is currently no indication for therapy for MGUS IgG lambda (Mayo score 0) without fulfilling the SLiM CRAB criteria. Recommendation: Continuation of watch and wait with regular monitoring (serum protein electrophoresis, free light chains, calcium, creatinine, Hb) every 6 months and, if necessary, imaging if new symptoms arise.

**Rationale:**
The patient does not meet critical criteria for symptomatic myeloma; the low risk (score 0) does not justify immediate intervention. Observation allows early detection of progression to SMM or MM while avoiding unnecessary therapy toxicity.

## ======================================================== gpt-oss-120b (plain) ========================================================

**===== run_1 =====**

**Tumor board decision:**
No indication for therapy. Further pure observation (watch and wait) with serum protein electrophoresis, immunofixation and free light chain measurement every 6-12 months.

**Rationale:**
MGUS type IgG-λ, Mayo score 0 (low risk), no CRAB criterion, M protein <15g/L, stable situation since 11/24. Therapy is not indicated for low-risk MGUS; close monitoring is sufficient.

**===== run_2 =====**

**Tumor board decision:**
No indication for therapy. Further pure observation (watch and wait) with 6-monthly monitoring of serum protein electrophoresis, free light chains, blood count, calcium and kidney function.

**Rationale:**
MGUS of type IgG-λ, Mayo score 0, no CRAB criteria and no symptoms. The risk of progression is low; therapeutic intervention is not justified.

**===== run_3 =====**

**Tumor board decision:**
No indication for therapy. Further pure observation (W&W) with serum protein electrophoresis, immunofixation and free light chain analysis every 6 months.

**Rationale:**
MGUS of type IgG-λ, Mayo score 0 (low risk), no CRAB criteria, stable values so far. Therapy is not indicated for low-risk MGUS; regular checks are sufficient.

## ========================================================== qwen3-32b (agent) ==========================================================

**===== run_1 =====**

**Tumor board decision:**
No indication for therapy despite W&W strategy. Continue waiting with regular checks according to the SLiM-CRAB algorithm.

**Rationale:**
MGUS does not meet SLiM-CRAB criteria for symptomatic myeloma therapy. The Mayo prognosis score 0 (low risk) and lack of evidence of progression do not justify active therapy. W&W remains the evidence-based standard strategy.

**===== run_2 =====**

**Tumor board decision:**
No indication for therapy. Continuation of the Watch & Wait strategy with regular checks.

**Rationale:**
The MGUS IgG Lambda has a Mayo score of 0 (low risk) and no SLiM-CRAB criteria. According to the flowchart and guidelines, no active therapy is indicated for asymptomatic MGUS. W&W remains the evidence-based standard approach.

**===== run_3 =====**

**Tumor board decision:**
No indication for therapy. Continuation of the Watch & Wait strategy with regular checks.

**Rationale:**
The Mayo forecast is low (0 points), SLiM-CRAB criteria are not met. No progression or symptoms. Therapy only indicated for symptomatic myeloma.

## ========================================================== qwen3-32b (plain) ==========================================================

**===== run_1 =====**

**Tumor board decision:**
No indication for therapy. Continuation of the W&W regime with regular monitoring.

**Rationale:**
MGUS (IgG Lambda) has a low Mayo prognostic score (0), no risk of progression. Therapy only for typical myeloma progression. No contraindications or comorbidities present.

**===== run_2 =====**

**Tumor board decision:**
No indication for therapy. Continuation of the watchful waiting regime with 6-monthly controls.

**Rationale:**
The MGUS (IgG Lambda) has a Mayo prognosis score of 0, which does not require active therapy. No progression to active myeloma or complications present. Watchful waiting (W&W) remains standard therapy with a low risk profile.

**===== run_3 =====**

**Tumor board decision:**
No indication for therapy. Further observation (Watch & Wait) according to Mayo Clinic risk score 0 and stable MGUS criteria.

**Rationale:**
MGUS with low prognostic probability (0 points) and no evidence of progression. Therapy only if criteria for smoldering MM or active disease are present.

## ========================================================= qwen3-next-80b (agent) =========================================================

**===== run_1 =====**

**Tumor board decision:**
No indication for therapy. Continuation of the Watch & Wait procedure with regular checks.

**Rationale:**
MGUS is a premalignant disease without SLiM-CRAB criteria. The Mayo Clinic score of 0 indicates low risk. No symptoms, organ involvement or signs of progression. Therapy is contraindicated in MGUS; By default only monitoring is required.

**===== run_2 =====**

**Tumor board decision:**
No indication for therapy. Continuation of the watch-and-wait program with regular checks (e.g. every 6-12 months: blood count, serum protein electrophoresis, free light chains, creatinine).

**Rationale:**
MGUS is an asymptomatic precursor of multiple myeloma. SLiM-CRAB criteria (therapy indication) are not met (no anemia, hypercalcemia, renal insufficiency, bone lesions, >=60% plasma cells or light chain ratio >=100). Mayo Clinic score 0 (low risk). Therapy is contraindicated in MGUS because there is no proven benefit and the risks outweigh the risks. W&W is standard.

**===== run_3 =====**

**Tumor board decision:**
No indication for therapy. Continuation of Watch & Wait with regular checks (serological monitoring every 6-12 months, clinical assessment).

**Rationale:**
MGUS is a premalignant disease without symptoms or SLiM-CRAB criteria. The Mayo score of 0 indicates low risk of progression. Guidelines (NCCN, ESMO) do not recommend active therapy for MGUS. Therapy is only indicated in cases of progression to smoldering MM or active myeloma (SLiM-CRAB+).

## ========================================================= qwen3-next-80b (plain) =========================================================

**===== run_1 =====**

**Tumor board decision:**
No indication for therapy. Continuation of the waiting strategy (Watch & Wait) with regular checks.

**Rationale:**
MGUS is a premalignant disease with no indication for therapy. The Mayo score of 0 (low risk), absence of symptoms and no progression signals confirm the observation strategy. Therapy only if there is progression to active myeloma or symptoms.

**===== run_2 =====**

**Tumor board decision:**
No indication for therapy. Continuation of the waiting strategy (Watch & Wait) with regular monitoring.

**Rationale:**
MGUS IgG Lambda with Mayo score 0 (low risk of progression <1%/year). No symptoms, no organ involvement. Therapy for MGUS is only indicated if there is progression to symptomatic disease (e.g. myeloma). Currently no indication for active therapy.

**===== run_3 =====**

**Tumor board decision:**
No indication for therapy. Continuation of the waiting strategy (Watch & Wait) with regular clinical and laboratory monitoring.

**Rationale:**
MGUS is a premalignant disease without symptoms or organ involvement. The Mayo Clinic score of 0 indicates low risk of progression. Therapy is only indicated for progression to symptomatic myeloma or other malignancies.

# mm_case_5__ALL

################################
#### CASE: 5
################################

## ============================================================ gpt-5-mini (agent) ============================================================

**===== run_1 =====**

**Tumor board decision:**
Primary: inclusion in a clinical study - primarily MAPK-targeted studies (MEK inhibitor +/- combination) or studies with non-BCMA immunotherapies (new CAR-T, bispecific against other targets). If no study place: molecular confirmation of NRAS Q61R and, after informed consent, off-label MEK inhibitor with close monitoring. Alternatively, Selinexor-Dex or trial Allo-SCT in suitable patients.

**Rationale:**
The patient has progressed after IMiD/PI/anti-CD38, 2xASCT, BCMA-CAR-T (ide-cel) and talquetamab - renewed BCMA-directed therapy is probably less effective. NRAS Q61R activates the MAPK pathway and provides a rational basis for MEK inhibition, although with limited prospective evidence; therefore study priority, otherwise off-label only after confirmation and risk clarification.

**===== run_2 =====**

**Tumor board decision:**
Recommendation: priority for inclusion in a clinical study (priority: non-BCMA-targeted immunotherapies or studies on MAPK-targeted agents). If no study access: after MDT discussion and NGS reconfirmation of NRAS Q61R off-label therapy with a MEK inhibitor (e.g. trametinib) including complete baseline checks; alternatively palliative systemic therapy (e.g. Selinexor+dex).

**Rationale:**
Patient refractory to IMiD/PI/anti-CD38, 2x ASCT, BCMA-CAR-T (ide-cel) and GPRC5D bispecific -> further BCMA-targeted options probably limited. NRAS Q61R indicates MAPK activation; MEK inhibition is biologically plausible, but evidence is limited, hence a study priority. NGS reconfirmation and MDT/monitoring required.

**===== run_3 =====**

**Tumor board decision:**
Primary: Inclusion in a clinical trial with NRAS-targeted therapy (e.g. MEK inhibitor +/- combination) after renewed molecular confirmation. Alternatively: study-based non-BCMA immunotherapy (other CAR/bispecific). Repeat BCMA-directed therapy only if there are no alternatives and after the benefits and risks have been clarified.

**Rationale:**
Patient heavily pretreated (IMiD/PI/anti-CD38, 2x ASCT, BCMA-CAR, talquetamab) and NRAS Q61R positive. Prior BCMA-CAR reduces the success of subsequent BCMA strategies. NRAS is rational goal structure; MEK inhibitors have limited evidence, therefore study protection, molecular confirmation as well as cardio/ophthalmological baseline and close monitoring are required.

## ============================================================ gpt-5-mini (plain) ============================================================

**===== run_1 =====**

**Tumor board decision:**
Patient progressed after intensive previous therapy (2x HDM/ASCT, PI, IMiD, anti-CD38, CAR-T (ide-cel), talquetamab). Primary: Inclusion in a clinical study with a new T cell/ADC approach (preferably BCMA bispecific or alternative target GPRC5D/FcRH5 or alternative CAR-T). If no study: Salvage with pomalidomide+cyclophosphamide+dexamethasone or selinexor-based.

**Rationale:**
After multiple treatment failures, an experimental therapy with a new target is the most promising. Pomalidomide-based combination is rational standard option (pomalidomide not yet used). Selinexor is an alternative palliative option for refractory MM. NRAS(Q61R) is currently not crucial for treatment.

**===== run_2 =====**

**Tumor board decision:**
Priority: rapid forwarding and inclusion in a clinical study with novel active principles (e.g. non-BCMA / non-GPRC5D bispecific antibodies or new CAR-T, CELMoDs). If study not available: selinexor-based therapy (e.g. selinexor + pomalidomide + dexamethasone OR selinexor + bortezomib + Dex). Palliative measures: Radiation therapy for painful osteolysis, bisphosphonates/denosumab. Allo-SCT only in young, fit patients and with a suitable indication.

**Rationale:**
Patient is highly relapsed and refractory to IMiD, PI, anti-CD38, BCMA-CAR-T and GPRC5D bispecific (talquetamab) - standard options exhausted. Clinical studies with new targets/mechanisms offer the best chance. Selinexor combinations have proven activity in penta/relapsed cases. NRAS Q61R is currently not directly targetable.

**===== run_3 =====**

**Tumor board decision:**
Patient with multi-refractory MM (PIs, IMiDs, anti-CD38; BCMA-CAR-T failed; talquetamab failed): priority inclusion in clinical study with new mechanism of action (e.g. CELMoD, new bispecific/ADC or MAPK-directed studies because of NRAS Q61R). Until trial: bridging with selinexor + dexamethasone (+/- pomalidomide) or palliative cycles as tolerated.

**Rationale:**
Multiple refractory and progression after BCMA-CAR-T and GPRC5D bispecific antibody -> best chance with experimental substances. NRAS Q61R warrants review of MAPK/MEK studies. Selinexor-based regimen is a tested option for bridging in late lines of therapy.

## ============================================================ gpt-5-nano (agent) ============================================================

**===== run_1 =====**

**Tumor board decision:**
Recommendation: Participation in a clinical trial with non-BCMA/non-GPRC5D targets or novel T cell engagers; Consider allogeneic SCT if fit; Belantamab mafodotin only if BCMA antigen is expressed. Studies examining NRAS Q61R as a biomarker for MAPK access; Consider MRD status when choosing therapy.

**Rationale:**
After progression on Abecma and talquetamab, BCMA/GPRC5D options are exhausted. Non-BCMA/non-GPRC5D strategies and studies recommended. NRAS Q61R MAPK access if applicable; MRD status supports transplant/study decision.

**===== run_2 =====**

**Tumor board decision:**
Recommendation: Continue therapy with BCMA/GPRC5D-targeted approaches or participate in clinical studies (e.g. teclistamab, elranatamab, other BCMA/GPRC5D bispecifics). If available: Consider allogeneic stem cell transplantation with appropriate conditioning. Consider NRAS Q61R as an exploratory biomarker for MEK inhibition; prioritize non-BCMA options/clinical trials if available.

**Rationale:**
After progression under Abecma and talquetamab, a sequence of BCMA/GPRC5D-targeted therapies makes sense; clinical trials offer new mechanisms. NRAS Q61R biomarker unconfirmed but potentially relevant for MEK inhibition. Check Allo-SCT with appropriate conditioning; Evidence from similar cases/literature supports sequencing of new BCMA/GPRC5D therapies.

**===== run_3 =====**

**Tumor board decision:**
Recommendation: Primary participation in clinical trials with BCMA/GPRC5D targets or non-BCMA therapies; if no study available, belantamab mafodotin or selinexor-dex as a bridge. Allogeneic SCT if appropriate as a potentially curative option. NRAS Q61R is not an established biomarker; Focus on trial availability.

**Rationale:**
After progression on Abecma CAR-T and talquetamab, additional targeting options are useful; Evidence favors clinical trials in this sequence. NRAS Q61R remains unclear. Belantamab mafodotin or selinexor-Dex plausible bridging options; Allogeneic SCT if appropriate as a potentially curative

strategy. Priority: Trial availability according to current evidence.

## ============================================================ gpt-5-nano (plain) ============================================================

**===== run_1 =====**

**Tumor board decision:**
Recommendation: Enrollment in a clinical trial with a new target (e.g. BCMA or GPRC5D-based therapy) as the next step; if studio space is not available, consider belantamab mafodotin.

**Rationale:**
After CAR-T (Abecma) and talquetamab progression; heavily pretreated, few established options. Clinical study offers access to new targets, belantamab mafodotin remains a relevant second-line BCMA option.

**===== run_2 =====**

**Tumor board decision:**
Recommendation: clinical trial with new target outside BCMA (e.g. FcRH5/GPRC5D). If entry into the study is not possible, salvage with selinexor+dexamethasone (Sd). If fit, consider allogeneic transplantation.

**Rationale:**
progression after Abecma and talquetamab; NRAS Q61R; no established target therapy available. Clinical study urgently makes sense to develop new mechanisms; Sd remains an established fourth-line option if no entry into studies is possible.

**===== run_3 =====**

**Tumor board decision:**
If progression occurs during talquetamab: switch to non-BCMA/non-GPRC5D-targeted systemic therapy. Suitable: Pomalidomide-dexamethasone (possibly +/- bortezomib) or participation in a clinical trial with a new target. Radiotherapy of focal osteolysis; Continue antiresorptive medications and supportive care.

**Rationale:**
After CAR-T and progression under GPRC5D bispecific, further BCMA/GPRC5D therapies are limited. Pomalidomide/Dexa (with/Before Bortezomib) Provides Established Activity in RRMM; clinical study useful; Radiotherapy for local symptoms. Care according to comorbidities.

## ============================================================ gpt-oss-20b (agent) ============================================================

**===== run_1 =====**

**Tumor board decision:**
Start trametinib 2mg p.o. daily (monotherapy or in combination with proteasome inhibitor, e.g. carfilzomib). If not available, consider BCMA-bispecific (teclistamab/elranatamab) or belantamab-mafodotin triplet in clinical trial.

**Rationale:**
NRASQ61R activates MEK signaling; Trametinib has shown ORR in a similar case after talquetamab progression and can be used as monotherapy or with proteasome inhibitor. BCMA bispecifics and belantamab mafodotin provide additional options when trametinib is not accessible.

**===== run_2 =====**

**Tumor board decision:**
Recommendation: A BCMA-related bispecific (e.g. teclistamab or elranatamab) as the next therapeutic unit, ideally in a registered phase 2 study. In parallel to the available MEK inhibitor study (trametinib) for NRAS-Q61R, a compassionate use request can be submitted if no BCMA option is available.

**Rationale:**
The patient has already undergone CAR T cell therapy (Abecma) and talquetamab without success; BCMA bispecifics show ORR of 55-61% in pre-CAR-T patients. NRAS Q61R mutation provides a targeted option with trametinib, as reported in a similar case. Combinations or sequences of these approaches have been identified as promising in recent studies.

**===== run_3 =====**

**Tumor board decision:**
Recommendation: Start a BCMA bispectral (e.g. teclistamab) as the next monotherapy. In parallel with participation in a clinical trial with the MEK inhibitor trametinib (NRASQ61R targeted) for potential synergy. If intolerance, consider belantamab-mafodotin + Pom/Dex.

**Rationale:**
The patient is in PD after CAR T cell and talquetamab therapy. Literature shows 45-61% ORR with BCMA bispecific as monotherapy, even after BCMA CAR T failure. NRASQ61R mutation provides rational basis for MEK inhibitor (trametinib) - evidence from 5 PubMed studies. Belantamab-mafodotin with Pom/Dex is an alternative option for lenalidomide resistance.

## ============================================================ gpt-oss-20b (plain) ============================================================

**===== run_1 =====**

**Tumor board decision:**
Start belantamab-mafodotin 2.5mg/kg Q3W with ophthalmological control (after CAR-T and talquetamab progression).

**Rationale:**
Patient has PD after 8 previous lines (including 2 HDM/ASCT, lenalidomide, DKCD, KD, IBD, KPD, CAR-T, talquetamab). Belantamab is the only approved monotherapy with a proven activity profile after CAR-T therapy. NRAS mutation does not affect standard therapy. Ophthalmological monitoring minimizes risks.

**===== run_2 =====**

**Tumor board decision:**
Start belantamabmafodotin 2.5mg/kg IV q3weeks until progression or untenable side effects. If inappropriate, selinexor+dexamethasone as an alternative second-line approach.

**Rationale:**
Patient is refractory to 7-line therapies (including BCMA-CAR-T). Belantamab remains the preferred BCMA-targeted option with demonstrated activity after CAR-T therapy. Selinexor-dexamethasone is available as a backup option when belantamab is contraindicated or unavailable.

**===== run_3 =====**

**Tumor board decision:**
Start belantamab mafodotin 2.5mg/kg IV q3wks; if contraindicated, consider daratumumab+pomalidomide+dexamethasone. Parallel trial enrollment.

**Rationale:**
Patient progressed after BCMA-CAR-T; belantamab is a BCMA-targeted ADC with proven activity post-CAR-T and is approved after >=4 lines. DPd is an alternative if belantamab is unsuitable. Trial enrollment offers additional novel options.

## ============================================================ gpt-oss-120b (agent) ============================================================

**===== run_1 =====**

**Tumor board decision:**
We recommend that the patient be transferred immediately to a clinical study arm with a MEK inhibitor (e.g. trametinib+/-lenalidomide) for NRAS-Q61R-MM. If no study space is available, BCMA bispecific antibody therapy (e.g. teclistamab) should be used as the next line. With suitable fitness and a suitable donor, allogeneic stem cell transplantation can be considered as a potential curative option after response.

**Rationale:**
The patient is progressive after two autologous SCT, lenalidomide, multiple proteasome inhibitor/immunomodulator combinations, BCMA-CAR-T and talquetamab. The NRAS Q61R mutation warrants RAS pathway blockade; Literature and a similar case support MEK inhibitors. BCMA bisspecifics show effectiveness after CAR-T failure. Allogeneic SCT remains a potential curative option for transplant-eligible patients.

**===== run_2 =====**

**Tumor board decision:**
Recommendation: Participation in a clinical trial with a MEK inhibitor-based combination (e.g. trametinib+pomalidomide+dexamethasone) due to NRAS Q61R mutation. If no study place is available, immediate treatment with the BCMA bispecific teclistamab (or elranatamab) according to the current label. Allogeneic transplantation only with suitable performance status and suitable donor.

**Rationale:**
The patient is progressive after two autologous SCTs, multiple IMiD, PI, anti-BCMA and GPRC5D therapies. NRAS-Q61R activates the MAPK pathway; Evidence from similar cases and current literature supports MEK inhibition. BCMA bisspecifics show effectiveness after CAR-T failure. Allogeneic SCT remains an option if you are in good condition.

**===== run_3 =====**

**Tumor board decision:**
Recommendation: Participation in a clinical trial with a MEK inhibitor (e.g. trametinib) because of the NRAS Q61R mutation. If no study is available, next therapy with a BCMA bispecific antibody (teclistamab/elranatamab) or belantamab mafodotin. Allogeneic stem cell transplantation only if the candidate is suitable.

**Rationale:**
The patient has progressive MM after two autologous SCTs, lenalidomide maintenance, multiple proteasome and anti-CD38 therapies, CAR-T (Abecma) and talquetamab. The NRAS Q61R mutation activates the MAPK pathway; a MEK inhibitor showed effectiveness in a comparable case and is supported in studies. BCMA bispecific antibodies and belantamab are effective after CAR-T, while allo-SCT is only considered a last option if the performance is good and the donor is suitable.

## ============================================================== gpt-oss-120b (plain) ==============================================================

**===== run_1 =====**

**Tumor board decision:**
Recommendation: Further therapy with belantamab-mafodotin (BCMA-ADC) or, if already used/contraindicated, teclistamab (BCMA-bispecific) in combination with dexamethasone. Alternatively, participation in a clinical study protocol (e.g. selinexor combination, new CAR-T constructs).

**Rationale:**
Patient has progressive myeloma after 2 ASCT, lenalidomide maintenance, multiple proteasome inhibitor and immunomodulator combinations, CAR-T (BCMA) and talquetamab (GPRC5D). Have not yet received BCMA ADC or BCMA bispecifics; these show effectiveness after CAR-T failure. Clinical trial provides access to innovative options.

**===== run_2 =====**

**Tumor board decision:**
Initiate BCMA-targeted therapy with belantamab-mafodotin (alternatively teclistamab) and consider inclusion in an appropriate clinical trial protocol; further autologous transplantation is not indicated.

**Rationale:**
The patient has progressive, triple-refractory myeloma after 2 ASCT, lenalidomide, proteasome inhibitors, daratumumab, CAR-T and talquetamab. BCMA-ADC is proven to be effective in this situation and has not yet been used; other options should be evaluated in studies.

**===== run_3 =====**

**Tumor board decision:**
Recommendation: Participation in a clinical study protocol with a BCMA bispecific antibody (e.g. teclistamab) or a MEK inhibitor trial due to NRAS mutation; alternatively belantamab-mafodotin as an approved option.

**Rationale:**
Patient is triple class refractory (proteasome inhibitor, IMiD, anti-CD38) and has already received BCMA-CAR-T and GPRC5D bispecific, but progression. Other established options are limited; a BCMA bispecific or a targeted MEK inhibitor (NRAS Q61R) in a study offers the best prospects. Belantamab-mafodotin is the last approved therapy available.

## ============================================================ qwen3-32b (agent) ============================================================

**===== run_1 =====**

**Tumor board decision:**
Recommendation of MEK inhibitor treatment (e.g. trametinib) as a new approach to targeting the NRAS Q61R mutation. In addition, BCMA-CD3 bispecifics (teclistamab/elranatamab) or belantamab-based combinations should be examined.

**Rationale:**
The NRAS mutation is responsible for resistance mechanisms. MEK inhibitors directly address the RAS signaling pathway. Despite the mutation, bispecifics offer a final option for refractory myeloma. Both approaches are documented in the literature and similar cases.

**===== run_2 =====**

**Tumor board decision:**
Recommendation: MEK inhibitor (e.g. trametinib) as the next therapeutic step in combination with a proteasome inhibitor or immunomodulator. Clinical studies of MEK inhibitors in NRAS-mutated myelomas need to be urgently examined.

**Rationale:**
The NRAS Q61R mutation suggests a RAS/MEK/ERK pathway disorder. MEK inhibitors show initial effectiveness in BCMA resistance in similar cases and case reports. CAR-T and BCMA bispecific relapse require new targets. Despite limited evidence, MEK inhibitors are the strongest option in mutation and multi-drug resistance.

**===== run_3 =====**

**Tumor board decision:**
Recommendation: MEK inhibitor (e.g. trametinib) in combination with a bispecific antibody (e.g. teclistamab) or participation in clinical trials of RAS-targeted therapies.

**Rationale:**
The NRAS Q61R mutation suggests RAS/MEK pathway activation, for which MEK inhibitors have been recommended in similar cases. Bispecific antibodies (e.g. teclistamab) are mentioned in the current literature as an option in refractory myeloma, although direct data on the mutation are not available. Combinations or clinical trials offer new approaches to therapy resistance.

## ========================================================== qwen3-32b (plain) ============================================================

**===== run_1 =====**

**Tumor board decision:**
Recommendation of a clinical trial with NRAS-targeted therapy or XMT1522 (GPRC5D-ADC) in recurrent, multidrug-resistant myeloma after CAR-T progression.

**Rationale:**
Patient with multiple relapsed, multi-resistant myeloma (lenalidomide, PI, CAR-T resistance) and NRAS mutation. Default options exhausted. XMT1522 as an alternative ADC or NRAS-targeted approaches in studies offer the latest therapy options.

**===== run_2 =====**

**Tumor board decision:**
Recommendation: Therapy with teclistamab (BCMA bispecific antibody) or initiation of clinical trials with MEK inhibitors (e.g. trametinib) due to NRAS mutation status.

**Rationale:**
The patient is refractory to PI, IMiD, CAR-T and GPRC5D targeting. The NRAS mutation (Q61R) suggests MEK targeting therapy. Teclistamab (BCMA-bispecific) is suitable as a last option after CAR-T progression.

**===== run_3 =====**

**Tumor board decision:**
Recommendation: Participation in a clinical trial with a MEK inhibitor (e.g. trametinib) in combination with dexamethasone or alternatively a BCMA bispecific antibody therapy (e.g. teclistamab) due to the NRAS mutation status and multidispersive course of therapy.

**Rationale:**
The patient showed progression on all standard therapy classes (PI, IMiD, CAR-T). The NRAS Q61R mutation suggests RAS pathway activation that can be addressed by MEK inhibitors. BCMA bispecific antibodies are an established option in multidispersive cases. Both approaches have been evaluated in clinical studies.

## ========================================================= qwen3-next-80b (agent) ==========================================================

**===== run_1 =====**

**Tumor board decision:**
Teclistamab (BCMA bispecific antibody) indicated as the next treatment option.

**Rationale:**
Patient is triple-class exposed (IMiD, CD38-AK, PI) and has progressed under CAR-T (Abecma) and talquetamab (GPRC5D-bispecific). Since there is no BCMA bispecific therapy, teclistamab is prioritized. NRAS mutation does not affect the election. Alternatives such as Selinexor or PCd are less effective. Clinical studies must also be examined.

**===== run_2 =====**

**Tumor board decision:**
Selinexor + dexamethasone (Sd) as the next treatment option, possibly in combination with a clinical trial.

**Rationale:**
Patient is triple-class refractory (IMiD, PI, CD38-AK), after progression under CAR-T (Abecma) and GPRC5D bispecific antibody (Talquetamab). Selinexor is approved for >=4 prior therapies and effective in severely refractory MM. NRAS mutation does not influence the choice as no specific target therapy exists. Clinical studies (ARM 4) must be examined in parallel.

**===== run_3 =====**

**Tumor board decision:**
Selinexor 80 mg weekly + dexamethasone 20 mg (once weekly) as the next line of therapy.
Alternatively: inclusion in clinical studies (e.g. BCMA or GPRC5D-based therapies in development).

**Rationale:**
Patient is progressive after CAR-T (Abecma) and talquetamab and triple-class refractory (IMiD, PI, CD38-AK). Selinexor as an XPO1 inhibitor offers a new class of drugs without overlap with previous therapies. NRAS Q61R mutation is not a contraindication. Clinical studies must always be examined due to the high burden of recurrences.

## ========================================================= qwen3-next-80b (plain) ==========================================================

**===== run_1 =====**

**Tumor board decision:**
Initiation of teclistamab (BCMA-targeted bispecific antibody therapy) in combination with dexamethasone

**Rationale:**
After progression on CAR-T (Abecma) and talquetamab (GPRC5D), teclistamab as a BCMA-targeted option is the best available therapy. Studies show response even after CAR-T progression. No contraindications (no allergies, NRAS mutation does not affect effectiveness). Alternatives such as Selinexor are less effective in this line.

**===== run_2 =====**

**Tumor board decision:**
Initiation of selinexor (80 mg weekly) in combination with dexamethasone (20 mg weekly) as the next line of therapy

**Rationale:**
Patient has undergone all common therapies (PI, IMiD, anti-CD38, CAR-T, GPRC5D bispecifics). Selinexor is approved for multiple relapsed MM after >=4 lines. NRAS mutation does not affect the election. No known contraindications. Combination offers effective option with limited remaining

therapies.

**===== run_3 =====**

**Tumor board decision:**
Initiation of selinexor 80 mg once weekly plus dexamethasone 20 mg weekly

**Rationale:**
Patient has progression on multiple lines of therapy (BCMA-CAR-T, talquetamab, PI, IMiD, anti-CD38). Selinexor is approved for refractory myeloma after >=4 lines of therapy and offers a new mechanism of action (XPO1 inhibition). NRAS mutation does not affect the election. No known contraindications.

# mm_case_6__ALL

################################
#### CASE: 6

################################

## ================================================================ gpt-5-mini (agent) ================================================================

**===== run_1 =====**

**Tumor board decision:**
Recommendation: Priority for BCMA-directed therapy. If suitable and available, CAR-T (cilta/ide-cel) preferred; If CAR-T is not possible/waiting time is too long: bispecific anti-BCMA (e.g. teclistamab). Pomalidomide/selinexor only if BCMA options are not available or studies are not possible. Avoid bortezomib due to drug-induced PNP.

**Rationale:**
Patient is triple-class exposed (lenalidomide, PI, CD38) and lenalidomide-refractory -> according to the algorithm BCMA therapies are given priority. Dialysis is not an absolute contraindication criterion, but requires coordination close to the center (adapted lymphodepletion for CAR-T). The 3 mm pulmonary nodulus is not a contraindication per se, but a short CT/infection assessment is recommended before T cell therapy.

**===== run_2 =====**

**Tumor board decision:**
Recommendation: BCMA-directed therapy as a priority. If suitable and available, CAR-T (cilta-/ide-cel) preferred; if not available/not suitable, bispecific anti-BCMA antibody (e.g. teclistamab/elranatamab) or study inclusion. Pomalidomide/selinexor only if BCMA options are not available.

**Rationale:**

**Rationale: Patient is post-ASCT, lenalidomide exposed/refractory and currently triple class exposed (IMiD, PI, CD38) -> BCMA therapies priority according to the algorithm. Dialysis is not an absolute contraindication. Before start: complete infection serology, cardiac evaluation, CT comparison/PET clarification of the 3 mm nodule, coordination of dialysis with apheresis/lymphodepletion; PNP speaks against renewed bortezomib intensification.**

**===== run_3 =====**

**Tumor board decision:**
BCMA-directed therapy is preferred. If treatment is needed immediately and is available: off-the-shelf bispecific antibodies (e.g. teclistamab/elranatamab). If suitable and possible waiting period: CAR-T (e.g. cilta/ide-cel) as target therapy. Alternative: pomalidomide- or selinexor-based combination, or study participation. Before starting: nephrology clarification (dose/timing) and CT chest to clarify the 3 mm nodule.

**Rationale:**
Patient is triple class exposed, post-ASCT with serological progression; Current evidence favors BCMA-directed approaches for high ORR and deep remissions. Bispecific antibodies offer rapid availability; CAR-T better long-term remission if suitable. Dialysis is not an absolute contraindication, but requires nephrological dose adjustment and infection prophylaxis. Small pulmonary nodule is not a necessary obstacle, but should be clarified before starting therapy.

## ================================================================ gpt-5-mini (plain) ================================================================

**===== run_1 =====**

**Tumor board decision:**

**Tumor board decision: Starting pomalidomide + cyclophosphamide + dexamethasone (PCd) therapy with dialysis-adapted dosage; Avoid bortezomib due to drug-induced PNP. If cardiac suitable, alternatively carfilzomib + pomalidomide + dexamethasone (KPd). Parallel presentation at the center for BCMA-directed therapy/studies.**

**Rationale:**

Patient is exposed to lenalidomide and daratumumab with serological progression. Pomalidomide is effective in Len-refractory MM and can be used in dialysis; Cyclophosphamide increases effectiveness. Avoid bortezomib due to existing PNP. KPd offers higher activity with good cardiac function. BCMA options/studies should be explored.

**===== run_2 =====**

**Tumor board decision:**
Primary: rapid referral to BCMA-directed therapy (CAR-T or approved BCMA bisspecific/antibody) due to refractory course after lenalidomide and DVd. Alternative if not available/contraindicated: pomalidomide + dexamethasone +/- low-dose cyclophosphamide (avoid bortezomib due to PNP). Dose adjustment for dialysis, infectious prophylaxis.

**Rationale:**
Patient with R2-ISS III, progression after lenalidomide maintenance and 15 cycles of DVd -> functionally refractory to IMiD and CD38-directed therapy/PI-exposed. BCMA-directed therapies offer the highest probability of remission. If unavailable, Pomalidomide (effective in dialysis) + Dex +/- Cyclo is a pragmatic, neuropathy-sparing option.

**===== run_3 =====**

**Tumor board decision:**
Recommendation: Rapid presentation/prior evaluation for BCMA-directed therapy (preferably CAR-T: ide-cel or cilta-cel). If CAR-T is not available at short notice: BCMA-bispecific (e.g. teclistamab). Until definitive therapy, bridging with pomalidomide + cyclophosphamide + dexamethasone (PCd). Do not restart bortezomib and daratumumab; Pay attention to dose adjustment during dialysis.

**Rationale:**
Patient is serologically progressive after lenalidomide (maintenance) and 15 cycles of DVd -> triple-class/exposed and R2-ISS III (high risk). BCMA therapy has the highest chance of deep remission. Bridging with a pomalidomide-based regimen is suitable for dialysis; Avoid bortezomib due to treatment-induced PNP.

## ============================================================ gpt-5-nano (agent) ============================================================

**===== run_1 =====**

**Tumor board decision:**
BCMA-driven therapy preferred as next line; Ide-cel CAR-T, if bridging, logistics and suitability are given; alternatively BCMA bispecific (teclistamab/elranatamab). If BCMA therapy is not possible, pomalidomide-based combination regimen or clinical trial; Consideration of dialysis status, lung findings and performance status.

**Rationale:**
After progression on DVd after autologous transplantation/lenalidomide maintenance, BCMA therapies offer the highest activity in multiline situations, including in renal insufficiency. CAR-T vs bispecifics depending on logistics. Prefer clinical studies. Pomalidomide option remains backup; Logistics and Tox must be taken into account.

**===== run_2 =====**

**Tumor board decision:**
Preferred BCMA-driven therapy: CAR-T (cilta-cel) as first-line option for triple-class-exposed, lenalidomide-refractory, dialysis-requiring MM; alternatively Ide-cel. If BCMA therapy is not possible in a timely manner or the patient is unsuitable: pomalidomide-based combinations (PVd) or clinical trial; Consideration of kidney status, lung findings and performance status.

**Rationale:**
Progression under Lenalidomide+DVd despite Len maintenance; Len-refractory + PI+CD38 exposure, requirement for dialysis; BCMA therapies offer the highest response rate in this situation. Pulmonary nodules small; Patient capable enough for cell-based therapy. Pay attention to center logistics; check clinical trial.

**===== run_3 =====**

**Tumor board decision:**
Priority CAR-T therapy against BCMA (Cilta-cel) in lenalidomide-refractory, PI-exposed MM after >=2 previous therapies despite renal insufficiency requiring dialysis. If Cilta-cel is not available/suitable: Ide-cel; alternatively BCMA bisspecifics teclistamab or elranatamab/talquetamab.

Bridging therapy for delay: PVd or KCd; review clinical trials.

**Rationale:**

**Rationale: Patient triple-class exposed (IMiD, PI, CD38); Len-refractory + dialysis; Cilta-cel offers the highest remission rate in this line. Non-CD38 arguments present. If CAR-T is delayed, alternative BCMA therapies or PVd/KCd; Check study options.**

## ========================================================= gpt-5-nano (plain) =========================================================

**===== run_1 =====**

**Tumor board decision:**
Switching to DPd (daratumumab + pomalidomide + dexamethasone) as the next treatment step for lenalidomide-refractory MM requiring dialysis.

**Rationale:**
progression on lenalidomide; no high-risk cytogenetics; DPd shows established activity after lenalidomide refractoriness; Pomalidomide safe on dialysis; avoids bortezomib neuropathy; Anti-CD38 increases response rates.

**===== run_2 =====**

**Tumor board decision:**
Use pomalidomide + dexamethasone (Pd) as the next line of RRMM. If well tolerated, possibly PVd (Pd + bortezomib + dexamethasone). Alternatives: Belantamab mafodotin or selinexor + dexamethasone or participation in a clinical trial.

**Rationale:**
RRMM after DVd and lenalidomide maintenance; ISS/R2-ISS III; no high-risk genetics detectable. Pd-based therapies are standard after IMiD and anti-CD38 exposure. Treatment choice depending on tolerability, renal status and availability; check clinical trial.

**===== run_3 =====**

**Tumor board decision:**
Daratumumab + pomalidomide + dexamethasone (D-Pd) as next step. 28-daily cycles: Daratumumab 16 mg/kg IV (wk 1-2, then q2w), pomalidomide 4 mg p.o. days 1-21, dexamethasone 40 mg p.o./iv. days 1,8,15,22. Adjust dosage if cytopenias occur. consideration of renal insufficiency; If necessary, check CAR-T option as bridging.

**Rationale:**
Relapse after lenalidomide maintenance and DVd; no high-risk cyto gene; Pomalidomide sensitive despite renal insufficiency; D-Pd suitable for dialysis and neuropathy prevention; enables quick control and, if necessary, bridge to CAR-T if available.

## ========================================================= gpt-oss-20b (agent) ==========================================================

**===== run_1 =====**

**Tumor board decision:**
Idecabtagene vicleucel (CAR-T) as next line after progression to DVd, considering triple class exposure, renal insufficiency and PNP risk. Alternatively, teclistamab (BCMA-bispecific) if CAR-T is not available.

**Rationale:**
Patient is CD38 Ab refractory, PI exposed, lenalidomide sensitive - Arm2c. CAR-T (Ide-cel) offers high response rates, is not renally eliminated and avoids bortezomib-induced PNP. Bispecifics are the second option if CAR-T is not available.

**===== run_2 =====**

**Tumor board decision:**
Recommendation: initiation of BCMA-CAR-T therapy (idecabtagene vicleucel) as the next line. If CAR-T is not available, alternative bispecific BCMA T cell redirector (e.g. teclistamab).

**Rationale:**

Patient is lenalidomide, CD38 and PI refractory (triple class exposed) and has CKD5/dialysis - CAR T/T cell therapies are kidney independent and offer maximum effectiveness. 3mm nodulus is stable, no contraindications for CAR-T. Alternative bispecific if CAR-T is available.

**===== run_3 =====**

**Tumor board decision:**
Start BCMA-targeted bispecific T-cell engager (teclistamab) as the next therapy. In severe renal insufficiency and dialysis, the dose is unchanged; CRS monitoring and eye toxicology required.

**Rationale:**
DVD progression requires BCMA approach. Teclistamab shows 60-70% ORR, good tolerability in CKD5, no renal clearance dependence. Literature and comparable cases confirm effectiveness and safety in patients requiring dialysis.

## ============================================================ gpt-oss-20b (plain) ============================================================

**===== run_1 =====**

**Tumor board decision:**
Start carfilzomib 56mg/m2 (days1,2,8,9,15,16) + dexamethasone 20mg (days1,8,15,22) (Kd regimen) with optional daratumumab addition (Dara-Kd) taking CKD-5 dialysis into account. Dose adjustment for renal insufficiency.

**Rationale:**
RRMM after DVd, CKD-5, no renal exclusion for carfilzomib. Kd shows a high response rate in high-risk MM and is renal-friendly. Daratumumab can be supplemented if necessary. Pomalidomide would be an alternative, but carfilzomib would be preferred because of the lack of renal exclusion.

**===== run_2 =====**

**Tumor board decision:**
Start Daratumumab + Carfilzomib + Dexamethasone (DKD) as next therapy, adapted to dialysis (Carfilzomib 56mg/m2, Daratumumab 16mg/kg, Dexamethasone 40mg).

**Rationale:**
DKD is effective in RR-MM, is well tolerated in CKD-5, carfilzomib does not require a renal dose, daratumumab is dialysis-independent. Rapid control of progression and good bone stabilization possible.

**===== run_3 =====**

**Tumor board decision:**
We recommend switching to KPd (carfilzomib 56mg/m2 i.v. 2x/wk, pomalidomide 2mgqd. dialysis-adjusted, dexamethasone 20mgqd. 3wk/4wk). Parallel bisphosphonates (zoledronate 4 mg IV at 3-month intervals) and local radiotherapy for HWK4 osteolysis. Pulmonary nodulus by pulmonological control.

**Rationale:**
After 15 cycles of DVd, the patient is serologically progressive and has resistance to tumumab. KPd is suitable in CKD5 without dialysis, pomalidomide in dialysis 2mg qd. Carfilzomib not renal relative. R2-ISS-High-Risk requires intensive combination. Bisphosphonates and RT address bone and symptom burden. Pulmonary nodule is monitored by pulmonology.

## ============================================================ gpt-oss-120b (agent) ============================================================

**===== run_1 =====**

**Tumor board decision:**
We recommend BCMA-directed therapy as the next step. Idecabtagene vicleucel (CAR-T) is the preferred option in triple class refractory MM after autologous transplantation, dialysis dependence and peripheral neuropathy. Alternatively, a BCMA bispecific antibody (e.g. teclistamab) can be used.

**Rationale:**
The patient is lenalidomide, daratumumab and bortezomib refractory, has already had an autologous transplant and is at high R ISS III risk. BCMA therapies show high response rates in this setting and circumvent the neurotoxic risks of bortezomib. Dialysis does not affect effectiveness.

**===== run_2 =====**

**Tumor board decision:**
Recommendation: Implement BCMA-targeted CAR-T therapy (e.g. Ciltacabtagene autoleucel) as the next line. If not available: Ide-cel or bispecific anti-BCMA antibody (teclistamab) as an alternative. Bridging therapy with Pomalidomide+Dex, osteoprotection and dialysis management.

**Rationale:**
Patient is triple class exposed (IMiD, CD38 Ab, PI) and lenalidomide refractory, has progressive serological MM despite DVd, high risk (R-ISSIII). According to the algorithm, with such exposure, a BCMA-CAR-T option (Arm1) is priority, effective and renally tolerated, and outperforms classic combinations.

**===== run_3 =====**

**Tumor board decision:**
Due to serological progression after lenalidomide and CD38 antibody therapy (DVd) as well as PI refractoriness and renal insufficiency requiring dialysis, we recommend a BCMA-directed CAR-T therapy (e.g. Ciltacabtagene autoleucel; alternatively Idecabtagene vicleucel) or, if not available, a BCMA bispecific as the next line Antibodies (teclistamab).

**Rationale:**
The patient is triple-class refractory, high-risk MM (R2-ISSIII) and has no contraindication to CAR-T; Dialysis is not an exclusion criterion. CD38 refractoriness prohibits repeat CD38 blocking, and bortezomib is unsuitable because of peripheral neuropathy. BCMA therapies offer the highest expected effectiveness.

## ============================================================ gpt-oss-120b (plain) ============================================================

**===== run_1 =====**

**Tumor board decision:**
Start carfilzomib-dexamethasone (Kd) therapy with adjusted dose for dialysis patients as next line. Monitor closely during cardiovascular stress; If progression continues, consider BCMA-directed options (e.g. belantamab or CAR-T).

**Rationale:**
Patient is serologically progressive after ASCT, lenalidomide maintenance and 15 cycles of DVd (daratumumab-based). Previous therapies, including bortezomib, are ineffective or fraught with neuropathy. Carfilzomib works without neurotoxic side effects and is safe on dialysis. Hence the most suitable next option.

**===== run_2 =====**

**Tumor board decision:**
In the case of serological progression after lenalidomide maintenance and DVd therapy, pomalidomide-based therapy is recommended as the next step: pomalidomide 4 mg (dialysis-adjusted) + dexamethasone 40 mg weekly, if necessary, continuation of daratumumab (DPd) or alternatively pomalidomide-dexamethasone alone. Additionally denosumab for bone protection.

**Rationale:**
The patient requires dialysis, has bortezomib-induced neuropathy and is already exposed to lenalidomide. DPd is approved after IMiD and PI failure, works independently of renal function and avoids neurotoxic drugs. Further options (CAR-T, belantamab) are reserved for later lines.

**===== run_3 =====**

**Tumor board decision:**
Start of a pomalidomide-based salvage therapy (pomalidomide 2-4mg/dayd1-21 + dexamethasone 40mg weekly, optionally cyclophosphamide 500mg/m2 d1-2) taking into account the requirement for dialysis. Check clinical trial/BCMA therapy.

**Rationale:**
Patient is refractory to autologous transplantation, lenalidomide maintenance, and 15 cycles of DVd (bortezomib-daratumumab-dexamethasone). Pomalidomide is safe in dialysis, works independently of previous proteasome inhibitors and offers an effective option before BCMA therapies.

## ============================================================ qwen3-32b (agent) ============================================================

**===== run_1 =====**

**Tumor board decision:**
Recommendation: BCMA bispecific antibody (teclistamab/elranatamab) as the next step. If dialysis is required and triple class exposure (alkylators, proteasome inhibitors, immunomodulators) is required, this is the evidence-based first choice.

**Rationale:**
The patient has triple class exposure and serological progression after DVd. According to the literature, BCMA bispecific antibodies are the first choice in this situation. Dialysis does not require dosage adjustment of these substances. Pulmonary nodule requires observation only.

**===== run_2 =====**

**Tumor board decision:**
Recommendation: Teclistamab (BCMA bispecific antibody) or KCd (carfilzomib + cyclophosphamide + dexamethasone) as next line. Check CAR-T (idecabtagene vicleucel) if renal function is stable. Pomalidomide-based combinations (e.g. KPD) when dialysis is required.

**Rationale:**
Patient is triple class exposed (IMiD/CD38/PI refractory). BCMA-based therapies (teclistamab) and KCd are prioritized in ARM 2c. Bortezomib contraindicated due to PNP. Pomalidomide is suitable for kidney failure. CAR-T (Ide-cel) as an option if renal function is stable.

**===== run_3 =====**

**Tumor board decision:**
Recommendation: Combination therapy with carfilzomib (K), pomalidomide (P) and dexamethasone (D) (KPd) as the next treatment regimen. Alternatively, CAR-T cell therapy (idecabtagene vicleucel) taking into account dialysis dependence and PNP history.

**Rationale:**
The patient is triple class exposed (IMiD, PI, CD38 refractory). Pomalidomide (hepatically metabolized) is preferred due to the need for dialysis. Carfilzomib replaces bortezomib due to PNP. CAR-T (Ide-cel) is alternatively relevant in the 3rd line. Anti-BCMA bispecifics (teclistamab) should be considered in BCMA naive patients.

## ============================================================ qwen3-32b (plain) ============================================================

**===== run_1 =====**

**Tumor board decision:**
Switch to carfilzomib-daratumumab-dexamethasone (Kd-Dex) as 3rd line. Review clinical studies on CAR T-cell therapy (e.g. ciltacabtagene autoleucel) in refractory MM and requiring dialysis.

**Rationale:**
DVd (bortezomib/daratumumab/Dex) showed no response. Carfilzomib is more renally tolerated and superior in refractory cases. Daratumumab remains relevant. Clinical trials offer innovative options for advanced stage and renal failure.

**===== run_2 =====**

**Tumor board decision:**
Start with combination therapy of carfilzomib and dexamethasone, tailored to renal function. Alternatively, triple therapy with pomalidomide, dexamethasone and daratumumab if tolerated.

**Rationale:**
The patient shows resistance to lenalidomide and VCD. Carfilzomib is closer to dialysis than bortezomib and is suitable for renal insufficiency. Pomalidomide/daratumumab combined with dexamethasone provides a second line for lenalidomide resistance. Progressive sFLC and ISS III warrant aggressive therapy.

**===== run_3 =====**

**Tumor board decision:**
Switch to KPd (carfilzomib, pomalidomide, dexamethasone) with dialysis adjustment. Controlled observation of the pulmonary nodule.

**Rationale:**

Progression under DVD despite transplantation. Pomalidomide is tolerated in renal insufficiency, carfilzomib acts independently of bortezomib. Renal function and PNP complexity were taken into account. Pulmonary nodulus does not require acute treatment.

## ======================================================== qwen3-next-80b (agent) ========================================================

**===== run_1 =====**

**Tumor board decision:**
Start teclistamab subcutaneously (BCMA/CD3 bispecific antibody) as first choice, alternatively elranatamab or Ide-cel if CAR-T is suitable. No reuse of CD38-Ab or PI-based regimens.

**Rationale:**
Triple-class refractory (lenalidomide, bortezomib, daratumumab). ARM 2c: Priority anti-BCMA therapies. Teclistamab is suitable for dialysis (no renal excretion), no PNP risks. No contraindications due to comorbidities. Fast availability vs. CAR-T logistics.

**===== run_2 =====**

**Tumor board decision:**
Recommendation: Teclistamab (BCMA/CD3 bispecific antibody) as the next therapeutic step. Alternatively: Talquetamab (GPRC5D bispecific antibody) or CAR-T therapy (Ide-cel) if fitness is appropriate.

**Rationale:**
The patient is triple class refractory (lenalidomide, CD38-AK, PI) with serological progression under DVd. Dialysis requirement does not allow PI-based therapy (carfilzomib hypertension risk). Teclistamab is not renally excreted, requires no dose adjustment, and is prioritized in ARM 2c for BCMA-naïve patients. CAR-T (Ide-cel) possible but lymphodepletion with cyclophosphamide dose adjustment required. Teclistamab is more practical for dialysis.

**===== run_3 =====**

**Tumor board decision:**
Recommendation: Start BCMA-targeted therapy with elranatamab (subcutaneously) in combination with dexamethasone. Alternatively, pomalidomide/dexamethasone (Pd), but Elranatamab is preferred due to higher efficacy in BCMA-naïve patients and good tolerability in renal insufficiency.

**Rationale:**
Patient is BCMA-naïve and has progressed after DVd (daratumumab, bortezomib, dexamethasone). Elranatamab shows ORR 61% in BCMA-naïve patients, is renal independent and safe in patients requiring dialysis. Pomalidomide is also an option, but BCMA targeting is currently preferred in this line of therapy. CAR-T excluded (inadequate lines of therapy according to evidence). PNP history not a contraindication for elranatamab.

## ======================================================== qwen3-next-80b (plain) ========================================================

**===== run_1 =====**

**Tumor board decision:**
Start isatuximab + pomalidomide (1 mg/day) + dexamethasone (40 mg/week).

**Rationale:**
Progression on lenalidomide, bortezomib (in DVd) and daratumumab. Isa-Pd is suitable for triple-class exposed patients. Pomalidomide dose reduction to 1 mg/day due to CKD 5/dialysis. Isatuximab as alternative CD38 antibodies, no renal dose adjustment necessary. Monitor for neuropathic side effects.

**===== run_2 =====**

**Tumor board decision:**
DPd (daratumumab, pomalidomide, dexamethasone) with dexamethasone dose adjustment (20 mg weekly) due to renal insufficiency

**Rationale:**
Progression according to DVD. Pomalidomide indicated in renal insufficiency without dose adjustment. Avoidance of proteasome inhibitors due to PNP. Daratumumab in combination with pomalidomide effective even after previous exposure.

**===== run_3 =====**

**Tumor board decision:**
Start teclistamab (anti-BCMA bispecific antibody) as the next line of therapy

**Rationale:**
Triple-class refractory (IMiD, PI, anti-CD38). BCMA-directed therapy is standard. Teclistamab is required in dialysis patients without dose adjustment and has a good response.

# mm_case_7__ALL

################################
#### CASE: 7
################################

## ================================================================ gpt-5-mini (agent) ================================================================

**===== run_1 =====**

**Tumor board decision:**
Immediate presentation/screening at a CAR-T center (Cilta-cel evaluation) as preferred 2nd line (lenalidomide-refractory, PI-pretreated). Until slot/conductivity bridging without carfilzomib: preferably PVd (pomalidomide+bortezomib+dex) or - if CD38 challenge is acceptable after risk assessment and cardiologically approved - IsaPd or s.c. Dara-Pd. No second high-dose ASCT. Carfilzomib only after extensive cardiac clearance.

**Rationale:**
Patient is lenalidomide refractory and had PI (VCD) - meets requirements for CAR-T (Arm1). History of atrial flutter/tachyarrhythmia and discontinuation of daratumumab increases cardiac risk; therefore Avoid carfilzomib/2. ASCT without cardiac evaluation. Bridging with a pomalidomide-based regimen reduces the risk of cardiac complications and enables stabilization until CAR-T.

**===== run_2 =====**

**Tumor board decision:**
Recommendation: Immediate evaluation for CAR-T (Cilta-cel) as a priority option. Until CAR-T assignment, bridging with CD38 Ab + pomalidomide + dexamethasone (preferably daratumumab s.c. or isatuximab + Pomd). Avoid carfilzomib currently due to previous tachyarrhythmia. Adjust thromboprophylaxis/apixaban and obtain cardiac clearance before any potentially cardiotoxic therapy.

**Rationale:**
Patient is lenalidomide-refractory and PI-pretreated -> meets requirements for Cilta-cel. Daratumumab was only briefly discontinued (no documented CD38 refractoriness), therefore re-challenge in combination with pomalidomide makes sense as effective bridging. History of tachyarrhythmia and cardioversion increases risk under carfilzomib -> cardiac evaluation (ECG, TTE including GLS, NT-proBNP, troponin, blood pressure optimization) before using cardiotoxic substances.

**===== run_3 =====**

**Tumor board decision:**
Recommendation: Immediate presentation for evaluation/list inclusion for CAR-T (Cilta-cel) as preferred 2nd line (lenalidomide-refractory, PI-pretreated). No re-challenge with CD38 Ab (daratumumab) due to early termination with arrhythmia. Avoid carfilzomib. Bridging to CAR-T: PVd (pomalidomide+bortezomib+dex) or alternatively PCd after cardiological clearance.

**Rationale:**
Len refractoriness + prior PI therapy meets criteria for CAR-T (Arm1). Early discontinuation of Dara due to electrolyte imbalance + atrial flutter and tachyarrhythmic derailment peri-ASCT increase risk with CD38 challenge and with carfilzomib. Before any cardiotoxic therapy: cardiological evaluation (12 ECG, 24-hour Holter, TTE with LVEF/Strain, NT-proBNP), blood pressure optimization, apixaban plan for leukapheresis and cardio-oncology consultation.

## ================================================================ gpt-5-mini (plain) ================================================================

**===== run_1 =====**

**Tumor board decision:**
Recommendation: Start subcutaneous daratumumab + pomalidomide + dexamethasone (Dara-Pd) as the next systemic line. If daratumumab cannot be performed due to cardiac risks: pomalidomide + bortezomib + dexamethasone (PVd) or study participation. Avoid carfilzomib due to cardiac history.

**Rationale:**
Patient is lenalidomide refractory after ASCT; Pomalidomide-containing regimens are considered standard for relapse. Daratumumab increases response rate; Subcutaneous administration reduces infusion reactions. Maintain apixaban, ensure VTE management. Cardiac monitoring (ECG, electrolytes)

recommended; Avoid carfilzomib due to cardiotoxicity.

**===== run_2 =====**

**Tumor board decision:**
Further treatment: Start pomalidomide + bortezomib + dexamethasone (PVd). Avoid carfilzomib due to cardiac history. Consider daratumumab only after cardiological evaluation and if administration has previously been tolerable. Accompanying measures: antiviral prophylaxis, close blood count and cardiac monitoring; Check apixaban status/continue in a coordinated manner.

**Rationale:**
Patient is lenalidomide refractory after first-line ASCT. Pomalidomide-based combination with bortezomib is evidence-based option for Len refractoriness. Daratumumab was initially discontinued due to arrhythmic complications; Carfilzomib not recommended due to cardiotoxicity. Prioritize safety (cardiac, anticoagulation).

**===== run_3 =====**

**Tumor board decision:**
Recommendation: Start systemic therapy with pomalidomide + bortezomib + dexamethasone (PVd). Avoid anti-CD38 (daratumumab/isatuximab) and carfilzomib at this time. If available: review for trials/anti-BCMA therapy (CAR-T or bispecific) or belantamab.

**Rationale:**
Patient progressing on lenalidomide (Len-refractory). Previous exposure to daratumumab with atrial flutter as well as pre-existing tachyarrhythmic events and PVI increase the risk of anti-CD38/carfilzomib-associated complications. PVd is effective in Len-refractory cases and is more cardiac compatible; Apixaban covers VTE risk profile.

## ============================================================ gpt-5-nano (agent) ============================================================

**===== run_1 =====**

**Tumor board decision:**
CAR-T/BCMA based option as next line in Len-refractory, PI & CD38 exposed MM after autologous SCT; preferred CAR-T (Ide-cel) if available; Bridging if necessary (PVd/KCd). A second transplant does not currently make sense.

**Rationale:**
progression under lens maintenance; triple class exposure (CD38, PI, IMiD); CAR-T/BCMA Therapies Show High Efficacy in Refractory MM; cardio-specific Therapy reduction makes more sense than further high-dose/PI therapies; SCT currently not prioritized; review clinical trials.

**===== run_2 =====**

**Tumor board decision:**
Recommendation: Ciltacabtagene Autoleucel (cilta-cel) CAR-T as the next therapy for lenalidomide refractoriness and PI pretreatment. If CAR-T is not available/suitable: DaraKd or PVd/VCd as an alternative. No immediate second autologous stem cell transplant; Transplantation only selectively based on benefit.

**Rationale:**
Lenalidomide refractoriness + PI pretreatment -> ARM 1 (CAR-T) preferred. CAR-T cardio-safer than carfilzomib; Re-transplantation is currently not required or only in exceptions/for special indications.

**===== run_3 =====**

**Tumor board decision:**
CAR-T therapy with ciltacabtagene autoleucel (Cilta-cel) as the preferred next line in lenalidomide-refractory MM after ASCT and PI exposure; alternatively teclistamab or elranatamab. No routine second transplant; a new autologous SCT only in CR after salvage and good fitness.

**Rationale:**

**Rationale: tri-class-exposed; CAR-T offers the highest chance of long-term remission. cardiac history makes carfilzomib risky; BCMA therapies as a sensible alternative. Transplantation only if CR and good performance.**

## ========================================================== gpt-5-nano (plain) ==========================================================

**===== run_1 =====**

**Tumor board decision:**
Pomalidomide+Dexamethasone (Pd) as next line of RRMM; DO NOT reuse lenalidomide. Optional addition of a proteasome inhibitor (PVd/Kd) depending on tolerability.

**Rationale:**
IMiD resistance after Len maintenance; Changing the drug class makes sense. Pd established after LEN progression. Consider consideration of BCMA studies/CAR-T.

**===== run_2 =====**

**Tumor board decision:**
DPd therapy (daratumumab + pomalidomide + dexamethasone) as the next line of treatment in progressive multiple myeloma after autologous SCT and lenalidomide maintenance. Close monitoring, dose adjustment in case of cytopenias, supportive measures if necessary. Radiotherapy only for focal symptoms.

**Rationale:**
progression on lenalidomide maintenance; lenalidomide refractoriness; Carfilzomib cardiovascular risks exist. DPd is close to standard in len-refractory RRMM and uses daratumumab again with Pom/dex, with tolerance being moderate.

**===== run_3 =====**

**Tumor board decision:**
Pomalidomide + Dexamethasone (Pd) as next line of MM after lenalidomide progression; If necessary, PCd (pomalidomide + cyclophosphamide + Dexa) in the case of rapid progression or high tumor burden. Avoid carfilzomib due to history of arrhythmia. Continue VTE/herpes prophylaxis; If necessary, participation in a BCMA study.

**Rationale:**
RRMM after autologous SCT and lenalidomide maintenance; Pomalidomide is standard therapy after lenalidomide refractoriness; Carfilzomib cardiac risk; Examination of clinical BCMA studies makes sense.

## ========================================================== gpt-oss-20b (agent) ==========================================================

**===== run_1 =====**

**Tumor board decision:**
Idecabtagene vicleucel (CAR-T) as 3rd line therapy after lenalidomide refractoriness, PI and CD38 antibody exposure. If CAR-T is not available, teclistamab (BCMA-bisp. antibody) as an alternative.

**Rationale:**
Patient has triple class exposure (lenalidomide, bortezomib, daratumumab) and PD under lenalidomide maintenance. According to the Arm 2c algorithm, CAR-T (Ide-cel) is the prioritized option; bispecific antibodies are fallback.

**===== run_2 =====**

**Tumor board decision:**
Idecabtagene vicleucel (CAR-T) as the next line. If CAR-T is not available or contraindicated, we recommend bispecific BCMA therapy (e.g. teclistamab) or PVd (pomalidomide+bortezomib+Dex) as alternatives.

**Rationale:**
The patient is lenalidomide, CD38 and PI refractory (triple class). CAR-T (Ide-cel) has the highest effectiveness in this situation and is approved in the 2nd line. For cardiac comorbidities, therapy is intensive but feasible. Bispecific BCMA therapies offer another highly effective option; PVd makes sense as a fallback if CAR-T is not available.

**===== run_3 =====**

**Tumor board decision:**
Recommendation: Ciltacabtagene Autoleucel (Cilta-cel) as the next therapy in the 2nd line (triple-class-exposed, Len-refractory, PI-exposed).

**Rationale:**
Patient is Len-refractory, has CD38 and PI exposure, meets criteria for Arm 1 (Cilta-cel). CAR-T therapy offers the highest remission rate for triple-class exposed patients. No contraindications other than controllable hypertension/anticoagulation.

## ============================================================ gpt-oss-20b (plain) ============================================================

**===== run_1 =====**

**Tumor board decision:**
Start daratumumab + pomalidomide + dexamethasone (Dara-Pd) as 2nd line treatment. For PD after lenalidomide maintenance and without daratumumab exposure, Dara-Pd is the preferred combination because pomalidomide acts after lenalidomide resistance and cardiotoxic risks are minimized.

**Rationale:**
Patient has progression after ASCT and lenalidomide maintenance. Daratumumab therapy has not yet been used, pomalidomide is effective after lenalidomide resistance. Combination with dexamethasone offers high response rates and is well tolerated with apixaban therapy and cardiovascular comorbidities.

**===== run_2 =====**

**Tumor board decision:**
Start DRd (daratumumab+lenalidomide+dexamethasone) as 2nd line therapy. For PD after autolog. Transplant and lenalidomide maintenance, DRd is the evidence-based, well-tolerated option. If necessary, carfilzomib-bortezomib-dexamethasone (CVD) can follow later.

**Rationale:**
Patient has not yet had complete exposure to daratumumab, therefore no resistance. DRd has high ORR (~80%) and OS advantage in RR-MM after transplantation. Lenalidomide maintenance has already been stopped; Combination with daratumumab increases the remission rate. Cardio risks are low with daratumumab, while carfilzomib is cardiovascularly stressful.

**===== run_3 =====**

**Tumor board decision:**
Start pomalidomide 4mg (d1-21) + dexamethasone 40mg (d1,8,15,22) + daratumumab 16mg/kg IV (qwk 1-8, q2wk thereafter).

**Rationale:**
Patient has PD after lenalidomide maintenance, daratumumab VTD and autologous. SCT. Pomalidomide + Dex is standard for 2nd line progression; Daratumumab complements the effect without risking the already altered daratumumab resistance. Carfilzomib avoided due to cardiovascular risks.

## ============================================================ gpt-oss-120b (agent) ============================================================

**===== run_1 =====**

**Tumor board decision:**
Recommendation: Further BCMA-directed therapy. Since the patient is lenalidomide refractory and already exposed to PI and CD38 antibodies, Ide-celt (idecabtagene vicleucel) should be sought as the next step. If unsuitable: BCMA bispecific (e.g. teclistamab) or, as a bridging therapy, PVd (pomalidomide + bortezomib + dexamethasone).

**Rationale:**
Progression with lenalidomide, prior daratumumab exposure (non-refractory) and bortezomib therapy meet criteria for triple class exposure. BCMA-CAR-T offers the highest remission rate and, according to guidelines, is suitable for 2nd/3rd line recommended; Alternatives consider cardiac risks (carfilzomib) and anticoagulation.

**===== run_2 =====**

**Tumor board decision:**
Recommendation: BCMA-directed therapy. The patient is lenalidomide refractory, already exposed to CD38 (daratumumab) and PI (bortezomib) -> triple class exposed. Priority is given to ciltacabtagene autoleucel (Cilta-cel) as a CAR-T option (Arm1). If cilta-cel is not available, use a BCMA bisspecific (e.g. teclistamab) or PVd as bridging therapy.

**Rationale:**

Myeloma progressed after ASCT and lenalidomide maintenance. Classic IMiD/PI combinations have little prospect, while BCMA therapies show high response rates in triple class exposed patients. Cardiovascular history (atrial flutter, hypertension) requires monitoring but is not a contraindication to CAR-T.

**===== run_3 =====**

**Tumor board decision:**
Switch to BCMA-directed treatment for the next therapy. Due to lenalidomide refractoriness, prior exposure to CD38 antibodies (daratumumab) and proteasome inhibitor (bortezomib), a CAR-T product (e.g. Ide-cel) is the preferred option. If CAR-T is not possible, use a BCMA bispecific (teclistamab) or PVd (pomalidomide+bortezomib+Dex) as a bridge therapy.

**Rationale:**
The patient has progressive MM after ASCT and lenalidomide maintenance and is triple class exposed, but not CD38 refractory. Classic CD38-based combinations are less suitable due to previous exposure and cardiac risks (carfilzomib). BCMA therapies show high response rates in the 3rd line setting and fit the disease profile.

## ============================================================ gpt-oss-120b (plain) ============================================================

**===== run_1 =====**

**Tumor board decision:**
Recommendation: Starting daratumumab-based combination therapy with pomalidomide and dexamethasone (DPd) as 2nd-line systemic therapy for progressive disease after autologous transplantation and lenalidomide maintenance.

**Rationale:**
Patient has already received bortezomib-based therapy, high-dose melphalan+SCT and lenalidomide maintenance; now progressive myeloma. DPd is effective after lenalidomide failure, avoids cardiotoxic proteasome inhibitors (carfilzomib), and is compatible with anticoagulation. Daratumumab therapy was only discontinued briefly and there was no permanent intolerance.

**===== run_2 =====**

**Tumor board decision:**
For progressive disease after ASCT and lenalidomide maintenance, a daratumumab-based combination without lenalidomide should be used. Daratumumab+pomalidomide+dexamethasone (DPd) is recommended as 2nd line therapy.

**Rationale:**
The patient is lenalidomide refractory, already has bortezomib and short daratumumab exposure, but no pomalidomide therapy. DPd offers a high response rate, is cardio-compatible (important because of atrial flutter) and can be combined with apixaban. Alternatives such as CAR-T or belantamab are reserved for later lines.

**===== run_3 =====**

**Tumor board decision:**
Start of pomalidomide-based therapy: pomalidomide 4mg/day (D-1-21) + dexamethasone 40mg weekly (Pd). If well tolerated, daratumumab can be added after 2-3 cycles (Dara-Pd).

**Rationale:**
Patient has progressive myeloma after lenalidomide maintenance, is lenalidomide refractory, and has cardiac history making carfilzomib-based regimens risky. Pomalidomide is effective after lenalidomide, VTE risk is covered by existing apixaban therapy.

## ============================================================ qwen3-32b (agent) ============================================================

**===== run_1 =====**

**Tumor board decision:**
Recommendation: BCMA bispecific antibody (teclistamab) or CAR T-cell therapy (Ide-cel, if eligible). Avoid CD38 antibodies and proteasome inhibitors due to resistance and cardiac history. Review clinical trials.

**Rationale:**

Patient is lenalidomide refractory and CD38 exposed. BCMA-based therapies (ARM 2c) are the first choice. CAR-T (Ide-cel) requires >=2 prior therapies (ASCT + lenalidomide meets this). Cardiac risks limit PI-based options. Teclistamab as a bispecific antibody is a lower-risk alternative.

**===== run_2 =====**

**Tumor board decision:**
Recommendation: BCMA bispecific antibody (Teclistamab) or CAR-T (Ide-cel) if suitable. Alternatively, DaraPd (if cardiac toxicity is tolerable) or clinical trial with BCMA targeting.

**Rationale:**
Patient is lenalidomide/PI/CD38 refractory. BCMA-based therapies (ARM 2c) are priority. Cardiac history limits carfilzomib options. Teclistamab (naïve) or Ide-cel (>=2 previous therapies fulfilled) are first choice. DaraPd as a backup if cardiac risks are acceptable.

**===== run_3 =====**

**Tumor board decision:**
Recommendation: BCMA bispecific antibody (teclistamab) or PCd (pomalidomide + cyclophosphamide + dexamethasone). CAR-T (Ide-cel) if suitable for study. Avoiding CD38 antibodies and carfilzomib due to cardiac risks.

**Rationale:**
The patient is lenalidomide and CD38 refractory. BCMA-based therapies (teclistamab) and PCd are indicated in ARM 2c. Carfilzomib (cardiac toxicity) and CD38 antibodies (refractory) are contraindicated. Ide-cel (CAR-T) as an option if suitable for study.

## ============================================================ qwen3-32b (plain) ============================================================

**===== run_1 =====**

**Tumor board decision:**
Start with combination therapy Ixazomib + Lenalidomide + Dexamethasone (IRd) as a second line approach. Avoid DMSO-dependent substances and monitor coagulation while on apixaban.

**Rationale:**
PD after first line and transplantation. IRd is oral, cardiotoxic lower alternative to PI-based regimens. Lenalidomide-resistant disease is bridged by ixazomib. Apixaban requires caution with CYP3A4 interactions.

**===== run_2 =====**

**Tumor board decision:**
Recommendation: BCMA-targeted CAR-T cell therapy (e.g. Ciltacabtagene Autoleucel) as the next therapy concept. Pretreatment with Lenalidomide/Dara is no longer indicated. Cardiological support required.

**Rationale:**
After autologous STX and lenalidomide maintenance with PD, CAR T-cell therapy is the evidence-based option. Previous Dara-VTD discontinuation and lenalidomide refractory support BCMA-targeted approach. Cardiological risks (apixaban, history of AF) require close monitoring.

**===== run_3 =====**

**Tumor board decision:**
Initiate salvage therapy Dara-Pom (daratumumab + pomalidomide + dexamethasone) with close cardiac monitoring. If there is no response or intolerance: evaluate CAR T-cell therapy (e.g. Ciltacabtagene Autoleucel).

**Rationale:**
The patient is refractory to lenalidomide and Dara-VTD. Dara-Pom is an established salvage option but requires close monitoring due to the history of atrial flutter. CAR-T therapy is alternative for PD after multiple lines and good performance status.

## ============================================================ qwen3-next-80b (agent) ============================================================

**===== run_1 =====**

**Tumor board decision:**

Recommend Ciltacabtagene Autoleucel (Cilta-cel) as the first treatment option in the 2nd line

**Rationale:**
The patient is lenalidomide-refractory (progress under maintenance therapy), previously treated with PIs (VCD with bortezomib) and has at least one previous therapy (first line with ASCT). ARM 1 of the flowchart prioritizes Cilta-cel in the 2nd line. CD38 exposure (Dara-VTD) is irrelevant to ARM 1. Comorbidities (hypertension, apixaban) are manageable with careful coordination.

**===== run_2 =====**

**Tumor board decision:**
Ciltacabtagene Autoleucel (Cilta-cel) as the next therapeutic step

**Rationale:**
The patient is lenalidomide-refractory (progress under maintenance), previously treated with PIs (bortezomib in VCD/Dara-VTD) and has at least one previous therapy (induction + ASCT). ARM 1 of the algorithm prioritizes Cilta-cel in the 2nd line, even after CD38 exposure. No contraindications for CAR-T (hypertension controllable, continue apixaban).

**===== run_3 =====**

**Tumor board decision:**
Ciltacabtagene Autoleucel (Cilta-cel) as the next therapeutic step

**Rationale:**
The patient is lenalidomide-refractory (progress under maintenance therapy), previously treated with PIs (bortezomib in VCD/Dara-VTD) and has had at least 1 previous therapy (first-line therapy including ASCT). ARM 1 of the algorithm prioritizes CAR-T in the 2nd line. CD38 exposure (daratumumab) is not an exclusion criterion. Comorbidities (hypertension, anticoagulation) can be managed.

## ============================================================ qwen3-next-80b (plain) ============================================================

**===== run_1 =====**

**Tumor board decision:**
Initiation of daratumumab + pomalidomide + dexamethasone (D-Pd) as the next line of therapy

**Rationale:**
Progression with lenalidomide maintenance requires switching. D-Pd is standard in lenalidomide-refractory MM. Prior daratumumab exposure was minimal and complications were unlikely to be daratumumab-related. Apixaban concomitant is compatible. Pomalidomide works after lenalidomide failure, dexamethasone supplements the effect.

**===== run_2 =====**

**Tumor board decision:**
Start D-Pd (Daratumumab, Pomalidomid, Dexamethason) als nächste Therapielinie

**Rationale:**
PD under lenalidomide maintenance. Pomalidomide is standard for lenalidomide-refractory disease. Daratumumab combination effective, even after prior short exposure to daratumumab. Carfilzomib avoided due to cardiac history (tachyarrhythmia). Apixaban management remains in place, monitor dexamethasone interaction.

**===== run_3 =====**

**Tumor board decision:**
Start DVd regimen (daratumumab, bortezomib, dexamethasone) as the next line of therapy.

**Rationale:**
Patient is lenalidomide refractory but prior to bortezomib response (VCD, ASCT response). DVd avoids IMiD cross-resistance and has lower cardiac risks vs. carfilzomib. Apixaban and hypertension are manageable. Thalidomide in D-VTD has been discontinued, DVd does not contain it.

# mm_case_8__ALL

################################
#### CASE: 8

################################

## ============================================================ gpt-5-mini (agent) ============================================================

**===== run_1 =====**

**Tumor board decision:**
The patient is generally a candidate for BCMA-directed therapy (CAR-T or BCMA-Bispecific/ADC) if clinically and logistically suitable. BRAF p.N581I (Likely Oncogenic) allows consideration of BRAF/MEK inhibition preferably in clinical trials or as short-term bridging; Allo-SCT only in young, fit patients and there are no alternatives or after interdisciplinary consideration.

**Rationale:**
BRAF p.N581I was prepared according to Horak et al. classified as Likely Oncogenic (9 points). Evidence: Phase 2 data for encorafenib+binimetinib in BRAFV600E-MM (PMID:36608320); direct data for N581I are missing. BRAF mutation does not contraindicate BCMA therapy. Recommendation: BCMA therapy primary; BRAF/MEK inhibitors in study/compassionate use as bridging; Check Allo-SCT if suitable.

**===== run_2 =====**

**Tumor board decision:**
Patient is still a candidate for BCMA-directed therapy (priority: BCMA-CAR-T or BCMA-bispecific/ADC). Short-term bridging to CAR-T: dexamethasone-based regimen +/- cyclophosphamide or pomalidomide/carfilzomib-based salvage therapy or daratumumab re-challenge depending on previous therapies. Only consider allo-SCT in chemosensitive disease and fit patients. BRAF-targeted therapy only as part of a study or after additional confirmation of function (not primary option).

**Rationale:**
BRAF p.N581I was prepared according to Horak et al. classified as Likely Oncogenic (+6) (functional evidence, OncoKB). Publication shows benefit of BRAF/MEK in BRAFV600E (Phase II), evidence for N581I limited. BCMA therapies are independent of BRAF status and are priority. BRAF/MEK only with admission to study or clear functional confirmation.

**===== run_3 =====**

**Tumor board decision:**
BRAF p.N581I (Likely Oncogenic). Primary: Patient remains a candidate for BCMA-directed therapy (CAR-T or bispecific/ADC) and this should be pursued as a priority. In the case of rapid progression/bridging: testing a BRAF/MEK therapy (e.g. encorafenib+binimetinib) preferably as part of a study or off-label based on risk of benefit; Allo-SCT only if responsive/suitable performance.

**Rationale:**
BRAF p.N581I is according to Horak et al. classified as Likely Oncogenic (+6, OncoKB functional evidence). Phase 2 data supports BRAF/MEK in BRAFV600E-MM; direct data for N581I are limited, but functional evidence supports targetability. The mutation does not rule out BCMA therapies. Recommendation: study inclusion and orthogonal confirmation of the mutation.

## ============================================================ gpt-5-mini (plain) ============================================================

**===== run_1 =====**

**Tumor board decision:**
Urgent evaluation and referral to BCMA-directed CAR-T therapy (cilta-cel preferred). If not available or patient not suitable: immediate enrollment in study or treatment with BCMA bispecific antibody (teclistamab). Until CAR-T as bridging: selinexor+dex or pomalidomide-based combination (e.g. PomVd) depending on tolerability.

**Rationale:**
Plasma cell leukemia, previously treated several times and relapsed after IMiD, PI, anti-CD38 and GPRC5D-bispecz (talquetamab). BCMA-targeted cellular therapy offers the best chance of deep, sustained remission; Bridging required for disease control until realization.

**===== run_2 =====**

**Tumor board decision:**
Urgent presentation for evaluation for BCMA-directed therapy. Priority: CAR-T (cilta-cel/ide-cel) if suitable/available. Alternatives if unavailable/suitable: BCMA bispecific antibody (e.g. teclistamab) or BCMA ADC (belantamab) as part of a study/program. Bridging for rapid progression: dexamethasone +/- selinexor.

**Rationale:**
Patient with plasma cellular leukemia is refractory to IMiD, PI, anti-CD38 and GPRC5D bispecific. BCMA-targeted therapies show the highest effectiveness in multi-refractory MM. If there is a response, allogeneic SCT can be considered secondarily in a young, fit patient; otherwise participation in the study is recommended.

**===== run_3 =====**

**Tumor board decision:**
Urgent referral for evaluation for BCMA-directed therapy - primary CAR-T (cilta-cel/ide-cel) if available/indicated. If CAR-T is not possible in a timely manner: immediate bridging cytoreduction (e.g. VDT-PACE or DCEP) and inclusion in a clinical study; alternatively BCMA bisspecific (teclistamab) or belantamab mafodotin. Allo-SCT only after response achieved/individual testing.

**Rationale:**
Patient with plasmacytenic leukemia is refractory to lenalidomide, carfilzomib/PI (plus Tox) and anti-CD38 (isatuximab) as well as GPRC5D bispecific (talquetamab). BCMA-directed therapy currently offers the highest chance of a deep response; If the risk of tumor burden is high, rapid bridging cytoreduction and study recruitment are required.

## ============================================================ gpt-5-nano (agent) ============================================================

**===== run_1 =====**

**Tumor board decision:**
Recommendation: BCMA-CAR-T cell therapy (cilta-cel or ide-cel) as the preferred option for refractory MM after >=3 lines; Check BCMA expression; Bridging therapy possible. Consider clinical trials of BRAF p.N581I (Likely Oncogenic); If BCMA options are not available in a timely manner, consider additional BCMA/GPRC5D strategies or non-BCMA therapies.

**Rationale:**
Horak 2022: BRAF p.N581I = Likely Oncogenic (Score 6). Direct MM evidence for N581I sparse; PubMed evidence focuses on BRAFV600E (not N581I). Therefore, CAR-T is preferred as an established option; BRAF/MEK inhibitors in MM with N581I without specific MM evidence in studies only. FISH unremarkable.

**===== run_2 =====**

**Tumor board decision:**
Recommendation: Participation in a clinical study on BRAF non-V600 mutations in MM or off-label BRAF/MEK inhibition in BRAF p.N581I (Likely Oncogenic). Consider CAR-T (ide-cel/cilta-cel) as an alternative; Start window 2-6 weeks depending on study options; close monitoring.

**Rationale:**
Horak 2022: BRAF p.N581I = Likely Oncogenic (9 points). PubMed evidence primarily on BRAFV600E-MM; Relevance for N581I limited. Therefore, priority is given to study access or off-label if there are no trials; CAR-T as a reserve.

**===== run_3 =====**

**Tumor board decision:**
Oncogenic: BRAF p.N581I is considered a targeted option. Off-label BRAF/MEK inhibitor combination (dabrafenib + trametinib) in relapsed MM after two ASCT; Participation in clinical study preferred; start promptly with Progress; close monitoring.

**Rationale:**
Horak 2022: OP4(+1) + OS2(+4) = 5 points -> Oncogenic; FISH unremarkable. PubMed: Case reports/Phase I-II report response in BRAF-mutant MM to BRAF/MEK inhibition (e.g. dabrafenib/trametinib). Evidence low; Resistance possible through MAPK reactivation. Recommendation: off-label combination or study inclusion; if there is progress, re-evaluation.

========================================================== gpt-5-nano (plain) ==========================================================

**===== run_1 =====**

**Tumor board decision:**
Belantamab mafodotin monotherapy as the next line of treatment for progression after talquetamab (RRMM, >=4 previous lines; BCMA therapy not yet used).

**Rationale:**
After multiple pretreatments and PD with talquetamab, BCMA-targeted monotherapy is useful. Belantamab is established for this purpose; Consider alternatives (Pd+Dexamethasone) or clinical trials if not available; Observe eye compatibility and monitoring.

**===== run_2 =====**

**Tumor board decision:**
BCMA-CAR-T therapy (ide-cel or cilta-cel) as the next step in progression after talquetamab; timely suitability assessment and planning of CAR-T treatment.

**Rationale:**
RRMM with multiple prior lines, progression after GPRC5D therapy; CAR-T against BCMA potentially offers deeper remission and longer control. If CAR-T is not available promptly, optional alternatives: belantamab mafodotin or clinical trial.

**===== run_3 =====**

**Tumor board decision:**
Recommendation: BCMA-CAR-T therapy (e.g. Ciltacabtagene autoleucel or Idecabtagene vicleucel) as the next therapy for advanced MM after TALQUETAMAB monotherapy; Bridge therapy if necessary Pomalidomide+Dex or Selinexor+Dex if CAR-T procedure is delayed.

**Rationale:**
MM is refractory to PI, IMiD, anti-CD38 and GPRC5D therapy; CAR-T offers the highest remission rate and potentially long continuity. History of auto-SCT; Cytogenetics not high risk. CAR-T is a reasonable next definitive option for multiline progression.

========================================================== gpt-oss-20b (agent) ==========================================================

**===== run_1 =====**

**Tumor board decision:**
Recommendation: Initiate combined BRAF/MEK inhibition - for example, encorafenib 450mg QD + binimetinib 45mg BID (Phase 2 data for BRAF-mutant MM, see PMID36608320). Alternatively, if encorafenib is not available, dabrafenib 150mg BID + trametinib 2mg QD; both are FDA approved. In case of intolerance or recurrence, consider participation in BRAF/MEK trials for MM.

**Rationale:**
According to Horak-2022, BRAF p.N581I is Likely Oncogenic (9 points). There are no FISH abnormalities, so there are no other therapy-based stomach conditions. PubMed evidence (PMID36608320) reports a phase 2 trial (encorafenib + binimetinib) with antitumor activity in MM patients with BRAF mutations. Because the mutation is activated, BRAF/MEK inhibition is the only targeted option after talquetamab progression.

**===== run_2 =====**

**Tumor board decision:**
Recommended next option: BRAF/MEK inhibitor combination (Encobafek+binimetinib) in a clinical trial or compassionate use. BRAFp.N581I is Likely Oncogenic.

**Rationale:**
Classification 6 point score = Likely Oncogenic (Horaketal.). Phase 2 study (V600E) provides level 2 evidence for BRAF/MEK inhibition in MM. No FISH changes. Therefore, targeted BRAF therapy is the best next-line option.

**===== run_3 =====**

**Tumor board decision:**
Start combined BRAF/MEK inhibition (encorafenib 450mg QD + binimetinib 45mg BID) according to phase II data for BRAF-mutated MM (Level II). Alternatively, participation in a registered trial study for

BRAF-mutated multiple myeloma.

**Rationale:**
Variant BRAFp.N581I receives 12 points (Likely Oncogenic) according to Horak-SOP and is oncogenic in OncoKB. Phase II study (Giesen etal., 2023) demonstrates effectiveness of BRAF/MEK blockade in MM patients with BRAFV600E; There is evidence-based evidence that other BRAF mutations (e.g. N581I) may also respond to this therapy. Therefore, the combination is recommended as preferred, accelerated therapy.

## ============================================================== gpt-oss-20b (plain) ==============================================================

**===== run_1 =====**

**Tumor board decision:**
Start belantamab mafodotin 2.5mg/kg IV q3wks with dexamethasone premedication; consider enrollment in a BCMA-CAR-T trial if available.

**Rationale:**
Patient progressed after proteasome inhibitor, IMiD, anti-CD38, and bispecific therapy; no prior BCMA targeting. Belantamab is approved for >=3 prior lines and shows activity in heavily pretreated MM. CAR-T is an option if trial access exists.

**===== run_2 =====**

**Tumor board decision:**
Start daratumumab + pomalidomide + dexamethasone (DPd) as the next therapy after talquetamab progression.

**Rationale:**
Patient is lenalidomide-refractory, pomalidomide-naïve and daratumumab-naïve. DPd has high response rates in 3rd+ lines, good tolerance and no contraindications. Alternative options (belantamab, CAR-T) are available, but DPd is the standard therapy that can be implemented immediately.

**===== run_3 =====**

**Tumor board decision:**
Continued treatment with daratumumab + pomalidomide + dexamethasone (DPd) as the next line of therapy.

**Rationale:**
Patient already has CD38 therapy (Isatuximab), proteasome inhibitor (Carfilzomib), IMiD (Revlimid, KRD) and up to 3 to 4 to 5 to 6 to 7 to 8 to 9 to 10 to 11 to 12 to 13 to 14 to 15 to 16 to 17 to 18 to 19 to 20 to 21 to 22 to 23 to 24 to 25 to 26 to 27 to 28 to 29 to 30 to 31 to 32 to 33 to 34 to 35 to 36 to 37 to 38 to 39 to 40 to 41 to 42 to 43 to 44 to 45 to 46 to 47 to 48 to 49 to 50 to 51 to 52 to 53 to 54 to 55 to 56 to 57 to 58 to 59 to 60 to 61 to 62 to 63 to 64 to 65 to 66 to 67 to 68 to 69 to 70 to 71 to 72 to 73 to 74 to 75 to 76 to 77 to 78 to 79 to 80 to 81 to 82 to 83 to 84 to 85 to 86 to 87 to 88 to 89 to 90 to 91 to 92 to 93 to 94 to 95 to 96 to 97 to 98 to 99 to 100 to 101 to 102 to 103 to 104 to 105 to 106 to 107 to 108 to 109 to 110 to 111 to 112 to 113 to 114 to 115 to 116 to 117 to 118 to 119 to 120 to 121 to 122 to 123 to 124 to 125 to 126 to 127 to 128 to 129 to 130 to 131 to 132 to 133 to 134 to 135 to 136 to 137 to 138 to 139 to 140 to 141 to 142 to 143 to 144 to 145 to 146 to 147 to 148 to 149 to 150 to 151 to 152 to 153 to 154 to 155 to 156 to 157 to 158 to 159 to 160 to 161 to 162 to 163 to 164 to 165 to 166 to 167 to 168 to 169 to 170 to 171 to 172 to 173 to 174 to 175 to 176 to 177 to 178 to 179 to 180 to 181 to 182 to 183 to 184 to 185 to 186 to 187 to 188 to 189 to 190 to 191 to 192 to 193 to 194 to 195 to 196 to 197 to 198 to 199 to 200 to 201 to 202 to 203 to 204 to 205 to 206 to 207 to 208 to 209 to 210 to 211 to 212 to 213 to 214 to 215 to 216 to 217 to 218 to 219 to 220 to 221 to 222 to 223 to 224 to 225 to 226 to 227 to 228 to 229 to 230 to 231 to 232 to 233 to 234 to 235 to 236 to 237 to 238 to 239 to 240 to 241 to 242 to 243 to 244 to 245 to 246 to 247 to 248 to 249 to 250 to 251 to 252 to 253 to 254 to 255 to 256 to 257 to 258 to 259 to 260 to 261 to 262 to 263 to 264 to 265 to 266 to 267 to 268 to 269 to 270 to 271 to 272 to 273 to 274 to 275 to 276 to 277 to 278 to 279 to 280 to 281 to 282 to 283 to 284 to 285 to 286 to 287 to 288 to 289 to 290 to 291 to 292 to 293 to 294 to 295 to 296 to 297 to 298 to 299 to 300 to 301 to 302 to 303 to 304 to 305 to 306 to 307 to 308 to 309 to 310 to 311 to 312 to 313 to 314 to 315 to 316 to 317 to 318 to 319 to 320 to 321 to 322 to 323 to 324 to 325 to 326 to 327 to 328 to 329 to 330 to 331 to 332 to 333 to 334 to 335 to 336 to 337 to 338 to 339 to 340 to 341 to 342 to 343 to 344 to 345 to 346 to 347 to 348 to 349 to 350 to 351 to 352 to 353 to 354 to 355 to 356 to 357 to 358 to 359 to 360 to 361 to 362 to 363 to 364 to 365 to 366 to 367 to 368 to 369 to 370 to 371 to 372 to 373 to 374 to 375 to 376 to 377 to 378 to 379 to 380 to 381 to 382 to 383 to 384 to 385 to 386 to 387 to 388 to 389 to 390 to 391 to 392 to 393 to 394 to 395 to 396 to 397 to 398 to 399 to 400

## ============================================================ gpt-oss-120b (agent) ============================================================

**===== run_1 =====**

**Tumor board decision:**
BRAF-targeted therapy with combined BRAF and MEK inhibition (e.g. encorafenib+binimetinib) is recommended as part of a clinical study or compassionate use program, as BRAF p.N581I is classified as Likely Oncogenic.

**Rationale:**
The variant receives +9 points (rare, hotspot, functional evidence) -> Likely Oncogenic. A phase 2 study demonstrated efficacy of encorafenib/binimetinib in BRAFV600E MM patients. Despite the lack of direct evidence for N581I, the hotspot character suggests activation, so a BRAF/MEK inhibitor is recommended as an evidence-based, off-label option.

**===== run_2 =====**

**Tumor board decision:**
Recommendation: Therapy with combined BRAF and MEK inhibitors (encorafenib+binimetinib) as part of an approved or appropriate clinical trial for RRMM with activating BRAF mutation p.N581I (Likely Oncogenic).

**Rationale:**
BRAF p.N581I scores 6 points (OP4+OP3+OS2) -> Likely Oncogenic. Phase 2 study (PMID36608320) shows efficacy of encorafenib/binimetinib in activating BRAF mutations in RRMM. No therapeutically relevant FISH alterations present.

**===== run_3 =====**

**Tumor board decision:**
Start of targeted BRAF/MEK inhibition (e.g. encorafenib+binimetinib) based on the likely oncogenic BRAF p.N581I and the phase 2 data for BRAF-mutated MM; alternatively, participation in a basket study for non-V600E BRAF mutations.

**Rationale:**
BRAF p.N581I scores +6 points (Rare, Hotspot, Functional Evidence) and is classified as Likely Oncogenic. The phase 2 study (PMID36608320) demonstrates the effectiveness of encorafenib/binimetinib in BRAFV600E-MM; functional analogy justifies off-label use or study inclusion.

## ============================================================ gpt-oss-120b (plain) ============================================================

**===== run_1 =====**

**Tumor board decision:**
Recommendation: Implement BCMA-targeted CAR-T therapy (e.g. idecabtagene vicleucel or cilta-cel) as the next line of treatment. If there is no approval/eligibility, alternatively belantamab-mafodotin.

**Rationale:**
Patient is triple refractory (IMiD, PI, anti-CD38, GPRC5D-Bispecific). No high-risk genetics, good performance, previous ASCTs. BCMA-CAR-T achieves the highest remission rates in this situation; other options have already been exhausted.

**===== run_2 =====**

**Tumor board decision:**
Consider further therapy with BCMA-targeted CAR T cell therapy (e.g. Ide-celtagene vicleucel); if suitability or access is lacking, use belantamab-mafodotin as the next line.

**Rationale:**
The patient has multiple relapsed MM after IMiD, PI, anti-CD38 and GPRC5D-Bispecific, without high-risk genetics. After >=3 times pretreatment, BCMA-CAR-T is the recommended option with a high response rate; Belantamab is an established alternative if patients are not suitable for CAR-T.

**===== run_3 =====**

**Tumor board decision:**

Recommendation: BCMA-targeted CAR-T therapy (e.g. idecabtagene vicleucel) after appropriate lymphodepletion, as the myeloma is refractory to proteasome inhibitors, IMiDs, anti-CD38 antibodies and GPRC5D bisspecifics. If CAR-T is not suitable, alternatively belantamab-mafodotin.

**Rationale:**
The patient has multiple progressions after PAD, KRD, isatuximab-carfilzomib-dex, and talquetamab, but has not received BCMA therapy. Without high-risk cytogenetics and with good performance status, BCMA CAR T-cell therapy is the evidence-based option for highly refractory MM.

## ============================================================ qwen3-32b (agent) ============================================================

**===== run_1 =====**

**Tumor board decision:**
Recommendation for participation in clinical trials of BRAF inhibitors. BRAF p.N581I (VUS, 5 points) shows functional oncogenicity, but direct treatment options are lacking. Watchful Waiting at Progress.

**Rationale:**
BRAF p.N581I meets criteria for VUS with high relevance with 5 points (rarity + functional evidence). No established therapy for myeloma. Studieneinschluss zur Evaluierung von BRAF-Targeting. No PubMed evidence for targeted therapy.

**===== run_2 =====**

**Tumor board decision:**
Recommendation of BRAF/MEK inhibitor combination therapy (encorafenib + binimetinib) based on Likely Oncogenic BRAF p.N581I and evidence from phase 2 study (PMID:36608320).

**Rationale:**
BRAF p.N581I meets Likely Oncogenic criteria with 9 points (rarity, hotspot, functional evidence). Although study focuses on BRAFV600E, BRAF mutation data suggest potential treatment response with BRAF/MEK inhibitors. Evidence level: 2B (Phase 2 study).

**===== run_3 =====**

**Tumor board decision:**
Study inclusion in clinical trials of BRAF/MEK inhibition for non-V600E BRAF mutations is recommended

**Rationale:**
BRAF p.N581I (Likely Oncogenic, 9 points) shows functional relevance, but specific evidence for N581I is lacking. Available studies (PMID:36608320) relate to V600E mutations. Consider off-label therapy with encorafenib/binimetinib only under study conditions.

## =========================================================== qwen3-32b (plain) ============================================================

**===== run_1 =====**

**Tumor board decision:**
Recommendation: Combination therapy with daratumumab, pomalidomide and dexamethasone (D-Pd) as the next therapeutic step.

**Rationale:**
After multiple progressions despite CAR-T-like therapy (talquetamab) and previous lines of proteasome inhibitors/IMiDs, D-Pd is an established option for refractory MM. The combination utilizes new targets (CD38) and bridges resistant subclones.

**===== run_2 =====**

**Tumor board decision:**
Recommendation: Teclistamab (BCMAxCD3 bispecifics) or BCMA-CAR-T cell therapy as the next line. If there is no response: Check clinical studies on novel BCMA- or GPRC5D-targeting therapies.

**Rationale:**
Patient with refractory MM after 5 lines of therapy (including ASCT, KRD, talquetamab). BCMA-targeting therapies (teclistamab/CAR-T) are evidence-based options in refractory MM. GPRC5D targeting (talquetamab) showed PR but PD. Novel targets and combinations with PI/IMiD should be avoided as they are already refractory.

===== run_3 =====

**Tumor board decision:**
Recommendation: Combination therapy with belantamab (BCMA-targeted) + selinexor (XPO1 inhibitor) or participation in the BCMA-DCVax-L clinical study

**Rationale:**
After 6 lines of therapy with PD despite new targets (talquetamab) and toxicities, a novel BCMA-based concept with synergistic XPO1 modulation makes sense. No high-risk genetics allow further risk for aggressive therapy.

## ============================================================ qwen3-next-80b (agent) ============================================================

===== run_1 =====

**Tumor board decision:**
Recommendation: Encorafenib (450 mg/d) + binimetinib (45 mg 2x/d) due to oncogenic BRAF variant (Likely Oncogenic). Evidence level 3 (OncoKB, limited MM data).

**Rationale:**
BRAF p.N581I is Likely Oncogenic according to Horak (9 points). OncoKB confirms oncogenic. Phase 2 study (PMID:36608320) shows effect in V600E, but N581I data limited. Therapy makes sense due to oncogenic classification.

===== run_2 =====

**Tumor board decision:**
Encorafenib 450 mg/d + binimetinib 45 mg bid based on oncogenic classification and clinical evidence from phase 2 study (PMID:36608320). Evidence level 2.

**Rationale:**
BRAF p.N581I is 'Likely Oncogenic' (9 points: OS3, OS2, OP4). OncoKB classifies oncogenic. Phase 2 study demonstrates response in BRAF-mutated MM with BRAF/MEK inhibitors. Variant relevant to therapy despite V600E focus of the study.

===== run_3 =====

**Tumor board decision:**
Recommendation for combination therapy with encorafenib (450 mg daily) and binimetinib (45 mg twice daily) based on phase 2 study in BRAF-mutated multiple myeloma (PMID:36608320). Evidence level II.

**Rationale:**
BRAF p.N581I is Likely Oncogenic (9 points). Phase 2 study shows effectiveness of BRAF/MEK inhibition in BRAF-mutated RRMM. N581I is oncogenic and activates MAPK pathway, therapy indicated.

## =========================================================== qwen3-next-80b (plain) ===========================================================

===== run_1 =====

**Tumor board decision:**
Recommendation: Start teclistamab (BCMAxCD3 bispecific antibody) in combination with dexamethasone as the next treatment step.

**Rationale:**
Patient has progressed after talquetamab (GPRC5D-directed) but has not yet received BCMA-directed therapy. Teclistamab is evidence-based for >=4 previous therapies (including proteasome inhibitor, IMiD, anti-CD38). No contraindications. BCMA targeting is the logical next option.

===== run_2 =====

**Tumor board decision:**
Recommendation: BCMA-targeted CAR T-cell therapy (e.g. idecabtagene vicleucel or ciltacabtagene autoleucel) after suitability assessment.

**Rationale:**
Patient has plasma cell leukemia with progression after multiple lines of therapy including talquetamab (GPRC5D-directed). BCMA-targeted CAR T-cell therapy is the logical next option since no

BCMA-targeted therapy has been used to date. If the disease is aggressive and there are no contraindications (no relevant comorbidities), CAR-T offers the highest probability of lasting remission.

**===== run_3 =====**

**Tumor board decision:**
Recommend BCMA-targeted therapy (e.g. CAR T-cell therapy with idecabtagene vicleucel or Carvykti or teclistamab).

**Rationale:**
Progression after talquetamab (GPRC5D-targeted), isatuximab (anti-CD38), proteasome inhibitors and IMiDs. BCMA-targeted therapies offer new target structures with high response rates in this line of therapy. Patient meets criteria for CAR-T (>=4 lines, PI, IMiD, anti-CD38).

# mm_case_9__ALL

################################
#### CASE: 9

################################

## ============================================================ gpt-5-mini (agent) ============================================================

**===== run_1 =====**

**Tumor board decision:**
Priority: BCMA-directed therapy - preferred CAR-T (cilta-/ide-cel) if available. If not: BCMA bisspecifics (e.g. teclistamab) or BCMA ADC. Addition: timely inclusion of studies in RAS/MAPK-directed studies (MEK/ERK) is recommended. Allo-SCT only if there is no CAR-T option, good fitness and preferably as part of a study.

**Rationale:**
NRAS p.Q61R classified as Likely Oncogenic (Horak et al.). Literature (PMID:24335104,27458004) shows high prevalence in refractory MM and reduced sensitivity to PI (bortezomib). Therefore, avoid PI monotherapy; prioritize BCMA-targeted therapies (talquetamab was GPRC5D) and RAS/MAPK inhibitor trials; Allo-SCT only restrictive.

**===== run_2 =====**

**Tumor board decision:**
Priority: BCMA-directed therapy. Preferred BCMA CAR-T (e.g. cilta-cel/ide-cel) if available; alternatively BCMA bisspecifics (e.g. teclistamab) or BCMA ADC. Parallel/subsequent inclusion in clinical studies with RAS/MAPK inhibitors is recommended. Allo-SCT only if there is no CAR-T/study access and good transplant tolerance.

**Rationale:**
NRAS p.Q61R = Oncogenic (Horak +10). Literature (PMID:24335104, PMID:27458004) shows high prevalence of NRAS in refractory MM and reduced response to bortezomib alone -> avoid single-PI. NRAS justifies the review of RAS/MAPK-directed studies, but does not represent a contraindication for BCMA therapies.

**===== run_3 =====**

**Tumor board decision:**
NRAS p.Q61R is classified as an oncogenic. Primary recommendation: BCMA-directed therapy with priority - CAR-T (e.g. cilta-cel/ide-cel) if suitable; if not available/no CAR-T: BCMA-bispecifics (e.g. teclistamab) or participation in BCMA-directed studies/ADC. Avoid further proteasome inhibitor monotherapies. Allo-SCT only with selection criteria/study options or after CAR-T failure.

**Rationale:**
Classification: NRAS p.Q61R = Oncogenic (+10 points). Literature (PMID:24335104, 27458004) shows NRAS Q61 mutations are common in refractory MM and associated with reduced PI sensitivity; supports prioritization of BCMA immunotherapies and recruitment into MAPK-targeted trials. Use allo-SCT cautiously due to the risk of mortality.

## ============================================================ gpt-5-mini (plain) ============================================================

**===== run_1 =====**

**Tumor board decision:**
Recommendation: Rapid presentation/work-up on BCMA-directed CAR-T therapy (e.g. cilta-cel/ide-cel) if suitable. If bridging is necessary: short-cycle cytoreduction (dexamethasone +/- cyclophosphamide or pomalidomide + dexamethasone). If CAR-T is not available: BCMA bispecific antibody preparation (teclistamab) or study inclusion.

**Rationale:**
Patient with plasma cell leukemia, previously treated several times and refractory to PI, IMiD and anti-CD38; Progression after GPRC5D bispecific talquetamab. BCMA-CAR-T offers the highest chance of deep remission in highly refractory, rapidly progressive disease. Bridging/alternative recommended due to availability or disease burden.

**===== run_2 =====**

**Tumor board decision:**
Recommendation: Rapid evaluation and referral to BCMA-directed CAR-T therapy (e.g. cilta-cel) as the next therapeutic step. If CAR-T is not available/unsuitable: primarily BCMA-bispecific antibody preparation (e.g. teclistamab) or study inclusion. Until therapy: bridging for disease control (e.g. dexamethasone +/- cyclophosphamide/bendamustine).

**Rationale:**
Patient with plasma cellular leukemia after multiple previous therapies (PI, IMiD, anti-CD38, GPRC5D-bsAb). BCMA-directed therapy is mechanistically not cross-resistant and offers the best chance of a deep, sustained response in this situation. No high-risk cytogenetics; rapid disease control required.

**===== run_3 =====**

**Tumor board decision:**
Recommendation: Immediate presentation of BCMA-targeted CAR-T (cilta-cel) as the preferred option. If not available/contraindicated: use BCMA bispecific (teclistamab). Until definitive therapy bridging/cytoreduction with dexamethasone +/- cyto cycle (e.g. cyclophosphamide +/- pomalidomide). Do not re-use previous, ineffective substance classes (isatuximab, carfilzomib, talquetamab, lenalidomide, bortezomib).

**Rationale:**
Patient heavily pretreated including anti-CD38 and GPRC5D bispec.; BCMA-naïve. Plasma cell leukemia is aggressive - BCMA-CAR-T currently offers the highest chance of a deep/remitting effect. If unavailable, BCMA-Bispec. sensible alternative. Bridging necessary due to high tumor burden; Cytogenetics without high risk.

## ============================================================ gpt-5-nano (agent) ============================================================

**===== run_1 =====**

**Tumor board decision:**
Participation in clinical trial with Ras/MAPK inhibitors (e.g. MEK inhibitors) or Ras-targeted therapies in refractory MM; NRAS p.Q61R is considered Likely Oncogenic; currently no established standard option with clear superiority.

**Rationale:**
Classification: NRAS p.Q61R Likely Oncogenic (Horak 2022; Hotspot + Oncogenic + Computational). PubMed: Mulligan 2014 - NRAS p.Q61R reduces bortezomib sensitivity; Kortüm 2016 - Ras pathway mutations common in refractory MM; Ras-targeted therapies/MAPK inhibitors in trials; FISH: no relevant changes documented.

**===== run_2 =====**

**Tumor board decision:**
Likely Oncogenic NRAS p.Q61R. Therapy recommendation: Participation in clinical trial with MEK/ERK inhibition or Ras pathway combinations in refractory MM (experimental; no established standard). If no trial available, examine BCMA/GPRC5D targeting in the study context; Allograft only if there is a suitable risk.

**Rationale:**
Horak 2022: NRAS p.Q61R = Likely Oncogenic (9 points: Hotspot + Oncogenic + Computation); FISH negative. PubMed: Ras pathway mutations common in refractory MM; NRAS Q61R with reduced bortezomib response; Ras pathway inhibition/combinations to be explored, no approved targeted therapy. Priority: clinical trial; BCMA-/GPRC5D depending on availability.

**===== run_3 =====**

**Tumor board decision:**
NRAS p.Q61R is oncogenic. Therapy recommendation: Participation in a clinical study with Ras pathway inhibitors (MEK/ERK inhibitors) or Ras-targeted trials; no established NRAS-specific MM therapy. If no study access, consideration of BCMA/GPRC5D immunotherapy or allogeneic transplantation according to risk profile.

**Rationale:**
Classification Oncogenic according to Horak 2022 (+9). FISH unremarkable. PubMed shows Ras pathway mutations common in refractory MM and evidence of Ras-targeted trials; Direct approved NRAS-specific

therapies are lacking. Primary recommendation: Ras clinical trials; Additional options subject to availability.

## ============================================================ gpt-5-nano (plain) ============================================================

**===== run_1 =====**

**Tumor board decision:**
BCMA-CAR-T therapy (Cilta-cel) as the next step in progression after talquetamab; Check availability/approval; alternatively Ide-cel if Cilta-cel is not available.

**Rationale:**
Multiple refractory MM; CAR-T offers highest chance of CR/nCR and longer remission; Transplant history not excluded, provided organ functions remain OK.

**===== run_2 =====**

**Tumor board decision:**
BCMA-targeted therapy as next line: preferred CAR-T (ide-cel or cilta-cel) if available/suitable; alternatively belantamab mafodotin; if not available/suitable, pomalidomide+dexamethasone or selinexor+dexamethasone.

**Rationale:**
Progression with talquetamab requires target switching: BCMA targeting offers good remission rates; CAR-T or belantamab are appropriate options; review clinical trials; Consider transplant and organ status as well as toxic profile.

**===== run_3 =====**

**Tumor board decision:**
Recommend pomalidomide + dexamethasone (Pd) as next line.

**Rationale:**
After progression on talquetamab in severely predisposed MM, Pd is an established, active option after lenalidomide exposure. check compatibility; Participation in clinical trial preferred, if available.

## ============================================================ gpt-oss-20b (agent) =============================================================

**===== run_1 =====**

**Tumor board decision:**
KRAS inactive - NRASp.Q61R has a purely oncogenic effect and reduces bortezomib sensitivity. Recommendation: Inclusion in Ras-targeted research program (e.g. MEK inhibitor/NRAS-specific approach) or combination proteasome inhibitor + IMiD (carfilzomib+pomalidomide) to overcome PI resistance.

**Rationale:**
Horak score 10 -> Oncogenic. Literature shows NRAS mutations associate with PIK pathway resistance (PMID24335104;27458004). No FISH change. Ras-targeted trials offer the highest level of evidence in refractory MM. Alternative PI/IMiD intensifies therapy option.

**===== run_2 =====**

**Tumor board decision:**
Patient should be enrolled in a clinical trial targeting the NRAS-mediated MAPK pathway (e.g., trametinib +/- carfilzomib). If no trial, combine a non-bortezomib proteasome inhibitor with a MEK inhibitor. NRASp.Q61R confers PI resistance; MAPK blockade restores response.

**Rationale:**
Horak-onco score 10 (oncogenic). Literature (PMIDs24335104,27458004) shows NRASp.Q61R predicts bortezomib resistance and responds to MEK/ERK inhibition. No FISH alterations present. Thus a MAPK-targeted strategy is evidence-based and actionable.

**===== run_3 =====**

**Tumor board decision:**

For NRASp.Q61R (Oncogenic), we recommend participation in a phase I/II study with the MEK inhibitor trametinib (or possibly selinexor in combination with PI). Off-label trametinib + bortezomib/dexamethasone is also permitted.

**Rationale:**
NRASp.Q61R scores 10 points (Oncogenic). Literature (PMID24335104,27458004) shows that NRAS mutations induce bortezomib resistance and significantly increase sensitivity to PI/MEK inhibitors. Evidence: retrospective MM cohorts, phase II data. Therefore, officially suitable MEK therapy.

## ============================================================ gpt-oss-20b (plain) ============================================================

**===== run_1 =====**

**Tumor board decision:**
Continued treatment with daratumumab+pomalidomide+dexamethasone (DPd) as standard second-line option after talquetamab PD; with CAR-T therapy (ide-cel) available as the preferred next option.

**Rationale:**
Patient has received 8+ therapies, no anti-BCMA therapy, daratumumab pomalidomide-dexamethasone is well tolerated, shows high response rates in PD scenarios and is available; CAR-T should only be preferred after several lines and when available.

**===== run_2 =====**

**Tumor board decision:**
Recommendation: Daratumumab + pomalidomide + dexamethasone (DPd) as the next therapy, possibly in combination with selinexor if intolerance. Alternatively: Participation in CAR T-cell therapy (BCMA targeting) in a clinical study.

**Rationale:**
The patient progressed again after several lines (PAd, CAD, HD-Melphalan, ASCT, KRD, Isatuximab, Carfilzomib, Talquetamab). Daratumumab (CD38) and pomalidomide (IMiD) have not yet been used but offer high response rates in refractory MM. Selinexor can be added if DPd is not tolerated. CAR T-cell therapy (BCMA targeting) is the next innovative option in the 4th or 5th line, if available.

**===== run_3 =====**

**Tumor board decision:**
Start belantamab mafodotin 2.5mg/kg IV q3wks (or selinexor 80mg PO q2wks) as next line; consider CAR-T (ide-cel/cilta-cel) if available. Monitor keratopathy, cytopenias; stop at >=2-grade toxicity or progression.

**Rationale:**
Refractory to IMiDs, PIs, anti-CD38, GPRC5D bispecific; progressed after talquetamab. Belantamab (anti-BCMA ADC) is approved for >=4 lines and effective in heavily pretreated MM. Selinexor offers a non-PI/IMiD mechanism. CAR-T is curative if available.

## ============================================================ gpt-oss-120b (agent) ============================================================

**===== run_1 =====**

**Tumor board decision:**
Based on the oncogenic assessment of NRAS p.Q61R (10 points), therapy should be aimed at MAPK signaling pathway inhibition. Immediate inclusion in a study with a MEK inhibitor (e.g. selumetinib or trametinib) combined with a proteasome or IMiD component (e.g. pomalidomide) is recommended. Additionally, BCMA-CAR-T therapy should be examined as a standard option for refractory PCL.

**Rationale:**
NRAS Q61R is oncogenic (hotspot, functional evidence, absent in gnomAD). PubMed articles (PMID:24335104, 27458004) show reduced bortezomib efficacy and benefit of MEK/ERK inhibitors in RAS-mutant myeloma. Therefore, a MAPK inhibitor trial is the variant-specific evidence option; CAR-T remains an important, non-variant-specific therapy option.

**===== run_2 =====**

**Tumor board decision:**
Due to the oncogenic NRAS p.Q61R mutation (Oncogenic, +10 points), we recommend inclusion in a clinical trial that uses a MEK inhibitor (e.g. trametinib or binimetinib) in combination with an IMiD

(pomalidomide) and dexamethasone (Phase II/III, evidence level B). Alternatively, bortezomib should be avoided due to proven resistance and an alternative proteasome inhibitor (e.g. carfilzomib) should be used.

**Rationale:**
The NRAS Q61R mutation is oncogenic (hotspot, functional evidence, missing population). PubMed studies (PMID:24335104, PMID:27458004) show reduced bortezomib sensitivity and association with refractory myeloma; MAPK inhibition is discussed as an effective strategy. No treatment-relevant FISH results available.

**===== run_3 =====**

**Tumor board decision:**
Recommendation: Participation in a clinical trial with a MEK inhibitor (e.g. trametinib, selumetinib or cobimetinib) +/- proteasome inhibitor (e.g. bortezomib combination). If a study is not available, an off-label MEK inhibitor can be considered.

**Rationale:**
NRASp.Q61R is described according to Horak et al. Classified as Oncogenic (+10) in 2022. Literature (PMID:24335104, PMID:27458004) shows reduced bortezomib sensitivity and recommends Ras/MEK inhibition in refractory MM. No other FISH changes available.

## ============================================================ gpt-oss-120b (plain) ============================================================

**===== run_1 =====**

**Tumor board decision:**
We recommend transitioning to BCMA-directed therapy. A BCMA-CAR-T product (ide-cel or cilta-cel) is preferred if the approval criteria are met; alternatively, belantamab-mafodotin can be used.

**Rationale:**
The patient is triple class refractory (PI, IMiD, anti-CD38) and progressive after GPRC5D bispecific (talquetamab). No high-risk genetics, good performance and no contraindications for cell therapy. BCMA targeting delivers the highest efficacy at this advanced stage.

**===== run_2 =====**

**Tumor board decision:**
Further BCMA-directed therapy: Implementation of BCMA CAR T cell therapy (e.g. Ide-cel) after appropriate lymphocyte depletion; if not suitable, use belantamab-mafodotin.

**Rationale:**
Patient has progressive myeloma disease after >=7 lines of therapy (IMiD, PI, CD38 mAb, GPRC5D bispecific), no high-risk genetics, remains transplant eligible. BCMA targeting provides the highest expected response rate in this refractory setting.

**===== run_3 =====**

**Tumor board decision:**
Recommendation: Implement BCMA-targeted CAR-T therapy (e.g. Ide-celt) as the next systemic treatment. Alternatively: BCMA bisspecific (teclistamab) or selinexor-pomalidomide combination if CAR-T is not possible.

**Rationale:**
Patient has refractory MM after IMiD, PI, anti-CD38 and GPRC5D bispecific, without high-risk genetics. CAR-T offers the highest deep remission in multi-refractory disease; other options only if contraindicated.

## ============================================================= qwen3-32b (agent) =============================================================

**===== run_1 =====**

**Tumor board decision:**
Recommendation for study inclusion in RAS-targeted therapies and evaluation of MEK inhibitors. Avoiding bortezomib/IMiD monotherapies due to NRAS-p.Q61R-associated resistance.

**Rationale:**

The NRAS p.Q61R variant is classified as oncogenic (10 points) and is associated with resistance to bortezomib (PMID:24335104) and IMiDs/proteasome inhibitors (PMID:27458004). Targeted therapies for RAS mutations in myeloma are in clinical trials (e.g. MEK inhibitors), standard options are not evidence-based.

**===== run_2 =====**

**Tumor board decision:**
Recommendation of a MEK inhibitor-based combination therapy (e.g. trametinib) in a clinical trial due to the NRAS p.Q61R mutation and resistance to PIs/IMiDs

**Rationale:**
The NRAS p.Q61R mutation (Oncogenic, 10 points) correlates with resistance to bortezomib and IMiDs (PMID:24335104, 27458004). Direct RAS targeting therapies are not established; MEK inhibitors as a downstream target in the RAS pathway are being evaluated in clinical studies. Study inclusion for combination with new RAS modulators is recommended.

**===== run_3 =====**

**Tumor board decision:**
Recommendation: Therapy with MEK inhibitors (e.g. trametinib) or inclusion in clinical studies on RAS pathway targeting. Avoid bortezomib monotherapy due to NRAS-p.Q61R-associated resistance.

**Rationale:**
The NRAS p.Q61R variant is classified as oncogenic (10 points). PubMed evidence shows resistance to bortezomib and standard therapies. MEK inhibitors have preclinical activity in RAS-mutant tumors. Study inclusion should be urgently considered for refractory myeloma.

## ============================================================ qwen3-32b (plain) ============================================================

**===== run_1 =====**

**Tumor board decision:**
Recommendation: Combination therapy with teclistamab (BCMA-bispecific) + pomalidomide + dexamethasone

**Rationale:**
Patient showed PR to BCMA therapy (talquetamab) but PD. Teclistamab as an alternative BCMA targeting route in combination with new IMiD (pomalidomide) and corticoid. Previous resistance to lenalidomide and carfilzomib considered. No high-risk genetics present.

**===== run_2 =====**

**Tumor board decision:**
Recommendation: BCMA-targeted therapy (e.g. belantamab mafodotin) or BCMA-CAR-T cell therapy if appropriate

**Rationale:**
Patient underwent multiple cycles of therapy with PIs, IMiDs, anti-CD38/anti-GPRC5D antibodies and ASCT, all with PD. BCMA targeting (belantamab) is indicated for >=4 prior therapies. CAR T cells as an option with sufficient performance. No high-risk genetics present.

**===== run_3 =====**

**Tumor board decision:**
Recommendation: Selinexor in combination with dexamethasone or another active substance (e.g. pomalidomide) as the next therapeutic step. Evaluation of a clinical study on the therapeutic option.

**Rationale:**
The patient re-progressed after multiple lines including talquetamab. Selinexor (XPO1 inhibitor) is established in refractory-resistant cases and is not pretreated. Combination therapies optimize response.

## ============================================================ qwen3-next-80b (agent) ============================================================

**===== run_1 =====**

**Tumor board decision:**

Enrollment in a clinical trial targeting Ras/MEK pathway (e.g., trametinib) is recommended. Standard options (e.g., anti-CD38 antibodies, BCMA-targeted therapies) may be considered if no trial available.

**Rationale:**
NRAS p.Q61R is Oncogenic (10 pts). Literature shows resistance to bortezomib and high Ras pathway mutation prevalence in refractory MM. MEK inhibition is rational per PMID:24335104, 27458004. Clinical trial advised for targeted approach.

**===== run_2 =====**

**Tumor board decision:**
Recommend BCMA-targeted therapy (teclistamab or CAR-T cell therapy). Alternatively, selinexor. Consider clinical trials of MEK inhibitors.

**Rationale:**
NRAS p.Q61R oncogenic (10 points). Studies show reduced bortezomib sensitivity and need for therapies beyond IMiDs/PIs. BCMA therapies effective in refractory MM (PMID:24335104, 27458004).

**===== run_3 =====**

**Tumor board decision:**
Recommendation for study inclusion in a clinical trial with MEK inhibitor (e.g. trametinib) due to the oncogenic NRAS p.Q61R variant, which is associated with resistance to proteasome inhibitors and IMiDs.

**Rationale:**
NRAS p.Q61R is oncogenic (10 points according to Horak 2022). Studies show association with bortezomib and IMiD resistance (PMID:24335104, 27458004). MEK inhibitors are a possible targeted therapy option in clinical trials.

## ============================================================ qwen3-next-80b (plain) ============================================================

**===== run_1 =====**

**Tumor board decision:**
BCMA-targeted therapy (teclistamab or CAR-T cell therapy) recommended

**Rationale:**
Previous therapies (CD38, GPRC5D, PI, IMiD) failed; BCMA directed options are standard next steps in RRMM. Plasma cell leukemia requires aggressive therapy, BCMA targeting is indicated along multiple lines.

**===== run_2 =====**

**Tumor board decision:**
Initiate BCMA-targeted CAR T-cell therapy (e.g. Cilta-cel or Ide-cel).

**Rationale:**
Progression after talquetamab (GPRC5D-directed), BCMA-directed therapies (CAR-T) are next option. CAR-T shows high response rates in relapsed MM, particularly in plasma cell leukemia.

**===== run_3 =====**

**Tumor board decision:**
Recommend BCMA-targeted CAR-T therapy (e.g. idecabtagene vicleucel or ciltacabtagene autoleucel).

**Rationale:**
Patient has PD after multiple lines of therapy (including CD38 antibodies, GPRC5D bispecifics), but has not yet received BCMA-directed therapy. CAR-T offers high response rates in advanced MM. No known contraindications. Prioritize targeted, effective option after failure of all previous classes.

# mm_case_10__ALL

################################
#### CASE: 10

################################

**========================================================= gpt-5-mini (agent) =========================================================**

**===== run_1 =====**

**Tumor board decision:**
Recommendation: If t(11;14) (FISH 36%) is confirmed, give priority to a venetoclax-based regimen (e.g. venetoclax + dexamethasone +/- proteasome inhibitor) because of t(11;14)-selective effectiveness (phase II/subgroup findings). In parallel or as a consequence: BCMA-directed therapy (CAR-T, e.g. cilta-cel, or BCMA bisspecifics/ADC) preferably in a clinical trial. Consider allo-SCT only in young/fit patients after remission. Before deciding on therapy: repeat FISH/NGS, BCL2 test and trial screening.

**Rationale:**

**Rationale: t(11;14) (36%) is predictive of BCL2 dependence and response to venetoclax (Phase II and subgroup analyses). BCMA-CAR-T and bispecifics show high remission rates in refractory MM; suitable if there is no BCMA resistance yet. 1q-gain and del13q have a negative prognosis -> trial/highly effective therapy recommended.**

**===== run_2 =====**

**Tumor board decision:**
Recommendation: Prioritize BCMA-targeted cell therapy (ciltacabtagene autoleucel / idecabtagene vicleucel) if possible based on the patient. If rapid cytoreduction or CAR-T is not available: BCMA bispecific (e.g. teclistamab) or venetoclax-containing combination (due to t(11;14), off-label) as bridging. Prefer clinical studies. Allo-SCT only in young/fit people after CR and in studies; Consider high risk.

**Rationale:**

**Rationale: FISH shows t(11;14) (36%) - predictive of BCL2 dependency; Venetoclax is effective in phase II/subgroup analyses. Patient is BCMA-naïve -> approved BCMA-CAR-T and BCMA-Bispecials show high remission rates in R/R MM (Phase II/III). Plasma cell leukemia is high risk; Allo-SCT should only be tested selectively/clinically.**

**===== run_3 =====**

**Tumor board decision:**
Recommendation: 1) Primarily a BCMA-directed therapy (if available CAR-T e.g. cilta-cel/ide-cel or BCMA-bispecific such as teclistamab) as the next line (approval/phase II-III). 2) Alternative/parallel venetoclax-based therapy for t(11;14) (preferably in a study or off-label with monitoring). In cases of deep remission, consider allo-SCT as consolidation. Prefer clinical studies.

**Rationale:**
No somatic oncogene variants. FISH: t(11;14) (36%) - predictive of venetoclax responsiveness (Phase II/subgroup analyses). Gain 1q21 -> unfavorable. BCMA-CAR-T and BCMA-bispecifics show high ORR in refractory MM (Phase II/III, approvals). Allo-SCT only if remission has been achieved and suitable fitness; Studies preferred.

**========================================================= gpt-5-mini (plain) =========================================================**

**===== run_1 =====**

**Tumor board decision:**
Rapid referral to evaluation and primary therapy with BCMA-targeted CAR T-cell therapy (e.g. cilta- or ide-cel). If CAR-T is not available/unsuitable: primarily BCMA bispecific (e.g. teclistamab) or study option; If bridging is required, short-term systemic therapy (e.g. selinexor-containing regimen).

**Rationale:**

Aggressive plasma cell leukemic, multiple pretreated MM (refractory to PI, IMiD, anti-CD38 and GPRC5D bispecific). No prior BCMA therapy documented. BCMA-CAR-T currently offers the highest chance of deep/sustained remission; BCMA bispecific or clinical Study sensible alternatives; Bridging in case of delay.

**===== run_2 =====**

**Tumor board decision:**
Urgent referral to a specialized myeloma center for BCMA-directed therapy. Preference: CAR-T (cilta-cel/ide-cel) if suitable; if not available/eligible: BCMA bispecific antibody (e.g. teclistamab) or enrollment in the study. Until then, bridging therapy according to center consultation (e.g. prednisolone/Dex +/- cyclophosphamide or Pom-Dex).

**Rationale:**
Patient with plasma cellular leukemia has been treated multiple times (PI, IMiD, anti-CD38) and is progressing after GPRC5D bispecific talquetamab. BCMA-directed therapies currently have the most evidence/efficacy profile in this setting. Consider allo-SCT only in young, fit patients after remission.

**===== run_3 =====**

**Tumor board decision:**
Rapid presentation to a specialized center for evaluation and primary therapy with a BCMA-targeted CAR-T (e.g. cilta-cel/ide-cel) or alternatively BCMA-bispecific (teclistamab). If implementation is delayed, immediate bridge therapy e.g. pomalidomide + dexamethasone +/- cyclophosphamide or bortezomib + dexamethasone. Allo-SCT test in CR/VGPR and young, fit patient.

**Rationale:**
Patient with plasma cell leukemia has progressive disease after proteasome inhibitors, IMiD, anti-CD38 and GPRC5D bispecific. No documented BCMA therapy - BCMA-CAR-T offers the highest remission rates. Bridging necessary due to aggressive course; Allo-SCT only as a consolidation for a suitable candidate.

## ============================================================ gpt-5-nano (agent) ============================================================

**===== run_1 =====**

**Tumor board decision:**
Venetoclax-based therapy recommended (e.g. venetoclax + dexamethasone; if necessary + bortezomib) for t(11;14) MM; Off label; Evidence level IIb (Phase II); Prefer studies.

**Rationale:**
Classification: t(11;14) is relevant to therapy; Horak doesn't know any individual variants; FISH also shows high-risk profiles (1q21 gain, 13q14 del, 14q32 del). PubMed: Phase II data show good response rates in t(11;14) MM with venetoclax; Evidence level IIb. Allogeneic transplant as an option depending on fitness; Prefer trials.

**===== run_2 =====**

**Tumor board decision:**
No oncogenic/likely oncogenic variant identified. Due to high-risk cytogenetics (1q21 gain, del(13q), del(14q32), IgH rearr., t(11;14)) and progression after talquetamab: focus on clinical studies (BCMA-/GPRC5D-targeted therapies). Check allogeneic transplant if the condition is suitable; otherwise continued therapy according to study availability.

**Rationale:**
Horak variances are missing; cytogenetic high-risk factors present. No directly targetable mutation. PubMed Evidence Supports BCMA-/GPRC5D-targeted Therapies in Trials; Resistance mechanisms such as BCMA downregulation are possible. Allogeneic transplant worth considering in well-conditioned patients; prioritizes clinical trials based on progression after BCMA/GPRC5D targets.

**===== run_3 =====**

**Tumor board decision:**
Actionable: t(11;14) MM. Recommendation: Prioritize venetoclax-based therapy (e.g. venetoclax + dexamethasone; trials if necessary). Allogeneic transplantation as a consolidating option in a suitable situation capable of remission. Urgently consider participation in BCMA-/GPRC5D-targeted studies. 1q21 gain = high risk.

**Rationale:**
Assessment: Horak classification favors t(11;14) as oncogenic/therapy relevant. Evidence: Venetoclax approach in t(11;14) MM shows activity in phase II/III trials; 1q21 gain prognostically high risk; other CNAs are less directly targetable. prefer trials; AlloSCT makes sense depending on the condition.

## ============================================================== gpt-5-nano (plain) ==============================================================

**===== run_1 =====**

**Tumor board decision:**
BCMA-CAR-T therapy (ide-cel) as the next step; if not available/suitable, alternative treatment with belantamab mafodotin.

**Rationale:**
MM refractory to PI, IMiD, anti-CD38 and GPRC5D therapy; Progression with talquetamab. BCMA-CAR-T (ide-cel) offers the highest response rate with potentially long-lasting remission. Early CAR-T assessment makes sense; If CAR-T is not possible: belantamab mafodotin.

**===== run_2 =====**

**Tumor board decision:**
BCMA-CAR-T cell therapy (cilta-cel or ide-cel) as the next treatment option for progressive MM after >=3 lines; Check suitability, organize lymphapheresis, prefer CAR-T. If CAR-T is not available, belantamab mafodotin or pomalidomide dexamethasone as an alternative; If necessary, integration into clinical studies.

**Rationale:**
PD after Isa-Kd and talquetamab; BCMA targeting has not been used yet. Autologous transplant 2022. No high-risk genes. Good performance status/hopefully capable of therapy. CAR-T offers high depth of remission and potentially longer control; Check options and access, alternatives available.

**===== run_3 =====**

**Tumor board decision:**
Switching to pomalidomide + dexamethasone (Pd) as the next treatment regimen. If tolerable, a triple regimen with a proteasoma inhibitor (e.g. carfilzomib) may be used. Goal: continued remission or stable disease control after progression on talquetamab. Consideration of CAR-T or clinical studies if available.

**Rationale:**
Progression under talquetamab (GPRC5D) requires target switching. Pd is an established option after lenalidomide exposure, is mechanistically distinct and well tolerated. If possible, review CAR-T/targeted therapies or trials if available

## ============================================================== gpt-oss-20b (agent) ===============================================================

**===== run_1 =====**

**Tumor board decision:**
Venetoclax 400 mg PO daily (t(11;14) MM, Phase II COU-AA-556), if necessary combined with daratumumab; alternatively BCMA-CAR-T or Bispecific-T-cell-Engager-Trials.

**Rationale:**
Not Oncogenic/Likely Oncogenic Coded; t(11;14) is considered actionable for BCL-2 inhibition, evidence-based from Phase II studies; 1q21, 13q14, 11q22.3 no specific therapy. Recommendation based on gene status.

**===== run_2 =====**

**Tumor board decision:**
t(11;14)-positive MM: Venetoclax (BCL-2 inhibitor) in combination with lenalidomide/dexamethasone (RR>=60% Phase2, 2021-23). Alternatively, venetoclax + daratumumab/lenalidomide if patients are ineligible for proteasome inhibitors. In case of progression: entry in open studies (e.g. NCT04070021).

**Rationale:**

No somatic oncogenic variants found; FISH resonance t(11;14) indicates BCL-2 dependence. Phase 2 studies show conformal response with venetoclax-based. 1q21 gain or 13q14 deletions are not directly targeted; therefore no further monotherapy options.

**===== run_3 =====**

**Tumor board decision:**
t(11;14) (perf. BCL-2 target) -> Venetoclax in combination (e.g. POM+dexa) according to phase II data (BELLINI-MM). In the absence of response enrollment in BCMA targeting (CAR-T/bispecific). No other oncogenic variant identified.

**Rationale:**
No somatic oncogenic variants. FISH-t(11;14) offers a therapeutically relevant option. Venetoclax has phase II evidence (E2b). 1q21 gain, del13q14 prognostic, no specific therapy.

## ============================================================ gpt-oss-20b (plain) ============================================================

**===== run_1 =====**

**Tumor board decision:**
Start daratumumab + pomalidomide + dexamethasone (DPd) as the next therapy.

**Rationale:**
The patient is progressive after proteasome inhibitor, IMiD and anti-BCMA (talquetamab). DPd is approved for triple class exposed RRMM, shows high response rates and acceptable side effect profiles. There are no contraindications.

**===== run_2 =====**

**Tumor board decision:**
Start belantamab-mafodotin 2.5 mg/kg IV q3 weeks in combination with dexamethasone 20 mg PO daily 4 days (or, if not available, selinexor 40 mg PO daily 5 days + dexamethasone).

**Rationale:**
Patient has PR after talquetamab but 4/25 progression. Belantamab is the only approved anti-BCMA ADC that is effective after up to 6 lines of therapy and has no cross-resistance to talquetamab. Selinexor + dexamethasone is another established option in multi-refractory MM. Both regimens are well tolerated and aim for further remission.

**===== run_3 =====**

**Tumor board decision:**
Recommendation: Start a combination of belantamab mafodotin, pomalidomide and dexamethasone (BOMP regimen) as the next treatment option. If the response is inadequate or worsening, CAR-T therapy (ide-cel or cilta-cel) should be considered immediately.

**Rationale:**
The patient is triple class sensitive (IMiD, PI, anti-CD38) and has shown progression after talquetamab (BCMA monotherapy). Belantamab-mafodotin, a BCMA ADC, achieved an ORR of >50% in combination with pomalidomide/dexamethasone in the phase 2 study and is suitable for patients with prior BCMA therapy. If BOMP does not work, CAR T cells are the current standard option for multi-refractory MM. Secondary diagnoses (allergies, appendectomy) are not contraindications.

## ============================================================ gpt-oss-120b (agent) ============================================================

**===== run_1 =====**

**Tumor board decision:**
Recommendation: Implement BCMA-directed therapy, preferably BCMA CAR T cell therapy (e.g. idecabtagene vicleucel) as the next treatment option. If t(11;14) is present and BCL-2 expression is detectable, off-label therapy with venetoclax can be considered. Allogeneic stem cell transplantation only as part of a clinical trial.

**Rationale:**
No somatic oncogenic variants; FISH shows t(11;14) (therapy sensitivity for venetoclax) and 1q21 gain (high risk). The patient is refractory to >=4 lines of therapy including talquetamab. BCMA-CAR-T (KarMMa-3, Phase3) has been shown to provide clinical benefit and is approved for highly prevalent MM.

**===== run_2 =====**

**Tumor board decision:**
Due to the t(11;14)(IGH-CCND1) translocation, which is considered oncogenic and treatable, we recommend venetoclax (+/- dexamethasone) or inclusion in a venetoclax-based study. In addition, if not already done, BCMA-targeted CAR-T therapy (ide-cel or cilta-cel) should be considered. Allogeneic stem cell transplantation only as part of a clinical trial.

**Rationale:**
t(11;14) in MM associated with BCL-2 dependence; Phase 1/2 data and BELLINI results show ORR >50% with venetoclax. BCMA-CAR-T (ide-cel, cilta-cel) has FDA approval after >=3 prior therapies and delivers high response rates. Other FISH alterations (1q21-Amp, del13q, del14q32) have no directly targeted therapies.

**===== run_3 =====**

**Tumor board decision:**
Recommendation: start BCMA-directed therapy. Ide-cabtagenevicleucel (CAR-T) is approved based on MajesTEC-1 (Phase3) after multiple therapy, including talquetamab. Alternatively, participation in a BCMA bispecific study. Additionally, venetoclax-based therapy can be considered for t(11;14), but only as part of a clinical trial or off-label protocol.

**Rationale:**
FISH shows t(11;14) -> BCL-2 dependence (venetoclax sensitivity). 1q21 amplification and IGH rearrangements indicate aggressive disease behavior, benefit from BCMA targeting. BCMA-CAR-T (ide-celt) has significant ORR and OS improvement after >=3 previous lines, including bispecific GPRC5D approach (evidence level A).

## ======================================================== gpt-oss-120b (plain) ========================================================

**===== run_1 =====**

**Tumor board decision:**
Recommendation: Implementation of BCMA-targeted CAR-T therapy (e.g. Ide-celt) after appropriate lymphocyte depletion, if necessary bridging therapy, as part of an approved program.

**Rationale:**
The patient is triple class refractory (IMiD, PI, GPRC5D bispecific), has already had several HD melphalan SCTs and is progressing after talquetamab. Without high-risk genetics and with good performance, BCMA-CAR-T is the therapy with the highest expected remission rate; other options (belantamab, selinexor) are secondary.

**===== run_2 =====**

**Tumor board decision:**
We recommend BCMA-targeted CAR T-cell immunotherapy (e.g. idecabtagene vicleucel) as the next therapy if the patient meets the criteria for leukapheresis and lymphocytopenia management. If unsuitable, belantamab-mafodotin or selinexor+dexamethasone can be used as alternatives.

**Rationale:**
The patient is refractory to proteasome inhibitors, IMiDs, anti-CD38 antibodies and the GPRC5D bispecific talquetamab. After two ASCTs, he meets the eligibility criteria for BCMA-CAR-T, which provides the highest expected response rate in this setting.

**===== run_3 =====**

**Tumor board decision:**
Recommendation: Implementation of BCMA-targeted CAR-T therapy (e.g. idecabtagene vicleucel) or participation in a corresponding clinical study as the next therapy step.

**Rationale:**
The patient is progressive after multiple PI, IMiD and anti-CD38 based regimens as well as talquetamab therapy. No high-risk genetics, good organ function, no contraindication. CAR-T offers the highest expected deep remission in severely predisposed, refractory MM patients.

## ========================================================= qwen3-32b (agent) =========================================================

**===== run_1 =====**

**Tumor board decision:**
Recommendation: Therapy with CDK4/6 inhibitors (e.g. palbociclib) and BTK inhibitors (e.g. zanubrutinib) due to the t(11;14) translocation. Avoiding lenalidomide due to 14q32 deletion. Consider study inclusion in NCT03238821.

**Rationale:**
The t(11;14) translocation (oncogenic) can be addressed with CDK4/6 inhibitors (Phase II, NCT03238821) and BTK inhibitors. The 14q32 deletion (14q32.31) correlates with lenalidomide resistance. No classified somatic variants, but cytogenetic findings suggest targeted therapies.

**===== run_2 =====**

**Tumor board decision:**
Recommendation for therapy with palbociclib (CDK4/6 inhibitor) due to the t(11;14) translocation. Consider study inclusion in clinical trials of BCL-2 inhibitors or CAR T-cell therapy.

**Rationale:**
The t(11;14) (CCND1/FGFR3) is an established prognostic factor and therapeutic target for CDK4/6 inhibitors. Palbociclib (evidence level 2B) has shown efficacy in this translocation in studies. The 14q32 deletion (BCL6) could index BCL-2 targets. No classified variants according to Horak, but FISH-based therapy options exist.

**===== run_3 =====**

**Tumor board decision:**
Recommend bortezomib-based therapy in combination with lenalidomide and dexamethasone. Consider study inclusion in t(11;14)-specific trials.

**Rationale:**
The t(11;14) translocation is associated with CCND1 overexpression, bortezomib shows clinical activity in this subgroup. No direct target mutations according to Horak et al. identified.

## ============================================================ qwen3-32b (plain) ============================================================

**===== run_1 =====**

**Tumor board decision:**
Recommendation: BCMA bispecific antibody therapy (e.g. teclistamab) or clinical trial with CAR T cells. Pretreatment with talquetamab and isatuximab was taken into account.

**Rationale:**
Patient with refractory MM after multiple lines including ASCT and BCMA monotherapy. Teclistamab as a new BCMA targeting with synergistic potential. Clinical trials required with limited options.

**===== run_2 =====**

**Tumor board decision:**
Recommendation: Combination therapy with daratumumab, carfilzomib and dexamethasone (D-KRd) as the next therapeutic step.

**Rationale:**
The patient showed progression after multiple lines (PI, IMiD, BCMA targeting). D-KRd combines proven mechanisms of action (CD38 targeting, protease inhibition) and has been used successfully in refractory cases. Talquetamab and isatuximab have already been used, hence prioritizing a more established combination.

**===== run_3 =====**

**Tumor board decision:**
Recommendation: Combination therapy with talquetamab + carfilzomib + dexamethasone (TKD) as the next therapeutic step. If there is no response: evaluation for BCMA-targeted CAR-T cell therapy or clinical trials.

**Rationale:**
The patient demonstrated a PR after talquetamab monotherapy, highlighting the BCMA targeting relevance. Combination with carfilzomib (successful in previous KRD) and dexamethasone maximizes synergy. If PD recurs, innovative cell therapies (CAR-T) or bispecific antibodies are a priority.

## ========================================================== qwen3-next-80b (agent) ==========================================================

**===== run_1 =====**

**Tumor board decision:**
Venetoclax in combination with dexamethasone due to t(11;14) translocation (evidence level 2B). Alternatively: BCMA-targeted therapies (e.g. teclistamab, CAR T-cell therapy).

**Rationale:**
t(11;14) is a therapy-relevant FISH finding that is associated with venetoclax response (Chari et al., Blood 2020). NCCN guidelines recommend venetoclax for t(11;14)-positive RRMM. No somatic variants classified, FISH findings are crucial.

**===== run_2 =====**

**Tumor board decision:**
Venetoclax in combination with dexamethasone recommended due to t(11;14) translocation (evidence level 2A). Alternatively, BCMA-directed therapies (e.g. belantamab, CAR-T) after progression to talquetamab.

**Rationale:**
No somatic variants classified. FISH shows t(11;14) (36%) associated with venetoclax effectiveness in clinical trials (BELLINI, phase 2). BCMA targeting is another option after progressing to GPRC5D targeting.

**===== run_3 =====**

**Tumor board decision:**
Venetoclax in combination with dexamethasone recommended (evidence level 2A, NCCN 2023) due to the t(11;14) translocation.

**Rationale:**
FISH findings show t(11;14) (36%), a therapy-relevant cytogenetic. Venetoclax shows response in t(11;14)-positive myeloma in clinical trials (BELLINI, NEJM 2020) and NCCN guidelines (2A). No somatic variants classified, FISH findings warrant action.

## ========================================================= qwen3-next-80b (plain) =========================================================

**===== run_1 =====**

**Tumor board decision:**
BCMA-targeted CAR-T therapy (e.g. ciltacabtagene autoleucel) as the next therapeutic step

**Rationale:**
Patient with plasma cell leukemia and multiple relapsed MM after anti-CD38, proteasome inhibitor, IMiD and GPRC5D bispecific. BCMA-CAR-T offers high response rates in high-risk disease and is recommended by guidelines.

**===== run_2 =====**

**Tumor board decision:**
Recommend BCMA-targeted CAR T-cell therapy (e.g. ciltacabtagene autoleucel).

**Rationale:**
Patient has undergone multiple lines of therapy including CD38 and GPRC5D targeted therapies with no response. BCMA-targeted CAR-T therapies demonstrate high response rates in advanced RRMM in the absence of prior BCMA therapy. No relevant contraindications (hay fever/appendectomy irrelevant).

**===== run_3 =====**

**Tumor board decision:**
Recommendation: CAR T-cell therapy with ciltacabtagene autoleucel (Carvykti).

**Rationale:**
Patient triple-class refractory (PI, IMiD, anti-CD38, GPRC5D-directed). BCMA-directed therapy is the next option. CAR-T shows high response rates in heavily pretreated MM. No contraindications, therefore suitable.

# mm_case_11__ALL

################################
#### CASE: 11
################################

## ============================================================ gpt-5-mini (agent) ============================================================

**===== run_1 =====**

**Tumor board decision:**
Continuation of isatuximab + carfilzomib + dexamethasone as bridging; immediate evaluation/referral for BCMA-directed CAR-T (Cilta-cel). Immediate treatment of hypercalcemia, bone protective therapy (bisphosphonate or denosumab) and local radiotherapy for symptomatic osteolysis. No further high-dose therapy/auto-SCT now.

**Rationale:**
Patient is Len-refractory under maintenance, PI- and CD38-exposed and meets criteria for early BCMA-CAR-T. Ongoing IsaKd can serve as bridging until CAR-T is available. Bone complications and hypercalcemia require immediate supportive/local measures. Re-ASCT not recommended after short remission.

**===== run_2 =====**

**Tumor board decision:**
Continuation of the started isatuximab+carfilzomib+dexamethasone therapy exclusively as bridging; immediate referral for evaluation for BCMA-targeted CAR-T (Cilta-cel). Parallel acute measures: correction of hypercalcemia, immediate bone-protective therapy (zoledronate/denosumab) and palliative local radiotherapy for symptomatic osteolysis. No immediate second high-dose therapy/auto-SCT.

**Rationale:**
The patient is refractory to lenalidomide and already exposed to CD38 and PI (functional triple-class exposed) - early BCMA-CAR-T is in accordance with the guidelines. Ongoing IsaKd can be continued as bridging but must not delay CAR-T production. Re-transplantation is not recommended for such an early recurrence. Immediate symptomatic/osteoprotective measures are mandatory.

**===== run_3 =====**

**Tumor board decision:**
Indication for BCMA-targeted CAR-T therapy (Cilta-cel) - immediate clarification/registration. Continue the already started Isa+Carfilzomib+Dex therapy as bridging if tolerated. In parallel: immediate bone-protective therapy (zoledronate or denosumab), acute therapy for hypercalcemia and local radiotherapy for painful osteolysis. Re-HD-Melphalan/2. ASCT is not primarily recommended.

**Rationale:**
Patient is Len-refractory after first-line (D-VRd, HD-Mel), PI-exposed -> candidate for BCMA-CAR-T (branch 1). Running IsaKd serves as acceptable bridging. Immediate osteoprotection/RT and hypercalcemia treatment are mandatory. Second autologous SCT does not appear primarily after a short interval and the CAR-T option is available.

## ============================================================ gpt-5-mini (plain) ============================================================

**===== run_1 =====**

**Tumor board decision:**
Carfilzomib is not recommended due to CVI grade III and increased cardiovascular risk. Immediate treatment of hypercalcemia (IV volume, bisphosphonate/denosumab, if necessary calcitonin). Systemic myeloma therapy: pomalidomide + dexamethasone +/- cyclophosphamide (PCd). Parallel clarification for BCMA therapy/study or anti-CD38 only if sensitivity is documented.

**Rationale:**
Patient is lenalidomide exposed/refractory; Pomalidomide-based therapy is standard in this situation. Carfilzomib has relevant cardiovascular toxicity and is risky in grade III CVI. Hypercalcemia requires immediate supportive treatment. Anti-CD38 rechallenge only if sensitivity is proven; otherwise check BCMA-directed options.

**===== run_2 =====**

**Tumor board decision:**
Continuation of the initiated therapy isatuximab + carfilzomib + dexamethasone with rapid response monitoring after 2 cycles. Treat acute hypercalcemia immediately (hydration, calcitonin if necessary), start bisphosphonate or denosumab and skeletal protection promptly. If there is no response/further progression: immediate evaluation for BCMA-directed therapy (CAR-T or bispecific) or inclusion in a clinical study.

**Rationale:**
Patient progressed after induction+D-VRd, HD-melphalan/auto-SCT and LEN maintenance. Isatuximab+carfilzomib represents a sensible salvage option (IMiD and PI already given; anti-CD38 acceptable after completion of primary therapy). Hypercalcemia/skeletal manifestation requires immediate supportive measures. In cases of refractoriness, BCMA-directed therapy is the evidence-based next line.

**===== run_3 =====**

**Tumor board decision:**
Discontinue isatuximab (class redundancy to prior daratumumab exposure). Switch to pomalidomide + bortezomib + dexamethasone (PVd). If bortezomib is contraindicated, pomalidomide + cyclophosphamide + dexamethasone. In parallel: immediate evaluation/referral for BCMA-directed therapies (CAR-T or bispecific antibodies) as well as acute treatment of hypercalcemia (IV volume, bisphosphonate/denosumab).

**Rationale:**
Patient is lenalidomide refractory and exposed to daratumumab; a second anti-CD38 is expected to be ineffective. Pomalidomide-based triplets show good effectiveness after Len refractoriness. We avoid carfilzomib (cardiovascular risk) because of CVI grade III. BCMA therapies are the next potentially long-term effective option; Treat hypercalcemia urgently.

## ================================================================ gpt-5-nano (agent) ================================================================

**===== run_1 =====**

**Tumor board decision:**
BCMA-CAR-T therapy (Cilta-cel preferred) as the next measure in case of progression under IsaKd. Continue bridging with IsaKd/DaraKd. If CAR-T is not possible: non-CD38 regimens KCd or PVd; Treat hypercalcemia.

**Rationale:**
progression under CD38-AK + PI; R-ISS II; triple class exposed. CAR-T offers the best chance; Bridging makes sense; Alternatives only in case of unavailability/intolerance.

**===== run_2 =====**

**Tumor board decision:**
BCMA-CAR-T therapy (Cilta-cel or Ide-cel) as primary next-gen therapy for progression on IsaKd after auto-SCT. Bridging therapy without CD38-AK (e.g. PVd/KCd) until infusion. If CAR-T is not available: BCMA bispecifics (teclistamab/elranatamab) or non-CD38 combinations (VCd/KCd).

**Rationale:**
Progression under IsaKd; CD38 refractoriness; triple class exposed. CAR-T offers highest response rate/durable remission; Early access recommended. Bridging makes sense without CD38-AK; alternative BCMA or non-CD38 regimens if unavailable.

**===== run_3 =====**

**Tumor board decision:**
BCMA-CAR-T therapy (Cilta-cel or Ide-cel) as the next therapeutic goal after IsaKd progression; Bridging with non-BCMA regimens (e.g. DaraPd or KCd/VPd) until CAR-T infusion; If CAR-T is not possible, consider teclistamab or elranatamab or PVd/KCd; Check studies.

**Rationale:**
CD38 progression under IsaKd; triple-exposed MM (PI, IMiD, CD38); CAR-T against BCMA offers highest probability of remission; Bridging necessary. If CAR-T is not achievable, consider alternatives such as teclistamab/elranatamab or PVd/KCd; clinical studies make sense.

## ========================================================== gpt-5-nano (plain) ==========================================================

**===== run_1 =====**

**Tumor board decision:**
Recommendation: Switch to pomalidomide + dexamethasone (Pd) as the next line of RRMM after progression on isatuximab + carfilzomib + Dex. If rapid remission is necessary or tolerated, consider PVd (pomalidomide + bortezomib + Dex); review clinical trials.

**Rationale:**
Progression under IsaCfDex; Lenalidomide exposure is present; Changing the active ingredient classes makes sense; Pomalidomide shows activity after Len exposure; no high-risk gentopias; SCT completed; Trials possible.

**===== run_2 =====**

**Tumor board decision:**
BCMA-targeted CAR-T therapy (Cilta-cel or Ide-cel) as the next treatment for progression under Isa-Kd after AutoSCT and Len maintenance. If CAR-T cannot be achieved in a timely manner, alternatively Pom-dex or Selinexor-dex as bridging therapy.

**Rationale:**
Patient is triple pretreated, progressive despite anti-CD38+PI; no high-risk FISH; The goal is deep remission/PFS extension. CAR-T against BCMA offers the highest chances of remission and PFS in this setting; options available; Pay attention to availability/bridging.

**===== run_3 =====**

**Tumor board decision:**
BCMA therapy preferred: CAR-T Cilta-cel or Ide-cel; Alternative belantamab mafodotin; if not accessible, pomalidomide+dexamethasone. Adequately treat hypercalcemia; Bone protection (bisphosphonates/denosumab) and RT as needed.

**Rationale:**
Progression under Isa-Kd; no BCMA pretreatment; BCMA approach offers realistic chances of remission after anti-CD38, accompanied by supportive measures.

## ========================================================== gpt-oss-20b (agent) ===========================================================

**===== run_1 =====**

**Tumor board decision:**
Continuation of isatuximab/carfilzomib/dexamethasone combination (ICd) as next therapy.

**Rationale:**
Patient has RRMM after D-VRd, Mel200, auto-SCT, LEN; ICd shows ORR~70% in phase 2 studies, no contraindications. Alternative BVd carries ocular risks, D-K-Rd less data for RRMM. ICd is the evidence-based choice.

**===== run_2 =====**

**Tumor board decision:**
Continuation of the IsaKd bridge until CAR-T infusion (cilta-cel or ide-cel) - maintain IsaKd as bridging therapy, plan CAR-T as definitive therapy.

**Rationale:**
Patient triple-class exposed, Len-refractory; Guidelines recommend immunotherapy. IsaKd acts quickly and can serve as bridging to CAR-T; Carfilzomib cardiotoxicity minimal in CVI-III. CAR-T offers long-term remission.

**===== run_3 =====**

**Tumor board decision:**
We recommend switching from isatuximab/carfilzomib/dexamethasone to daratumumab/carfilzomib/lenalidomide (D-KRd) as the next phase of therapy.

**Rationale:**
No clinical evidence for isatuximab+carfilzomib+Dex; Studies (CANDOR, IKEMA) show higher ORR and PFS in RRMM with carfilzomib + daratumumab + lenalidomide. Patient has no high-risk genetics, can

tolerate carfilzomib and requires rapid control of hypercalcemia.

## ======================================================= gpt-oss-20b (plain) =======================================================

**===== run_1 =====**

**Tumor board decision:**
We recommend discontinuing carfilzomib due to CVI grade III and reducing the current Isa-Car-D regimen to isatuximab+dexamethasone. Perform response assessment after 4-6 cycles; if progression occurs, immediately switch to daratumumab+pomalidomide+dexamethasone (DPd).

**Rationale:**
Patient already has D-VRd, ASCT, Len-Maintenance, now IsaCarD. CVI-Grade III increases the risk of carfilzomib-associated heart failure. Isatuximab+Dex remains effective; If there is no remission, DPd is the established 3-line option without further CV risks.

**===== run_2 =====**

**Tumor board decision:**
Further treatment with daratumumab + pomalidomide + dexamethasone (Dara-Pd) as 2nd line after lenalidomide maintenance. Carfilzomib should be avoided in CVI grade III. Dara-Pd has high efficacy and acceptable tolerance after lenalidomide and proteasome inhibitor.

**Rationale:**
Patient has PD with hypercalcemia after lenalidomide maintenance. No high-risk cytogenetic factors are present. Daratumumab-based triplet (Dara-Pd) is evidence-based, works independently of previous proteasome inhibitors and is better tolerated than carfilzomib in CV comorbidities.

**===== run_3 =====**

**Tumor board decision:**
Continuation of isatuximab-carfilzomib-dexamethasone (ICD) regimen with bisphosphonate therapy and hypercalcemia control. In case of progression: daratumumab-pomalidomide-dexamethasone or clinical trial.

**Rationale:**
Patient is in RRMM after ASCT and lenalidomide maintenance. ICD is evidence-based 3-drug therapy for 2nd or 3rd line. Hypercalcemia is treated with corticosteroids, hydration, and bisphosphonates. In case of progression, daratumumab-pomalidomide-dexamethasone or clinical trials are the next option.

## ======================================================= gpt-oss-120b (agent) =======================================================

**===== run_1 =====**

**Tumor board decision:**
We recommend continuing the planned switch to isatuximab+carfilzomib+dexamethasone (Isa-Kd) as the next treatment regimen. If carfilzomib is intolerable or after further progression, a BCMA bispecific antibody therapeutic (e.g. teclistamab) or a clinical trial should be considered.

**Rationale:**
The patient has already received daratumumab (D-VRD), so a further CD38 change with isatuximab is evidence-based effective (Isa-Kd after daratumumab failure). Carfilzomib provides rapid calcium control and lenalidomide resistance is already present. BCMA bispecifics show ORR~60% in triple-class refractory patients and represent a useful alternative, especially in cases of cardiovascular stress caused by carfilzomib.

**===== run_2 =====**

**Tumor board decision:**
We recommend the indication for BCMA-targeted CAR-T therapy (Cilta-cel) and continue the current Isa-Kd therapy as bridging until infusion. If CAR-T is not available, choose a pomalidomide-based regimen (e.g. PVd or Isa-Pd) as the next line.

**Rationale:**
The patient is triple class exposed (CD38 Ab, PI, IMiD) and shows progressive MM with hypercalcemia. CAR-T offers the highest expected deep remission. Isa-Kd is tolerable, serves as bridging and avoids immediate repetition of a CD38-AK in the event of possible refractoriness. For cardiovascular risk (CVIIII), a pomalidomide-based regimen is a safe alternative.

**===== run_3 =====**

**Tumor board decision:**
We recommend continuing the planned therapy with isatuximab+carfilzomib+dexamethasone (Isa-Kd), as it represents the evidence-based standard option for RRMM after lenalidomide maintenance according to IKEMA data. In cases of severe cardiac stress (CVIIII), daratumumab+pomalidomide+dexamethasone can be considered as an alternative.

**Rationale:**
The patient has already received proteasome inhibitor (bortezomib), IMiD (lenalidomide) and alkylator, but not yet CD38 MAB therapy. Isa-Kd achieved high ORR in the IKEMA trial and prolonged PFS in R-ISSII patients. BCMA bispecifics are only recommended after triple class exposure. DKd is effective, but carfilzomib-related cardiotoxicity should be considered in CVIIII.

## ============================================================ gpt-oss-120b (plain) ============================================================

**===== run_1 =====**

**Tumor board decision:**
Continuation of current therapy with isatuximab+carfilzomib+dexamethasone (Isa-Kd) as 2nd line, accompanied by bisphosphonates and calcium control. If there is no/insufficient response after 2-3 cycles, immediately switch to BCMA-directed therapy (CAR-T or bispecific).

**Rationale:**
Patient has progressive MM after lenalidomide maintenance, hypercalcemia and no contraindications to CD38 antibody or proteasome inhibitor. Isa-Kd is evidence-based 2nd line; If there is no response, an early switch to BCMA therapy is recommended.

**===== run_2 =====**

**Tumor board decision:**
Continuation of the therapy started with isatuximab + carfilzomib + dexamethasone (Isa-K-Dex) as the next step. If there is no clinical or serological response after 2-3 cycles, switch to a pomalidomide-based triplet (e.g. Pom-Dara-Dex) or, if available, to a BCMA-directed therapy (CAR-T/Bispecific) in a study or compartment setting.

**Rationale:**
Patient is lenalidomide refractory, has already received exposure to bortezomib and daratumumab, but has not received carfilzomib or isatuximab therapy. According to current guidelines, Isa-K-Dex is the recommended triplet option for RRMM after len refractoriness. Comorbidities (Basedow, CVIIII) do not constitute a contraindication. If there is no response, switching to pomalidomide-based regimens or BCMA targeting is the evidence-based alternative.

**===== run_3 =====**

**Tumor board decision:**
Continuation of the therapy started with isatuximab+carfilzomib+dexamethasone (Isa-Kd) and evaluation of the response after 2 cycles. In case of insufficient response or renewed progression, switch to a pomalidomide-based triple (pomalidomide+carfilzomib+dexamethasone) or BCMA-directed therapy (belantamab-mafodotin or CAR-T, if suitable).

**Rationale:**
Patient has already received 1st line (D-VRD) + ASCT and lenalidomide maintenance; Progression requires an effective CD38 antibody combination. Isa-Kd is the current standard for RR-MM after pretreatment. If there is no response, pomalidomide-based or BCMA therapies are the next options, with the presence of CVI and lack of high-risk cytogenetics favoring tolerability.

## ============================================================ qwen3-32b (agent) ============================================================

**===== run_1 =====**

**Tumor board decision:**
Indication for BCMA-targeted CAR T-cell therapy (Cilta-cel). Continuation of bridging therapy with IsaKd.

**Rationale:**

Progression under Isa/Carfilzomib suggests triple class refractory (IMiD, PI, CD38). Cilta-cel is approved in the 2nd line, even without prior exposure to CD38. Manage hypercalcemia with intensive fluid regimens and bisphosphonates.

**===== run_2 =====**

**Tumor board decision:**
Indication for BCMA-targeted CAR T-cell therapy (Cilta-cel). Continuation of bridging therapy with IsaKd. Hypercalcemia: IV fluids, zoledronate, calcitonin if necessary.

**Rationale:**
Patient is triple class exposed (IMiD, PI, CD38) with PD on IsaKd. CAR-T (Cilta-cel) is approved in the 2nd line. Bridging with IsaKd due to tolerated therapy. Manage hypercalcemia according to guidelines.

**===== run_3 =====**

**Tumor board decision:**
Therapy adjustment to D-KR (without dexamethasone) for PD treatment, combined with zoledronate to reduce hypercalcemia. If progression occurs, consider BCMA-targeted therapies.

**Rationale:**
PD on isatuximab/carfilzomib/dexamethasone requires switching to D-KR (dexamethasone-sparing). Hypercalcemia is addressed by zoledronate and dexamethasone reduction. BCMA targeting (e.g. teclistamab) as the next option for recurrent PD.

## ============================================================ qwen3-32b (plain) ============================================================

**===== run_1 =====**

**Tumor board decision:**
Recommendation: Therapy with daratumumab, pomalidomide and dexamethasone (D-Pd) as the next step. In case of resistance to therapy against IMiD/proteasome inhibitors and anti-CD38 antibodies.

**Rationale:**
The patient is refractory to lenalidomide, carfilzomib and isatuximab. D-Pd is an established option in triple-class refractoriness. CVI III requires careful dexamethasone dosing.

**===== run_2 =====**

**Tumor board decision:**
Further therapy with daratumumab, pomalidomide and dexamethasone (D-Pd) is recommended.

**Rationale:**
The patient is refractory to lenalidomide, proteasome inhibitors, and isatuximab. D-Pd is an established option for lenalidomide and proteasome inhibitor-refractory MM. The lack of high-risk cytogenetics favors the prognosis. Hypercalcemia requires accompanying symptom control.

**===== run_3 =====**

**Tumor board decision:**
Recommendation: Therapy with pomalidomide, daratumumab and dexamethasone (Pd-Dex) as the next step. In case of progression under current therapies (carfilzomib, isatuximab) and lenalidomide refractoriness, a triplet with pomalidomide (new IMiD), daratumumab (alternative anti-CD38) and dexamethasone is evidence-based.

**Rationale:**
The patient is refractory to lenalidomide, proteasome inhibitors (bortezomib, carfilzomib), and isatuximab. Pd-Dex is standard for triple-refractory MM. CVI III requires IV medications (daratumumab). No contraindications for pomalidomide.

## ============================================================ qwen3-next-80b (agent) ============================================================

**===== run_1 =====**

**Tumor board decision:**
Indication for BCMA-targeted CAR-T cell therapy (Ciltacabtagene Autoleucel). Continuation of bridging therapy with isatuximab, carfilzomib and dexamethasone.

**Rationale:**
The patient is lenalidomide-refractory, previously treated with PIs (bortezomib + carfilzomib) and previously treated with CD38-AK (daratumumab + isatuximab), thus triple-class exposed. Cilta-cel is approved from the 2nd line with these criteria. IsaKd is tolerated and continued as bridging as no BCMA-directed bridging therapy is required.

**===== run_2 =====**

**Tumor board decision:**
Indication for CAR T-cell therapy with Ciltacabtagene Autoleucel (Cilta-cel). Bridging therapy with VCd (bortezomib, cyclophosphamide, dexamethasone) until CAR-T infusion.

**Rationale:**
The patient is lenalidomide-refractory, PI-pretreated and CD38-AK-pretreated (triple-class exposed). Progression under IsaKd requires switching. According to the flowchart, CAR-T is indicated from the 2nd line. VCd as bridging, since carfilzomib is already progressive and CD38-AK exposure no longer makes sense. No contraindications for VCd (Graves' disease, CVI grade III).

**===== run_3 =====**

**Tumor board decision:**
Indication for BCMA-targeted CAR-T cell therapy (Ciltacabtagene Autoleucel). Continuation of bridging therapy with isatuximab, carfilzomib and dexamethasone.

**Rationale:**
The patient is lenalidomide-refractory, PI-pretreated (D-VRd, carfilzomib) and CD38-Ab-exposed (D-VRd, IsaKd). Triple class refractory. Cilta-cel is approved from the 2nd line with these criteria. Continue IsaKd as tolerated bridging therapy as it is not a BCMA-targeting substance. No contraindications (CVI, Graves) for CAR-T.

## ============================================================ qwen3-next-80b (plain) ============================================================

**===== run_1 =====**

**Tumor board decision:**
Continuation of current therapy with isatuximab, carfilzomib and dexamethasone. Regular response assessment after 2-3 cycles. In case of progression, switch to BCMA-directed therapy (e.g. belantamab mafodotin) or clinical trial.

**Rationale:**
Current therapy is standard upon progression to lenalidomide. Continuation indicated without evidence of progression or toxicity. BCMA-targeted therapies are an established option for progression to anti-CD38-based therapies.

**===== run_2 =====**

**Tumor board decision:**
Continuation of current therapy with isatuximab, carfilzomib and dexamethasone. Response evaluated after 4-6 cycles.

**Rationale:**
PD on lenalidomide maintenance, therefore therapy with isatuximab/carfilzomib/dexamethasone initiated. Current therapy is continued, response evaluated after 4-6 cycles. No contraindications due to secondary diagnoses (Graves, CVI).

**===== run_3 =====**

**Tumor board decision:**
Continuation of current therapy with isatuximab, carfilzomib and dexamethasone with sustained response rate and tolerability.

**Rationale:**
Progression to lenalidomide maintenance required therapy with isatuximab/carfilzomib/dexamethasone. No evidence of treatment failure or contraindications due to Graves' disease/CVI. Standard procedure for sustained response rate.

# mm_case_12__ALL

################################
#### CASE: 12

################################

## ================================================================ gpt-5-mini (agent) ================================================================

**===== run_1 =====**

**Tumor board decision:**
Recommendation: Change/optimize induction to Dara-VRd (daratumumab s.c. + bortezomib + lenalidomide + Dex) for a total of 3-4 cycles, then stem cell mobilization and high-dose therapy with autologous SCT (melphalan 200 mg/m2) as consolidation, then lenalidomide maintenance (10 mg). Thrombosis prophylaxis and osteoprotection are mandatory.

**Rationale:**
R2-ISS III with CRAB indication and no evidence of high-risk aberrations -> patient presumably capable of HDT. Guideline preferred induction regimen for transplant candidates is anti-CD38 + PI + IMiD (Dara-VRd) for deeper remission before SCT. Hypothyroidism speaks for s.c. Daratumumab. Plan stem cell harvest before prolonged exposure to lenalidomide.

**===== run_2 =====**

**Tumor board decision:**
Recommendation: For transplantable R2-ISS III MM, change the induction to DaraVRd (anti-CD38 + bortezomib + lenalidomide + Dex) for a total of 3-4 cycles, then high-dose therapy (melphalan 200 mg/m2) + autologous SCT. Consider tandem SCT in cases of high risk or lack of CR. Followed by maintenance lenalidomide 10 mg. If switching is not possible: continue the started DaraVCD as bridging until ASCT.

**Rationale:**
R2-ISS III represents high risk; Anti-CD38 + VRd induction results in deeper response/MRD negativity before ASCT. ASCT is standard in transplant-eligible patients; Tandem SCT is recommended for high-risk patients. Lenalidomide maintenance improves PFS No clear contraindication (hypertension/hypothyroidism) to proposed therapy. Thromboprotection and osteoprotection are mandatory.

**===== run_3 =====**

**Tumor board decision:**
Continuation of the ongoing induction regimen (Dara-VCD) until completion of 3-4 cycles; Stem cell mobilization (G-CSF +/- plerixafor) after cycle 3; High-dose therapy with melphalan 200 mg/m2 and autologous SCT as consolidation; followed by lenalidomide maintenance (10 mg). Daratumumab s.c. consider for hypothyroidism; Thrombosis, infection and osteoprotection prophylaxis is mandatory.

**Rationale:**
R2-ISS III with CRAB findings requires intensive therapy. Dara-VCD is a guideline-compliant induction for patients suitable for HD; ASCT improves PFS/OS. Lenalidomide maintenance is standard. Pay attention to mobilization under Dara (G-CSF +/- Plerixafor); s.c. Administration reduces respiratory reactions.

## ================================================================ gpt-5-mini (plain) ================================================================

**===== run_1 =====**

**Tumor board decision:**
Continuation of the started induction with Dara-VCD (4 cycles in total if tolerable). Treat acute hypercalcemia immediately: IV volume, loop diuretic if necessary, immediate administration of zoledronic acid (denosumab for severe renal insufficiency). Start of monthly bisphosphonate/antiresorptive therapy, pain therapy, radiation if fracture is imminent, thrombosis prophylaxis. After 2-4 cycles, response assessment and clarification for stem cell transplantation/mobilization.

**Rationale:**

Patient with symptomatic MM (anemia, multiple osteolysis, hypercalcemia) started Dara-VCD correctly. Continuation as standard induction is recommended. Antiresorptive therapy and acute hypercalcemia treatment as well as local radiotherapy/supportive measures are mandatory. After induction, decision on ASCT based on response and comorbidities.

**===== run_2 =====**

**Tumor board decision:**
Continuation of the induction that has begun with Dara-VCD (4 cycles in total), reevaluation after cycles 3-4; If suitable, plan an autologous stem cell transplant. Supportive measures: skeletal protection (IV bisphosphonate/denosumab), acute hypercalcemia treatment, HSV prophylaxis (acyclovir) and thrombosis prophylaxis.

**Rationale:**
Newly diagnosed MM IgG kappa with indication for therapy (anemia, osteolysis, hypercalcemia), ISS/R2-ISS III. No high-risk cytogenetics known. Already initiated Dara-VCD is an appropriate first-line therapy; Reevaluation after induction for further therapy planning (SCT) and immediate supportive measures are indicated.

**===== run_3 =====**

**Tumor board decision:**
Continuation of the started induction with DaraVCD (daratumumab + bortezomib + cyclophosphamide + dexamethasone) for a total of 4 cycles. Parallel: acute therapy of hypercalcemia (IV volume, bisphosphonate or denosumab), monthly antiresorptive therapy, VZV prophylaxis (acyclovir), pain/orthopedic evaluation +/- palliative RT. If suitable for transplant: stem cell mobilization/collection after 3-4 cycles; Re-staging afterwards.

**Rationale:**
Symptomatic ISS III MM with anemia, osteolysis, and hypercalcemia warrants rapid tumor-reducing induction. DaraVCD is suitable as first-line therapy; Supportive measures address acute complications and reduce skeletal morbidity. No known contraindication; Review ASCT plan for response.

## ================================================================ gpt-5-nano (agent) ================================================================

**===== run_1 =====**

**Tumor board decision:**
ASCT-indicated: high-risk MM after DaraVCD induction; AutoSCT with melphalan 200 mg/m2 as consolidation; if necessary, tandem SCT if CR is inadequate; then lenalidomide maintenance. For painful lesions, RT; Treat hypercalcemia/anemia symptomatically.

**Rationale:**
High risk (ISS III, R2-ISS III) with active osteolysis/hypercalcemia; ASCT standard in transplantable patients; RT useful for focal lesions; supportive care essential.

**===== run_2 =====**

**Tumor board decision:**
ASCT-indicated after DaraVCD: high-dose autologous SCT; if necessary, tandem SCT if high risk; then lenalidomide maintenance. Radiation therapy for focal osteolysis if necessary; Treat hypercalcemia/anemia acutely (hydration, denosumab/bisphosphonate, transfusion). Prophylaxis: osteoprotection, infection and thrombosis prophylaxis.

**Rationale:**
High-risk profile (ISS III, R2-ISS III) and residual osteolysis/hypercalcemia/anemia after induction; SCT offers the best long-term control. Radiotherapy useful for focal lesions; Subsequent supportive care to stabilize and maintain performance.

**===== run_3 =====**

**Tumor board decision:**
ASCT timing: After DaraVCD induction, rapid evaluation for high-dose therapy with autologous SCT (melphalan 200 mg/m2). If the response is good, individual/tandem SCT may be necessary. Then lenalidomide maintenance. Radiotherapy for symptomatic osteolysis. Treat hypercalcemia/anemia aggressively (hydration, bisphosphonate/denosumab, transfusion).

**Rationale:**

High-risk MM (ISS III, R2-ISS III) + CRAB criteria. Induction suitable; High-dose autologous therapy maximizes CR/survival. Tandem SCT for suboptimal CR is useful. Maintenance supports long-term remission. RT for osteolysis to relieve symptoms; Aggressive treatment of hypercalcemia/anemia required.

## ============================================================ gpt-5-nano (plain) ============================================================

**===== run_1 =====**

**Tumor board decision:**
Switch to KRd (carfilzomib, lenalidomide, dexamethasone) or Pd (pomalidomide, dexamethasone) depending on availability/tolerability; If necessary, check autologous SCT after response; Correct hypercalcemia and continue supportive measures.

**Rationale:**
Progress under DaraVCD; new active principle needed; no high-risk cytogenetics; Aim for remission and, if necessary, SCT.

**===== run_2 =====**

**Tumor board decision:**
Transplant-eligible MM after DaraVCD induction: high-dose therapy followed by autologous stem cell transplantation (ASCT) followed by lenalidomide maintenance. If transplant inability: continue DaraVCD maintenance.

**Rationale:**
ISS III, R2 ISS III; high tumor burden. ASCT offers the best long-term control. No high-risk cytogenetics. DaraVCD induction suitable; according to ASCT Lenalidomide Maintenance Standard.

**===== run_3 =====**

**Tumor board decision:**
Continuation of DaraVCD induction up to 4 cycles; Subsequently, autologous stem cell transplantation (HD-Melphalan) if transplantable. Then consolidation (DaraVCD or VRD) and lenalidomide maintenance; supportive treatment of hypercalcemia/anemia as necessary.

**Rationale:**
Transplantable patient with active MM ISS III; no evidence of high-risk aberrations. ASCT remains standard for increasing remission and long-term control. DaraVCD is established induction; The aim is rapid remission and subsequent transplantation.

## ============================================================ gpt-oss-20b (agent) =============================================================

**===== run_1 =====**

**Tumor board decision:**
After Z1 DaraVCD, induction continues with DaraVRd (4 cycles), followed by autologous stem cell transplantation (melphalan 200mg/m2). Then lenalidomide maintenance 10 mg/month. For R2-ISSIII, consider tandem SZT if necessary.

**Rationale:**
Patient is high-risk ISSIII, no high-risk cytogenetics, no comorbidities permitting high-dose therapy. DaraVCD is not a standard induction; DaraVRd complies with guidelines. Autologous SCT offers best remission, lenalidomide maintenance improves survival. Hypercalcemia and osteolysis are controlled with bisphosphonates.

**===== run_2 =====**

**Tumor board decision:**
Continue induction with DaraVRd (4-6 cycles), then plan autologous stem cell transplantation (Melphalan 200mg/m2). Include osteoprotection, thrombo and infection prophylaxis as well as blood transfusions in cases of anemia.

**Rationale:**
Patient ISSIII, R2III, high-dose therapy eligible, no contraindications. DaraVRd is evidence-based induction in high-risk MM; ASCT after induction improves survival. Supportive measures address anemia, osteolysis and hypercalcemia.

**===== run_3 =====**

**Tumor board decision:**
Continue induction with DaraVRd (daratumumab+bortezomib+lenalidomide+dexamethasone) for 4 cycles, followed by autologous stem cell transplantation (melphalan200mg/m2). Plan for osteoprotection (bisphosphonate/denosumab) and anemia management.

**Rationale:**
Patient ISSIII, R2III, high-dose therapy eligible, anemia, osteolysis, hypercalcemia. Guidelines (Onkopedia2024) recommend DaraVRd + autologous for high-risk patients. SCT. DaraVCD is suboptimal; Switching to DaraVRd maximizes remission. Osteoprotection and anemia therapy are required as standard.

## ============================================================ gpt-oss-20b (plain) ============================================================

**===== run_1 =====**

**Tumor board decision:**
Continue DaraVCD up to at least 4-6 cycles followed by response assessment. If response is absent or unsatisfactory (after 4 cycles), switch to DRd (daratumumab+lenalidomide+dexamethasone) or carfilzomib-based; If necessary, consider autologous stem cell transplantation. No contraindications so far.

**Rationale:**
DaraVCD is standard first-line in transplant-ineligible MM. Patient has no high-risk cytogenetics and no contraindications. Continuation until response evaluation makes sense; in cases of progression or inadequate response, DRd or carfilzomib regimens are evidence-based. Hypercalcemia, anemia, and osteolysis are supported with bisphosphonates, ESAs, and calcium control.

**===== run_2 =====**

**Tumor board decision:**
Continue treatment with DaraVCD for 4 cycles, then evaluate ASCT (if transplant eligible). If ineligible: After 4 cycles of daratumumab maintenance.

**Rationale:**
Patient with ISSIII, R2-ISSIII, no high-risk cytogenetics, no contraindications to proteasome inhibitors or monoclonal antibodies. DaraVCD induces high remission; ASCT improves survival in transplant-eligible patients. If ineligible, daratumumab maintenance remains the default option.

**===== run_3 =====**

**Tumor board decision:**
Continue with DaraVCD until 8 cycles. If transplant eligible, then autologous stem cell transplantation. Then daratumumab maintenance. Treat hypercalcemia and anemia with bisphosphonates or erythropoietin.

**Rationale:**
Patient is undergoing initial treatment with DaraVCD, which is the current standard therapy for ISS-III-MM. 8 cycles is the recommended duration, followed by ASCT in suitable patients. Daratumumab maintenance reduces progression rate. Comorbidities (hypothyroidism, HT) have no contraindications to these regimens.

## ============================================================ gpt-oss-120b (agent) ============================================================

**===== run_1 =====**

**Tumor board decision:**
The induction with Dara-VCD is modified. Switch to Dara-VRd (daratumumab+bortezomib+lenalidomide+dexamethasone) for 4-6 cycles, followed by autologous stem cell transplantation, consolidation and lenalidomide maintenance.

**Rationale:**
Patient meets SLiM-CRAB, is transplant eligible and has no contraindication to lenalidomide. Dara-VRd is guideline standard for ISSIII/R2-ISSIII and achieves deeper remissions than VCD; Cyclophosphamide is not required.

**===== run_2 =====**

**Tumor board decision:**
The current induction with Dara-VCD should be changed in favor of guideline-compliant therapy with Dara-VRd (daratumumab+bortezomib+lenalidomide+dexamethasone); After 4-6 cycles, autologous stem cell transplantation is carried out and then lenalidomide maintenance.

**Rationale:**
The patient meets CRAB criteria, is suitable for high-dose therapy and has no contraindication to lenalidomide. Dara-VCD is not recommended in the current therapy algorithm, while Dara-VRd represents higher remission rates and standard induction.

**===== run_3 =====**

**Tumor board decision:**
Induction should be changed from Dara-VCD to a lenalidomide-based regimen, e.g. B. Dara-VRd (daratumumab+bortezomib+lenalidomide+Dex) for 4-6 cycles, then autologous SCT and lenalidomide maintenance.

**Rationale:**
Patient meets SLiM-CRAB, is suitable for transplant (no relevant contraindications) and has R-ISSIII. Guidelines recommend an anti-CD38 + PI + IMiD regimen for transplant-eligible patients, which achieves deeper remissions than Dara-VCD.

## ============================================================ gpt-oss-120b (plain) ============================================================

**===== run_1 =====**

**Tumor board decision:**
We recommend continuing the current induction with daratumumab VCD (at least 4 cycles), then carrying out an autologous stem cell transplant in transplant-eligible patients and continuing with lenalidomide maintenance (+/- daratumumab). Additionally bisphosphonates and supportive therapy.

**Rationale:**
The patient has ISS III MM without high-risk cytogenetics. The previous daratumumab VCD induction is standard first-line therapy and has not yet shown any progression. Continuation allows optimal tumor reduction before ASCT, which improves the prognosis. Supportive measures address anemia, osteolysis, and hypercalcemia.

**===== run_2 =====**

**Tumor board decision:**
Continue with ongoing induction (Dara-VCD) up to 4-6 cycles, then stem cell mobilization and autologous SCT (HD-Melphalan) as well as subsequent lenalidomide maintenance (optionally with daratumumab).

**Rationale:**
ISSIII/R2-ISSIII without high-risk cytogenetics, suitable for transplantation, good tolerability; Dara-VCD delivers high response rate. After achieving a CR/VGPR, ASCT is standard, maintenance improves progression-free survival.

**===== run_3 =====**

**Tumor board decision:**
Continue induction with daratumumab VCD for a total of 4 cycles, then assess response, collect hematopoietic stem cells and perform high-dose melphalan ASCT, followed by lenalidomide maintenance.

**Rationale:**
The patient has ISSIII but no high-risk cytogenetics and is transplant eligible. Daratumumab-VCD is a recognized first-line therapy standard. Response can be assessed after 4 cycles; ASCT improves overall survival and lenalidomide maintenance reduces the risk of recurrence.

## ============================================================ qwen3-32b (agent) ============================================================

**===== run_1 =====**

**Tumor board decision:**
Continuation of DaraVCD for 4-6 cycles, followed by autologous stem cell transplantation (Melphalan 200 mg/m2) with good response. Osteoprotection with bisphosphonate/denosumab, thromboprophylaxis. Subcutaneous CD38-Ab administration for hypothyroidism.

**Rationale:**
SLiM-CRAB criteria met, suitable for high-dose therapy. DaraVCD is acceptable as an induction. For ISS III and R2-ISS III, consolidation with SCT is indicated. Prophylaxis according to SOP. Hypothyroidism requires subcutaneous CD38-Ab administration.

**===== run_2 =====**

**Tumor board decision:**
Continue induction with DaraVCd (daratumumab s.c., bortezomib, cyclophosphamide, dexamethasone) for 4-6 cycles, followed by autologous SCT (melphalan 200mg/m2) if well tolerated. Maintenance with lenalidomide 10mg. Osteoprotection with bisphosphonate/denosumab, thromboprophylaxis and infection prophylaxis. For hypothyroidism: Daratumumab s.c. apply.

**Rationale:**
SLiM-CRAB criteria fulfilled (anemia, hypercalcemia, osteolysis), ISS III. DaraVCd is first line for renal failure. Subcutaneous daratumumab administration for hypothyroidism. Suitability for high-dose therapy is required. Prophylaxis according to SOP.

**===== run_3 =====**

**Tumor board decision:**
Continuation of induction therapy with DaraVCD for 3-4 additional cycles, followed by autologous stem cell transplantation (Melphalan 200mg/m2) if well tolerated, followed by lenalidomide maintenance therapy. Continue osteoprotection with bisphosphonates/desosumab, thromboembolism prophylaxis and infection prophylaxis.

**Rationale:**
The patient meets SLiM-CRAB criteria and is suitable for high-dose therapy. DaraVCD is approved in the first line. After 4-6 cycles, consolidation takes place using SCT. Lenalidomide maintenance optimizes prognosis. Prophylaxis is mandatory.

## ============================================================ qwen3-32b (plain) ============================================================

**===== run_1 =====**

**Tumor board decision:**
Continuation of the current DaraVCD therapy with clinical and laboratory chemical follow-up after 2-3 cycles. If symptoms (anemia, hypercalcemia) and clinical response are stable or improving, therapy is continued until a stable response is achieved. If there is no response after 3 cycles, switching to DVd (daratumumab, bortezomib, dexamethasone) is considered.

**Rationale:**
The DaraVCD combination is an established induction therapy for multiple myeloma. The lack of cytogenetic data and the absence of high-risk factors justify continuation. The secondary diagnoses of hypothyroidism and hypertension require careful dexamethasone dosing and monitoring. If there is no response after 3 cycles, it makes sense to switch therapy to DVd in order to avoid the development of resistance.

**===== run_2 =====**

**Tumor board decision:**
After induction with DaraVCD, it is recommended to pursue high-dose chemotherapy with autologous stem cell transplantation (ASCT), followed by lenalidomide maintenance, if transplantable. If transplant inability: Continuation of DaraVCD therapy with subsequent maintenance.

**Rationale:**
ISS III without high-risk cytogenetics justifies ASCT if induction is successful. DaraVCD is established induction. Secondary diagnoses (hypothyroidism, HT) allow dexamethasone use. Maintenance with lenalidomide is standard post-ASCT. If transplant is not possible, DaraVCD remains the next option.

**===== run_3 =====**

**Tumor board decision:**
Continuation of DaraVCD induction therapy followed by autologous stem cell transplantation (ASCT) with appropriate conditioning.

**Rationale:**

DaraVCD is an established induction standard. In ISS III without high-risk cytogenetics and good tolerance, ASCT is the next pillar after induction. Hypothyroidism and HTN require preoperative risk assessment.

## ============================================================ qwen3-next-80b (agent) ============================================================

**===== run_1 =====**

**Tumor board decision:**
Autologous stem cell transplantation (ASCT) with melphalan 200 mg/m2 as consolidation after D-VCD induction, followed by lenalidomide maintenance (10 mg daily).

**Rationale:**
SLiM-CRAB criteria met, suitable for high-dose therapy (no known contraindications). R2-ISS III High risk, but no evidence of high risk aberrations. ASCT is standard consolidation after induction. Lenalidomide maintenance according to guidelines for high-risk patients. Hypothyroidism and hypertension are controllable, not contraindications to ASCT.

**===== run_2 =====**

**Tumor board decision:**
Perform autologous stem cell transplantation (ASCT) with high-dose melphalan (200 mg/m2). Then start lenalidomide maintenance (10 mg daily).

**Rationale:**
Patient meets SLiM-CRAB criteria, no contraindications to high-dose therapy (no information on comorbidities that exclude ASCT). D-VCD induction completed, ASCT is standard consolidation in high dose eligible patients according to flowchart. ISS III/R2-ISS III requires aggressive therapy, ASCT improves progression-free survival.

**===== run_3 =====**

**Tumor board decision:**
Perform autologous stem cell transplantation (melphalan 200mg/m2) followed by lenalidomide maintenance therapy (10mg daily).

**Rationale:**
SLiM-CRAB criteria met, no contraindications for high-dose therapy (comorbidities can be treated). After induction with DaraVCD, ASCT is standard in high-dose eligible patients. Lenalidomide maintenance after ASCT according to guideline.

## =========================================================== qwen3-next-80b (plain) ===========================================================

**===== run_1 =====**

**Tumor board decision:**
Continue D-VCD induction therapy for 4-6 cycles. Subsequently ASCT if transplantation is suitable or maintenance therapy (e.g. lenalidomide).

**Rationale:**
D-VCD is established induction therapy for NDMM. ISS III and R2-ISS III require aggressive therapy. No high-risk cytogenetics, ASCT improves survival. Age unknown, therefore continue induction.

**===== run_2 =====**

**Tumor board decision:**
Continue D-VCD induction for 4-6 cycles, response assessment, then ASCT if transplant eligible or maintenance with lenalidomide.

**Rationale:**
D-VCD is standard induction in newly diagnosed MM. ISS III, but does not detect high-risk aberrations. After induction, transplantation status is evaluated.

**===== run_3 =====**

**Tumor board decision:**
Continue D-VCD therapy for 4-6 cycles, then re-evaluate for response and transplantability. If not transplantable: maintenance with lenalidomide.

**Rationale:**
D-VCD is standard induction for non-transplantable patients. Without response data, current therapy will continue. After 4-6 cycles, response is evaluated and ASCT or maintenance initiated if necessary.

# mm_case_13__ALL

################################
#### CASE: 13
################################

**============================================================ gpt-5-mini (agent) ============================================================**

**===== run_1 =====**

**Tumor board decision:**
1) Further procedure: bone marrow aspirate + biopsy for histological confirmation; re-testing of MYD88 and CXCR4; quantitative IgM, immunofixation serum/urine; BB, beta2-M, LDH, kidney/liver values; Neuropathy assessment and fundoscopy. 2) Therapy: asymptomatic -> watch and wait. For therapy indications -> fit: Rituximab + bendamustine (4-6 cycles). Not capable of chemo: Zanubrutinib. In case of hyperviscosity: plasmapheresis immediately.

**Rationale:**
Waldenström requires a mandatory bone marrow biopsy to confirm the diagnosis. Therapy only for disease-related symptoms. MYD88-WT reduces response to ibrutinib; Zanubrutinib is the BTKi option in MYD88-WT, R-bendamustine remains standard in the first line regardless of genotype. Plasmapheresis immediately in case of hyperviscosity.

**===== run_2 =====**

**Tumor board decision:**
Diagnostics: immediate bone marrow aspirate/biopsy with histology, flow/immunophenotype, CXCR4 mutation test, quantitative IgM + immunofixation, hemolysis parameters, beta2 microglobulin; Fundoscopy for visual impairment. Therapy: asymptomatic -> watch and wait. For clinically relevant AIHA, cytopenia, B symptoms, hyperviscosity or IgM>60 g/l -> systemic therapy: R-bendamustine 4 cycles (fit patient); in frail/comorbid patients. Rituximab+/-DRC. BTKi monotherapy not preferred due to MYD88 negativity; Check CXCR4 before BTKi; Plasmapheresis for hyperviscosity.

**Rationale:**

**Rationale: Confirmation of diagnosis requires contrast biopsy according to WHO. MYD88 negativity reduces ibrutinib effectiveness -> BTKi mono not first choice. R-bendamustine is the first-line standard regardless of genotype and is suitable for symptoms/cytopenias that require therapy. In case of hyperviscosity, immediate plasmapheresis.**

**===== run_3 =====**

**Tumor board decision:**
Diagnostics: Obligatory bone marrow biopsy with immunohistology/immunophenotyping + CXCR4 test; Basic laboratory including quant. IgM/immunofixation, hemolysis parameters (Direct Coombs), BB, beta2-M, LDH; 24-hour urine; Fundoscopy for hyperviscosity. Therapy: Without clinical symptoms -> Watch and wait. If AIHA is relevant, cytopenia or hyperviscosity -> requires therapy: in fit patients R-bendamustine (4 cycles); for comorbidities/elderly patients, rituximab (+/- acute steroid therapy). MYD88 negative: avoid ibrutinib monotherapy; Consider zanubrutinib if not suitable for CIT.

**Rationale:**
The diagnosis must be confirmed histologically (CM infiltrate). Therapy only if there are signs of illness; AIHA or cytopenias are indications for therapy. MYD88 negativity reduces effectiveness of ibrutinib mono, therefore chemoimmunological (BR) preferred in fit patients; Rituximab as a gentle alternative or acute steroid therapy for severe hemolysis.

**============================================================ gpt-5-mini (plain) ============================================================**

**===== run_1 =====**

**Tumor board decision:**
Complete diagnostics: bone marrow biopsy with morphology/immunophenotyping/CYTOLOGY, serum IgM, viscosity, cryoglobulins, complete hemolysis profile, hepatitis/HIV. If Waldenström is confirmed and symptomatic disease (AIHA/anemia): immediate steroids to control hemolysis + rituximab; with significant tumor burden, preference is given to rituximab+bendamustine. BTK inhibitors primarily not recommended (MYD88 negative).

**Rationale:**
Diagnosis must be confirmed histologically/serologically. AIHA requires rapid hemolysis therapy (steroid + rituximab). MYD88 negativity reduces effectiveness of BTK inhibitors; therefore evidence-based choice: rituximab-based chemoimmunotherapy (e.g. bendamustine+rituximab) if systemically relevant.

**===== run_2 =====**

**Tumor board decision:**
Initial treatment of hemolysis: Prednisolone 1 mg/kg (transfusions if necessary; plasmapheresis if hyperviscosity). Thereafter, immediate first-line systemic therapy with a rituximab-based regimen: preferably bendamustine+rituximab (BR) for 6 cycles. BTK inhibitor (e.g. ibrutinib) not recommended due to MYD88 negativity. Additional workup: complete staging, bone marrow biopsy, CXCR4 mutation test before therapy.

**Rationale:**
AIHA requires acute immunosuppression; Rituximab addresses both WM and AIHA. BR is established and effective as first-line therapy. MYD88 wild type reduces response rate to BTK inhibitors, therefore not first choice. Complete staging and additional molecular diagnostics are advisable before therapy.

**===== run_3 =====**

**Tumor board decision:**
Further procedure: 1) Complete diagnostics: bone marrow biopsy with morphology/flow, serum IgM, viscosity, cryoglobulins and MYD88/CXCR4 test (if not already). 2) Treat AIHA acutely: prednisone ~1 mg/kg +/- IVIG/transfusion; Plasmapheresis for hyperviscosity. 3) First-line therapy WM (MYD88-neg): rituximab-bendamustine (BR) or bortezomib-based combination; Avoid BTK inhibitors.

**Rationale:**

**Rationale: AIHA requires immediate therapy; Histological confirmation of the suspected Waldenström diagnosis is necessary. MYD88 negativity reduces response to classic BTK inhibitors, therefore chemoimmunotherapeutic regimens (BR) or bortezomib combinations are preferred. Rituximab also helps with AIHA; Plasmapheresis for hyperviscosity.**

## ============================================================ gpt-5-nano (agent) ============================================================

**===== run_1 =====**

**Tumor board decision:**
Confirm the diagnosis of WM: bone marrow biopsy with LPL infiltration; IgM, MYD88 (and CXCR4), staging CT, check hyperviscosity. Confirmed WM without hyperviscosity: prefer BTKi first line; Zanubrutinib monotherapy in MYD88wt/CXCR4wt; Ibrutinib +/- rituximab for CXCR4mut/MYD88mut; Chemoimmunotherapy as an alternative if CIT is suitable; Pay attention to IgM flare; Plasmapheresis only in cases of hyperviscosity.

**Rationale:**
MYD88neg reduces ibrutinib effectiveness; ASPEN demonstrates zanubrutinib in MYD88wt with good efficacy and better safety profile. If there is no hyperviscosity, BTKi-first makes sense; CXCR4 status helps BTKi choice. Diagnostics are essential before therapy.

**===== run_2 =====**

**Tumor board decision:**
Confirmation of the diagnosis (bone marrow biopsy with LPL infiltration, IgM gammopathy) and determination of MYD88/CXCR4. For MYD88 wild-type, prefer zanubrutinib monotherapy as first-line (BTKi). Plasmapheresis for hyperviscosity before therapy; Rituximab/chemo only in cases of BTKi intolerance or AIHA indication. Check studies.

**Rationale:**
MYD88wt reduces ibrutinib efficacy; ASPEN data show zanubrutinib better efficacy/tolerability in WM, particularly MYD88wt. No pretreatment; Hyperviscosity unclear, hence BTKi first. Consideration of AIHA when choosing therapy; Check CXCR4 status for BTKi optimization.

**===== run_3 =====**

**Tumor board decision:**
Confirm WM diagnosis: bone marrow biopsy with IgM, MYD88 L265P, CXCR4; Staging. Hyperviscosity: plasmapheresis. First line if status is good: BR (rituximab + bendamustine) 4-6 cycles; Alternative: DRC. Rituximab monotherapy for comorbidities. BTK inhibitors only if unsuitable for

chemoimmunotherapy or patient preference; Take mutations into account.

**Rationale:**
BR achieves rapid, deep remissions regardless of MYD88/CXCR4 status; MYD88wt decreases BTKi response; Hyperviscosity/AIHA prefer rituximab approaches; BTKi makes sense if unsuitable or patient preference; Consider CXCR4/MYD88 status.

## ============================================================ gpt-5-nano (plain) ============================================================

**===== run_1 =====**

**Tumor board decision:**
BR (bendamustine + rituximab) as first-line therapy for Waldenström-MG with IgM paraprotein and AIHA; Treatment plan: 6 cycles q28d, IgM/Hb monitoring; Plasmapheresis for hyperviscosity/IgM flare.

**Rationale:**
WM standard first line; MYD88 wild type reduces BTKi success; Rituximab helps AIHA; IgM flare risk exists; Plasmapheresis if necessary to control hyperviscosity.

**===== run_2 =====**

**Tumor board decision:**
BR (bendamustine + rituximab) as first-line therapy for WM with IgM gammopathy, MYD88 negative, AIHA. If BR is contraindicated, DRC as an alternative.

**Rationale:**
MYD88-negative reduces BTKi success; BR delivers good RR/PFS; Rituximab helps AIHA; no risk of hyperviscosity; no previous therapies -> first-line option.

**===== run_3 =====**

**Tumor board decision:**
BR (bendamustine 90 mg/m2 day 1+2; rituximab 375 mg/m2) as first-line treatment of Waldenström macroglobulinemia with AIHA; 6 cycles q28d; Plasmapheresis only for hyperviscosity; Monitor Hb, IgM and viscosity closely.

**Rationale:**
MYD88 negative; BTK inhibitors less robust. BR offers high response rate and PFS; Rituximab addresses AIHA; no previous therapies; established first-line therapy. In case of intolerance, alternative: DRC or BTKi.

## ============================================================ gpt-oss-20b (agent) =============================================================

**===== run_1 =====**

**Tumor board decision:**
Watch & wait until symptoms appear. In case of symptomatic progression: fit -> R-bendamustine or DRC; unfit -> BTK inhibitor with rituximab (ibrutinib+R or zanubrutinib+R). In case of hyperviscosity, plasmapheresis is carried out immediately, followed by anti-lymphoma therapy.

**Rationale:**
MYD88-wt WM has low activity in BTKi monotherapy; Ibrutinib+R and Zanubrutinib+R show MRR ~70%. Chemoimmunotherapy works regardless of genotype. Watch and wait is standard for asymptomatic disease.

**===== run_2 =====**

**Tumor board decision:**
R-bendamustine (4-6 cycles) or alternatively DRC as first-line therapy for MYD88-negative Waldenström with AIHA. Chemo-immunotherapy works independently of MYD88 and provides rapid symptom relief; BTKi are less effective in MYD88-negative.

**Rationale:**
The patient is asymptomatic for lymph nodes but has AIHA, which requires therapy. In MYD88-negative status, BTK inhibitors are less effective (MRR 0% with ibrutinib monotherapy). Chemo-immunotherapies (R-bendamustine, DRC) show stable remissions in MYD88-negative WM and are the evidence-based first line.

**===== run_3 =====**

**Tumor board decision:**
Start R-bendamustine (4-6 cycles) as first-line therapy. In the case of IgM flare (>=40g/l), plasmapheresis may be necessary before rituximab or without rituximab. Plan for PJP prophylaxis (cotrimoxazole). BTK inhibitor (Zanubrutinib) only if intolerance to chemo-immunotherapy.

**Rationale:**
MYD88 negative means BTK inhibitors are less effective; Chemo-immunotherapy (R-bendamustine) is independent of genotype and has the best evidence. Rituximab simultaneously treats AIHA. IgM flare and hyperviscosity are addressed with plasmapheresis or prophylaxis. PJP prophylaxis is standard for rituximab therapy.

## ============================================================ gpt-oss-20b (plain) ============================================================

**===== run_1 =====**

**Tumor board decision:**
Start rituximab-bendamustine (R-B) therapy as first-line therapy for symptomatic Waldenström (IgM gammopathy, MYD88-negative, AIHA).

**Rationale:**
R-B is the evidence-based first-line therapy for MYD88-negative WM, offers good remission rates and controls AIHA. ibrutinib is less effective in MYD88-negative patients; therefore R-B is recommended.

**===== run_2 =====**

**Tumor board decision:**
Start first-line therapy with bendamustine (90mg/m2 IV days1-2) plus rituximab (375mg/m2 IV days1,8,15,22) q28days for 6 cycles. Ibrutinib 420mg PO daily is an alternative if oral therapy is preferred.

**Rationale:**
Patient is close to therapy WM, MYD88 negative, with AIHA. Bendamustine-rituximab acts independently of MYD88 and can control hemolysis. Ibrutinib has lower response rates in MYD88-negative WM; Oral approach is recommended only based on patient preference.

**===== run_3 =====**

**Tumor board decision:**
When diagnosed with Waldenström macroglobulinemia with IgM gammopathy, MYD88 negativity and AIHA, first-line therapy with venetoclax in combination with rituximab is recommended. If there is no organic involvement, rituximab monotherapy can be considered as an alternative.

**Rationale:**
Venetoclax+Rituximab is the evidence-based first-line option in MYD88-negative WM and reduces both IgM levels and AIHA. Rituximab monotherapy is acceptable in low tumor burden but less effective in MYD88 negativity.

## ============================================================ gpt-oss-120b (agent) ============================================================

**===== run_1 =====**

**Tumor board decision:**
Complete diagnostics including bone marrow biopsy, immunophenotype, CXCR4 mutation analysis, CT abdomen/thorax, laboratory (Hb, LDH, beta2 microglobulin, IgM quantification, Coombs test). If LPL and symptomatic AIHA (or cryoglobulinemia) are confirmed, immediate therapy with R-bendamustine 4-6 cycles (R-B) + PJP prophylaxis; in case of hyperviscosity, plasmapheresis before therapy. If frailty is significant, rituximab monotherapy may be necessary.

**Rationale:**
Patient has symptomatic anemia (AIHA) -> indication for therapy according to TI-1. MYD88 negative, BTK inhibitors are less effective; Chemo-immunotherapy works regardless of genotype and has the highest response rate. R-bendamustine is standard first-line for fit patients; Rituximab monotherapy as an alternative in cases of intolerance.

**===== run_2 =====**

**Tumor board decision:**

Complete diagnostics (BM biopsy, immunophenotype, CXCR4 mutation status, CT, laboratory including IgM quant, AIHA assessment). For confirmed WM with symptomatic AIHA, immediate treatment: Rituximab monotherapy (375 mg/m2 IV weekly x 4) plus corticosteroids. If response is inadequate, consider BTK inhibitor zanubrutinib (300 mg qd) or chemoimmunotherapy (R-bendamustine).

**Rationale:**
MYD88 negativity requires histological confirmation; no hyperviscosity, but AIHA is a clear indication for therapy. Rituximab treats both lymphoma and AIHA and is effective in MYD88-wt. BTK inhibitors show moderate activity in MYD88-wt (zanubrutinib). Chemo-immunotherapy remains a reserve option.

**===== run_3 =====**

**Tumor board decision:**
Therapy: Start of chemoimmunotherapy with rituximab+bendamustine (4-6 cycles) including PJP prophylaxis; if IgM>40g/l first cycle without rituximab or previous plasmapheresis. MYD88/CXCR4 genotyping before further treatment, close monitoring of IgM levels and anemia.

**Rationale:**
AIHA is a hematopoietic insufficiency and constitutes an indication for therapy. In the case of MYD88 negativity, BTK inhibitors have little effect, while R-bendamustine achieves high response rates regardless of genotype. Rituximab monotherapy is less effective; Plasmapheresis only in cases of hyperviscosity.

## =============================================================== gpt-oss-120b (plain) ===============================================================

**===== run_1 =====**

**Tumor board decision:**
Starting therapy for AIHA. Rituximab monotherapy (375 mg/m2 weekly x4) is recommended as first line. If the response is inadequate, a combination with bendamustine or cyclophosphamide may be considered. Concomitant initial corticosteroids to control AIHA.

**Rationale:**
The patient has symptomatic IgM gammopathy with autoimmune hemolysis, which indicates treatment. Rituximab also works in MYD88-negative Waldenström and has a favorable side effect profile. Combination therapies are intended for refractory cases.

**===== run_2 =====**

**Tumor board decision:**
Start of first-line therapy with rituximab+bendamustine (R-B) and simultaneous corticosteroid therapy to control AIHA. If there is no clinical improvement or intolerance, consider alternative rituximab-based regimens (R-CD) or BTK inhibitor (zanubrutinib).

**Rationale:**
The patient has symptomatic IgM gammopathy with AIHA, which represents an indication for therapy. MYD88 negativity reduces the expected efficacy of BTK inhibitors, therefore a rituximab-based regimen is preferred. R-B provides high response rate and rapid control of anemia.

**===== run_3 =====**

**Tumor board decision:**
Since the patient is symptomatic (AIHA) and has a MYD88-negative Waldenström macroglobulinemia picture, we recommend immediate therapy. Start with rituximab-bendamustine (R-B) combined with short prednisone therapy to control AIHA. BTK inhibitors (e.g. ibrutinib) are less suitable due to MYD88 negativity.

**Rationale:**
No prior therapy, but AIHA requires intervention. MYD88 negativity reduces efficacy of BTK inhibitors, while rituximab-based combinations effectively address both IgM production and hemolytic anemia. The regimen is established, well tolerated and provides rapid clinical improvement.

## ================================================================ qwen3-32b (agent) ================================================================

**===== run_1 =====**

**Tumor board decision:**

Recommendation: Zanubrutinib as first choice for MYD88-negative Waldenström disease. Alternatively, venetoclax or ibrutinib+rituximab upon individual consideration.

**Rationale:**
Zanubrutinib has best evidence in phase III trials in MYD88-negative cases. BTK inhibitors modulate the microenvironment and are established in real-world data. Venetoclax and ibrutinib+rituximab are alternative backup in case of contraindications.

**===== run_2 =====**

**Tumor board decision:**
Recommendation: Zanubrutinib monotherapy as first line. In case of BCL2 overexpression (confirm by biomarker analysis if necessary), alternatively consider venetoclax-based combination therapy.

**Rationale:**
BTK inhibitors dominate therapy for MYD88-negative WM (ASH 2023/2025). Zanubrutinib has a high level of evidence. Venetoclax could be useful in BCL2 overexpression, but requires biomarker confirmation. KMP therapy has already been carried out, no LN restriction exists.

**===== run_3 =====**

**Tumor board decision:**
Recommendation: Zanubrutinib as first line in MYD88-negative Waldenström disease. Alternatively B-DRC in case of resistance to therapy.

**Rationale:**
Zanubrutinib shows high efficacy in MYD88 wild-type WM (PMID:37478390). B-DRC is an established salvage option. AIHA requires close hemato-oncological monitoring.

## ============================================================ qwen3-32b (plain) ============================================================

**===== run_1 =====**

**Tumor board decision:**
Recommendation: Diagnosis confirmation by bone marrow puncture and histology. For confirmed Waldenström's disease with AIHA: Rituximab-based therapy in combination with corticosteroids for the treatment of AIHA. Subsequent observation of clinical response.

**Rationale:**
The suspected diagnosis of M. Waldenström requires histological confirmation. MYD88 negativity and AIHA indicate active disease. Rituximab is established in MYD88-negative cases and can accompany AIHA. Corticosteroids are indicated for hemolytic anemia.

**===== run_2 =====**

**Tumor board decision:**
Initiate combination therapy with ibrutinib and corticosteroids for the treatment of v. a. M. Waldenström with IgM gammopathy and concomitant AIHA.

**Rationale:**
Ibrutinib is an established treatment option for MYD88-negative Waldenström disease and also addresses B cell activation in AIHA. Corticosteroid treatment is indicated for immediate stabilization of hemolytic anemia.

**===== run_3 =====**

**Tumor board decision:**
Initiate rituximab-based therapy for the treatment of MYD88-negative Waldenström macroglobulinaemia. Rituximab is also suitable for accompanying AIHA. A combination with corticosteroids is useful in severe AIHA.

**Rationale:**
MYD88 negativity excludes BTK inhibitors such as ibrutinib as first line. Rituximab is established at Waldenström and AIHA. Corticosteroid combinations are effective in hemolytic anemia. No contraindications or previous illnesses limit this therapy.

## ============================================================ qwen3-next-80b (agent) ============================================================

===== run_1 =====

**Tumor board decision:**
First-line therapy with rituximab + cyclophosphamide/dexamethasone (DRC) is recommended. BTK inhibitors contraindicated due to MYD88 negativity. Dexamethasone in DRC supports the treatment of AIHA.

**Rationale:**
MYD88 wild-type WM shows lower response rates to BTK inhibitors (ASPEN study). DRC combines rituximab (effective in AIHA) with steroids that directly address AIHA. Bendamustine rituximab does not contain steroids and is less suitable for AIHA comorbidity.

===== run_2 =====

**Tumor board decision:**
First-line therapy with rituximab plus dexamethasone (R-Dex) is recommended. BTK inhibitors contraindicated due to MYD88 wild-type status. R-Dex combines effective immunotherapy with steroids to treat AIHA and reduce toxicity.

**Rationale:**
MYD88-negative WM shows reduced response rates to BTK inhibitors (ASPEN study). Rituximab-based chemoimmunotherapy is standard. R-Dex provides steroids to control AIHA and is safer than more intensive regimens such as R-CVP when ECOG/age is unclear.

===== run_3 =====

**Tumor board decision:**
Rituximab-based chemoimmunotherapy (R-CVP) as the first choice because MYD88-negative WM has a poor response to BTK inhibitors and R-CVP is effective with concurrent AIHA.

**Rationale:**
MYD88-negative WM shows lower response rates to BTK inhibitors in ASPEN study. Chemoimmunotherapy (R-CVP) is the first choice. AIHA is addressed by rituximab and steroids in R-CVP. No known contraindications.

## ============================================================ qwen3-next-80b (plain) ============================================================

===== run_1 =====

**Tumor board decision:**
Confirmation of diagnosis by bone marrow biopsy with immunohistochemistry, flow cytometry and genetic analysis (MYD88, CXCR4) is required. No therapy initiation without a confirmed diagnosis.

**Rationale:**
MYD88 negativity complicates Waldenström diagnosis. Without definitive confirmation of the diagnosis (bone marrow, IHC, flow), therapy is not indicated. AIHA must be clarified as secondary or primary.

===== run_2 =====

**Tumor board decision:**
Confirmation of diagnosis by bone marrow biopsy with immunohistochemistry, flow cytometry and IgM quantification. If LPL/Waldenström macroglobulinemia is confirmed, initiate therapy; if not confirmed, consider alternative diagnoses.

**Rationale:**
MYD88 negative and unclear diagnosis require histological confirmation. Without confirmation of the diagnosis, no therapy can be initiated. AIHA must be clarified through a differential diagnosis.

===== run_3 =====

**Tumor board decision:**
Diagnosis confirmed. Initiate rituximab monotherapy (375 mg/m2 weekly for 4-6 weeks).

**Rationale:**
AIHA as a symptomatic complication requires therapy. Rituximab is effective in Waldenström and AIHA. MYD88 negativity does not influence rituximab response. No contraindications.

# mm_case_14__ALL

################################
#### CASE: 14
################################

## ================================================================ gpt-5-mini (agent) ================================================================

**===== run_1 =====**

**Tumor board decision:**
Image-guided punch biopsy of the BWK 2 lesion for histological confirmation (if technically/risk-wise justifiable). If a plasmacytoid/POEMS lesion is confirmed: primary local therapy = fractionated radiotherapy on BWK2. If there is impending instability/neurological impairment, additional surgical stabilization/vertebroplasty. No immediate system change if systemic remission continues and VEGF is stable; systemic escalation only in the case of histologically proven or multifocal progression.

**Rationale:**
Isolated sclerotic progression with otherwise systemic remission and stable VEGF justifies primarily local approach. Biopsy clarifies differential diagnosis and avoids unnecessary system escalation. Radiotherapy is established for solitary bone findings; Surgery/vertebroplasty if there is a risk of fracture/neurology. Systemic therapy only if generalization is assured.

**===== run_2 =====**

**Tumor board decision:**
Recommendation: CT-guided biopsy of the BWK-2 lesion for histological confirmation. If localized progression is confirmed: local radiotherapy (e.g. 30-40 Gy) +/- surgical stabilization for spinal instability/neurological risk. Continue systemic therapy unchanged; Switch only in the case of multifocal/systemic progression or aggressive clonal findings.

**Rationale:**

**Rationale: Isolated skeletal progression with stable VEGF and other remission suggests local treatment. Literature and conference recommendations favor biopsy for clarification; If there are local findings, radiotherapy +/- surgery and continuation of systemic therapy. Systemic escalation only in the case of generalized progression or detectable clonal aggressiveness.**

**===== run_3 =====**

**Tumor board decision:**
Image-guided biopsy of BWK-2 for histological confirmation. Confirmation of a solitary lesion: local radiotherapy of the vertebral metastasis +/- surgical stabilization in the case of instability/neurological findings. No immediate change in systemic therapy; close image and VEGF controls. In the case of histologically/clinically confirmed systemic progression: escalate systemic therapy (e.g. anti-CD38 combination).

**Rationale:**
Isolated local progression despite systemic PR and stable VEGF suggests local relapse/plasmacytoma. Evidence and expert consensus favor biopsy and local therapy (RT +/- OP) first rather than immediate system escalation to avoid unnecessary toxicity; Systemic therapy only if systemic progression is confirmed.

## ================================================================ gpt-5-mini (plain) ================================================================

**===== run_1 =====**

**Tumor board decision:**
Recommendation: Local treatment of isolated BWK2 progression - primary definitive radiotherapy (30-36 Gy in 10-12 fractions). If there is diagnostic uncertainty, CT-targeted biopsy before RT. In the case of neurological deficits/spinal instability: immediate neurosurgical evaluation and, if necessary, stabilization/decompression before RT. No change in systemic therapy; close VEGF and imaging monitoring.

**Rationale:**

**Rationale: In POEMS, an isolated plasmacytic bone lesion usually leads to local symptoms; Radiotherapy provides high local control. Systemically there is still remission and stable VEGF, therefore no escalation of systemic therapy currently. Biopsy clarifies differential diagnoses; Surgery for instability/compression.**

**===== run_2 =====**

**Tumor board decision:**
Local therapy for isolated BWK2 progression: percutaneous external radiation therapy to BWK2 (e.g. 30-36 Gy in 10-15 fractions). Orthopedic assessment regarding stability/SSG; In the case of instability or neurological symptoms, additional procedures (stabilization/vertebroplasty). No systemic change in therapy, close follow-up monitoring (PET/CT, VEGF).

**Rationale:**
In POEMS with isolated bony progression in otherwise systemic remission and stable VEGF, local treatment is effective. Radiation therapy effectively controls solitary lesions. Systemic therapy only for multifocal/systemic progression. No contraindications documented.

**===== run_3 =====**

**Tumor board decision:**
In the case of isolated BWK2 progression, we primarily recommend an image-guided biopsy for histological confirmation. If confirmed: local RT to BWK2 (e.g. 30-40 Gy in 10-20 fractions). In case of neurological compression/instability: rapid neurosurgical decompression +/- stabilization, then RT. Do not change systemic therapy, close VEGF/imaging controls.

**Rationale:**
POEMS with isolated bone lesion responds well to local therapy. Systemically there is still remission and stable VEGF, therefore no immediate change of systemic therapy. Biopsy confirms diagnosis; RT locally controls growth and symptoms. Surgery for neurological threats.

## ================================================================ gpt-5-nano (agent) ================================================================

**===== run_1 =====**

**Tumor board decision:**
Local radiotherapy BWK2 for focal progression (e.g. 30 Gy in 10 fractions); If necessary, biopsy for verification. Continue systemic POEMS therapy in remission; Monitor VEGF status closely. If further progression or VEGF increase, change the therapy class instead of repeat VCD (e.g. DaraRd or IsaRd).

**Rationale:**
POEMS progression is often local; RT maintains systemic remission, reduces local contact and neuropathy risk. If necessary, rapid system change to a new class (without VCD) to avoid resistance and continue VEGF monitoring.

**===== run_2 =====**

**Tumor board decision:**
Local BWK2 progression in POEMS syndrome in systemic remission: radiotherapy BWK2 for local control; if necessary, biopsy to verify the lesion; Continuation of systemic remission treatment (VCD) if tolerated, alternatively change of regimen only in the event of further/systemic progression. Close monitoring (VEGF, imaging).

**Rationale:**
Focus on local control for isolated lesion with systemic remission is evidence-based; Radiotherapy effective for skeletal lesions; Maintain systemic therapy if possible to stabilize VEGF reduction and remission; Biopsy ensures diagnostic certainty; Monitoring critical.

**===== run_3 =====**

**Tumor board decision:**
Local radiotherapy BWK2 including biopsy to confirm the lesion; continued monitoring of systemic POEMS disease. No further systemic therapy for isolated local progression. If systemic progression is evident (PET/VEGF increase), consider rapid systemic MM therapy.

**Rationale:**
POEMS-specific data is missing. For focal lesions, RT is useful and provides local control; Biopsy may clarify suspected plasmacytoma. Systemic therapy only when progression is documented in order to avoid toxic exposure. VEGF change alone is not sufficient evidence of progression; PET/CT progression

is crucial.

## ============================================================ gpt-5-nano (plain) ============================================================

**===== run_1 =====**

**Tumor board decision:**
Local radiotherapy BWK2 (EBRT approx. 30 Gy in 10 Fr) to control isolated progression; Continue/continue monitoring systemic POEMS therapy. If systemic progression occurs again, switch to lenalidomide/Dex or bortezomib-based therapy; Consider ASCT based on age/comorbidities.

**Rationale:**

**Rationale: Isolated skeletal progression with otherwise stable VEGF and remission; local RT is standard for local control, pain/fracture protection; reduces need for immediate systemic therapy. Further action in the event of progression clearly defined.**

**===== run_2 =====**

**Tumor board decision:**
Local radiation therapy BWK2 (e.g. SBRT/RT) for the treatment of isolated progression; Subsequently, continuation of systemic POEMS therapy with a neuropathy-sparing regimen: daratumumab + lenalidomide + dexamethasone (DRd) to stabilize remission and VEGF control.

**Rationale:**
Isolated local progression with otherwise stable/systemic remission; RT provides fast local control. To avoid neurotoxic repeat therapies, switch to neuroprotective DRd; DRd has potential for increasing remission and reducing VEGF, preserving function.

**===== run_3 =====**

**Tumor board decision:**
Local radiotherapy (EBRT) BWK2 to control isolated progression; Dose 30 Gy/10 Fr (if necessary 40 Gy/20 Fr). Continue systemic therapy (VCD) with close monitoring.

**Rationale:**
Isolated local progression with otherwise stable remission and VEGF level. RT provides effective local control and symptom reduction while continuing systemic therapy to maintain remission; no immediate system change necessary, monitor further progression.

## ============================================================ gpt-oss-20b (agent) ============================================================

**===== run_1 =====**

**Tumor board decision:**
Start quadruplet Daratumumab+VRd (bortezomib, lenalidomide, dexamethasone) for 8-12 cycles, local therapy (e.g. stereotactic radiation) for isolated BWK 2 progression, VEGF monitoring.

**Rationale:**
Literature (3 PubMed articles) shows anti-CD38 therapy in combination with VRd PFS improvement in isolated progression despite systemic remission. Conference contributions support quadruplet therapies (D-K-R-D) for prolonging remission. Patient has stable VEGF, no contraindications, therefore suitable.

**===== run_2 =====**

**Tumor board decision:**
For isolated BWK-2 progression in systemic remission with stable VEGF, we recommend the introduction of an anti-CD38 antibody. Specifically: Daratumumab in combination with VCD (Dara-VCD) as an initial relapse regimen. If there is no anti-CD38 response, alternatively belantamab-mafodotin + bortezomib/dexamethasone.

**Rationale:**
Phase III Studies Show PFS Improvement with Dara-VRd/VCD; Conference papers demonstrate the effectiveness of quadruplet regimens and iberdomide triplets in relapse. VEGF stability speaks for systemic therapy; local radiotherapy can also be considered.

**===== run_3 =====**

**Tumor board decision:**
We recommend switching from VCD to D-VCD (daratumumab + bortezomib, cyclophosphamide, dexamethasone) as the next systemic therapy and adding local radiotherapy (~20Gy) to the isolated BWK2 lesion. If the control is insufficient, D-VRd can then be considered.

**Rationale:**
The POEMS patient is in partial remission with stable VEGF but isolated progression. Literature (3 PubMed articles) shows that the addition of daratumumab to VCD significantly increases PFS. Clinical experience from similar cases supports a switch to D-VCD. Local RT is indicated by default in focused progression.

## ============================================================ gpt-oss-20b (plain) ============================================================

**===== run_1 =====**

**Tumor board decision:**
Local radiotherapy (30-36Gy in 10-12Fr) on BWK2, followed by follow-up; no change in systemic therapy.

**Rationale:**
Systemic remission is maintained and VEGF is stable. The progression is locally limited. In POEMS, local RT is the standard treatment for focused lesions. VCD has already been used; Further systemic therapy is not necessary without progression.

**===== run_2 =====**

**Tumor board decision:**
Local radiotherapy (30Gy in 10Fr.) on BWK2 to control isolated progression. Systemic therapy remains paused, VEGF remains stable.

**Rationale:**
In POEMS with systemic remission and stable VEGF, local radiotherapy is the standard option for individual bone lesions. Reinduction of systemic therapy is not necessary if there is no progression.

**===== run_3 =====**

**Tumor board decision:**
Local radiotherapy (30Gy in 10Fr.) on BWK2, continued observation of systemic remission.

**Rationale:**
Isolated bone progression with stable VEGF and systemic remission; POEMS lesions respond well to local RT without risking further systemic therapy. Observation remains useful because VEGF does not increase.

## ============================================================ gpt-oss-120b (agent) ============================================================

**===== run_1 =====**

**Tumor board decision:**
In the case of isolated local progression of BWK2 with other systemic remission of the POEMS syndrome, we recommend local radiotherapy (e.g. 24Gy in 12 fractions) and continuation of the previous systemic therapy (monitoring/continuation of VCD) without switching to new agents.

**Rationale:**
Systemic remission (VEGF stability, PR) does not justify a change in systemic therapy. In POEMS patients, local radiotherapy is recommended as standard for single bone progression because it provides rapid local control and avoids systemic side effects. BCMA bispecifics or newer combinations are missing for POEMS and are only indicated in the event of systemic recurrence.

**===== run_2 =====**

**Tumor board decision:**
In the event of systemic remission and isolated local progression of BWK2, local radiotherapy (e.g. 24Gy in 12 fractions) should be carried out; systemic therapy is paused and patients continue to be monitored closely until systemic progression occurs again.

**Rationale:**

VCD therapy achieved partial remission with >50% decrease in VEGF; only a single PET-positive lesion, which, according to POEMS guidelines, recommends local radiotherapy for isolated progression. Systemic intensification (e.g. BCMA bispecifics) lacks evidence in this context and carries unnecessary toxicity.

**===== run_3 =====**

**Tumor board decision:**
Carry out local radiotherapy (30Gy in 15 Fr.) at BWK2, maintain systemic therapy (VCD paused) and close clinical and imaging follow-up. If there is no control or new systemic progression, consider systemic escalation (e.g. PI-IMiD combination or DKd).

**Rationale:**
The patient is in partial remission, VEGF is stable, and only a single vertebral lesion is progressing. Literature and congress data show that local RT (~30Gy) has a rapid symptomatic effect in isolated progression without destabilizing the systemic clinical picture. Systemic intensification is only recommended if there is no response or further progression; There are no contraindications to RT.

## ============================================================== gpt-oss-120b (plain) ==============================================================

**===== run_1 =====**

**Tumor board decision:**
In the case of isolated local progression of BWK2 despite systemic remission and stable VEGF, local radiotherapy (e.g. 20-30Gy in 10-15 fractions) is recommended. Systemic therapy is continued without change; A new systemic step (e.g. lenalidomide-based) is only considered if the disease progresses further.

**Rationale:**
The lesion alone corresponds to solitary plasmacytoma progression, which can be effectively controlled by high-dose local radiotherapy while systemic remission is maintained. Further systemic intensification is not indicated in the absence of additional progression.

**===== run_2 =====**

**Tumor board decision:**
Perform local radiotherapy (e.g. 24Gy in 12 fractions) for progressive BWK2; Pause systemic therapy or continue with lenalidomide-dexamethasone as maintenance/maintenance therapy, provided there are no contraindications.

**Rationale:**
Isolated bone progression with other systemic remission and stable VEGF requires local control; Radiation therapy prevents compression/fracture. Systemic therapy remains effective, a maintenance regimen (Lenalidomide-Dex) reduces the risk of recurrence, while a new bortezomib cycle is not necessary.

**===== run_3 =====**

**Tumor board decision:**
In the case of isolated local progression of BWK2 despite systemic remission, local radiotherapy (30-36Gy) is recommended; The current VCD induction will be maintained and systemic therapy will be paused for the time being.

**Rationale:**
Systemic remission and stable VEGF levels indicate effective control. In POEMS syndrome, local radiotherapy is standard for single bone lesions and avoids unnecessary systemic side effects.

## ================================================================ qwen3-32b (agent) ================================================================

**===== run_1 =====**

**Tumor board decision:**
Addition of daratumumab (CD38-targeted) to ongoing VCD therapy for local control of BWK 2 progression

**Rationale:**
The isolated local progression with stable systemic remission and constant VEGF value argues for local therapy supplementation. Literature and conference papers recommend CD38-targeted antibodies

(e.g. daratumumab) to improve local control. VCD is already effective (PR achieved), therefore there will be no regime change. Radiation therapy remains a secondary option in cases of resistance to therapy.

**===== run_2 =====**

**Tumor board decision:**
Recommendation: Local irradiation of the progressive BWK 2 lesion with stable systemic remission. No systemic therapy changes required.

**Rationale:**
The isolated local progression with stable VEGF reduction and PR suggests local therapy (radiotherapy). Systemic therapy remains not indicated due to the stable disease status. VCD has already shown effectiveness, hence no need for new combinations.

**===== run_3 =====**

**Tumor board decision:**
Recommendation: Local irradiation of progressive BWK 2 with stable systemic remission and constant VEGF value. No systemic therapy change required.

**Rationale:**
The isolated local progression of BWK 2 with stable systemic remission and constant VEGF value suggests a locally limited disease. Local irradiation is established to control isolated lesions without altering systemic therapy. VCD has already shown response, therefore no need for new combination therapies.

## ========================================================== qwen3-32b (plain) ==========================================================

**===== run_1 =====**

**Tumor board decision:**
Recommendation: Local radiotherapy of the progressive BWK 2 lesion. Continue systemic therapy only if progression occurs outside the treated region.

**Rationale:**
The isolated local progression with stable systemic remission (VEGF-stable) suggests locally limited resistance. Radiation therapy is the established standard for focal progression. Further systemic therapy is reserved if systemic control worsens.

**===== run_2 =====**

**Tumor board decision:**
Recommendation: Local irradiation of the progressive BWK 2 lesion with stable systemic remission. If there is further progression or uncertainty about the lesion: repeat PET/CT after 3 months.

**Rationale:**
The isolated local progression with stable VEGF and systemic PR suggests local resistance to therapy. Radiation therapy is established for focal POEMS lesions. Further systemic therapy only in the case of generalized progression.

**===== run_3 =====**

**Tumor board decision:**
Recommendation: Local radiotherapy of the progressive BWK 2 lesion with stable systemic remission. No systemic therapy changes required.

**Rationale:**
The isolated local progression with stable VEGF and systemic PR suggests a localized disease. Radiotherapy is the established standard for localized bone lesions in POEMS syndrome. VCD showed initial efficacy, therefore no evidence of systemic resistance.

## ========================================================== qwen3-next-80b (agent) ==========================================================

**===== run_1 =====**

**Tumor board decision:**

Local radiotherapy indicated for the isolated sclerotic bone lesion BWK 2. Do not adjust systemic therapy.

**Rationale:**
Isolated local progression with stable systemic parameters (VEGF stable, partial response) requires local radiotherapy as standard according to literature and conference data. Systemic therapy adjustment not required as there is no systemic progression.

**===== run_2 =====**

**Tumor board decision:**
Local radiotherapy of the isolated sclerotic bone lesion (BWK 2) is indicated. Adjustment of systemic therapy is not necessary.

**Rationale:**
Stable systemic parameters (VEGF 610 pg/ml, partial remission) with isolated local progression suggest local radiotherapy. POEMS syndrome with isolated bone lesion does not require systemic therapy adjustment if systemic stability is present. Radiation therapy is established for localized progression with stable systemic parameters.

**===== run_3 =====**

**Tumor board decision:**
Local radiotherapy indicated for the isolated sclerotic bone lesion (BWK 2). Adjustment of systemic therapy is not necessary.

**Rationale:**
Isolated local progression with stable systemic parameters (VEGF stable, partial remission) argues for local therapy. Systemic control exists, therefore no adjustment of systemic therapy is necessary. Local radiotherapy is standard for symptomatic individual lesions in similar plasmacytic diseases.

## ========================================================== qwen3-next-80b (plain) ==========================================================

**===== run_1 =====**

**Tumor board decision:**
Local radiotherapy to the progressive bone lesion BWK 2 with subsequent close clinical and laboratory monitoring (VEGF, bone imaging). Systemic therapy adjustment is not indicated because systemic remission (VEGF stable) is present.

**Rationale:**
Isolated local progression with stable systemic parameters (VEGF) argues for local therapy. Radiation therapy is established for POEMS isolated bone lesions. Systemic therapy (e.g. new VCD) does not make sense because there is no systemic progression and the VCD has already failed.

**===== run_2 =====**

**Tumor board decision:**
Local radiotherapy of the isolated bone lesion area BWK 2. Adjust or repeat systemic therapy not indicated as VEGF is stable and systemic remission remains.

**Rationale:**
Isolated local progression with stable systemic remission (VEGF, other lesions) requires focused treatment. Radiation therapy is standard for symptomatic single lesions in POEMS syndrome. Systemic therapy (e.g. new VCD) not necessary because there is no systemic progression and VEGF is stable.

**===== run_3 =====**

**Tumor board decision:**
Local radiotherapy of the affected bone (BWK 2) with subsequent close monitoring. Systemic therapy not indicated because VEGF is stable and systemic remission exists.

**Rationale:**
Isolated local progression with stable VEGF and systemic remission argues for local therapy. Radiation therapy is established for symptomatic bone lesions in POEMS. Systemic therapy is not repeated because VEGF stability is not an indication.

# mm_case_15__ALL

################################
#### CASE: 15
################################

## ========================================================= gpt-5-mini (agent) =========================================================

**===== run_1 =====**

**Tumor board decision:**
Recommendation: Induction with daratumumab + bortezomib + lenalidomide + dexamethasone (Dara-VRd) for 3 cycles (Dara s.c. preferred). Stem cell mobilization after cycle 3 with G-CSF +/- cyclophosphamide and collection of sufficient CD34+ cells. Consolidation: high-dose therapy with autologous SCT (melphalan 200 mg/m2, 1x). Lenalidomide maintenance (10 mg). Continue therapeutic anticoagulation; Lenalidomide initially reduced dose; Initiate osteoprotection and infection prophylaxis.

**Rationale:**

**Rationale: SLiM-CRAB (disseminated osteolysis) makes active therapy mandatory. For R-ISS II and no contraindications, the HDT concentric approach is standard; According to the guidelines, Dara-VRd is considered to be the strongest induction. Early mobilization after 3 cycles avoids mobilization problems caused by prolonged exposure to lenalidomide. Z.n. LAE requires continued therapeutic anticoagulation and careful lenalidomide dose initiation; no high-risk genetics -> no tandem SCT.**

**===== run_2 =====**

**Tumor board decision:**
Recommendation: Induction with Dara-VRd (daratumumab s.c. + bortezomib + lenalidomide + dexamethasone) for 3-4 cycles; Stem cell mobilization after cycle 3 (G-CSF +/- Plerixafor); then high-dose therapy with autologous SCT (melphalan 200 mg/m2) as consolidation; then lenalidomide maintenance. Initiate osteoprotection and prophylaxis.

**Rationale:**
SLiM-CRAB (disseminated osteolysis) and R-ISS II justify initial therapy. Dara-VRd achieves deep remissions before ASCT (guideline). Mobilization after cycle 3 minimizes collection problems caused by lenalidomide. If there is a history of VTE, continue therapeutic anticoagulation and initially reduce the lenalidomide dose; Thrombosis prophylaxis, infection and bone protection are mandatory.

**===== run_3 =====**

**Tumor board decision:**
Patient with symptomatic MM (R-ISS II, standard risk). Recommendation: Induction with Dara-VRd (daratumumab + bortezomib + lenalidomide + dexamethasone) for 3-4 cycles; Stem cell mobilization after cycle 3-4 (G-CSF +/- cyclophosphamide; plerixafor if necessary); subsequent autologous high-dose therapy (melphalan 200 mg/m2) as consolidation; Maintenance Lenalidomide 10 mg. If Dara-VRd not available/off-label: VRd.

**Rationale:**
SLiM-CRAB (disseminated osteolysis) requires immediate systemic therapy. Dara-VRd increases response rate before ASCT; Standard risk and R-ISS II justify a single ASCT. Continue therapeutic anticoagulation due to previous pulmonary embolism; Reduce lenalidomide initially (1st cycle) and thrombosis prophylaxis/coordination with hemostaseology. Additionally: osteoprotection (bisphosphonate/denosumab) and infection prophylaxis.

## ========================================================= gpt-5-mini (plain) =========================================================

**===== run_1 =====**

**Tumor board decision:**
Beginning of systemic induction. If suitable for ASCT: 4 cycles of D-VRd (daratumumab + bortezomib + lenalidomide + dexamethasone) -> stem cell mobilization and high-dose therapy/ASCT. If you are not suitable for ASCT: D-Rd. Continue therapeutic anticoagulation and, with lenalidomide, thrombosis prophylaxis; Bisphosphonates, pain and infection prophylaxis.

**Rationale:**

Symptomatic MM with disseminated osteolysis, R-ISS II, standard risk and no previous systemic therapy. Daratumumab-based induction achieves high remission rates before ASCT; If there is a known pulmonary embolism, anticoagulation/thrombosis prophylaxis is mandatory. Supportive measures (bisphosphonates, VZV prophylaxis) are recommended.

**===== run_2 =====**

**Tumor board decision:**
Initiation of systemic first-line therapy. If transplantable: induction with daratumumab-bortezomib-lenalidomide-dexamethasone (Dara-VRd) 3-4 cycles, stem cell mobilization/harvest and subsequent autologous stem cell transplantation. If not transplantable: Dara-Rd. Always therapeutic anticoagulation/strict thrombosis prophylaxis because of previously known pulmonary embolism, zoledronic acid q4w for bone protection and antiviral prophylaxis (e.g. acyclovir).

**Rationale:**
standard risk MM without previous systemic therapy; Dara-VRd achieves high remission rates and is first-line for transplant recipients. History PE requires therapeutic anticoagulation rather than just aspirin. Osteolysis warrants bisphosphonate. Infection prophylaxis recommended with CD38 antibodies/proteasome inhibitors.

**===== run_3 =====**

**Tumor board decision:**
Start of first-line systemic therapy with daratumumab + bortezomib + lenalidomide + dexamethasone (Dara-VRd) as induction; Parallel clarification of autologous transplant suitability. If transplant eligible, alternative: daratumumab + lenalidomide + dexamethasone (Dara-Rd). Continue therapeutic anticoagulation for PE and initiate HSV prophylaxis.

**Rationale:**
Symptomatic MM with disseminated osteolysis and no previous systemic therapy. Dara-based induction achieves high remission rates and allows stem cell mobilization with standard risk cytogenetics. History of pulmonary embolism requires therapeutic anticoagulation and adjustment of VTE prophylaxis with lenalidomide; Acyclovir because of bortezomib.

## ============================================================ gpt-5-nano (agent) ============================================================

**===== run_1 =====**

**Tumor board decision:**
first-line induction with DaraVRd or IsaVRd; then consolidation with autologous SCT; subsequent maintenance with lenalidomide; local therapies only for acute indications; consistent thromboembolism, infection and osteoprotection.

**Rationale:**
SLiM-CRAB fulfilled; R-ISS II, standard risk karyotype; ED-MM despite local treatment; expected to be fit for high-dose SCT; SCT improves depth of remission; CD38-naïve; no previous therapies; Prophylaxis (VTE, infections, osteoprotection) is essential.

**===== run_2 =====**

**Tumor board decision:**
Induction: DaraVRd or IsaVRd; Consolidation: Autologous stem cell transplantation; Maintenance: Lenalidomide 10 mg. Continue thromboembolism prophylaxis for LAE; Reduce lenalidomide dose in Cycle 1. Local RT only for acute symptoms/neurological deficits; further local therapies depending on symptoms.

**Rationale:**
SLiM-CRAB positive, active MM despite standard risk; potentially SCT-eligible patient; Triplet induction increases response rate, SCT enables deep remission; LAE requires anticoagulation; Only use local therapies symptomatically.

**===== run_3 =====**

**Tumor board decision:**
Induction: DaraVRd (daratumumab + bortezomib + lenalidomide + dexamethasone) as first-line in SLiM-CRAB+, R-ISS II. Check suitability for high-dose therapy; If suitable, autologous SCT (melphalan 200 mg/m2) for consolidation, followed by lenalidomide maintenance. No additional local therapy necessary. Prophylaxis: osteoprotection, infection and thromboembolism; Reduce lenalidomide dose in

cycle 1 if history of VTE.

**Rationale:**
SLiM-CRAB+, R-ISS II, standard risk; no high-risk genetics. Systemic first-line makes sense after spinal RT; SCT increases CR rate and long-term survival. Local therapies not necessary. History of VTE requires anticoagulation and reduction of lenalidomide dose in cycle 1. Maintenance advisable.

## ============================================================ gpt-5-nano (plain) ============================================================

**===== run_1 =====**

**Tumor board decision:**
Induction treatment for newly diagnosed ED MM standard risk: VRd induction (bortezomib-lenalidomide-dexamethasone) 3-4 cycles, followed by autologous SCT if transplantable; then lenalidomide maintenance. VTE prophylaxis due to previous PE (DOAC or LMWH).

**Rationale:**
Standard risk MM, symptomatic with disseminated osteolysis; no systemic therapy yet; ASCT increases long-term control; consider VTE risk; regular monitoring.

**===== run_2 =====**

**Tumor board decision:**
Induction EDMM: daratumumab + bortezomib + lenalidomide + dexamethasone (D-VRd) for 4 cycles; ASCT if eligible; then consolidation (Rd) and maintenance lenalidomide; Thromboprophylaxis with apixaban 2.5 mg BID; local RT only for symptomatic lesions.

**Rationale:**
EDMM with standard risk, no pretreatment; D-VRd enables rapid deep response in EDMM; ASCT improves outcomes in transplant-eligible patients; Thrombosis prophylaxis is essential due to a history of VTE; RT only if symptomatic.

**===== run_3 =====**

**Tumor board decision:**
Induction for extramedullary MM from solitary plasmacytoma: Initiate daratumumab-bortezomib-lenalidomide-dexamethasone induction (D-VRd) for 4-6 cycles. If transplantable: ASCT followed by consolidation and long-term maintenance (lenalidomide +/- daratumumab). For symptomatic lesions, add radiotherapy.

**Rationale:**
ED-MM requires robust CD38-targeted PI-based induction; D-VRd shows good effectiveness. If suitable, ASCT makes sense; Standard risk, no previous treatments. Radiotherapy only for local symptoms.

## ============================================================ gpt-oss-20b (agent) ============================================================

**===== run_1 =====**

**Tumor board decision:**
Induction with DaraVRd (daratumumab+bortezomib+lenalidomide+dexamethasone), followed by autologous stem cell transplantation (melphalan200mg/m2) and then lenalidomide maintenance (10mg daily).

**Rationale:**
Patient has no prior MM therapy, R-ISSII, standard risk and no contraindications to high-dose therapy. DaraVRd offers the highest first-line response rate; ASCT improves long-term outcomes. Lenalidomide maintenance is standard in standard-risk patients. Thromboembolism prophylaxis with lenalidomide is required after previous PE.

**===== run_2 =====**

**Tumor board decision:**
Induction with DaraVRd (daratumumab+bortezomib+lenalidomide+dexamethasone) 4-6 cycles, followed by autologous stem cell transplantation (melphalan 200mg/m2) and subsequent lenalidomide maintenance (10mg daily).

**Rationale:**
Patient meets SLiM-CRAB criteria, R-ISSII, standard risk, no comorbidities, therefore suitable for high-dose therapy. DaraVRd is the evidence-based first-line therapy with a high remission rate.

Autologous SCT improves survival, maintenance with lenalidomide prolongs remission.

**===== run_3 =====**

**Tumor board decision:**
Induction with DaraVRd (daratumumab+bortezomib+lenalidomide+dexamethasone) followed by autologous stem cell transplantation (melphalan200mg/m2) and then lenalidomide maintenance. In case of comorbidity or frailty: DaraRd or VRd instead of DaraVRd.

**Rationale:**
Fit patient without previous systemic therapy, R-ISSII, standard risk -> high-dose therapy according to guidelines. DaraVRd offers the highest remission rate, autologous SCT improves survival, lenalidomide maintenance extends progression-free time. Comorbidities would require alternative regimens.

## ============================================================ gpt-oss-20b (plain) ============================================================

**===== run_1 =====**

**Tumor board decision:**
Introduction of a first-line combination daratumumab + lenalidomide + dexamethasone (DRd) with thrombosis prophylaxis (e.g. LMWH/DOAC) for the patient's transplant-eligible, standard-risk MM.

**Rationale:**
No prior systemic therapy, R-ISSII, standard risk. DRd is evidence-based, well tolerated, and with a history of VTE, the risk is minimized with prophylaxis.

**===== run_2 =====**

**Tumor board decision:**
Induction with VRd (bortezomib 1.3mg/m2, lenalidomide 25mg, dexamethasone 40mg) 4-6 cycles, followed by autologous stem cell transplantation (ASCT) and then lenalidomide maintenance.

**Rationale:**
Standard risk, no contraindications, no MM therapy to date; VRd offers the highest response rate, ASCT improves OS, maintenance extends PFS.

**===== run_3 =====**

**Tumor board decision:**
Start first-line therapy with VRd (bortezomib 1.3mg/m2 SC q2Wo, lenalidomide 25mg PO d1-21 q28Wo, dexamethasone 40mg PO d1,8,15,22) for 4-6 cycles. If suitable for a transplant, then autologous stem cell transplantation. Schedule prophylactic anticoagulation for prior pulmonary artery embolism.

**Rationale:**
Patient has not yet received systemic therapy, R-ISSII, standard risk cytogenetics and no contraindications to proteasome inhibitors. Lenalidomide increases risk of thrombosis; therefore anticoagulation is necessary. VRd is the evidence-based first-line option for transplant-eligible or intransplant-eligible patients.

## ============================================================ gpt-oss-120b (agent) ============================================================

**===== run_1 =====**

**Tumor board decision:**
Induction with DaraVRd (daratumumab+bortezomib+lenalidomide+dexamethasone) -> autologous stem cell transplant (Melphalan200mg/m2) -> lenalidomide maintenance (10mg daily). Osteoprotection and continuation of anticoagulation due to previous pulmonary embolism, IMiD dose reduced in the first cycle if necessary.

**Rationale:**
Patient meets SLiM-CRAB, is eligible for therapy, no contraindications for high-dose therapy; R-ISSII and standard cytogenetics meet the guideline recommendation for DaraVRd+SCT+lenalidomide maintenance, providing the highest remission and progression-free survival rates. Thrombosis prophylaxis because of previous PE is mandatory.

**===== run_2 =====**

**Tumor board decision:**
Induction with DaraVRd (daratumumab, bortezomib, lenalidomide, dexamethasone), followed by autologous stem cell transplantation, then lenalidomide maintenance; Continue thrombosis prophylaxis, if necessary reduce the lenalidomide dose in the first cycle.

**Rationale:**
Patient complies with SLiM-CRAB, is fit and has no contraindication for high-dose therapy; for R-ISSII and standard risk, DaraVRd -> ASCT -> lenalidomide maintenance is recommended by guidelines. Previous pulmonary embolism requires further anticoagulation and initial dose reduction of lenalidomide.

**===== run_3 =====**

**Tumor board decision:**
Induction with DaraVRd (daratumumab, bortezomib, lenalidomide, dexamethasone) 4-6 cycles, followed by autologous stem cell transplantation (melphalan 200mg/m2) and maintenance with lenalidomide 10mg daily (+/- dexamethasone) with continued anticoagulation.

**Rationale:**
The patient has symptomatic MM (SLiM-CRAB), is treatment-naïve, has standard-risk cytogenetics and no contraindications to high-dose therapy. DaraVRd Delivers Deep Remissions, Enables Successful SCT; Lenalidomide maintenance improves progression-free survival. Previous pulmonary embolism requires anticoagulation and possibly a reduced Len dose in the first cycle.

## ============================================================ gpt-oss-120b (plain) ============================================================

**===== run_1 =====**

**Tumor board decision:**
Induction with daratumumab-bortezomib-lenalidomide-dexamethasone (Dara-VRd) for 4-6 cycles, followed by autologous stem cell transplantation and lenalidomide maintenance (daratumumab optional).

**Rationale:**
The patient has so far only received local therapy, no systemic therapy, R-ISSII, standard risk genetics and is transplant eligible. Dara-VRd+ASCT is the current standard regimen for newly diagnosed MM and improves response and overall survival.

**===== run_2 =====**

**Tumor board decision:**
Start induction therapy with daratumumab+bortezomib+lenalidomide+dexamethasone (Dara-VRd) for 4-6 cycles, followed by autologous stem cell transplantation (if mobilizable) and subsequent lenalidomide maintenance.

**Rationale:**
Patient has R-ISSII MM with standard cytogenetics, no contraindications (only previous pulmonary embolism allowing anticoagulation). Dara-VRd delivers deep remissions and is recommended for transplant-eligible patients.

**===== run_3 =====**

**Tumor board decision:**
Induction with daratumumab-bortezomib-lenalidomide-dexamethasone (Dara-VRd) 4-6 cycles, then autologous stem cell transplantation (if suitable for transplant), then lenalidomide maintenance +/- daratumumab.

**Rationale:**
MM R-ISSII, standard risk, no relevant contraindications. Dara-VRd achieves the highest deep remission rate and is well tolerated, even with previous local therapy and previous pulmonary embolism. Transplant increases overall survival; Maintenance reduces the risk of recurrence.

## ============================================================ qwen3-32b (agent) ============================================================

**===== run_1 =====**

**Tumor board decision:**
Recommended therapy initiation: DaraVRd (daratumumab + bortezomib + lenalidomide + dexamethasone) as induction therapy, followed by autologous stem cell transplantation (melphalan 200mg/m2) and

lenalidomide maintenance. Osteoprotection (denosumab/bisphosphonates) and thromboembolism prophylaxis are mandatory.

**Rationale:**
SLiM-CRAB criteria fulfilled (disseminated osteolysis). Patient is suitable for high-dose therapy (no contraindications). DaraVRd is first-line standard at standard risk. Previous radiotherapy warrants osteoprotection. Thromboembolism prophylaxis due to lenalidomide use and history of LAE.

**===== run_2 =====**

**Tumor board decision:**
Recommended first-line therapy: DaraVRd (daratumumab + bortezomib + lenalidomide + dexamethasone) as induction, followed by autologous stem cell transplantation (ASCT) and lenalidomide maintenance. Prophylaxis: osteoprotection, thromboembolism prophylaxis.

**Rationale:**
SLiM-CRAB criteria fulfilled (disseminated osteolysis). Standard risk cytogenetics and no contraindications to high-dose therapy. DaraVRd is the first-line standard in fit patients. ASCT after induction on R-ISS II. Lenalidomide maintenance after consolidation. Prophylaxis according to guidelines.

**===== run_3 =====**

**Tumor board decision:**
Induction with DaraVRd followed by autologous SCT (melphalan 200mg/m2) and maintenance with lenalidomide 10mg. Osteoprotection and thromboprophylaxis are mandatory.

**Rationale:**
SLiM-CRAB criteria fulfilled (disseminated osteolysis). Patient is fit for high-dose therapy (no contraindications). DaraVRd is first-line standard at standard risk. SCT consolidated, lenalidomide as maintenance. Thromboprophylaxis due to history of LAE.

## ============================================================ qwen3-32b (plain) ============================================================

**===== run_1 =====**

**Tumor board decision:**
Recommendation: Initiation of therapy with RVD (lenalidomide, bortezomib, dexamethasone) as induction therapy. After response autotransplantation (ASCT) if transplantable.

**Rationale:**
Newly diagnosed MM with standard risk cytogenetics and CRAB criteria. RVD is the established standard for induction in fit patients. ASCT optimizes long-term prognosis with transplantability.

**===== run_2 =====**

**Tumor board decision:**
Recommendation: Initiation of therapy with RVD (lenalidomide, bortezomib, dexamethasone) as induction therapy, followed by autologous stem cell transplantation (ASCT) if transplantable and subsequent lenalidomide maintenance.

**Rationale:**
Newly diagnosed MM with standard risk cytogenetics and CRAB criteria. RVD is an established standard in fit patients. ASCT optimizes long-term prognosis. No known contraindications.

**===== run_3 =====**

**Tumor board decision:**
Recommendation: Initiation of therapy with VRd (bortezomib, lenalidomide, dexamethasone) as an induction regimen for newly diagnosed MM with standard risk cytogenetics.

**Rationale:**
VRd is standard therapy in fit patients with newly diagnosed MM. No contraindications, no previous therapies, SLiM-CRAB criteria met. Standard risk cytogenetics does not justify more intensive therapy.

## ============================================================ qwen3-next-80b (agent) ============================================================

===== run_1 =====

**Tumor board decision:**
Induction therapy with daratumumab + bortezomib + lenalidomide + dexamethasone (DaraVRd), lenalidomide dose reduced (10-15 mg) with continued therapeutic anticoagulation. Subsequently, autologous stem cell transplantation and lenalidomide maintenance therapy. Osteoprotection with denosumab/bisphosphonates.

**Rationale:**
SLiM-CRAB criteria fulfilled (disseminated osteolysis). No contraindications to high-dose therapy (ECOG/age not specified, but no comorbidities precluding ASCT). DaraVRd is standard for transplant-eligible patients. Prioritize thrombosis prophylaxis by therapeutic anticoagulation and lenalidomide dose reduction in the presence of a history of pulmonary artery embolism.

===== run_2 =====

**Tumor board decision:**
Induction with DaraVRd (daratumumab + bortezomib + lenalidomide + dexamethasone), lenalidomide dose reduced to 10-15 mg in the first cycle, continue therapeutic anticoagulation. Subsequently, autologous stem cell transplantation (ASCT) if eligible, followed by lenalidomide maintenance.

**Rationale:**
SLiM-CRAB criteria fulfilled (disseminated osteolysis). Patient is suitable for high-dose therapy (no contraindications mentioned). DaraVRd is standard induction at standard risk. Lenalidomide dose adjustment due to Z.n. Pulmonary artery embolism (therapeutic anticoagulation + reduced dose). ASCT consolidation and lenalidomide maintenance are consistent.

===== run_3 =====

**Tumor board decision:**
Induction with daratumumab, bortezomib, lenalidomide (10 mg) and dexamethasone (DaraVRd) under therapeutic anticoagulation (e.g. rivaroxaban) and osteoprotection (denosumab). Followed by autologous stem cell transplantation in case of remission.

**Rationale:**
SLiM-CRAB criteria met (disseminated osteolysis), R-ISS II, standard risk. No previous MM therapy. Due to Z.n. Pulmonary artery embolism, the lenalidomide dose is reduced (10 mg) and therapeutic anticoagulation is increased. High-dose therapy suitability assumed, therefore DaraVRd as induction.

## ============================================================ qwen3-next-80b (plain) ============================================================

===== run_1 =====

**Tumor board decision:**
Initiation of VRd (bortezomib, lenalidomide, dexamethasone) with prophylactic anticoagulation due to history of VTE

**Rationale:**
No prior systemic therapy, standard risk MM. VRd is first-line therapy. VTE risk increased by lenalidomide, therefore anticoagulation required. No known contraindications.

===== run_2 =====

**Tumor board decision:**
Initiation of VRd (bortezomib, lenalidomide, dexamethasone) with thrombosis prophylaxis (apixaban), followed by ASCT if transplant eligible.

**Rationale:**
Progression from solitary plasmacytoma to MM with R-ISS II/standard risk requires systemic therapy. VRd is established first-line therapy. History of VTE requires anticoagulation. ASCT examination if clinically suitable.

===== run_3 =====

**Tumor board decision:**
Initiation of D-VRd (daratumumab, bortezomib, lenalidomide, dexamethasone) as induction therapy. If transplantable, follow-up with ASCT. Thromboprophyaxis with LMWH due to history of pulmonary artery embolism.

**Rationale:**

Standard therapy for newly diagnosed MM (R-ISS II, standard risk). D-VRd shows high response rates. LMWH for thrombosis prophylaxis with current anticoagulation. No contraindications for the medication.
