# Supplementary material for: Clinical decision support in hematological malignancies using a case-grounded AI agent
Source: Nat Med. 2026 Jun 30;32(9):3224–34. doi: 10.1038/s41591-026-04494-4 (PMC13577921; doi:10.1038/s41591-026-04494-4)
Supplement: Supplementary file 2 [file 41591_2026_4494_MOESM2_ESM.pdf]

nature portfolio

Corresponding author(s): Mirco J. Friedrich, MD, PhD

Last updated by author(s): May 18, 2026

Reporting Summary

Nature Portfolio wishes to improve the reproducibility of the work that we publish. This form provides structure for consistency and transparency in reporting. For further information on Nature Portfolio policies, see our Editorial Policies and the Editorial Policy Checklist.

Statistics

For all statistical analyses, confirm that the following items are present in the figure legend, table legend, main text, or Methods section.

n/a	Confirmed	
☐	☒	The exact sample size (*n*) for each experimental group/condition, given as a discrete number and unit of measurement
☐	☒	A statement on whether measurements were taken from distinct samples or whether the same sample was measured repeatedly
☐	☒	The statistical test(s) used AND whether they are one- or two-sided<br>*Only common tests should be described solely by name; describe more complex techniques in the Methods section.*
☐	☒	A description of all covariates tested
☐	☒	A description of any assumptions or corrections, such as tests of normality and adjustment for multiple comparisons
☐	☒	A full description of the statistical parameters including central tendency (e.g. means) or other basic estimates (e.g. regression coefficient) AND variation (e.g. standard deviation) or associated estimates of uncertainty (e.g. confidence intervals)
☐	☒	For null hypothesis testing, the test statistic (e.g. F, t, r) with confidence intervals, effect sizes, degrees of freedom and P value noted<br>*Give P values as exact values whenever suitable.*
☒	☐	For Bayesian analysis, information on the choice of priors and Markov chain Monte Carlo settings
☒	☐	For hierarchical and complex designs, identification of the appropriate level for tests and full reporting of outcomes
☐	☒	Estimates of effect sizes (e.g. Cohen's *d*, Pearson's *r*), indicating how they were calculated

Our web collection on statistics for biologists contains articles on many of the points above.

Software and code

Policy information about availability of computer code

Data collection	Tumor board data was retrieved via the BRAIN database from the Department of Hematology, Oncology and Rheumatology (Internal Medicine V), Heidelberg University Hospital and from the Department of Hämatology and Oncology (Internal Medicine III), LMU Clinic. To construct our flowcharts we manually gathered data from German Onkopedia guidelines and internal SOPs.<br>Most data that was collected and processed is requested via python library requests (≥2.31.0). Some data (cosmic) was manually downloaded. Other libraries which were used for data gathering and processing: biopython (≥1.83), python-docx (≥1.2.0).<br>The following databases and APIs were used: PubMed/NCBI Entrez (https://www.ncbi.nlm.nih.gov/), Crossref (https://api.crossref.org/), gnomAD v4 (https://gnomad.broadinstitute.org/), MyVariant.info (https://myvariant.info/), CIViC (https://civicdb.org/), OncoKB (https://www.oncokb.org/), Ensembl VEP (https://rest.ensembl.org/), Cancer Hotspots (https://www.cancerhotspots.org/), UniProt (https://www.uniprot.org/), and COSMIC v103 (https://cancer.sanger.ac.uk/cosmic/). Parts of the data we used is not open-access, e.g. the cosmic data set (Cosmic_CompleteTargetedScreensMutant_v103_GRCh38) and therefore cannot be provided by us.<br>Code to test HemaGuide and reproduce our study can be found here: https://github.com/Friedrich-Lab/HemaGuide
Data analysis	We analyzed the data with Python 3.12+ and R 4.5.1. Core packages used were: numpy (≥1.24.0), pandas (≥2.0.0) and scipy (≥1.10.0). For dimensionality reduction we used scikit-learn (≥1.3.0), umap-learn (≥0.5.0), pacmap (≥0.7.0), trimap (≥1.1.0) and phate (≥1.0.0) in Python, and umap (0.2.10.0), uwot (0.2.4) and Rtsne (0.17) in R. For clustering quality evaluation and k-NN classification we used FNN (1.1.4.1), class (7.3-23), cluster (2.1.8.1) and MASS (7.3-65) in R. Data manipulation in R used dplyr (1.1.4) and stringr (1.6.0). We visualized data with matplotlib (≥3.7.0), seaborn (≥0.12.0) and ggplot2 (4.0.1). Moreover, we implemented the OpenAI Python API (1.107.1) and ollama (≥0.4.4) for LLM interactions and used ChromaDB (1.0.20) as a vector store.

For manuscripts utilizing custom algorithms or software that are central to the research but not yet described in published literature, software must be made available to editors and reviewers. We strongly encourage code deposition in a community repository (e.g. GitHub). See the Nature Portfolio guidelines for submitting code & software for further information.

Data

Policy information about availability of data

All manuscripts must include a data availability statement. This statement should provide the following information, where applicable:

- Accession codes, unique identifiers, or web links for publicly available datasets
- A description of any restrictions on data availability
- For clinical datasets or third party data, please ensure that the statement adheres to our policy

All constructed clinical cases used for benchmarking, along with the corresponding agent outputs, are available in the Supplementary Information.

Research involving human participants, their data, or biological material

Policy information about studies with human participants or human data. See also policy information about sex, gender (identity/presentation), and sexual orientation and race, ethnicity and racism.

Reporting on sex and gender	We did not perform real-world gender- or sex-based analyses in our study.
Reporting on race, ethnicity, or other socially relevant groupings	We did not perform real-world race- or ethnicity-based analyses in our study.
Population characteristics	We used all available patient cases that required an interdisciplinary tumor board evaluation between 2024-2025 at Heidelberg University Hospital, Heidelberg, Germany and Ludwig-Maximilians-University Hospital, Munich, Germany.
Recruitment	n/a
Ethics oversight	This study framework was approved by the Ethics committee of the Medical Faculty of Heidelberg University (reference S-837/2019 and S-149/2026).

Note that full information on the approval of the study protocol must also be provided in the manuscript.

Field-specific reporting

Please select the one below that is the best fit for your research. If you are not sure, read the appropriate sections before making your selection.

☒ Life sciences ☐ Behavioural & social sciences ☐ Ecological, evolutionary & environmental sciences

For a reference copy of the document with all sections, see nature.com/documents/nr-reporting-summary-flat.pdf

Life sciences study design

All studies must disclose on these points even when the disclosure is negative.

Sample size	Sample size was kept small for benchmarking cases as a proof-of-concept with extensive human evaluations: n1=45 cases x m=12 LLM runs. n2=555 cases for external validation from Munich. n3=64 cases for internal silent trial.
Data exclusions	We did not exclude any data from the analyses in this study.
Replication	Data replication is challenging especially due to the usage of several proprietary AI models (e.g., GPT-5 family) to which users have limited access to and that given its guardrails avoiding potentially harmful content, sometimes refuses in answering adequately to medical queries. In these cases we re-ran each sample in a newly instantiated setting until no refusals occurred. Additionally, reproducibility might be affected by silent changes that are made to the models and not disclosed to the public by the owners of the model (OpenAI) or by availabilities of the models. We used several data sources with limited availability, like PubMed api (email address, API-key for higher request limits), COSMIC database (free use for members of research institutions, registration only), internal hematology SOP (access limited to staff of University Hospital Heidelberg).
Randomization	There was only one pre-specified evaluation group (AI-agent or resident physician vs. senior physician) per experiment, so we could not perform randomization.
Blinding	Human evaluators performed the evaluations independently. Rating model answers was fully blinded to the model and if HemaGuide was implemented or not. Furthermore, humans were blinded to the models answers when defining the ground truth for required tool use, relevance of PubMed retrieval or similar clinical case and completeness of the model responses.

Reporting for specific materials, systems and methods

We require information from authors about some types of materials, experimental systems and methods used in many studies. Here, indicate whether each material, system or method listed is relevant to your study. If you are not sure if a list item applies to your research, read the appropriate section before selecting a response.

Materials & experimental systems

n/a	Involved in the study
☒	☐ Antibodies
☒	☐ Eukaryotic cell lines
☒	☐ Palaeontology and archaeology
☒	☐ Animals and other organisms
☒	☐ Clinical data
☒	☐ Dual use research of concern
☒	☐ Plants

Methods

n/a	Involved in the study
☒	☐ ChIP-seq
☒	☐ Flow cytometry
☒	☐ MRI-based neuroimaging

Plants

Seed stocks

Report on the source of all seed stocks or other plant material used. If applicable, state the seed stock centre and catalogue number. If plant specimens were collected from the field, describe the collection location, date and sampling procedures.

Novel plant genotypes

Describe the methods by which all novel plant genotypes were produced. This includes those generated by transgenic approaches, gene editing, chemical/radiation-based mutagenesis and hybridization. For transgenic lines, describe the transformation method, the number of independent lines analyzed and the generation upon which experiments were performed. For gene-edited lines, describe the editor used, the endogenous sequence targeted for editing, the targeting guide RNA sequence (if applicable) and how the editor was applied.

Authentication

Describe any authentication procedures for each seed stock used or novel genotype generated. Describe any experiments used to assess the effect of a mutation and, where applicable, how potential secondary effects (e.g. second site T-DNA insertions, mosiacism, off-target gene editing) were examined.
