# Supplementary material for: Clinical decision support in hematological malignancies using a case-grounded AI agent
Source: Nat Med. 2026 Jun 30;32(9):3224–34. doi: 10.1038/s41591-026-04494-4 (PMC13577921; doi:10.1038/s41591-026-04494-4)
Supplement: Supplementary file 3 [file 41591_2026_4494_MOESM3_ESM.pdf]

nature portfolio

Peer Review File

Clinical decision support in hematological malignancies using a case-grounded AI agent

Corresponding Author: Dr Mirco Friedrich

Parts of this Peer Review File have been redacted as indicated to remove third-party material.

This file contains all reviewer reports in order by version, followed by all author rebuttals in order by version.

Version 0:

Reviewer comments:

Reviewer #1

(Remarks to the Author)
This manuscript presents an AI tool for clinical decision-making support in the care of patients with hematologic malignancies. The tools is an LLM agent which extracts clinical information from unstructured clinical documents and then creates recommendation of treatment based on a combination of clinical guidelines and a large number of real-world tumor board cases. The authors evaluated the quality of the tool in (1) comparison to clinical evaluation, and (2) a study comparing the recommendations from medical residents to senior clinical experts without versus with the use of HemaGuide.
Overall, this seems to be an interesting and potentially useful tool. The most commendable aspect of the manuscript is the rigorous evaluation the authors have performed in testing the tool and comparing the performance in residents +/- use of the tool. However, as a reader and reviewer, it is difficult to assess the utility of this tool directly as the recommendations made by the tool in the few cases presented are not particularly enlightening and the software currently seems to require Python for evaluation. In addition, there a number of real-world clinical issues where more clarity of description is needed. These issues are noted below:
-In Figure 4a-c, there is a comparison of the recommendations from HemaGuide compared to clinical decision making from physicians versus other predictors. While HemaGuide performed well for myeloma and lymphoma cases, for “leukemia” cases 7 of 10 recommendations were correct. While 7/10 appears here to be lauded as good performance, in clinical practice, this would clearly not be ideal for patient care. There is also no discussion about why the cases which did not perform well failed. “Leukemia” itself is a broad disease area and having more information about the specifics of which cases were accurately managed versus not would be important.
-How does HemaGuide deal with cases where the diagnosis is not clear based on clinical and pathologic review? This is not clear from the manuscript.
-What is the input information for HemaGuide? It seems that the input cases and tumor bord information came from patients at Heidelberg University Hospital based on the Methods but this is not clearly described in the Results section.
-Related to the above, I imagine the authors want this tool to be disseminated for use beyond the institution where this was created but it is not clear to me from the text if that is the goal of publishing this study versus proof of concept that such a tool with utility can be created.
-Practice recommendations often differ by country and availability to approved therapies may differ by country. How is this handled by HemaGuide?
-The example case in Figure 3d was helpful to see but I was surprised to see no mention of patient age, functional status, or prior treatments – which are essential in clinical decision making. This figure as described as displaying an “extraction of relevant clinical context” but is clearly missing critical clinical details.
-The examples of cases evaluated all seem to rely on mutational information to make recommendations about treatment plans. For example, in the case in Figure 3d, Figure 5, and Extended Data Figure 2, the cases and recommendations seem to focus on mutational information. However, only a minority of approved therapies for patients with hematologic malignancies are based on genetic factors (for example, almost none of the successful therapies in myeloma are linked to a specific gene mutation). Moreover, there are many clinical trials which are not linked to mutational genotype. In the cases in Figure 3 and Figure 5, it is difficult to imagine that the only clinical trial for which this patient could be eligible for would be a MEK inhibitor regimen.

(Remarks on code availability)

Reviewer #2

(Remarks to the Author)
The manuscript describes HemaGuide, an LLM-based agentic framework designed to provide clinical decision support for hematological malignancies. The system integrates a clinical case memory (CCM), guideline flowcharts, and a molecular interpretation module to generate structured treatment recommendations. While the architectural design and the end-to-end workflow are clearly articulated, the current version of the manuscript does not provide sufficiently strong evidence to support its claims regarding clinical reliability and impact. The main concerns involve the scale of validation, the lack of controlled baselines, and potential data integrity issues.

Comments
1. The authors utilize a substantial "Clinical Decision Memory" consisting of >2,000 real-world cases to ground the agent's reasoning. However, the primary clinical performance claims rely on an evaluation of only 30 high-complexity cases. This sample size is insufficient to validate the generalizability and robustness of the system across the diverse and heterogeneous landscape of hematological oncology.

2. The benchmark compares HemaGuide (an agent with retrieval and tool-use layers) against plain LLMs that lack access to any external evidence. This experimental setup fails to isolate whether the observed performance gains are a result of the proposed "agentic routing" or simply the addition of external data. A more informative evaluation would include a comparison against a standard, non-agentic RAG (Retrieval-Augmented Generation) pipeline with equivalent access to the CCM and guidelines. Ablation studies removing specific layers (e.g., the routing mechanism or the enrichment protocol) are necessary to justify the system's complexity.

3.The manuscript reports that the Clinical Case includes cases from 2024–2025. Given this timeframe, it is unclear how the authors ensured strict physical and temporal separation between the retrieval substrate and the evaluation queries. There is no quantitative reporting on patient-level de-duplication or similarity-based filtering to prevent the agent from simply retrieving its own test cases. Explicit documentation of overlap control is required to confirm that the results reflect reasoning capabilities rather than historical precedent retrieval.

4. The molecular validation is restricted to 70 single-nucleotide missense variants. In hematological malignancies, structural variants (SVs), fusions, and copy-number alterations are fundamental to clinical stratification. By focusing exclusively on missense mutations, the authors leave the system's performance on more complex—yet clinically essential—genomic events untested.

5. The evidence for clinical utility is primarily based on subjective Likert-scale scoring (Q1–Q6) from a small group of clinicians. The manuscript would be significantly strengthened by the inclusion of inter-rater reliability (IRR) metrics for these scores. Furthermore, the prospective performance is based on a simulated practice study. Without data from a silent trial or a real-world deployment setting, the claims regarding workflow benefits and performance augmentation remain speculative.

6. While the authors acknowledge that the Clinical Case is derived from a single academic center , this narrative admission is insufficient. The authors should provide quantitative evidence of the system's generalizability, such as a hold-out validation on an external dataset or a stress test in a resource-constrained setting, to demonstrate that the agent's logic is not overfitted to a single institution's therapeutic style and trial availability.

7. The entire reasoning pipeline depends on the accurate conversion of unstructured documents into a 10-field JSON schema, yet the manuscript lacks a formal evaluation of this critical upstream module. The authors must report field-level metrics, such as macro-F1 scores or exact match rates, and provide an error propagation analysis to determine how a single extraction failure impacts the accuracy of the final generated recommendation .

8. The current evaluation describes execution time only in general "minutes", which is insufficient for characterizing performance in time-sensitive oncology workflows. The authors should provide a detailed performance profile, including p50 and p95 latency for each decision mode and the hardware requirements (e.g., GPU/RAM configurations) necessary to support local deployment of high-capacity 120B parameter backbones .

9. The reliance on manually curated, plain-text flowcharts for guideline adherence raises significant scalability and maintenance concerns . To ensure long-term clinical safety, the authors must define a formal governance mechanism, including specific triggers for updates, a human sign-off process, and a regression testing protocol to confirm that updates to one entity do not introduce unintended logic drift in others.

10. The manuscript uses a cosine similarity threshold of 0.8 for case retrieval but fails to specify the agent's behavior when

no high-confidence precedents are found. A robust decision-support tool requires a formal abstention policy or behavior tree for low-confidence queries. The authors should provide a calibration analysis or minimum evidence criteria to clarify how the system signals uncertainty to clinicians rather than forcing a structured recommendation in low-evidence settings .

11.All data comes from Heidelberg University Hospital, covering just six cancer types. I am concerned about whether this will work elsewhere. Can the authors discuss: how results might differ in other populations or resource settings; what happens with rare cancers not in the training data; and whether multi-site validation is planned? A short paragraph in the Discussion would help.

12.I did not see much discussion of errors or hallucinations. Did HemaGuide get anything wrong on the 30 test cases? For the molecular variant calls, what are the false positive and negative rates? This matters for clinical safety, and even a brief note on error patterns would be valuable.

13: The clinician study compares decisions against tumor board consensus. I wonder: were the evaluating doctors blinded to the study aims? Also, why use single TB decisions rather than multiple expert opinions as the reference? The authors should clarify the evaluation setup and acknowledge potential bias sources.

14: The paper focuses on accuracy metrics, but I am curious about real-world use. How fast does the system generate recommendations? Does it connect to existing hospital EMR systems, or does data need to be entered manually? These details would help readers judge whether this is practical for busy clinics.

15: The ClinGen-based scoring framework in Figure 3 is reasonable, but how well does the automated classification match expert manual review? Some inter-rater reliability statistics would strengthen confidence in the molecular mode.

(Remarks on code availability)

Reviewer #3

(Remarks to the Author)

In this study, the authors report an AI agentic system for hematologic malignancy clinical decision support. The system is very complex, including retrieval from guidelines and SOPs, similar case retrieval, PubMed search, and Crossmed search. They evaluate a range of models across open and closed source models, and – promisingly – show that the system implemented with an open source model, gpt-oss-120, performs best (and outperforms vanilla LLM implementations). They include a small simulation study, where they show using the system improves trainee performance to the level of senior attending physicians (30 cases evaluated). I commend the authors, who have clearly put in a huge effort in developing and evaluating this system. It is really great that they provide their code, as well. However, there are several significant concerns that would need to be addressed for this to be acceptable for publication:

Major:
• Overall, this reads a bit too much like spec report and is generally very difficult to follow. Much of the very detailed methods can be moved to supplement to put the focus on the research experiments, not the engineering. The introduction runs into the results with no separation, which makes it challenging to review.
• The discussion of how the system architecture was arrived at and optimized, are not sufficiently reported. It is therefore not possible to assess if the architecture was overfit to the evaluated cases. There are also no ablation studies, and so the relative contributions of the different components of the system are not clear. Perhaps a much simpler agentic approach could perform similarly or even better.
• More detail on the clinical data types used in the retrieval database and the evaluated cases, is needed. Was there a development/test split? How were the 30 cases for evaluation selected and removed from the broader set used in retrieval?
• More information is needed on how the de-identified clinical content was generated/extracted from patient records. Presumably this requires a large amount of manual effort, which impacts the scalability of any approach relying on it. It is also a bit unclear when clinical notes were vs were not used in the Clinical Case Memory dataset and evaluated cases – how were these deidentified? How were the embedded document chunks vs LLM-extracted elements used, and what LLMs were used to extract the information?
• There are many claims without sufficient methods or result to contextualize them. For example, lines 137-139, how did the expert hematologist determine clinical similarity, what are the credentials of this individual, and how was retrieved case value determined? Was there dual-rating to assess quality of these determinations? There are no results for this presented.
• I may be missing it, but I don't see the full results across all models.
• For the simulated study, what was the inter-rater agreement across the survey criteria? This is critical to understand to assess quality of the system evaluation.
• How was the interface optimized? There is no report of any assessment of its usability.
• It would be interesting and also important, for implementation considerations, to report and compare costs (for API models) and latency across the models. Please also provide information on the compute hardware used to run the local models.
• It is hard to understand exactly how the human evaluation was conducted. How were the triplicate runs handled? What was the distribution across PGYs (PGY1 is very different than PGY5)? How many senior attendings participated? Were the cases randomized, or the participants? Were the participants consented? A dedicated schema for this portion would be helpful.

• The discussion should mention that performance improvement for attendings has not been assessed and is an important future direction.
• The discussion would benefit from mentioning that best-in-class API models (GPT5-m) benefit less from HemaGuide integration versus the open source models, and what this might mean for future developments.

Minor
• It may just be me, but I find the use of “substrates” to refer to documents for retrieval to be awkward and unnecessary. Corpora or datasets are more straightforward terms to consider.
• Figure 2D: Please order the LLMs in the same order for the HemaGuide section and the vanilla LLM section. That will make it a lot easier to compare.

(Remarks on code availability)

Version 1:

Reviewer comments:

Reviewer #1

(Remarks to the Author)
The authors have sufficiently addressed my prior comments and questions.

(Remarks on code availability)

Reviewer #2

(Remarks to the Author)
The authors’ point-by-point rebuttal and the revised manuscript have been carefully reviewed. Overall, the authors have addressed most of the comments and the concerns raised by other reviewers in a detailed and comprehensive manner, with supplementary experiments and textual revisions that are generally consistent with the expected standards. However, several minor issues still need to be clarified:

Comment 1: In response to Comment 7 from Reviewer #1, the authors have supplemented age information in the structured case representation. However, the authors performed transformation on patient age during the data preprocessing stage. It is imperative to clarify whether such modifications may introduce potential bias and thereby impact the diagnostic performance of the proposed model.

Comment 2: Regarding Comment 2 from Reviewer #2, the authors have revised Figure 2E. However, the accuracy values of the full model (L10) across different cases are 100%, 80%, and 80%, respectively, while the overall accuracy across all cases is reported as 100%. This inconsistency is confusing and necessitates a clear explanation or appropriate correction.

Comment 3: Several formatting errors persist in the revised text. For example, line 312 lacks proper indentation, and the sentence on line 585 is incomplete. These minor discrepancies should be rectified to enhance the presentation quality of the manuscript.

(Remarks on code availability)

Reviewer #3

(Remarks to the Author)
The authors have responded nicely to my comments. The primary limitation is the level of clinical evidence and the lack of comparison to other commonly used LLM-based clinical recommendation systems.

(Remarks on code availability)

Open Access This Peer Review File is licensed under a Creative Commons Attribution 4.0 International License, which permits use, sharing, adaptation, distribution and reproduction in any medium or format, as long as you give appropriate credit to the original author(s) and the source, provide a link to the Creative Commons license, and indicate if changes were made.
In cases where reviewers are anonymous, credit should be given to 'Anonymous Referee' and the source.
The images or other third party material in this Peer Review File are included in the article's Creative Commons license, unless indicated otherwise in a credit line to the material. If material is not included in the article's Creative Commons license and your intended use is not permitted by statutory regulation or exceeds the permitted use, you will need to obtain permission directly from the copyright holder.
To view a copy of this license, visit https://creativecommons.org/licenses/by/4.0/

Point-by-Point Response to Reviewers

Manuscript NMED-A149343A

A case-grounded AI agent for clinical decision support in hematological malignancies

Zoller, Kalz et al.

We thank the Editor and Reviewers for their thorough and constructive evaluation of our manuscript. Their comments have led to substantial improvements in the scope, rigor, and clarity of our work. In this revision, we have:

(1) expanded the benchmark cohort from 30 to 45 high-complexity cases and added a systematic ablation study across 11 architectural layers to isolate the contribution of each pipeline component (**new Fig. 2e**);

(2) conducted an external validation on 555 independent, de-identified tumor board cases from a second academic center (LMU Munich), which achieved 81.8% concordance across 47 hematological entities without re-optimization (**new Fig. 5**);

(3) completed a prospective one-month silent trial on 64 consecutive, unselected cases processed in parallel to clinical routine, with concordance of 82.8% (**new Fig. 6**);

(4) introduced a formal Tumor Board Concordance Score (TBCS), a 10-point composite metric with demonstrated inter-rater reliability (Cohen's $\kappa = 0.934$ for external validation; $\kappa = 0.795$ for silent trial);

(5) performed a structured error analysis stratifying all non-concordant cases by clinically meaningful failure categories (**new Fig. 6e**);

(6) evaluated extraction accuracy with field-level metrics (macro-F1 = 0.99, Cohen's $\kappa = 0.886$) and conducted a simulated error propagation analysis (**Point-by-Point Fig. 1; Point-by-Point Table 1**);

(7) added detailed latency profiling (p50, p95 per decision mode), hardware specifications, and a cost comparison across local and cloud-based deployment configurations (**Extended Data Fig. 6**);

(8) implemented a guideline versioning mechanism with automated guideline timestamp checking (**Point-by-Point Fig. 3**) and added a formal usability evaluation of our graphical user interface (**Extended Data Fig. 7**);

(9) restructured the manuscript to separate Introduction and Results, expanded the Discussion to address generalizability, attending-level evaluation, and the differential benefit of agentic architecture for open- versus closed-source models, and provided end-to-end case examples for all three decision modes (**Extended Data Figs. 3-4, Supplementary Notes**).

Below, we address each comment in detail.

Referee #1

Comment 1

However, as a reader and reviewer, it is difficult to assess the utility of this tool directly as the recommendations made by the tool in the few cases presented are not particularly enlightening and the software currently seems to require Python for evaluation.

We appreciate this feedback and have addressed both concerns substantively.

Regarding software accessibility, we have re-engineered *HemaGuide* for single-click deployment. The tool now ships as a self-contained package: a launcher file (HemaGuide.command on macOS) automatically configures the runtime environment including dependency installation and language model download and opens a browser-based interface. The only prerequisites are cloning the repository and installing Ollama, a free standard application for local language model inference. No Python knowledge or command-line interaction is required from the end user.

Regarding clinical transparency, we agree that the original manuscript did not sufficiently illustrate the system's decision-making process. We have therefore added comprehensive end-to-end case examples for all three decision modes. **Extended Data Figure 3** presents a Guideline Mode example (relapsed B-ALL with CNS involvement), showing the autonomous flowchart traversal and the resulting recommendation with explicit consideration and decision references. **Extended Data Figure 4** presents an Advanced Mode example (relapsed PMBCL after CAR-T and bispecific antibody therapy), illustrating the multi-source evidence synthesis including similar case retrieval, PubMed and Crossref literature integration, and the resulting treatment recommendation. The Molecular Mode end-to-end workflow was already shown in **Figure 3d** but has been supplemented with additional detail. Furthermore, **Supplementary Note 1** and **Supplementary Table 7-9** now provides all original tumor board decisions and corresponding *HemaGuide* outputs for every evaluated case, enabling direct comparison.

Comment 2

In Figure 4a-c, there is a comparison of the recommendations from HemaGuide compared to clinical decision making from physicians versus other predictors. While HemaGuide performed well for myeloma and lymphoma cases, for "leukemia" cases 7 of 10 recommendations were correct. While 7/10 appears here to be lauded as good performance, in clinical practice, this would clearly not be ideal for patient care. There is also no discussion about why the cases which did not perform well failed.

We thank the reviewer for this important critique. Several points merit clarification.

First, the 45 benchmark cases (15 per entity group) were deliberately selected for high complexity to maximize the discriminatory power between plain LLMs and *HemaGuide* across model families. They do not represent a real-world case mix and were not designed to estimate population-level concordance. For that purpose, we now provide two additional evaluation settings: an external validation on 555 independent clinical cases from Munich University Hospital (LMU; overall concordance 81.8%; leukemia concordance 84.6%, with AML specifically reaching 94.4%; **Fig. 5**) and a prospective silent trial on 64 consecutive, unselected cases (overall concordance 82.8%; leukemia concordance 88.9%; **Fig. 6**). These data provide a more representative estimate of real-world performance.

Second, and critically, non-concordance with the tumor board decision does not equate to a clinically "wrong" recommendation. Tumor board decisions themselves are subject to inter-board variability and are shaped by local expertise, trial availability, and institutional culture. To contextualize non-concordant cases, we have performed a structured error analysis across all evaluation cohorts, categorizing discordances into eight clinically meaningful types: alternate regimen selection (correct indication, alternative, but guideline-compliant regimen), understaging, overstaging, undertreatment, overtreatment, workflow error, contraindication non-recognition, and hallucination.

Across the silent trial, the most frequent discordance category was *alternate regimen selection* (e.g., *HemaGuide* recommending BCMA-directed CAR-T with prior bridging while the tumor board additionally specified a specific bridging regimen), where *HemaGuide's* recommendation was clinically defensible but differed in specifics. Hallucinations were rare (2/119 incorrect cases across all evaluation cohorts, mean 0.7% ± 0.7% SEM across three cohorts) and both instances occurred in the external dataset; none were observed in the benchmark or silent trial cohorts. All non-concordant cases with their error classifications are provided in **Supplementary Table 11** for review.

Third, as the reviewer notes, "leukemia" spans a broad diagnostic spectrum. **Figure 5** (external validation) documents concordance across the full entity landscape including AML, ALL, CML, MDS, MPN, and aplastic anemia, demonstrating that the system handles this heterogeneity.

We have revised the Discussion to explicitly acknowledge that concordance rates must be interpreted alongside the error type distribution and that the treating physician remains the final arbiter of clinical decisions.

Comment 3

How does HemaGuide deal with cases where the diagnosis is not clear based on clinical and pathologic review?

HemaGuide implements a cascading fallback architecture for diagnostically ambiguous cases. When the entity classification module does not recognize a hematological diagnosis (either because the diagnosis is pending, ambiguous, or falls outside the system's current entity list) it assigns a generic fallback classification rather than forcing an incorrect entity assignment. Cases with molecular genetics data are routed to Molecular Mode regardless of entity classification certainty. Non-molecular fallback cases are automatically routed to Advanced Mode, where the similarity search operates without entity filtering: the system queries the Clinical Decision Memory across all disease groups using only the clinical history embedding vector, thereby broadening the evidence base to capture relevant precedents spanning multiple diagnostic categories. If no retrieval source meets the configured similarity threshold and literature searches return no relevant results, the system degrades to a plain LLM generation mode (flagged as such in the output to the user), transparently reporting that no supporting evidence could be identified. This design ensures that diagnostically uncertain cases are never forced into entity-specific guideline pathways, while making the degree of available evidentiary support explicit to the reviewing clinician. Further, we have added **Point-by-Point Fig. 1** to illustrate this fallback behavior on a representative incomplete-input case and contrasted standard and degraded-input pipelines side by side.

We have added a dedicated subsection to the Methods ("Handling of diagnostically uncertain cases") describing this architecture.

Comment 4

What is the input information for HemaGuide? It seems that the input cases and tumor board information came from patients at Heidelberg University Hospital based on the Methods but this is not clearly described in the Results section.

We thank the reviewer for identifying this ambiguity. *HemaGuide* accepts routine unstructured tumor board briefing summaries in Microsoft Word (.docx) or Adobe (.pdf) format the standard document types used for tumor board preparation at our institution and across many tertiary centers worldwide. Documents are ingested manually via batch upload through the graphical interface or command-line processing and this worked for documents of our own institution and the external cohort documents which differed substantially in structure and wording; the system does not currently integrate with electronic health record (EHR) systems. We have clarified this in both the Results and Methods sections, specifying the input format, the institutional origin of the data, and the manual ingestion workflow.

Comment 5

Related to the above, I imagine the authors want this tool to be disseminated for use beyond the institution where this was created but it is not clear to me from the text if that is the goal of publishing this study versus proof of concept that such a tool with utility can be created.

The primary aim of this study is to provide a rigorous proof of concept that a locally deployable, case-grounded LLM agent can deliver clinically meaningful decision support across the breadth of hematological malignancies, and that this is achievable with commodity hardware and open-weight models, without requiring proprietary cloud infrastructure or institutional IT integration. The external validation on 555 independent cases from a second academic center and the prospective silent trial provide initial evidence that the system's decision logic transfers beyond the development institution.

Architecturally, we designed *HemaGuide* to be adaptable: guideline flowcharts and Clinical Decision Memory content can be replaced with institution- or country-specific equivalents, and the model backbone is swappable. Demonstrating transferability across health systems with different drug approval landscapes, guideline frameworks, and resource constraints is a key next step that we now discuss explicitly in the revised manuscript. At its current stage, *HemaGuide* is a research tool intended for investigator-supervised evaluation, not a certified medical device.

Comment 6

Practice recommendations often differ by country and availability to approved therapies may differ by country. How is this handled by HemaGuide?

HemaGuide's guideline repository is modular: we have manually curated 39 hematological disease entity-specific flowcharts that encode diagnostics and treatment algorithms derived from national and international guidelines and institutional SOPs (**Point-by-Point Note 1**). These flowcharts can be replaced with country- or institution-specific equivalents (e.g., NCCN guidelines for US deployment) without modifying the underlying agent architecture. Similarly, the Clinical Decision Memory can be populated with local tumor board cases to reflect regional treatment patterns and trial availability.

For the present study, all evaluations used European guideline flowcharts because concordance was measured against decisions from German academic tumor boards. We have implemented an automated guideline versioning mechanism that fetches timestamps from the guideline websites for all flowcharts in use and compares them against local versions, alerting users when updates are available (**Point-by-Point Fig. 3**). This can be triggered via a single click in the *HemaGuide* interface. We recommend quarterly checks aligned with our institutional SOP review cycles.

Comment 7

The example case in Figure 3d was helpful to see but I was surprised to see no mention of patient age, functional status, or prior treatments — which are essential in clinical decision making.

We agree that the original depiction of the extraction output in Figure 3d was incomplete. In response, we have revised the extraction pipeline to explicitly surface patient age, ECOG performance status, and secondary diagnoses as dedicated fields in the structured case representation. Prior therapies are no longer embedded as unstructured prose within the clinical history narrative but are parsed into a structured, line-by-line therapy summary as a separate extraction step. The updated **Figure 3d** reflects this improved, actual output of the LLM, and **Extended Data Figures 3-4** provide additional end-to-end case examples that illustrate how all clinically critical contextual parameters are captured and made available to the model prior to recommendation generation.

Comment 8

The examples of cases evaluated all seem to rely on mutational information to make recommendations about treatment plans. [...] However, only a minority of approved therapies for patients with hematologic malignancies are based on genetic factors (for example, almost none of the successful therapies in myeloma are linked to a specific gene mutation).

We appreciate this important clarification and agree entirely with the reviewer's premise: the majority of approved therapies in hematological malignancies are selected on the basis of disease stage, patient fitness, prior treatment history, and comorbidities rather than specific somatic mutations. *HemaGuide's* architecture reflects this reality through its three-mode design. *Guideline Mode* and *Advanced Mode* (which handle the large majority of cases) make recommendations based on clinical parameters, prior treatment lines, toxicities, comorbidities, and treatment history, independent of molecular findings. *Molecular Mode* is triggered only for molecular tumor board consultations, where the clinical question centers on whether a somatic variant is pathogenic and potentially targetable, which we believe represents a distinct clinical scenario with a correspondingly distinct decision-making workflow.

The concentration of molecular examples in the original manuscript reflected the fact that *Molecular Mode* has the most visually demonstrable computational pipeline (variant scoring, database queries, literature retrieval). We have now added end-to-end examples for *Guideline Mode* (**Extended Data Fig. 3**: relapsed B-ALL case decided entirely on clinical parameters) and Advanced Mode (**Extended Data Fig. 4**: relapsed PMBCL after multiple immunotherapy lines, where clinical trial enrollment and alternative bispecific antibody therapy are recommended based on treatment trajectory and precedent cases - not molecular findings).

Regarding clinical trial eligibility, we acknowledge that the current system does not maintain a registry of open clinical trials because we believe this would make keeping the recommendations up-to-date less feasible as an integrated registry would need to be manually updated. However, trial recommendations arise through three channels: mention in guideline flowcharts (where locally available trials can be encoded), citation in retrieved PubMed/Crossref literature, and the LLM's training knowledge (which may eventually be outdated). We recommend that institutions encode their locally available trials in the entity-specific flowcharts to ensure up-to-date trial recommendations, and we discuss this limitation in the revised manuscript.

Referee #2

Comment 1

The primary clinical performance claims rely on an evaluation of only 30 high-complexity cases. This sample size is insufficient to validate the generalizability and robustness of the system across the diverse and heterogeneous landscape of hematological oncology.

We agree that 30 benchmark cases alone would be insufficient to support generalizability claims. In the revision, we have substantially expanded the evidence base across three complementary evaluation tiers:

(1) The benchmark cohort has been increased by 50% to 45 high-complexity cases (15 per entity group), deliberately enriched for rare and complex clinical scenarios to maximize the discriminatory power between model configurations. These cases serve a specific purpose: to quantify the performance delta between plain LLMs and HemaGuide across six foundation models, not to estimate population-level concordance.

(2) An external validation on 555 independent, de-identified tumor board cases from Munich University Hospital (LMU) spanning 47 distinct hematological entities achieved 81.8% concordance without any re-optimization of flowcharts, prompts, or the original Clinical Decision Memory (**Fig. 5**). This cohort covers the full breadth of malignant hematology encountered in tertiary centers.

(3) A prospective one-month silent trial on 64 consecutive, unselected cases processed in parallel to clinical routine achieved 82.8% concordance (**Fig. 6**), confirming that performance is maintained under real-time conditions without retrospective case selection and across all incoming heme/onc disease entities of that month.

To standardize concordance assessment across these cohorts, we developed the Tumor Board Concordance Score (TBCS), a 10-point composite metric capturing therapy agents, therapy intention, and patient-adapted dosing/risk integration (**Fig. 5c**). Inter-rater reliability was excellent for the external validation ($\kappa = 0.934$) and substantial for the silent trial ($\kappa = 0.795$).

Comment 2

The benchmark compares HemaGuide (an agent with retrieval and tool-use layers) against plain LLMs that lack access to any external evidence. This experimental setup fails to isolate whether the observed performance gains are a result of the proposed "agentic routing" or simply the addition of external data. Ablation studies removing specific layers are necessary to justify the system's complexity.

This is an excellent point and we fully agree that the original evaluation did not isolate the contributions of individual components. We have now conducted a comprehensive ablation study (**Fig. 2e**) with the following design:

We defined 11 ablation levels (L0-L10), ranging from a plain LLM baseline (L0; no tools, no additional information) through isolated data layers (L1: guideline flowcharts only; L2: Clinical Case Memory only; L3: literature retrieval only; L4: molecular tools only) and progressively enriched combinations (L5-L9; adding contextual enrichment and LLM-based intelligence to the data layers) to the full agent with autonomous routing (L10). Fifteen cases (five per routing type: Guideline, Advanced, Molecular) were processed at each ablation level, yielding 165 evaluation combinations. All outputs were independently

scored by two hematologists (inter-rater reliability: Cohen's κ = 0.849 for binary concordance, Pearson r = 0.919 for continuous scores).

The results reveal that no single component is sufficient across all case types; instead, concordance gains are strongly routing-type-dependent. The plain LLM baseline achieved 0% concordance across all 15 cases. For guideline-amenable cases, flowcharts alone (L1) restored full concordance (100%), whereas neither the Clinical Case Memory nor molecular tools provided additional benefit. For advanced cases requiring precedent-based reasoning, the combination of Clinical Case Memory, literature retrieval, and contextual enrichment (L8) was required to reach 80% concordance. For molecular cases, the molecular interpretation toolkit (L4) drove the primary performance gain, and the full molecular pipeline (L9) achieved 100% concordance. The full agent with autonomous routing (L10) achieved 87% concordance overall, confirming that the system's multi-modal architecture is justified by the heterogeneity of clinical scenarios rather than by any single dominant component.

Comment 3

It is unclear how the authors ensured strict physical and temporal separation between the retrieval substrate and the evaluation queries. Explicit documentation of overlap control is required.

We have implemented a patient-level de-duplication filter that checks the unique, fully anonymized case identifier before evaluation runs, excluding all tumor board documents from the same patient (across different time points and case identifiers). This prevents both retrieval of the query case itself and retrieval of temporally adjacent cases from the same patient's trajectory.

For the benchmark cohort, overlap is structurally precluded: benchmark cases were manually anonymized and transformed (age variation, date shifting, laboratory value approximation, therapy detail modification) to prevent re-identification and enable use with external LLM APIs (**Point-by-Point Fig. 2**). These anonymized cases were evaluated against an anonymized Clinical Case Memory (**Supplementary Note 1**), not against the original institutional case memory.

For the external validation (555 LMU Munich cases), overlap is precluded by the design of the validation: the Clinical Decision Memory was derived entirely from Heidelberg cases, while the evaluation cases originate from an independent institution with no shared patient identifiers.

For the silent trial (64 consecutive cases), the patient-level de-duplication filter was applied at runtime, ensuring that any prior tumor board documents from the same patient were excluded from the retrieval pool before similarity search. We have documented this overlap control strategy in the Methods section.

Comment 4

The molecular validation is restricted to 70 single-nucleotide missense variants. In hematological malignancies, structural variants (SVs), fusions, and copy-number alterations are fundamental to clinical stratification.

We agree that structural variants, fusions, and copy-number alterations are clinically essential in hematological malignancies, and we appreciate the opportunity to clarify how the system handles these alterations. The 70-variant benchmark specifically evaluates the automated Horak *et al.*

ClinGen/CGC/VICC oncogenicity classification (1), which applies a point-based scoring framework designed for sequence variants. This module is not the system's only mechanism for incorporating genomic information.

Cytogenetic and structural findings are processed through three complementary pathways:

Extraction: The extraction module preserves cytogenetic findings (FISH, karyotype) of the case and propagates them to all downstream modules. For *Molecular Mode* cases, a dedicated extraction step parses FISH and cytogenetic findings into structured records with fields for aberration type, clone percentage, copy number, and fusion partner.

Guideline Mode: Treatment-relevant SVs, fusions, and CNAs are encoded as therapeutic routing determinants in the guideline flowcharts. For example, t(15;17)/PML::RARA triggers the ATRA+ATO pathway in APL; BCR::ABL1 transcript type and resistance mutations govern TKI selection in CML; del(17p)/TP53 status mandates specific treatment combinations in CLL; and IPSS-R karyotype groups determine therapy intensity in MDS. The LLM receives all extracted molecular findings alongside the complete flowchart.

Molecular Mode: Beyond the Horak *et al.* point-based classification (which itself is not limited to missense variants but also scores nonsense, frameshift, canonical splice-site, stop-loss, and in-frame insertion/deletion variants), a rule-based post-processing module applies entity-specific risk classification schemas to cytogenetic findings for consistency with established prognostic frameworks including R-ISS for myeloma and ELN 2022 for AML.

Comment 5

The evidence for clinical utility is primarily based on subjective Likert-scale scoring from a small group of clinicians. The manuscript would be significantly strengthened by the inclusion of inter-rater reliability metrics. Furthermore, without data from a silent trial or a real-world deployment setting, the claims regarding workflow benefits remain speculative.

We fully agree and have addressed both concerns.

Regarding **inter-rater reliability**, we now report IRR metrics across all evaluation settings. For the Likert-based benchmark scoring (Q1-Q6 across model configurations), Cohen's weighted κ ranged from 0.737 (Q1, concordance) and 0.682 (Q2, guideline compliance) to 0.178-0.315 for the more subjective dimensions (Q3–Q6). The lower agreement on subjective dimensions (reasoning quality, clinical utility) is expected given the inherent subjectivity of these constructs and the small number of raters, and is consistent with published IRR ranges for analogous clinical AI evaluations(2–4). Importantly, for the dimensions most critical to clinical validity (concordance and guideline compliance), agreement was substantial.

For the external validation (555 cases), the Tumor Board Concordance Score showed excellent inter-rater reliability (κ = 0.934). For the ablation study (165 configurations), binary concordance agreement was κ = 0.849 and continuous score agreement reached Pearson r = 0.919.

Regarding **prospective evidence**, we have now conducted a one-month silent trial (**Fig. 6**) in which *HemaGuide* processed consecutive, unselected tumor board cases one day before the scheduled conference, with the multidisciplinary tumor board blinded to *HemaGuide* output. Overall concordance was 82.8%

(53/64 evaluable cases). While this is a preliminary study limited in scale and duration, it provides initial evidence that concordance is maintained under real-time conditions without retrospective case selection.

Comment 6

The authors should provide quantitative evidence of the system's generalizability, such as a hold-out validation on an external dataset or a stress test in a resource-constrained setting.

We now provide quantitative generalizability evidence through the external validation on 555 independent cases from Munich University Hospital (LMU, **Fig. 5**), achieving 81.8% concordance across 47 hematological entities without any re-optimization. This cohort was processed with the identical *HemaGuide* configuration (same flowcharts, same Clinical Decision Memory, same model backbone) used for the original institution (Heidelberg University Hospital) evaluations, which be believe confirms that the system's decision logic is not overfit to a single institution's therapeutic style or trial portfolio.

We acknowledge that both validation sites are German tertiary-care centers with comparable therapeutic access. Transferability to resource-constrained settings or non-European health systems remains to be demonstrated. The system's local deployment capability (running on commodity hardware at approximately \$0.003–\$0.008 per case) represents a potential advantage for resource-limited settings, but this has not been formally evaluated.

Comment 7

The entire reasoning pipeline depends on the accurate conversion of unstructured documents into a 10-field JSON schema, yet the manuscript lacks a formal evaluation of this critical upstream module. The authors must report field-level metrics and provide an error propagation analysis.

We have now performed a formal evaluation of the extraction module (**Point-by-Point Table 1**). Two clinical experts independently rated field-level outputs across 45 cases using a four-point ordinal scale -−1 = false extraction, 0 = missed, 0.5 = partial, 1 = correct). Inter-rater reliability was substantial (Cohen's κ = 0.886). Applying a strict criterion where any extraction rated below 1.0 by either rater is counted as a failure, we obtained a macro-F1 of 0.9896, based on 360/370 (97.3%) field extractions rated as fully correct by both raters. The remaining 2.7% were rated as partially correct; no false extractions (hallucinated content) or complete field omissions were observed.

To assess error propagation, we simulated extraction failures in the three most clinically consequential fields: main_diagnosis, secondary_diagnoses, and history (prior treatments). For each field, we systematically removed its content from correctly extracted cases and re-ran the full agent pipeline. Removing the history section caused the model to default to Guideline Mode and treat the patient as newly diagnosed in all tested cases, which is a predictable failure mode. Removing secondary diagnoses altered the recommendation only when at least one secondary diagnosis had direct treatment implications (e.g., cardiac comorbidity affecting anthracycline eligibility). Removing the main diagnosis had the most severe impact, impeding correct flowchart selection and degrading performance toward the quality of the plain LLM baseline (**Point-by-Point Fig. 1**). These results confirm that extraction accuracy is a critical determinant of downstream decision quality and that the extraction module's empirical accuracy (macro-F1 = 0. 9896) provides a sufficient performance floor for the current evaluation.

Comment 8

The current evaluation describes execution time only in general "minutes". The authors should provide a detailed performance profile, including p50 and p95 latency for each decision mode and the hardware requirements necessary to support local deployment.

We now provide comprehensive latency profiling across all 45 benchmark cases, stratified by decision mode and pipeline phase (**Extended Data Fig. 6**). Running locally on an Apple M3 Ultra (28-core CPU, 96 GB unified memory) with gpt-oss-120b under MXFP4 quantization via Ollama, the full agent pipeline (extraction + decision) completes in a median of 75.4 seconds (p95: 144.4 s; mean: 87.5 ± 36.8 s). The decision generation step alone achieves a median of 39.4 seconds (p95: 62.1 s), with *Guideline Mode* fastest (median 36.0 s, p95: 50.1 s) and *Molecular Mode* exhibiting the highest variability (median 45.2 s, p95: 122.9 s) due to multi-database variant annotation.

The hardware specification (**Extended Data Fig. 6e**) represents a commodity-grade workstation (Apple Mac Studio M3 Ultra, approximately €4,800) that runs inference using unified CPU/GPU memory on an ARM64 architecture. The minimum requirement is 96 GB unified memory (~65 GB for the model, ~15 GB for OS and tools). We observed 30-100 tokens/second during inference, compatible with pre-conference preparation workflows where recommendations are generated hours to a day before the tumor board convenes.

For comparison, we also profiled cloud-based deployment with GPT-5-nano (median 64.0 s, $0.0022/case) and GPT-5-mini (median 49.5 s, $0.0053/case). The local open-weight deployment achieves comparable latency at an effective cost of $0.003-$0.008 per case depending on utilization, with the advantage of complete data sovereignty.

Comment 9

The reliance on manually curated, plain-text flowcharts for guideline adherence raises significant scalability and maintenance concerns. The authors must define a formal governance mechanism, including specific triggers for updates and a regression testing protocol.

We have implemented an automated guideline versioning mechanism that addresses both scalability and maintenance. The system fetches the last-updated timestamp from the relevant guideline's websites for each guideline entity and compares it against the corresponding local flowchart's version date. This check can be triggered via a single click in the *HemaGuide* interface and generates an overview of all flowcharts with their concordance status (up-to-date vs. update recommended). We recommend a quarterly checking schedule in line with our own institutional SOP review cycles.

Regarding regression testing, the flowchart-per-entity architecture is already naturally isolated: each entity's guideline is encoded as an independent file, and changes to one entity's flowchart cannot introduce logic drift in others, as there are no cross-entity dependencies in the guideline processing pathway. For flowchart updates within an entity, the 45 benchmark cases and the associated evaluation framework provide a ready-made regression test suite that can be re-run by us and others after any modification.

We acknowledge that the current curation process is semi-manual, relying on clinician review of guideline updates with LLM-assisted translation into the flowchart format. Full automation of guideline-to-flowchart translation is a future direction that we discuss in the manuscript.

Comment 10

The manuscript uses a cosine similarity threshold of 0.8 for case retrieval but fails to specify the agent's behavior when no high-confidence precedents are found. A robust decision-support tool requires a formal abstention policy or behavior tree for low-confidence queries.

We have revised the similarity retrieval strategy and implemented a formal degradation cascade. First, the cosine similarity threshold was lowered from 0.8 to 0.7 based on empirical analysis of our Clinical Decision Memory, where the original threshold was overly restrictive. The system now over-retrieves by a factor of 3 (i.e., retrieves up to 9 candidates when 3 similar cases are desired) and applies a second-level LLM-based reranking that accounts for therapy-line distance and drug-class overlap, providing finer-grained case matching than embedding similarity alone.

Second, the system now reports the evidence basis across all three retrieval channels individually: 1) similar cases (CCM), 2) PubMed literature, and 3) conference proceedings (Crossref), showing how many sources were retrieved and how many were retained after relevance filtering (e.g., cases=2/5, PubMed=3/3, Crossref=0/2). When all channels yield no relevant results, the system degrades to plain LLM generation (effective_mode=PLAIN) and transparently flags the absence of grounding evidence in the output to the user. Each decision carries structured fields documenting the evidence status and any fallback events.

Comment 11

All data comes from Heidelberg University Hospital, covering just six cancer types. Can the authors discuss how results might differ in other populations or resource settings, what happens with rare cancers not in the training data, and whether multi-site validation is planned?

The external validation on 555 cases from Munich University Hospital (LMU, **Fig. 5**) now provides quantitative evidence that the system generalizes beyond the development institution and achieves 81.8% concordance across 47 distinct hematological entities, which is substantially broader than the six entity groups used for benchmarking. For rare entities not represented in the Clinical Decision Memory, the system's fallback behavior routes cases to Advanced Mode with cross-entity retrieval and literature search, or degrades to plain LLM generation when no relevant evidence is found, while transparently flagging this state.

We have added a dedicated paragraph to the Discussion addressing how results might differ in other settings. Both validation sites are German tertiary-care centers with comparable therapeutic access; transferability to institutions with different drug approval landscapes, guideline frameworks, or resource constraints remains to be demonstrated. The system's architecture is designed for adaptability (replaceable flowcharts and case memory), but this adaptability has not yet been validated outside the German academic setting. Multi-center validation across different health systems is a stated next step and the authors plan to initiate a formal clinical trial to prospectively test *HemaGuide* as a decision assistance tool for heme/onc tumor boards.

Comment 12

I did not see much discussion of errors or hallucinations. Did HemaGuide get anything wrong on the 30 test cases? For the molecular variant calls, what are the false positive and negative rates?

We have performed a comprehensive error analysis across all evaluation cohorts (**Fig. 6e**, **Supplementary Table 11**) . Across all evaluated cases (45 benchmark, 64 silent trial, 555 external validation cases rated by entity-group-level concordance), hallucinations were observed in exactly 2 cases (mean 0.7% ± 0.7% SEM across three cohorts), both occurring in the external dataset:

Case 1 (ALL, 79 years): The agent fabricated a BCR-ABL-positive B-ALL subtype for a patient with T-ALL relapse and referenced prior imatinib therapy that had not occurred. This constitutes a hallucination of molecular subtype, lineage, and treatment history.

Case 2 (MCL, 66 years): The agent fabricated prior CAR-T therapy and clinical findings (respiratory insufficiency, neurological deficits) to justify rejecting CAR-T, whereas the patient was being presented for first-time CAR-T consideration. This is a hallucination of both treatment history and clinical status.

No hallucinations were observed in the benchmark or silent trial cohorts. All non-concordant cases with structured error classifications (protocol mismatch, understaging, overstaging, undertreatment, overtreatment, sequencing error, contraindication non-recognition, hallucination) are documented in the Supplementary Tables.

For molecular variant classification, the system achieved sensitivity 0.88, specificity 0.84, PPV 0.83, and NPV 0.89 against the reference standard (**Fig. 3b**). No variant classified as (likely) oncogenic by expert consensus was downgraded to likely benign/benign by *HemaGuide*, indicating that clinically dangerous false-negative downgrades did not occur in this benchmark.

Comment 13

The clinician study compares decisions against tumor board consensus. Were the evaluating doctors blinded to the study aims? Why use single TB decisions rather than multiple expert opinions as the reference?

Evaluating physicians were not involved in the design or development of *HemaGuide* and had no prior exposure to its outputs. They were blinded to model identity and configuration throughout all evaluation experiments, with cases presented in randomized order. However, in the simulated practice study (**Fig. 4d-g**), residents who used *HemaGuide* as a decision support tool were aware they were interacting with an AI system, as this was inherent to the study design. They were not informed about which LLM backbone was used or how the system worked internally.

We use interdisciplinary tumor board consensus as the reference standard because it represents the highest routinely documented level of clinical deliberation. We acknowledge that tumor board decisions are not uniquely correct: inter-board variability is well documented, and decisions are shaped by local expertise, trial availability, and institutional culture. Using multiple independent expert panels as a reference standard would be methodologically preferable but was not feasible at the scale of our evaluation (555 external cases, 64 silent trial cases). The use of single-institution tumor board consensus as ground truth is a pragmatic limitation that may introduce systematic bias toward the therapeutic preferences and trial portfolio of the respective center, which we discuss explicitly in the revised manuscript.

The evaluation design, including rater credentials, blinding protocol, and case selection methodology, is now fully documented in the Methods section.

Comment 14

How fast does the system generate recommendations? Does it connect to existing hospital EMR systems, or does data need to be entered manually?

End-to-end latency on local hardware (Apple M3 Ultra, 96 GB) is detailed in **Extended Data Figure 6**: the full pipeline completes in a median of 75.4 seconds (p95: 144.4 s), with the decision generation step achieving a median of 39.4 seconds (p95: 62.1 s).

The system currently requires manual document ingestion: clinical documents (Word or PDF format) are uploaded via the browser-based interface or processed via command line. *HemaGuide* does not currently connect to electronic health record (EHR) or hospital information systems. This is a deliberate design choice for the current proof-of-concept stage, as EHR integration would require institution-specific interfaces and special regulatory and data protecion approvals. We discuss EHR integration as an important next step for future deployment studies.

Comment 15

The ClinGen-based scoring framework in Figure 3 is reasonable, but how well does the automated classification match expert manual review? Some inter-rater reliability statistics would strengthen confidence in the molecular mode.

The 70-variant benchmark (Fig. 3b-c) directly compares *HemaGuide's* automated classification against manually derived reference classifications from the ClinGen/CGC/VICC framework. At the categorical level (LB/B, VUS, LO/O), Cohen's κ was 0.645, reflecting substantial agreement. At the quantitative level (continuous point scores), intraclass correlation coefficients were 0.805 (ICC(3,1) consistency) and 0.806 (ICC(2,1) agreement), with Pearson $r = 0.837$ ($p < 0.001$) and Spearman $\rho = 0.885$ ($p < 0.001$). These statistics are now reported in the Results section. The strong quantitative agreement, combined with the absence of clinically dangerous false-negative downgrades (no oncogenic variant classified as benign), supports the system's suitability for automated pre-screening, while we emphasize that expert review remains necessary for final clinical decisions.

References

1. Horak P, Griffith M, Danos AM, Pitel BA, Madhavan S, Liu X, et al. Standards for the classification of pathogenicity of somatic variants in cancer (oncogenicity): Joint recommendations of Clinical Genome Resource (ClinGen), Cancer Genomics Consortium (CGC), and Variant Interpretation for Cancer Consortium (VICC). Genet Med. 2022 May;24(5):986–98.
2. Fabbri AR, Kryściński W, McCann B, Xiong C, Socher R, Radev D. SummEval: Re-evaluating Summarization Evaluation [Internet]. arXiv [cs.CL]. 2020. Available from: http://arxiv.org/abs/2007.12626
3. Casabianca JM, Beiting-Parrish M. Correcting human labels for rater effects in AI evaluation: An item response theory approach [Internet]. arXiv [cs.AI]. 2026. Available from: http://arxiv.org/abs/2602.22585
4. Ralla B, Biernath N, Lichy I, Kurz L, Friedersdorff F, Schlomm T, et al. How accurate is AI? A critical evaluation of commonly used large language models in responding to patient concerns about incidental kidney tumors. J Clin Med. 2025 Aug 12;14(16):5697.

Referee #3

Major Comment 1

Overall, this reads a bit too much like a spec report and is generally very difficult to follow. Much of the very detailed methods can be moved to supplement to put the focus on the research experiments, not the engineering. The introduction runs into the results with no separation.

We agree that the original manuscript structure prioritized engineering description over experimental results. In the revision, we have restructured the manuscript to clearly separate Introduction and Results sections. The Introduction now focuses on the clinical problem, the rationale for an agentic approach, and the study objectives. The Results section leads with experimental findings (benchmarking, ablation, external validation, silent trial, simulated practice study) and describes the system architecture only to the extent needed to interpret the data. Detailed methods have been moved to the Methods section and Supplementary Notes.

Major Comment 2

The discussion of how the system architecture was arrived at and optimized are not sufficiently reported. It is therefore not possible to assess if the architecture was overfit to the evaluated cases. There are also no ablation studies, and so the relative contributions of the different components of the system are not clear.

We have conducted a comprehensive ablation study addressing this concern (**Fig. 2e**; see also our response to Reviewer 2, Comment 2). The 11-level ablation across 15 cases (165 total evaluations) demonstrates that the observed performance is not attributable to a single component and that the gains are routing-type-dependent: guideline cases are fully resolved by flowcharts alone, advanced cases require the combination of case memory and literature, and molecular cases depend on the molecular toolkit. The full agent's 87% concordance reflects the autonomous routing mechanism's ability to select the appropriate tool combination for each clinical scenario. Importantly, the architecture was not optimized on the test cases: the system's design preceded the evaluation, and the same configuration was used across all evaluation settings including the external validation and silent trial.

Major Comment 3

More detail on the clinical data types used in the retrieval database and the evaluated cases is needed. Was there a development/test split? How were the 30 cases for evaluation selected and removed from the broader set used in retrieval?

The now 45 benchmark cases are anonymized, transformed cases derived from real complex clinical tumor boards . Because they were manually anonymized and transformed (age variation, date shifting, laboratory value approximation), they cannot match any case in the original Clinical Decision Memory and were never part of the retrieval substrate. For the benchmarking experiments with cloud-based (closed-source) models, these anonymized cases were evaluated against a correspondingly anonymized Clinical Case Memory. For the evaluations using local models (external validation, silent trial), the original institutional Clinical Decision Memory was used, with patient-level de-duplication enforced at runtime. The case selection

strategy (stratified sampling by entity and disease timepoint, with completeness and diversity checks) is now documented in **Point-by-Point Fig. 2**.

Major Comment 4

More information is needed on how the de-identified clinical content was generated/extracted from patient records. Presumably this requires a large amount of manual effort, which impacts the scalability of any approach relying on it.

De-identification was performed in two distinct contexts with different procedures, now fully documented in **Point-by-Point Fig. 2**:

For the Clinical Decision Memory (>2,000 cases): Cases were extracted from an institutional research database under an approved ethics framework. Clinical content was processed in pseudonymized form; the extraction module generates de-identified structured representations (diagnosis, therapy history, outcomes) from routine clinical documents. No patient-identifying information is stored in the Clinical Decision Memory.

For the benchmark cases (n = 45) and external validation cases (n = 555): A two-step de-identification process was applied: manual redaction of names, dates, locations, identifiers, and treating physicians, followed by manual review. For the benchmark cases, an additional active transformation step was performed (age variation, date shifting, laboratory value approximation, institutional context neutralization) to enable compliant use with external LLM APIs.

We acknowledge that a manual de-identification process would represent a scalability constraint. Automated de-identification pipelines are available but would require institutional IT integration and validation against local data protection requirements. However, in the envisioned deployment scenario (where *HemaGuide* runs locally and no patient data leaves the institutional network) the de-identification requirement is substantially relaxed, as the model processes clinical documents directly without external API transmission.

Major Comment 5

There are many claims without sufficient methods or results to contextualize them. For example, how did the expert hematologist determine clinical similarity, what are the credentials of this individual, and how was retrieved case value determined?

We thank the reviewer for pointing this out and to make sure readers can contextualize our benchmarking. In response, we have made several additions. **Point-by-Point Fig. 2** now details the rater credentials and evaluation design across all study components. We developed the Clinical Case Similarity Score (**Extended Data Fig. 2a**), a 10-point metric operationalizing expert similarity judgment across four dimensions: entity match (0-3 points), therapy phase concordance (0-2 points), prior therapy overlap (0-3 points), and patient age proximity (0-2 points; similarity defined as ≥8/10). Similarly, the Tumor Board Concordance Score (**Fig. 5c**) quantifies agreement between *HemaGuide* recommendations and ground-truth tumor board decisions across three dimensions: therapy agents, therapy intention, and patient-adapted dosing/risk integration. All retrieval evaluations, testing of convergence in external datasets and the silent trial were performed by two independent hematologists blinded to each other's assessments with inter-rater reliability

quantified across all experiments using Cohen's κ. When there was substantial discordance between the raters, a third board-certified hematologist was consulted to resolve it. All benchmarking evaluations were performed by 4 independent hematologists blinded to each other's assessments with inter-rater reliability quantified across all experiments using Cohen's κ.

Major Comment 6

[Individual data points in main figures, outputs of each model to be made available to reviewers.]

All original tumor board decisions and corresponding *HemaGuide* outputs (as well as plain LLM outputs for all model configurations) for every evaluated case are now provided in **Supplementary Note 1** and **Supplementary Table 7-9**. Individual data points are shown in all main figures where applicable.

Major Comment 7

[IRR for simulated study with the junior docs]

Inter-rater reliability for the simulated practice study is now reported in the Methods section. For the Likert-based evaluation (Q1-Q6), Cohen's weighted κ ranged from 0.737 (Q1) to 0.178 (Q4), with substantial agreement on the clinically most consequential dimensions (concordance and guideline compliance). Furthermore, we also provide the IRR for the Tumor Board Concordance Score, which was excellent for the external validation (κ = 0.934) and substantial for the silent trial (κ = 0.795).

Major Comment 8

How was the interface optimized? There is no report of any assessment of its usability.

We have conducted a structured usability evaluation (**Extended Data Fig. 7**). A questionnaire covering five domains (visual design, responsiveness/intuitiveness, data presentation, workflow efficiency, and overall satisfaction) using a 5-point Likert scale was administered to users with diverse professional backgrounds (MDs, biologists, bioinformaticians) and varying experience levels. The evaluation demonstrates high ratings across all domains, with particularly strong scores for intuitiveness (minimal training needed), workflow fit, and willingness to recommend the tool to colleagues. Participant demographics and domain-level results with individual data points are shown in **Point-by-Point Fig. 2**.

Major Comment 9

It would be interesting and also important, for implementation considerations, to report and compare costs (for API models) and latency across the models. Please also provide information on the compute hardware used to run the local models.

We now provide a comprehensive cost and latency comparison across three deployment configurations (**Extended Data Fig. 6**):

(1) **Local open-weight deployment** (gpt-oss-120b, Apple M3 Ultra, 96 GB): median decision latency 39.4 s (p95: 62.1 s); effective cost $0.003-$0.008 per case depending on utilization (8 h/day vs. 24 h/day), including hardware amortization over 3 years and electricity at household rates.

(2) **Cloud-based GPT-5-nano**: median decision latency 64.0 s (p95: 103.5 s); cost $0.0022 per case ($0.10 for 45 cases).

(3) **Cloud-based GPT-5-mini**: median decision latency 49.5 s (p95: 89.0 s); cost $0.0053 per case ($0.24 for 45 cases).

The local deployment achieves the fastest inference and, depending on utilization, competitive or lower per-case costs compared to the cheapest cloud API, with the decisive advantage of complete data sovereignty, meaning that no patient data leaves the institutional network. Hardware specification details are now provided in **Extended Data Figure 6e**.

Major Comment 10

It is hard to understand exactly how the human evaluation was conducted. How were the triplicate runs handled? What was the distribution across PGYs? How many senior attendings participated? Were the participants consented?

We have substantially expanded the Methods section to document the evaluation protocol in detail. The key design elements are:

Four resident physicians across the hematology/oncology postgraduate training spectrum (PGY-1, -2, -4, -5) independently evaluated all 45 benchmark cases. Case assignment to the *HemaGuide*-assisted vs. unassisted condition was determined by stratified randomization to ensure balanced distribution of entities across conditions. Cases were presented in randomized order. This within-subject design allows each resident to serve as their own control, which we believe addresses inter-individual variation in baseline clinical competence as a confounder.

For each model configuration, three independent runs per case were generated. Dimensions Q1-Q5 were then assessed; the three independent repeat outputs also enabled dimension Q6 (consistency) to be assessed across all three runs.

All participating physicians provided written informed consent.

The full evaluation schema, rater demographics, and case selection methodology are now documented in **Point-by-Poing Fig. 2**.

Major Comment 11

The discussion should mention that performance improvement for attendings has not been assessed and is an important future direction.

We thank the reviewer for this suggestion and have now added a dedicated paragraph to the Discussion acknowledging that the simulated practice study evaluated *HemaGuide's* augmentation effect exclusively

in resident physicians. Whether the system provides meaningful decision support for attending remains untested. We note that the observation of senior physicians performing below residents when assessing cases outside their subspecialty suggests a potential role for decision support even at the attending level, but this hypothesis requires formal evaluation in a prospective study and is stated as an important future direction.

Major Comment 12

The discussion would benefit from mentioning that best-in-class API models (GPT-5-mini) benefit less from HemaGuide integration versus the open source models, and what this might mean for future developments.

We have added this observation to the Discussion. The data show that the performance gap between plain LLMs and *HemaGuide*-embedded systems is largest for open-weight models (e.g., gpt-oss-120b: plain 3.21 ± 0.39 vs. *HemaGuide* 4.22 ± 0.06), while the highest-capacity closed-source models (GPT-5-mini) already achieve reasonably high baseline performance and show smaller absolute gains from agentic integration.

However, it is worth noting that even GPT5-mini (the highest-capacity closed-source model in our evaluation) did not perform particularly well in plain mode, with only 3.75/15 myeloma cases, 7.5/15 leukemia cases, and 6.25/15 lymphoma cases achieving a mean Q1 rating of ≥ 4 across raters (17.5/45 overall, 39%). By contrast, *HemaGuide* (with the embedded gpt-oss-120b model) reached this threshold in 12.5/15 myeloma, 11.5/15 leukemia, and 12.75/15 lymphoma cases (36.75/45 overall, 82%).

This pattern suggests that as foundation model capabilities continue to improve, the relative contribution of the agentic architecture may shift from compensating for model limitations toward providing value through institutional customization (local case memory, guideline encoding) and workflow integration (structured extraction, transparent reasoning traces) rather than raw reasoning augmentation. This has implications for the long-term design of these AI tools in medicine: the system's value proposition may increasingly center on data sovereignty, institutional specificity, and auditability rather than on outperforming the best available commercial models.

Minor Comment 1

I find the use of "substrates" to refer to documents for retrieval to be awkward and unnecessary.

We thank the reviewer for this suggestion and have accordingly replaced "substrates" with "corpora" throughout the manuscript.

Minor Comment 2

Figure 2D: Please order the LLMs in the same order for the HemaGuide section and the vanilla LLM section.

We thank the reviewer for this suggestion. The model order in **Figure 2d** is now consistent across the *HemaGuide* and plain LLM sections to facilitate direct comparison.

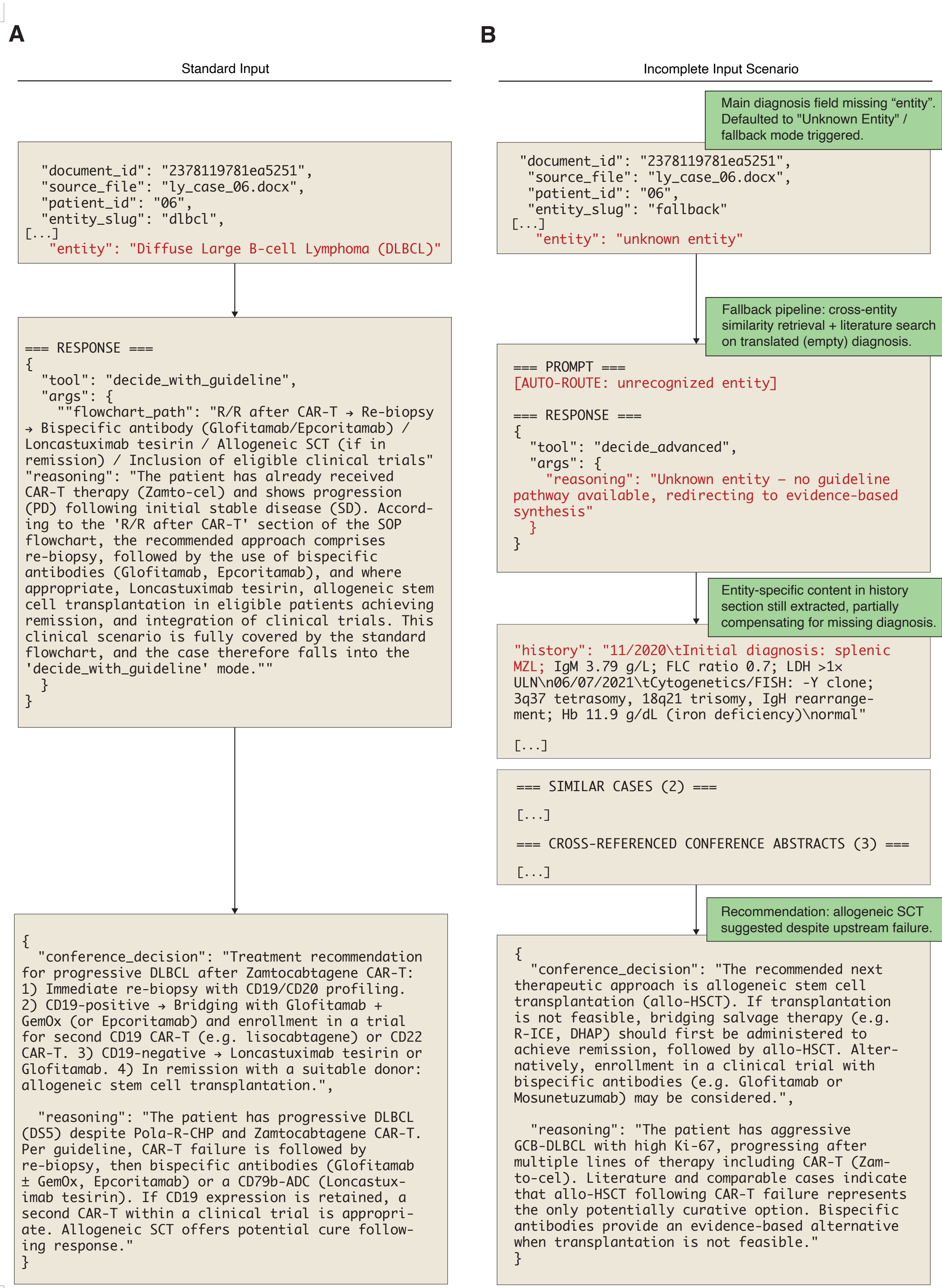

Point-by-Point Fig. 1

Point-by-Point Figure 1 | Diagnostic fallback architecture: standard versus incomplete input handling for diagnostically uncertain cases.

(A) Side-by-side comparison of *HemaGuide* processing for a DLBCL case under standard input (left) and a simulated incomplete input scenario in which the main diagnosis field is absent (right). Under standard input, the extraction module correctly identifies the entity (DLBCL), enabling config routing to the entity-specific pipeline; the agent selects *Guideline Mode* and traverses the R/R post-CAR-T flowchart to generate a structured treatment recommendation (re-biopsy with CD19/CD20 profiling, bispecific antibody bridging, and allogeneic SCT in remission). Under incomplete input, the entity classifier defaults to "unknown entity" / fallback mode, triggering automatic routing to *Advanced Mode* without entity filtering. The system performs cross-entity similarity retrieval and literature search using the clinical history embedding, and arrives at a clinically coherent recommendation (allogeneic SCT with salvage bridging) despite the missing diagnosis, demonstrating that entity-specific content preserved in the history section partially compensates for the absent diagnostic label.

(B) Corresponding JSON extraction outputs, agent routing prompts and responses, and final conference decisions for both scenarios, illustrating the cascading fallback logic from entity classification failure through cross-entity retrieval to evidence-grounded recommendation generation.

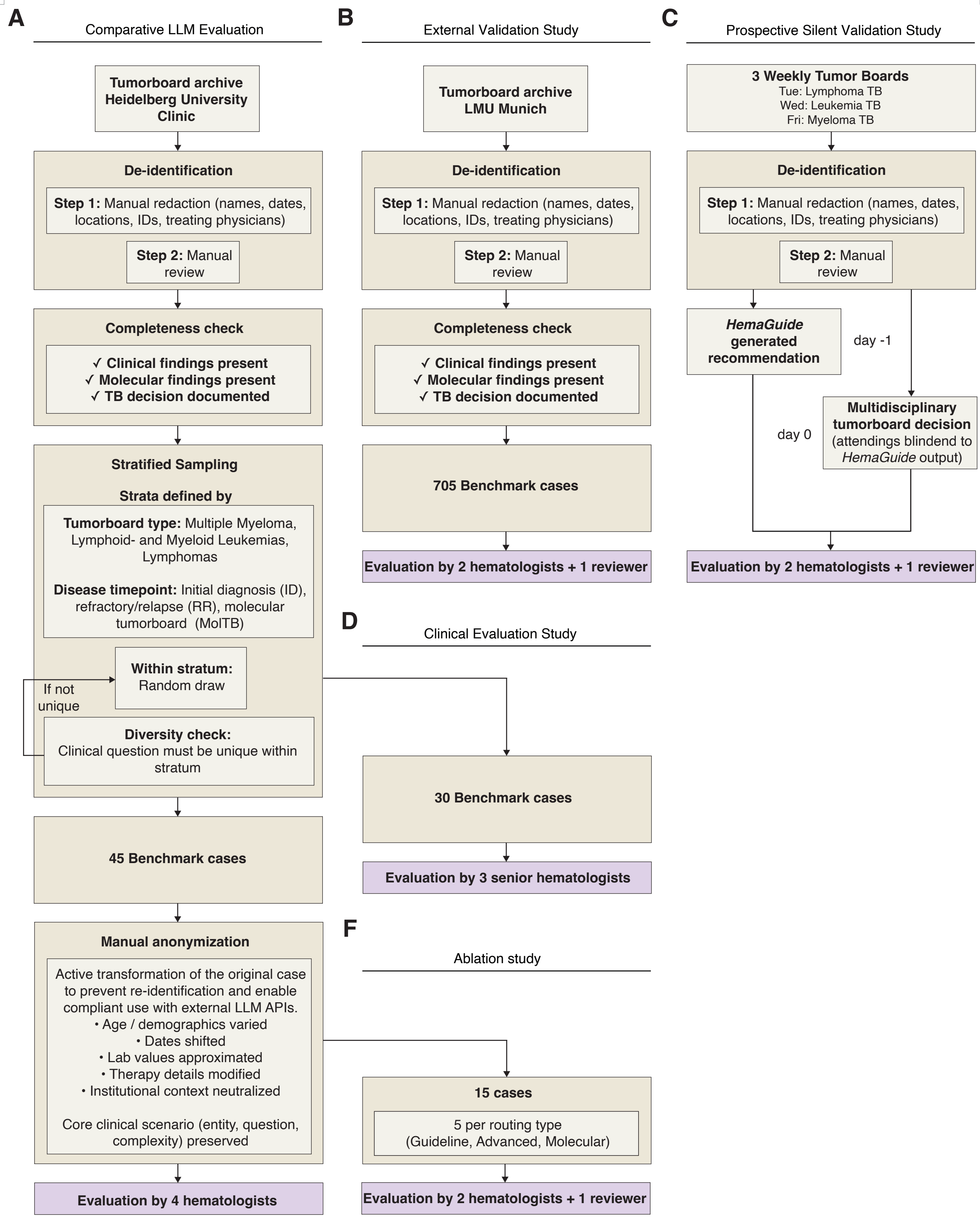

Point-by-Point Fig. 2

Point-by-Point Figure 2 | Study design overview: case selection, de-identification and evaluation workflow across all study components.

Schematic depicting the end-to-end workflow for case selection, de-identification, processing and evaluation across five study components. Benchmark cases (n = 45) were drawn from the Heidelberg University Hospital tumor board archive via stratified sampling by tumor board type (myeloma, leukemia, lymphoma) and disease timepoint (initial diagnosis, relapsed/refractory, molecular tumor board), with completeness and diversity checks applied before manual anonymization (age/demographics varied, dates shifted, laboratory values approximated, therapy details modified, institutional context neutralized; core clinical scenario preserved). These 45 cases were evaluated by four hematologists in the comparative LLM evaluation; a subset of 30 cases was used in the clinical evaluation study, assessed by three senior hematologists; and 15 cases (five per routing type: *Guideline*, *Advanced*, *Molecular*) were used in the ablation study, evaluated by two hematologists with a third reviewer resolving discordant assessments. External validation cases (n = 705 assessed for eligibility) from LMU Munich underwent a two-step de-identification process (manual redaction of names, dates, locations, identifiers and treating physicians, followed by manual review) with completeness verification, and were evaluated by two hematologists plus a third reviewer. The prospective silent trial used consecutive, unselected cases from three weekly tumor boards (Tuesday: lymphoma; Wednesday: leukemia; Friday: myeloma), with *HemaGuide* generating recommendations on day −1 and the multidisciplinary tumor board deciding on day 0, blinded to *HemaGuide* output; concordance was assessed by two hematologists plus a third reviewer. De-identification procedures and evaluator assignments for each study component are indicated.

Redacted

Point-by-Point Fig. 3

Point-by-Point Figure 3 | Automated guideline versioning mechanism for flowchart currency verification.

(A) Screenshot of the *HemaGuide* graphical user interface showing the guideline status dashboard, accessible via a single-click "Guideline check" button. The system automatically fetches the last-updated timestamp from European guideline websites for each disease entity and compares it against the corresponding local flowchart version date. Entities with matching timestamps are displayed with a green "Current" badge; entities where the online guideline has been updated more recently than the local flowchart are flagged with an orange "Update available" badge (illustrated here for Acute Lymphoblastic Leukemia, where the local version dates to 01/2026 and the online version to 03/2026). The dashboard reports the aggregate status (here, 29 of 30 flowcharts current) to enable rapid institutional oversight of guideline currency.
